# Root‐knot nematode genetic diversity associated with host compatibility to sweetpotato cultivars

**DOI:** 10.1111/mpp.12961

**Published:** 2020-06-17

**Authors:** Erika Asamizu, Kenta Shirasawa, Hideki Hirakawa, Hideaki Iwahori

**Affiliations:** ^1^ Faculty of Agriculture Ryukoku University Otsu Shiga Japan; ^2^ Kazusa DNA Research Institute Kisarazu Chiba Japan

**Keywords:** genome, GWAS, *Meloidogyne incognita*, race

## Abstract

Plant parasitic root‐knot nematodes (RKN) such as *Meloidogyne incognita* cause significant crop losses worldwide. Although RKN are polyphagous, with wide host ranges, races with differing host compatibilities have evolved. Associations between genotype and infection phenotype in *M. incognita* have not yet been discovered. In this study, 48 *M. incognita* isolates were collected from geographically diverse fields in Japan and their genomes sequenced. The isolates exhibited various infection compatibilities to five sweetpotato (SP) cultivars and were assigned to SP races. Genome‐wide association analysis identified 743 SNPs affecting gene coding sequences, a large number of which (575) were located on a single 1 Mb region. To examine how this polymorphic region evolved, nucleotide diversity (*Pi*) was scanned at the whole genome scale. The SNP‐rich 1 Mb region exhibited high *Pi* values and was clearly associated with the SP races. SP1 and 2 races showed high *Pi* values in this region whereas the *Pi* values of SP3, 4, and 6 were low. Principal component analysis of isolates from this study and globally collected isolates showed selective divergence in this 1 Mb region. Our results suggest for the first time that the host could be a key determining factor stimulating the genomic divergence of *M. incognita*.

## Graphical abstract

Genomic diversity level (*Pi*) of the 1 Mb SNP‐concentrated region segregated with the sweetpotato races. The majority of SP1 and 2 genotypes were heterozygotes, whereas SP3, 4, and 6 genotypes were mostly homozygotes.

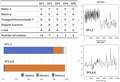

## 1 INTRODUCTION

Plant parasitic nematodes from the phylum Nematoda include several crop parasitic species. Of these, parasitic nematodes from the *Meloidogyne* and *Heterodera* genera are particularly harmful, causing an estimated 14% loss in annual crop production worldwide (Nicol *et al*., 2011). *Meloidogyne* spp. are commonly termed root‐knot nematodes (RKN) as they induce the development of knot‐like galls on the roots of infected host plants. RKN penetrate root cells and induce the production of giant cells close to the vascular bundle, from which the nematode obtains nutrients via an injected stylet. RKN exhibit sedentary lifestyles and reproduce asexually with the production of egg masses on the surface of plant galls. The four most damaging *Meloidogyne* species are considered to be *M. incognita*, *M. javanica*, and *M. arenaria* (all tropical species) and *M. hapla* (temperate) (Moen *et al*., 2009).

RKN are polyphagous and their host range encompasses most of the 250,000 known flowering plants (Trudgill and Blok, 2001). Nevertheless, infection phenotype varies among different RKN isolates, indicating that host range also varies. Testing of RKN isolates using a set of host plant species to categorize typical infection patterns into “races” was proposed in the North Carolina Differential Host Test (Hartman and Sasser, 1985). In this test, *M. incognita* isolates were categorized into four races according to reproduction patterns. All the isolates reproduced in pepper, watermelon, and tomato and did not reproduce in peanut, but isolates varied in their reproductive patterns in cotton and tobacco.

Sweetpotato (*Ipomoea batatas*) is an important food crop that is highly nutritious and a good source of dietary fibre. Sweetpotato is also used as a bioenergy crop via processing of starch obtained from the tuberous root. In Japan, the major area for sweetpotato cultivation region is found in the south‐west. Fields in this area are often infested with *M. incognita* isolates that can infect sweetpotato, causing substantial crop loss. Previous research showed that *M. incognita* isolates collected from geographically different fields showed distinct infection phenotypes against five sweetpotato cultivars, Norin‐1, Norin‐2, Tanegashimamurasaki‐7, Elegant Summer, and J‐red (Sano and Iwahori, 2005). Isolates were initially classified into nine sweetpotato (SP) races according to their infection patterns. Further research recently indicated that SP6 could be classified into SP6‐1 and SP6‐2 (Tabuchi *et al*., 2017).

Associations between genome‐wide nucleotide polymorphisms and RKN infection phenotypes have not been discovered to date. Host range is a complicated phenotype that can be analysed by methods such as quantitative trait locus (QTL) analysis or genome‐wide association study (GWAS). However, association techniques can be limited. For example, QTL analysis requires a mapping population, genetic maps, and DNA markers to allow association of genotype with phenotype. *M. incognita* is essentially a mitotic parthenogenic organism, and generation of genetically crossed populations is thus not possible. GWAS does not require production of a mapping population and can therefore be used for analysis of *M. incognita*. The genomes of 11 Brazilian *M. incognita* isolates were recently sequenced and shown to exhibit different host ranges according to the North Carolina Differential Host Test. However, attempts to associate host range with genetic factors were unsuccessful (Koutsovoulos *et al*., 2020).

In this study, a large number of *M. incognita* isolates was used to increase the possibility of identifying genetic markers associated with host range. Forty‐eight isolates were sequenced and phenotyped using five SP cultivars according to the SP race method. This resulted in the identification of 743 candidate single nucleotide polymorphisms (SNPs) with effects on gene sequences. Surprisingly, more than 77% of the SNPs were located on a single contig. Diversity within this region was clearly associated with SP race. The presence of a high number of SNPs within a single region suggests the presence of a hotspot region in the *M. incognita* genome that diversified during the host adaptation process. To our knowledge, this is the first report associating genotype with infection phenotype in *M. incognita*.

## 2 RESULTS

### 2.1 *M. incognita* phenotype analysis and infection race assignment

SP race infection phenotype was determined using five SP cultivars. Of the 48 *M. incognita* isolates that were assessed, phenotyping was successful for 32 isolates (Table 1). In total, 14, 7, 3, 2, and 6 isolates were classified as belonging to the SP1, SP2, SP3, SP4, and SP6 races, respectively. The remaining 16 isolates were excluded from further analysis as they either did not conform to typical SP race phenotype patterns or yielded inconsistent results.

**Table 1:** Phenotype test of the *Meloidogyne incognita* isolates using five sweetpotato cultivars

Cultivar	SP1	SP2	SP3	SP4	SP6
Norin‐1	V	V	V	V	V
Norin‐2	A	V	A	V	A
Tanegashimamurasaki‐7	A	A	V	V	V
Elegant Summer	A	A	A	V	V
J‐red	A	A	A	A	A
Number of isolates	14	7	3	2	6
ID of isolates	Chb_Ash001	Kch_Smnt001	Kmmt_Gs002	Ibrk_Tkb002	Kmmt_Gs007
	Fkok_Hkck001	Kgsm_Ky001	Kmmt_Gs005	Oknw_Ynsr001	Kmmt_Gs008
	Hkd_Mr002	Kmmt_Gs003	Kmmt_Msk002		Oknw_Isgk001
	Hkd_Mr003	Kmmt_Ots001			Oknw_Isgk002
	Kch_Ts001	Kmmt_Ymg002			Oknw_Ogm001
	Kmmt_Gs004	Mie_Ykic001			Ymgt_Unkwn001
	Kmmt_Gs006	Myzk_Hyg001			
	Kmmt_Gs009				
	Kmmt_Gs010				
	Kmmt_Ymg001				
	Myzk_Tn001				
	Ngt_Kjkw001				
	Oit_Tkd001				
	Ymgt_Unkwn002				

V, virulent; A, avirulent.

### 2.2 *M. incognita* genome sequencing and mapping to the reference genome

An *M. incognita* reference genome sequence was generated using a PacBio strategy. Approximately 300 ng genomic DNA with average length 63 kb was used for library preparation. In total, 34,000,000 long reads (15.4 Gb) were obtained and assembled into 193.1 Mb primary contigs consisting of 374 sequences with N50 of 974.8 kb and 10.7 Mb haplotigs (167 sequences with N50 of 72.3 kb). The primary contigs were designated as MINJ2. In the subsequent gene prediction, 31,206 genes were predicted in the MINJ2 sequences (Files S1, S2, and S3).

In total, 60.49 Gb Illumina reads were accumulated from 48 *M. incognita* isolates at an average of 1.26 Gb per isolate, equivalent to 7× coverage of the estimated 180 Mb *M. incognita* genome (Table S1). It was confirmed that the fold coverage did not affect mapping rate (Figure S1).

**Figure 1:**
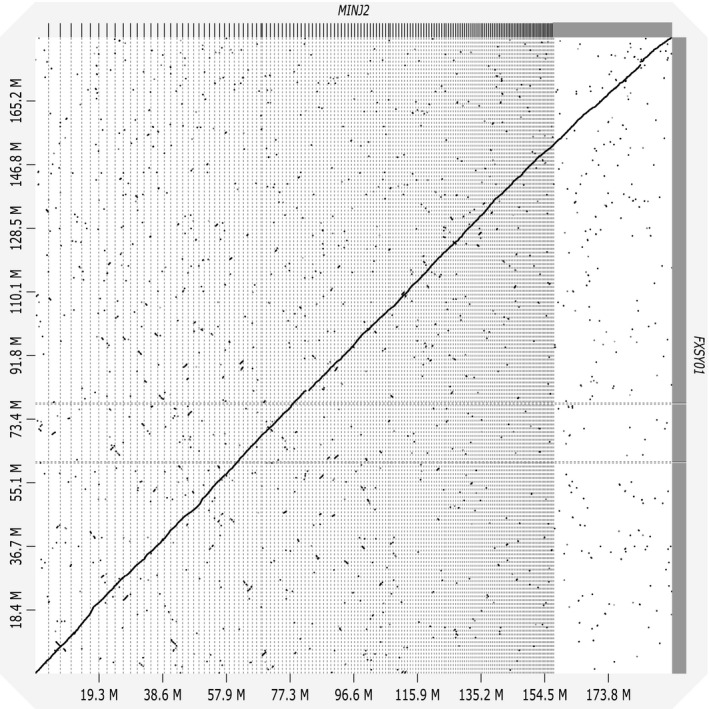
Comparison of genome structures between the previously reported *Meloidogyne incognita* genome (FXSY01) and the reference genome produced in this study (MINJ2). Dot plot analysis showed that the overall structures of the two assemblies were the same across the whole genome

The first reference genome of *M. incognita* was published in 2008 (Abad *et al*., 2008) and subsequently revised (accession GCA_900182535.1; Blanc‐Mathieu *et al*., 2017). To analyse the quality of our assembled reference genome, short reads were mapped onto both genome assemblies and mapping rates were compared. Mapping rates were similar (Figure S1), indicating that the structures of the two genome assemblies were also similar. The MINJ2 sequences covered the entire region of the published genome (Figure 1).

### 2.3 Genomic diversity among *M. incognita* isolates

**Figure 2:**
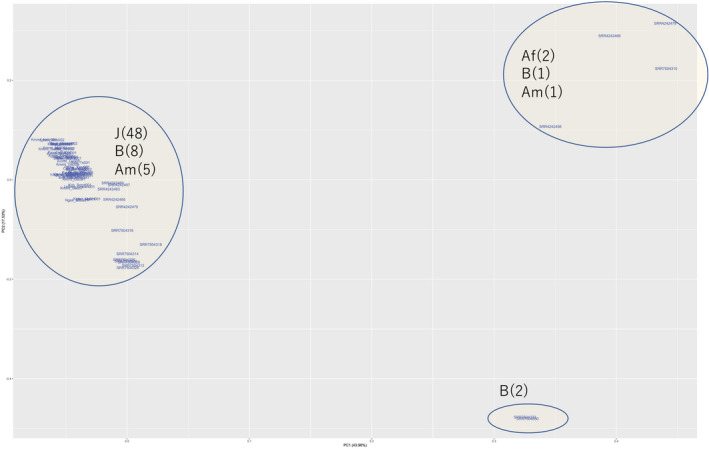
Principal component analysis of genome sequences of *Meloidogyne incognita* isolates of different geographical origins. Isolates from Japan (J) were clustered in a single group alongside eight isolates from Brazil (B) and five from the Americas (Am). The second group contained two isolates from Africa (Af), one from Brazil, and one from Anguilla (also included in Am). The third group contained two isolates from Brazil

To analyse the genomic diversity of *M. incognita* isolates with different geographical origins, sequence comparisons were performed with previously published genome sequences (Szitenberg *et al*., 2017; Koutsovoulos *et al*., 2020). Principal component analysis (PCA) divided *M. incognita* isolates into three main groups. The largest group included all 48 isolates from Japan, eight Brazilian isolates (SRR7504312, SRR7504314, SRR7504316, SRR7504318, SRR7504320, SRR7504324, SRR7504326, and SRR7504328), and five isolates from the Americas (SRR4242460, SRR4242463, SRR4242465, SRR4242467, and SRR4242470). The other two groups contained relatively few isolates. One group contained two African isolates (SRR4242456 and SRR4242469), one isolate from Anguilla (SRR4242479), and one isolate from Brazil (SRR7504310). The remaining group contained two Brazilian isolates (SRR7504322 and SRR7504330) (Figure 2).

### 2.4 Association of *M. incognita* genotype with infection phenotype

Genome‐wide association analysis comparing *M. incognita* genotypes and infection phenotypes was performed using the generalized linear model (GLM) implemented in TASSEL 5 (Bradbury *et al*., 2007). Extracted SNPs were correlated to virulence against cultivars Norin‐2, Tanegashimamurasaki‐7, and Elegant Summer. The impact of SNPs on coding sequences was assessed by SnpEff v. 4.3T (Cingolani *et al*., 2012). In total, 743 SNPs were identified that met the assessment criteria. Of these, one high‐impact SNP was identified that was predicted to remove the stop codon. Eleven moderate impact SNPs were identified that were predicted to result in missense mutations. Low impact effects (e.g., synonymous mutations, splice variants, and start/stop codon variants) were predicted for 43 SNPs. The remaining 688 SNPs were predicted to result in modifier mutations (Table S2). Interestingly, 575 of the 743 SNPs (77.4%) were located on a 1 Mb genomic region of contig MINJ2_005F.1.

### 2.5 Nucleotide diversity of the *M. incognita* genome

To identify the level of nucleotide diversity in the *M. incognita* genome, contigs were scanned using the site‐pi function of VCFtools v. 0.1.16 (Danecek *et al*., 2011). The result for each contig was shown graphically, indicating the moving average of *Pi*, with a 50 bp window size. Although the mean value of *Pi* was 0.15 over the whole genome, relatively extended high‐*Pi* (>0.3) regions of ≤500 kb were found on contigs MINJ2_001F.1 and MINJ2_0012F.1, and longer regions of approximately 1 Mb were found on contigs MINJ2_005F.1 and MINJ2_021F.1 (Figure S2).

**Figure 3:**
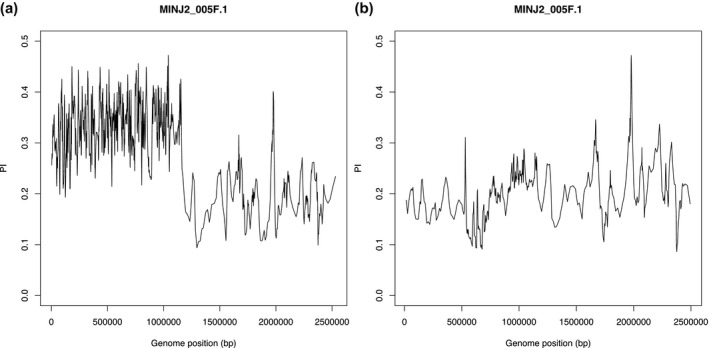
Comparison of nucleotide diversity (*Pi*) between sweetpotato races in contig MINJ2_005F.1. (a) *Pi* plot of races SP1 and 2, (b) *Pi* plot of races SP3, 4, and 6. The first 1 Mb region showed different *Pi* values between (a) and (b). The mean *Pi* value of the 1 Mb region in (a) was 0.34, while that of (b) was 0.19

A high proportion (77.4%) of the 743 SP‐related SNPs that affected gene sequences were located on the 1 Mb high‐*Pi* region on MINJ2_005F.1. To determine whether high *Pi* value segregated with race, site‐*Pi* analysis was performed with combinations of races. High‐*Pi* value (mean: 0.34) was detected in SP1 and 2, but not in SP3, 4, and 6 (mean: 0.19) (Figure 3). This phenomenon was specific to this contig: other high‐*Pi* regions did not segregate with the races (Figure S3).

**Figure 4:**
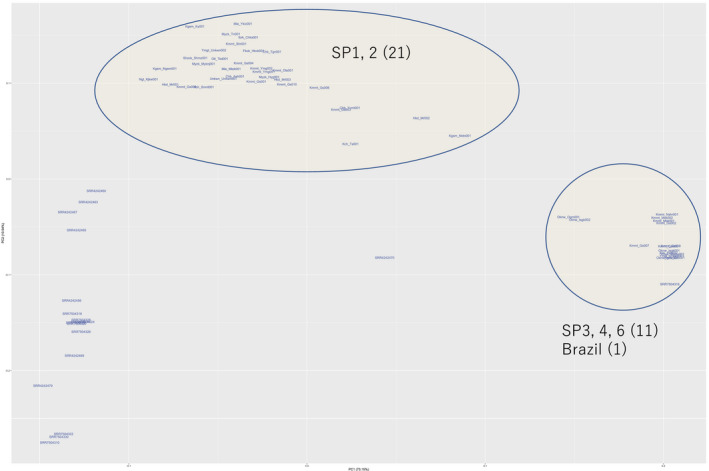
Principal component analysis of the 1 Mb high‐*Pi* region on MINJ2_005F.1. Races SP3, 4, and 6 clustered together alongside one isolate from Brazil. Races SP1 and 2 were scattered to form a large group, but no isolates from other continents were included

PCA was next performed with only the 1 Mb high‐*Pi* region to examine the evolution of the race‐associated genomic region (Figure 4), and showed separation of races SP1 and 2 from the cluster containing SP3, 4, and 6. With the exception of two isolates from Brazil, isolates from other continents were closely clustered. One exception (SRR7504316) belonged to the SP3, 4, and 6 cluster, and another (SRR4242470) was separated from all other isolates.

### 2.6 SNP genotypes

**Figure 5:**
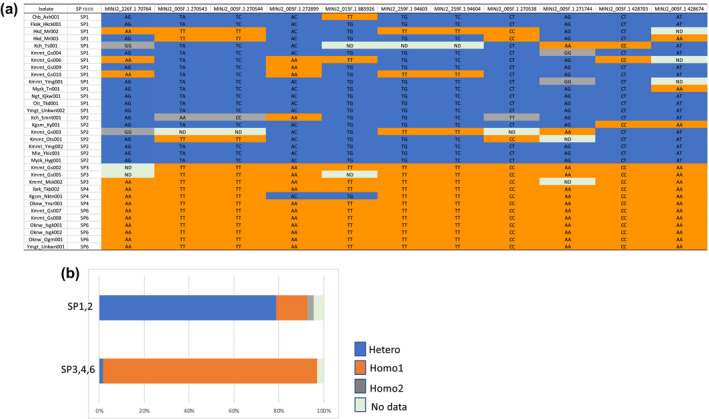
Genotypes of the significant single nucleotide polymoprohisms (SNPs) that correlated with virulence against sweetpotato cultivar Tanegashimamurasaki‐7. (a) List of SNP sites and genotypes. (b) Among the 221 SNP genotypes in SP1 and SP2, 182 (78.8%) were heterozygotes, 32 (13.9%) were SP3, 4, 6 type homozygotes, and 7 (3.0%) were the other type of homozygotes. Among the genotypes of 128 SNP genotypes in SP3, SP4, and SP6, 2 (1.5%) were heterozygotes and 126 (95.5%) were SP3, 4, 6 type homozygotes, while the other type of homozygote was not observed

The genotypes of one high‐impact SNP and 10 moderate‐impact SNPs that correlated with virulence against cultivar Tanegashimamurasaki‐7 were analysed (Figure 5). Among 221 SNP genotypes in SP1 and SP2, 182 (78.8%) were heterozygotes, 32 (13.9%) were SP3, 4, 6 type homozygotes, and seven (3.0%) were the other type of homozygotes. Among 128 SNP genotypes in SP3, SP4, and SP6, 2 (1.5%) were heterozygotes and 126 (95.5%) were SP3, 4, 6 type homozygotes, while the other type of homozygote was not observed.

### 2.7 Genes affected by SNPs

BLAST searches against the Refseq database were used to identify and characterize genes affected by SNPs. A total of 51 SNPs, comprising high‐impact (one SNP), moderate‐impact (11), and low‐impact (39) SNPs, were identified (Table 2). Removal of redundant SNPs identified for both the Tanegashimamurasaki‐7 and Elegant Summer phenotypes reduced the number of low‐impact SNPs from 43 (Table S2) to 39 (Table 2). Modifier SNPs are not shown in Table 2. Multiple SNPs affected MINJ2005Fg02303t1, which was predicted to encode a serine threonine protein kinase‐related domain containing protein, and MINJ2005Fg02312t3, which was predicted to encode a nuclear RNAi defective‐3 protein‐like protein. In addition, seven genes predicted to encode short proteins (<100 amino acids) were identified, six of which (MINJ2005Fg02274t1, MINJ2005Fg02275t1, MINJ2005Fg02276t1, MINJ2005Fg02304t1, MINJ2226Fg25258t1, and MINJ2259Fg26163t1) had no similarity to known sequences.

**Table 2:** Genes affected by SNPs

Annotated gene	Length (amino acids)	Trait^a^	SNP impact on gene sequences			BLASTP vs. RefSeq
			High	Moderate	Low	
MINJ2005Fg02249t1	163	TM	0	0	1	Vacuolar protein sorting‐associated protein 37B
MINJ2005Fg02274t1	88	TM	0	3	0	NA
MINJ2005Fg02275t1	76	TM	0	1	0	NA
MINJ2005Fg02276t1	80	TM	0	1	1	NA
MINJ2005Fg02281t1	89	TM	0	0	1	Chloride intracellular channel protein 6
MINJ2005Fg02303t1	430	TM	0	2	5	Serine threonine protein kinase‐related domain containing protein
MINJ2005Fg02304t1	74	TM	0	0	1	NA
MINJ2005Fg02307t1	187	TM	0	0	1	NA
MINJ2005Fg02311t1	176	TM	0	0	1	NA
MINJ2005Fg02312t3	947	TM	0	0	14	Nuclear RNAi defective‐3 protein‐like
MINJ2005Fg02325t2	374	TM	0	0	1	DNA‐(apurinic or apyrimidinic site) lyase
MINJ2005Fg02334t1	139	TM	0	0	1	Cleavage stimulation factor subunit 3
MINJ2005Fg02364t1	338	TM	0	0	1	Ezrin/radixin/moesin family protein
MINJ2015Fg05752t1	334	TM	0	1	0	Transcription initiation factor IIF, alpha subunit, putative
MINJ2015Fg05813t1	197	TM	0	0	1	Hypothetical protein L596_006851
MINJ2098Fg18485t1	954	N2	0	1	1	Unnamed protein product, partial
MINJ2226Fg25254t1	174	TM	0	0	1	NA
MINJ2226Fg25258t1	67	TM	1	0	0	NA
MINJ2226Fg25263t1	230	TM	0	0	1	Domain found in Dishevelled, Egl‐10, and Pleckstrin family protein
MINJ2226Fg25268t1	548	TM	0	0	1	MORN repeat protein
MINJ2226Fg25268t2	823	TM	0	0	1	MORN repeat protein
MINJ2259Fg26154t1	83	TM	0	0	1	Bacterial Fmu (Sun)/eukaryotic nucleolar NOL1/Nop2p domain and RNA (C5‐cytosine) methyltransferase family and tRNA (C5‐cytosine) methyltransferase, NCL1 family‐containing protein
MINJ2259Fg26159t2	415	TM	0	0	1	AGC/NDR protein kinase
MINJ2259Fg26160t1	5,102	TM	0	2	2	Hypothetical protein PRIPAC_47929
MINJ2259Fg26163t1	87	TM	0	0	1	NA

^a^ TM, virulent against cv. Tanegashimamurasaki‐7; N2, cv. Norin‐2.

**Figure 6:**
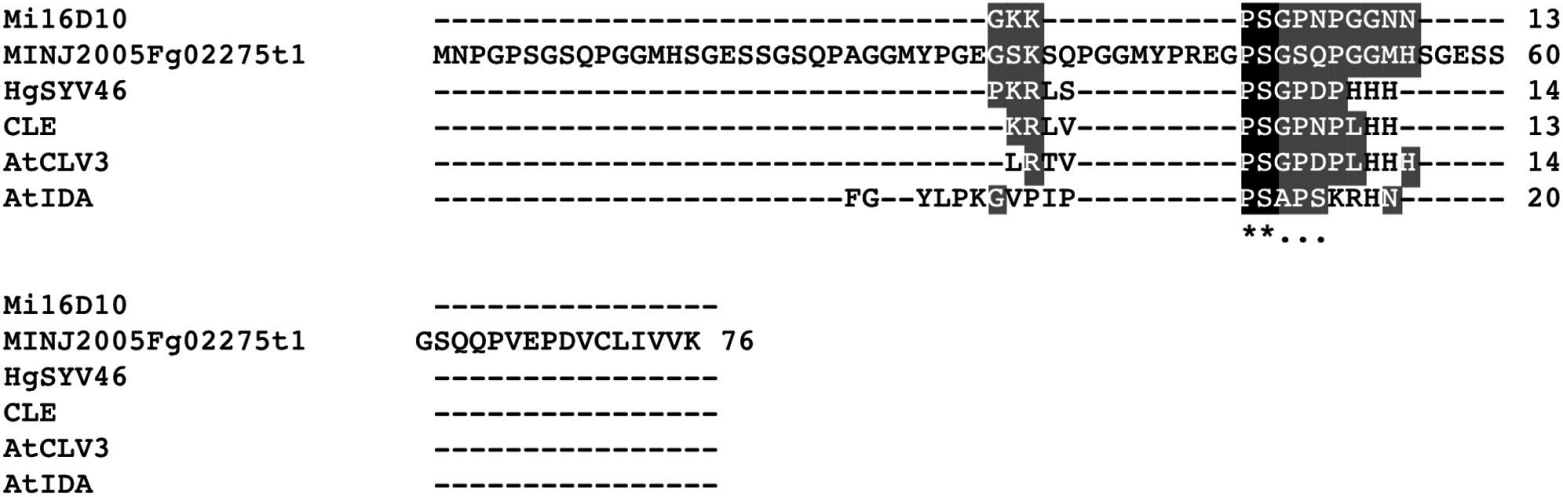
Alignment of MINJ2005Fg02275t1 and CLE motifs. Conserved C‐terminal CLE motifs were aligned by CLUSTALW. Mi, *Meloidogyne incognita*; Hg, *Heterodera glycines*; At, *Arabidopsis thaliana*. Mi16D10 (GenBank accession no. AY134435), AtCLV3 (AF126009), AtIDA (NP564941), HgSYV46 (AF273728), and CLE (the C‐terminal conserved motif of the plant CLE protein family) (Olsen and Skriver, 2003)

Active transcription of the six predicted genes of unknown function was confirmed by BLAST sequence searches against an *M. incognita* transcriptome data set (SRX2919273) encompassing several developmental stages (Choi *et al*., 2017). The predicted proteins were also assessed for the presence of signal peptide motifs CLAVATA3‐like (CLE) (Huang *et al*., 2006) and INFLORESCENCE DEFICIENT IN ABSCISSION (IDA) (Kim *et al*., 2018), and a CLE‐like motif was identified in the predicted protein encoded by MINJ2005Fg02275t1 (Figure 6).

## 3 DISCUSSION

Forty‐eight *M. incognita* isolates were collected from geographically diverse sweetpotato fields in Japan and their genomes sequenced. Previous studies have also sequenced multiple *M. incognita* isolates. Szitenberg *et al*. (2017) sequenced eight *M. incognita* isolates with geographical origins in Africa and the Americas. Recently, Koutsovoulos *et al*. (2020) published the genome sequences of 11 isolates collected across Brazil. The relatedness of these isolates and the 48 isolates from this study was assessed using PCA. The first component segregated six of the isolates from the remainder, and the second component further divided the six isolates into two groups. The 48 Japanese isolates and the remaining 13 isolates from other continents were closely clustered (Figure 2). These results indicate that *M. incognita* isolates in Japan exhibit very little genomic diversity, and that diversity is minimal even among some isolates from different continents.

Phylogenetic analysis was used to determine whether the geographical distribution of *M. incognita* isolates in Japan reflected genetic diversity (Figure S4). Three isolates obtained from the southernmost Okinawa islands (Oknw_Isgk002, Oknw_Ogm001, and Oknw_Ynsr001) were clustered into the same group. However, a fourth isolate from Okinawa, Oknw_Isgk001, was grouped with two other isolates from northern sites on the main island of Japan. Other parts of the phylogenetic tree indicated that, in general, there was no relationship between the genotype and the geographic origin of the isolates. Phylogenetic relatedness with SP races was also assessed but no clear relationship was observed (Figure S4), indicating that there was no association between genotypes and the infection phenotypes at the whole‐genome level.

To date, no reports have been published associating *M. incognita* genotype with infection phenotype. Various ranges of host compatibility have been observed as demonstrated by the North Carolina Differential Host Test (Hartman and Sasser, 1985), and SP races in this study, but the genetic mechanism underlying the variability in host range has not previously been elucidated. In this study, 743 SNPs with predicted effects on gene coding sequences were identified in a GWAS of SP phenotypes. Of these, 575 SNPs (77.4%) were located on a single 1 Mb genomic region. To structurally characterize the region, all genome contigs were scanned for nucleotide diversity (*Pi*) among isolates. Several regions were found that exhibited extended high‐*Pi* values, including the 1 Mb region with 575 SP‐related SNPs (Figure S2). A possibility that this concentrated SNP region resulted from a collapsed artefact of the assembly was eliminated by a coverage plot for the contig MINJ2_005F.1 together with the *Pi* (Figure S5). It was suggested that the infection phenotype was associated with the level of genetic diversity.

Genetic diversity is associated with evolution. In *Drosophila melanogaste*r, higher *Pi* populations tended to experience lower levels of extinction than lower *Pi* populations. In addition, nucleotide diversity explained evolutionary responses in productivity and body mass compared to expected inbreeding coefficients (Orsted *et al*., 2019). In this study, the *Pi* level of the SNP‐rich 1 Mb genomic region segregated with the SP races (Figure 3). Evolution of the SP‐associated genomic region among isolates of global origins was examined by PCA (Figure 4). SP1 and 2 were loosely clustered while the majority of isolates of SP3, 4, and 6 were densely clustered. One isolate from Brazil was found in the SP3, 4, and 6 group, but no isolates from other continents plotted close to the SP1 and 2 races. Isolates from other continents may possess different types of genetic diversity in this genomic region. These results were quite different from those obtained when PCA was performed to analyse genome‐wide SNPs (Figure 2), suggesting that genetic variation in this region may have evolved in response to exposure to environmental stimuli.

Genotypes of the significant SNPs indicated that SP1 and 2 possess a much larger ratio of heterozygotes than SP3, 4, and 6 (Figure 5). *M. incognita* is an obligate parthenogenesis species that reproduces clonally, and the divergence time from facultative meiotic parthenogenesis species (e.g., *M. hapla*) is estimated to be more than 43 million years ago (Castagnone‐Sereno, 2006). The heterozygous genotypes remaining in the *M. incognita* genome are considered to be imprints from amphimixis in the ancestral species. The homozygous genotypes of SP3, 4, and 6 were uniform, indicating that there was no exceptional allele. Races SP3, 4, and 6 are virulent against Tanegashimamurasaki‐7 while races SP1 and 2 are avirulent. These results suggest that fixing to the SP3, 4, and 6 homozygous genotype was important for adaptation to new hosts. It is not clear over which time scale this genetic selection occurred. Because the approximately 1 Mb region on MINJ2_005F.1 had a large number of SNPs associated with SP phenotypes, genes important for host infection are expected to be located in this region.

*M. incognita* is an obligatory mitotic parthenogenetic organism (Moen *et al*., 2009). It is generally believed that parthenogenetic species are evolutionary dead ends. The genome structure of *M. incognita* has been studied in detail to examine genomic plasticity. Resequencing of the *M. incognita* genome (Blanc‐Mathieu *et al*., 2017) increased the estimated assembled genome size to 183 Mb from the initial estimate of approximately 50 Mb (Abad *et al*., 2008). This increase was due to the successful separation of duplicated regions that developed from possible hybridization events in ancestral species. The duplicated genome gives rise to elevated gene copy numbers and redundancy, allowing selective transcription to enable adaptation to environment changes or different hosts. Our results showing genetic diversity in *M. incognita* are suggestive of an alternative adaptation strategy in this species. The genetic mechanisms underlying the generation of diversity within a particular genomic region remain to be discovered.

The genes located on the SNP‐rich high‐*Pi* region are of interest. Nematodes secrete effector proteins to mediate infection, and we thus focused on short proteins containing fewer than 100 amino acids. The role of CLV3‐like (CLE) proteins during the infection process of cyst nematodes (*Heterodera* spp.) is well understood. CLE proteins were necessary for the establishment of syncytium (Hewezi, 2015), and CLE signalling was transmitted through CLV1, CLV2, and RPK2 in *Arabidopsis thaliana* (Replogle *et al*., 2011, 2013). CLE motifs were also found in proteins in RKN. In *M. incognita*, a 43 amino acid protein (16D10) was processed into a 13 amino acid peptide of sequence GKKPSGPNPGGNN, similar to the CLE motif (Huang *et al*., 2006). However, 16D10 did not complement the *A. thaliana clv3‐1* mutant (Huang *et al*., 2006). More recently, a genome sequence survey identified tandem repeats of CLE‐like motifs encoded by a single gene, *MAP* (*Meloidogyne Avirulence Protein*), but it remained unknown whether the MAP protein was processed to produce functional CLE peptides (Rutter *et al*., 2014). As well as CLE peptides, *M. incognita* produced a signal peptide similar to and functionally complementary to INFLORESCENCE DEFICIENT IN ABSCISSION (IDA) in *A. thaliana* (Kim *et al*., 2018). In this study, genes encoding several short proteins were found in the SNP‐rich high‐*Pi* region, and one of these proteins contained a CLE‐like motif (Figure 6). The other predicted short proteins did not exhibit similarities to known peptides, suggesting that they may be previously unknown effectors. Further studies are required to elucidate the roles of these peptides in the *M. incognita* infection process and their roles in determining host compatibility.

## 4 EXPERIMENTAL PROCEDURES

### 4.1 Purification of *M. incognita* isolates

Soil samples were collected from cultivated fields in Japan. Most samples were obtained from sweetpotato fields, with a few samples collected from fields used for cultivation of other crops such as taro (Sano and Iwahori, 2005) (Figure S6). Establishment of single egg mass culture of each *M. incognita* isolate was performed as described previously (Sano and Iwahori, 2005). Briefly, a susceptible tomato cv. Pritz (Kaneko Seeds Co. Ltd) seedling was planted in a 15 cm pot containing a collected soil sample. After galls developed on the roots, forceps were used to remove a single egg mass, which was then used to inoculate a second tomato Pritz seedling planted in a 15 cm pot containing commercially available Kenbyo soil (Yaenogei Co. Ltd). The infected tomato plants were grown in a greenhouse at an average temperature of 25 °C. After 6–8 weeks, roots were harvested and washed to remove soil, and ≥200 egg masses were collected per *M. incognita* isolate. Isolates were maintained by inoculation of newly planted tomato seedlings with excised egg masses.

### 4.2 Plant materials and *M. incognita* race classification

Five sweetpotato cultivars, Norin‐1, Norin‐2, Tanegashimamurasaki‐7, Elegant Summer, and J‐red, were obtained from Kyushu Okinawa Agricultural Research Center, NARO, Japan. *M. incognita* races were assigned according to infection phenotype as previously described (Sano and Iwahori, 2005). Nodes were excised from each sweetpotato cultivar and placed in tap water to induce root emergence. After 1 week, nodes with roots were planted in 9 cm pots containing 200 g sterile Kenbyo soil (Yaenogei Co. Ltd). Planted pots were incubated for 4 days before inoculation with approximately 500 J2‐stage juveniles of each *M. incognita* isolate. Inoculated plants were grown in a greenhouse at an average temperature of 25 °C for 6–8 weeks. *M. incognita* isolates that produced more than two egg masses were considered virulent, while those that produced no or one egg mass were considered avirulent. The test was performed twice using five replicates from each of five individual plants per cultivar.

### 4.3 Genomic DNA isolation from *M. incognita*

Genomic DNA of *M. incognita* isolates was extracted as follows. For each isolate, at least 100 egg masses were placed on an absorbent cotton sheet partially immersed in tap water in a 100 ml beaker for 1 week at 25 °C to induce hatching. Hatched J2 juveniles were collected by centrifugation on a sucrose density gradient (4 ml 10% sucrose and 3 ml 40% sucrose) at 2,150 × g for 15 min at 20 °C using a high‐speed refrigerated centrifuge (CR22N) equipped with a swinging bucket rotor (R4SS) (Koki Holdings Co. Ltd). The J2 juveniles on the 40% sucrose layer were collected using a glass Pasteur pipette and rinsed twice with 2 ml sterile water in an Ultrafree‐CL centrifuge filter (Merck). The J2 juveniles were then pelleted by centrifugation at 15,300 × g at 20 °C for 10 min in an antistatic 1.5 ml tube. The pelleted J2 juveniles were stored at –20 °C until used for DNA isolation. Genomic DNA was isolated using Isohair (Nippongene) according to the manufacturer's instructions, with the exception that DNA was treated with RNase (Takara Bio Inc.) prior to extraction with phenol:chloroform:isoamyl alcohol (25:24:1 vol/vol/vol). DNA was ethanol precipitated, resuspended in 20 μl TE buffer, and stored at –20 °C.

### 4.4 Sequencing and de novo assembly of a reference genome followed by gene annotation

Genomic DNA was extracted from isolate Kmmt_Gs004 as described above. DNA quality was evaluated using electrophoresis on a Femto Pulse system (Agilent), and was then used for library preparation as described previously (Kingan *et al*., 2019). In accordance with the low DNA input library preparation protocol (PacBio), a sequence library was prepared using a SMRTbell Express Template Prep Kit 2.0 (PacBio) and sequenced using a Sequel system (PacBio). The sequence reads were assembled using FALCON_Unzip v. 1.8.8 (Chin *et al*., 2016) and the resultant primary contig sequences were polished three times to correct sequence errors using ARROW v. 2.2.1 implemented in SMRT Link v. 5.0 (PacBio). Augustus (Stanke *et al*., 2006) was used for prediction of genes in the genome sequences, using the *Caenorhabditis* training preset. Gene ontologies and domains of the predicted genes were annotated using BLAST searches against non‐redundant protein sequences and InterProScan, respectively. Genome structures were compared using D‐GENIES (Cabanettes and Klopp, 2018).

### 4.5 Mapping and SNP discovery

Genomic libraries were constructed from DNA samples using a TruSeq Nano DNA Library Prep kit (Illumina) with minor modifications. Genomic DNA samples (50–100 ng) were fragmented with NEBNext dsDNA Fragmentase (New England BioLabs) and purified with Agencourt AMPure XP (Beckman Coulter) to eliminate short (<300 bp) DNA fragments. Purified DNA was ligated to index adapters (Illumina), and amplified by PCR in accordance with the manufacturer's instructions (Illumina). Genomic libraries were sequenced using Illumina HiSeq4000 and NextSeq500 instruments to obtain 101 or 76 bp paired‐end reads, respectively. Low‐quality reads were trimmed using Trimmomatic v. 0.36 (Bolger *et al*., 2014) with the following parameters: SLIDINGWINDOW:4:15 LEADING:20 TRAILING:20 MINLEN:50. High‐quality reads were mapped onto the reference genome using Bowtie2 v. 2.3.5.1 (Langmead and Salzberg, 2012). SNPs were called from the mapping results using the mpileup command in SAMtools v. 1.9 (Li, 2011).

Previously published *M. incognita* genomic reads were also used. Data from BioProjects PRJNA480412 (SRR7504310–SRR7504331, Koutsovoulos *et al*., 2020) and PRJNA340324 (SRR4242456, SRR4242460, SRR4242461, SRR4242463, SRR4242464, SRR4242465, SRR4242466, SRR4242467, SRR4242469, SRR4242470, and SRR4242479; Szitenberg *et al*., 2017) were downloaded from the NCBI SRA Database (https://www.ncbi.nlm.nih.gov/sra), trimmed, and mapped on our reference genome as described above. The relatedness of the *M. incognita* genome data generated in the two published studies and the current study was assessed by PCA implemented in TASSEL 5 (Bradbury *et al*., 2007), after elimination of the redundant data set. Phylogenetic relatedness of the genome data was analysed by neighbour‐joining method implemented in TASSEL 5 (Bradbury *et al*., 2007).

### 4.6 SNP association with infection phenotype and whole genome nucleotide diversity

Association analysis was performed between genotype and infection phenotype using the generalized linear model (GLM) implemented in TASSEL 5 (Bradbury *et al*., 2007). The infection phenotype of each isolate was assessed against five sweetpotato cultivars, Norin‐1, Norin‐2, Tanegashimamurasaki‐7, Elegant Summer, and J‐red. The interaction between each isolate and cultivar was classified as virulent or avirulent. The analysis was able to identify SNPs associated with virulence to cultivars Norin‐2, Tanegashimamurasaki‐7, and Elegant Summer. However, all isolates were virulent to cultivar Norin‐1 and avirulent to J‐red, and therefore virulence‐associated SNPs could not be identified for these two cultivars. Phenotype determination was successful for 32 of the 48 isolates examined. Statistically significant SNPs were extracted with *df* > 24 and *p* < 10^−7^ (equivalent to *p* < .05 after Bonferroni correction). The effects of SNPs on gene sequences were determined using SnpEff v. 4.3T (Cingolani *et al*., 2012).

Nucleotide diversity among *M. incognita* isolates was determined using the site‐pi command in VCFtools v. 0.1.16 (Danecek *et al*., 2011) and plotted in R v. 3.6.1 (R Core Team, 2019).

## Supporting information


**FIGURE S1** Evaluation of the mapping results. (a) The fold coverage shown in Table S1 did not affect mapping rate. (b) Comparison of mapping rate with previously reported *Meloidogyne incognita* reference genome

**FIGURE S2** Moving average of *Pi* for each contig with window size of 50 bp. The mean value of *Pi* was 0.15 over the whole genome. Extended high‐*Pi* (>0.3) regions of ≤500 kb were found on contigs 001 and 012, and longer regions of approximately 1 Mb were found on contigs 005 and 021

**FIGURE S3** Comparison of nucleotide diversity (*Pi*) between sweetpotato races on contigs MINJ2_001F.1, MINJ2_012F.1, and MINJ2_021F.1. In each panel, black line shows the *Pi* plot of races 1 and 2, and red line shows the *Pi* plot of races 3, 4 and 6. These contigs did not show different *Pi* values in association with the races

**FIGURE S4** Phylogenetic tree of *Meloidogyne incognita* isolates of global origins. For isolates from Japan, no relationships between geographic origin and sweetpotato race were observed

**FIGURE S5** A coverage plot for the contig MINJ2_005F.1 together with the *Pi*. Values in sliding windows after dividing the entire contig into 50 blocks were indicated by bold line for the *Pi,* and by broken line for the read coverage. The result showed no clear difference of the mapping depth between the first 1 Mb region with the concentrated single nucleotide polymorphisms (SNPs) and the following regions, suggesting that the concentrated SNPs region in this contig did not result from a collapsed artefact of the assembly

**FIGURE S6** Collection sites of *Meloidogyne incognita* isolates used in this study. Forty‐eight isolates were collected from sweetpotato and other crop fields in Japan. Isolates were collected over a wide geographical range, from Mori, Hokkaido (42°06′17″N) in the north to Ishigaki, Okinawa (24°20′26″N) in the south. Red dots indicate cities where fields are located. Orange dots indicate sample locations where the precise field locations are unknown. One isolate (Unknown_unknown) is not shown in this Figure, because its record of collected site was lost

**TABLE S1** Summary of Illumina short read sequences accumulated for each isolate

**TABLE S2** List of significant single nucleotide polymorphisms (SNPs) affecting coding sequences, related to the infection trait

**FILE S1** Coding sequences of the predicted genes in MINJ2

**FILE S2** Genome positions of the predicted genes in MINJ2

**FILE S3** Protein translations of the predicted genes in MINJ2

## Data Availability

The MINJ2 assembly data that support this study are openly available in DNA Data Bank of Japan (DDBJ) at https://www.ddbj.nig.ac.jp, reference numbers BLLR01000001–BLLR01000374. The PacBio and Illumina raw reads are available in DDBJ Sequence Read Archive (DRA) at https://www.ddbj.nig.ac.jp/dra, accession number DRA009730.
