# Supplementary figures and images for: Root‐knot nematode genetic diversity associated with host compatibility to sweetpotato cultivars
Source: Mol Plant Pathol. 2020 Jun 17;21(8):1088–98. doi: 10.1111/mpp.12961 (PMC7368124; doi:10.1111/mpp.12961)

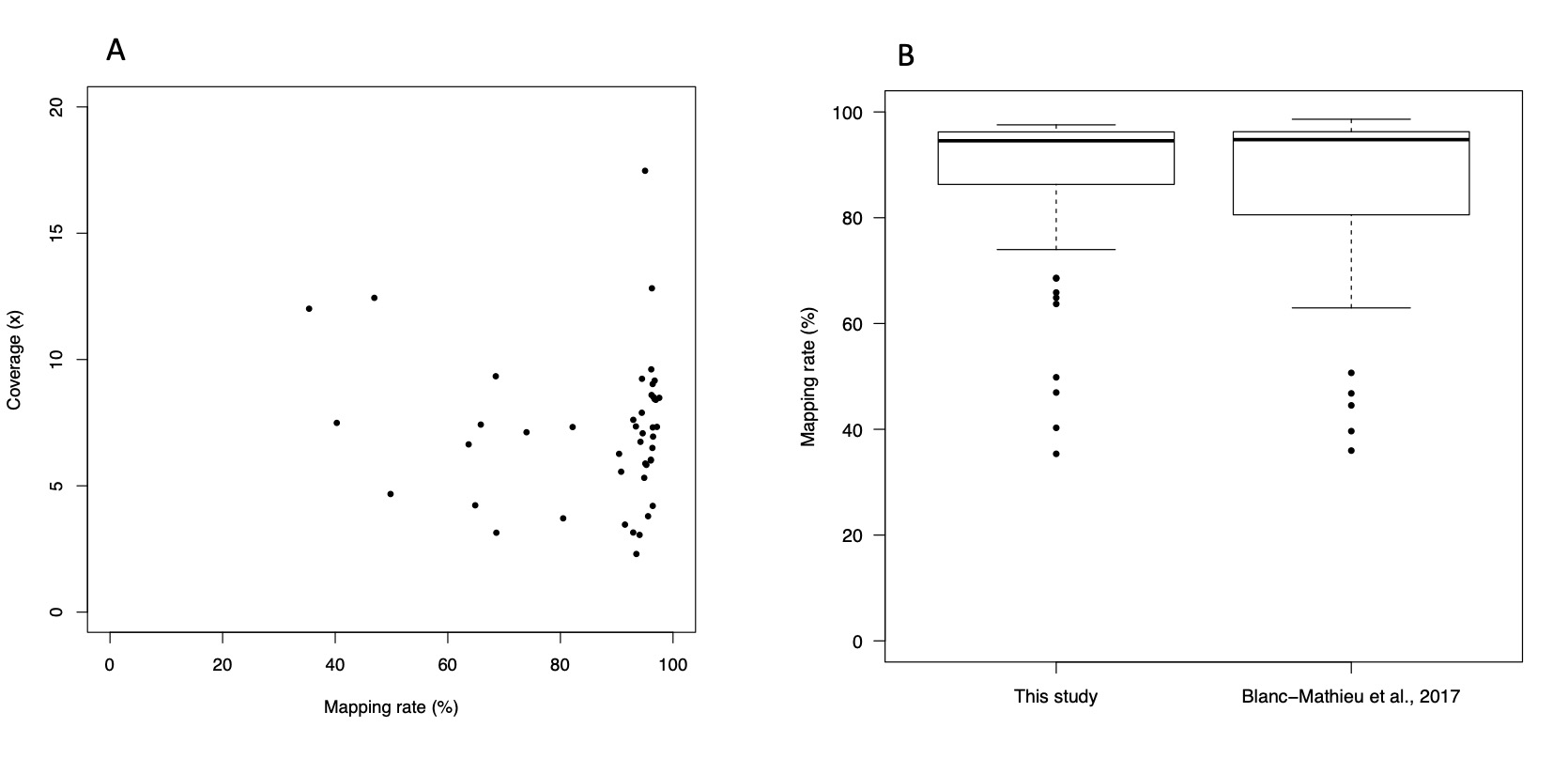

Supplement: Supplementary file 1 — FIGURE S1 Evaluation of the mapping results. (a) The fold coverage shown in Table S1 did not affect mapping rate. (b) Comparison of mapping rate with previously reported Meloidogyne incognita reference genome [file MPP-21-1088-s001.jpg]

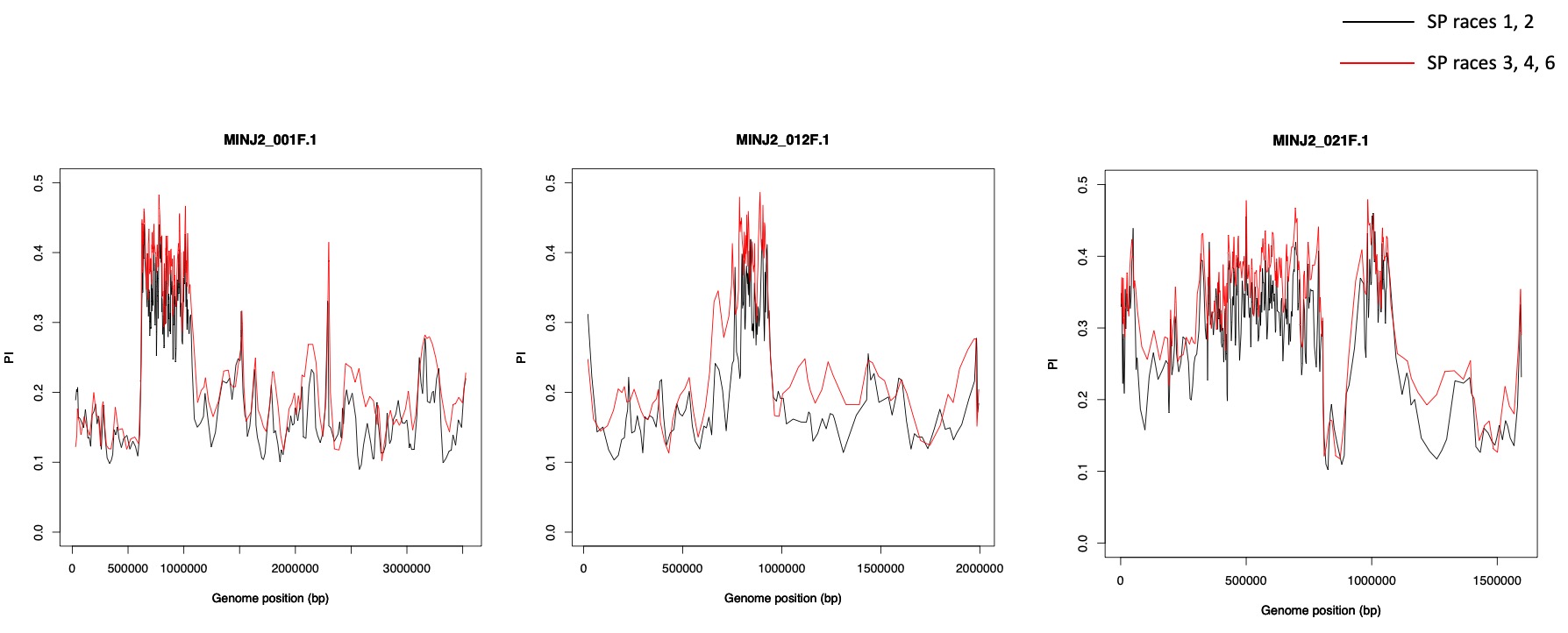

Supplement: Supplementary file 3 — FIGURE S3 Comparison of nucleotide diversity (Pi) between sweetpotato races on contigs MINJ2_001F.1, MINJ2_012F.1, and MINJ2_021F.1. In each panel, black line shows the Pi plot of races 1 and 2, and red line shows the Pi plot of races 3, 4 and 6. These contigs did not show different Pi values in association with the races [file MPP-21-1088-s003.jpg]

Fig. S4

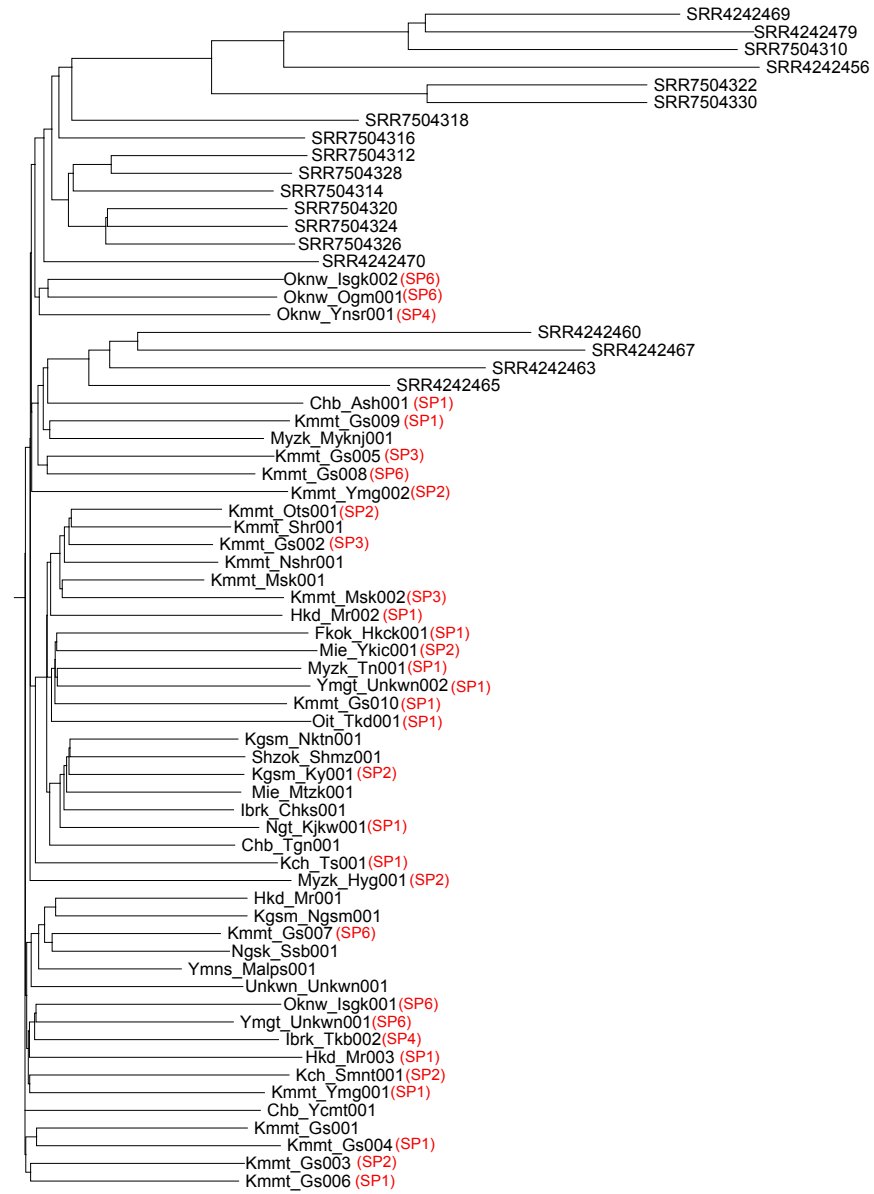

Supplement: Supplementary file 4 — FIGURE S4 Phylogenetic tree of Meloidogyne incognita isolates of global origins. For isolates from Japan, no relationships between geographic origin and sweetpotato race were observed [file MPP-21-1088-s004.pdf]

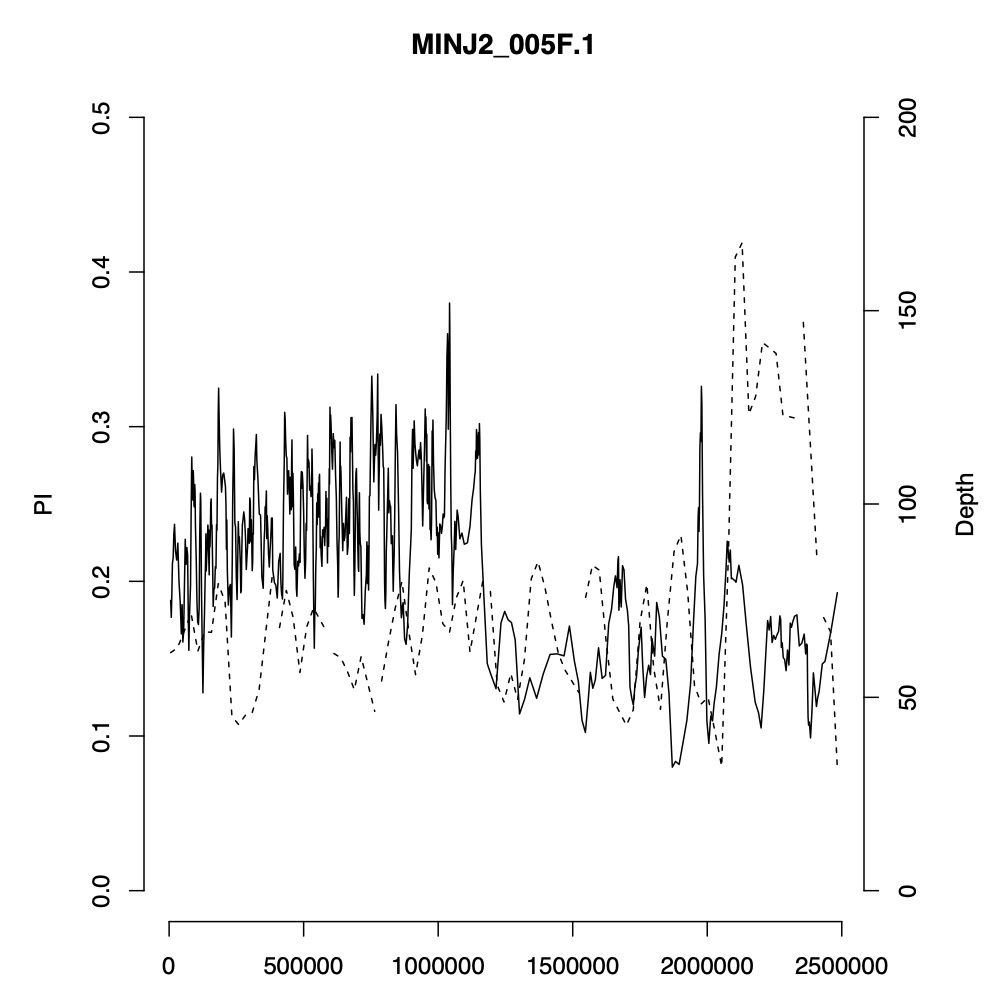

Supplement: Supplementary file 5 — FIGURE S5 A coverage plot for the contig MINJ2_005F.1 together with the Pi. Values in sliding windows after dividing the entire contig into 50 blocks were indicated by bold line for the Pi, and by broken line for the read coverage. The result showed no clear difference of the mapping depth between the first 1 Mb region with the concentrated single nucleotide polymorphisms (SNPs) and the following regions, suggesting that the concentrated SNPs region in this contig did not result from a collapsed artefact of the assembly [file MPP-21-1088-s005.jpg]

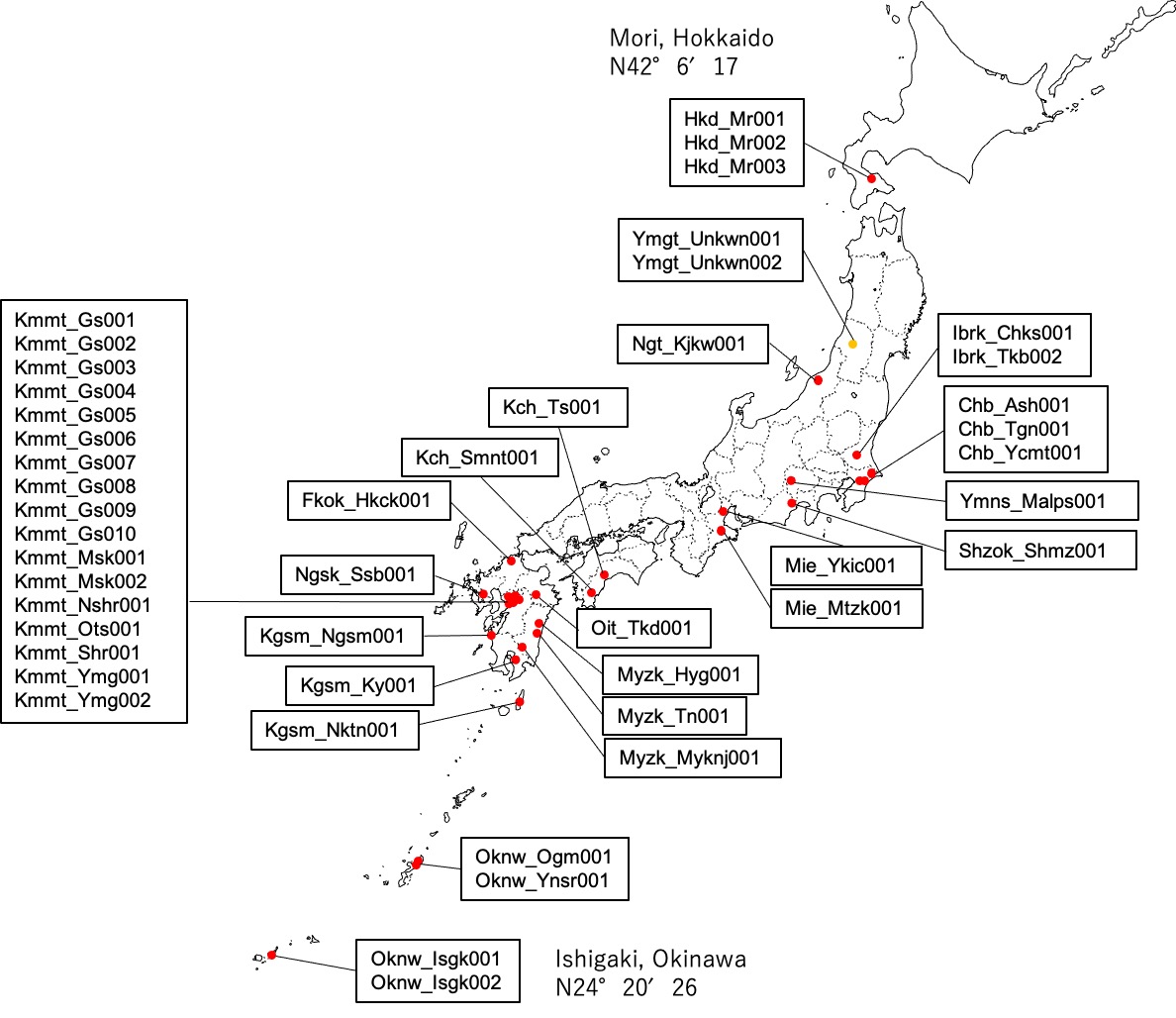

Supplement: Supplementary file 6 — FIGURE S6 Collection sites of Meloidogyne incognita isolates used in this study. Forty‐eight isolates were collected from sweetpotato and other crop fields in Japan. Isolates were collected over a wide geographical range, from Mori, Hokkaido (42°06′17″N) in the north to Ishigaki, Okinawa (24°20′26″N) in the south. Red dots indicate cities where fields are located. Orange dots indicate sample locations where the precise field locations are unknown. One isolate (Unknown_unknown) is not shown in this Figure, because its record of collected site was lost [file MPP-21-1088-s006.jpg]
