# Supplementary material for: Root‐knot nematode genetic diversity associated with host compatibility to sweetpotato cultivars
Source: Mol Plant Pathol. 2020 Jun 17;21(8):1088–98. doi: 10.1111/mpp.12961 (PMC7368124; doi:10.1111/mpp.12961)
Supplement: Supplementary file 2 — FIGURE S2 Moving average of Pi for each contig with window size of 50 bp. The mean value of Pi was 0.15 over the whole genome. Extended high‐Pi (>0.3) regions of ≤500 kb were found on contigs 001 and 012, and longer regions of approximately 1 Mb were found on contigs 005 and 021 [file MPP-21-1088-s002.pdf]

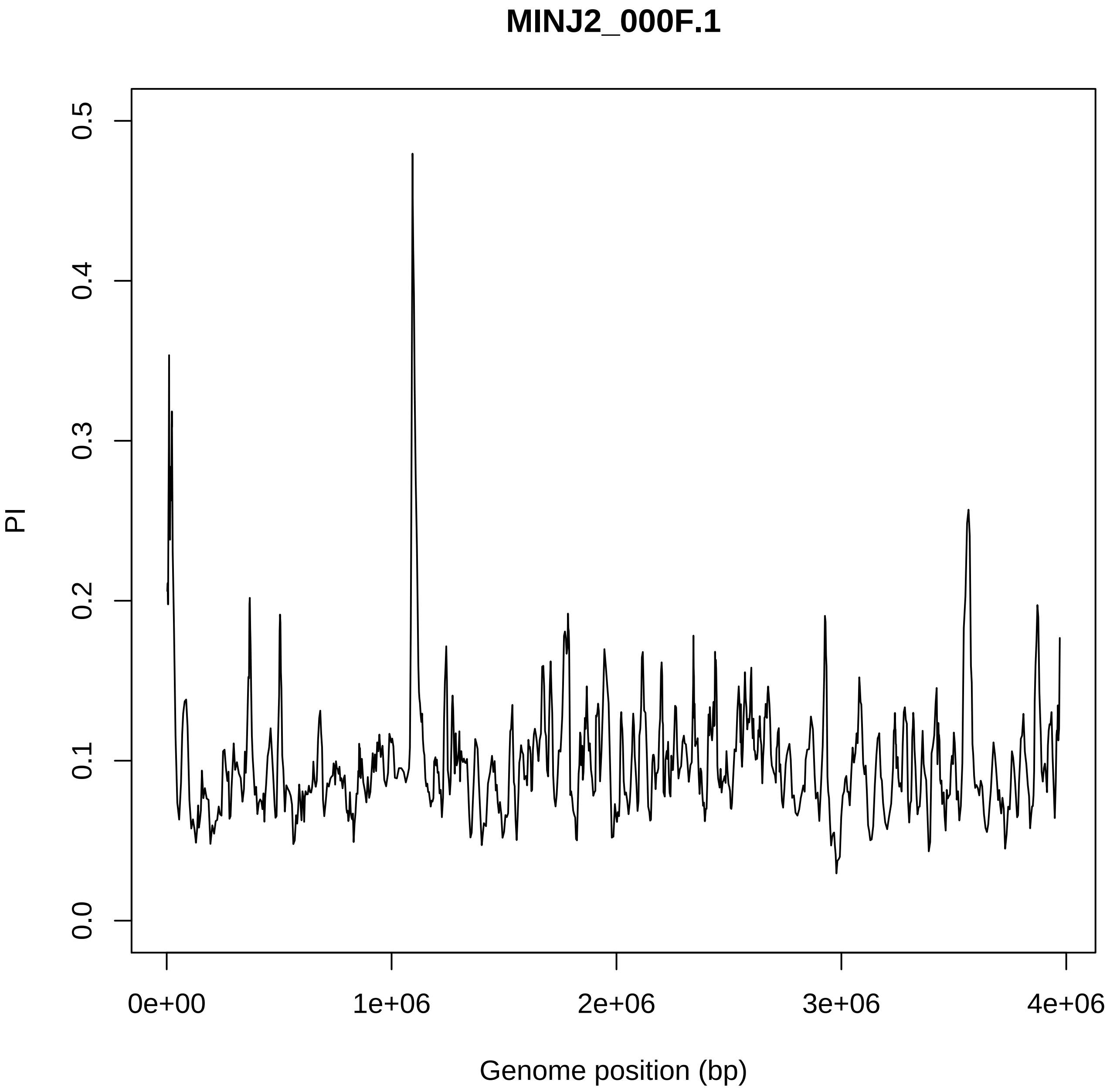
MINJ2_000F.1
PI
0.0
0.1
0.2
0.3
0.4
0.5
0e+00
1e+06
2e+06
3e+06
4e+06
Genome position (bp)

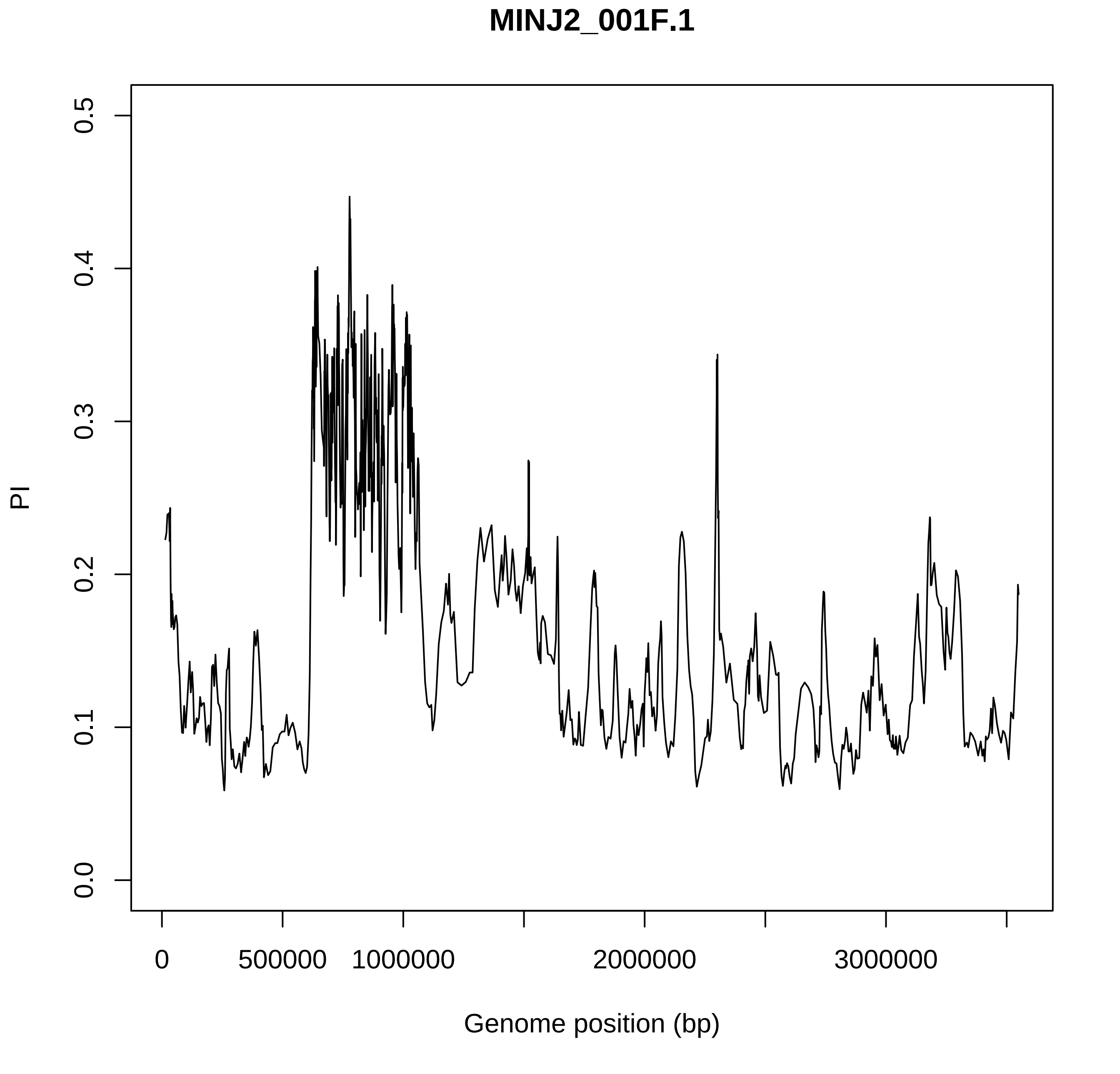

MINJ2_001F.1
PI
0.0
0.1
0.2
0.3
0.4
0.5
0
500000
1000000
2000000
3000000
Genome position (bp)

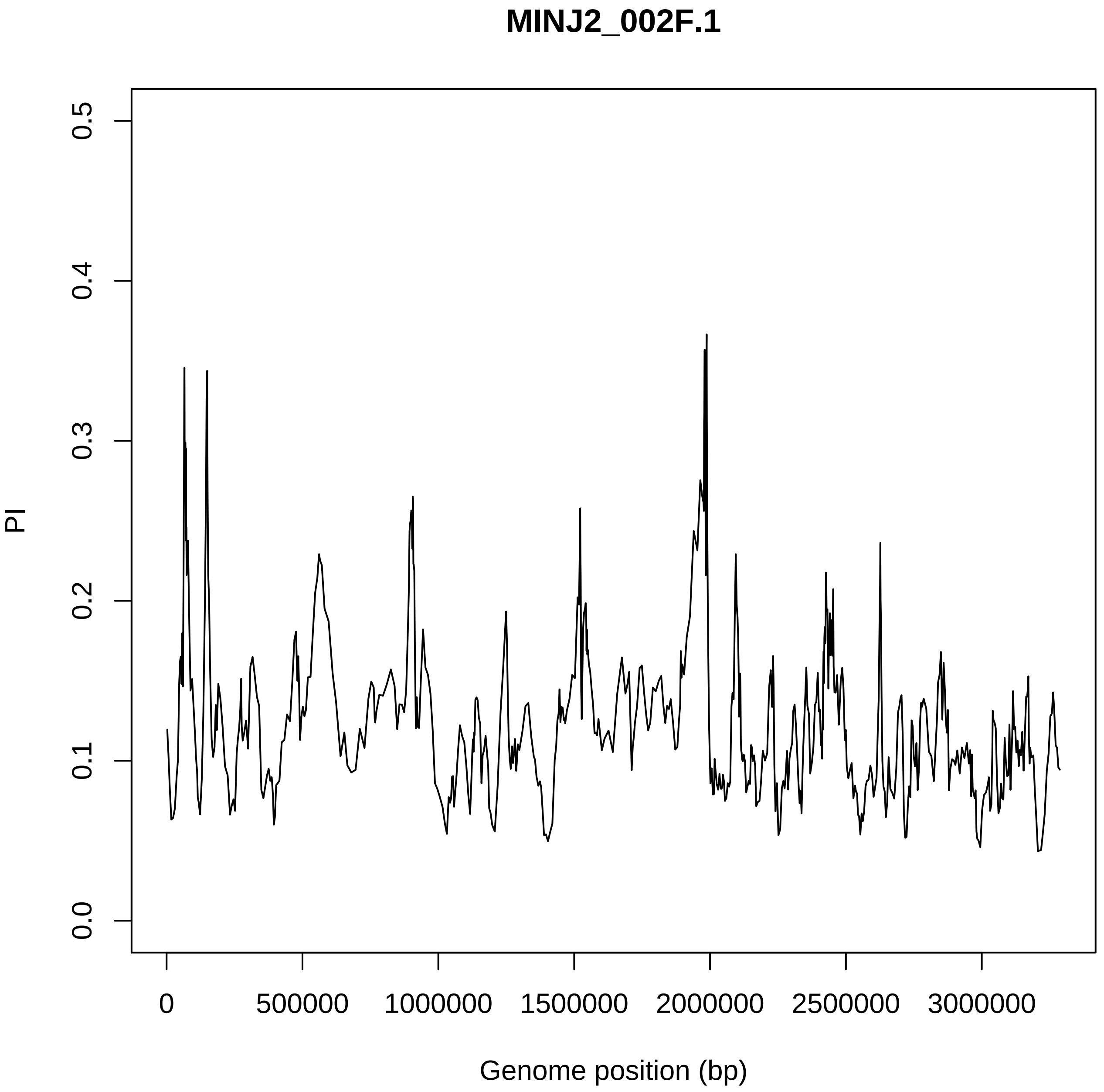
MINJ2_002F.1
PI
0.0
0.1
0.2
0.3
0.4
0.5
0
500000
1000000
1500000
2000000
2500000
3000000
Genome position (bp)

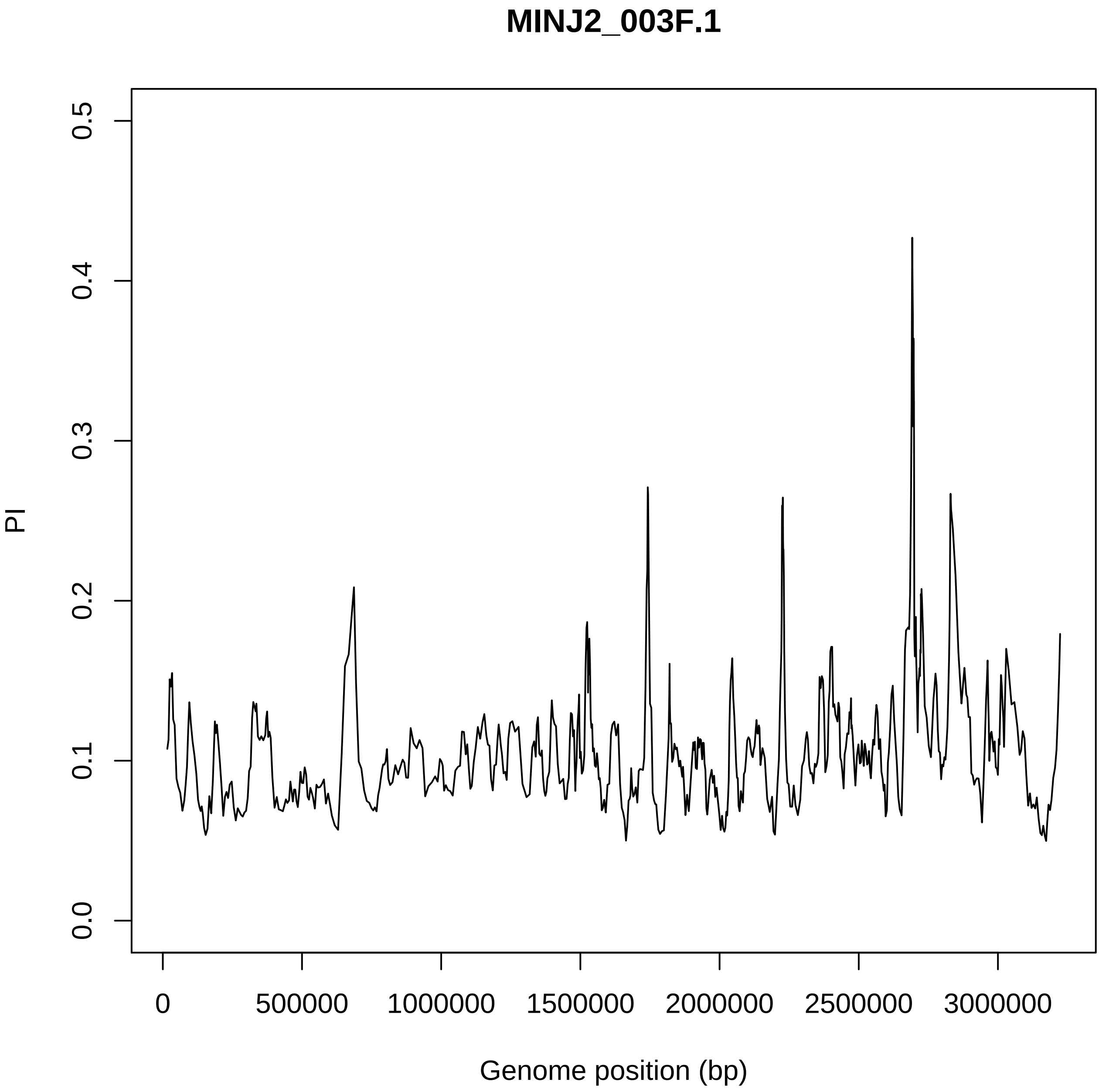
MINJ2_003F.1
PI
0.0
0.1
0.2
0.3
0.4
0.5
0
500000
1000000
1500000
2000000
2500000
3000000
Genome position (bp)

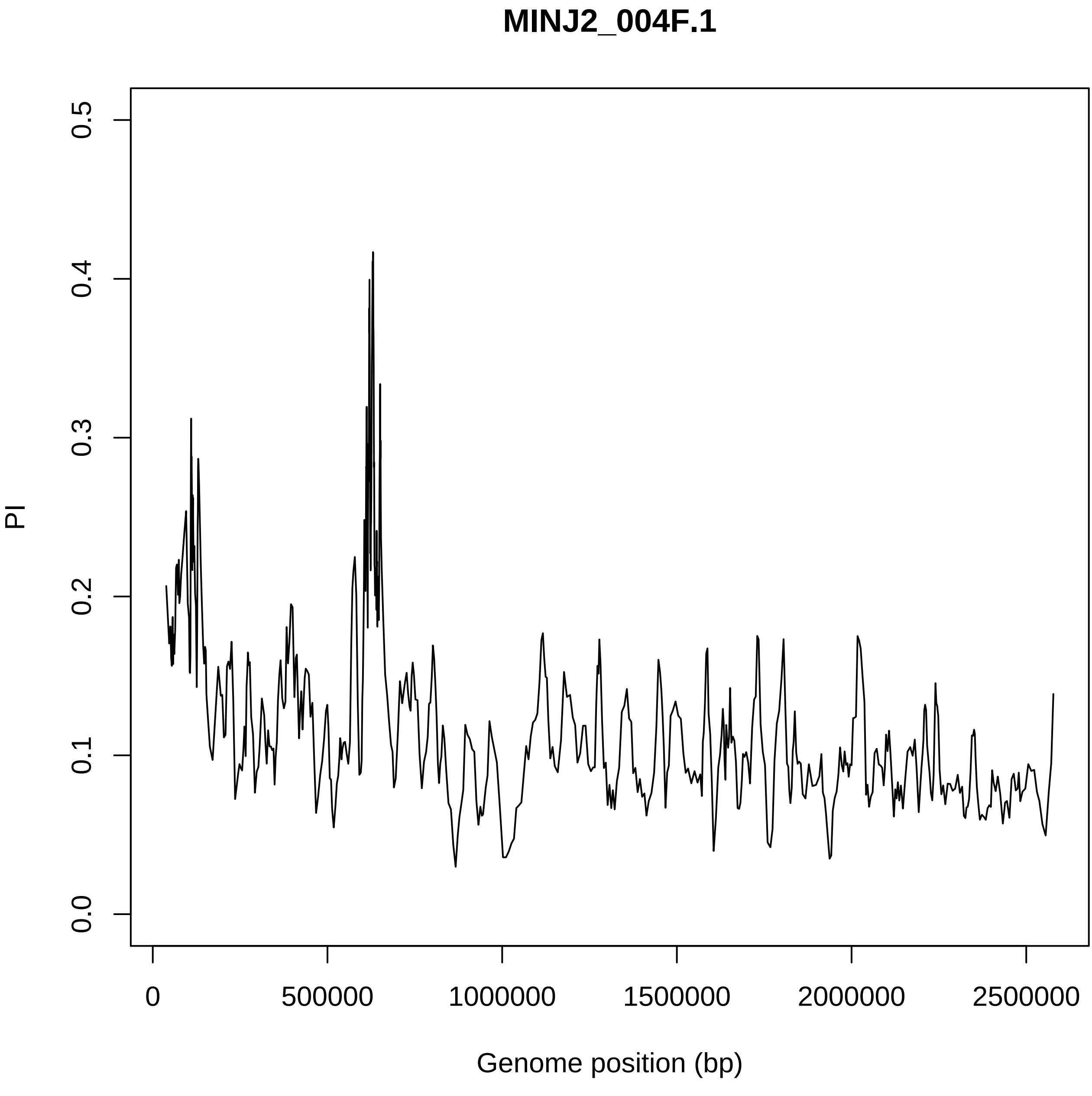
MINJ2_004F.1
PI
0.0
0.1
0.2
0.3
0.4
0.5
0
500000
1000000
1500000
2000000
2500000
Genome position (bp)

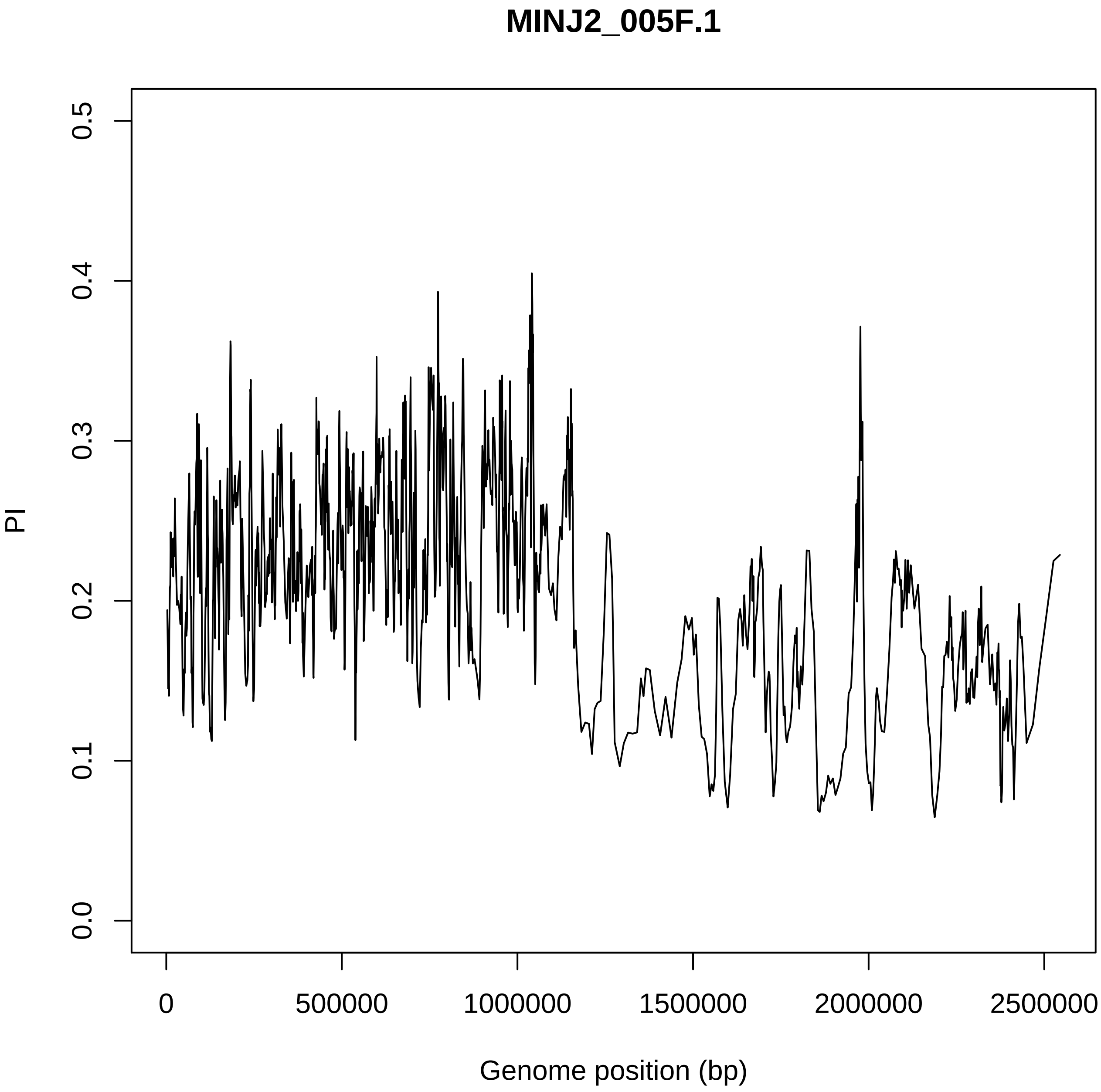

MINJ2_005F.1
PI
0.0
0.1
0.2
0.3
0.4
0.5
0
500000
1000000
1500000
2000000
2500000
Genome position (bp)

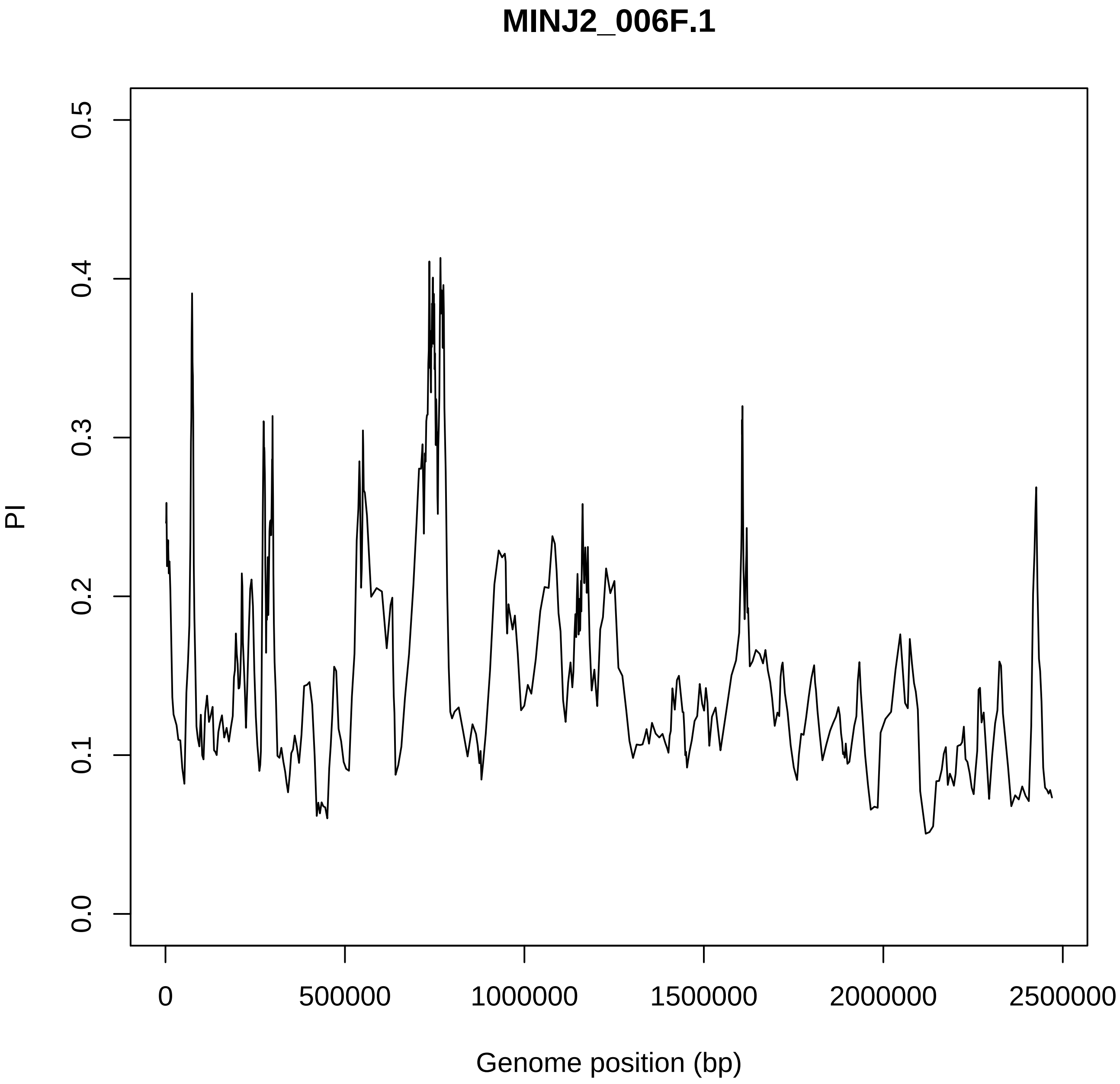
MINJ2_006F.1
PI
0.0
0.1
0.2
0.3
0.4
0.5
0
500000
1000000
1500000
2000000
2500000
Genome position (bp)

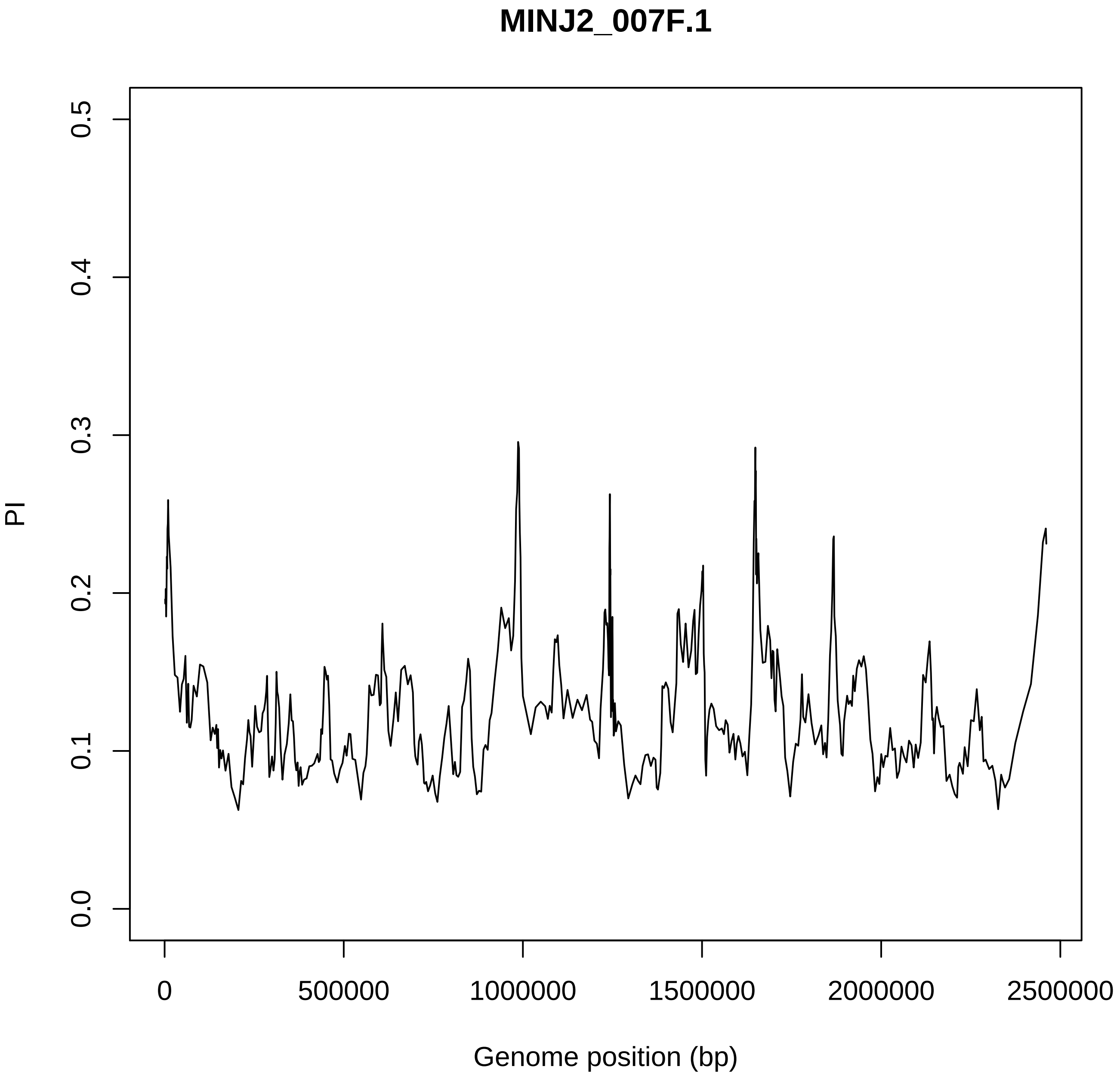
MINJ2_007F.1
PI
0.0
0.1
0.2
0.3
0.4
0.5
0
500000
1000000
1500000
2000000
2500000
Genome position (bp)

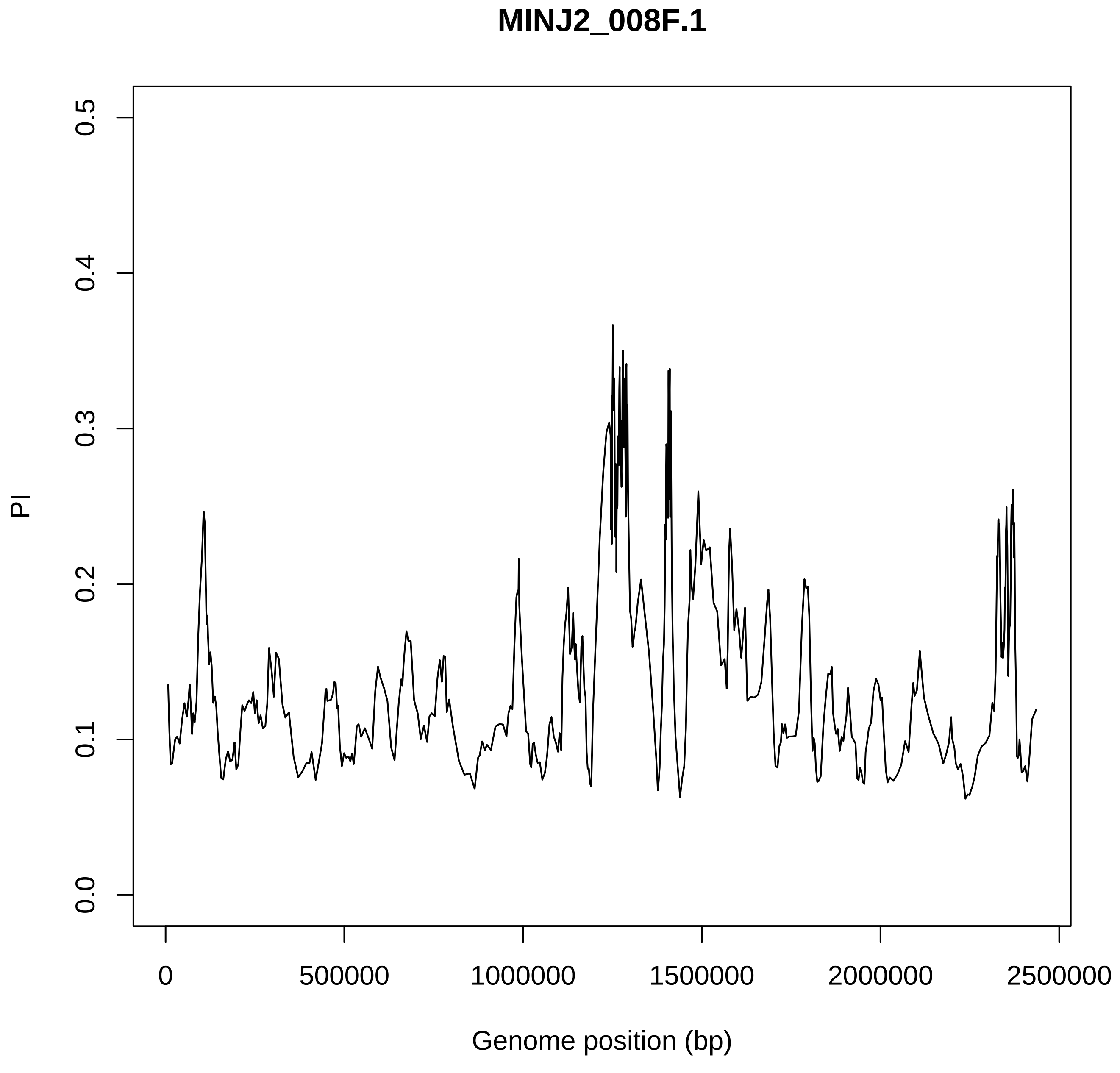

MINJ2_008F.1
PI
0.0
0.1
0.2
0.3
0.4
0.5
0
500000
1000000
1500000
2000000
2500000
Genome position (bp)

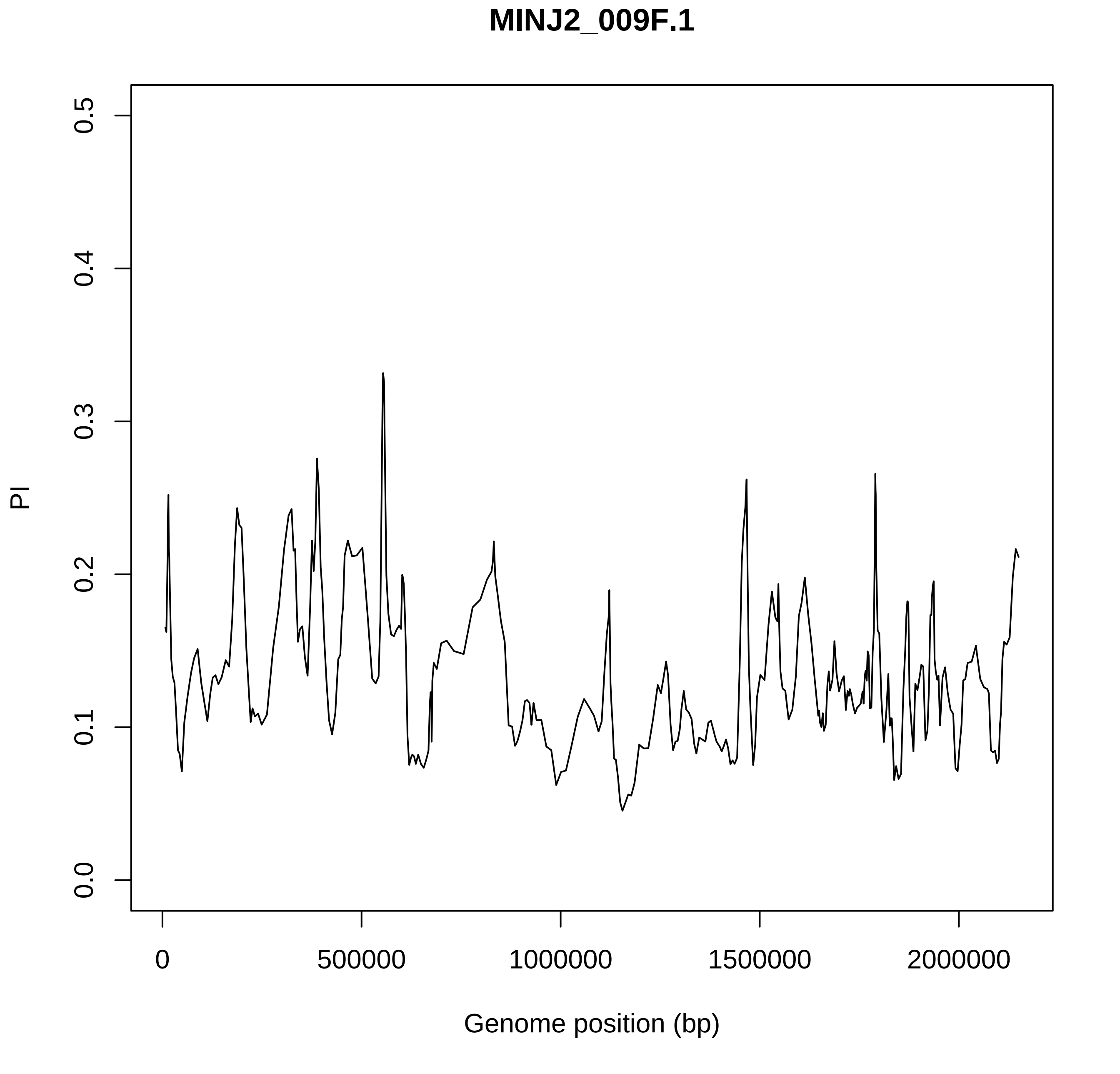
MINJ2_009F.1
PI
0.0
0.1
0.2
0.3
0.4
0.5
0
500000
1000000
1500000
2000000
Genome position (bp)

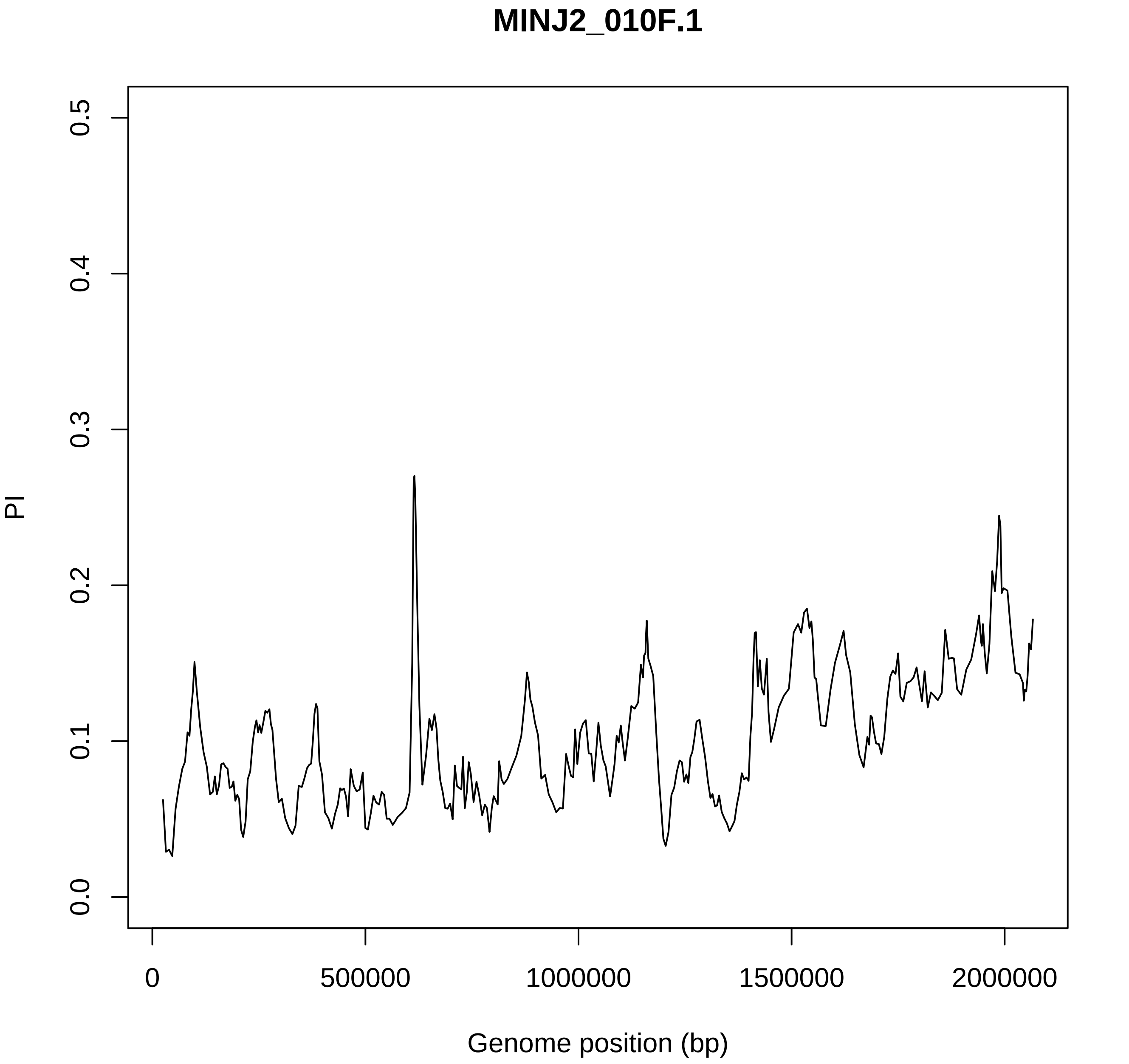
MINJ2_010F.1
PI
0.0
0.1
0.2
0.3
0.4
0.5
0
500000
1000000
1500000
2000000
Genome position (bp)

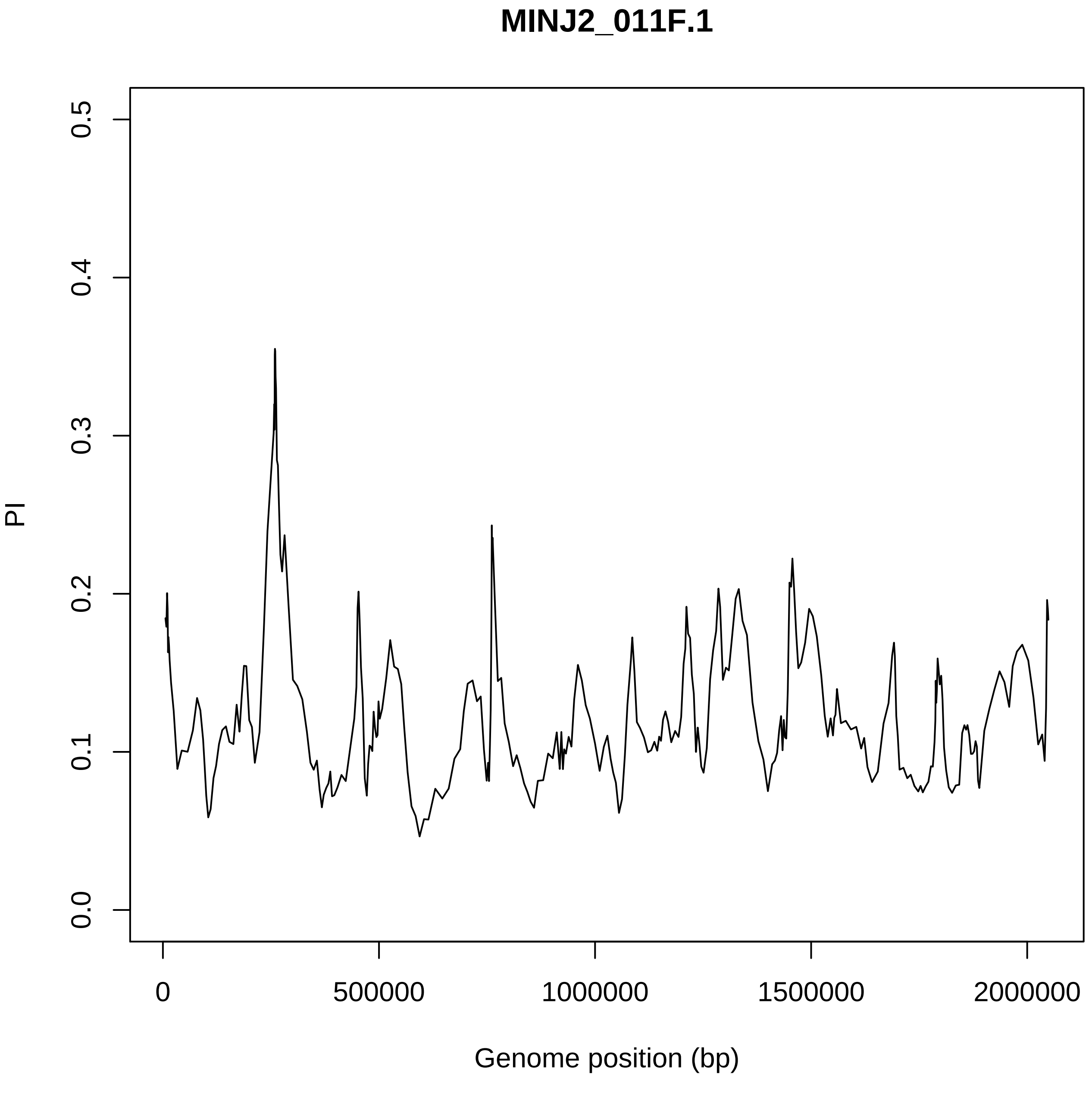
MINJ2_011F.1
PI
0.0
0.1
0.2
0.3
0.4
0.5
0
500000
1000000
1500000
2000000
Genome position (bp)

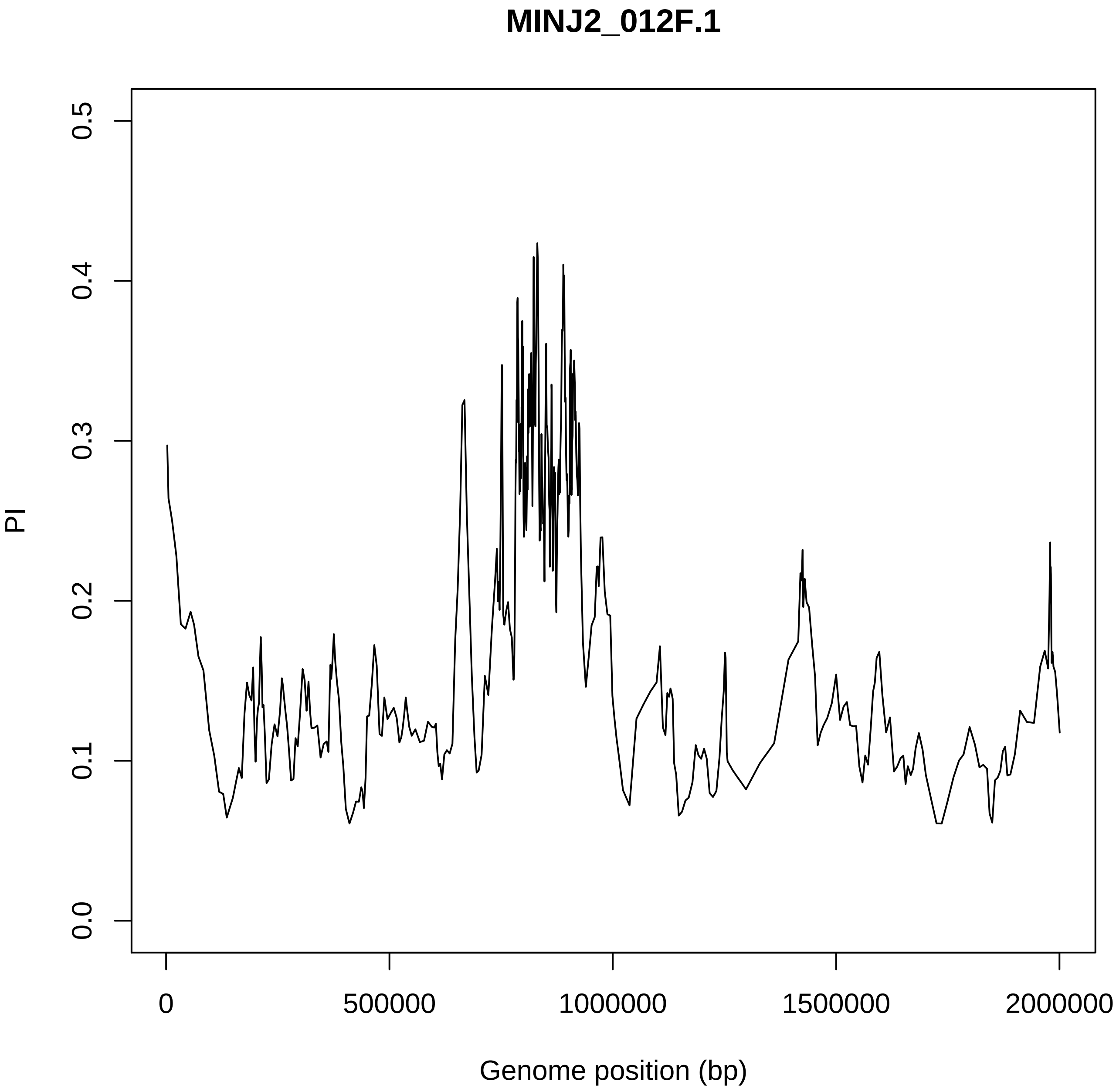
MINJ2_012F.1
PI
Genome position (bp)
0.0
0.1
0.2
0.3
0.4
0.5
0
500000
1000000
1500000
2000000

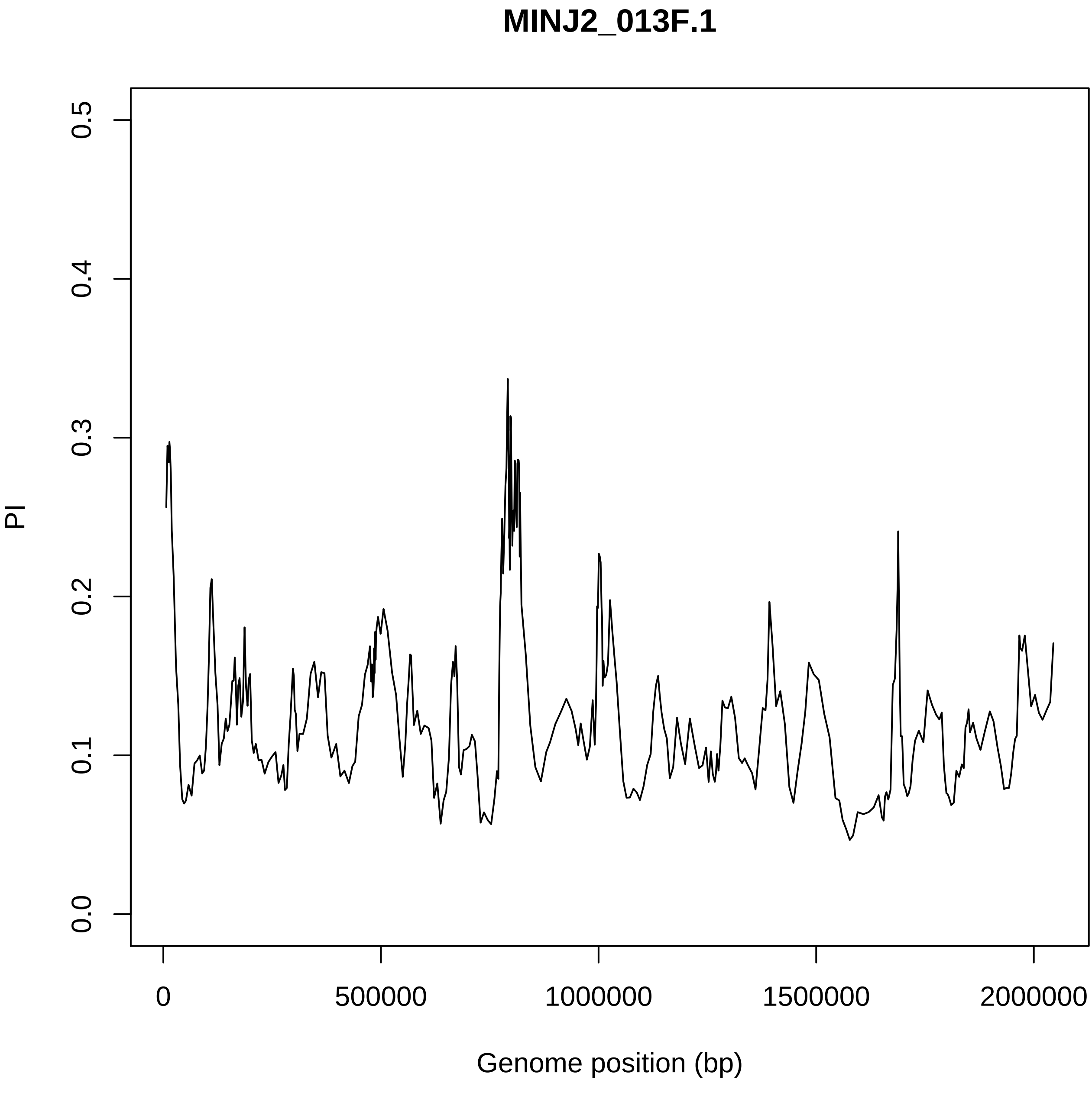

MINJ2_013F.1
PI
0.0
0.1
0.2
0.3
0.4
0.5
0
500000
1000000
1500000
2000000
Genome position (bp)

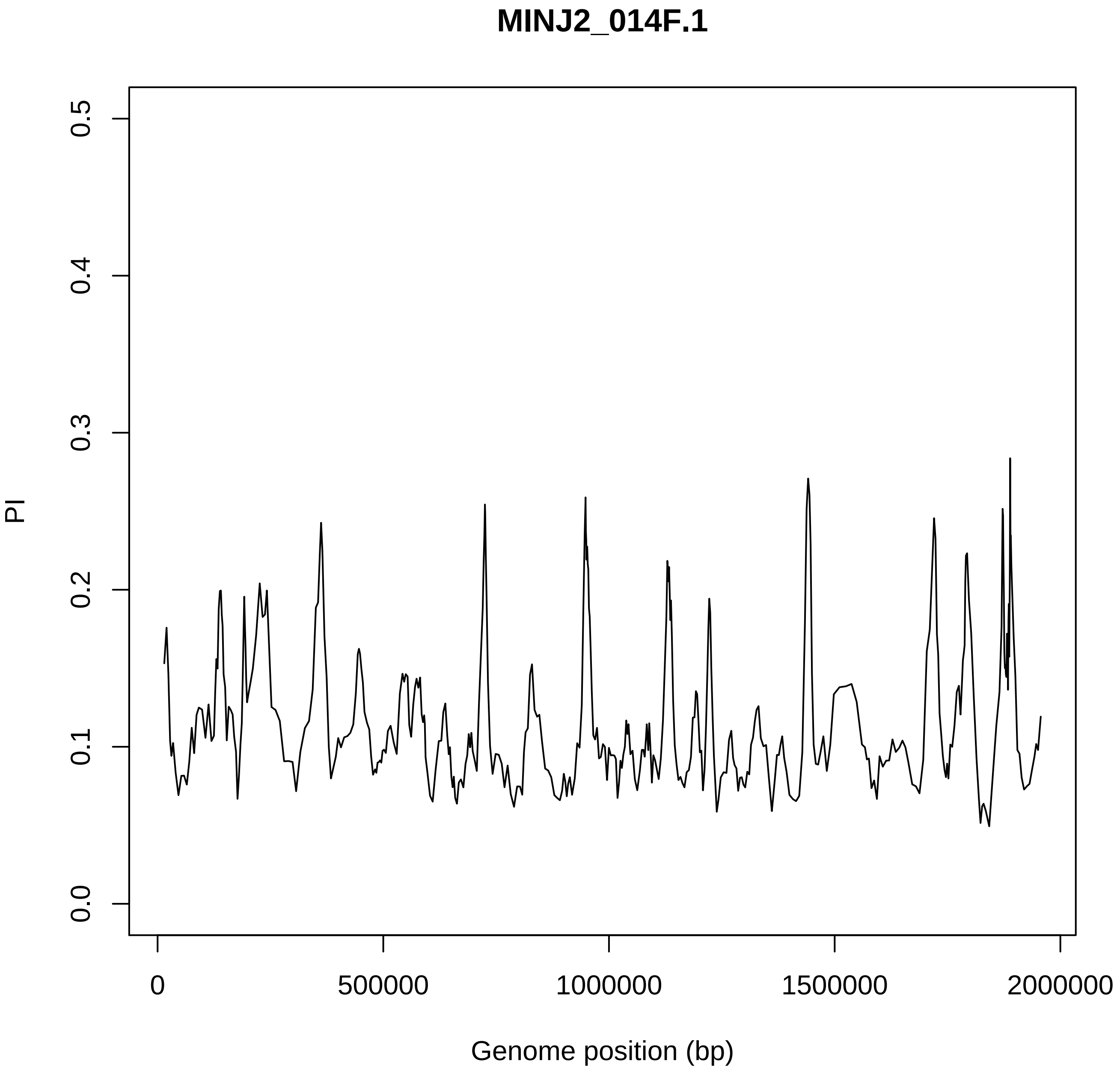

MINJ2_014F.1
PI
0.0
0.1
0.2
0.3
0.4
0.5
0
500000
1000000
1500000
2000000
Genome position (bp)

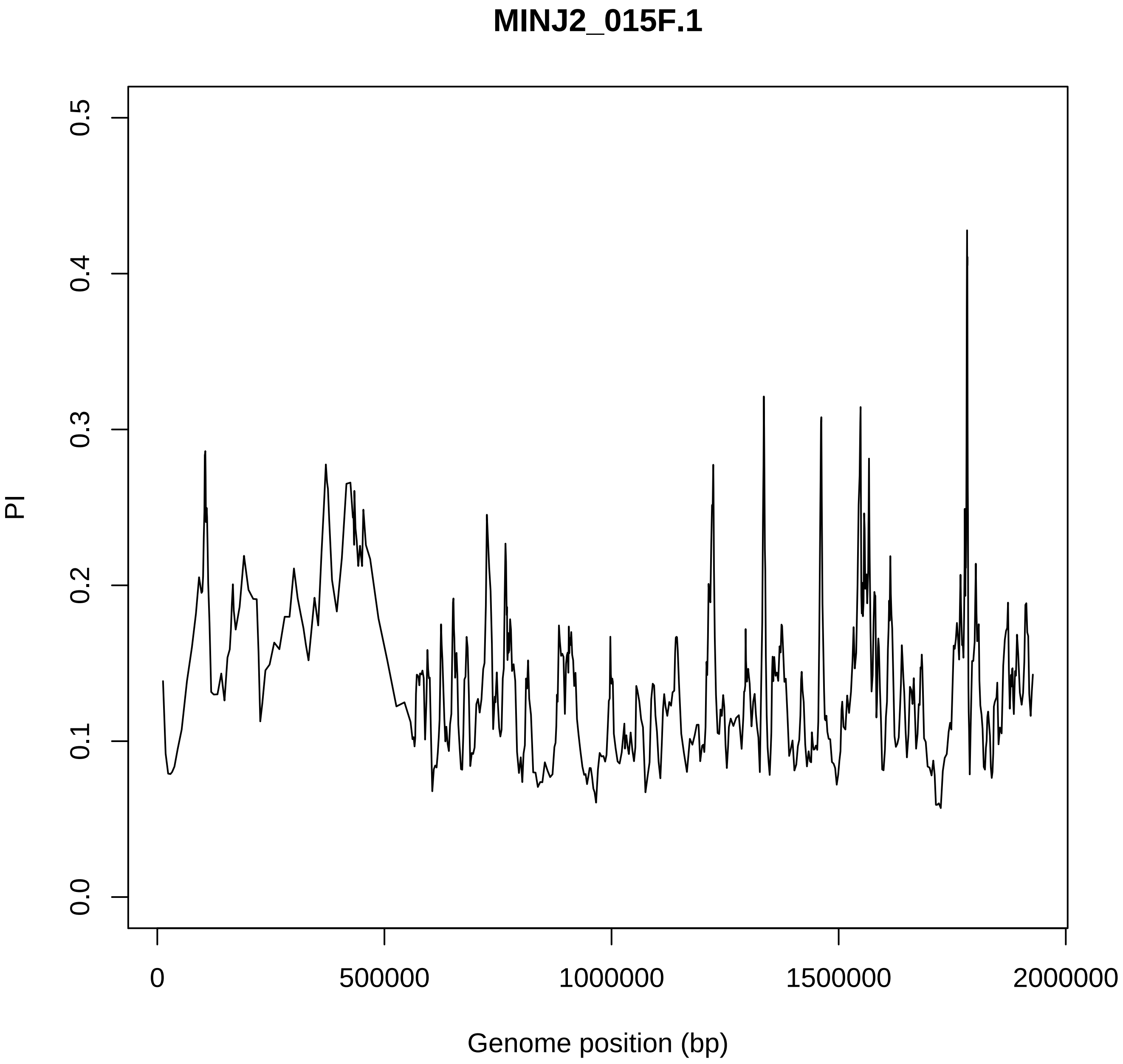
MINJ2_015F.1
PI
0.0
0.1
0.2
0.3
0.4
0.5
0
500000
1000000
1500000
2000000
Genome position (bp)

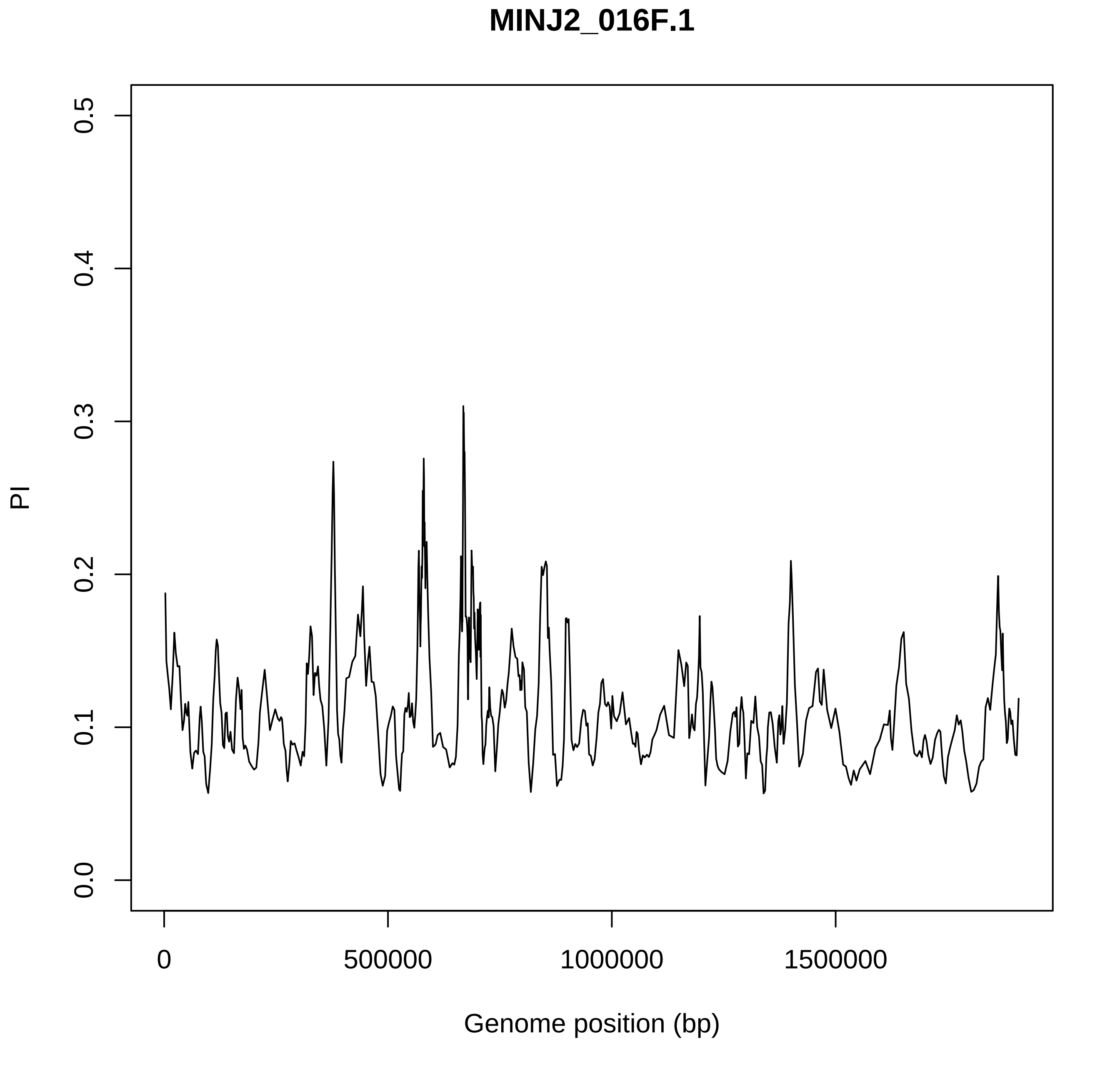
MINJ2_016F.1
PI
0.0
0.1
0.2
0.3
0.4
0.5
0
500000
1000000
1500000
Genome position (bp)

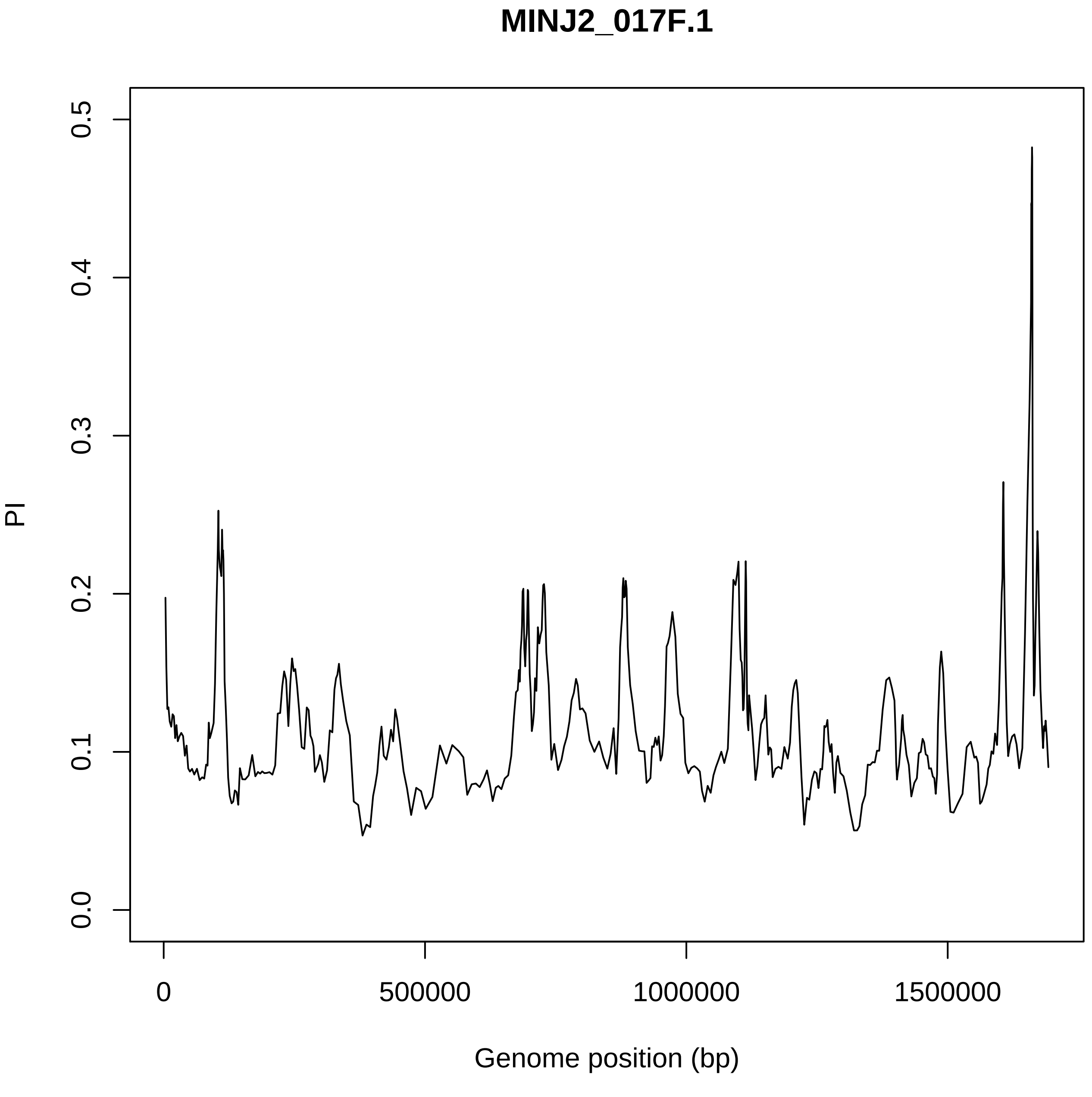
MINJ2_017F.1
PI
Genome position (bp)
0.0
0.1
0.2
0.3
0.4
0.5
0
500000
1000000
1500000

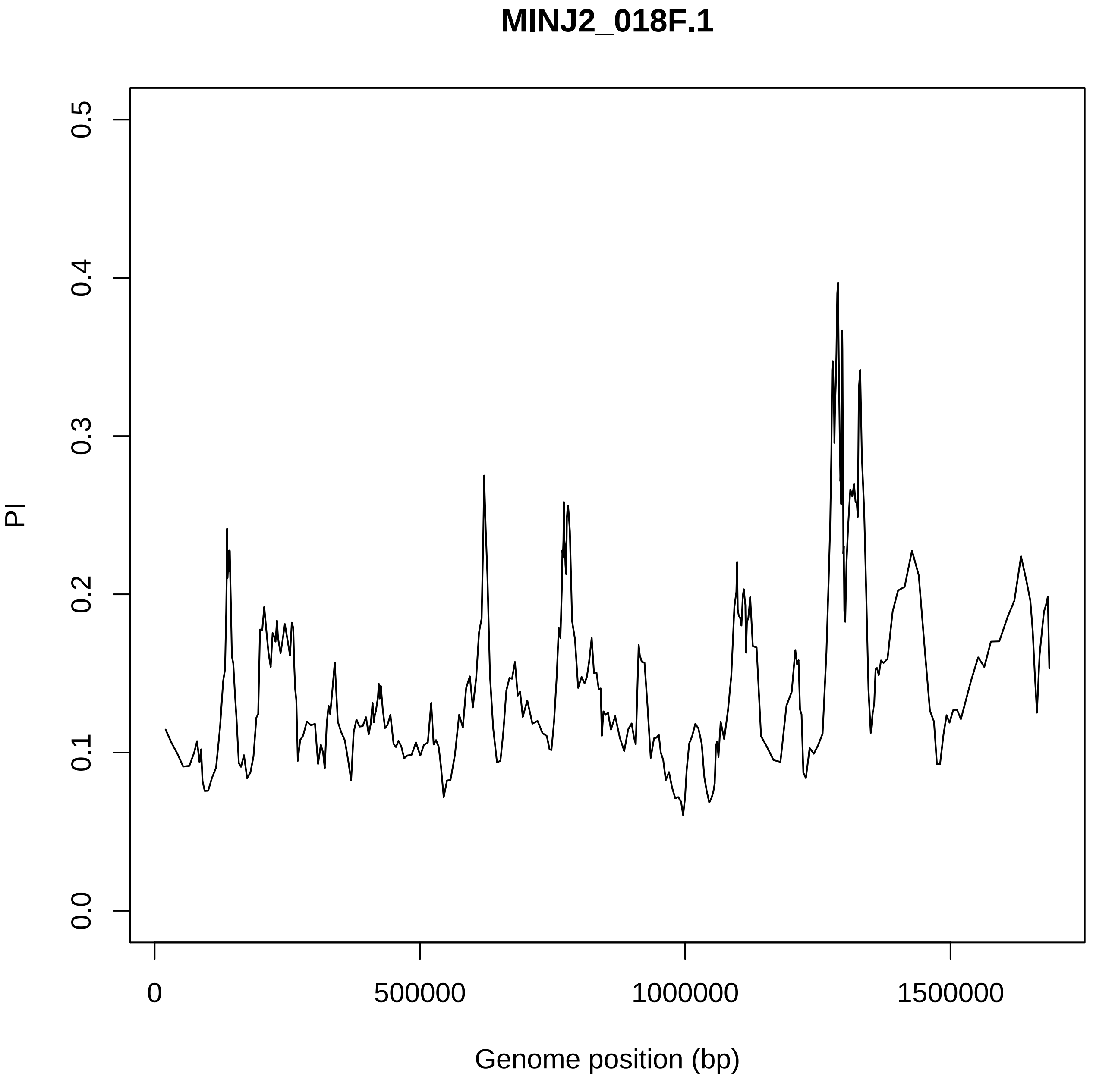

MINJ2_018F.1
PI
0.0
0.1
0.2
0.3
0.4
0.5
0
500000
1000000
1500000
Genome position (bp)

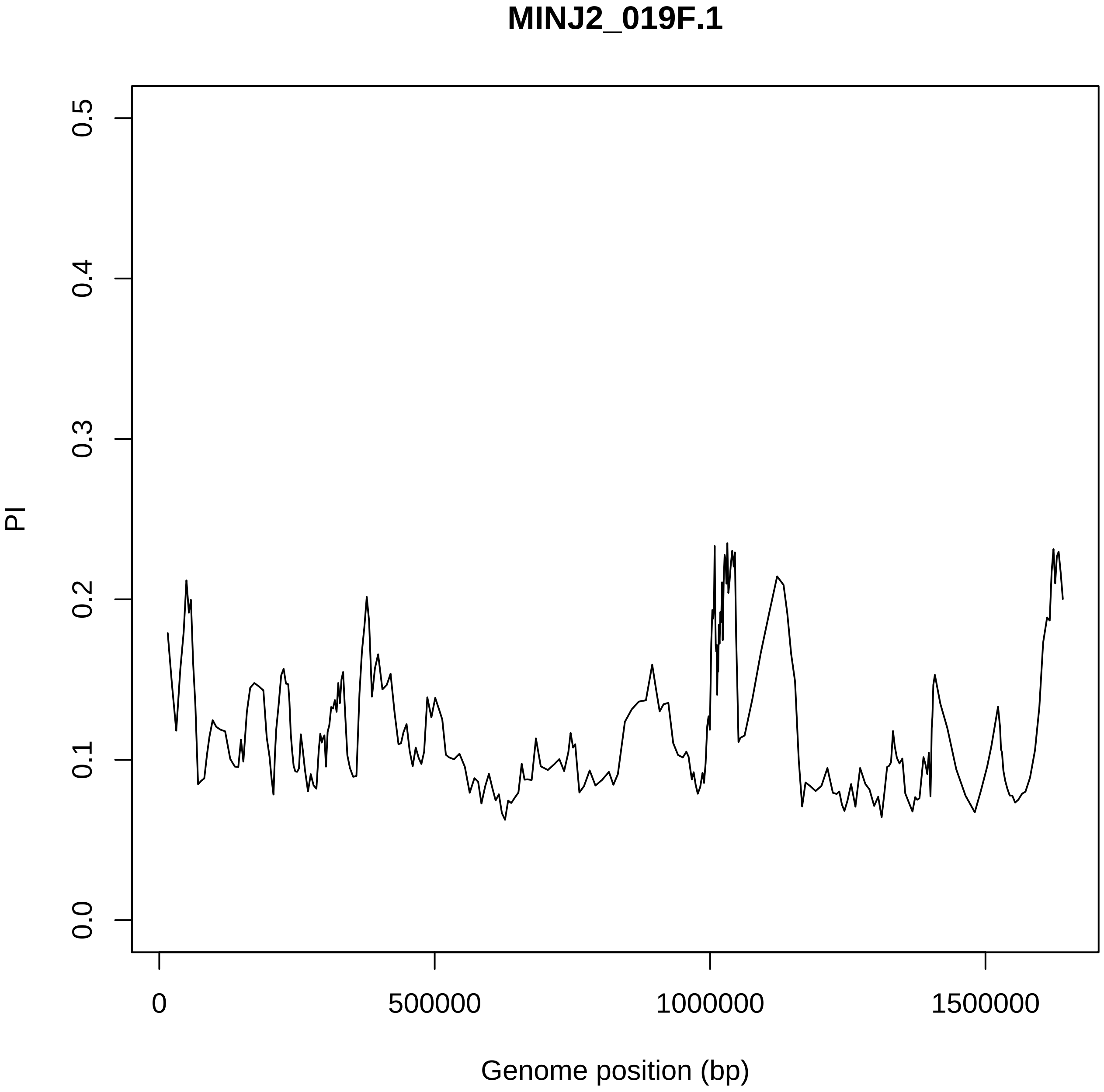
MINJ2_019F.1
PI
0.0
0.1
0.2
0.3
0.4
0.5
0
500000
1000000
1500000
Genome position (bp)

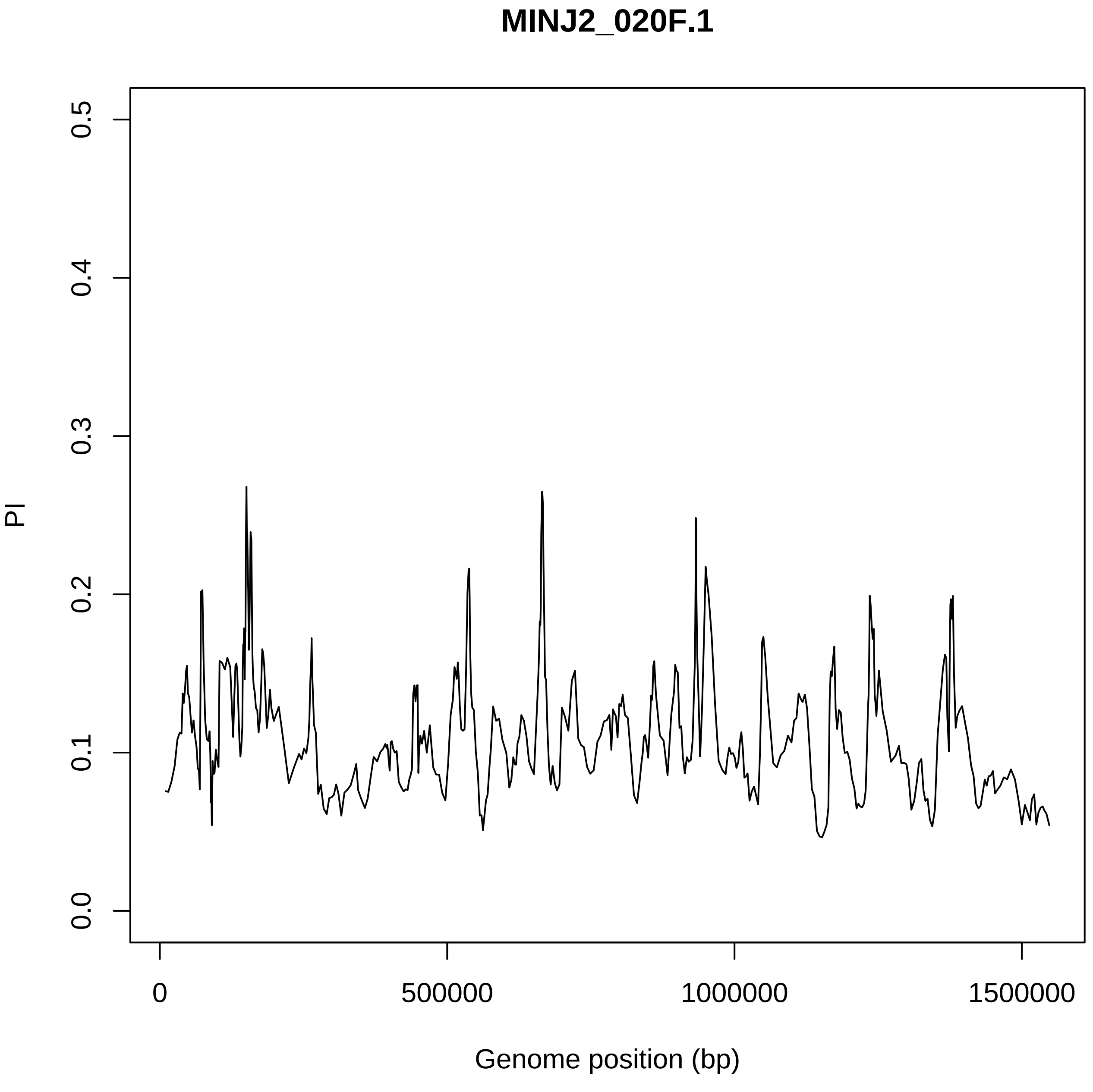
MINJ2_020F.1
PI
0.0
0.1
0.2
0.3
0.4
0.5
0
500000
1000000
1500000
Genome position (bp)

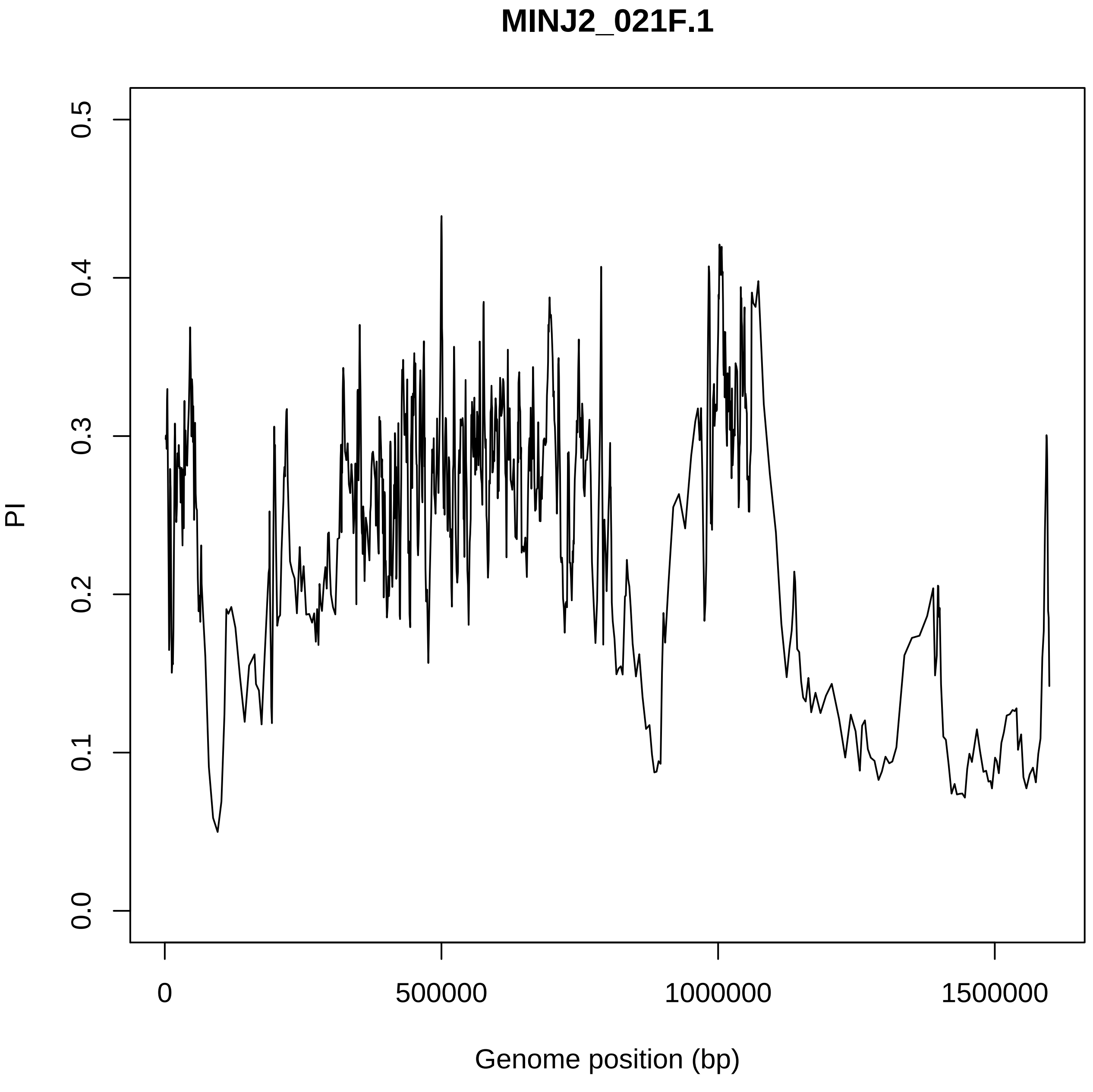
MINJ2_021F.1
PI
0.0
0.1
0.2
0.3
0.4
0.5
0
500000
1000000
1500000
Genome position (bp)

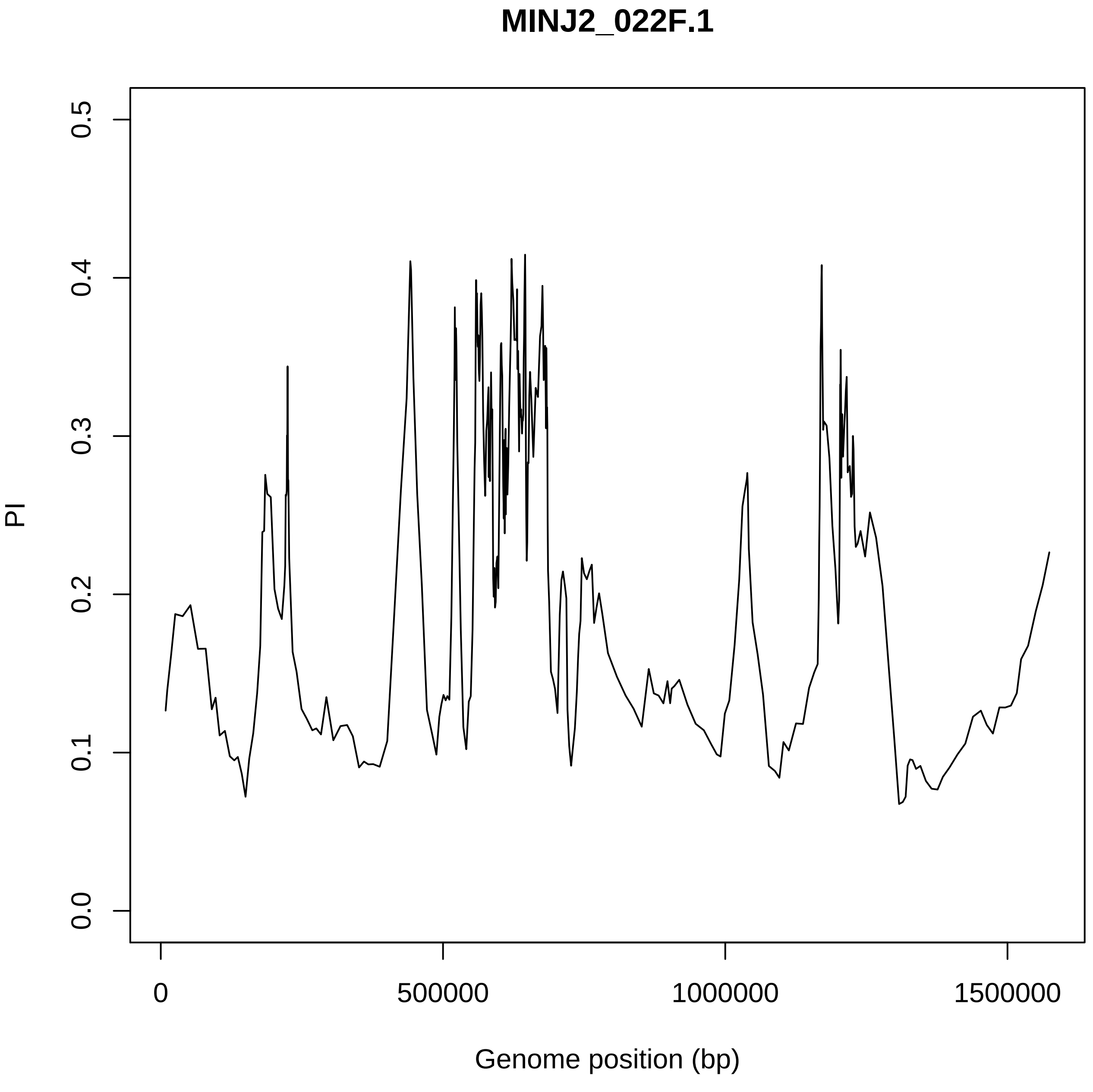

MINJ2_022F.1
PI
0.0
0.1
0.2
0.3
0.4
0.5
0
500000
1000000
1500000
Genome position (bp)

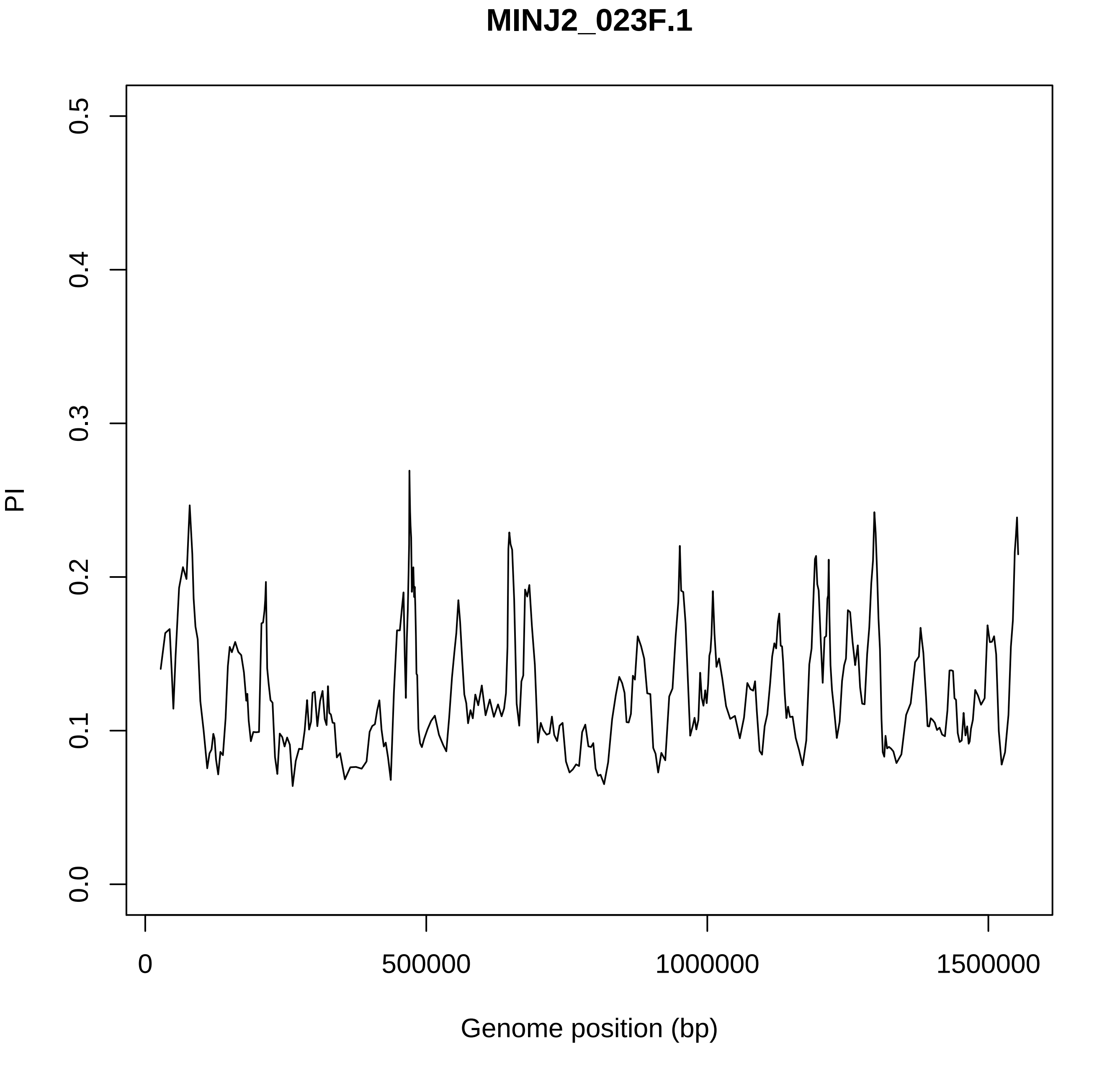
MINJ2_023F.1
PI
0.0
0.1
0.2
0.3
0.4
0.5
0
500000
1000000
1500000
Genome position (bp)

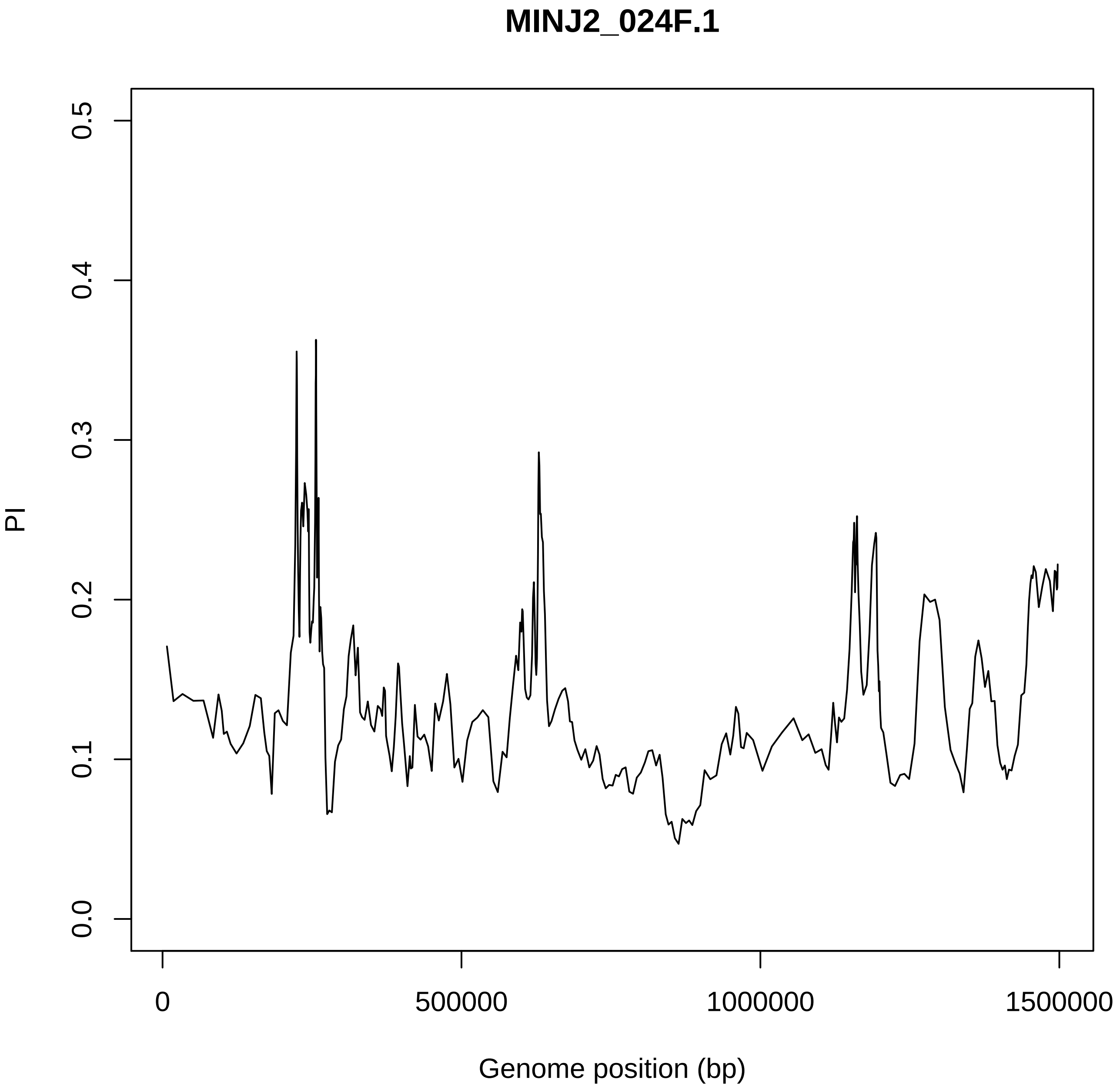
MINJ2_024F.1
PI
0.0
0.1
0.2
0.3
0.4
0.5
0
500000
1000000
1500000
Genome position (bp)

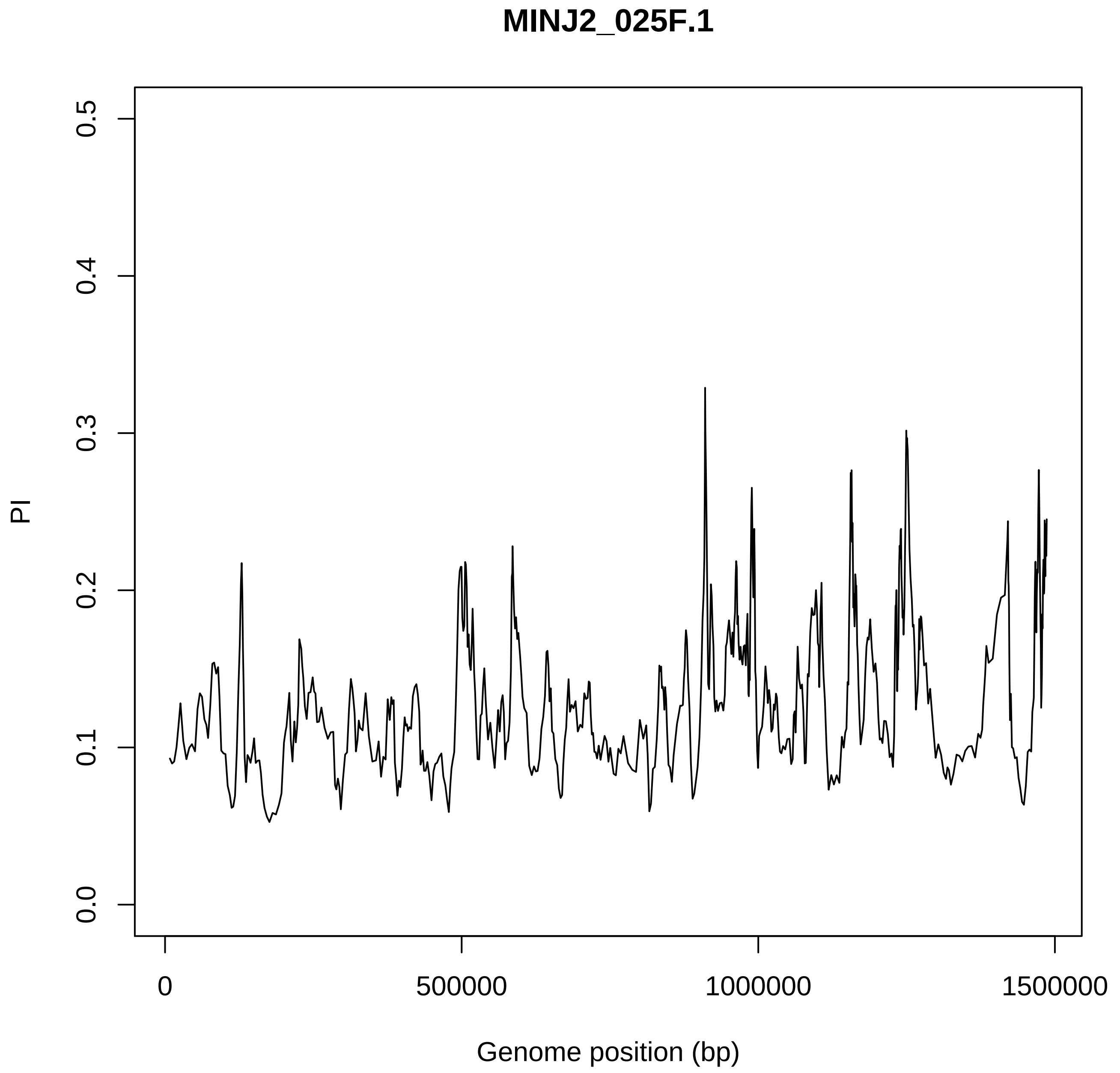

MINJ2_025F.1
PI
0.0
0.1
0.2
0.3
0.4
0.5
0
500000
1000000
1500000
Genome position (bp)

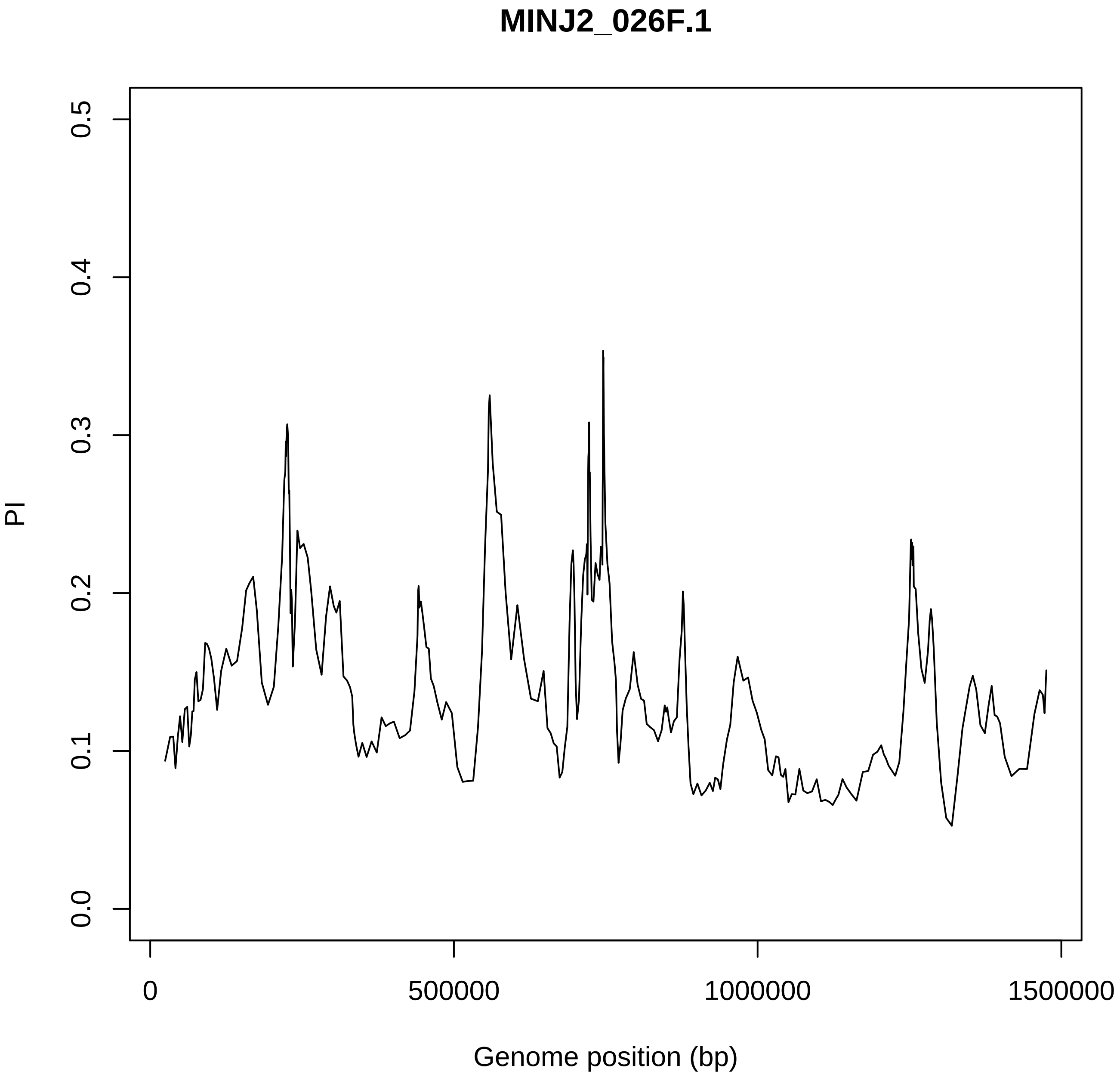

MINJ2_026F.1
PI
0.0
0.1
0.2
0.3
0.4
0.5
0
500000
1000000
1500000
Genome position (bp)

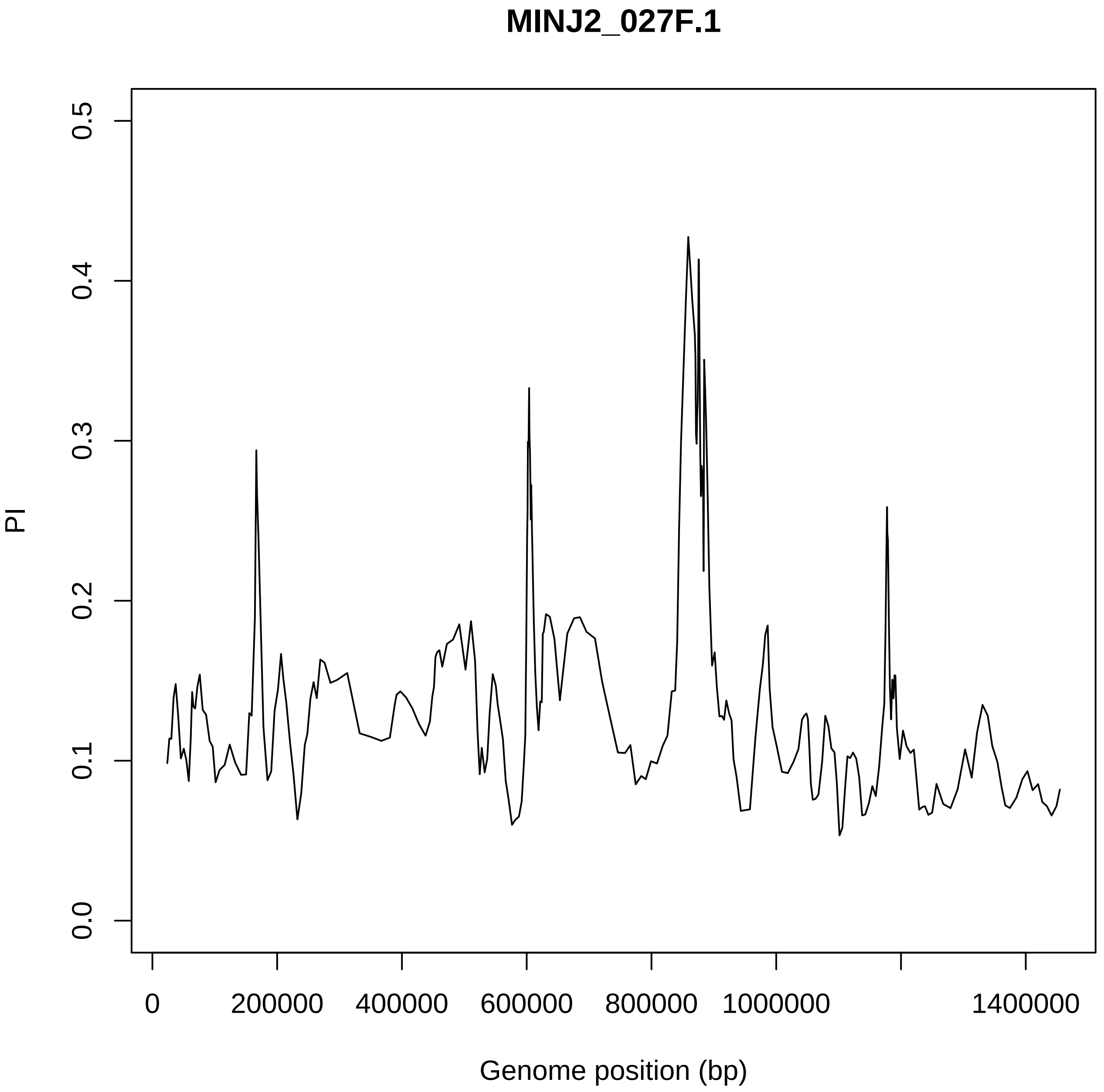

MINJ2_027F.1
PI
0.0
0.1
0.2
0.3
0.4
0.5
0
200000
400000
600000
800000
1000000
1400000
Genome position (bp)

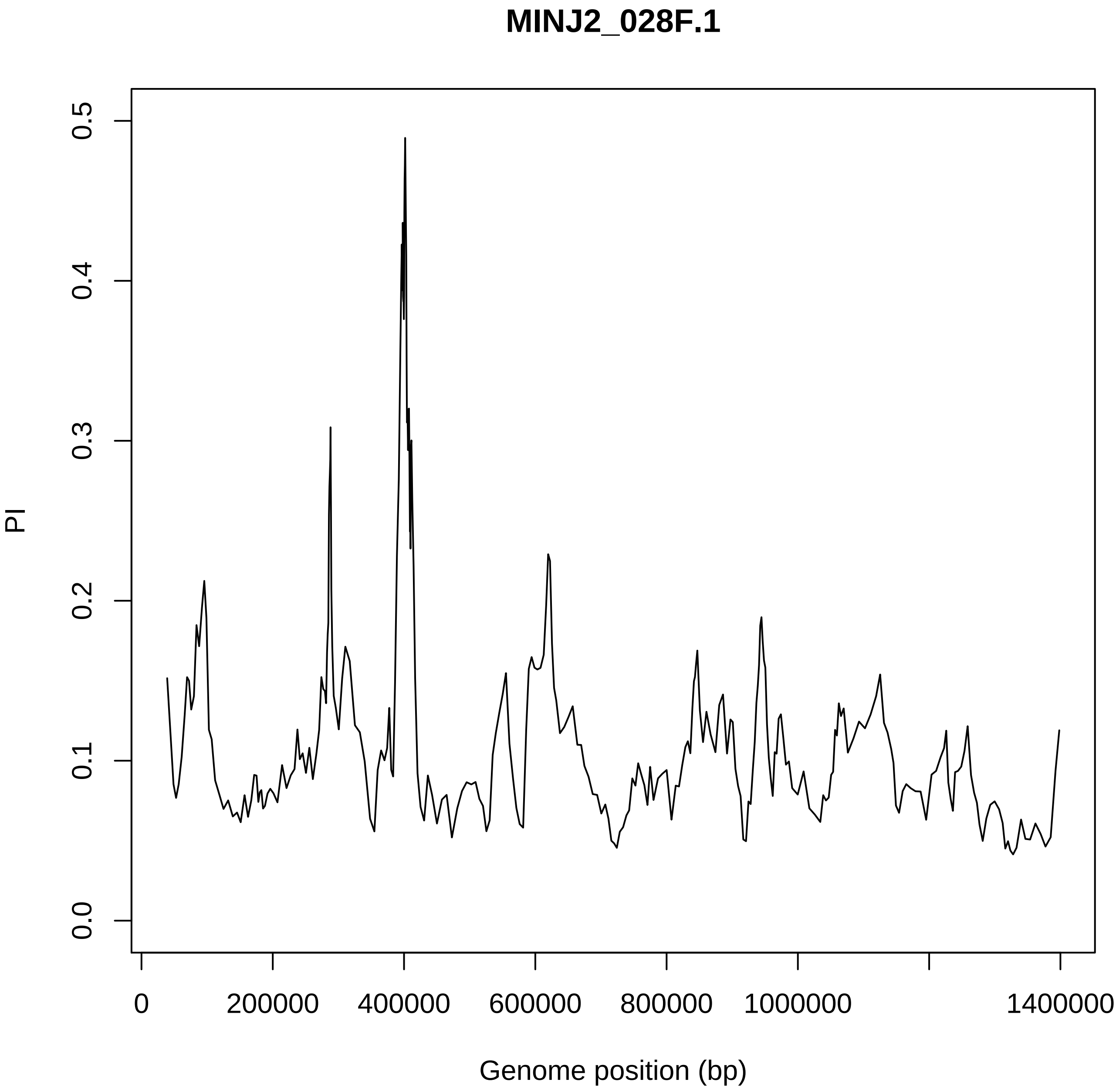
MINJ2_028F.1
PI
0.0
0.1
0.2
0.3
0.4
0.5
0
200000
400000
600000
800000
1000000
1400000
Genome position (bp)

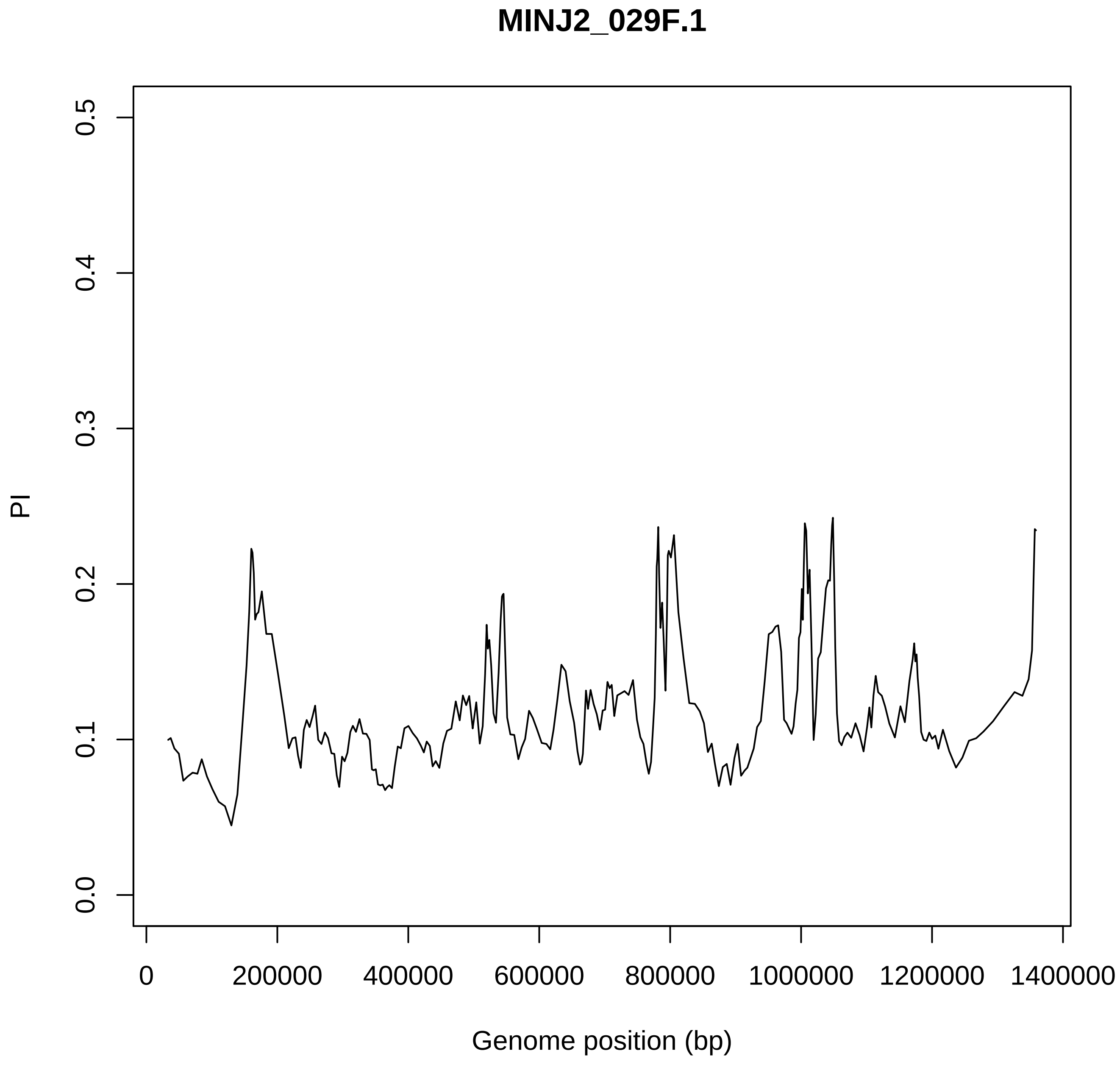
MINJ2_029F.1
PI
0.0
0.1
0.2
0.3
0.4
0.5
0
200000
400000
600000
800000
1000000
1200000
1400000
Genome position (bp)

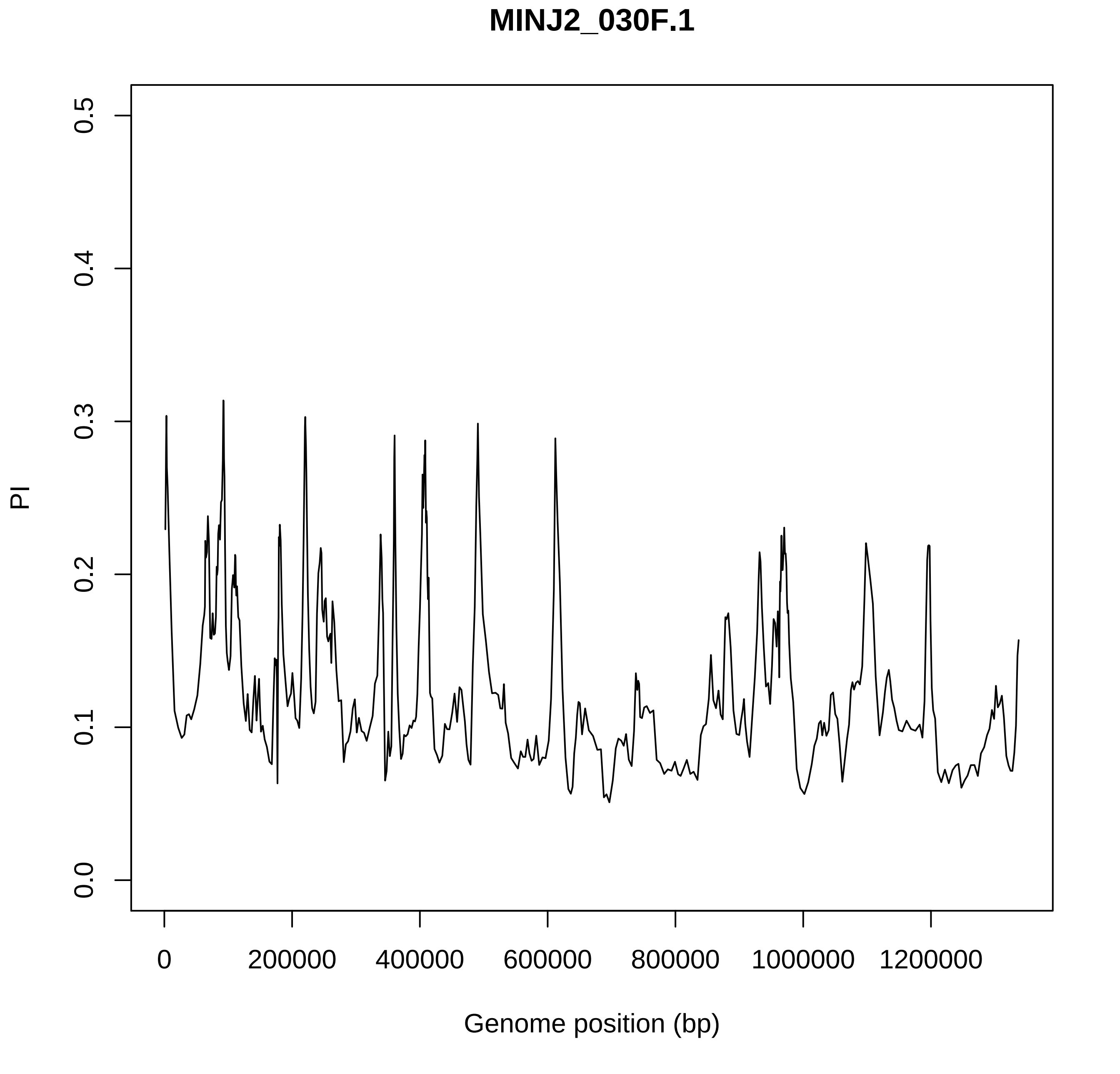
MINJ2_030F.1
PI
0.0
0.1
0.2
0.3
0.4
0.5
0
200000
400000
600000
800000
1000000
1200000
Genome position (bp)

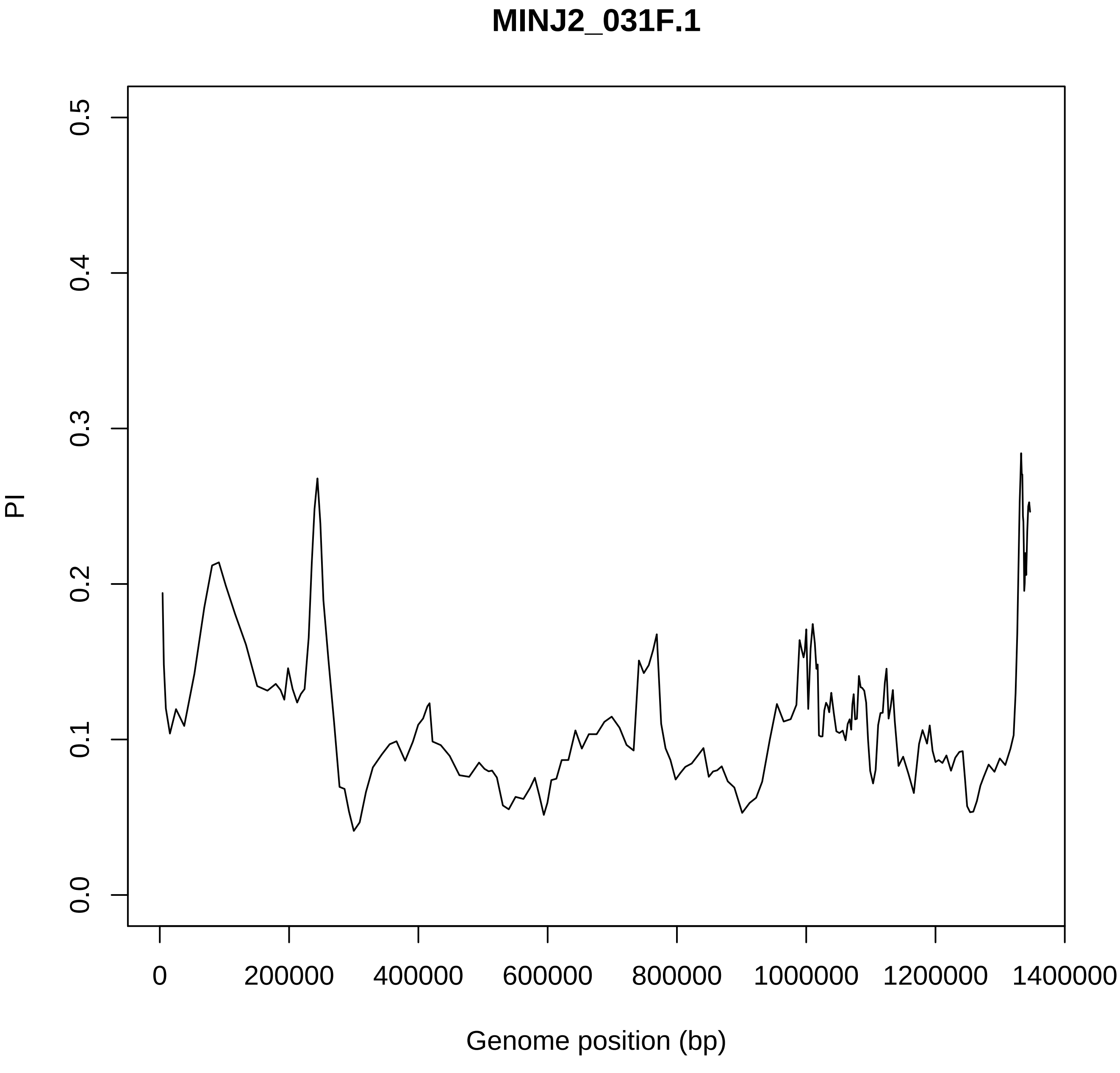

MINJ2_031F.1
PI
0.0
0.1
0.2
0.3
0.4
0.5
0
200000
400000
600000
800000
1000000
1200000
1400000
Genome position (bp)

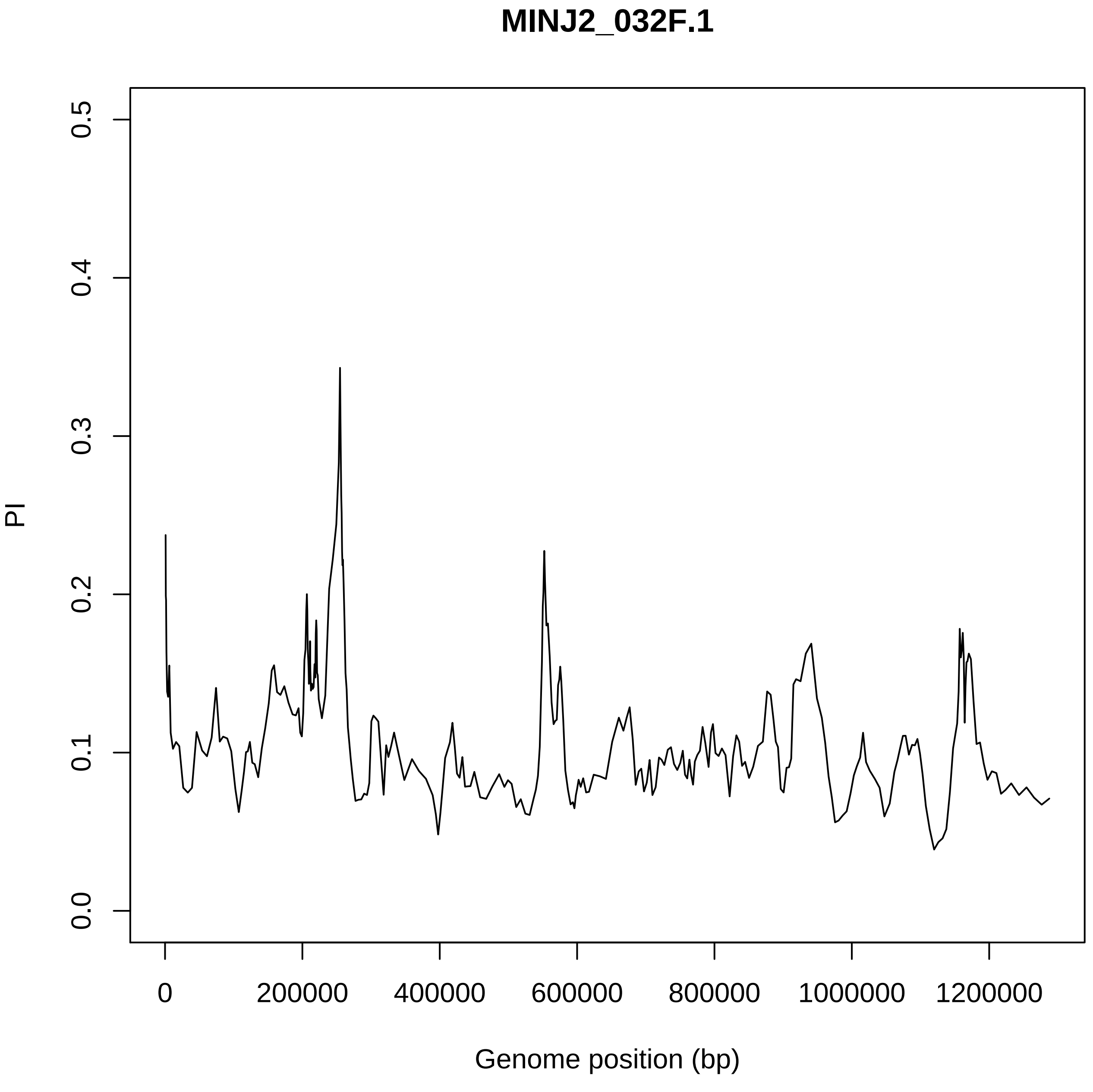
MINJ2_032F.1
PI
0.0
0.1
0.2
0.3
0.4
0.5
0
200000
400000
600000
800000
1000000
1200000
Genome position (bp)

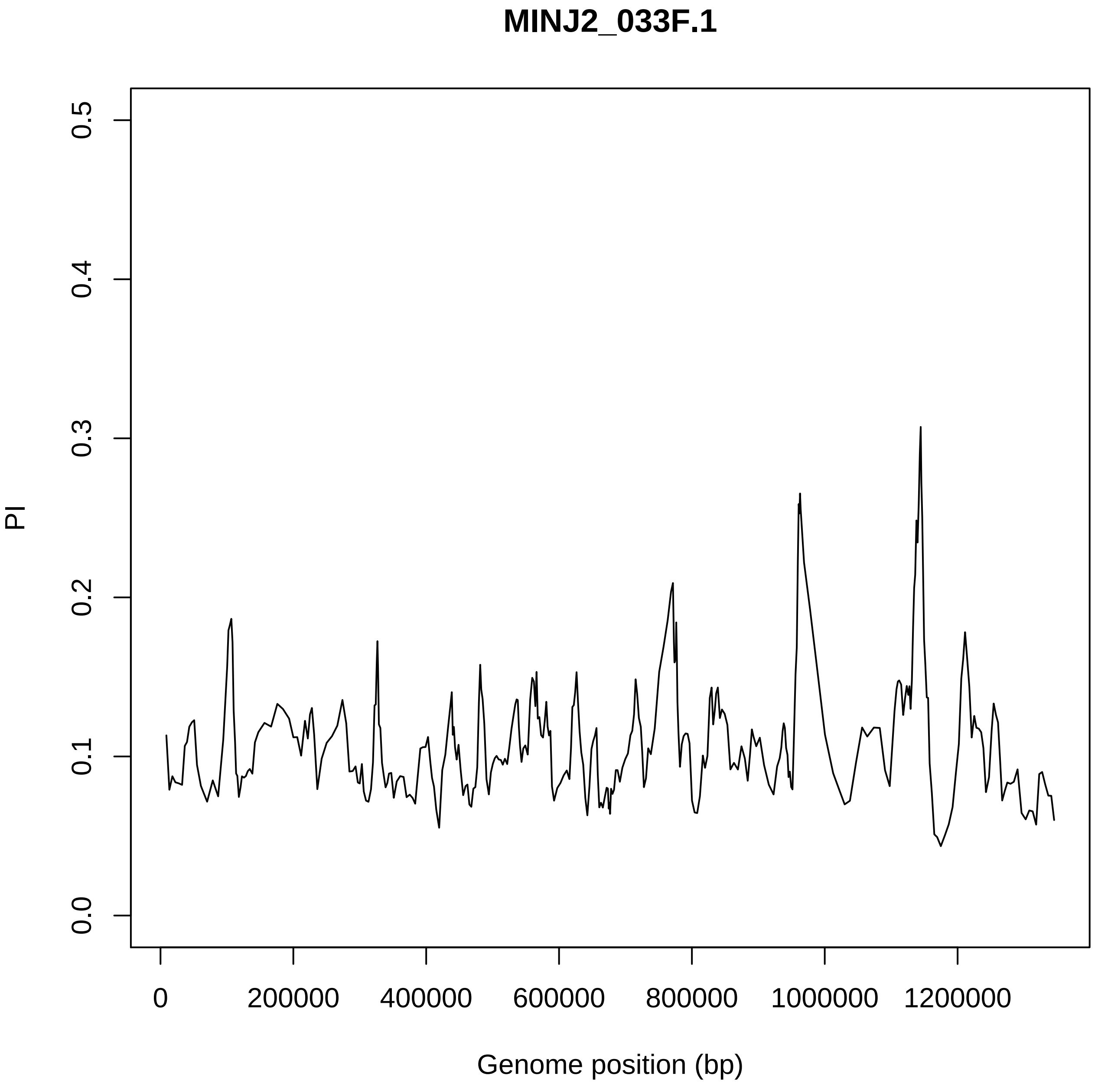

MINJ2_033F.1
PI
0.0
0.1
0.2
0.3
0.4
0.5
0
200000
400000
600000
800000
1000000
1200000
Genome position (bp)

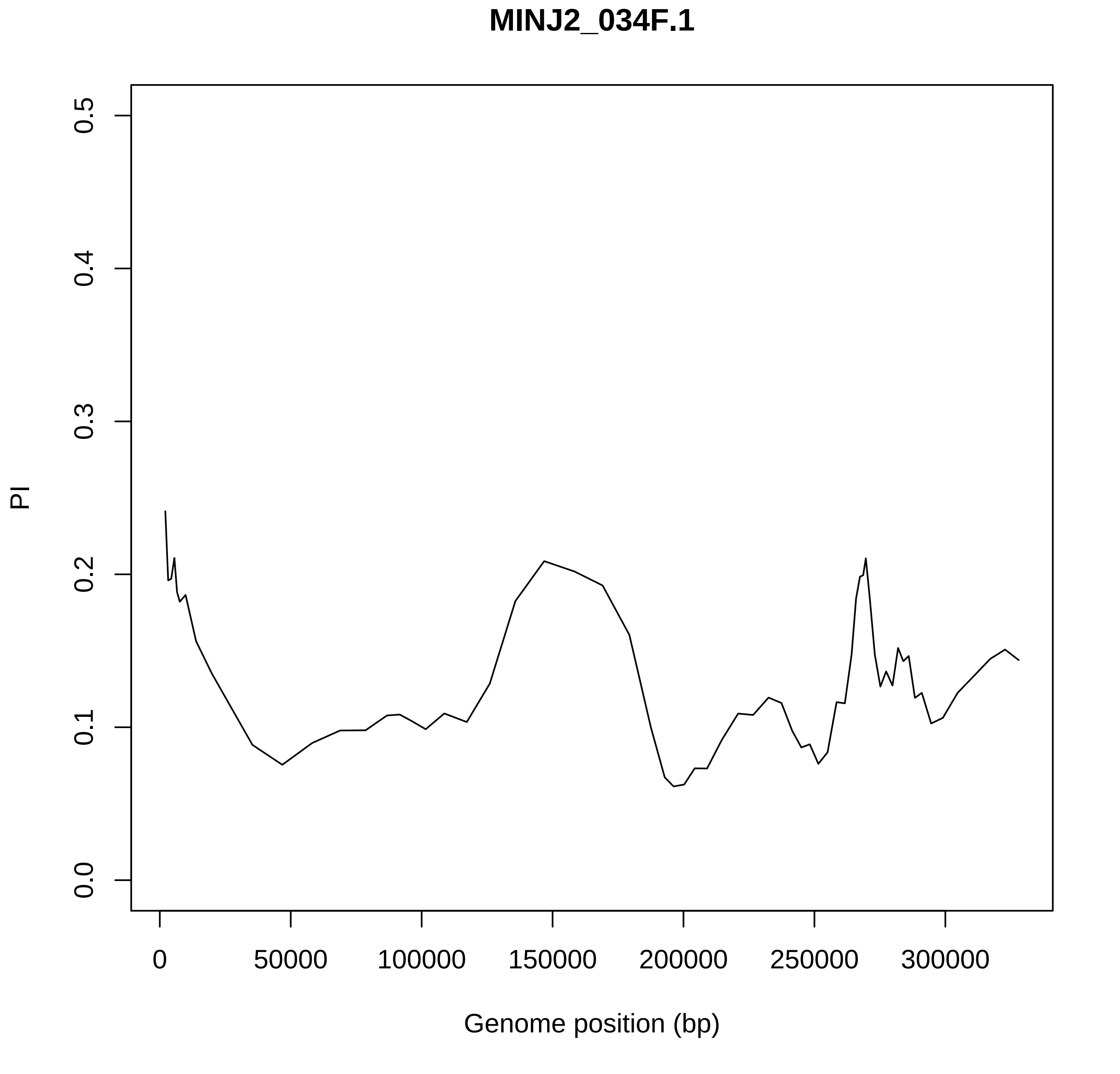
MINJ2_034F.1
PI
0.0
0.1
0.2
0.3
0.4
0.5
0
50000
100000
150000
200000
250000
300000
Genome position (bp)

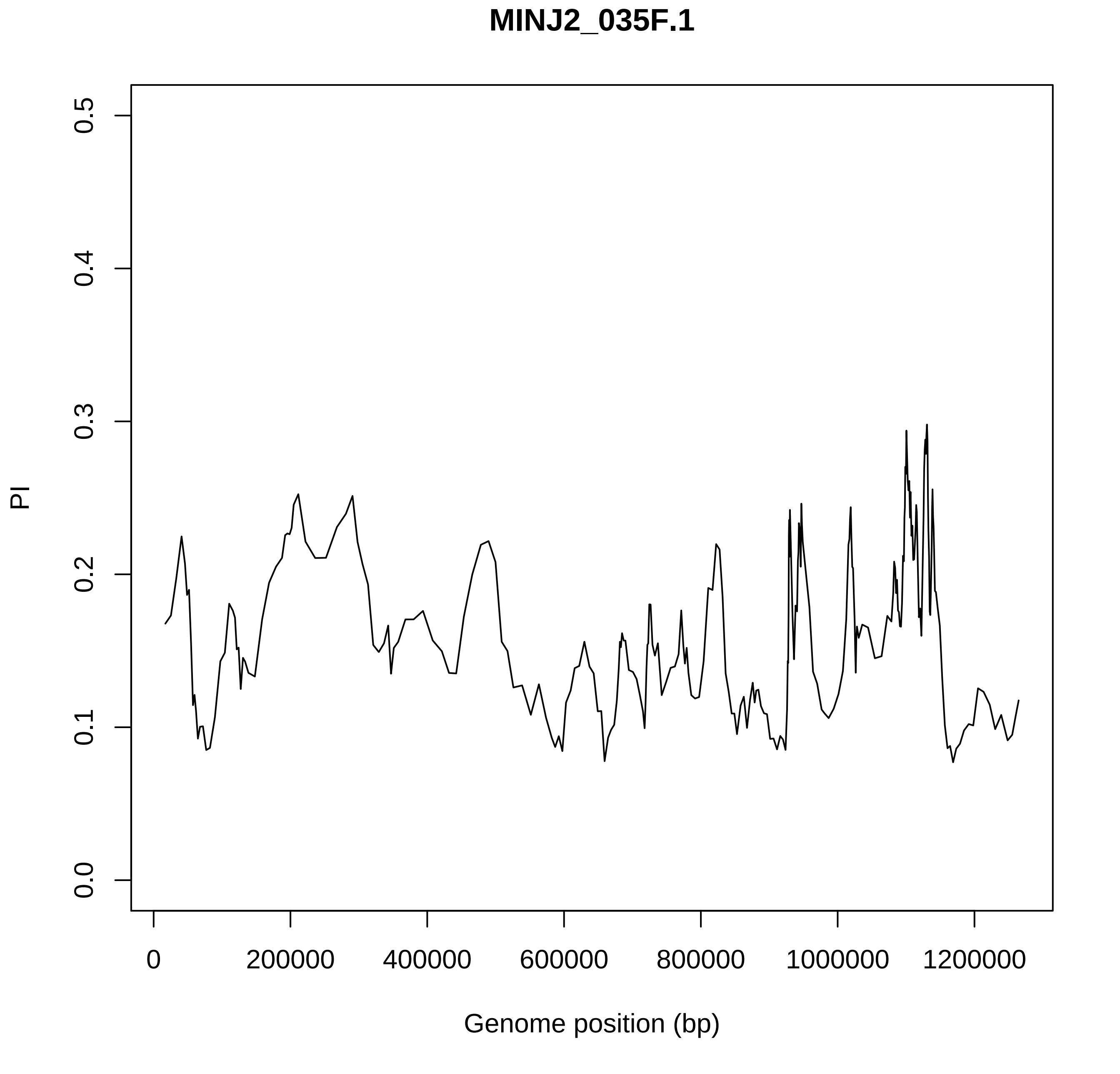

MINJ2_035F.1
PI
0.0
0.1
0.2
0.3
0.4
0.5
0
200000
400000
600000
800000
1000000
1200000
Genome position (bp)

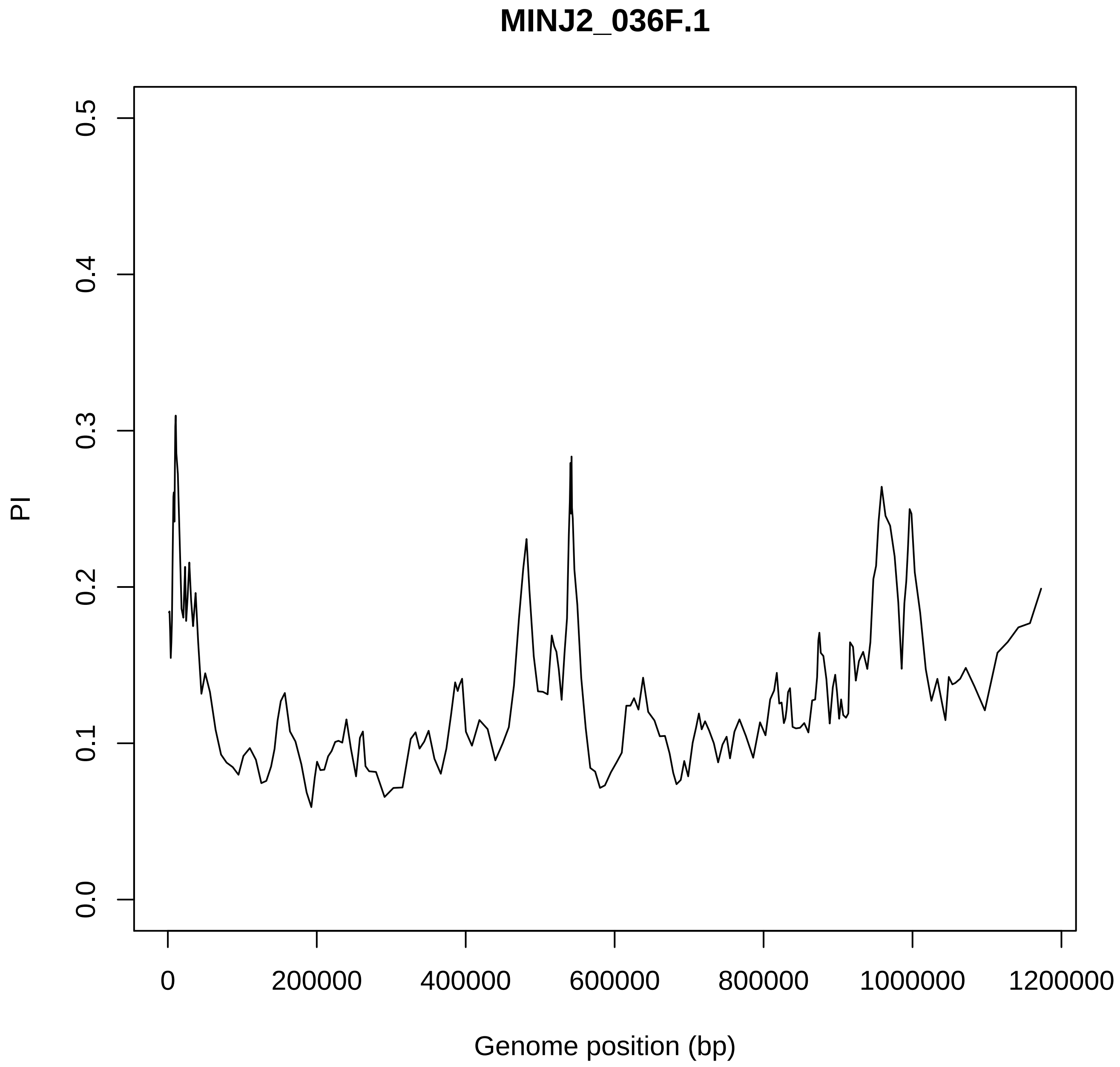

MINJ2_036F.1
PI
0.0
0.1
0.2
0.3
0.4
0.5
0
200000
400000
600000
800000
1000000
1200000
Genome position (bp)

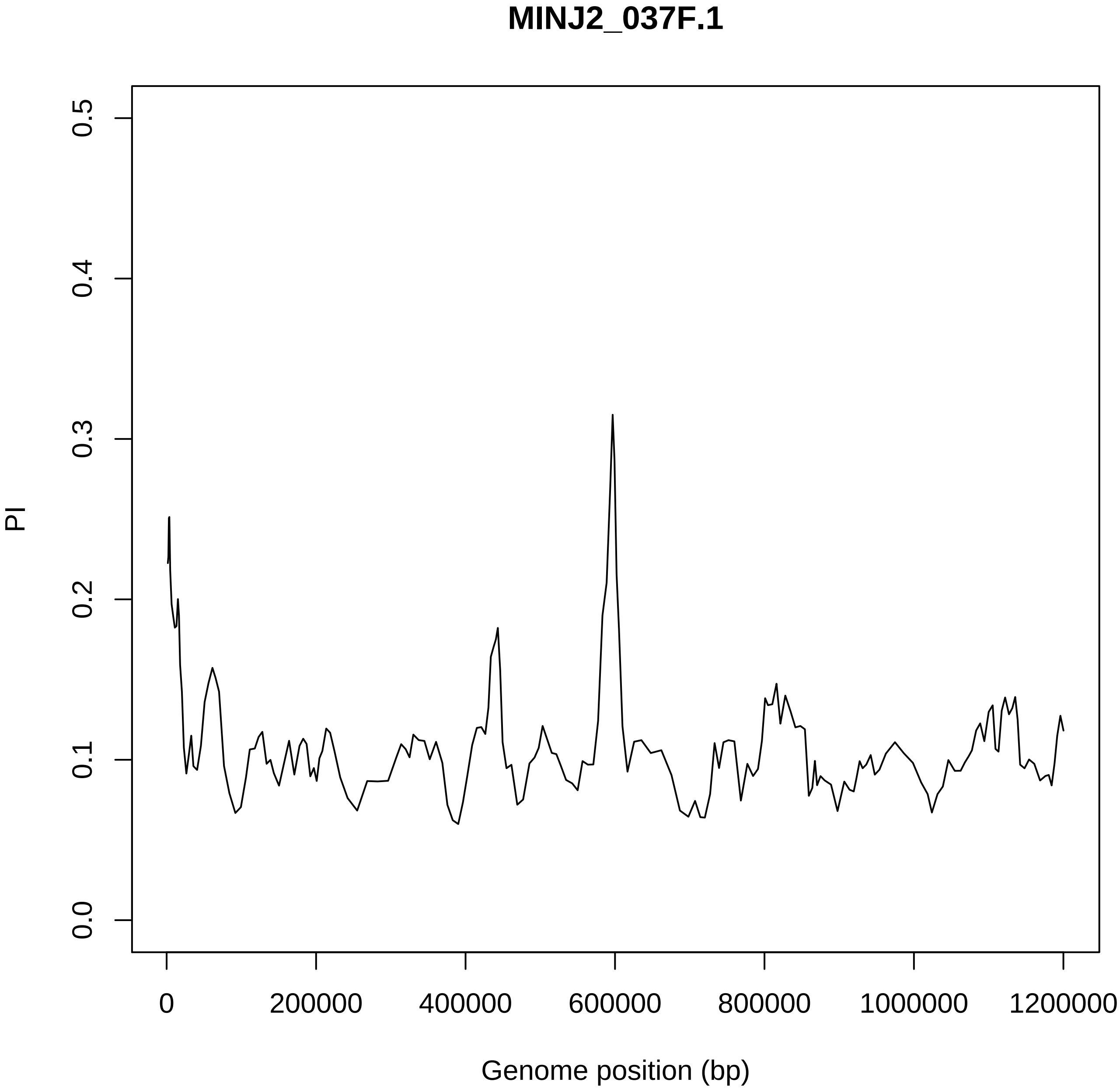

MINJ2_037F.1
PI
0.0
0.1
0.2
0.3
0.4
0.5
0
200000
400000
600000
800000
1000000
1200000
Genome position (bp)

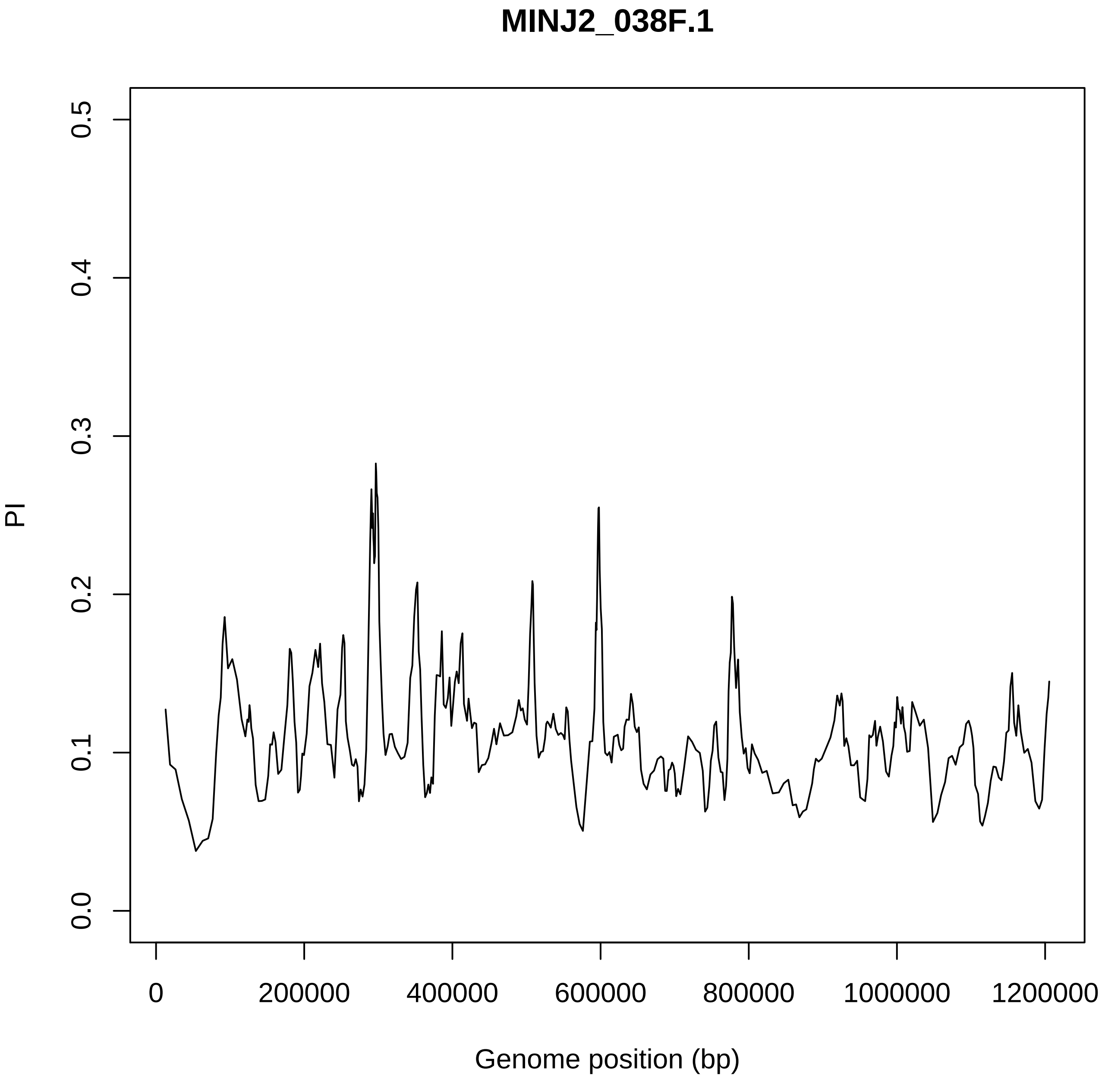
MINJ2_038F.1
PI
0.0
0.1
0.2
0.3
0.4
0.5
0
200000
400000
600000
800000
1000000
1200000
Genome position (bp)

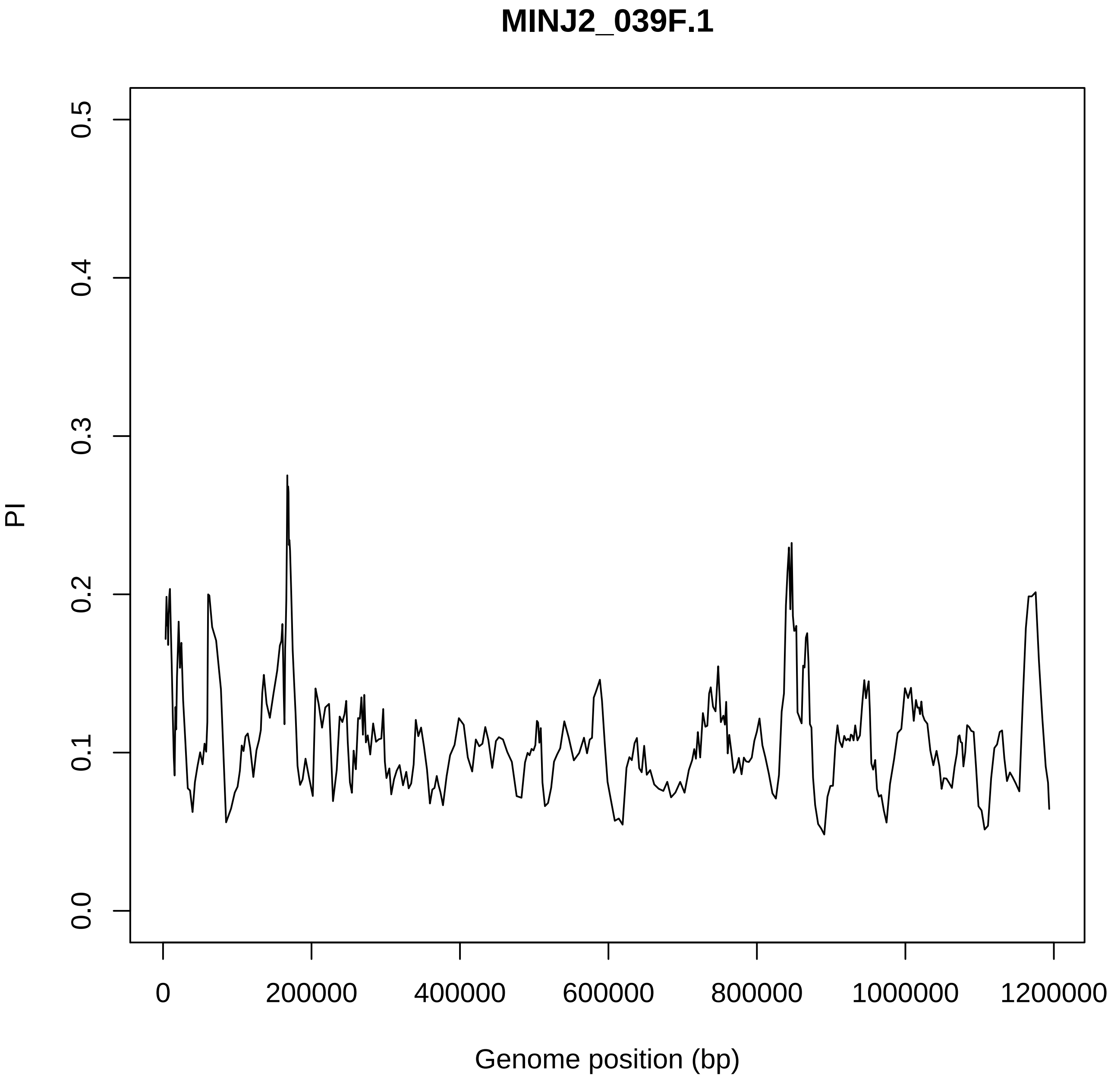
MINJ2_039F.1
PI
0.0
0.1
0.2
0.3
0.4
0.5
0
200000
400000
600000
800000
1000000
1200000
Genome position (bp)

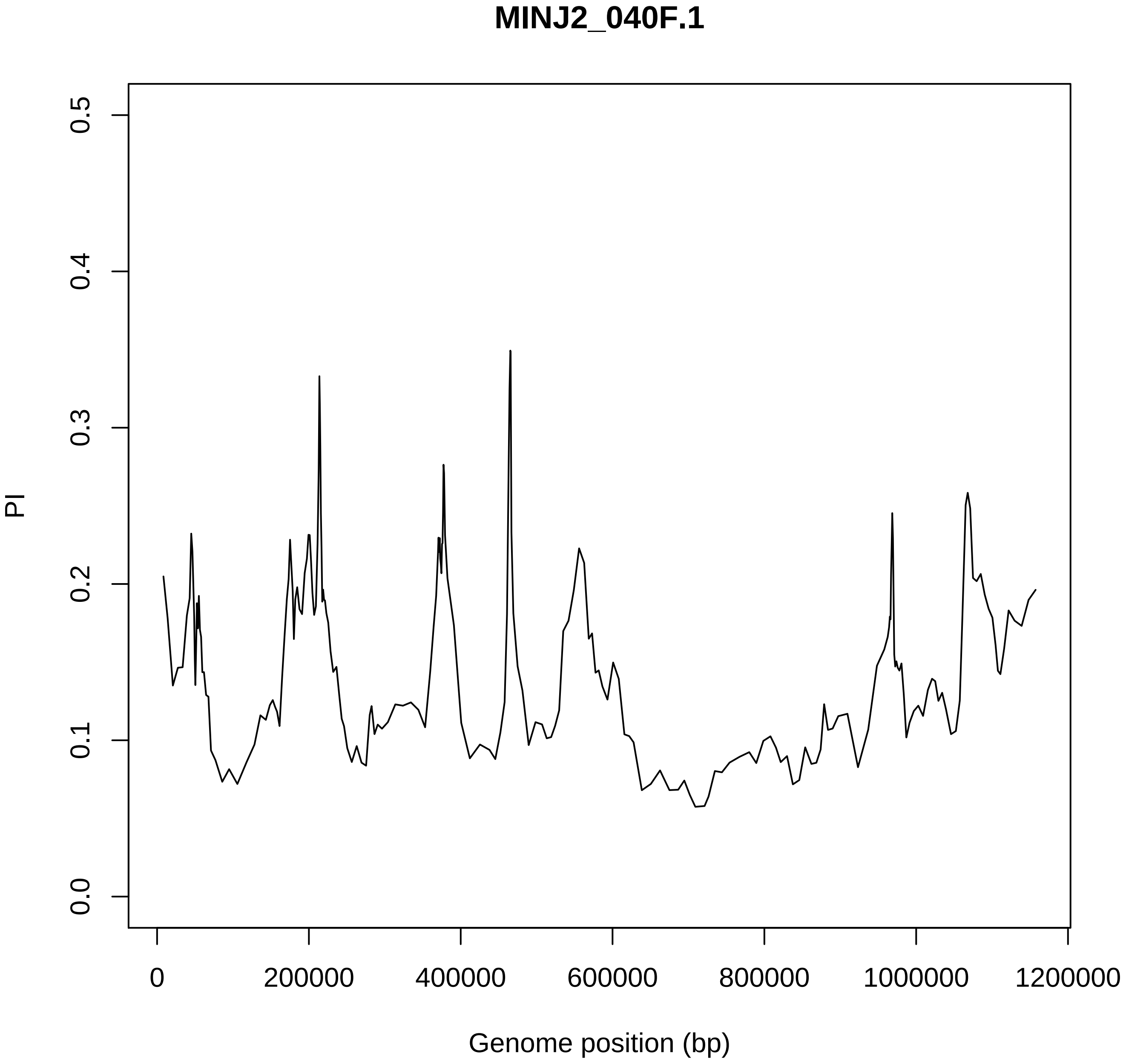
MINJ2_040F.1
PI
0.0
0.1
0.2
0.3
0.4
0.5
0
200000
400000
600000
800000
1000000
1200000
Genome position (bp)

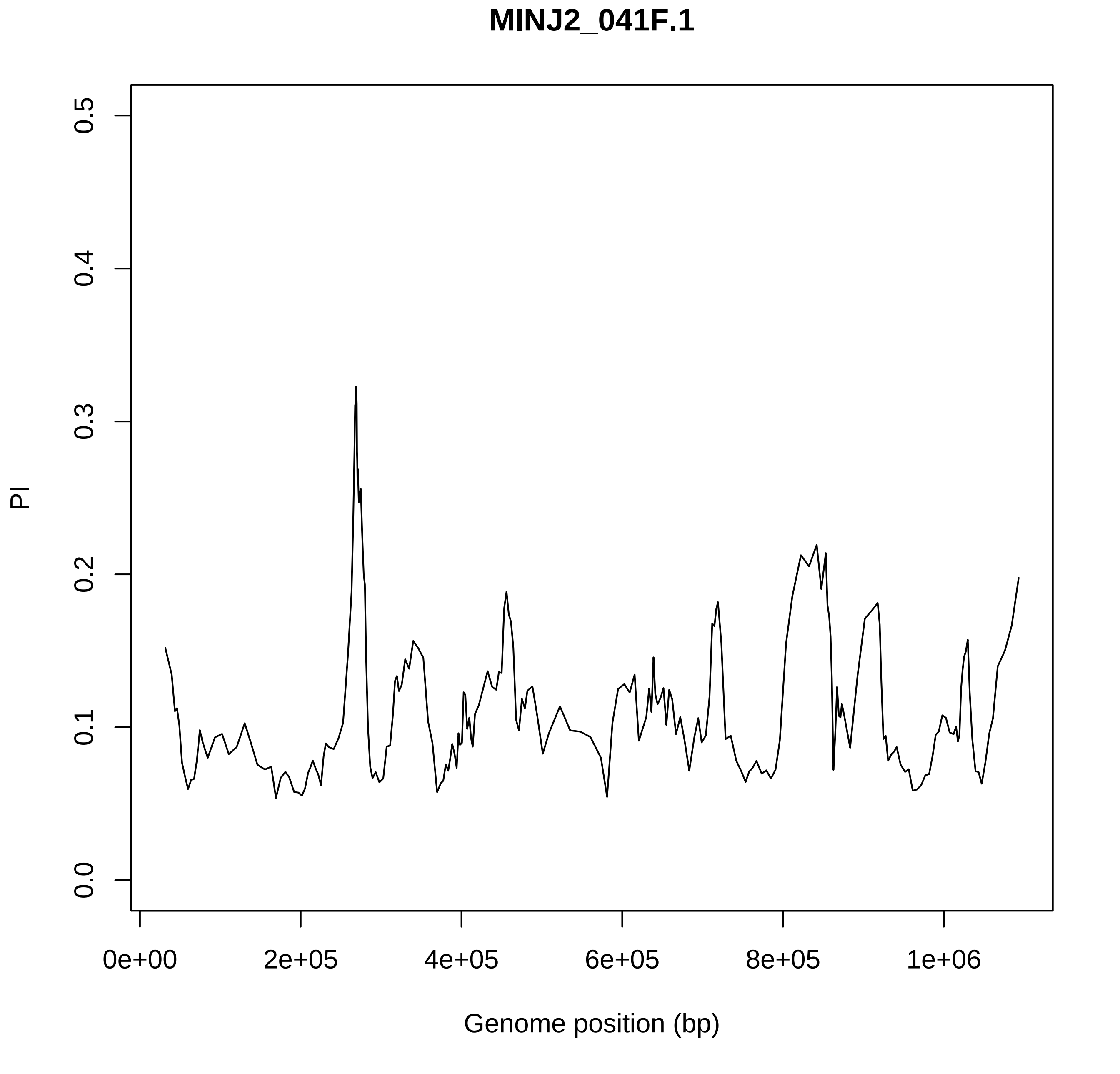
MINJ2_041F.1
PI
0.0
0.1
0.2
0.3
0.4
0.5
0e+00
2e+05
4e+05
6e+05
8e+05
1e+06
Genome position (bp)

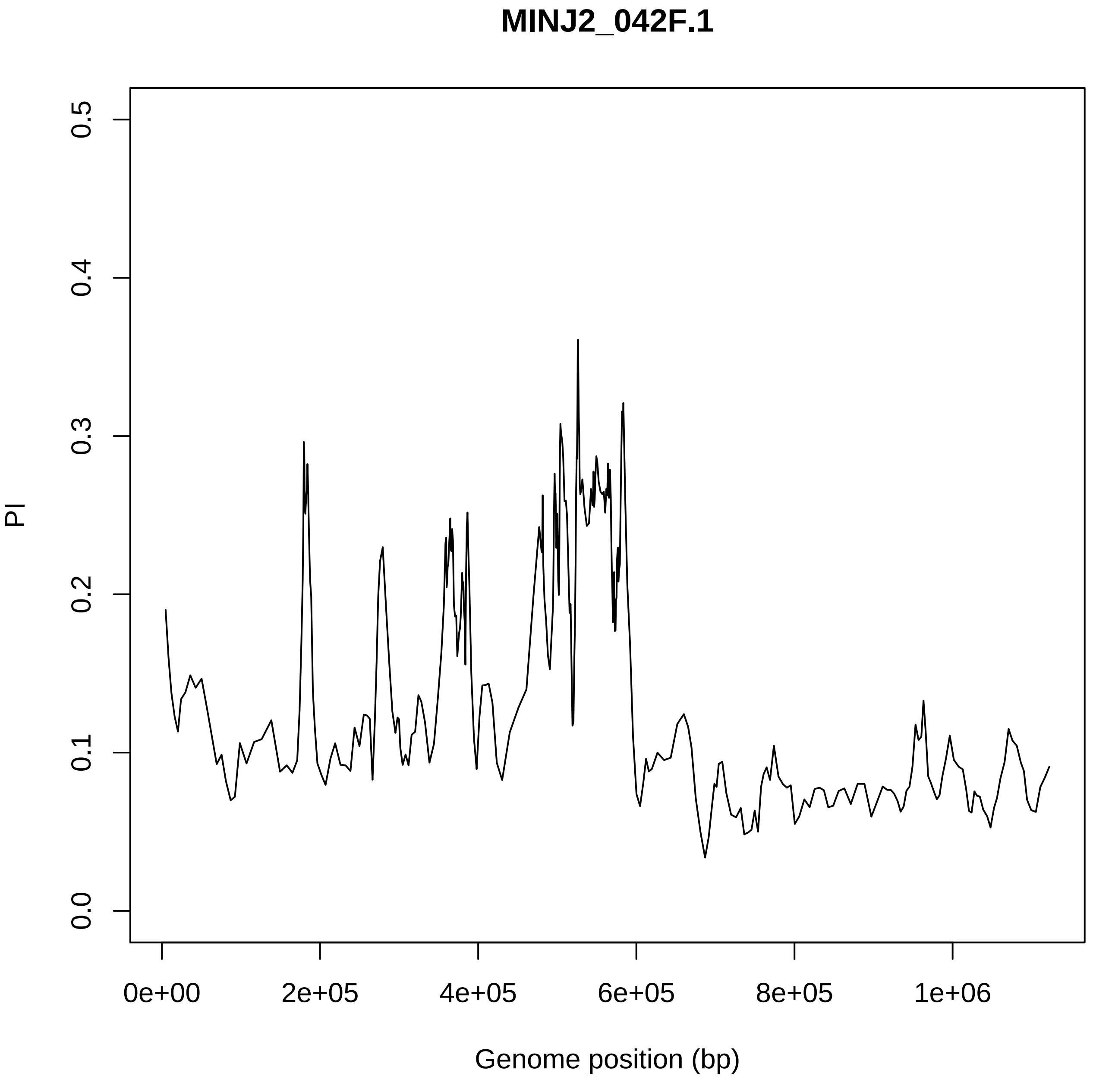
MINJ2_042F.1
PI
0.0
0.1
0.2
0.3
0.4
0.5
0e+00
2e+05
4e+05
6e+05
8e+05
1e+06
Genome position (bp)

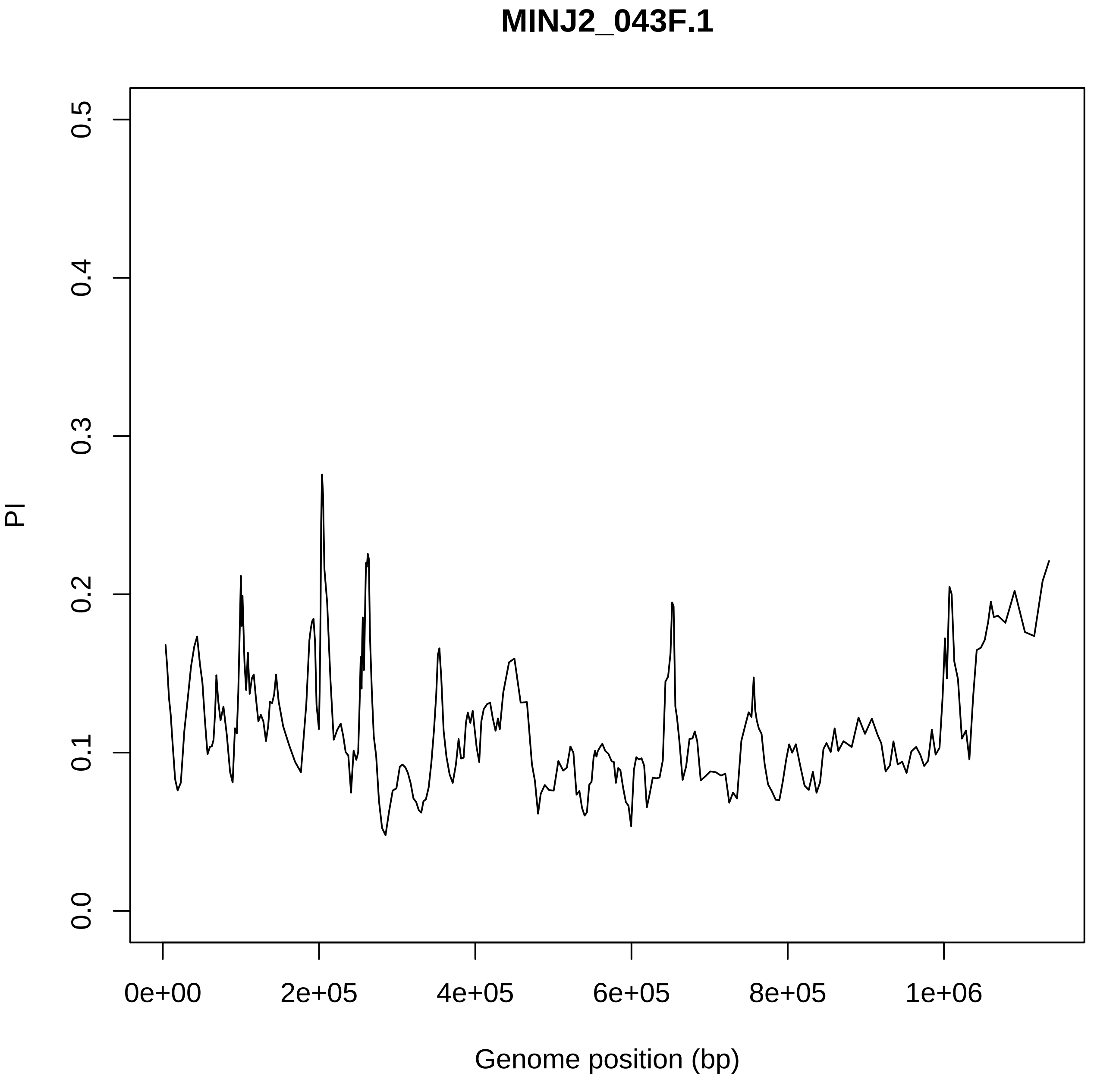
MINJ2_043F.1
PI
0.0
0.1
0.2
0.3
0.4
0.5
0e+00
2e+05
4e+05
6e+05
8e+05
1e+06
Genome position (bp)

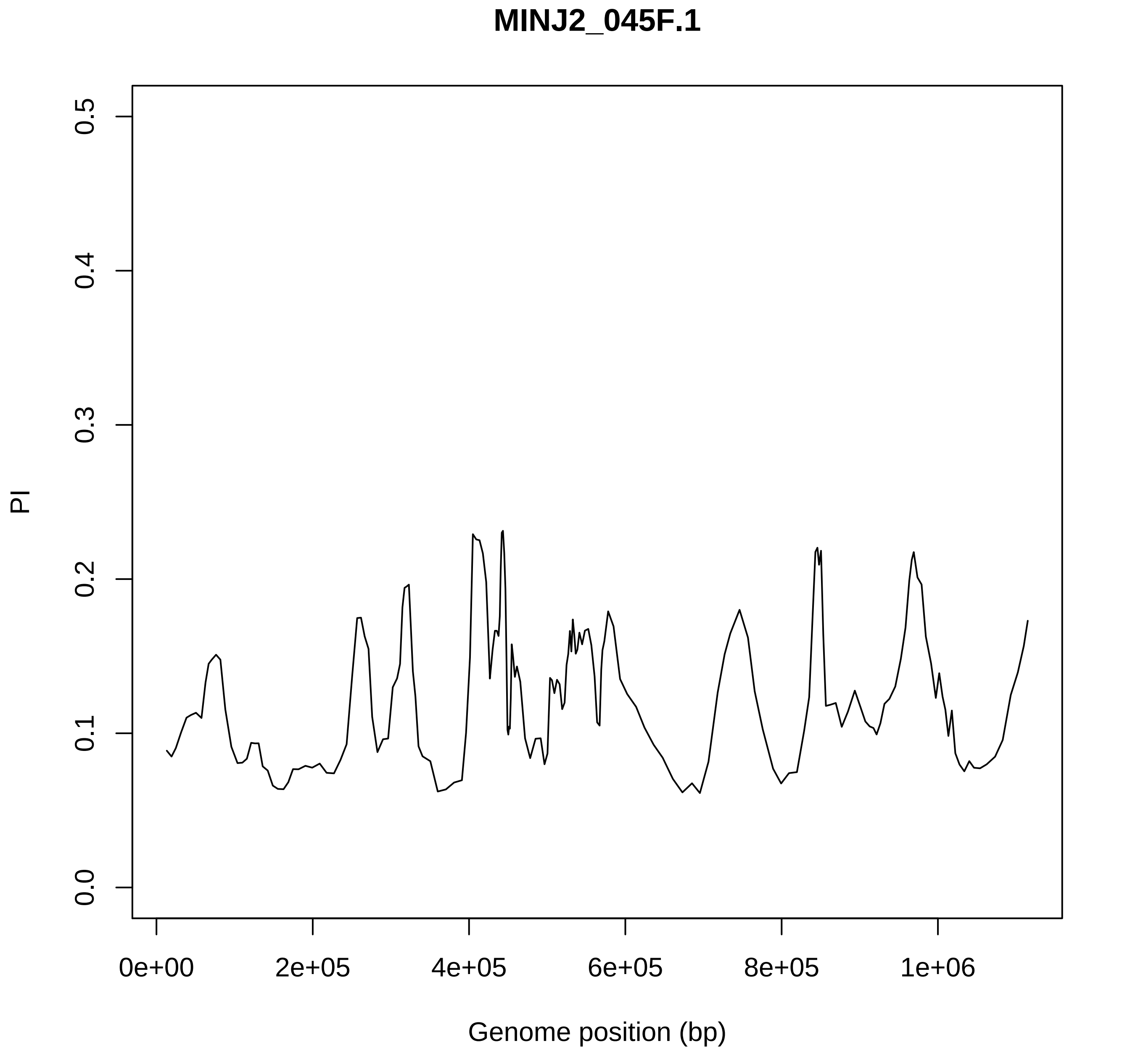
MINJ2_045F.1
PI
0.0
0.1
0.2
0.3
0.4
0.5
0e+00
2e+05
4e+05
6e+05
8e+05
1e+06
Genome position (bp)

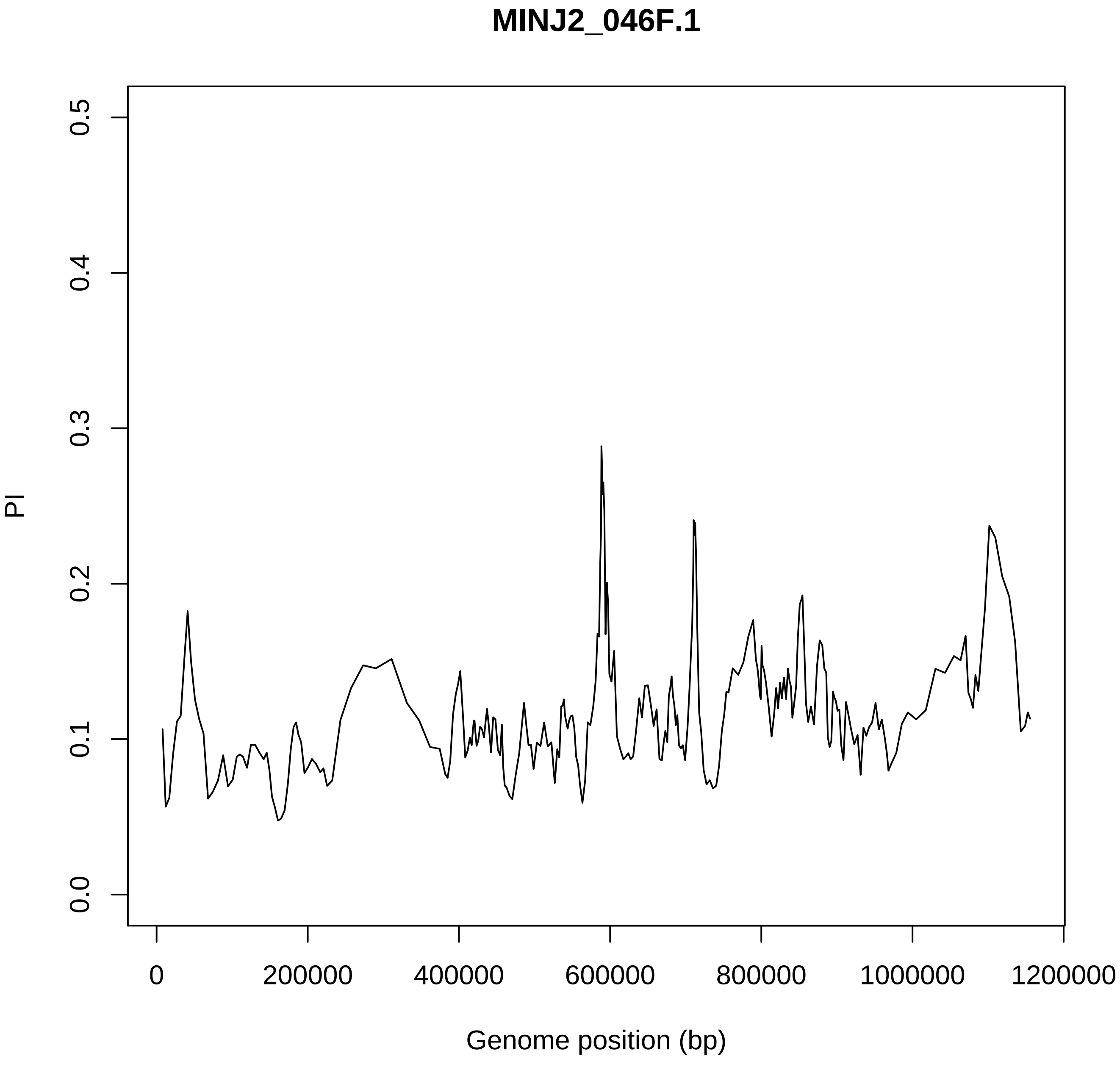

MINJ2_046F.1
PI
0.0
0.1
0.2
0.3
0.4
0.5
0
200000
400000
600000
800000
1000000
1200000
Genome position (bp)

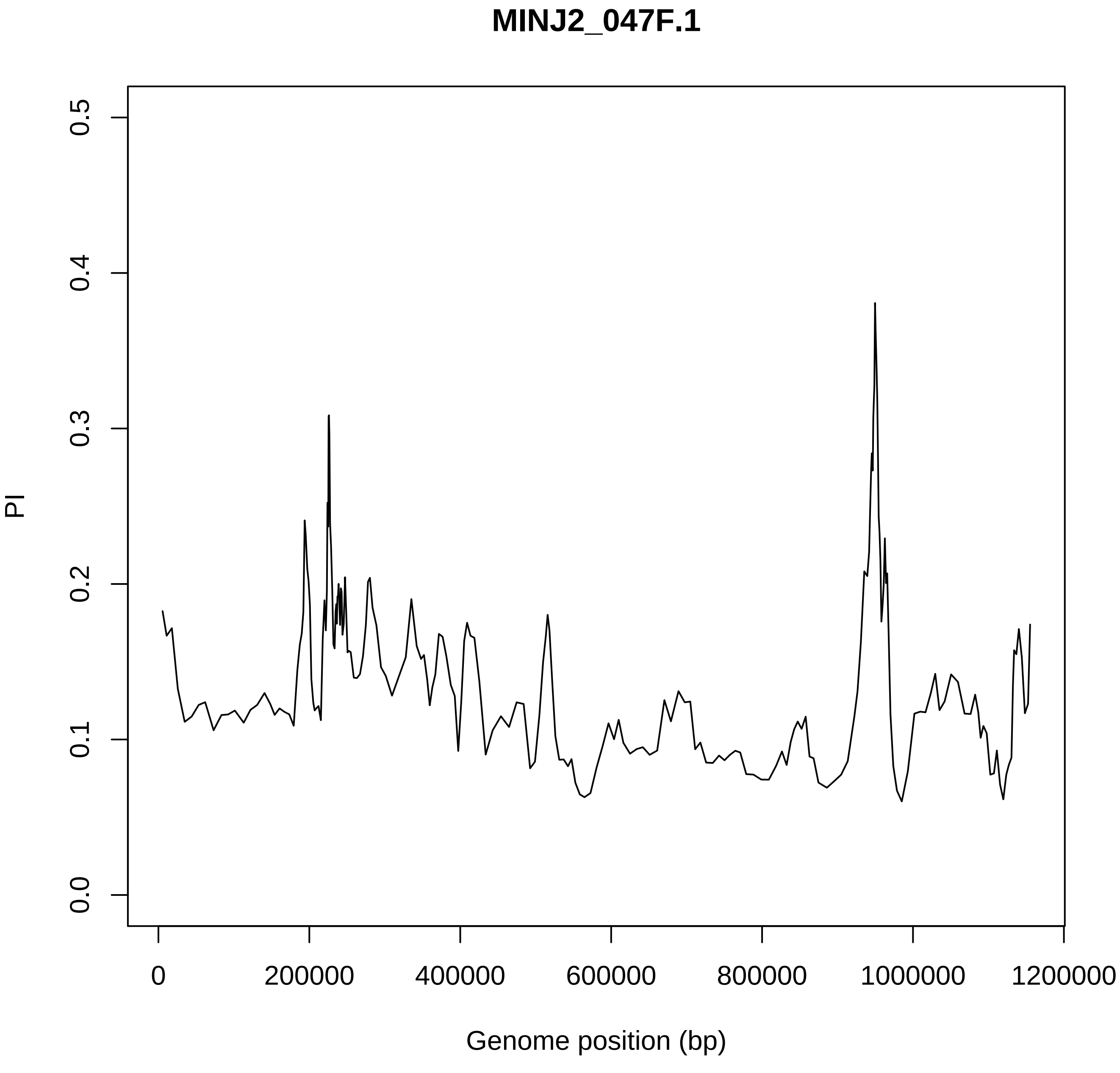
MINJ2_047F.1
PI
0.0
0.1
0.2
0.3
0.4
0.5
0
200000
400000
600000
800000
1000000
1200000
Genome position (bp)

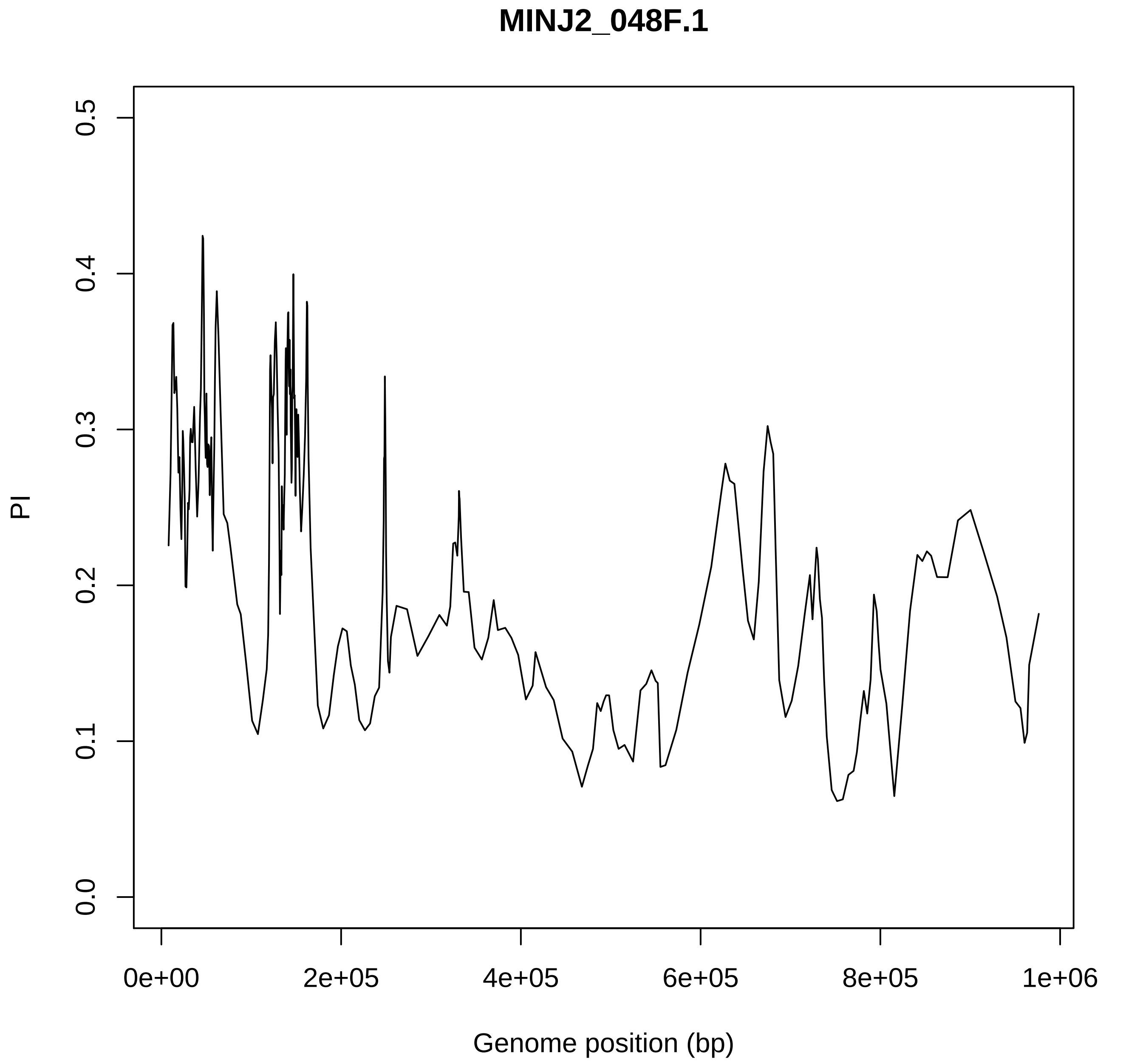
MINJ2_048F.1
PI
0.0
0.1
0.2
0.3
0.4
0.5
0e+00
2e+05
4e+05
6e+05
8e+05
1e+06
Genome position (bp)

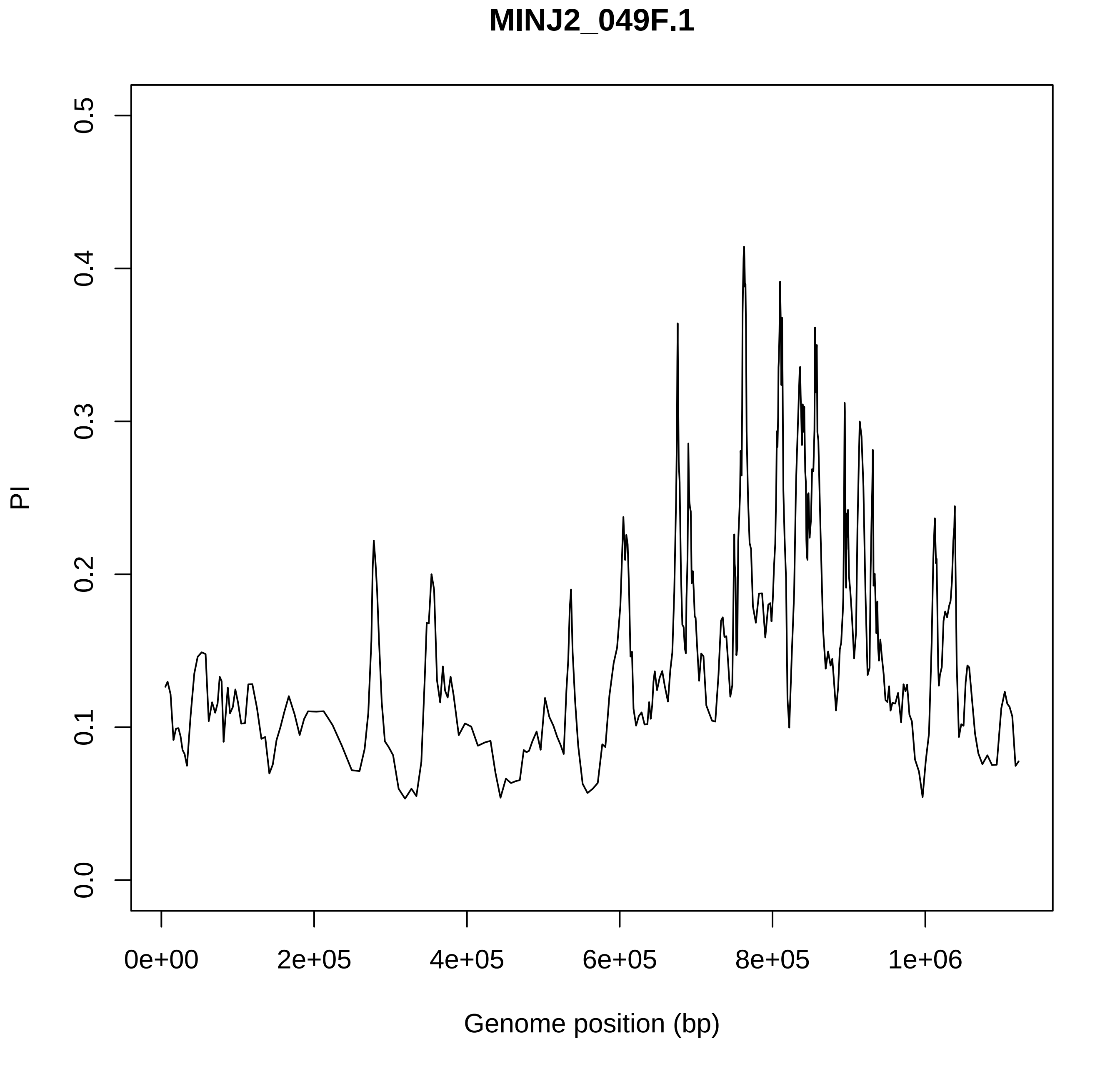

MINJ2_049F.1
PI
0.0
0.1
0.2
0.3
0.4
0.5
0e+00
2e+05
4e+05
6e+05
8e+05
1e+06
Genome position (bp)

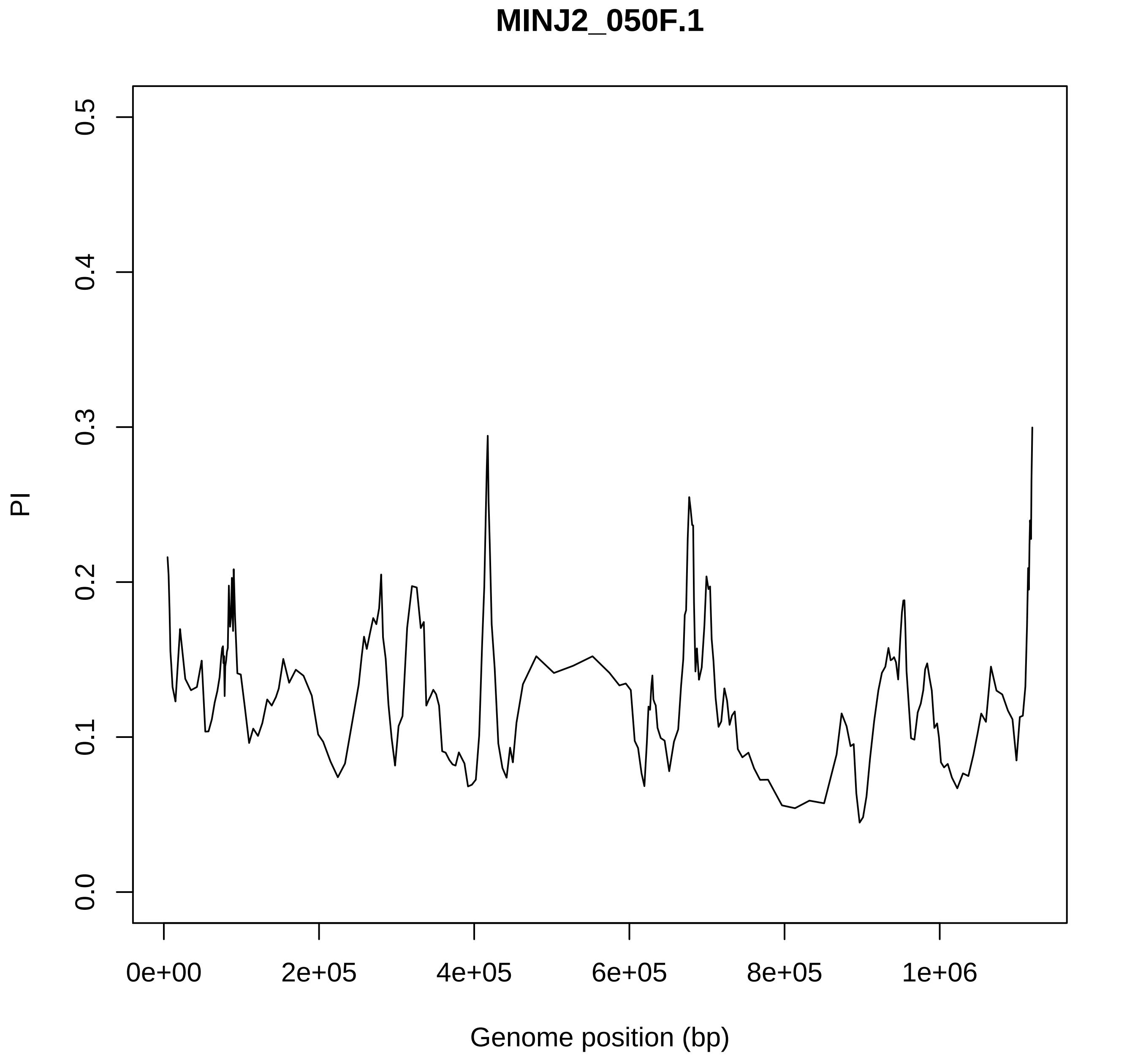

MINJ2_050F.1
PI
0.0
0.1
0.2
0.3
0.4
0.5
0e+00
2e+05
4e+05
6e+05
8e+05
1e+06
Genome position (bp)

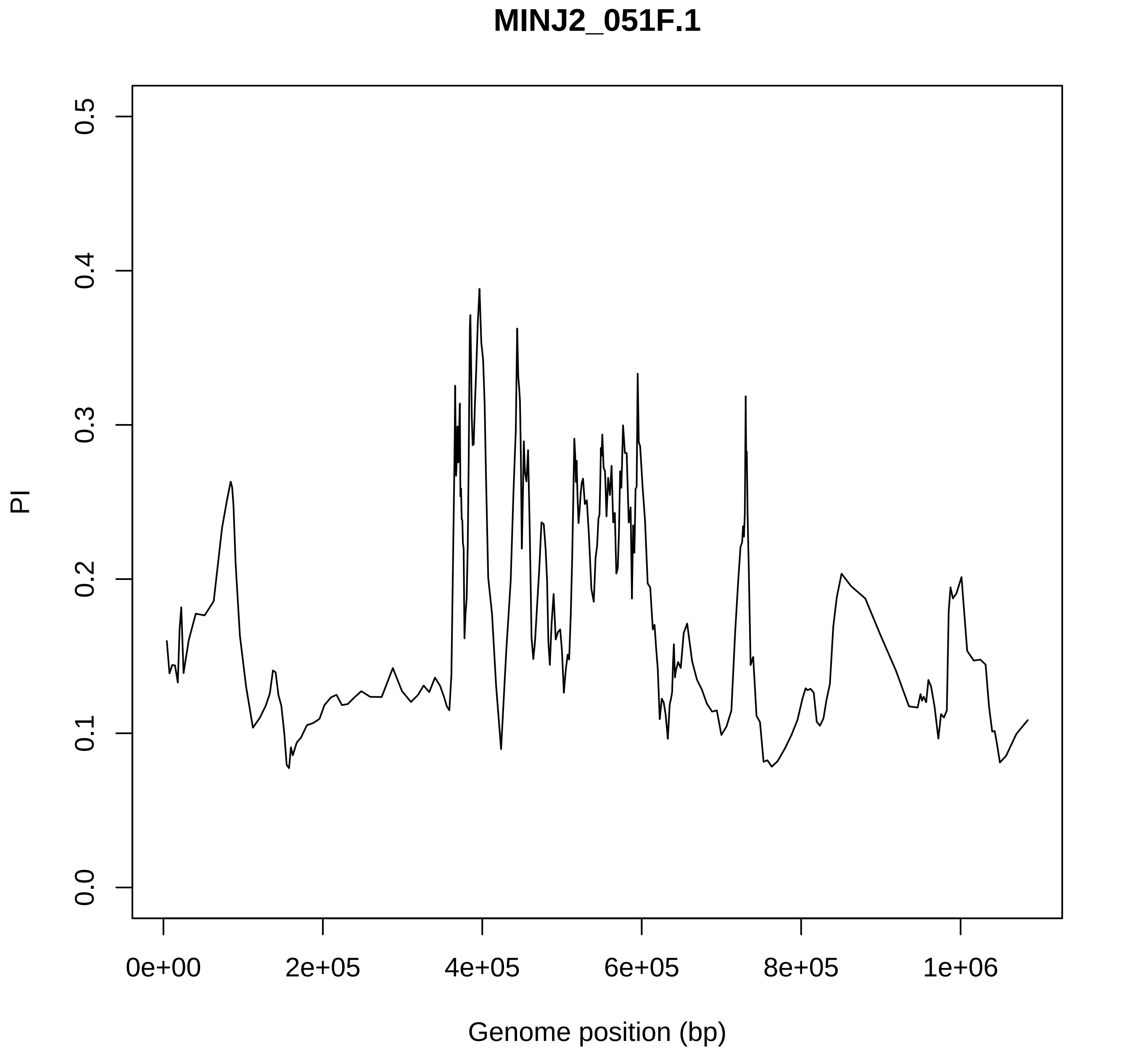
MINJ2_051F.1
PI
0.0
0.1
0.2
0.3
0.4
0.5
0e+00
2e+05
4e+05
6e+05
8e+05
1e+06
Genome position (bp)

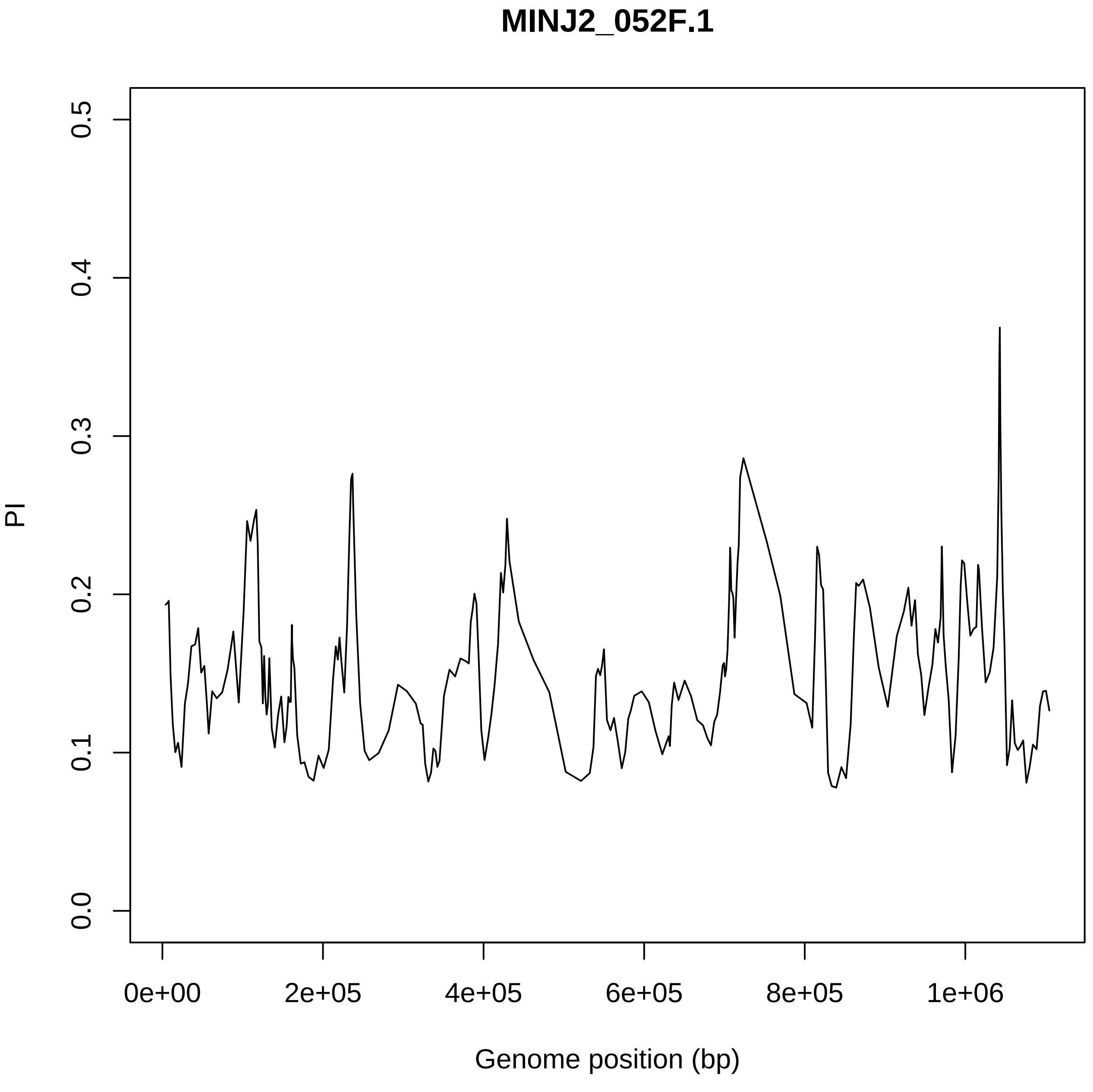
MINJ2_052F.1
PI
0.0
0.1
0.2
0.3
0.4
0.5
0e+00
2e+05
4e+05
6e+05
8e+05
1e+06
Genome position (bp)

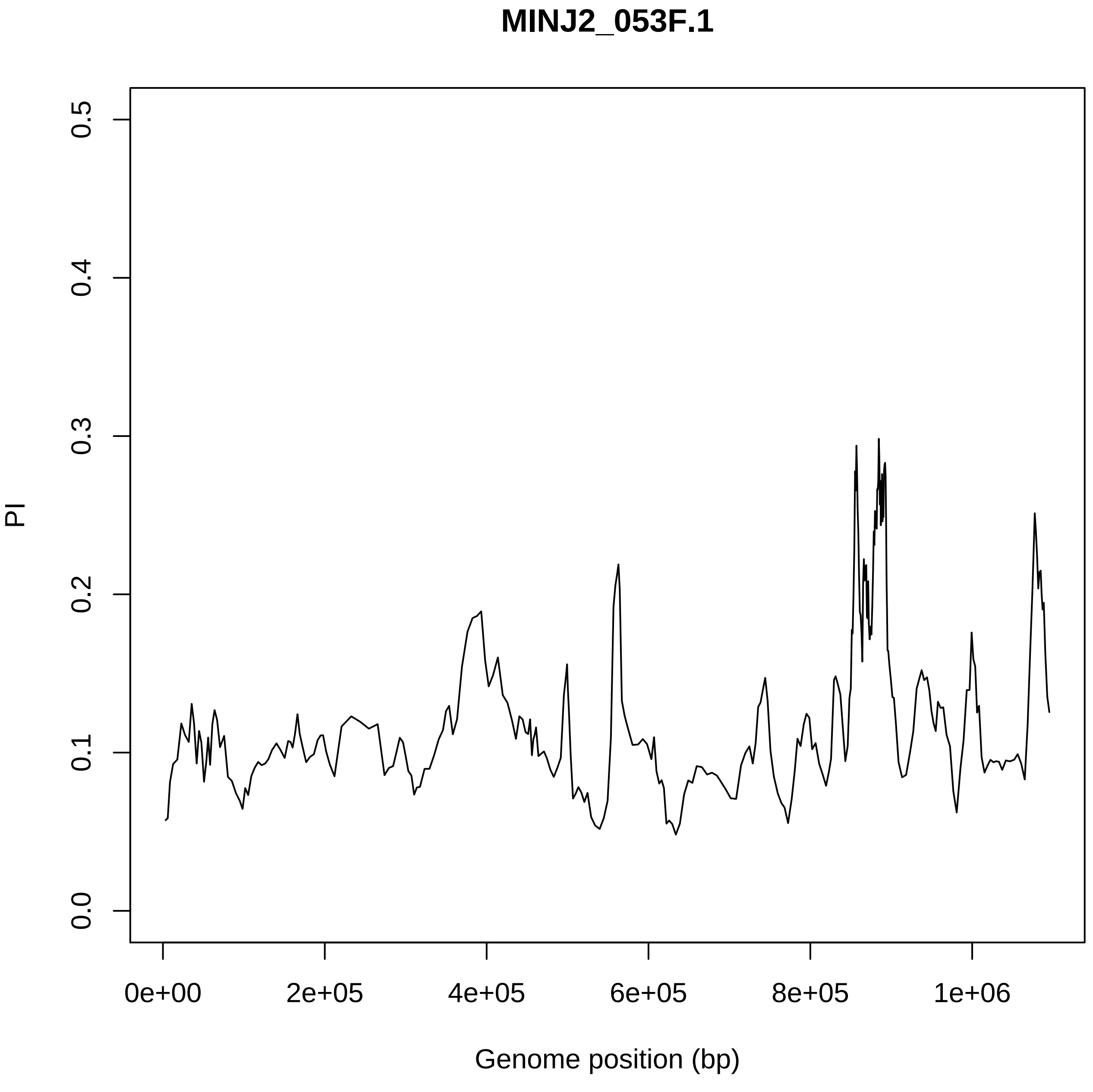

MINJ2_053F.1
PI
0.0
0.1
0.2
0.3
0.4
0.5
0e+00
2e+05
4e+05
6e+05
8e+05
1e+06
Genome position (bp)

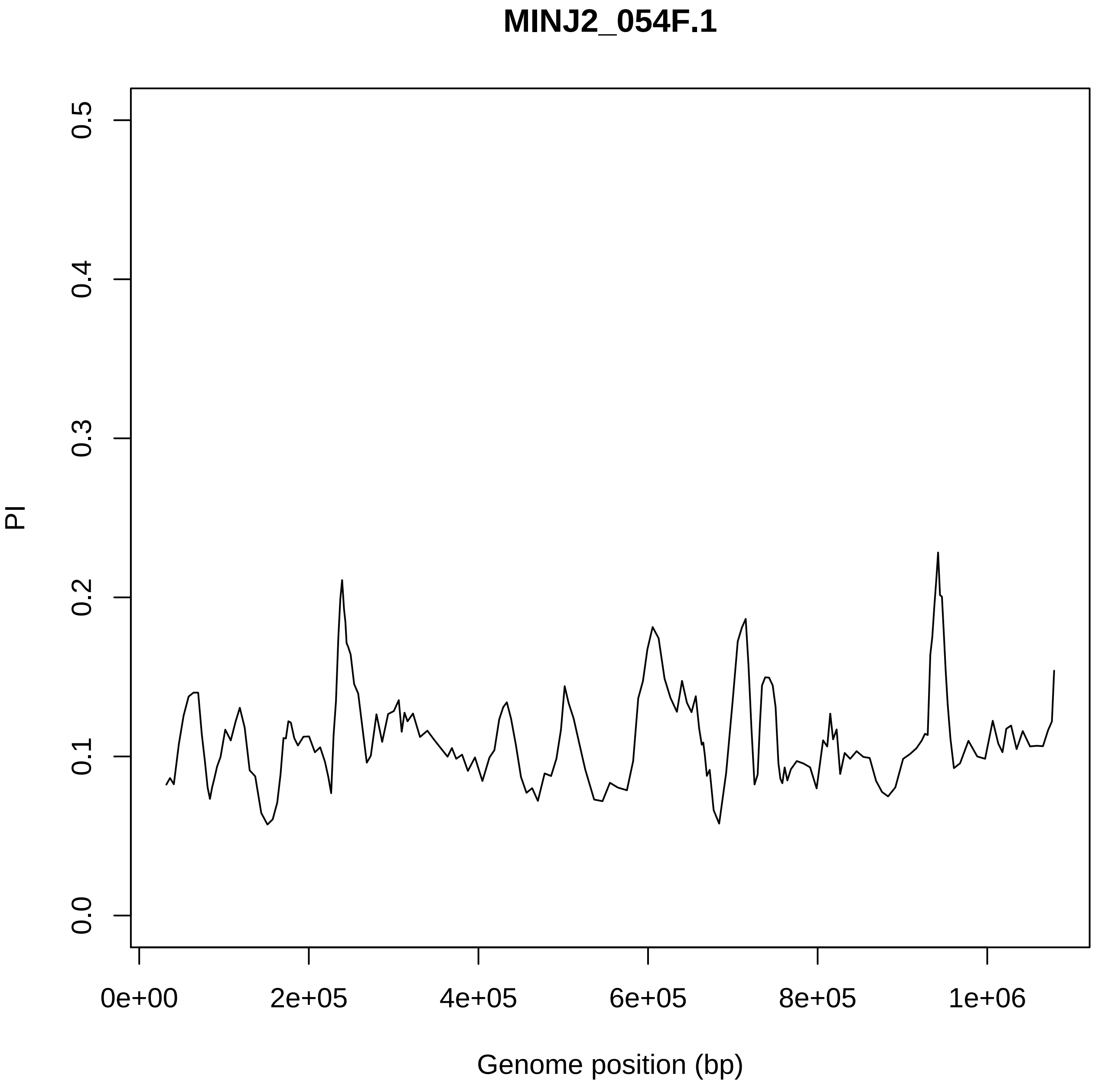

MINJ2_054F.1
PI
0.0
0.1
0.2
0.3
0.4
0.5
0e+00
2e+05
4e+05
6e+05
8e+05
1e+06
Genome position (bp)

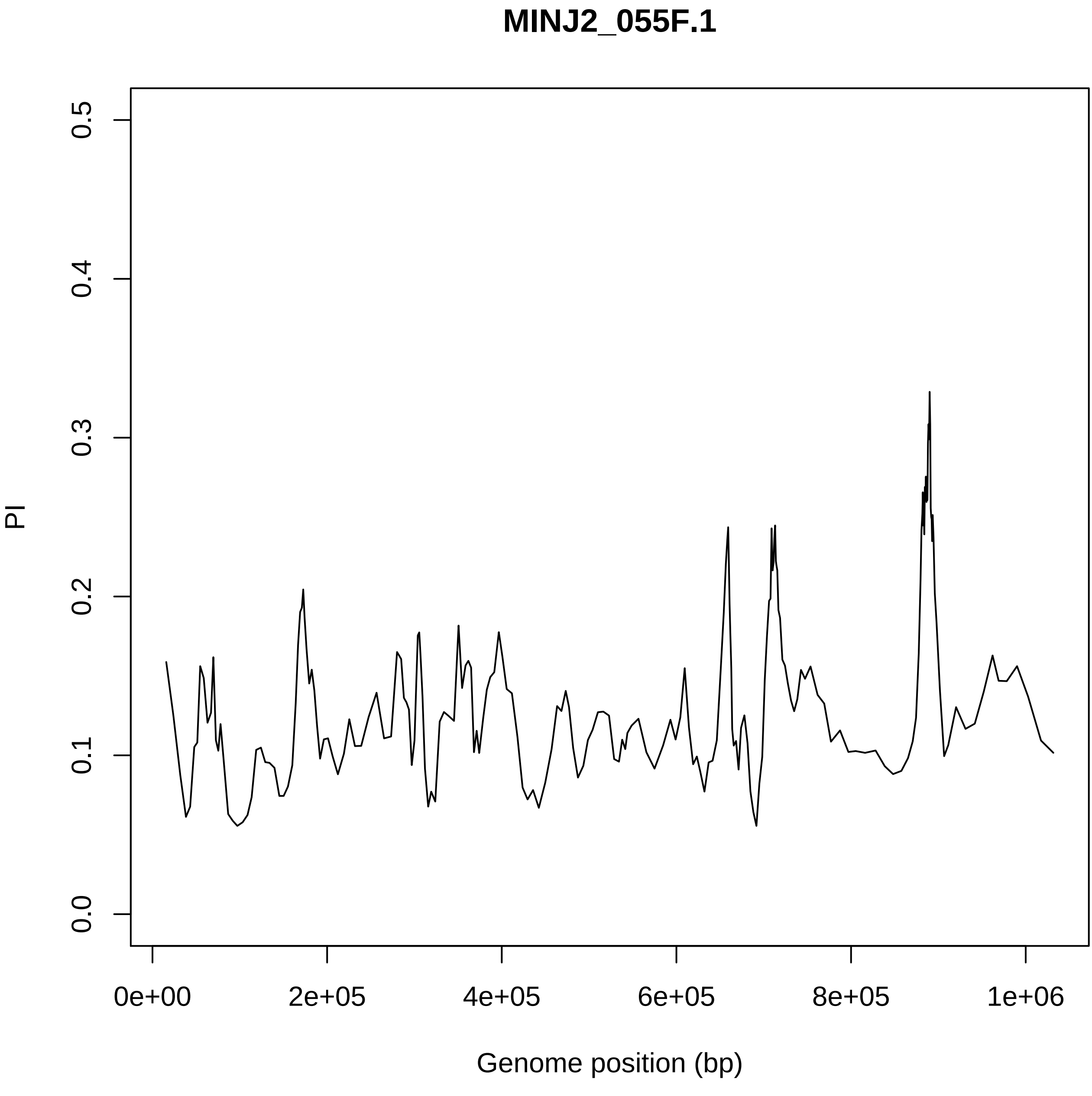

MINJ2_055F.1
PI
0.0
0.1
0.2
0.3
0.4
0.5
0e+00
2e+05
4e+05
6e+05
8e+05
1e+06
Genome position (bp)

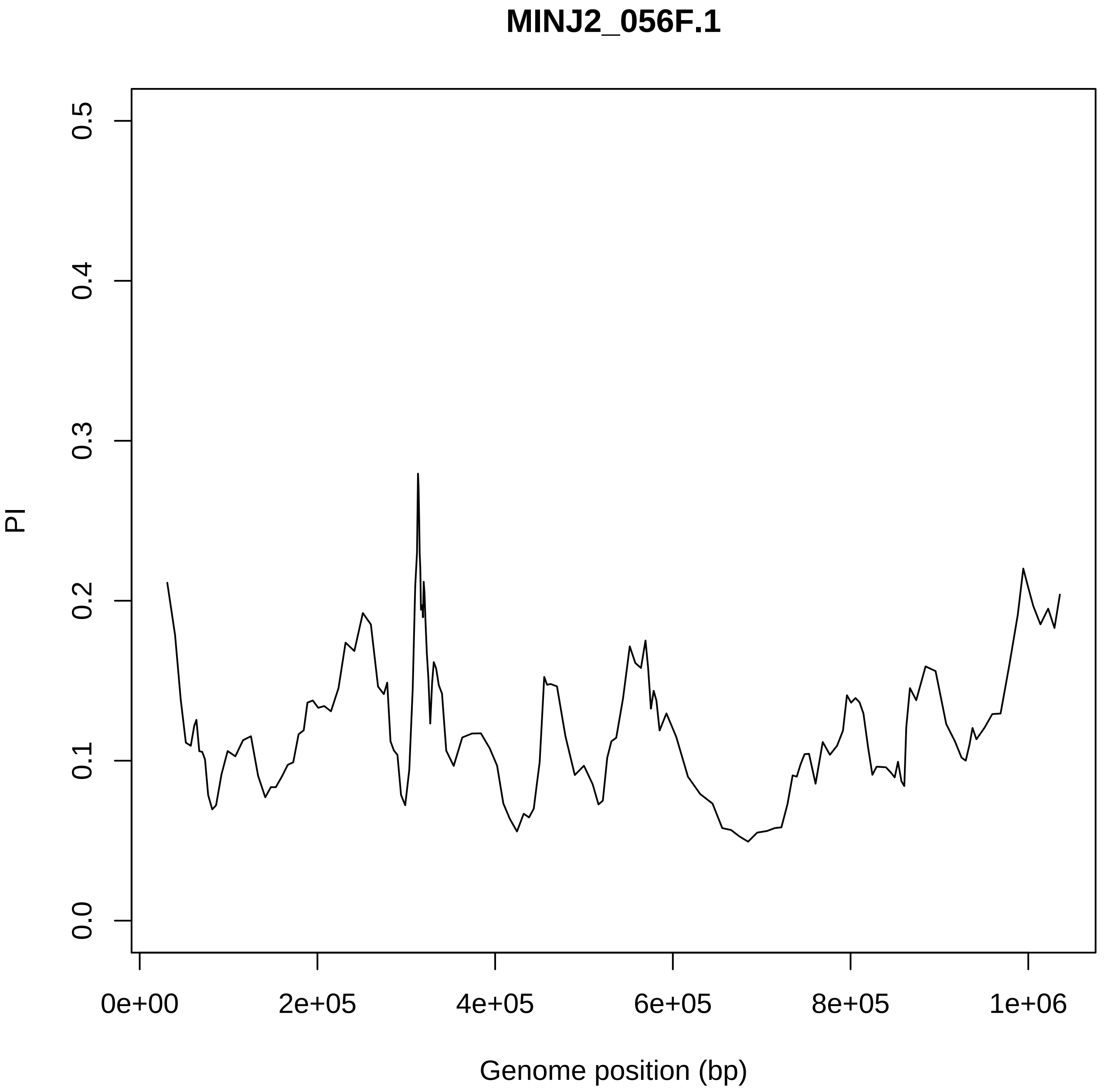
MINJ2_056F.1
PI
0.0
0.1
0.2
0.3
0.4
0.5
0e+00
2e+05
4e+05
6e+05
8e+05
1e+06
Genome position (bp)

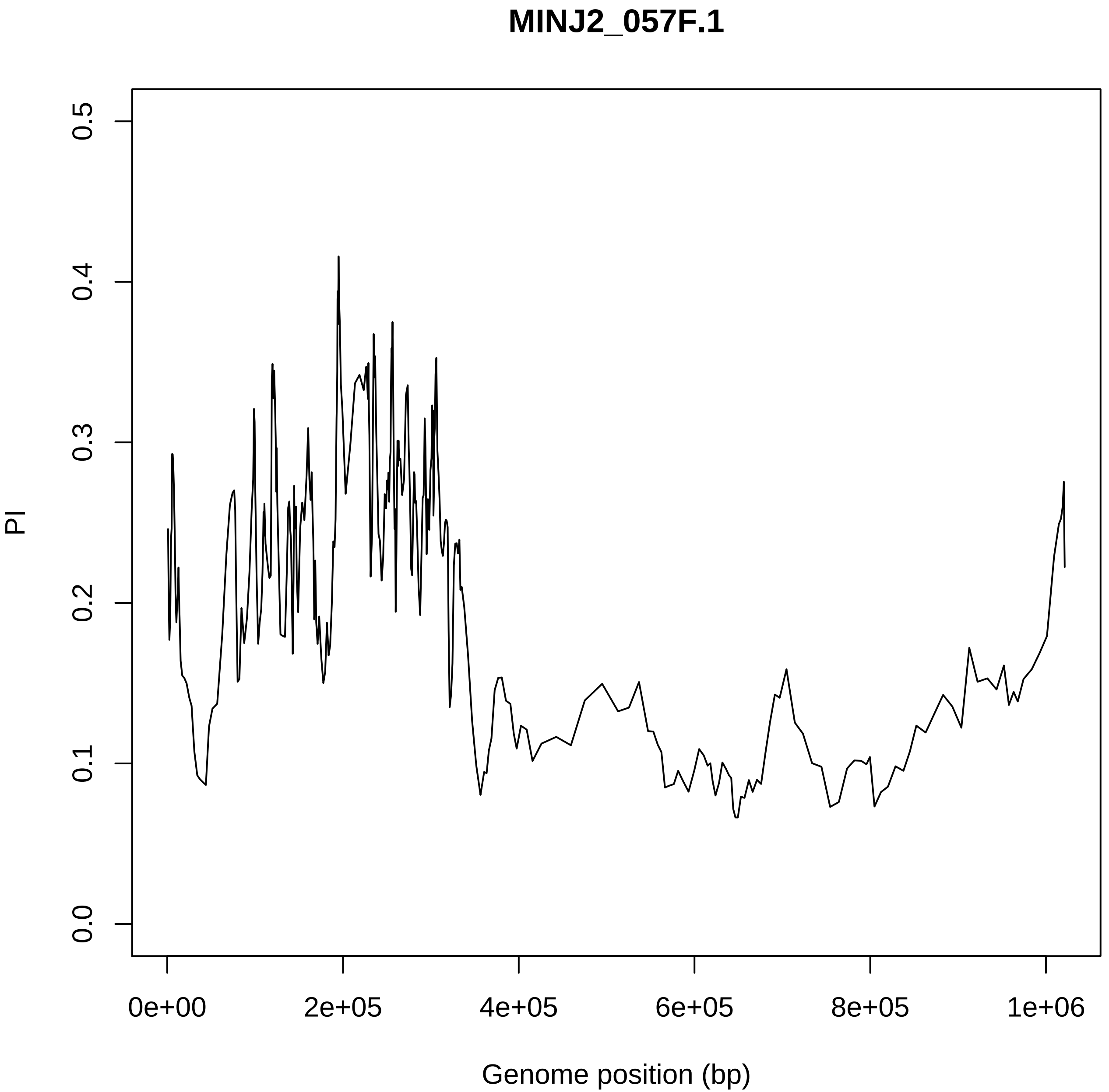
MINJ2_057F.1
PI
0.0
0.1
0.2
0.3
0.4
0.5
0e+00
2e+05
4e+05
6e+05
8e+05
1e+06
Genome position (bp)

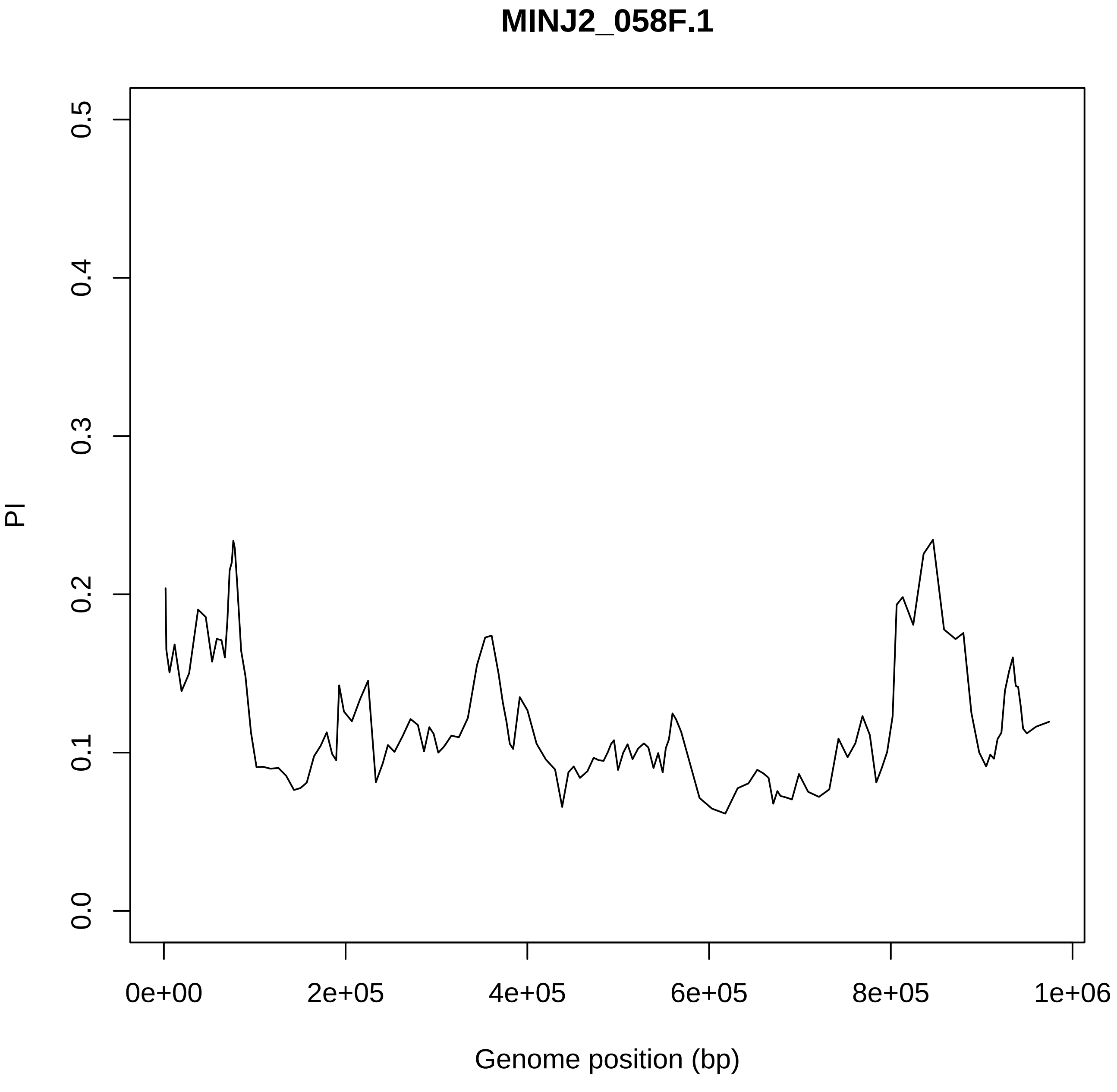

MINJ2_058F.1
PI
0.0
0.1
0.2
0.3
0.4
0.5
0e+00
2e+05
4e+05
6e+05
8e+05
1e+06
Genome position (bp)

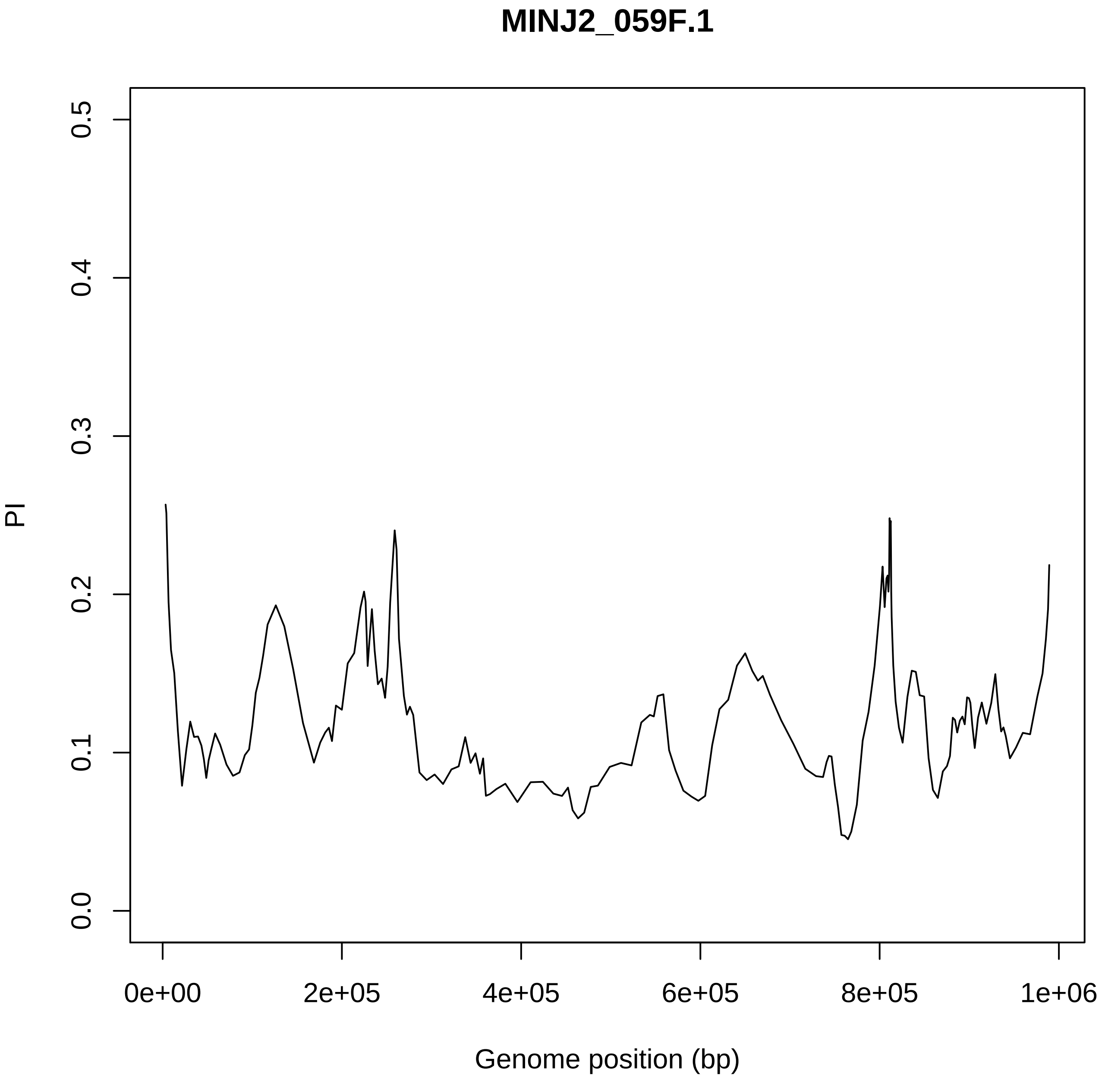
MINJ2_059F.1
PI
0.0
0.1
0.2
0.3
0.4
0.5
0e+00
2e+05
4e+05
6e+05
8e+05
1e+06
Genome position (bp)

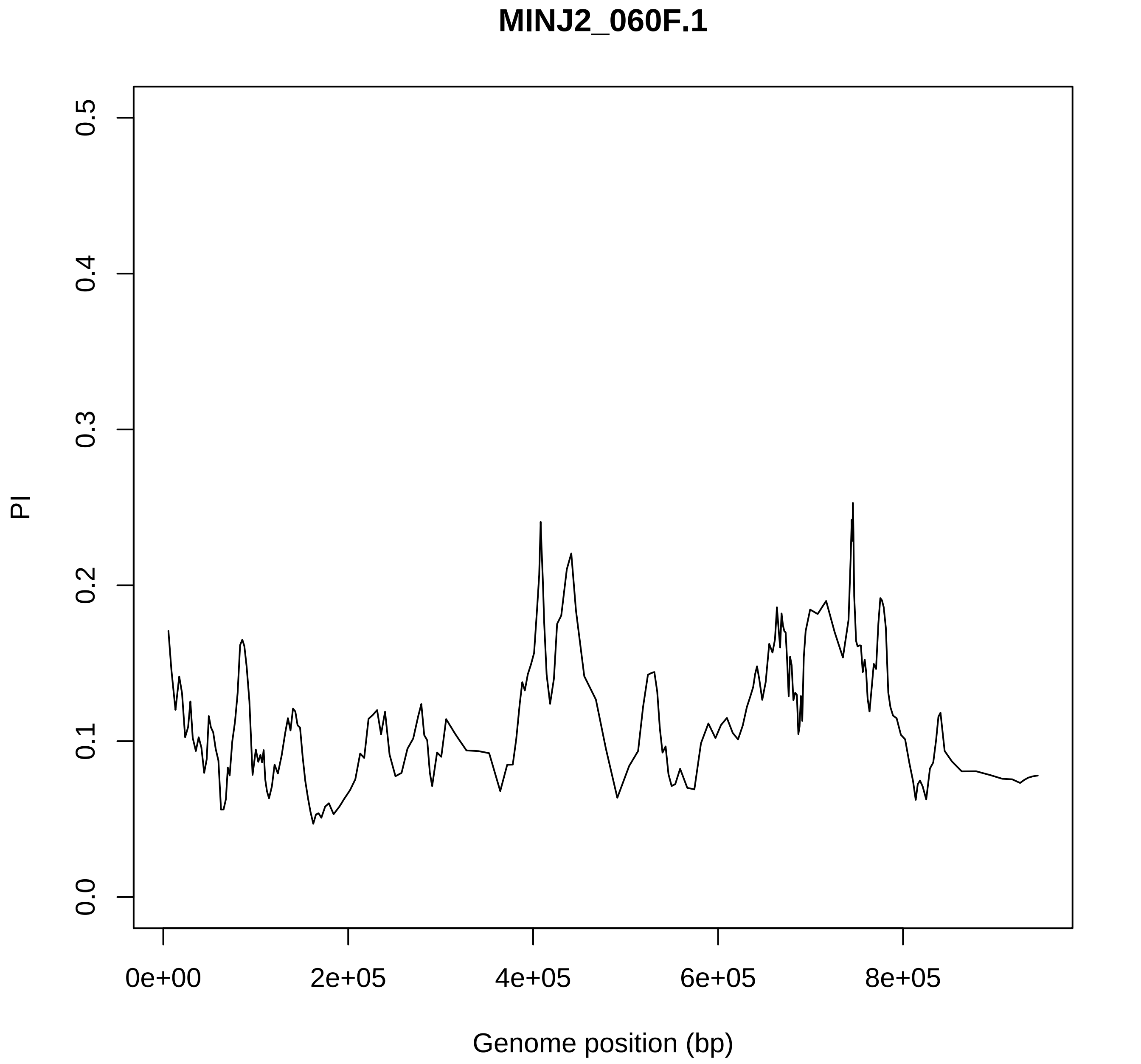
MINJ2_060F.1
PI
0.0
0.1
0.2
0.3
0.4
0.5
0e+00
2e+05
4e+05
6e+05
8e+05
Genome position (bp)

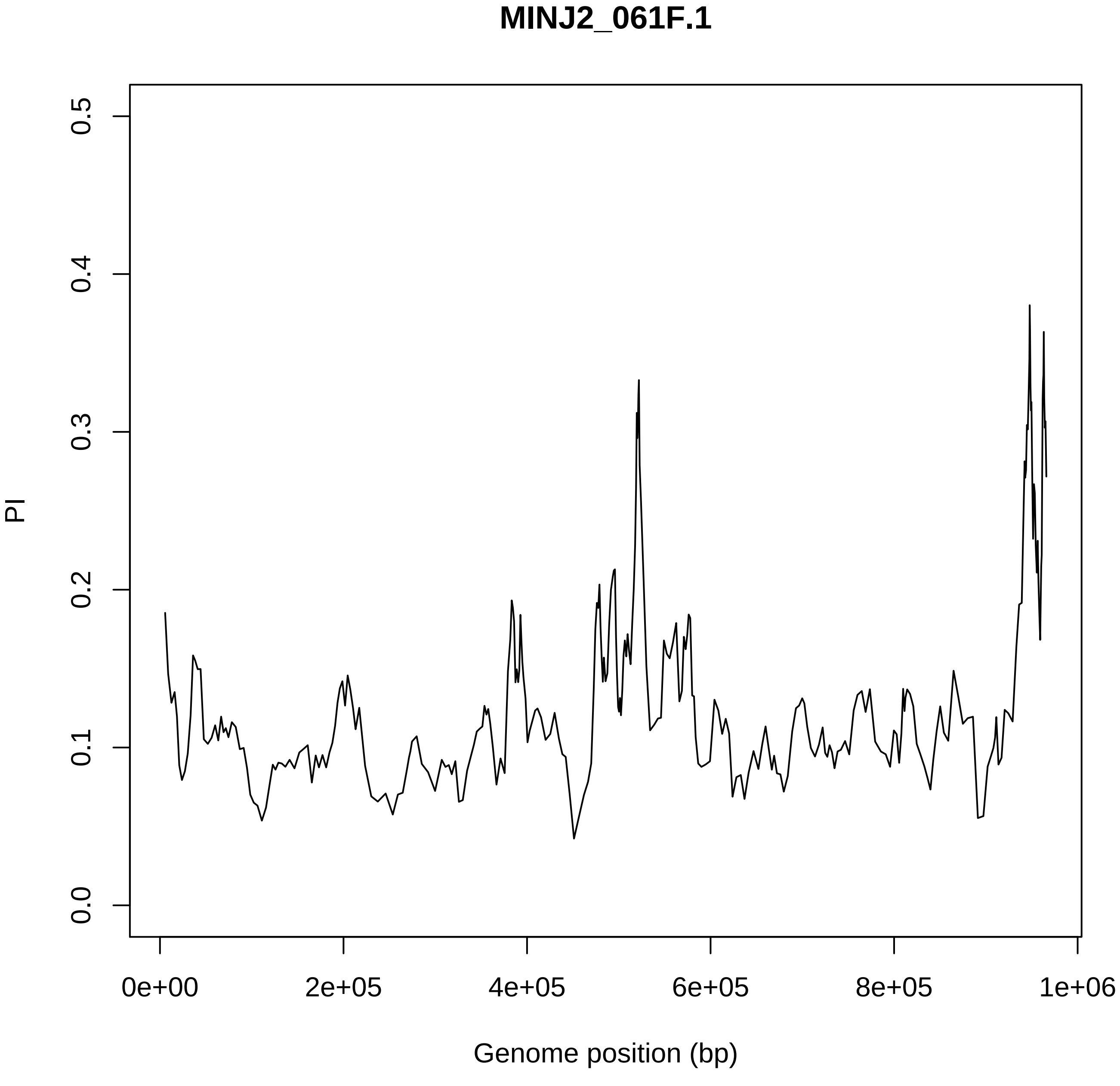

MINJ2_061F.1
PI
0.0
0.1
0.2
0.3
0.4
0.5
0e+00
2e+05
4e+05
6e+05
8e+05
1e+06
Genome position (bp)

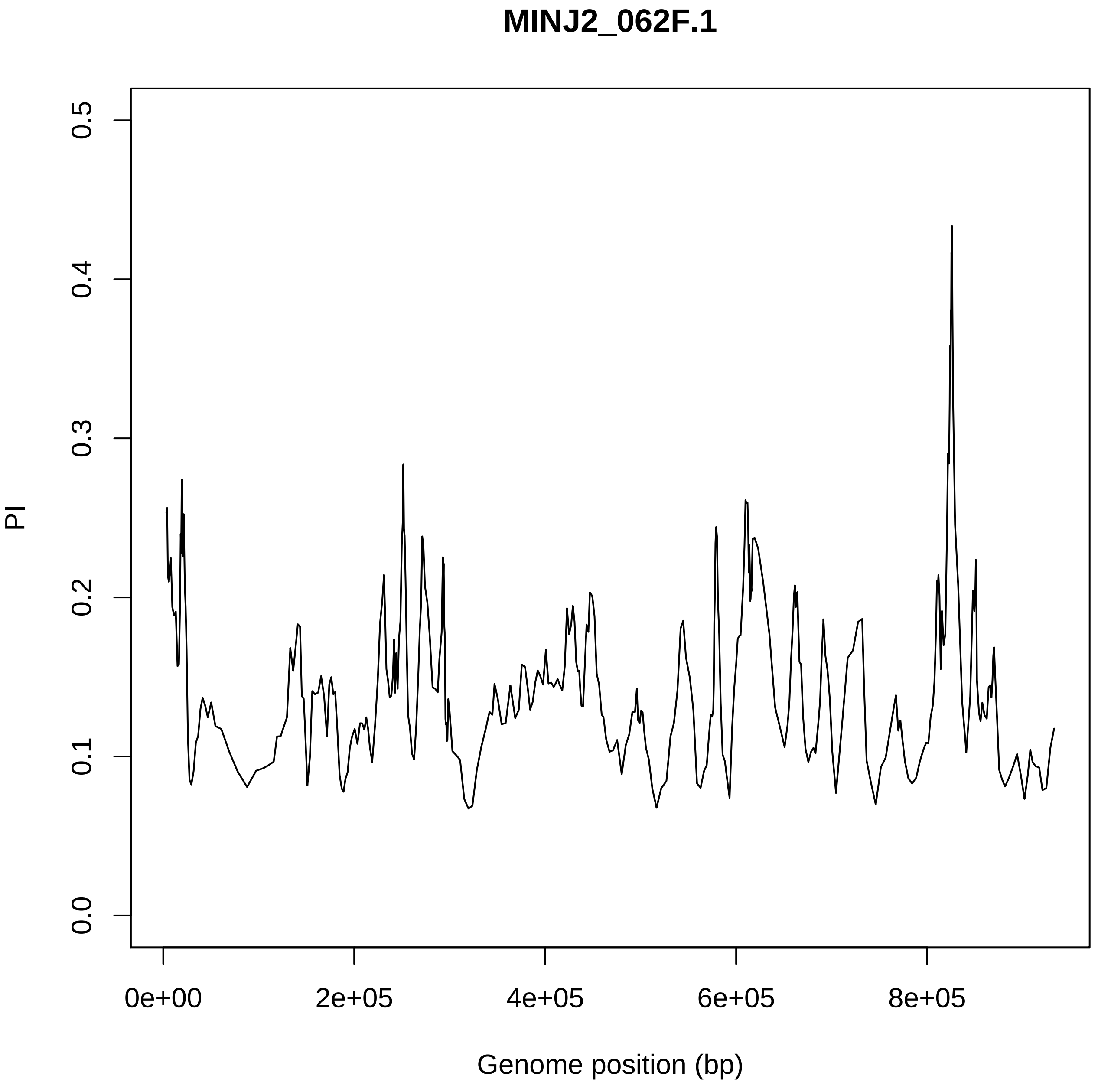

MINJ2_062F.1
PI
0.0
0.1
0.2
0.3
0.4
0.5
0e+00
2e+05
4e+05
6e+05
8e+05
Genome position (bp)

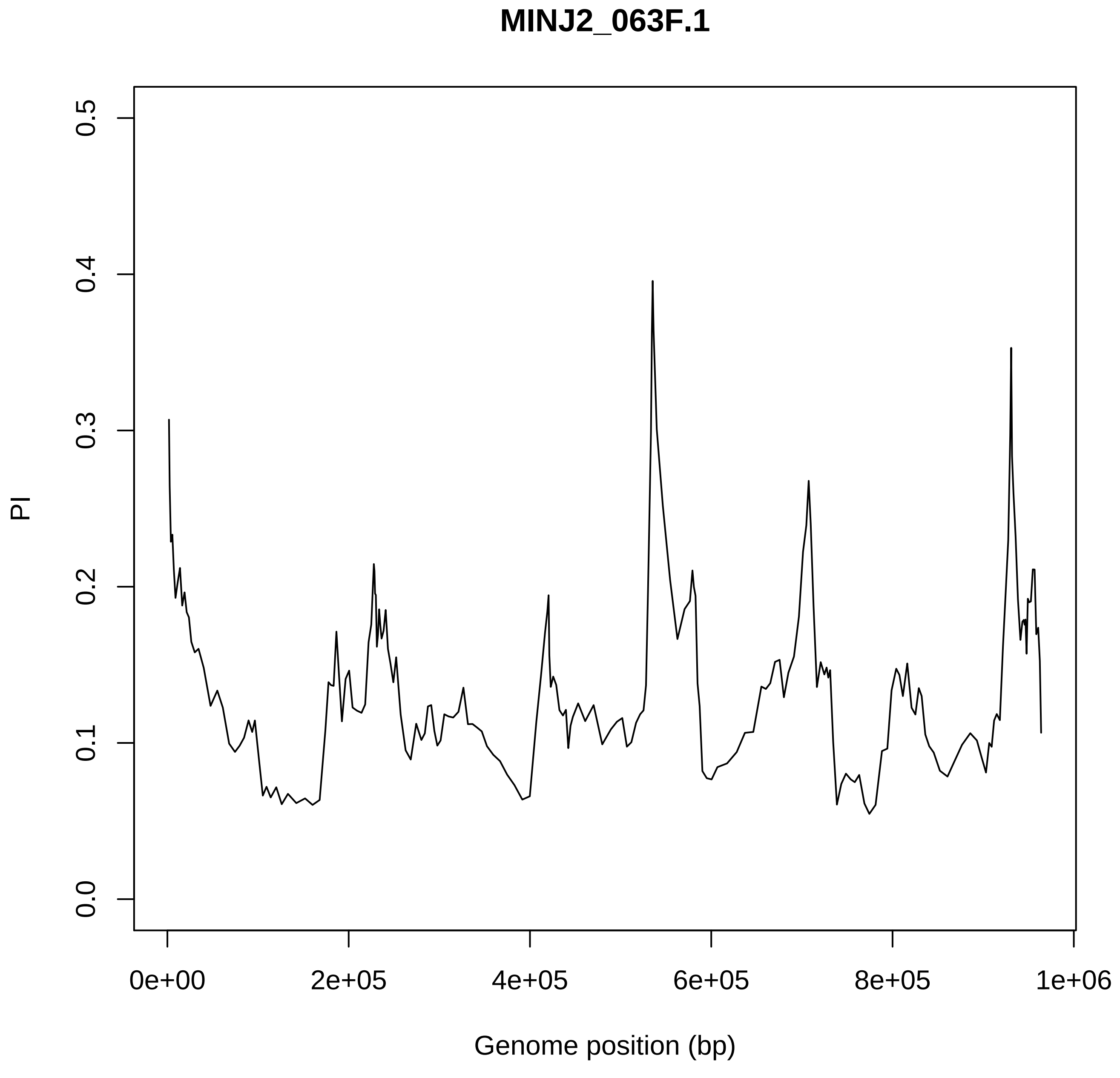

MINJ2_063F.1
PI
0.0
0.1
0.2
0.3
0.4
0.5
0e+00
2e+05
4e+05
6e+05
8e+05
1e+06
Genome position (bp)

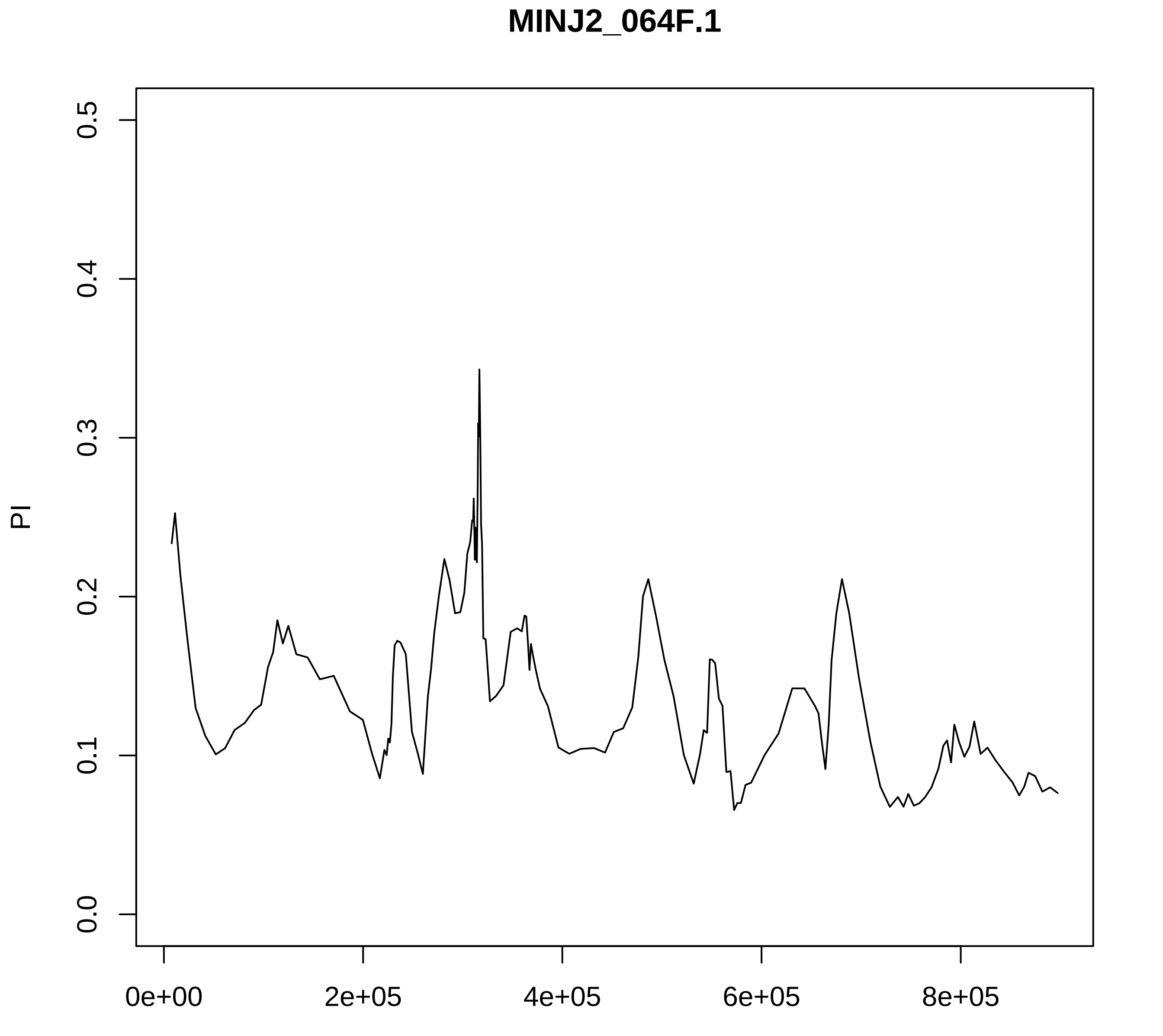
MINJ2_064F.1
PI
0.0
0.1
0.2
0.3
0.4
0.5
0e+00
2e+05
4e+05
6e+05
8e+05
Genome position (bp)

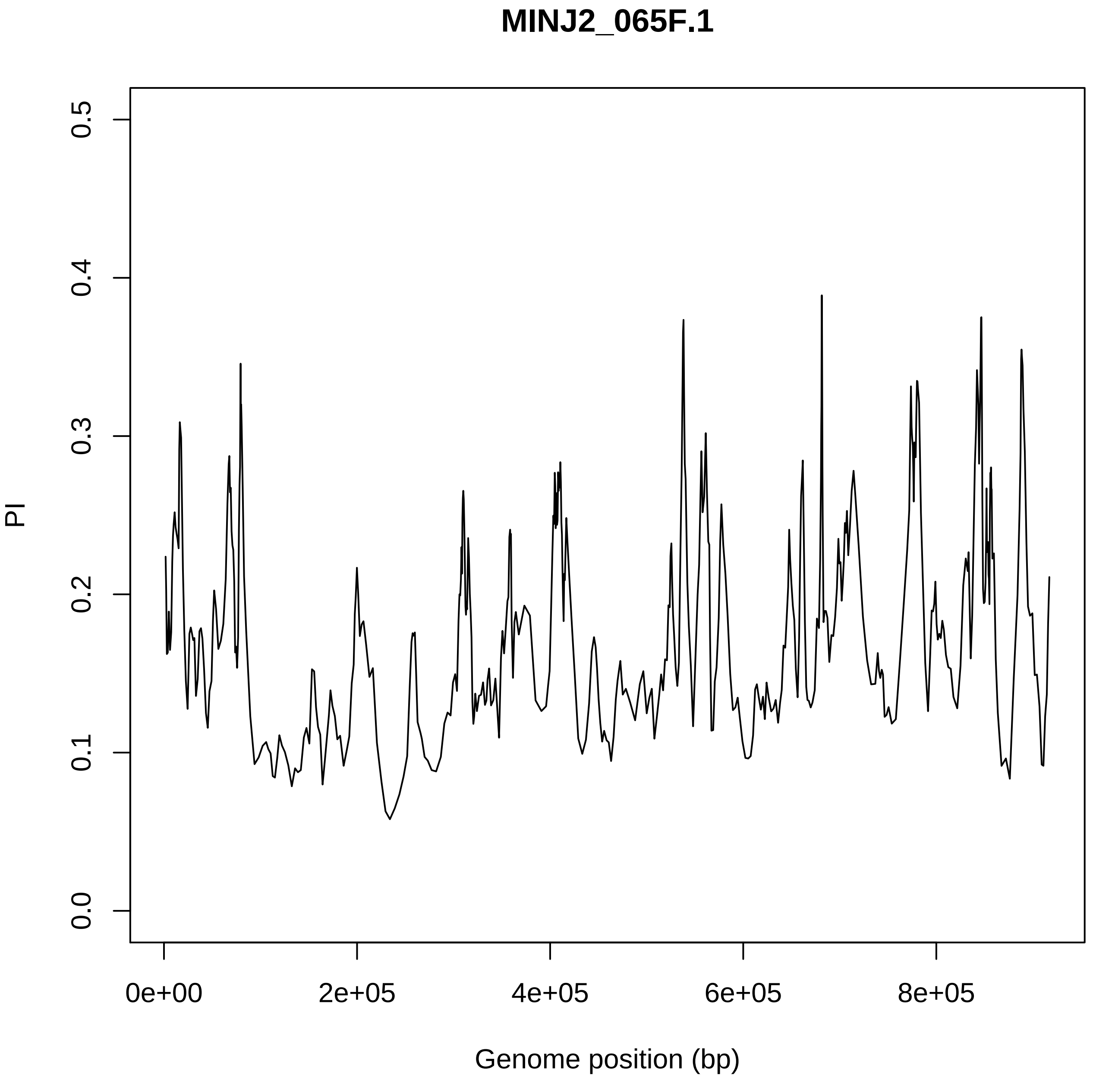
MINJ2_065F.1
PI
0.0
0.1
0.2
0.3
0.4
0.5
0e+00
2e+05
4e+05
6e+05
8e+05
Genome position (bp)

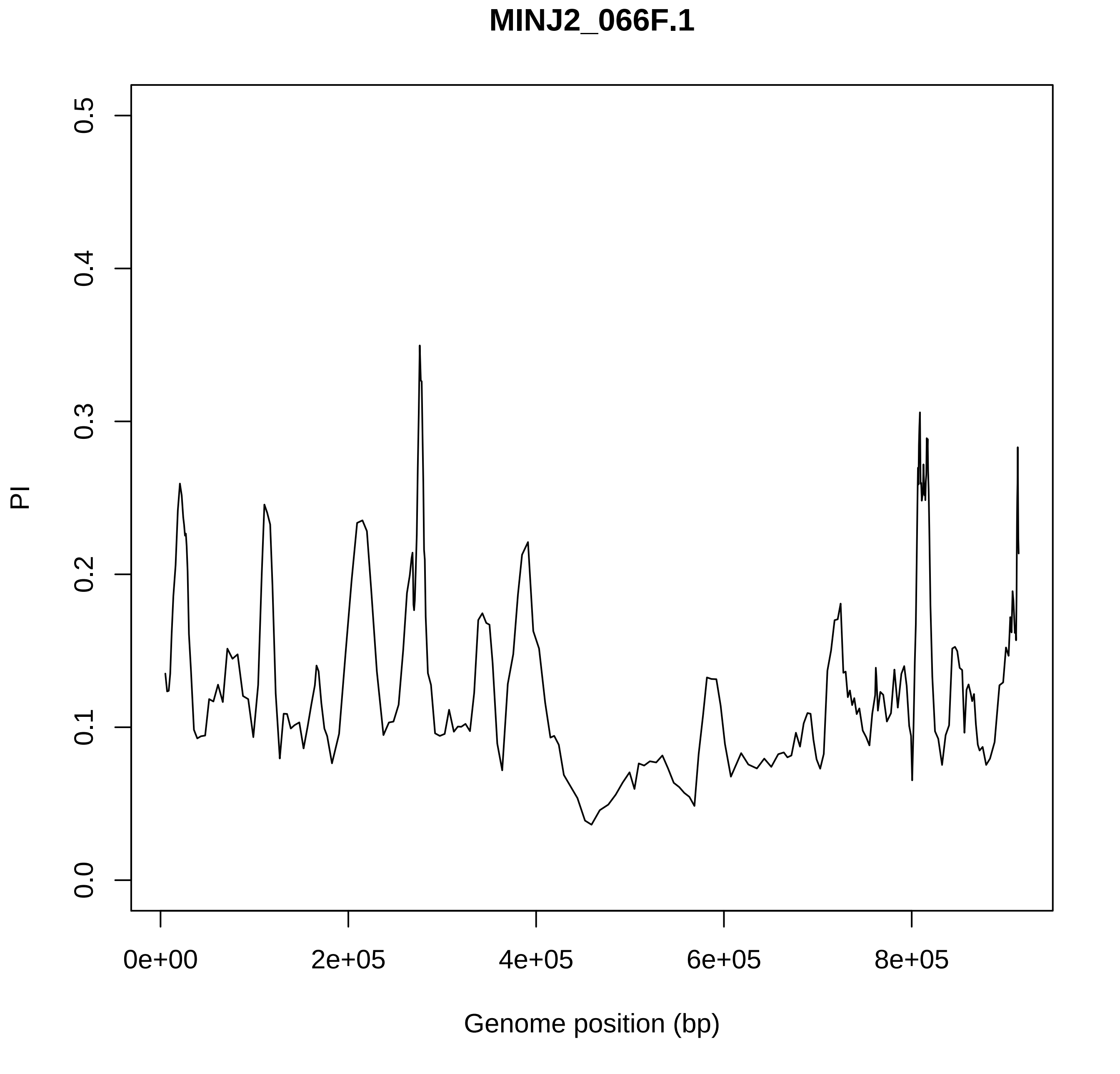
MINJ2_066F.1
PI
0.0
0.1
0.2
0.3
0.4
0.5
0e+00
2e+05
4e+05
6e+05
8e+05
Genome position (bp)

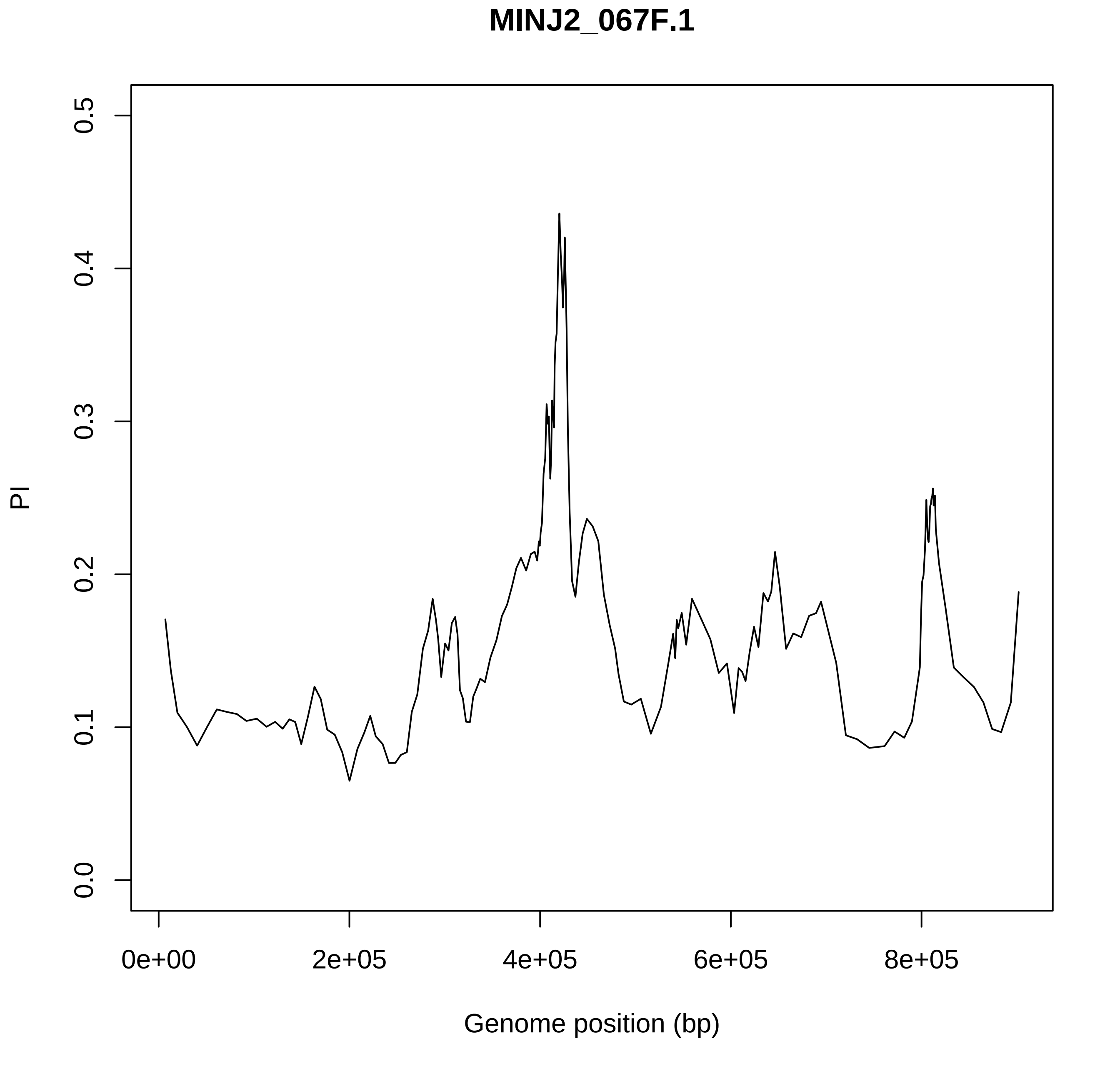
MINJ2_067F.1
PI
0.0
0.1
0.2
0.3
0.4
0.5
0e+00
2e+05
4e+05
6e+05
8e+05
Genome position (bp)

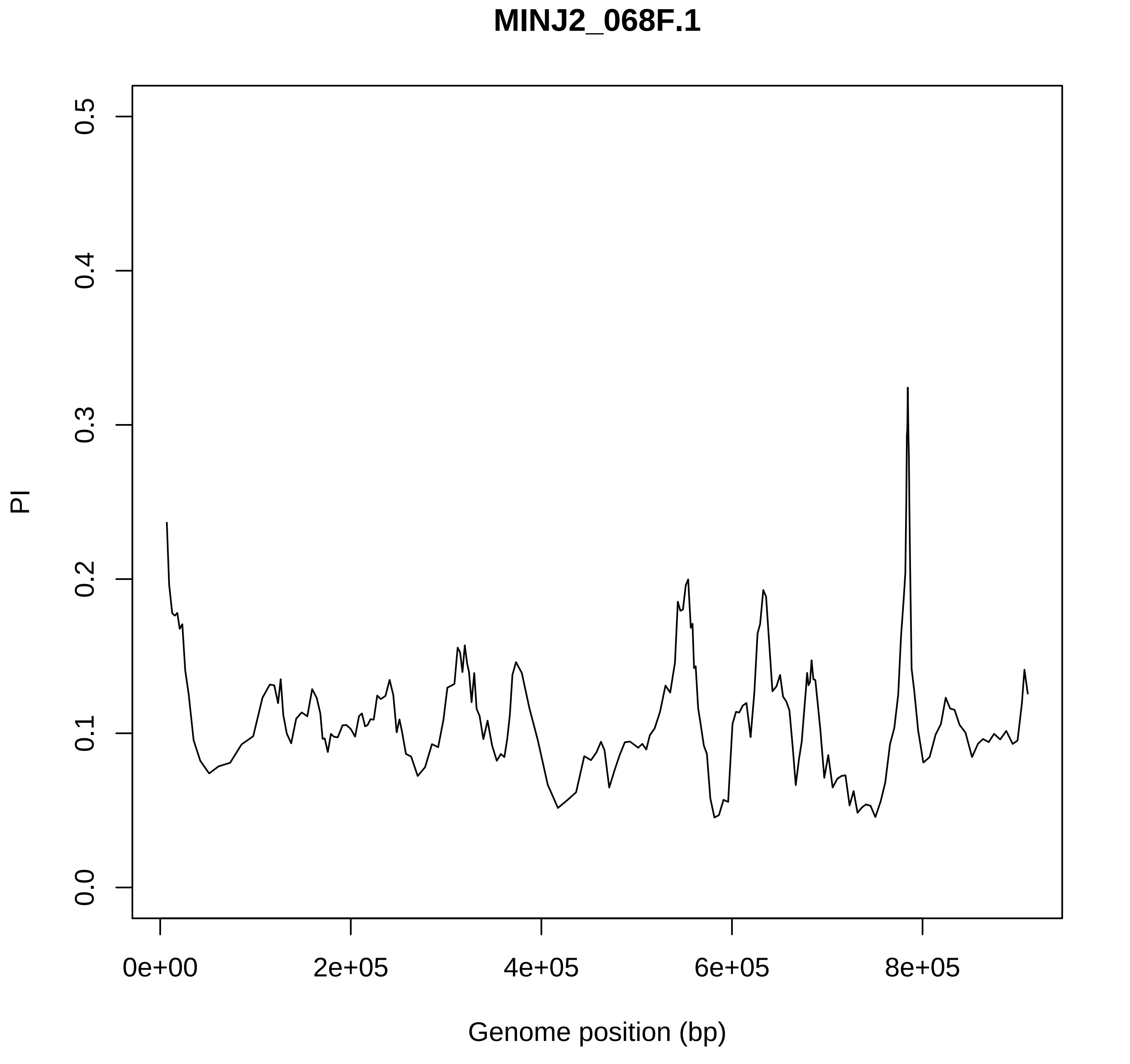
MINJ2_068F.1
PI
0.0
0.1
0.2
0.3
0.4
0.5
0e+00
2e+05
4e+05
6e+05
8e+05
Genome position (bp)

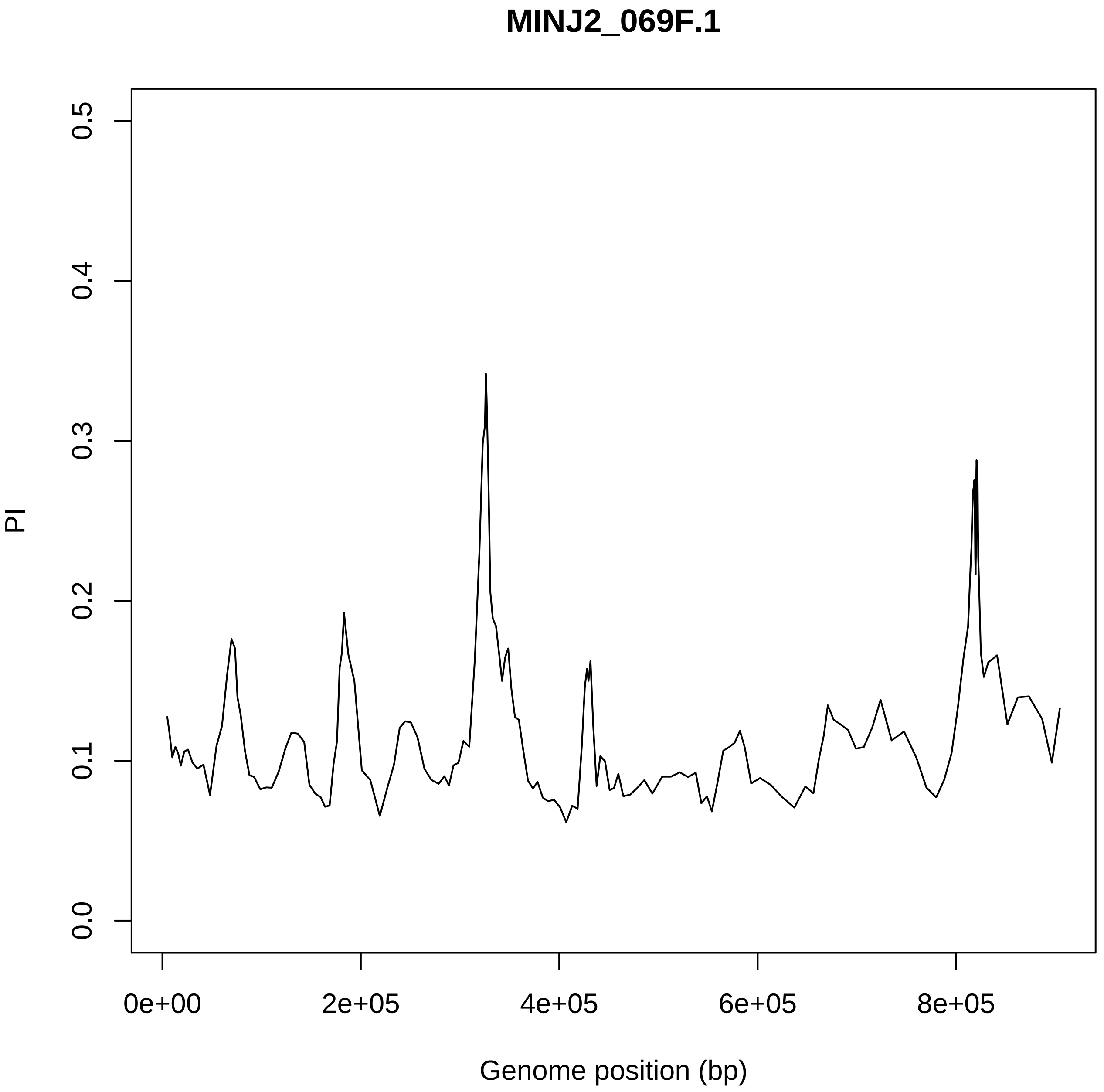
MINJ2_069F.1
PI
0.0
0.1
0.2
0.3
0.4
0.5
0e+00
2e+05
4e+05
6e+05
8e+05
Genome position (bp)

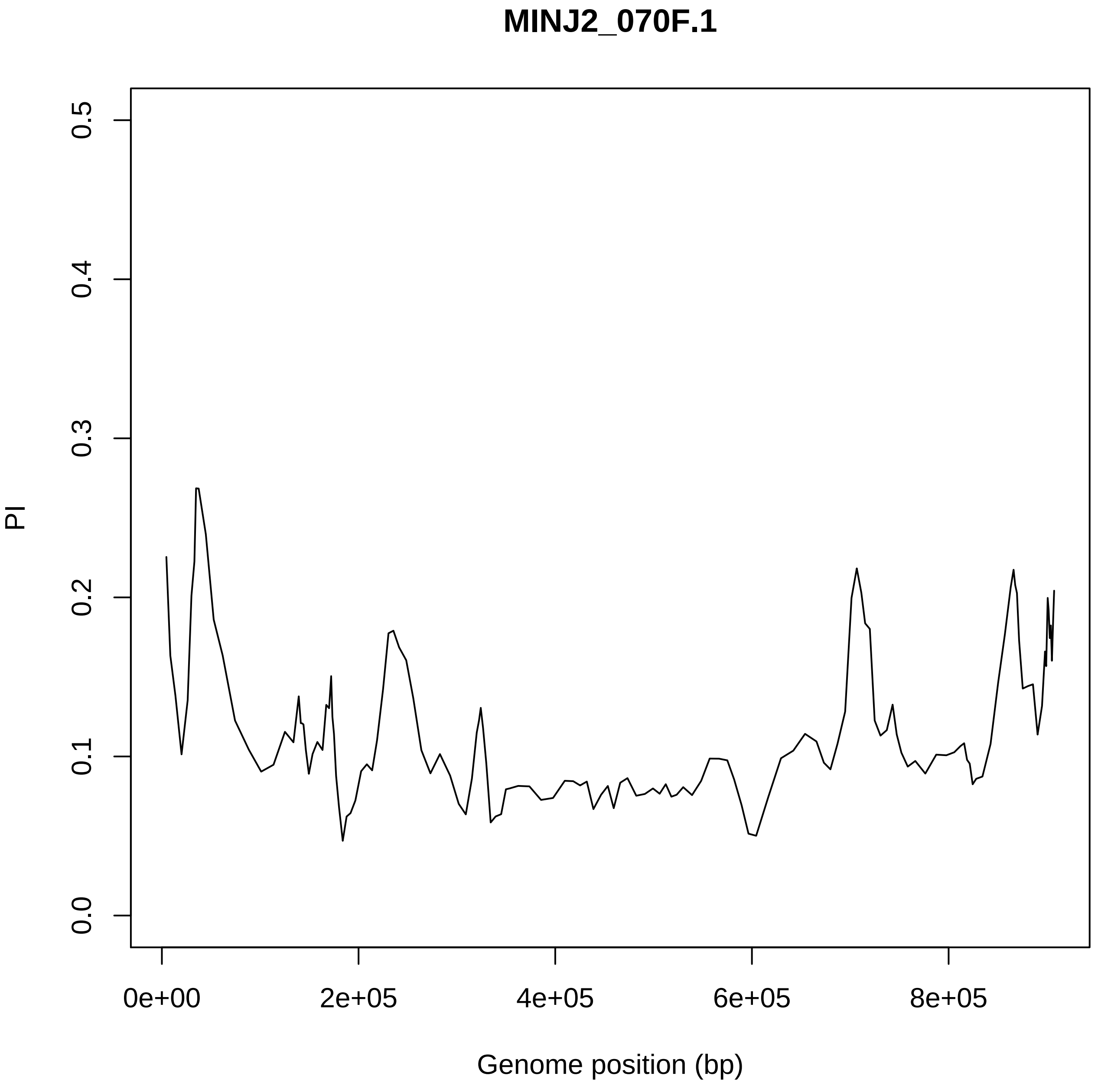
MINJ2_070F.1
PI
0.0
0.1
0.2
0.3
0.4
0.5
0e+00
2e+05
4e+05
6e+05
8e+05
Genome position (bp)

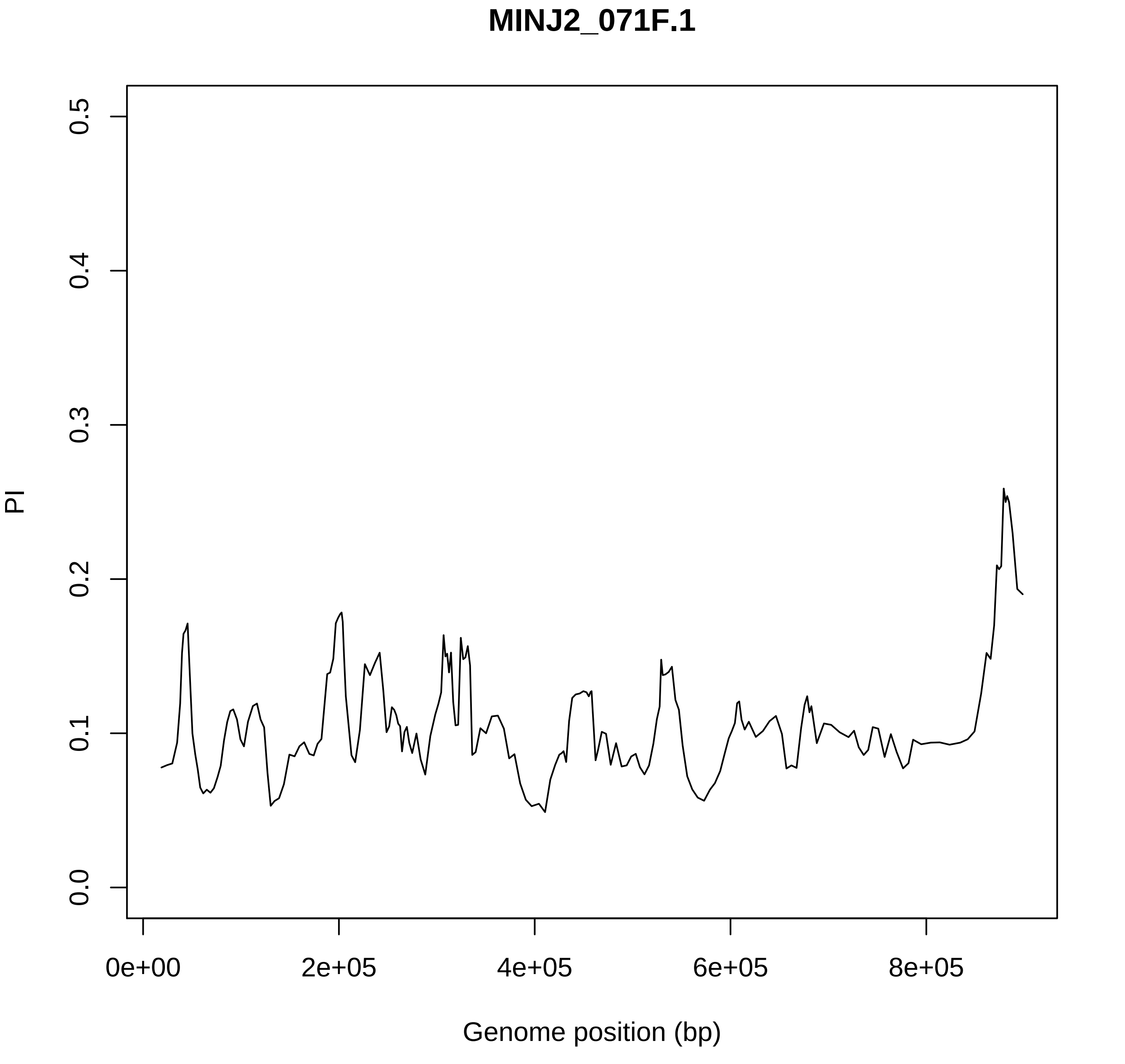

MINJ2_071F.1
PI
0.0
0.1
0.2
0.3
0.4
0.5
0e+00
2e+05
4e+05
6e+05
8e+05
Genome position (bp)

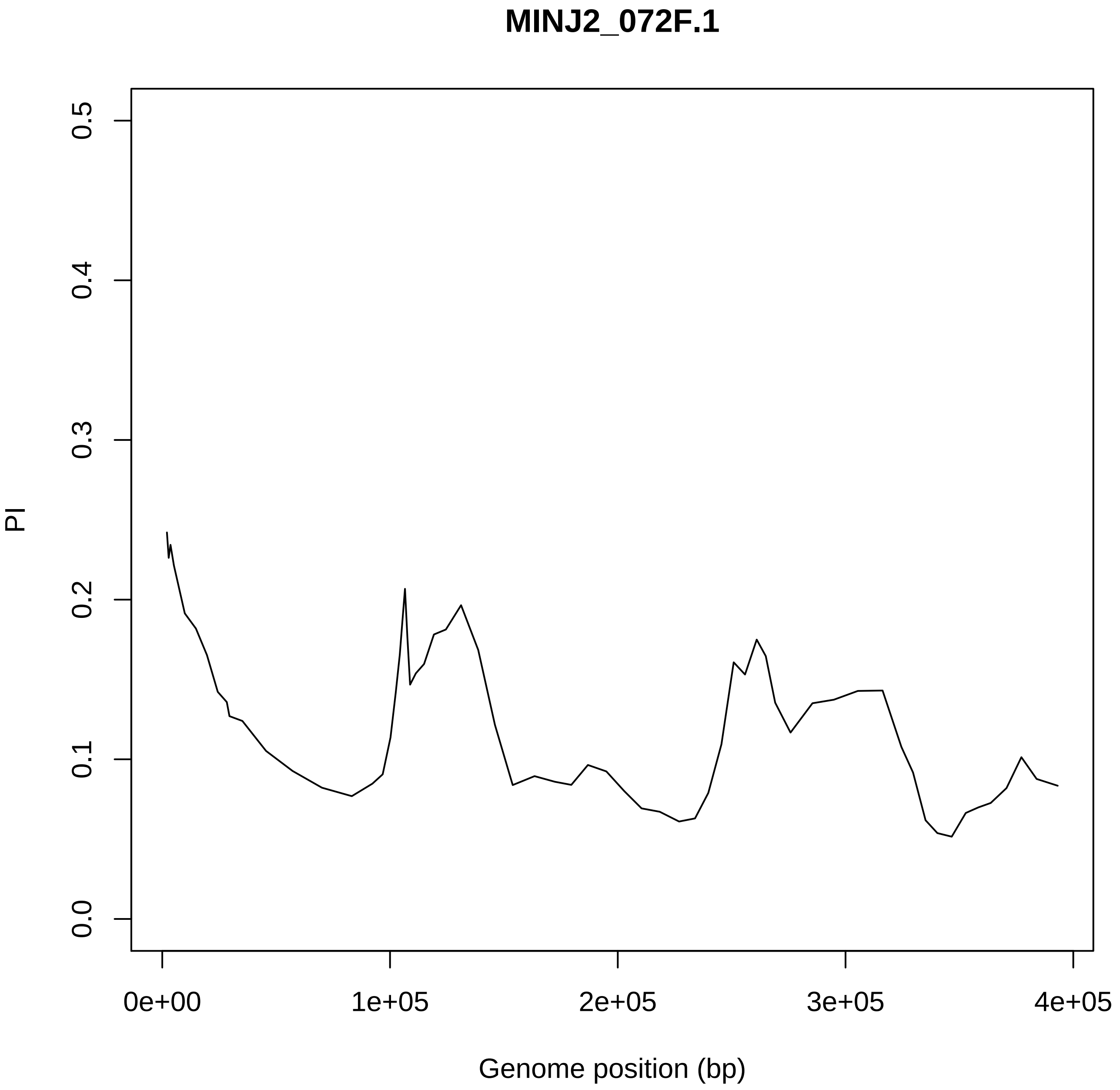
MINJ2_072F.1
PI
0.0
0.1
0.2
0.3
0.4
0.5
0e+00
1e+05
2e+05
3e+05
4e+05
Genome position (bp)

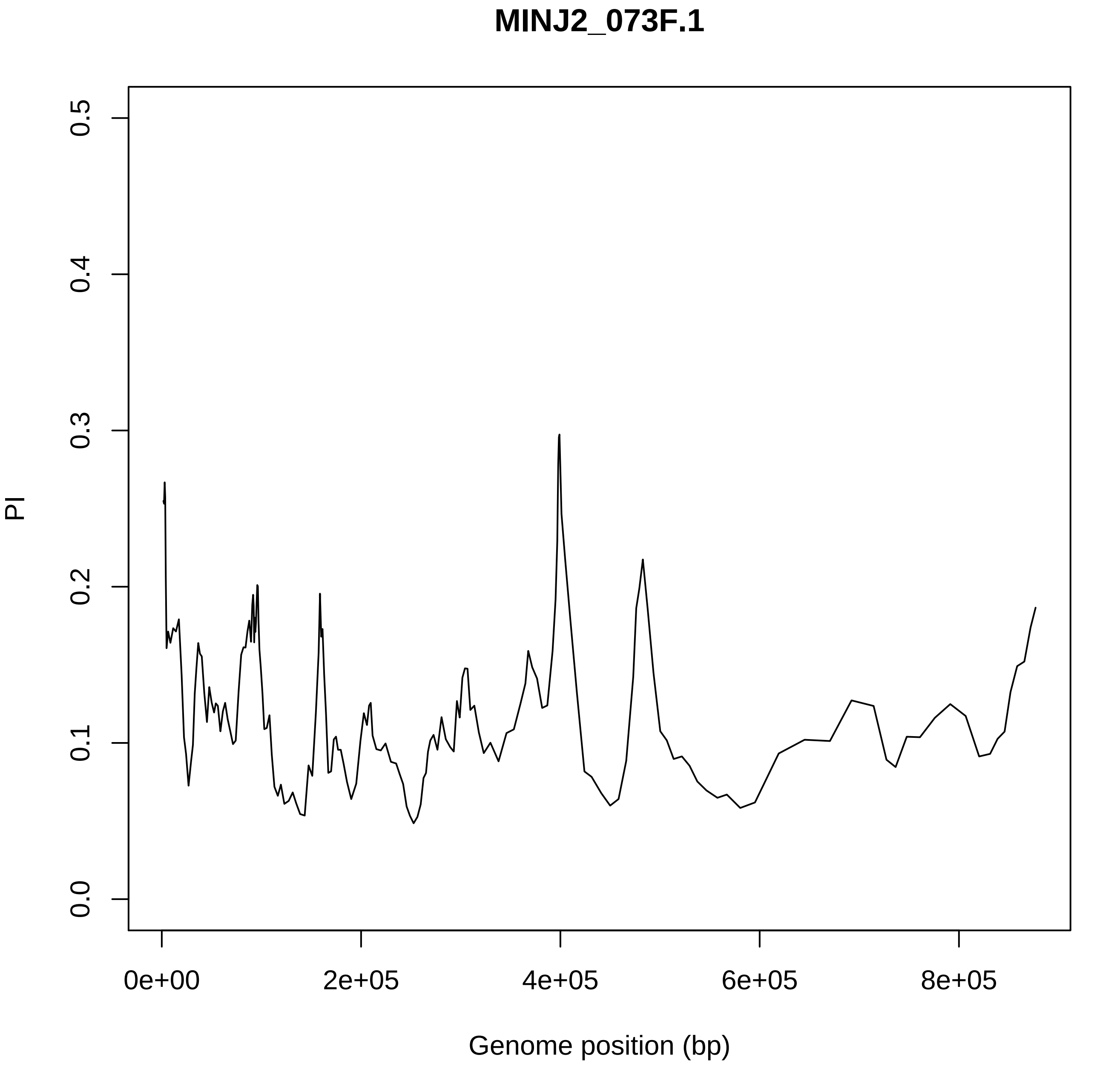

MINJ2_073F.1
PI
0.0
0.1
0.2
0.3
0.4
0.5
0e+00
2e+05
4e+05
6e+05
8e+05
Genome position (bp)

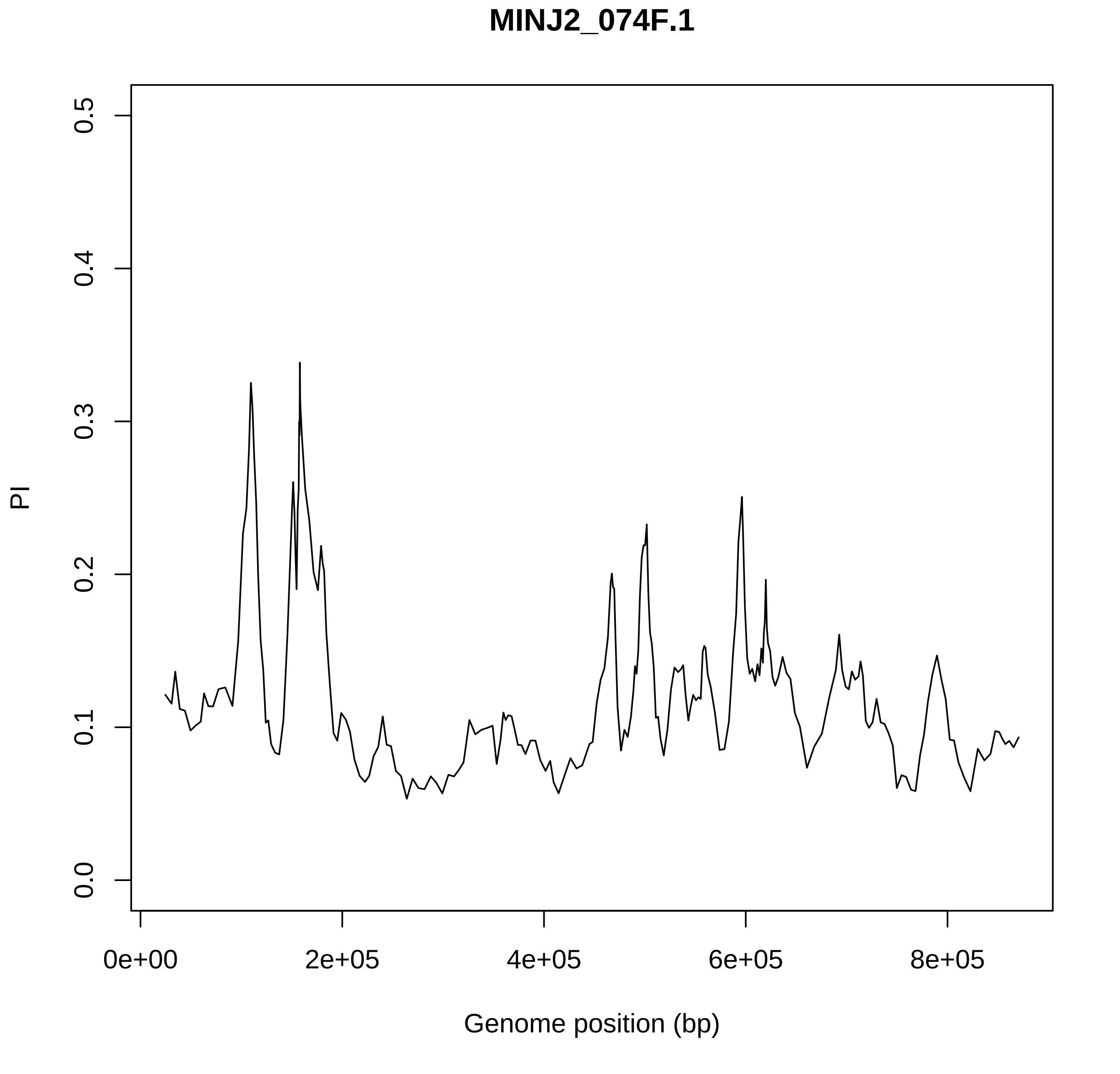
MINJ2_074F.1
PI
0.0
0.1
0.2
0.3
0.4
0.5
0e+00
2e+05
4e+05
6e+05
8e+05
Genome position (bp)

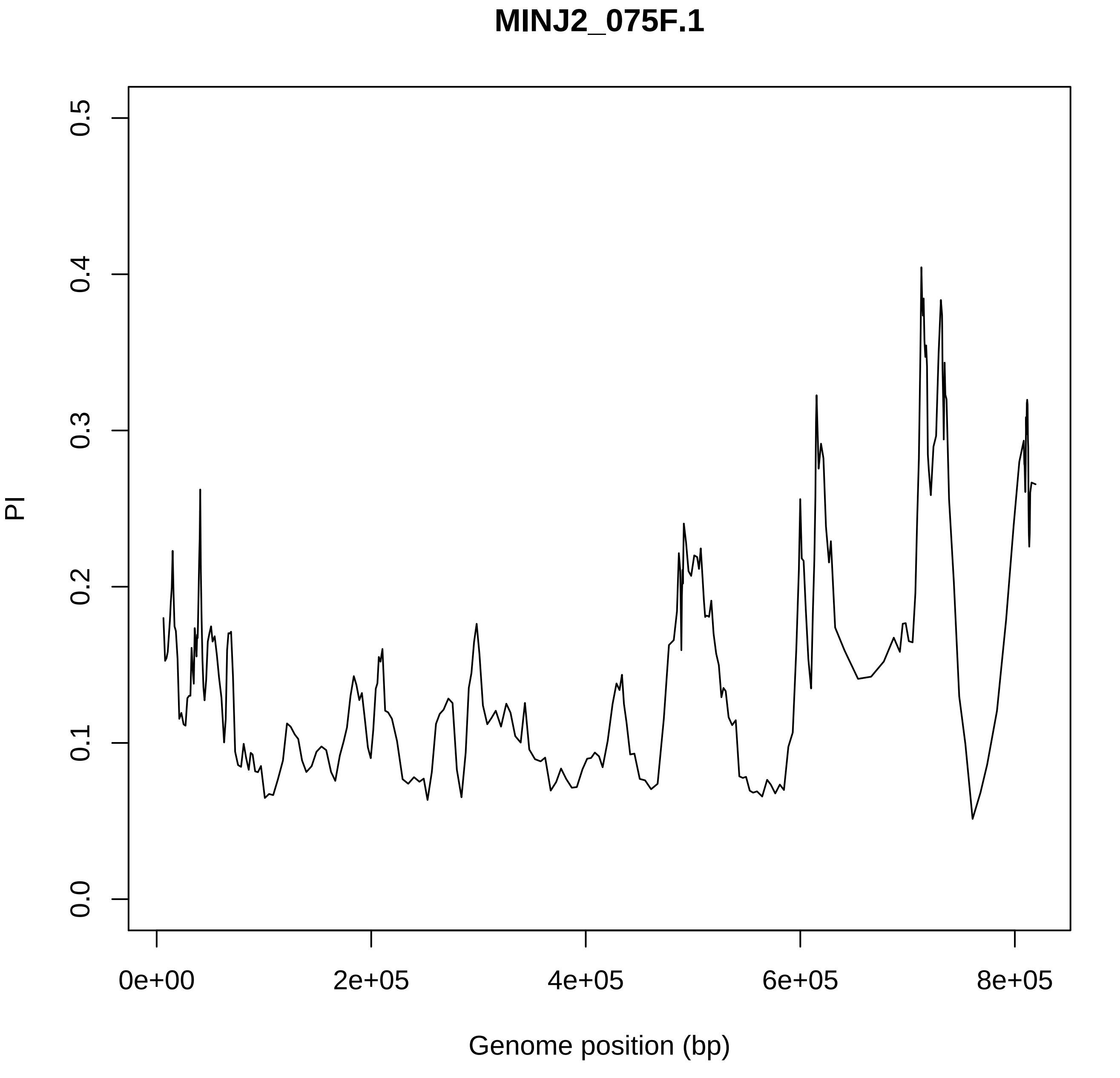
MINJ2_075F.1
PI
0.0
0.1
0.2
0.3
0.4
0.5
0e+00
2e+05
4e+05
6e+05
8e+05
Genome position (bp)

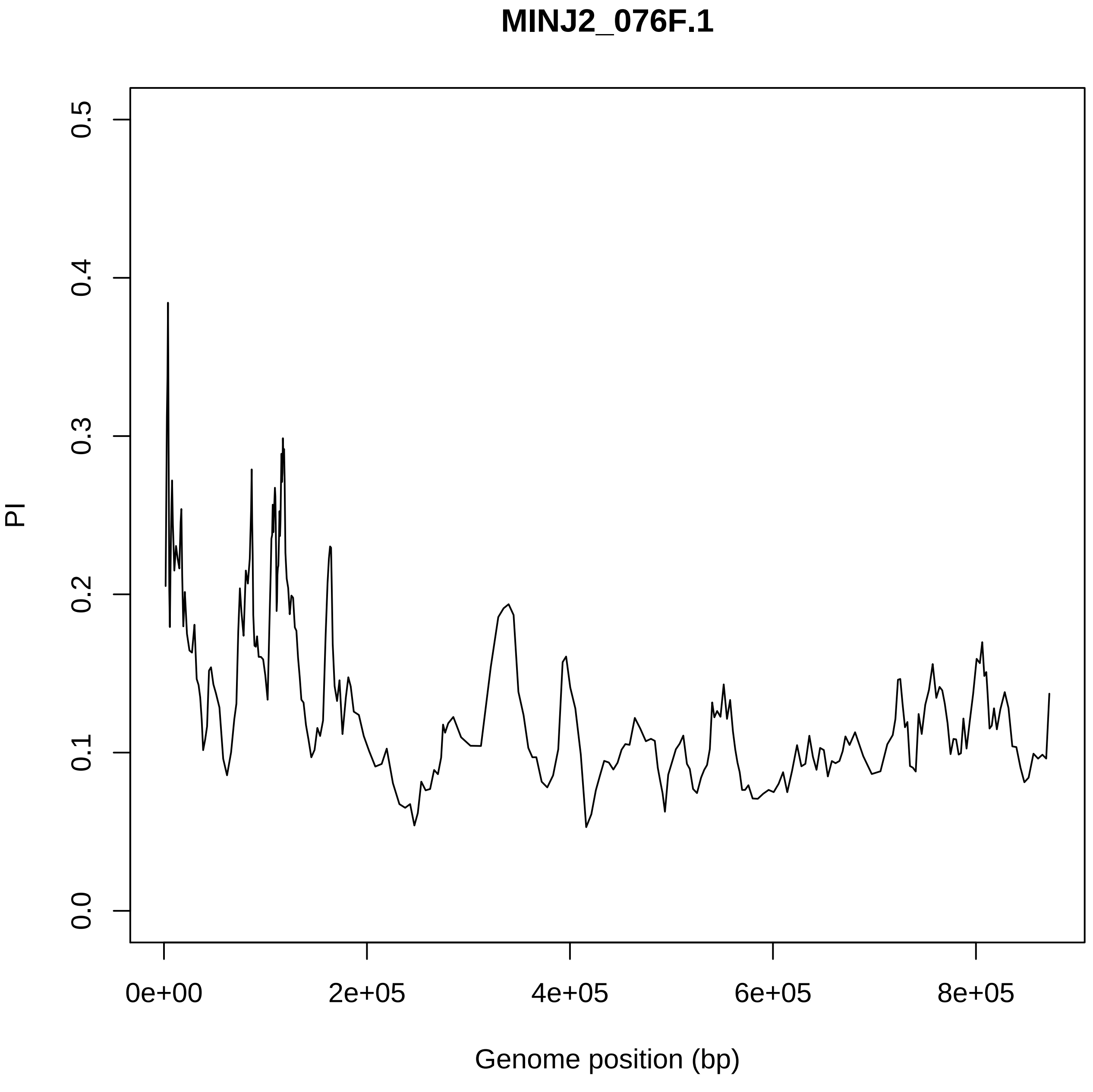
MINJ2_076F.1
PI
0.0
0.1
0.2
0.3
0.4
0.5
0e+00
2e+05
4e+05
6e+05
8e+05
Genome position (bp)

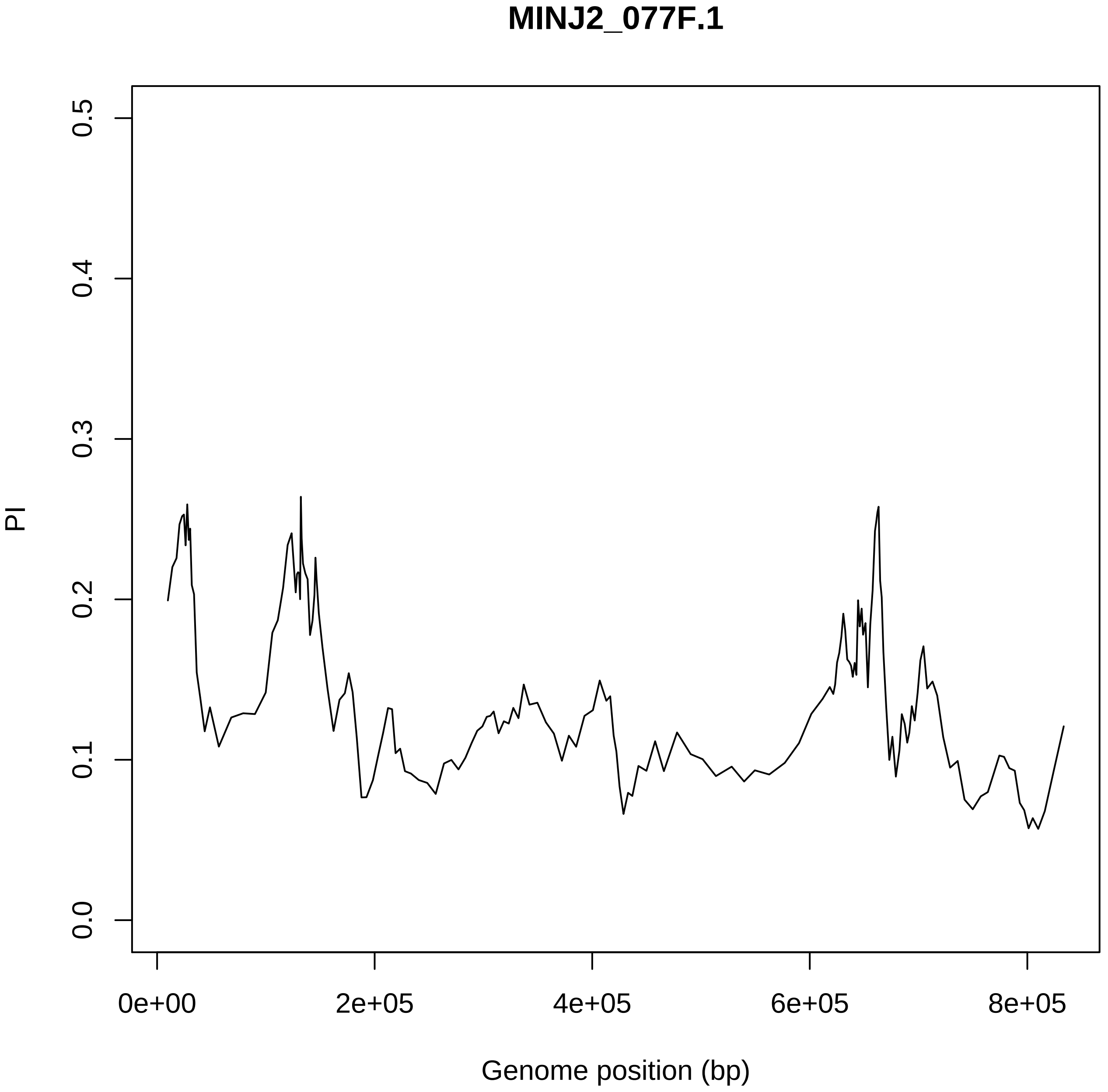
MINJ2_077F.1
PI
0.0
0.1
0.2
0.3
0.4
0.5
0e+00
2e+05
4e+05
6e+05
8e+05
Genome position (bp)

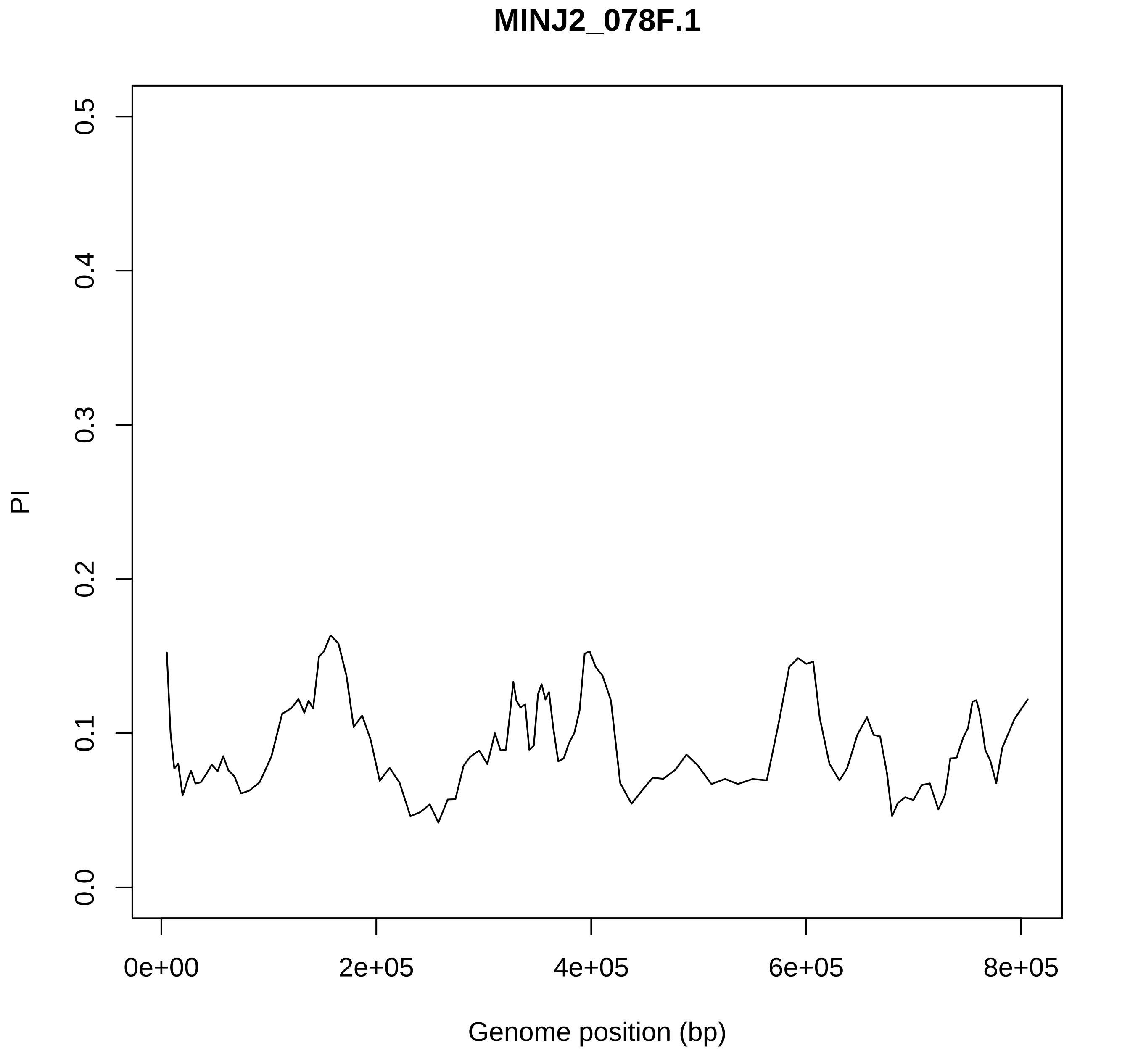

MINJ2_078F.1
PI
0.0
0.1
0.2
0.3
0.4
0.5
0e+00
2e+05
4e+05
6e+05
8e+05
Genome position (bp)

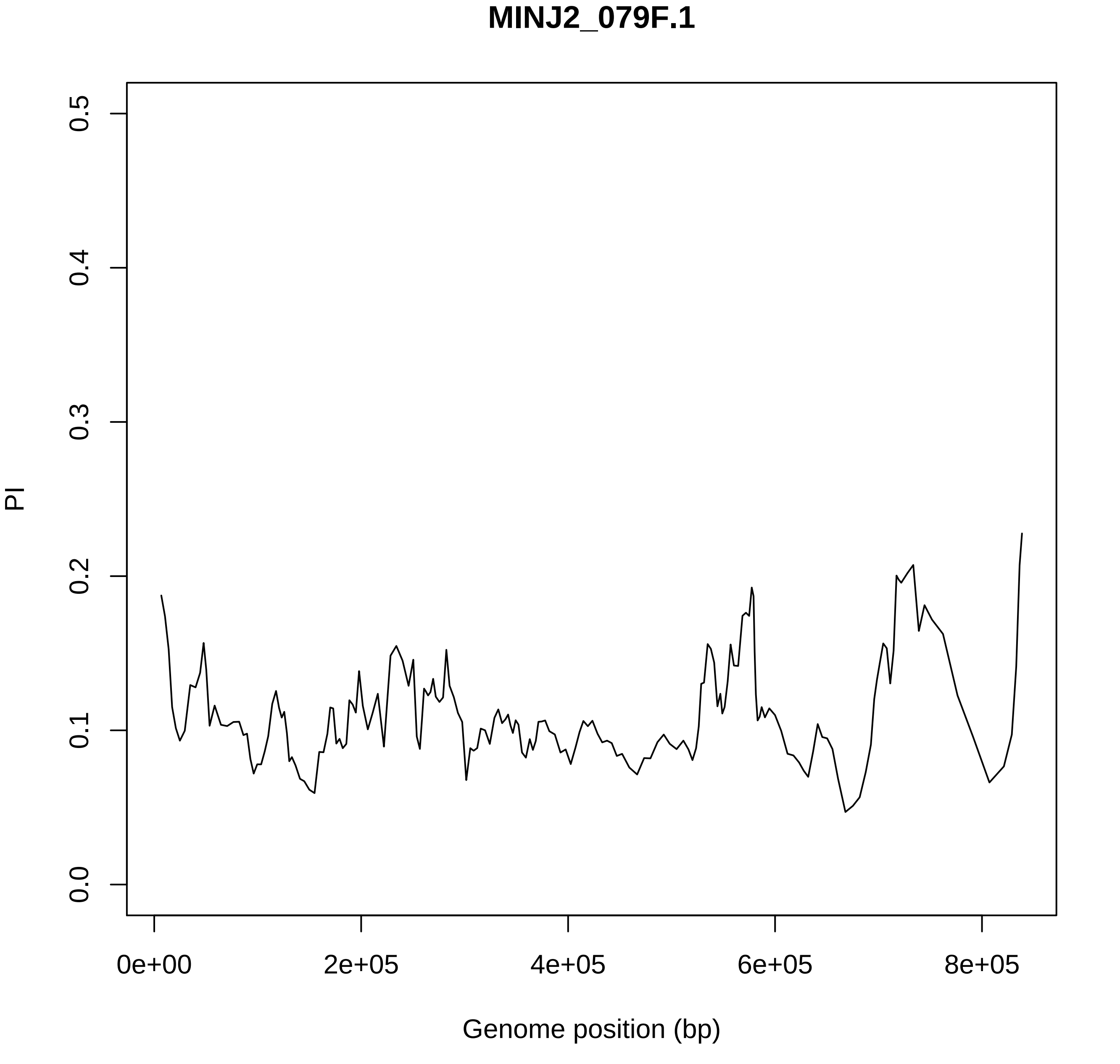

MINJ2_079F.1
PI
0.0
0.1
0.2
0.3
0.4
0.5
0e+00
2e+05
4e+05
6e+05
8e+05
Genome position (bp)

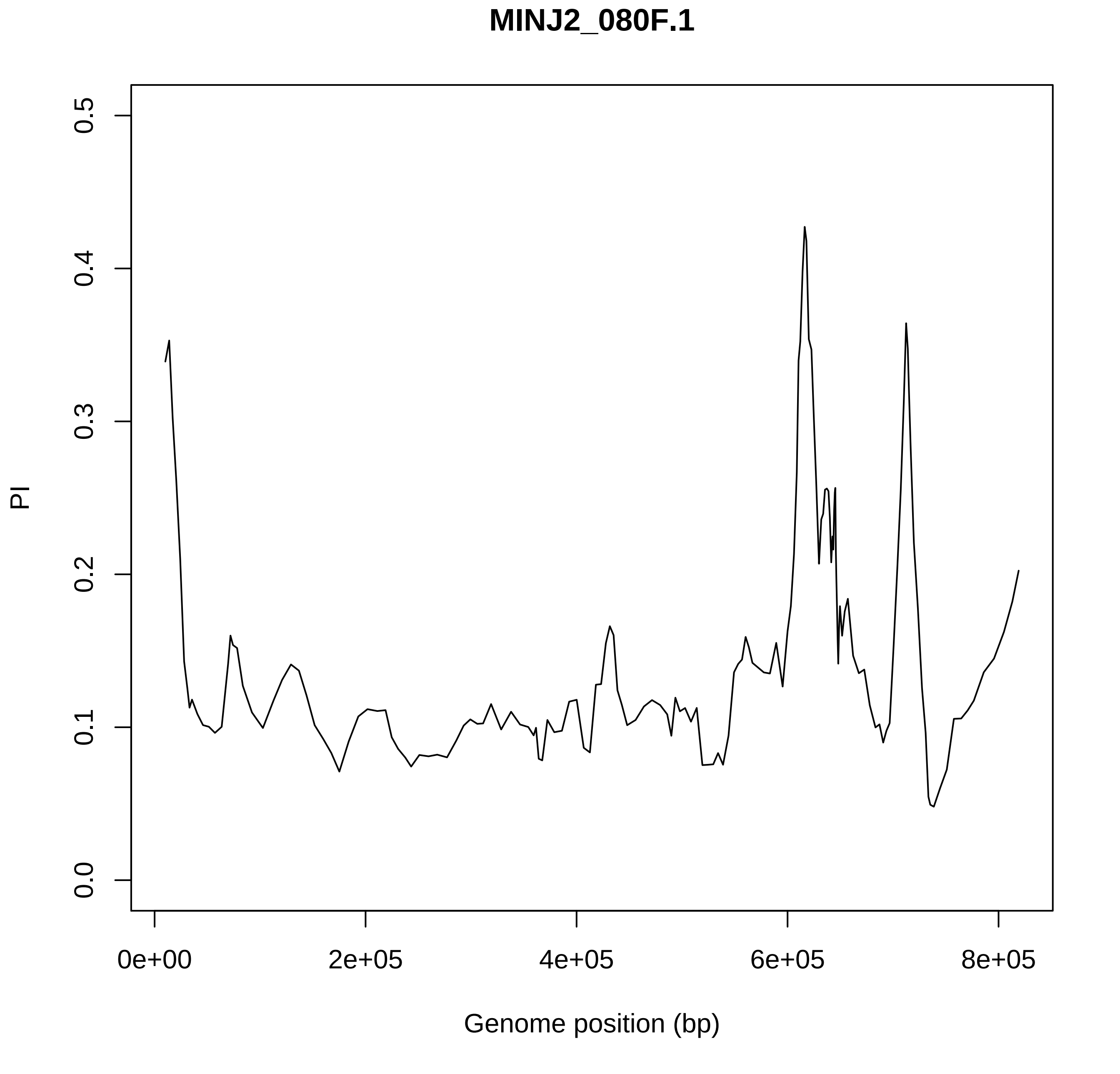
MINJ2_080F.1
PI
0.0
0.1
0.2
0.3
0.4
0.5
0e+00
2e+05
4e+05
6e+05
8e+05
Genome position (bp)

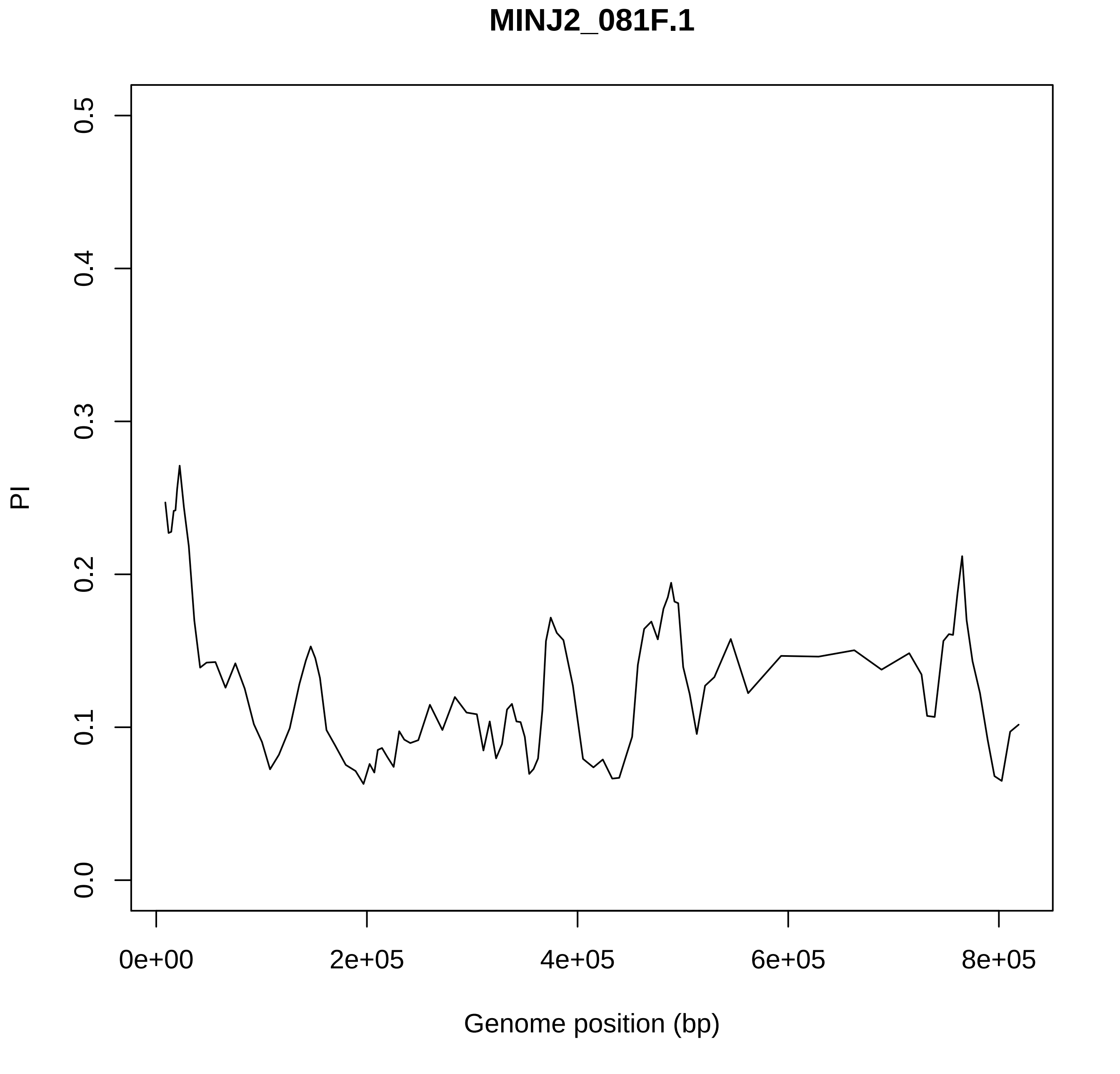
MINJ2_081F.1
PI
0.0
0.1
0.2
0.3
0.4
0.5
0e+00
2e+05
4e+05
6e+05
8e+05
Genome position (bp)

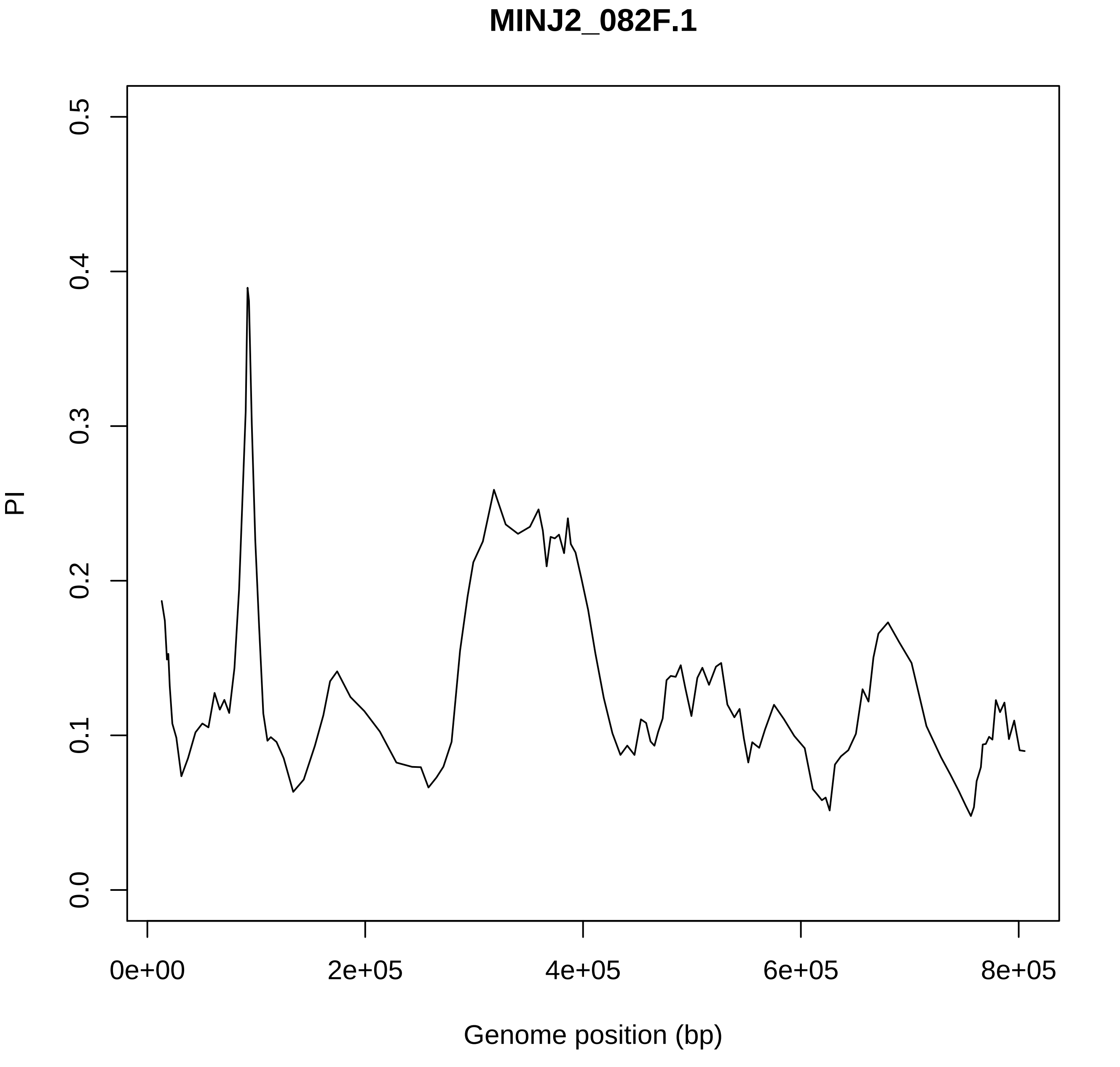
MINJ2_082F.1
PI
0.0
0.1
0.2
0.3
0.4
0.5
0e+00
2e+05
4e+05
6e+05
8e+05
Genome position (bp)

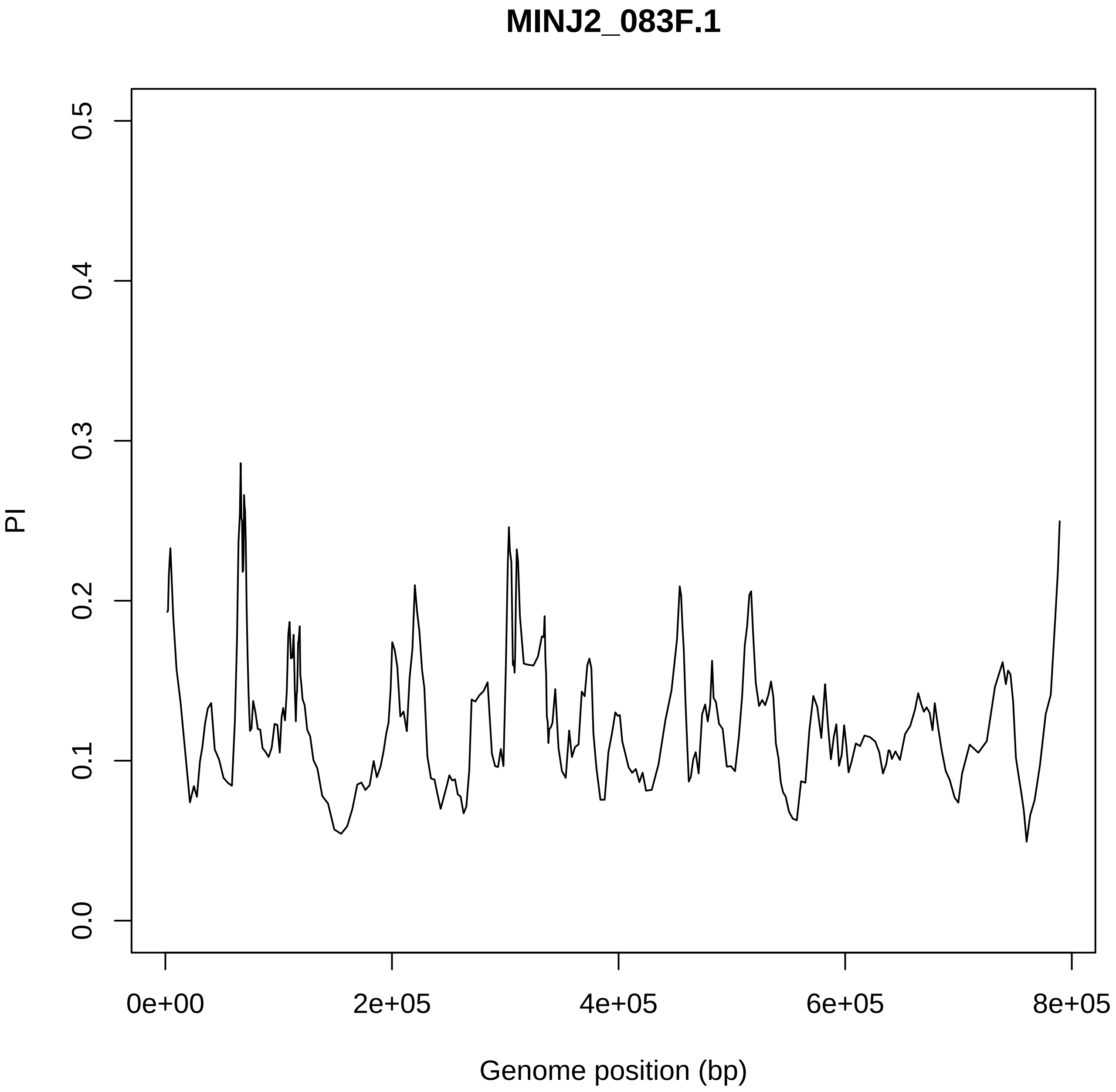
MINJ2_083F.1
PI
0.0
0.1
0.2
0.3
0.4
0.5
0e+00
2e+05
4e+05
6e+05
8e+05
Genome position (bp)

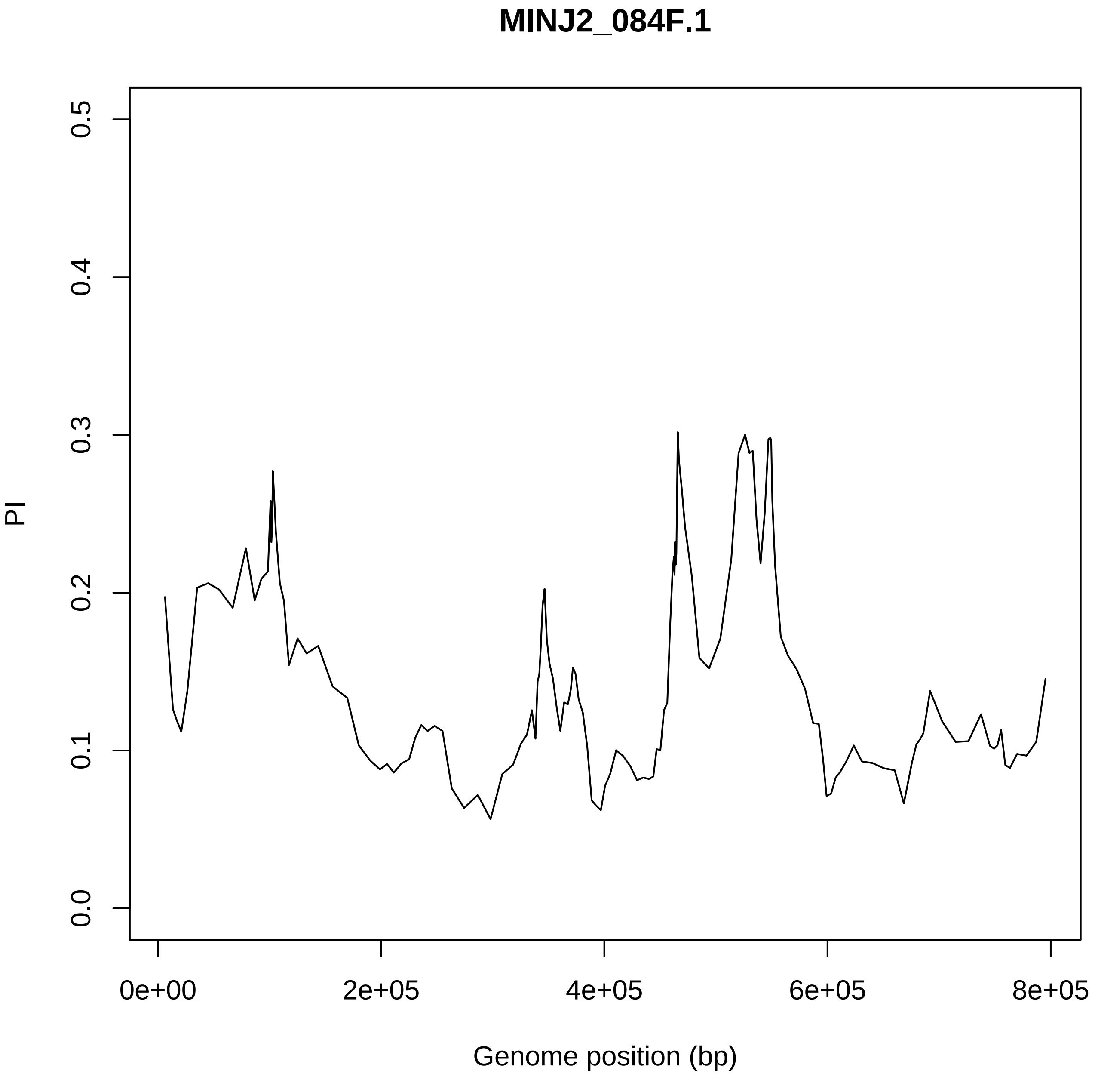
MINJ2_084F.1
PI
0.0
0.1
0.2
0.3
0.4
0.5
0e+00
2e+05
4e+05
6e+05
8e+05
Genome position (bp)

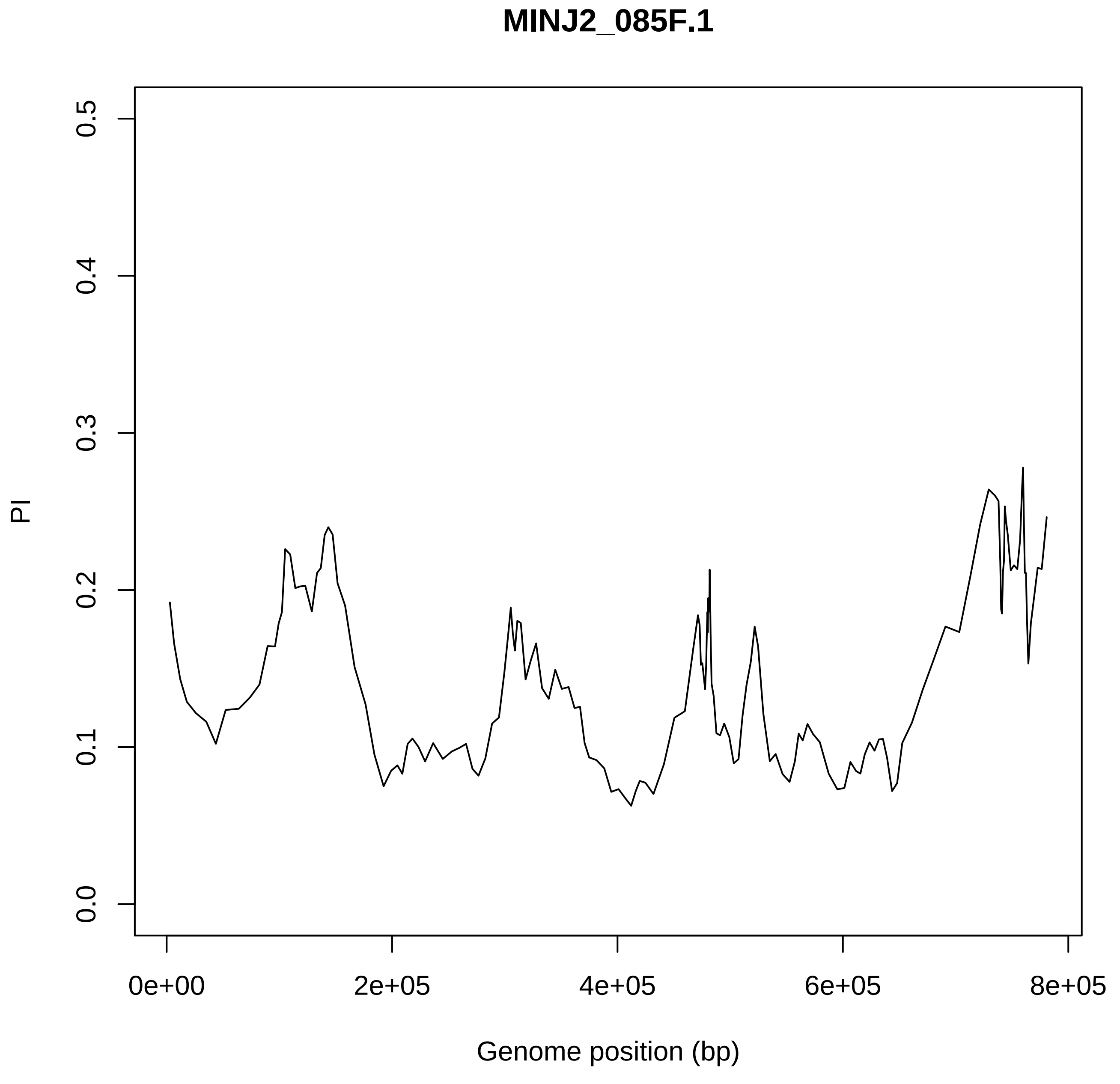

MINJ2_085F.1
PI
0.0
0.1
0.2
0.3
0.4
0.5
0e+00
2e+05
4e+05
6e+05
8e+05
Genome position (bp)

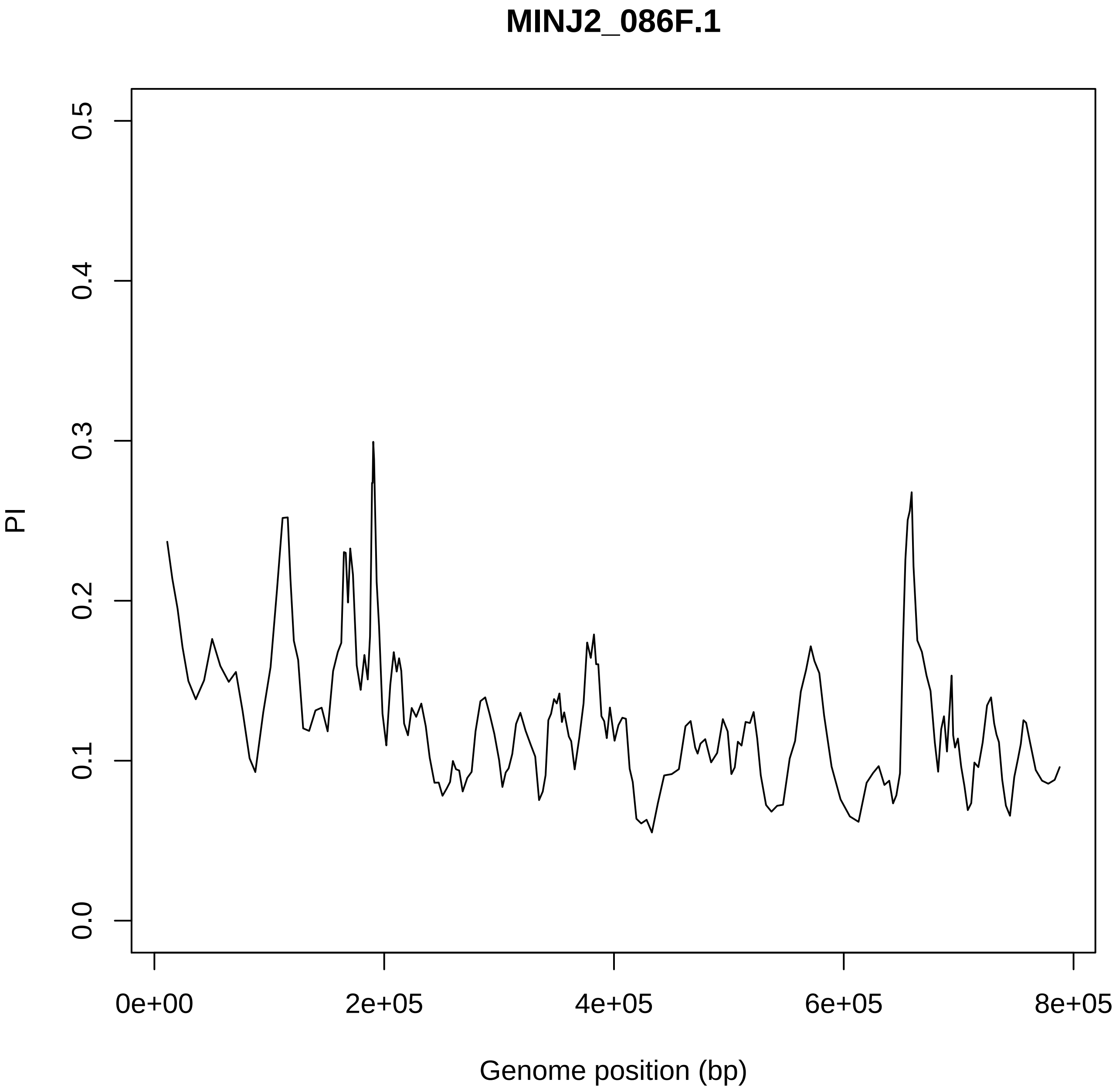

MINJ2_086F.1
PI
0.0
0.1
0.2
0.3
0.4
0.5
0e+00
2e+05
4e+05
6e+05
8e+05
Genome position (bp)

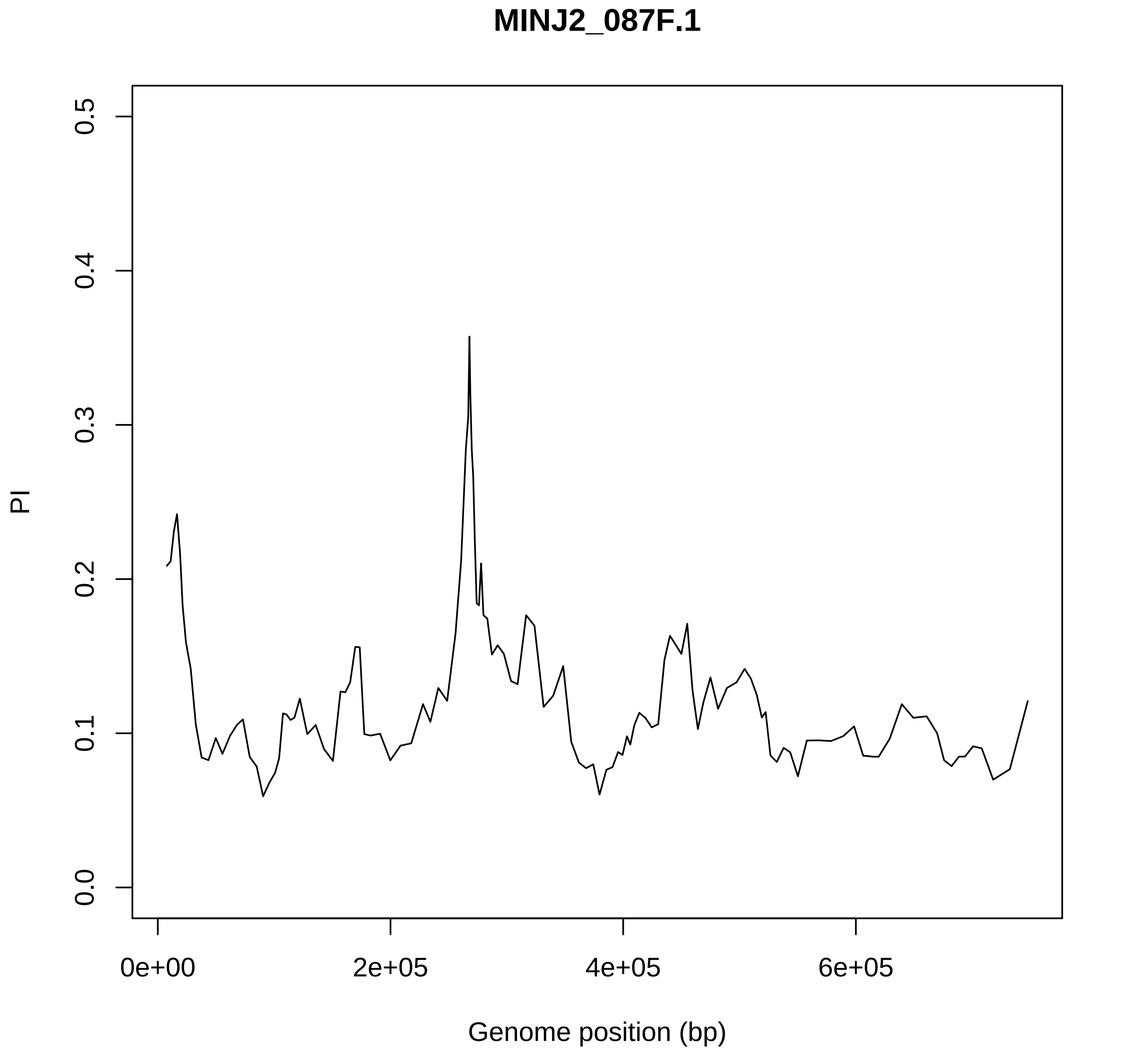

MINJ2_087F.1
PI
0.0
0.1
0.2
0.3
0.4
0.5
0e+00
2e+05
4e+05
6e+05
Genome position (bp)

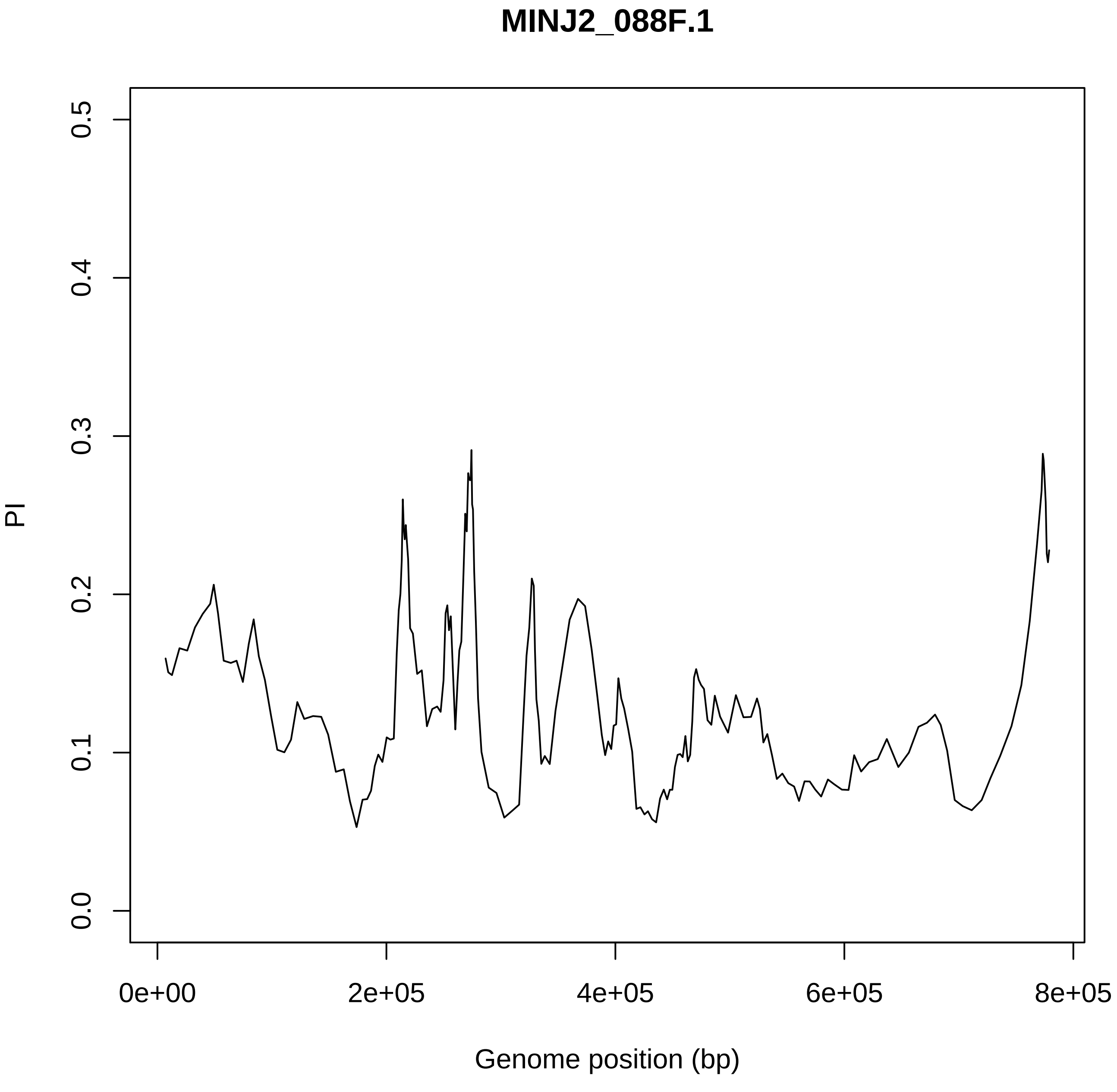

MINJ2_088F.1
PI
0.0
0.1
0.2
0.3
0.4
0.5
0e+00
2e+05
4e+05
6e+05
8e+05
Genome position (bp)

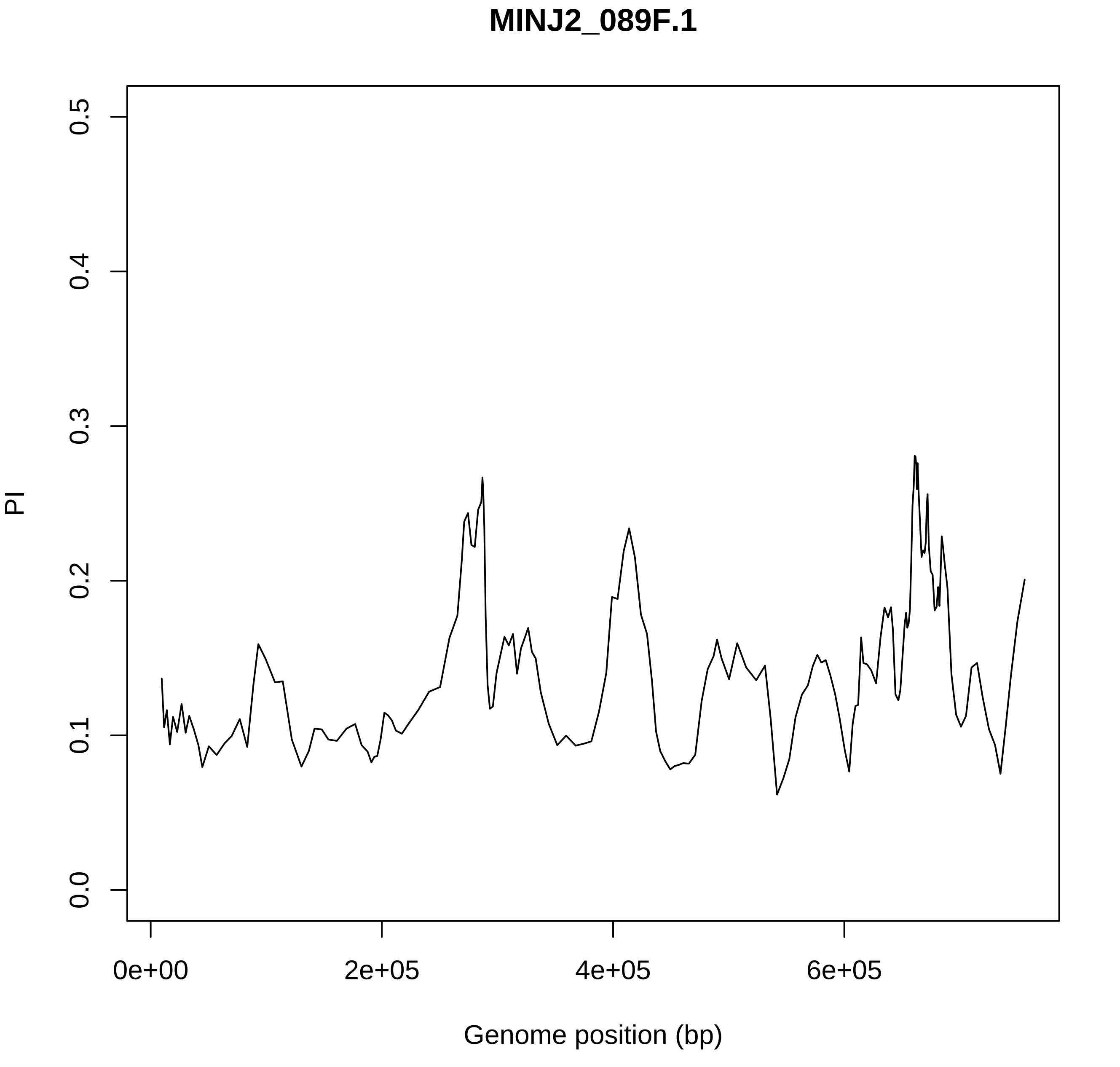
MINJ2_089F.1
PI
0.0
0.1
0.2
0.3
0.4
0.5
0e+00
2e+05
4e+05
6e+05
Genome position (bp)

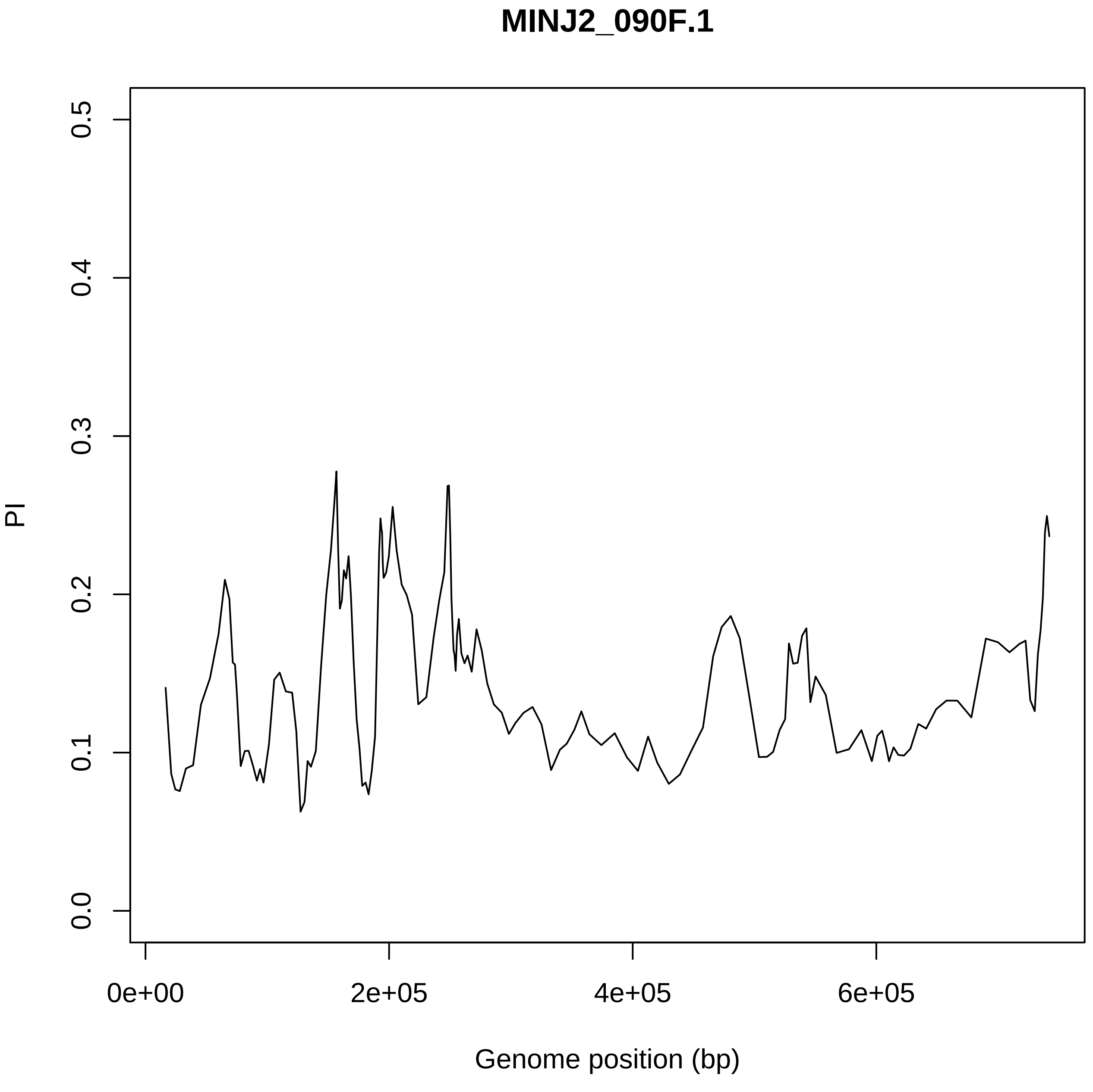
MINJ2_090F.1
PI
0.0
0.1
0.2
0.3
0.4
0.5
0e+00
2e+05
4e+05
6e+05
Genome position (bp)

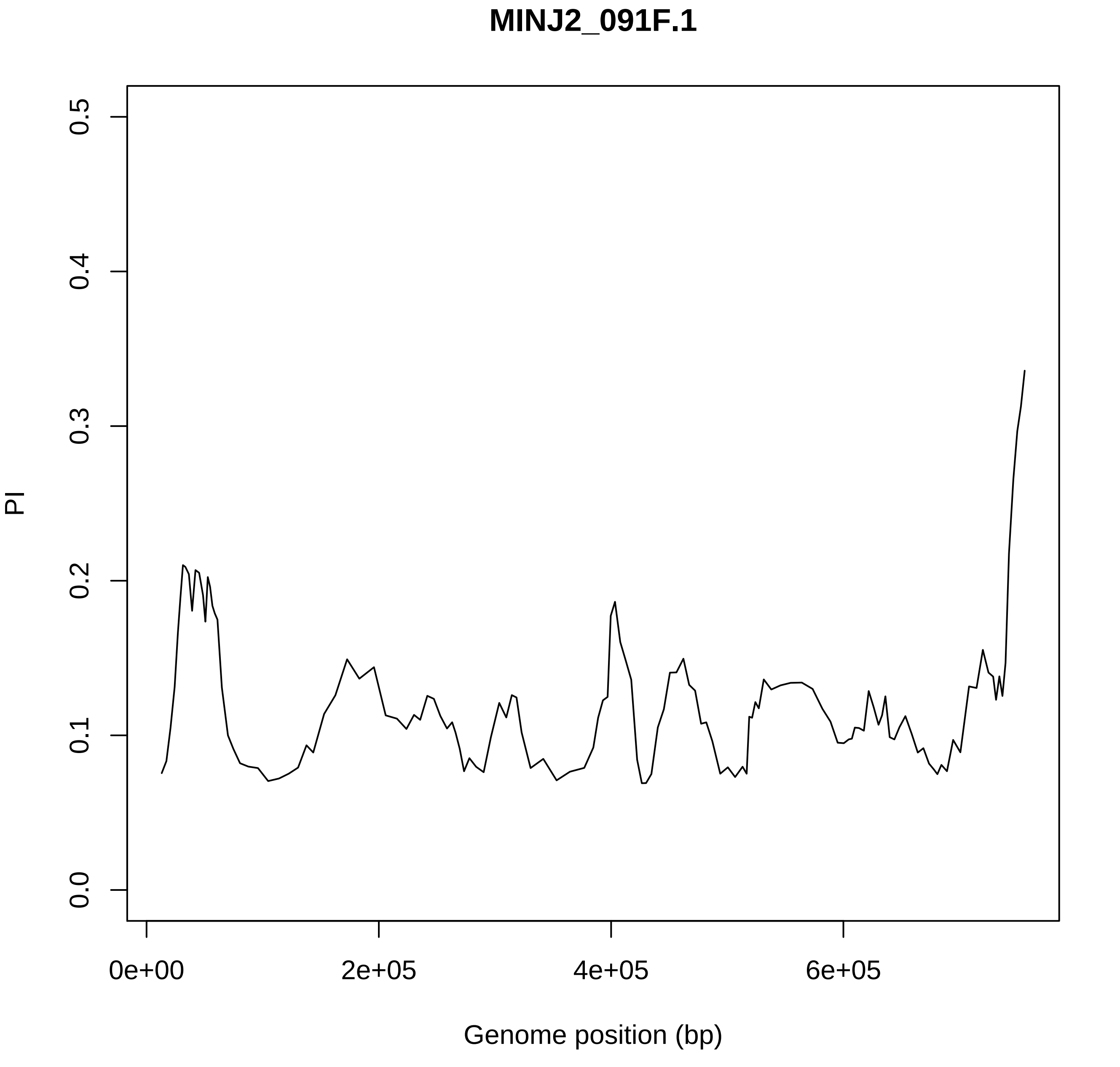
MINJ2_091F.1
PI
0.0
0.1
0.2
0.3
0.4
0.5
0e+00
2e+05
4e+05
6e+05
Genome position (bp)

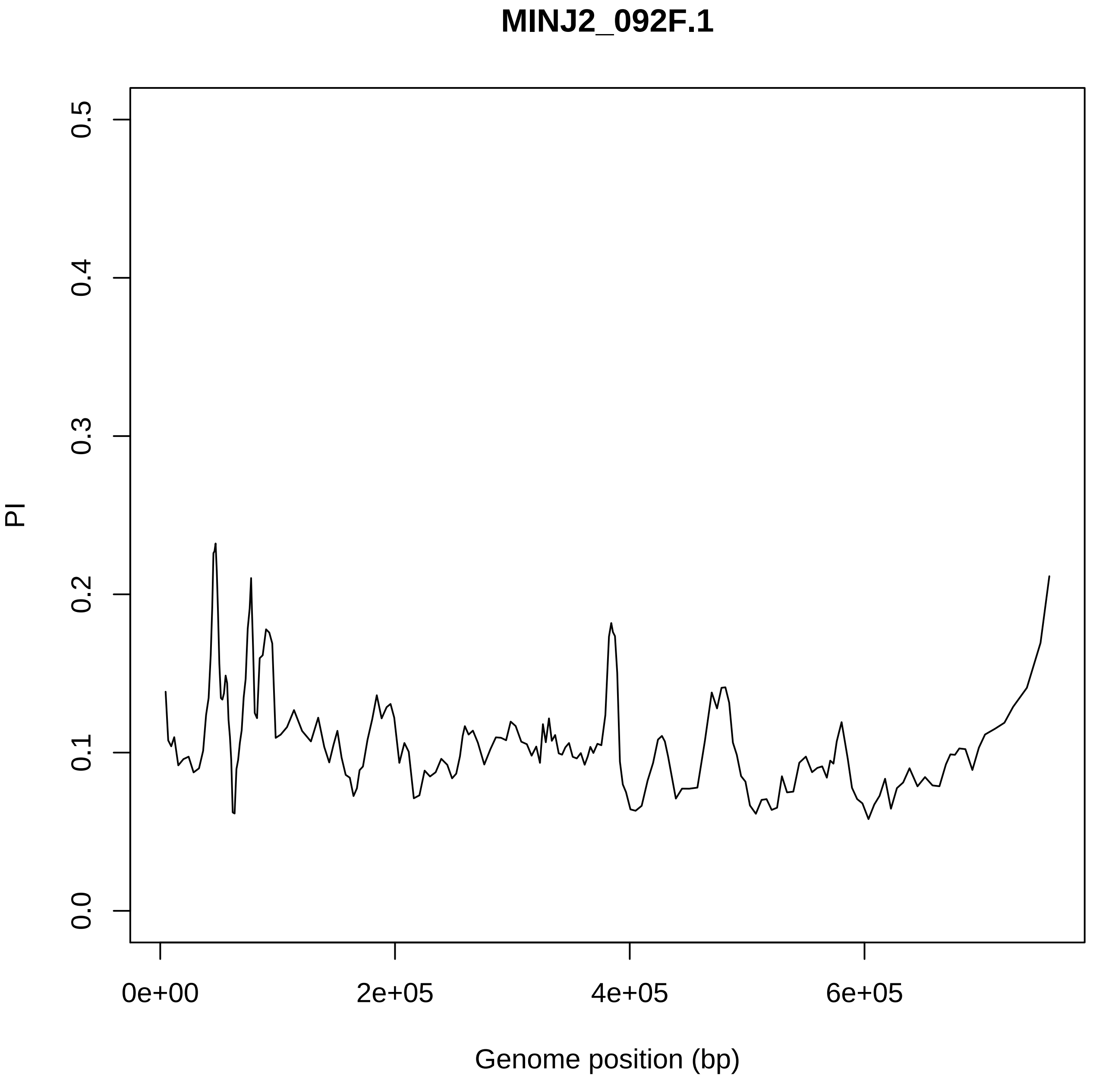
MINJ2_092F.1
PI
0.0
0.1
0.2
0.3
0.4
0.5
0e+00
2e+05
4e+05
6e+05
Genome position (bp)

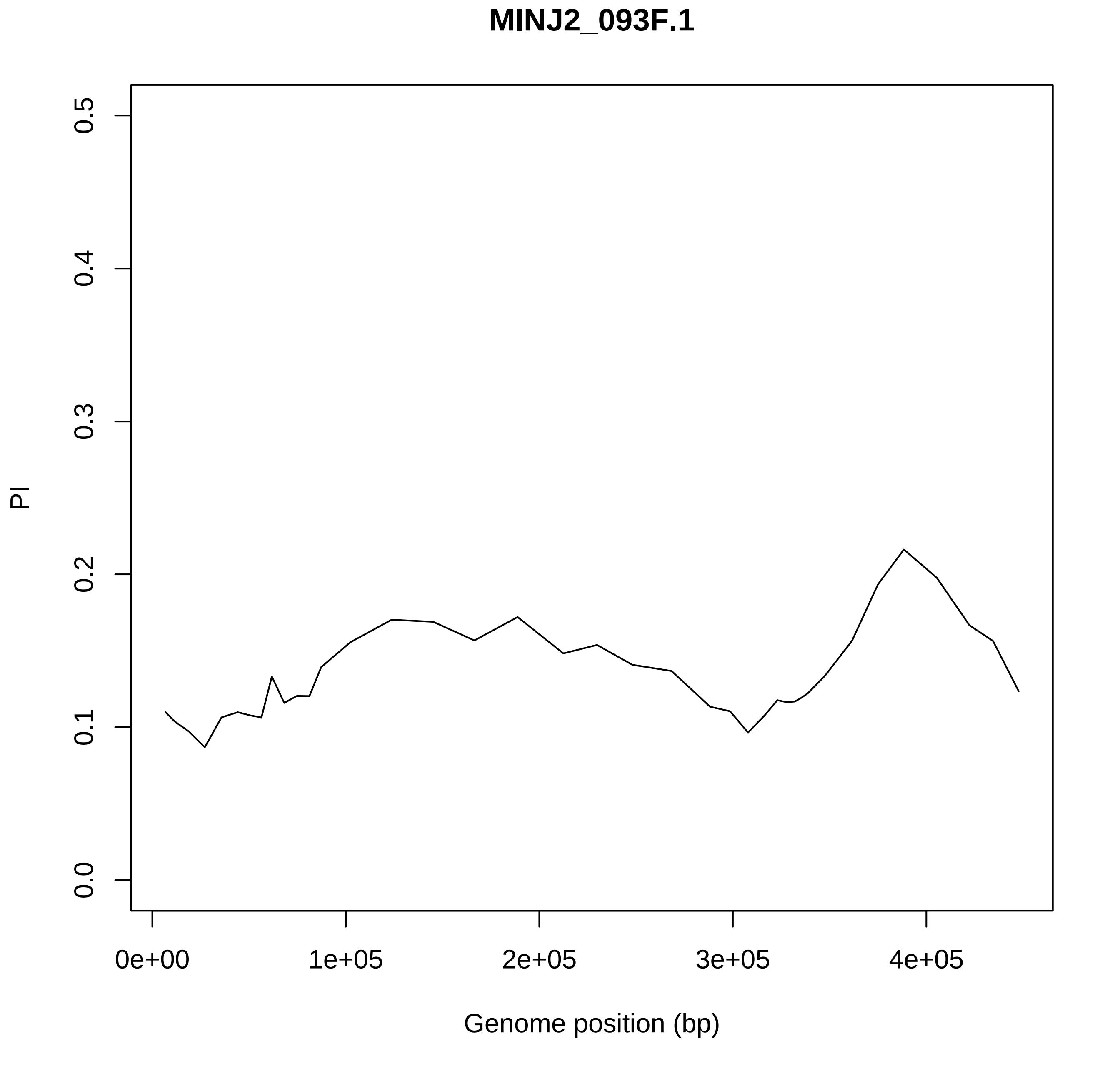

MINJ2_093F.1
PI
0.0
0.1
0.2
0.3
0.4
0.5
0e+00
1e+05
2e+05
3e+05
4e+05
Genome position (bp)

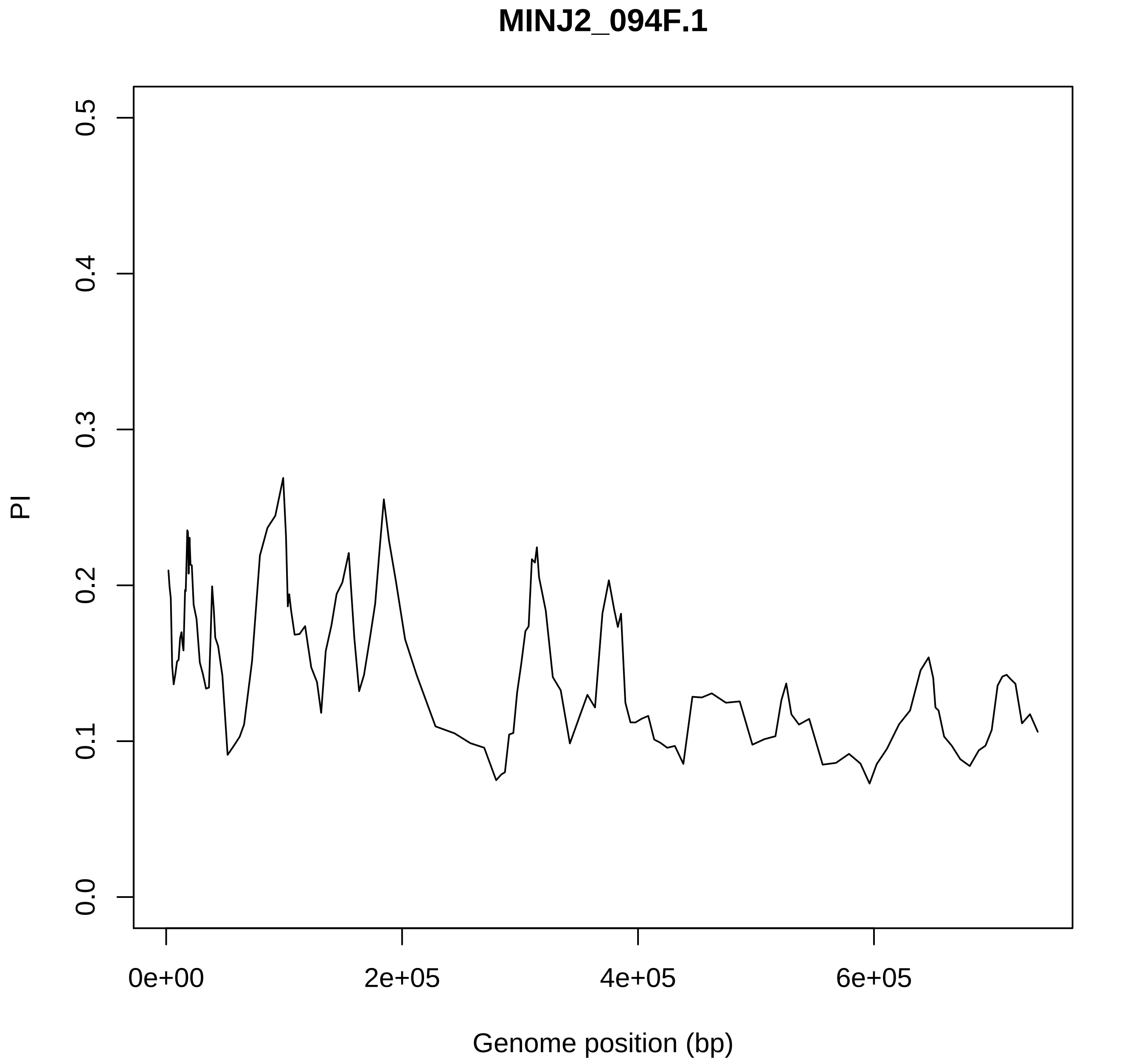

MINJ2_094F.1
PI
0.0
0.1
0.2
0.3
0.4
0.5
0e+00
2e+05
4e+05
6e+05
Genome position (bp)

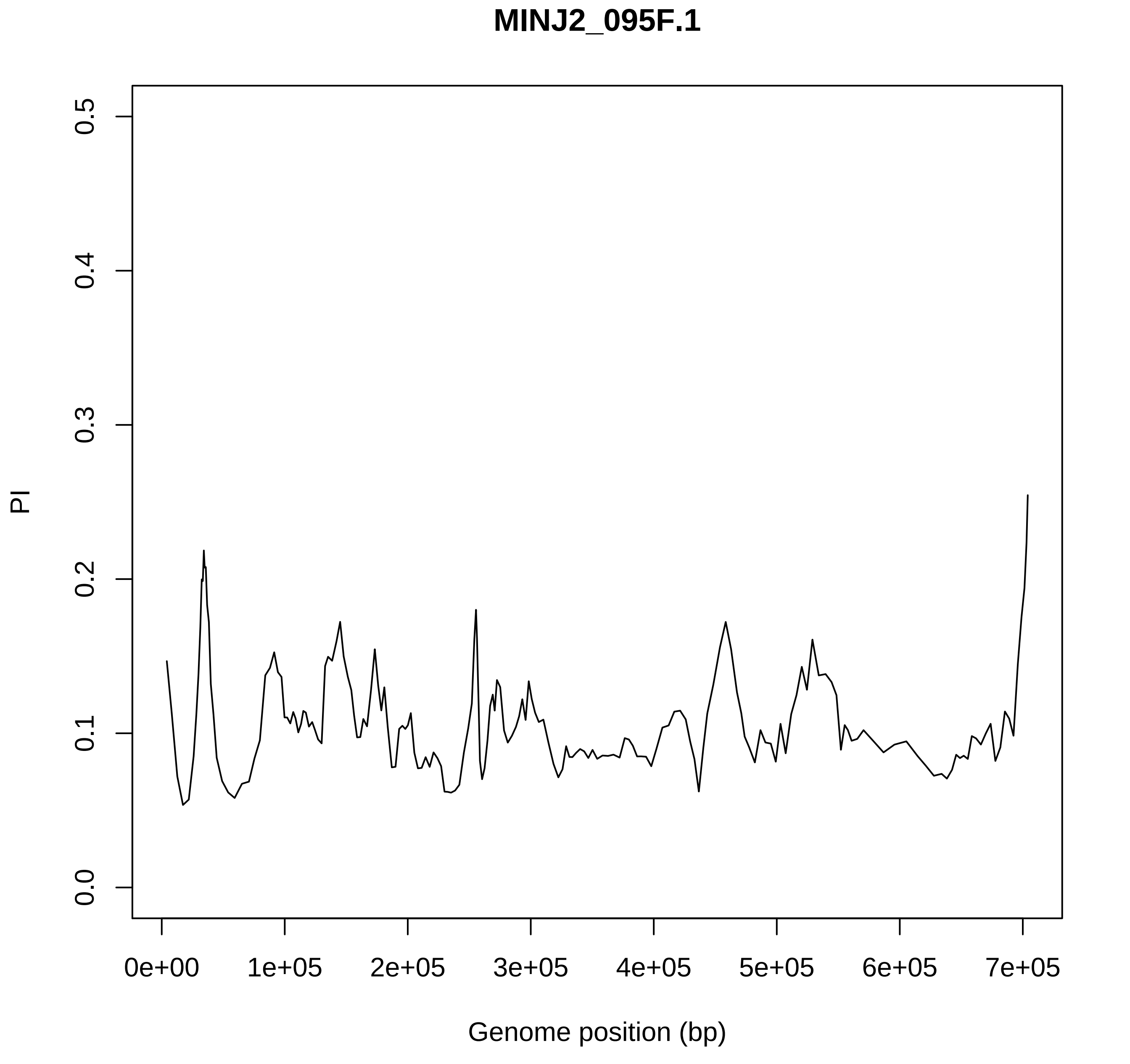

MINJ2_095F.1
PI
0.0
0.1
0.2
0.3
0.4
0.5
0e+00
1e+05
2e+05
3e+05
4e+05
5e+05
6e+05
7e+05
Genome position (bp)

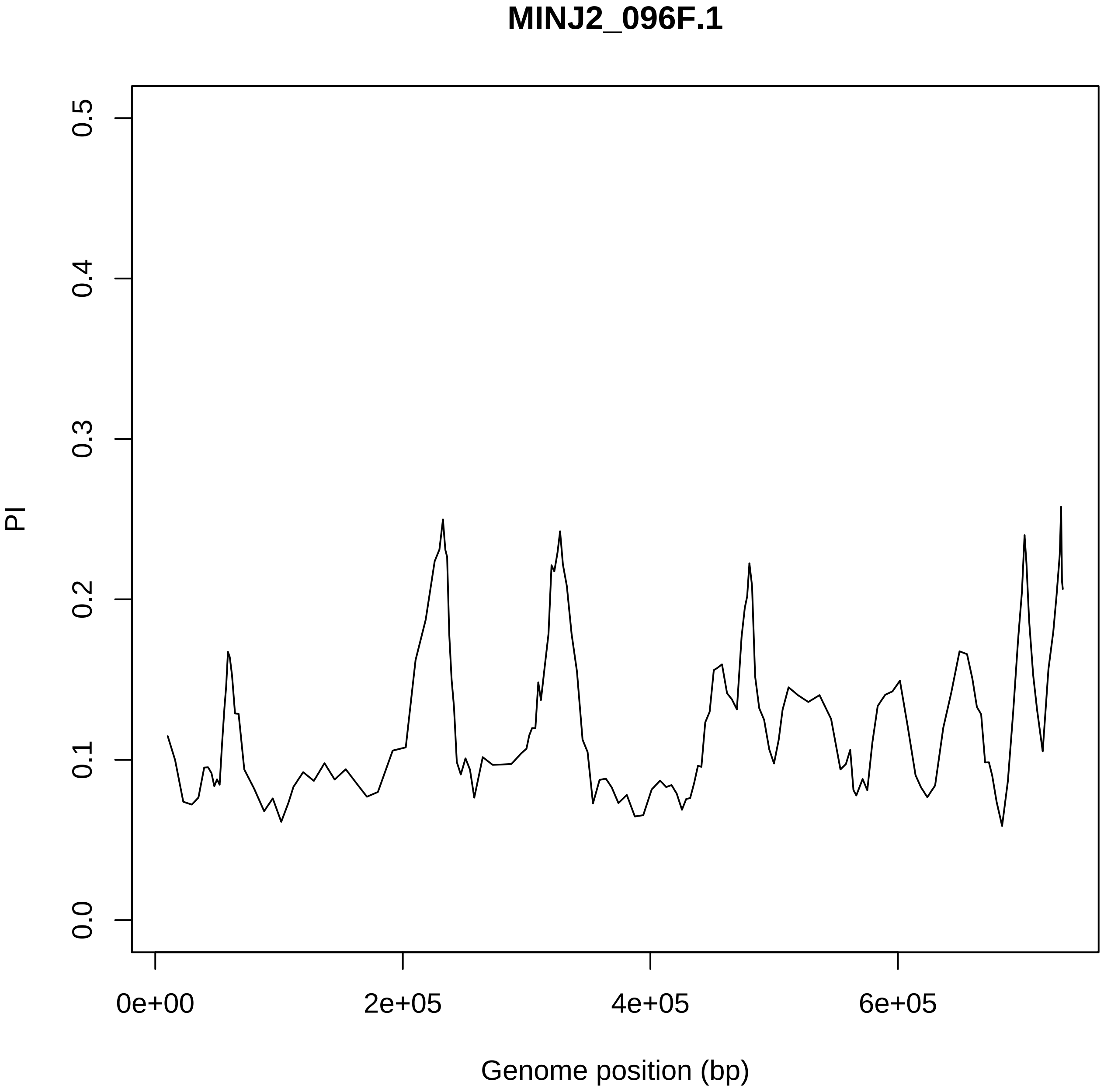

MINJ2_096F.1
PI
0.0
0.1
0.2
0.3
0.4
0.5
0e+00
2e+05
4e+05
6e+05
Genome position (bp)

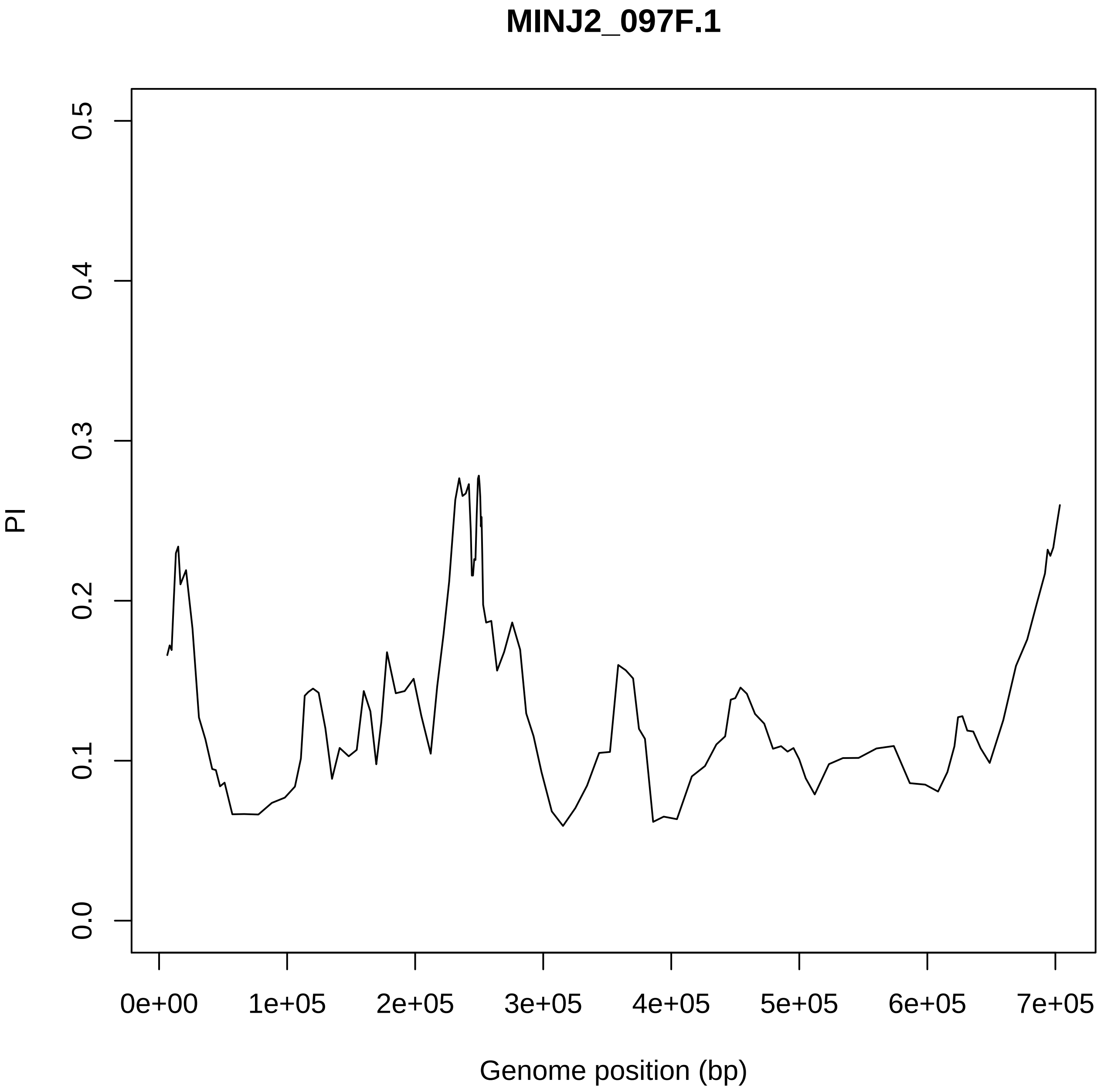
MINJ2_097F.1
PI
0.0
0.1
0.2
0.3
0.4
0.5
0e+00
1e+05
2e+05
3e+05
4e+05
5e+05
6e+05
7e+05
Genome position (bp)

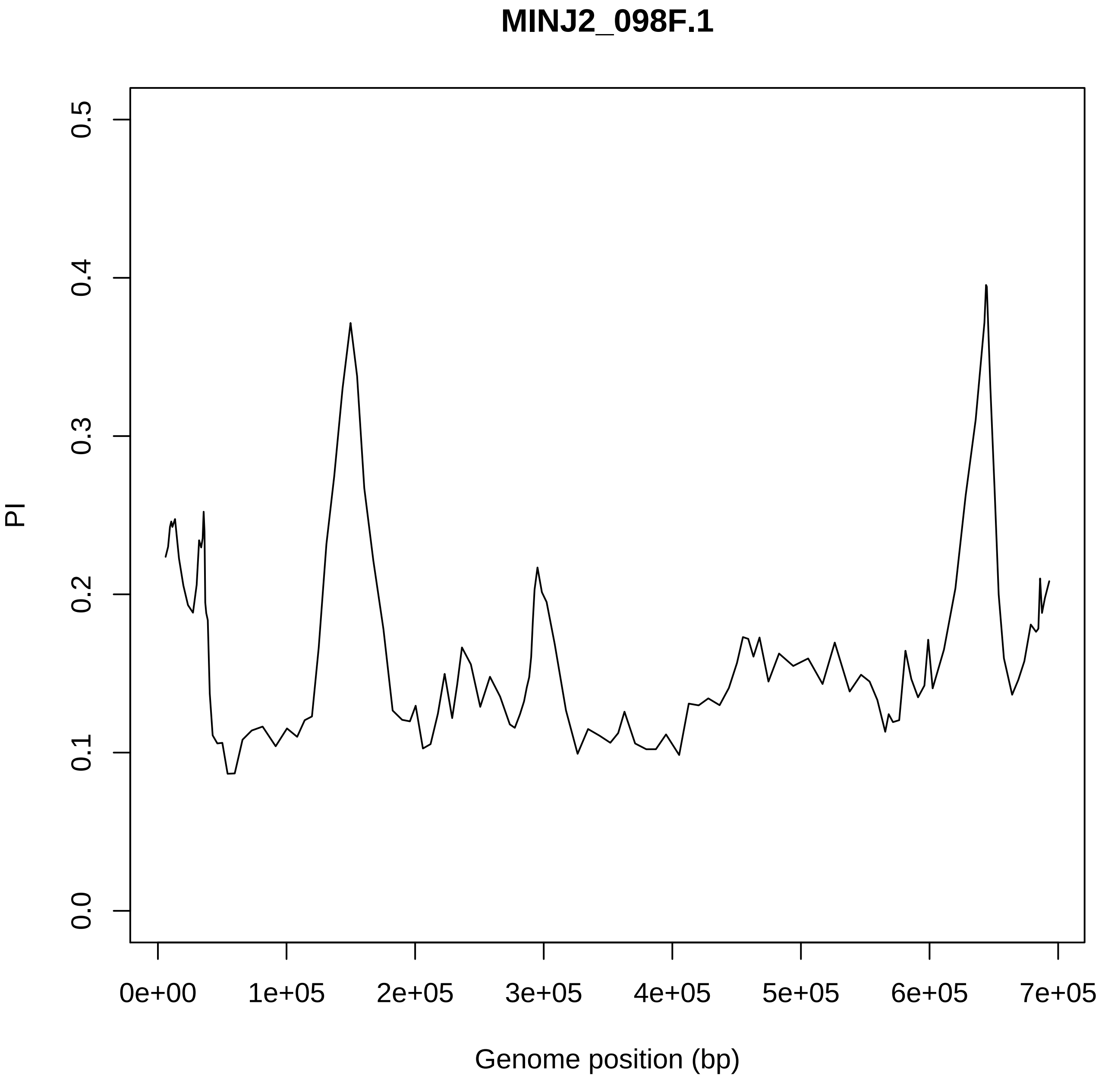
MINJ2_098F.1
PI
0.0
0.1
0.2
0.3
0.4
0.5
0e+00
1e+05
2e+05
3e+05
4e+05
5e+05
6e+05
7e+05
Genome position (bp)

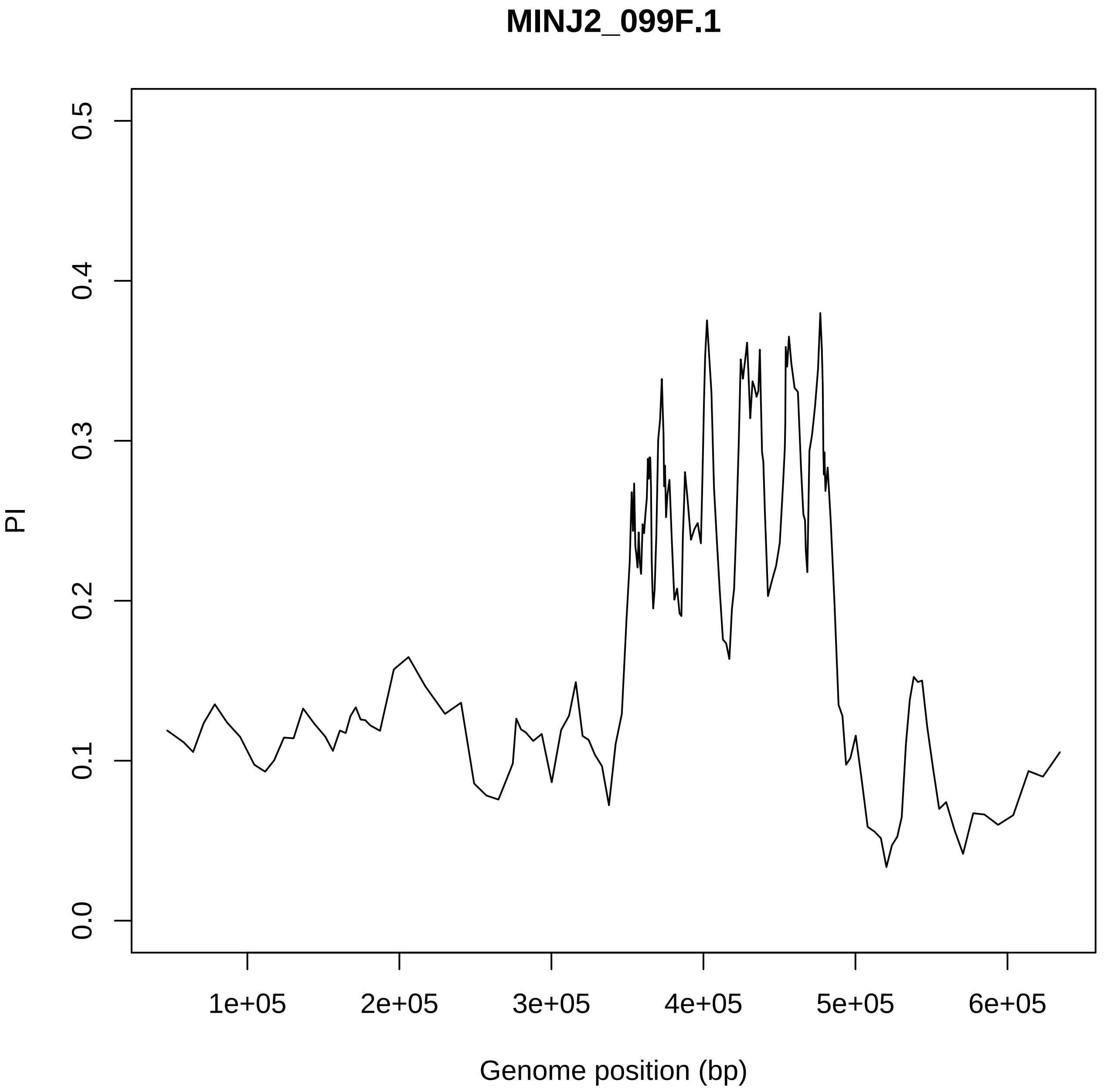

MINJ2_099F.1
PI
0.0
0.1
0.2
0.3
0.4
0.5
1e+05
2e+05
3e+05
4e+05
5e+05
6e+05
Genome position (bp)

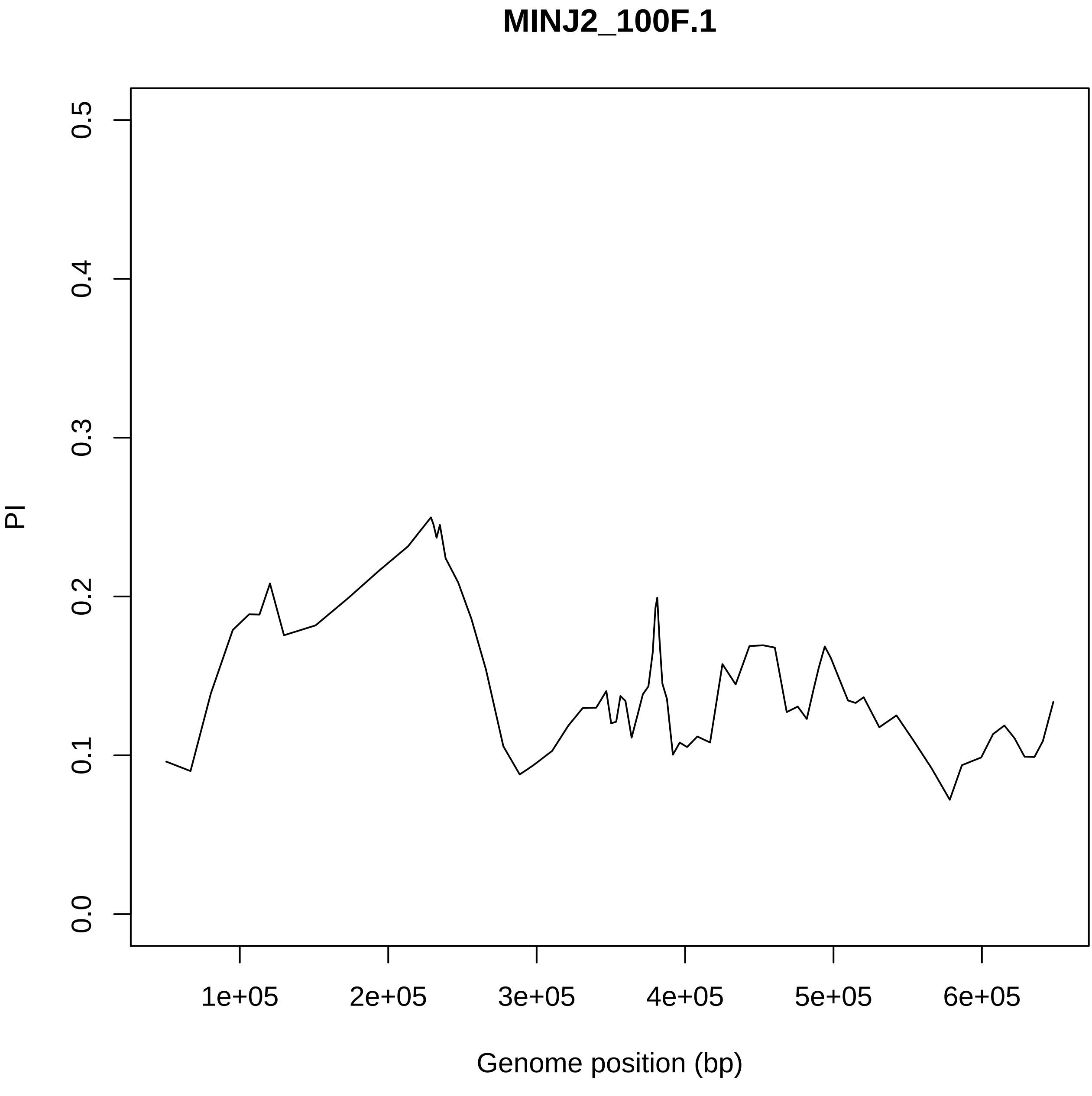

MINJ2_100F.1
PI
0.0
0.1
0.2
0.3
0.4
0.5
1e+05
2e+05
3e+05
4e+05
5e+05
6e+05
Genome position (bp)

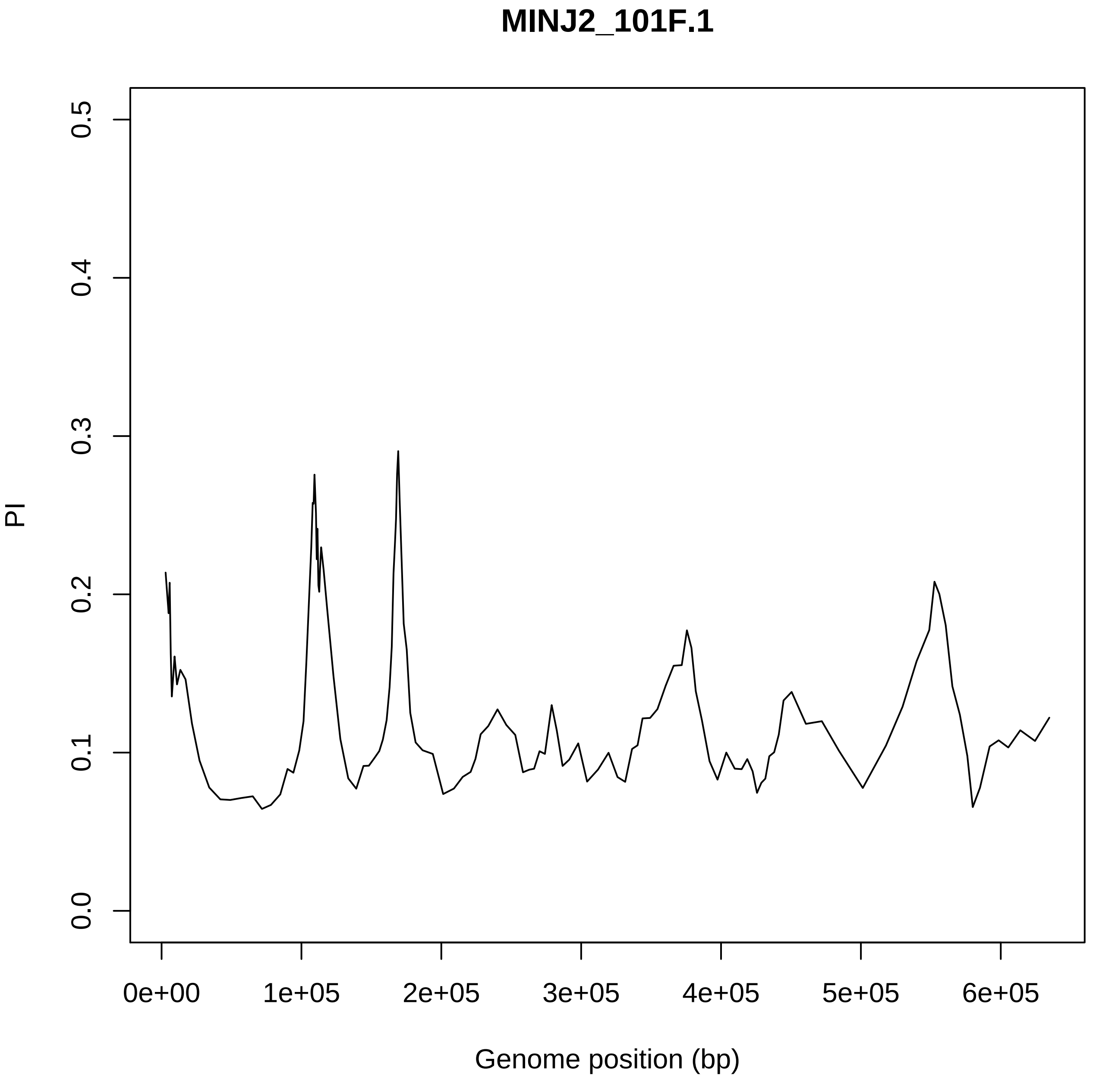
MINJ2_101F.1
PI
0.0
0.1
0.2
0.3
0.4
0.5
0e+00
1e+05
2e+05
3e+05
4e+05
5e+05
6e+05
Genome position (bp)

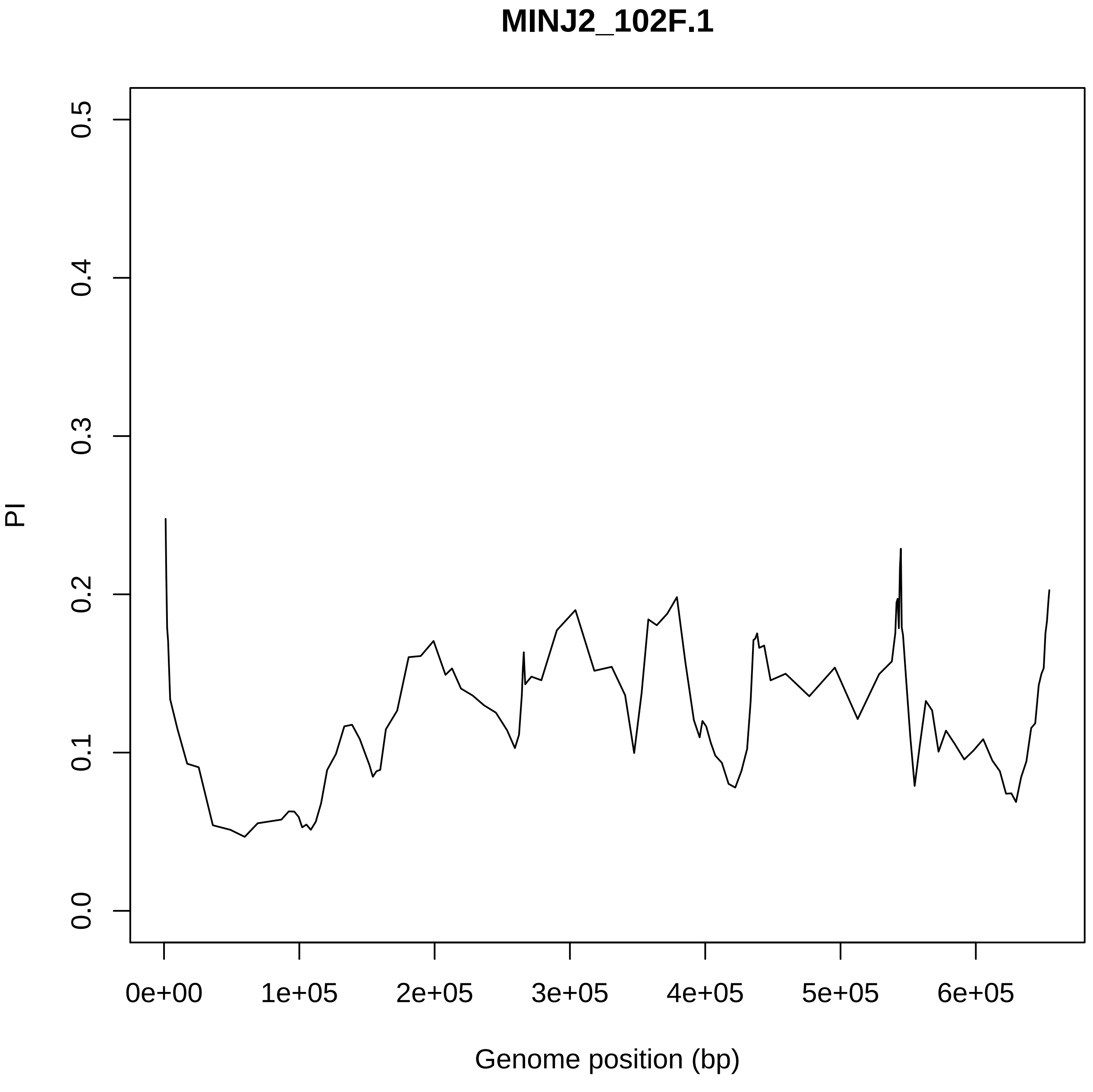
MINJ2_102F.1
PI
0.0
0.1
0.2
0.3
0.4
0.5
0e+00
1e+05
2e+05
3e+05
4e+05
5e+05
6e+05
Genome position (bp)

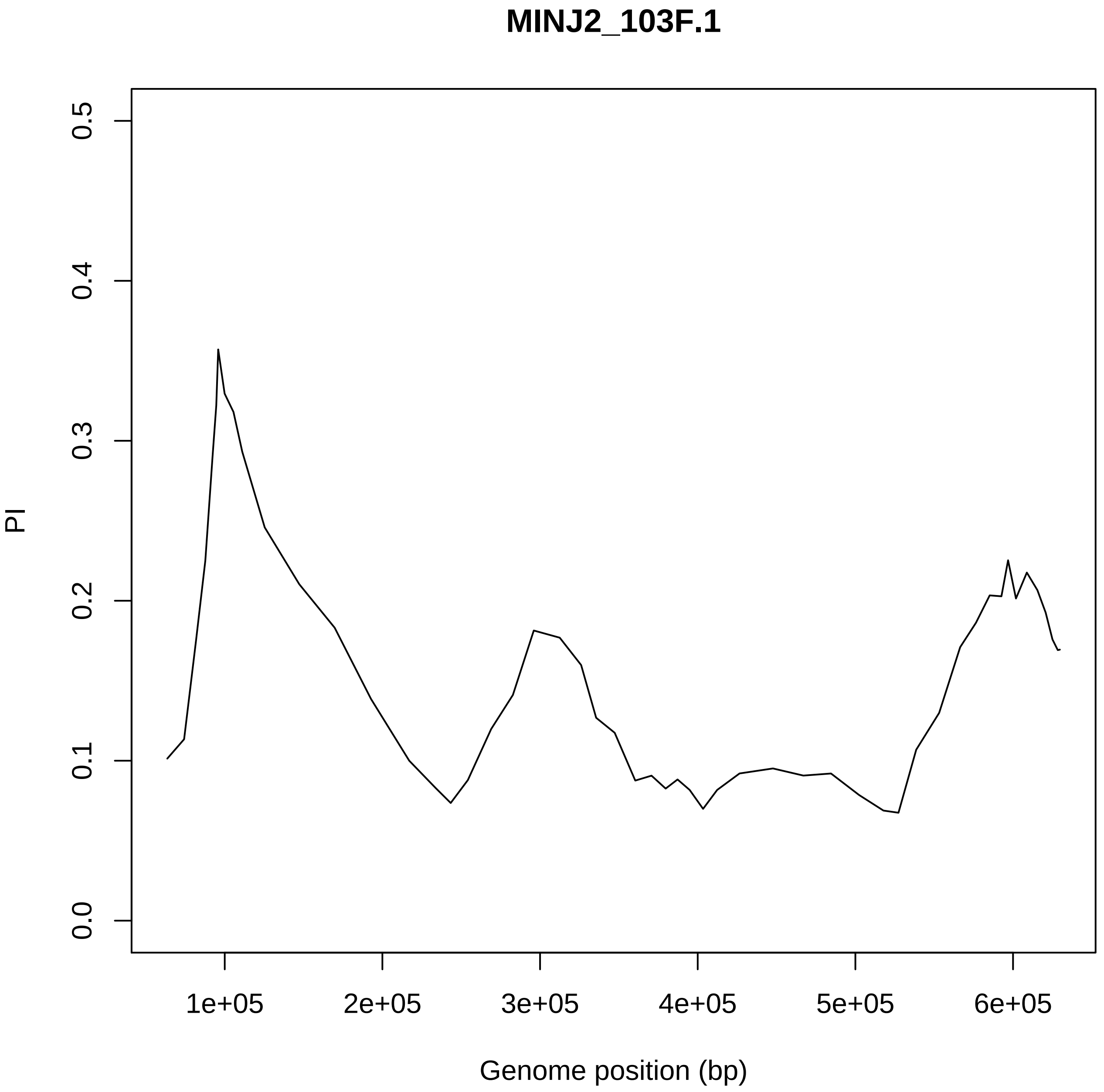
MINJ2_103F.1
PI
0.0
0.1
0.2
0.3
0.4
0.5
1e+05
2e+05
3e+05
4e+05
5e+05
6e+05
Genome position (bp)

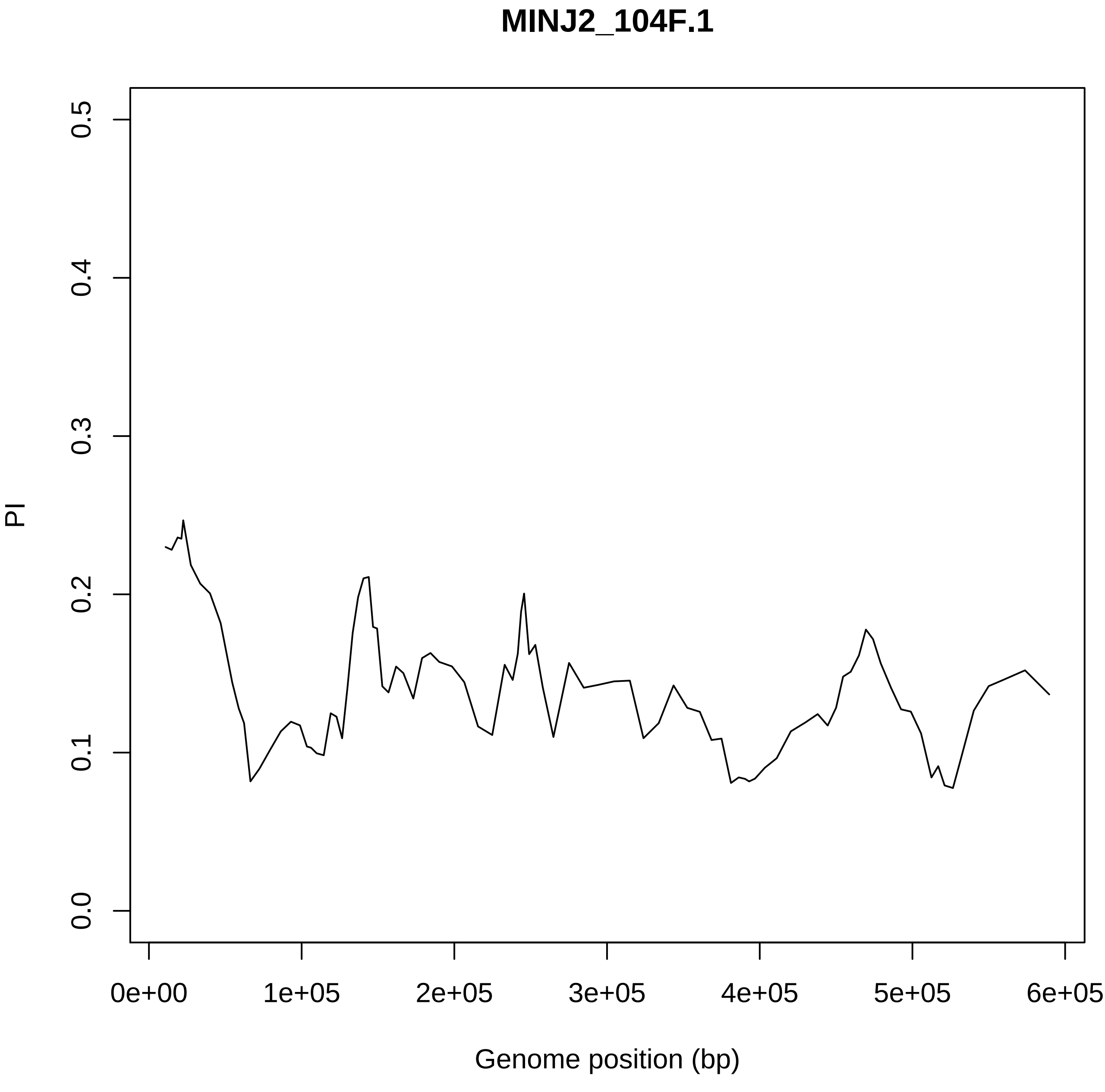

MINJ2_104F.1
PI
0.0
0.1
0.2
0.3
0.4
0.5
0e+00
1e+05
2e+05
3e+05
4e+05
5e+05
6e+05
Genome position (bp)

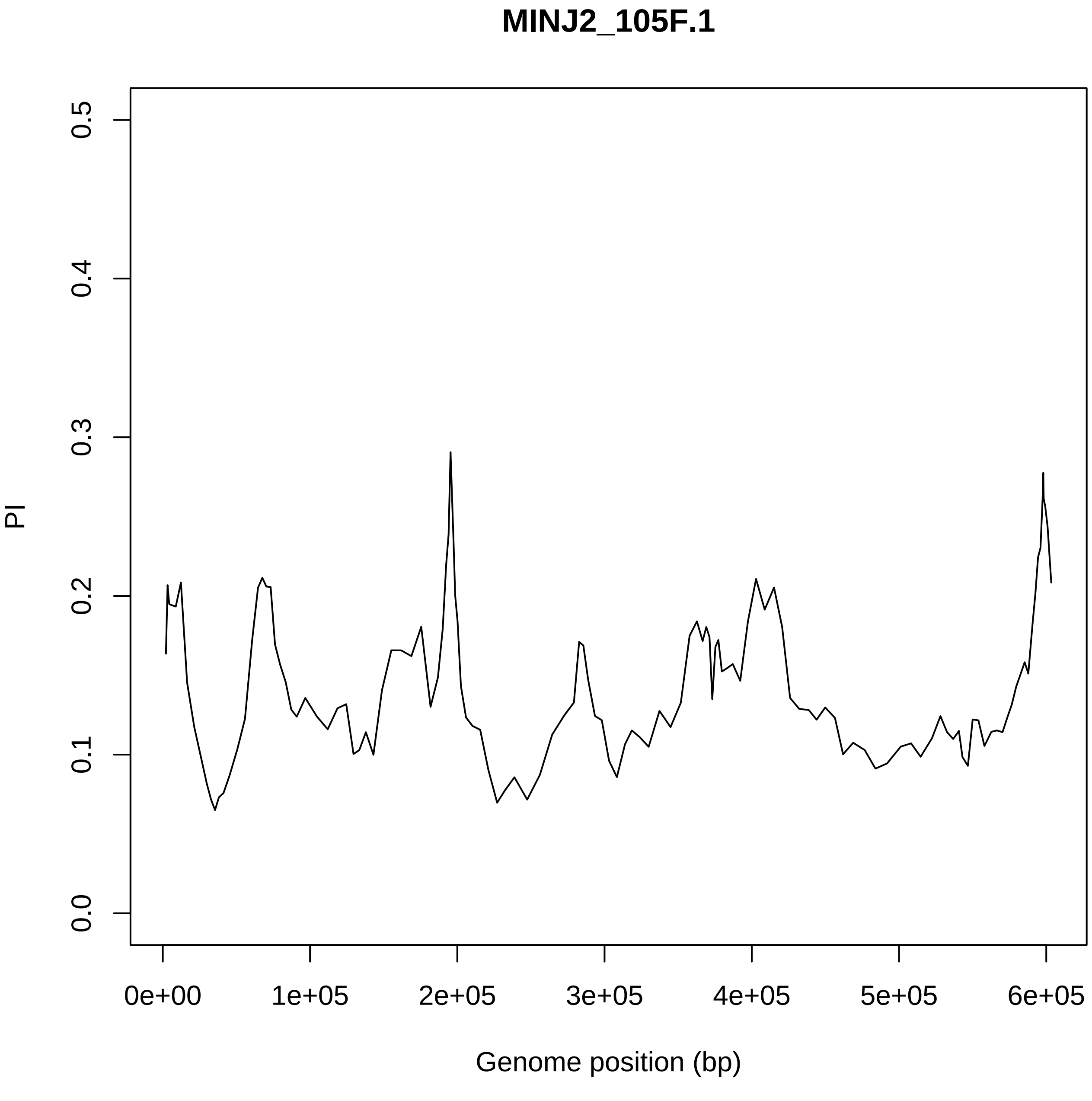
MINJ2_105F.1
PI
0.0
0.1
0.2
0.3
0.4
0.5
0e+00
1e+05
2e+05
3e+05
4e+05
5e+05
6e+05
Genome position (bp)

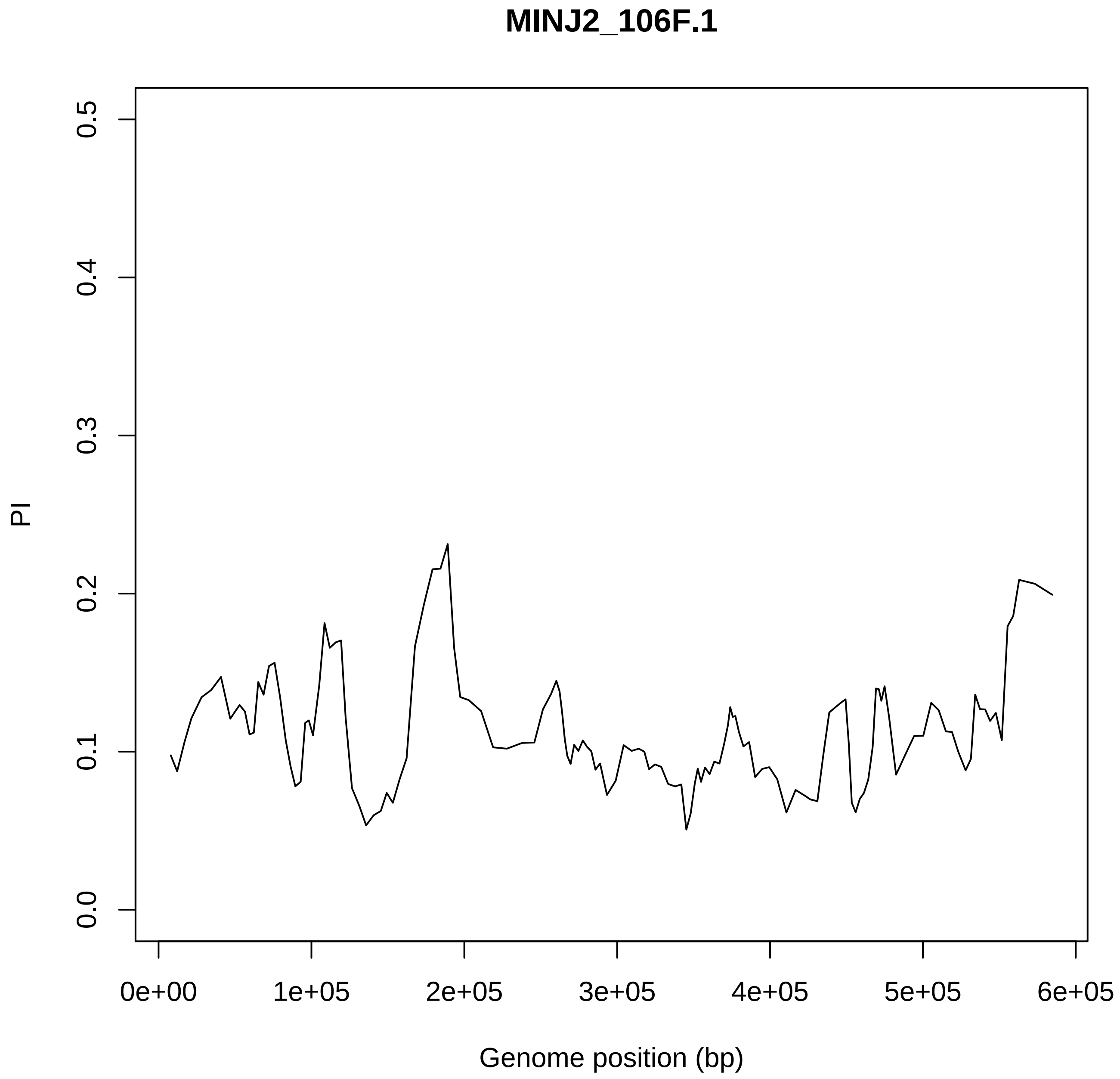

MINJ2_106F.1
PI
0.0
0.1
0.2
0.3
0.4
0.5
0e+00
1e+05
2e+05
3e+05
4e+05
5e+05
6e+05
Genome position (bp)

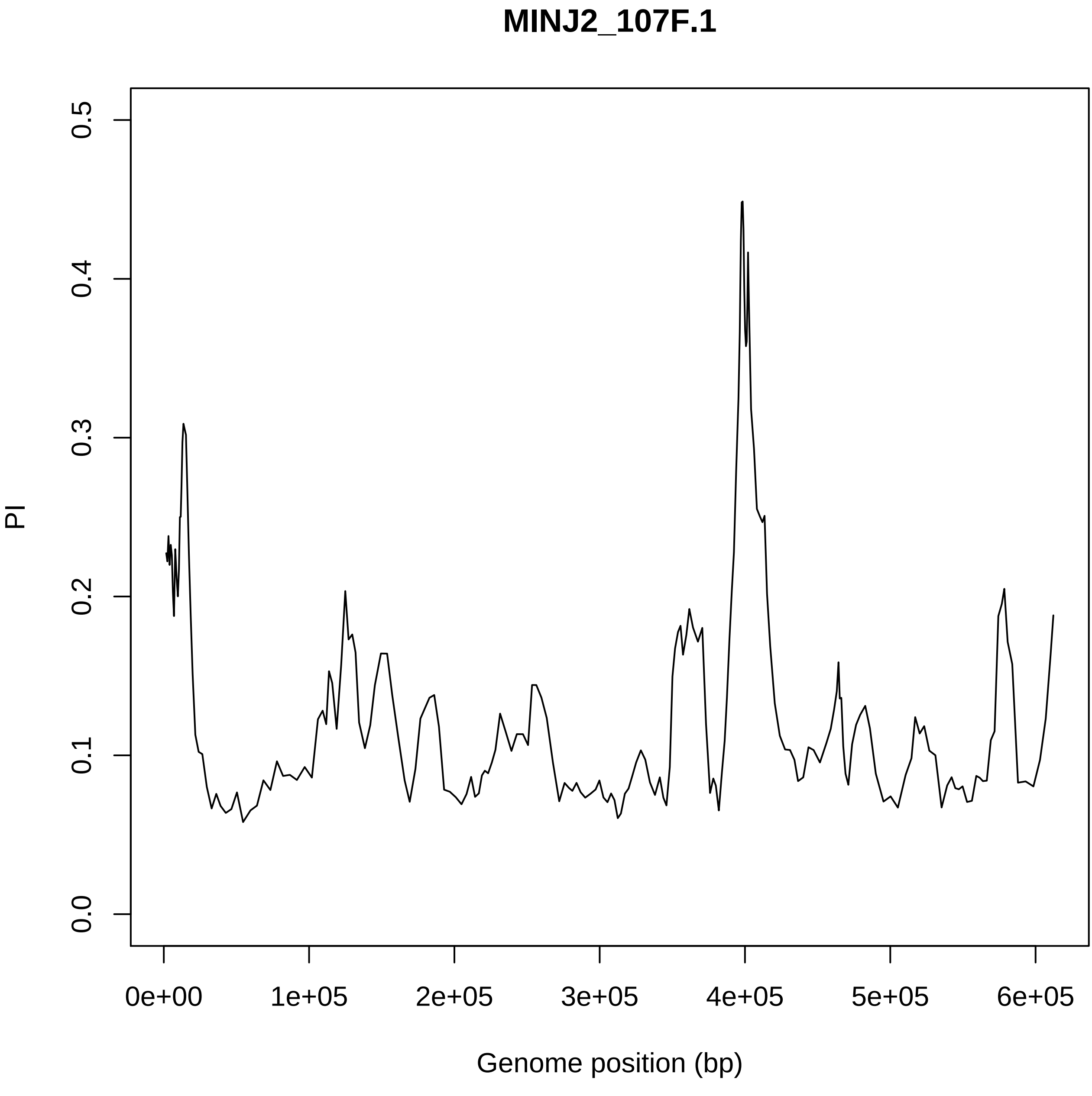

MINJ2_107F.1
PI
0.0
0.1
0.2
0.3
0.4
0.5
0e+00
1e+05
2e+05
3e+05
4e+05
5e+05
6e+05
Genome position (bp)

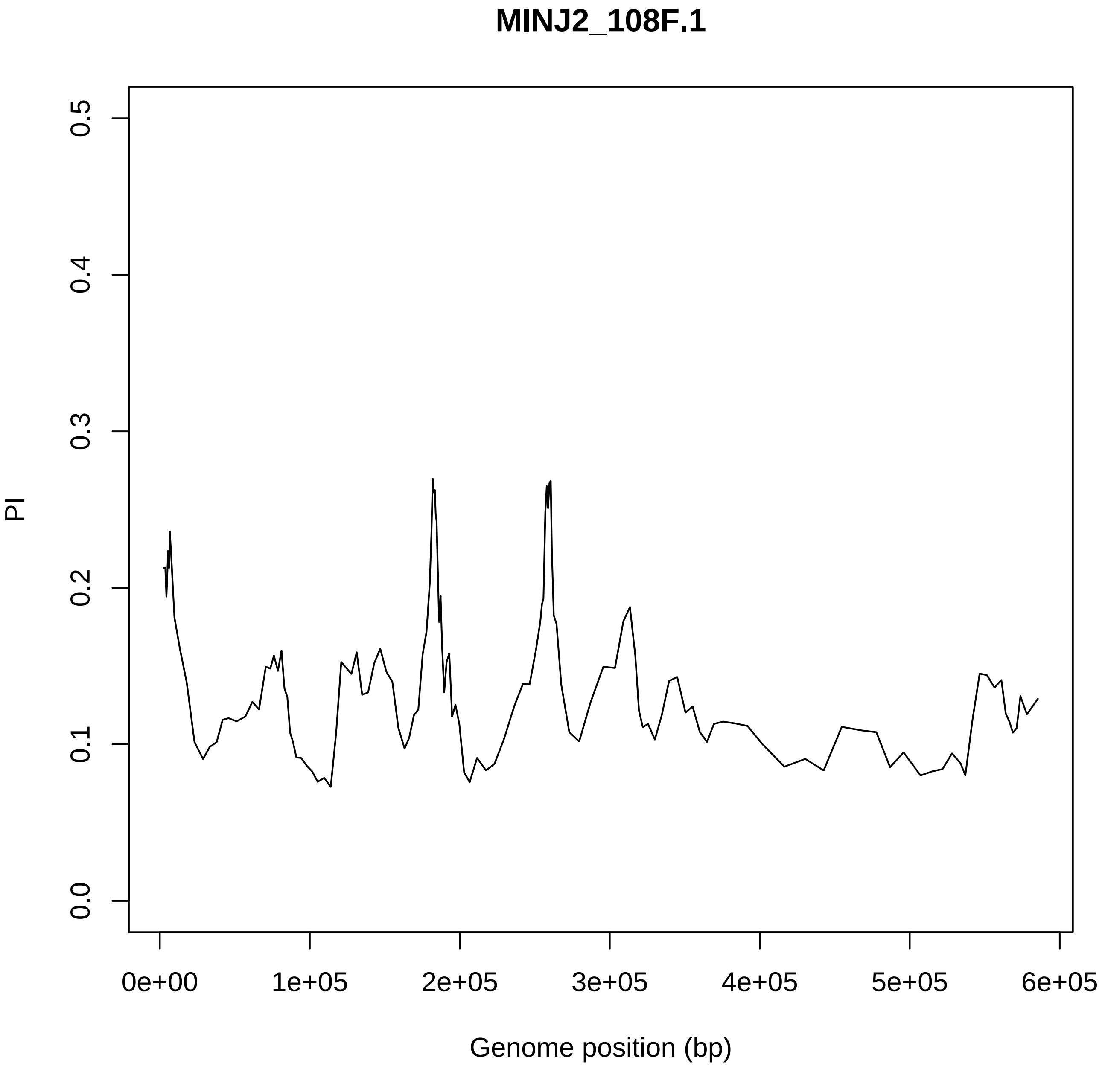
MINJ2_108F.1
PI
0.0
0.1
0.2
0.3
0.4
0.5
0e+00
1e+05
2e+05
3e+05
4e+05
5e+05
6e+05
Genome position (bp)

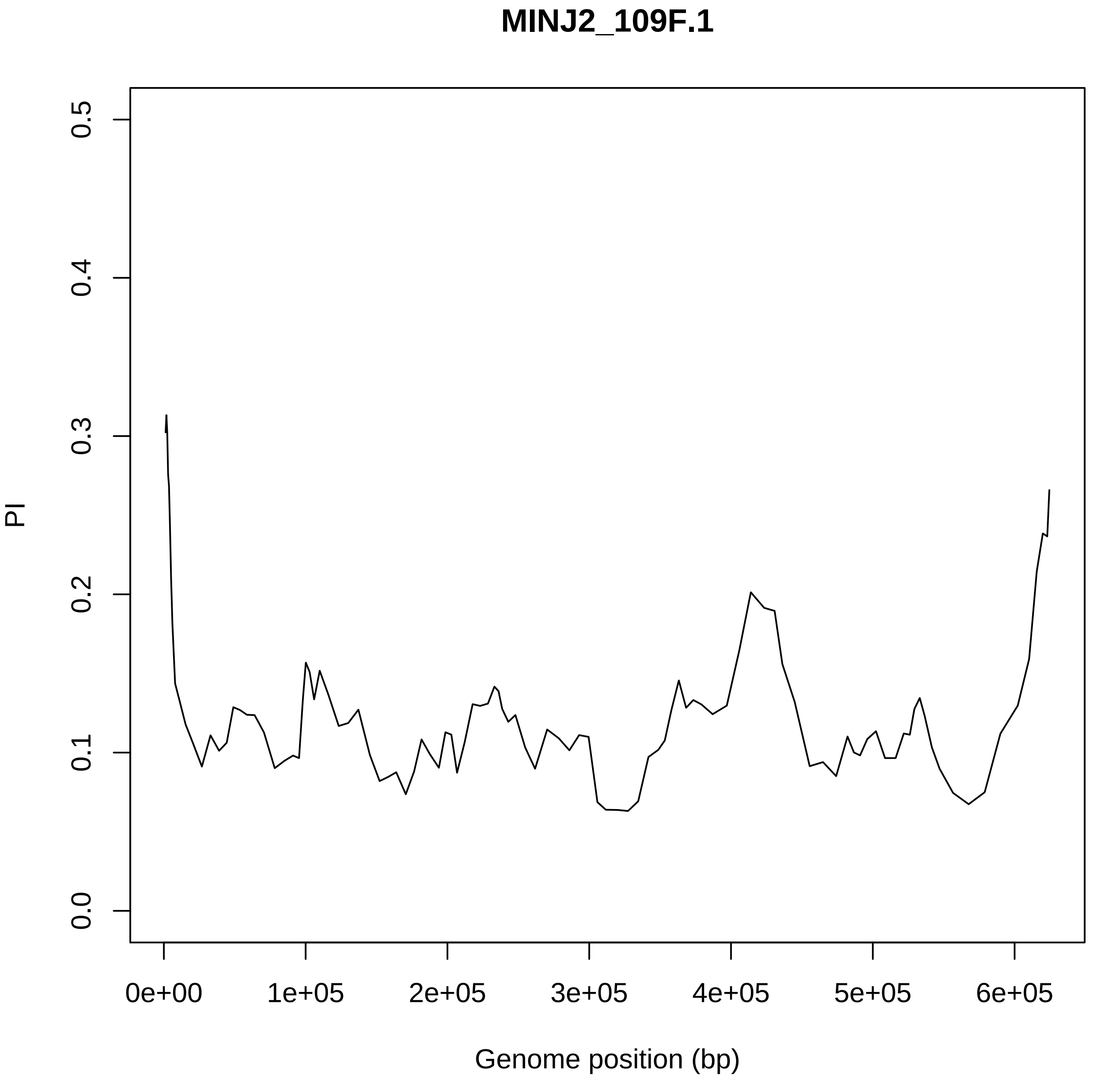
MINJ2_109F.1
PI
0.0
0.1
0.2
0.3
0.4
0.5
0e+00
1e+05
2e+05
3e+05
4e+05
5e+05
6e+05
Genome position (bp)

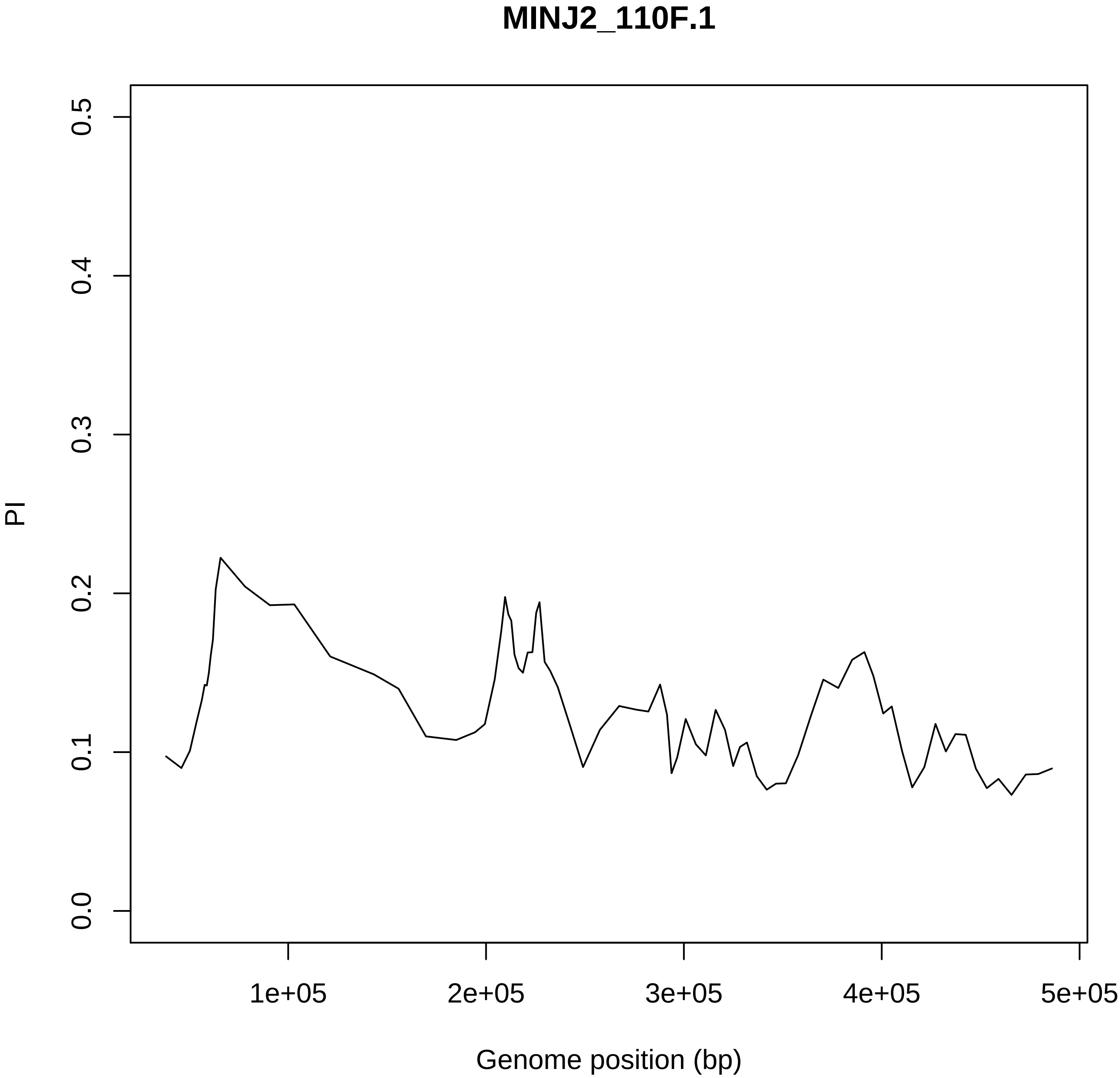
MINJ2_110F.1
PI
0.0
0.1
0.2
0.3
0.4
0.5
1e+05
2e+05
3e+05
4e+05
5e+05
Genome position (bp)

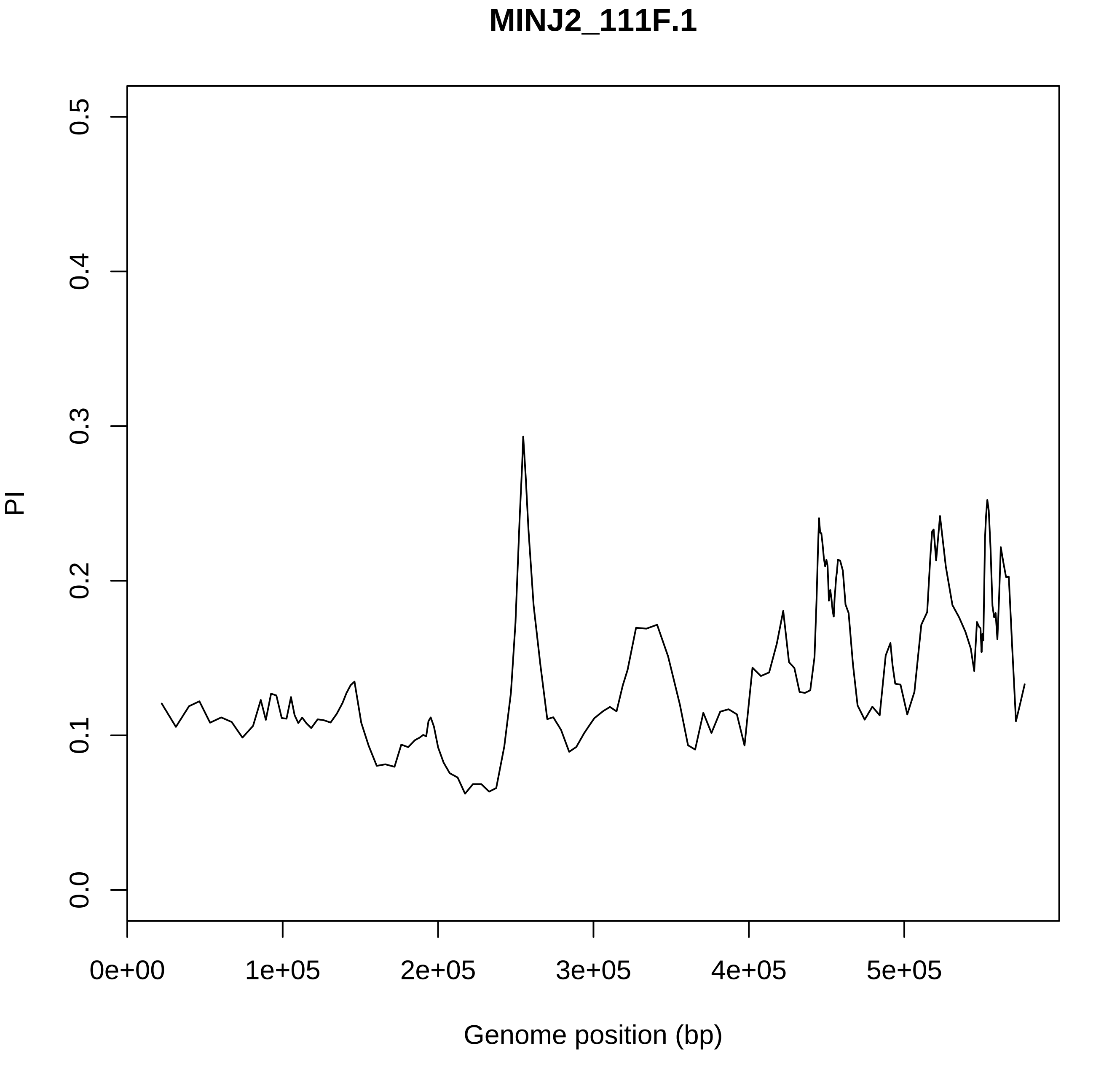
MINJ2_111F.1
PI
0.0
0.1
0.2
0.3
0.4
0.5
0e+00
1e+05
2e+05
3e+05
4e+05
5e+05
Genome position (bp)

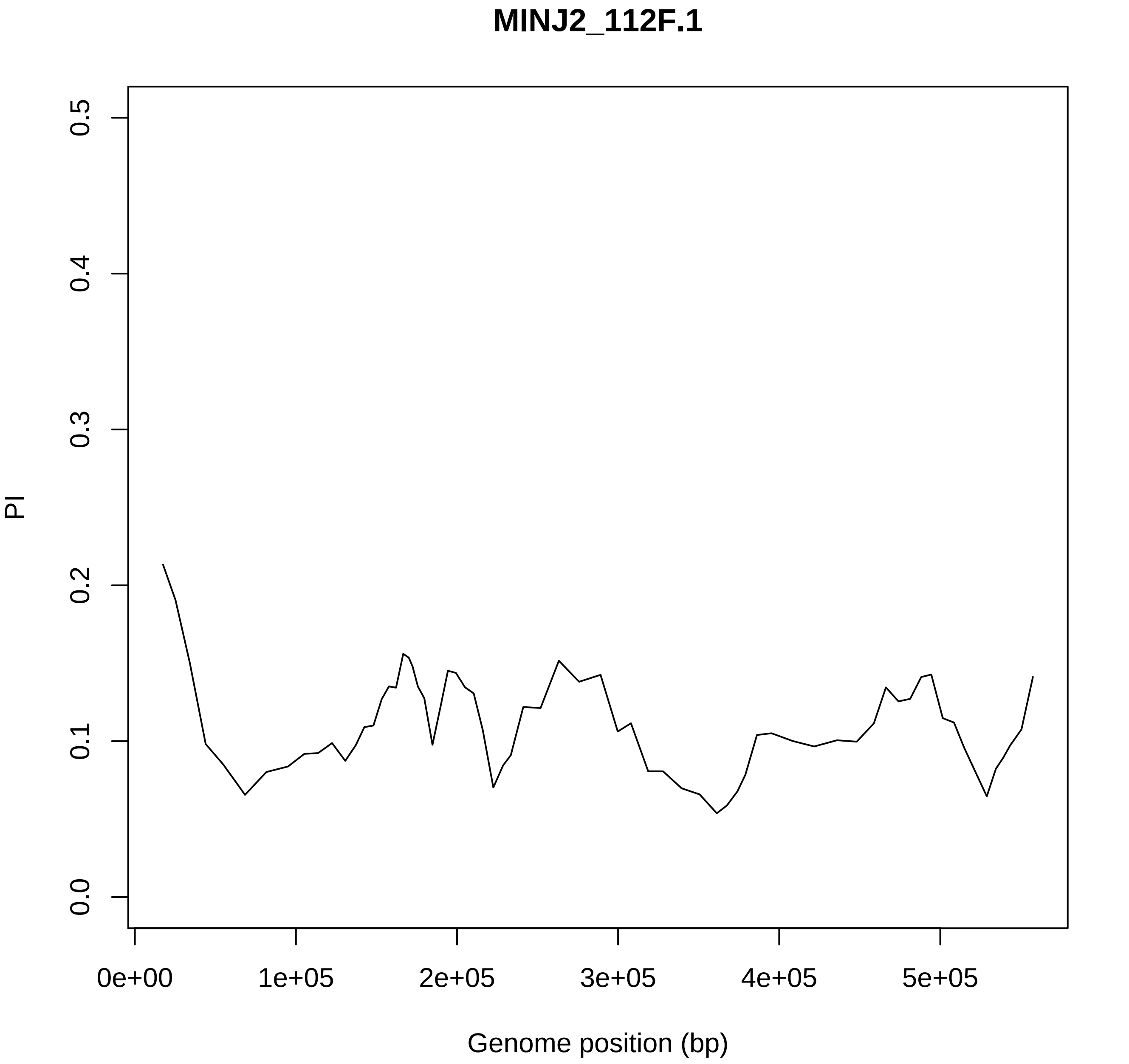
MINJ2_112F.1
PI
0.0
0.1
0.2
0.3
0.4
0.5
0e+00
1e+05
2e+05
3e+05
4e+05
5e+05
Genome position (bp)

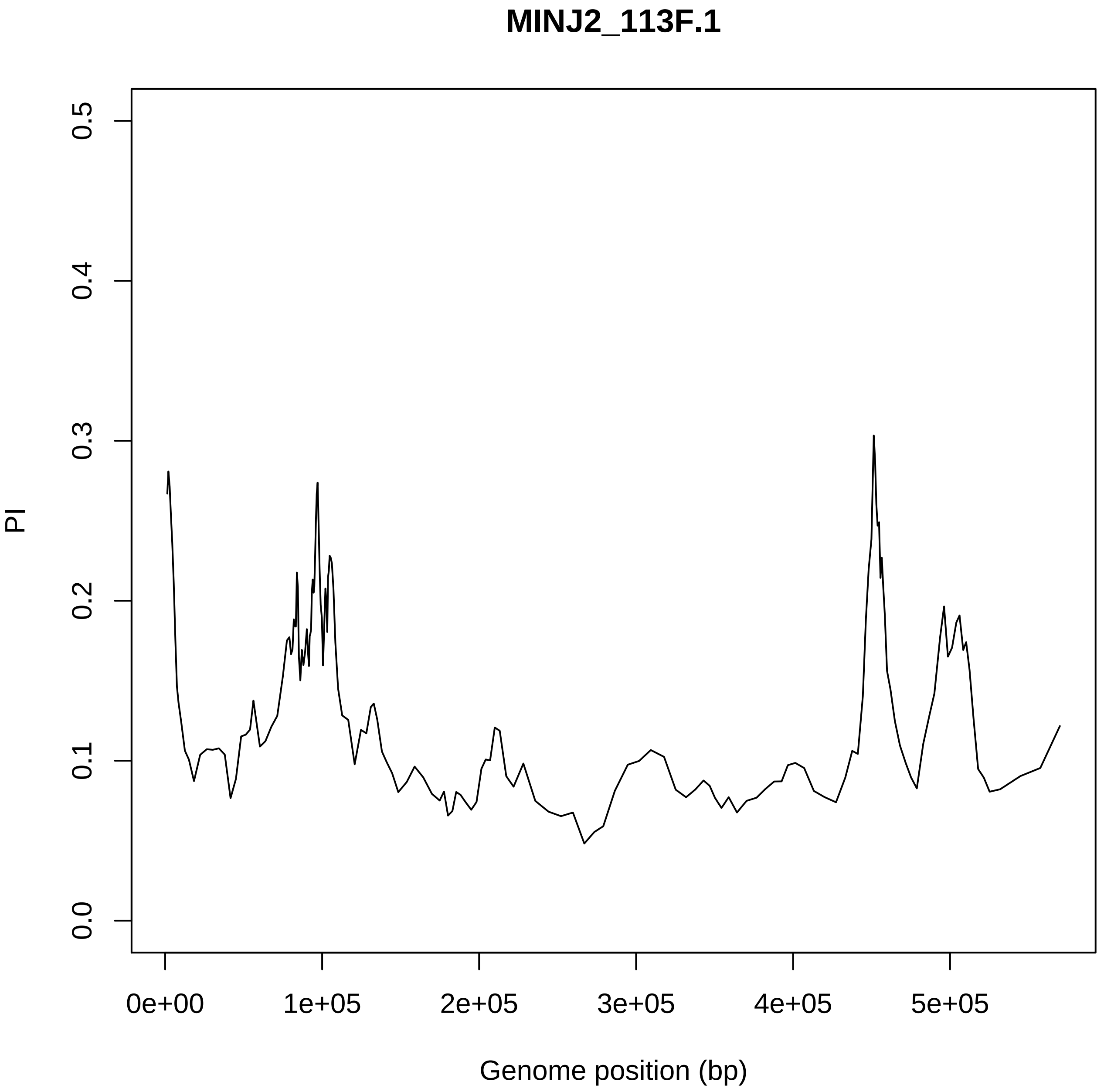

MINJ2_113F.1
PI
0.0
0.1
0.2
0.3
0.4
0.5
0e+00
1e+05
2e+05
3e+05
4e+05
5e+05
Genome position (bp)

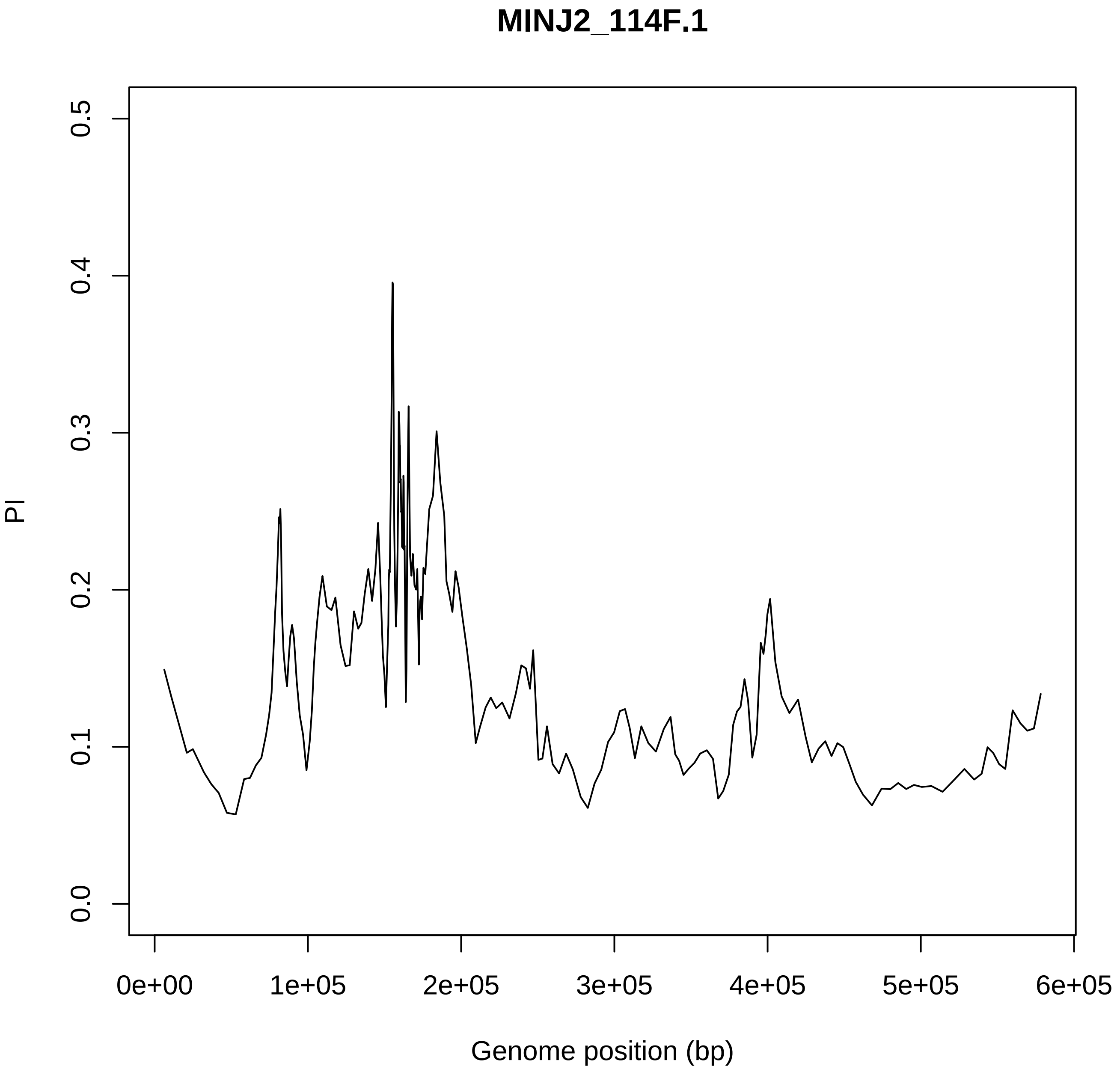
MINJ2_114F.1
PI
0.0
0.1
0.2
0.3
0.4
0.5
0e+00
1e+05
2e+05
3e+05
4e+05
5e+05
6e+05
Genome position (bp)

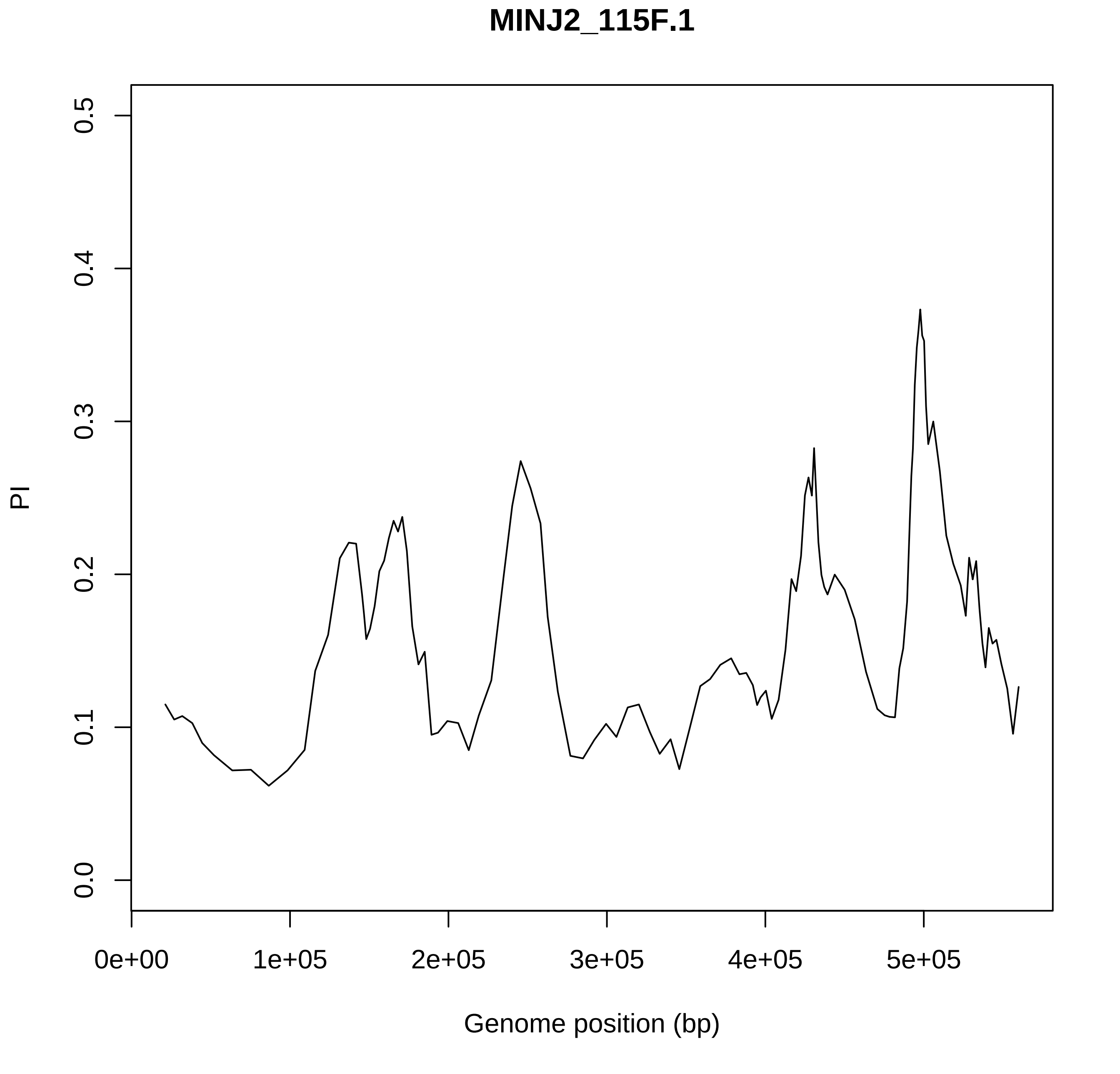
MINJ2_115F.1
PI
0.0
0.1
0.2
0.3
0.4
0.5
0e+00
1e+05
2e+05
3e+05
4e+05
5e+05
Genome position (bp)

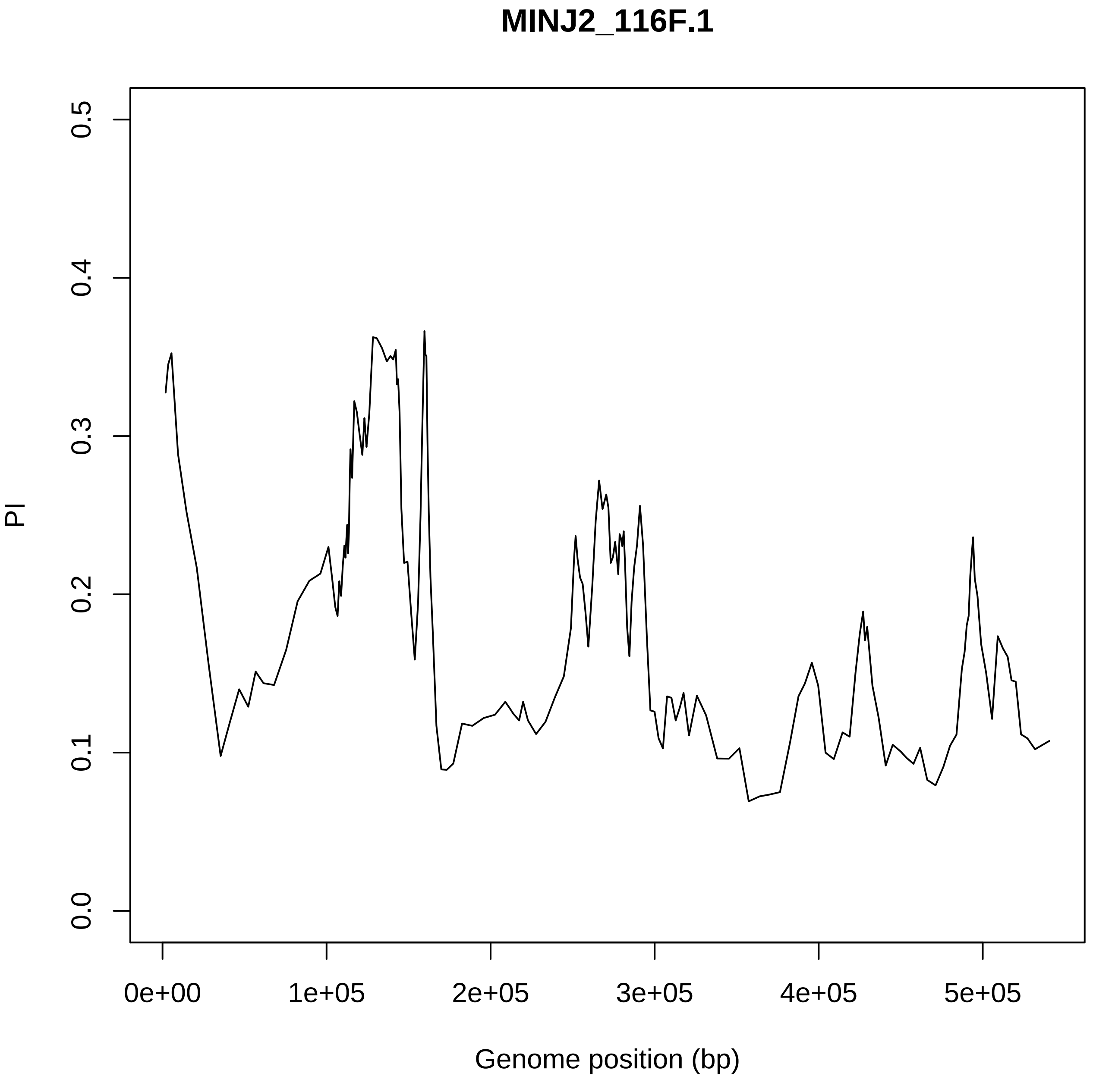
MINJ2_116F.1
PI
0.0
0.1
0.2
0.3
0.4
0.5
0e+00
1e+05
2e+05
3e+05
4e+05
5e+05
Genome position (bp)

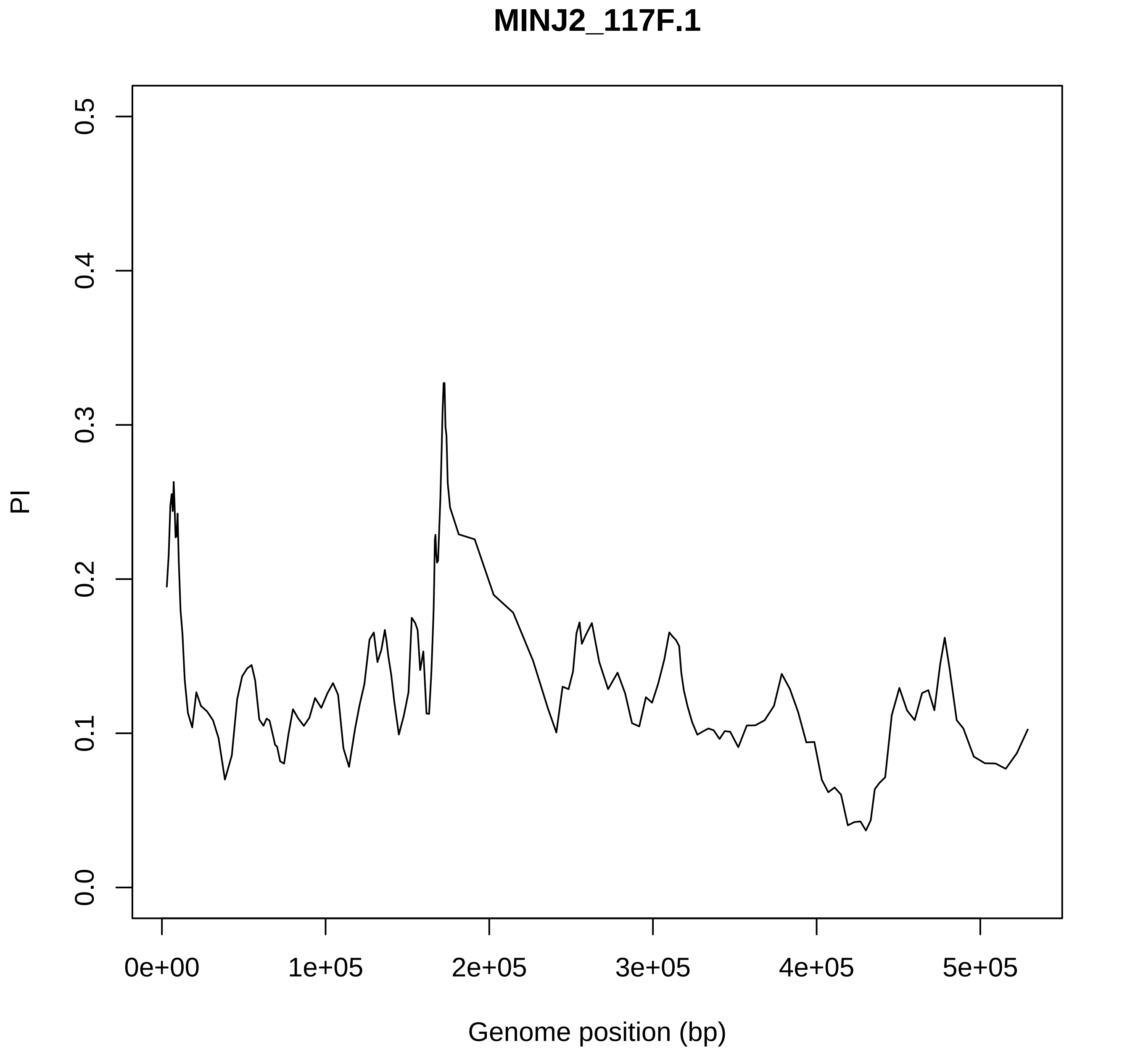
MINJ2_117F.1
PI
0.0
0.1
0.2
0.3
0.4
0.5
0e+00
1e+05
2e+05
3e+05
4e+05
5e+05
Genome position (bp)

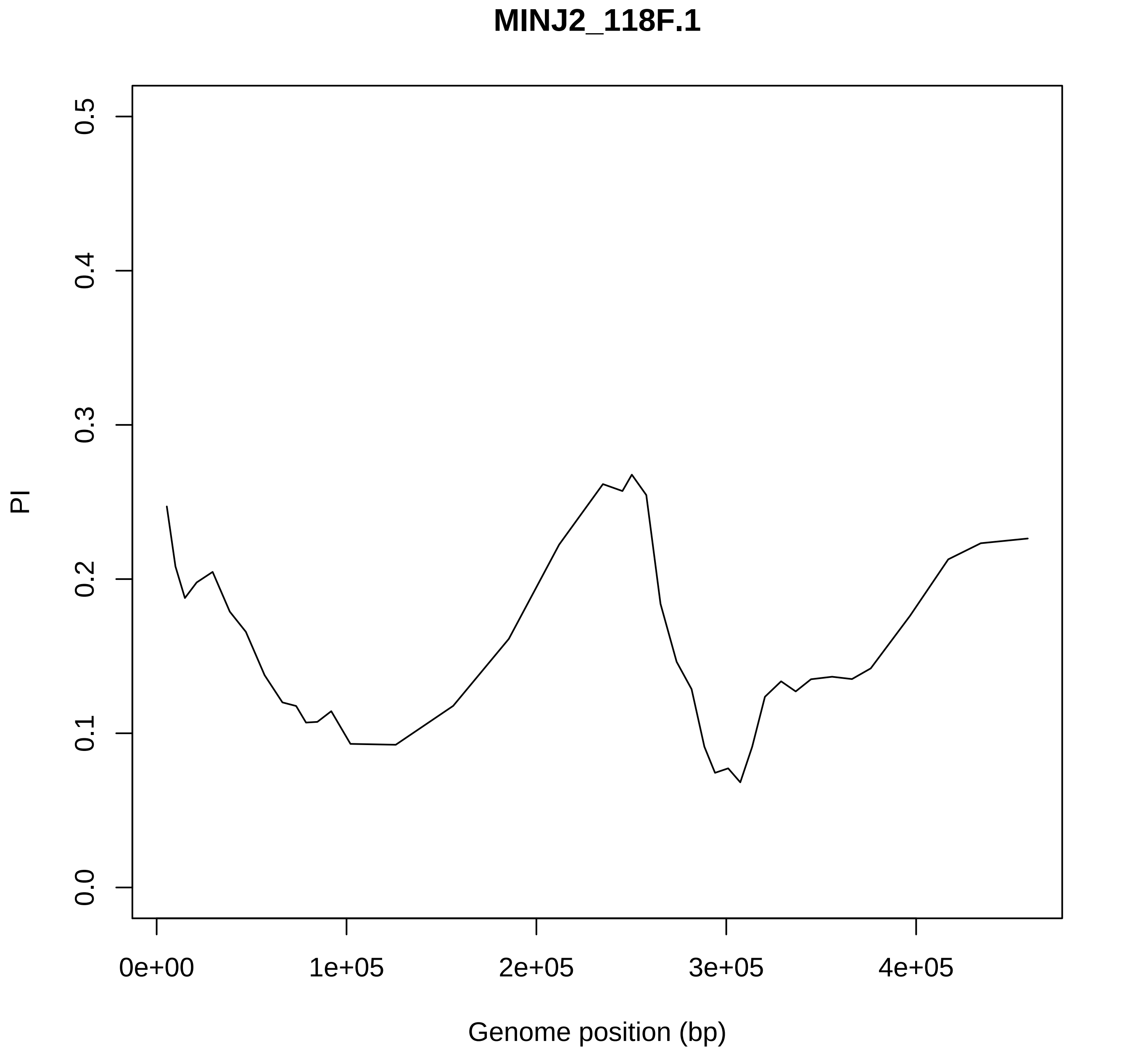

MINJ2_118F.1
PI
0.0
0.1
0.2
0.3
0.4
0.5
0e+00
1e+05
2e+05
3e+05
4e+05
Genome position (bp)

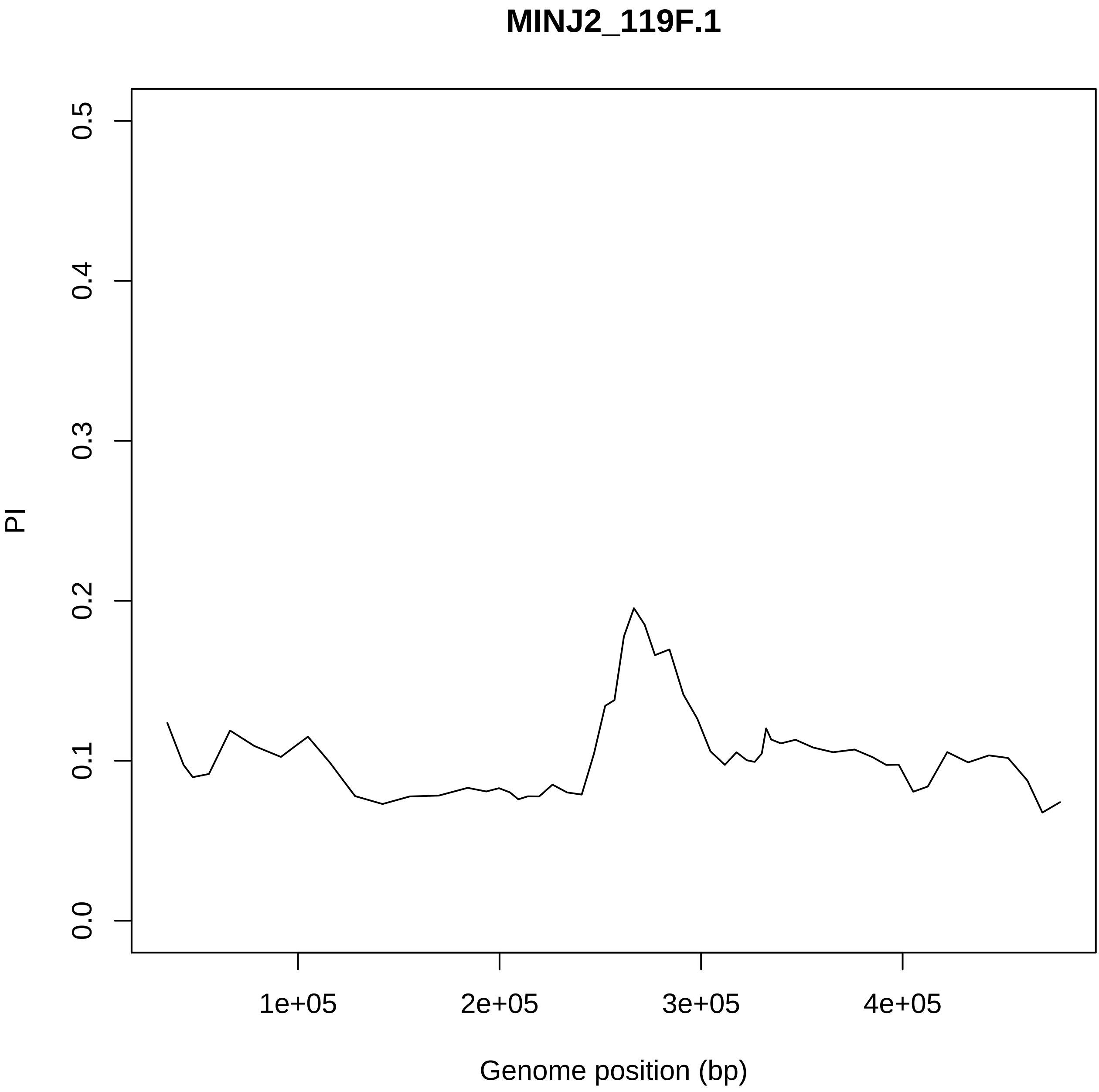
MINJ2_119F.1
PI
0.0
0.1
0.2
0.3
0.4
0.5
1e+05
2e+05
3e+05
4e+05
Genome position (bp)

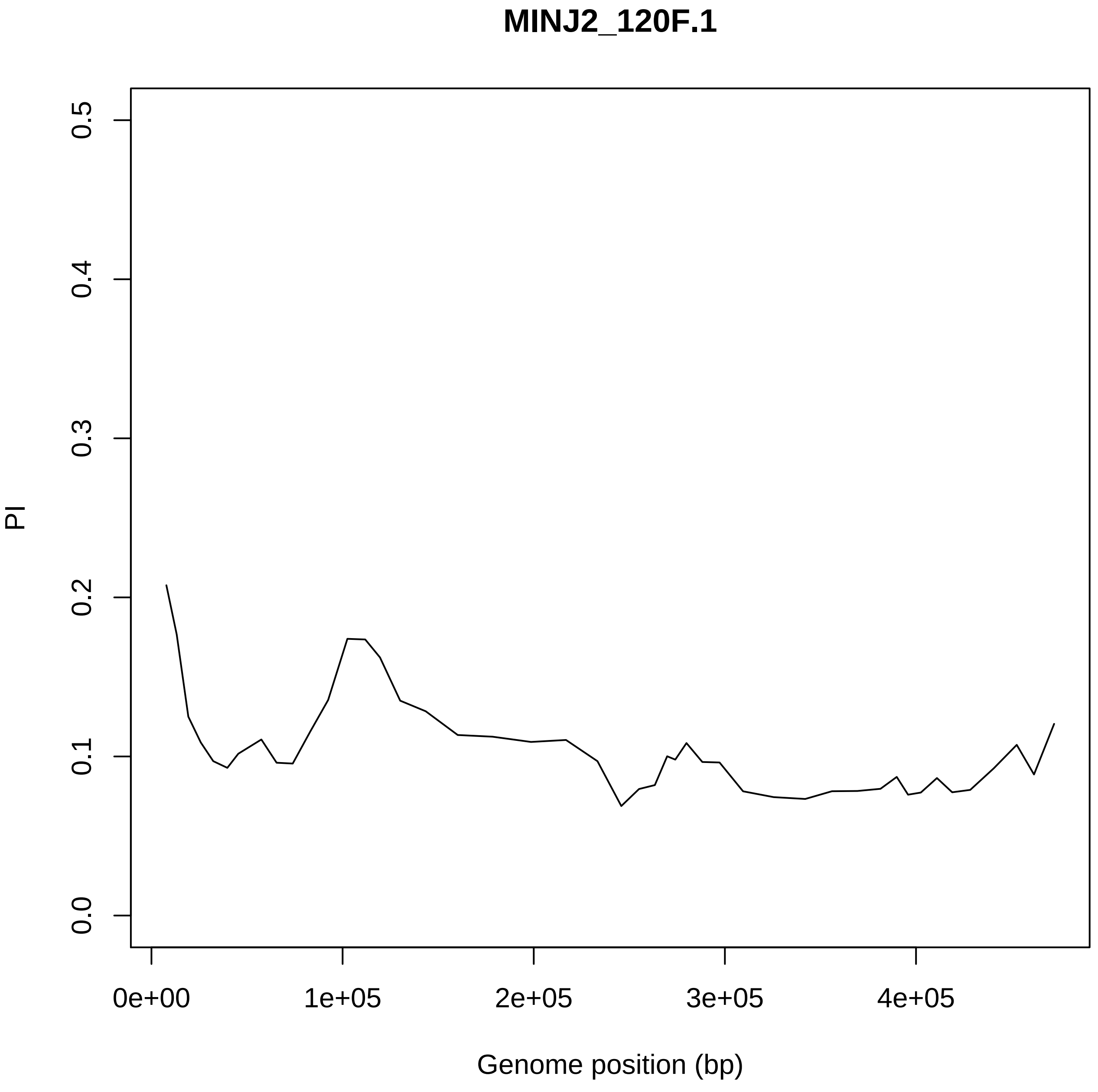

MINJ2_120F.1
0.0
0.1
0.2
0.3
0.4
0.5
PI
0e+00
1e+05
2e+05
3e+05
4e+05
Genome position (bp)

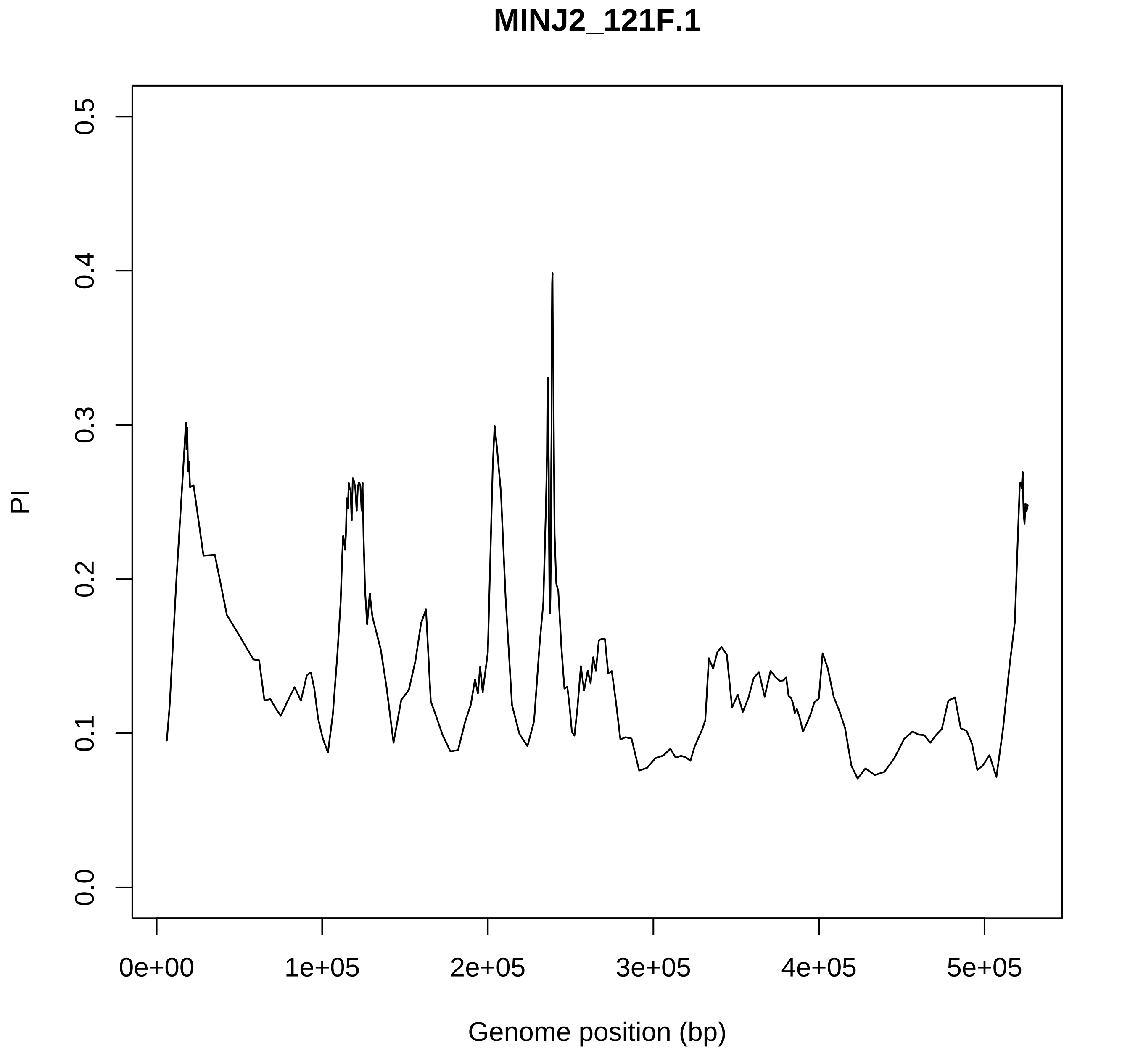
MINJ2_121F.1
PI
0.0
0.1
0.2
0.3
0.4
0.5
0e+00
1e+05
2e+05
3e+05
4e+05
5e+05
Genome position (bp)

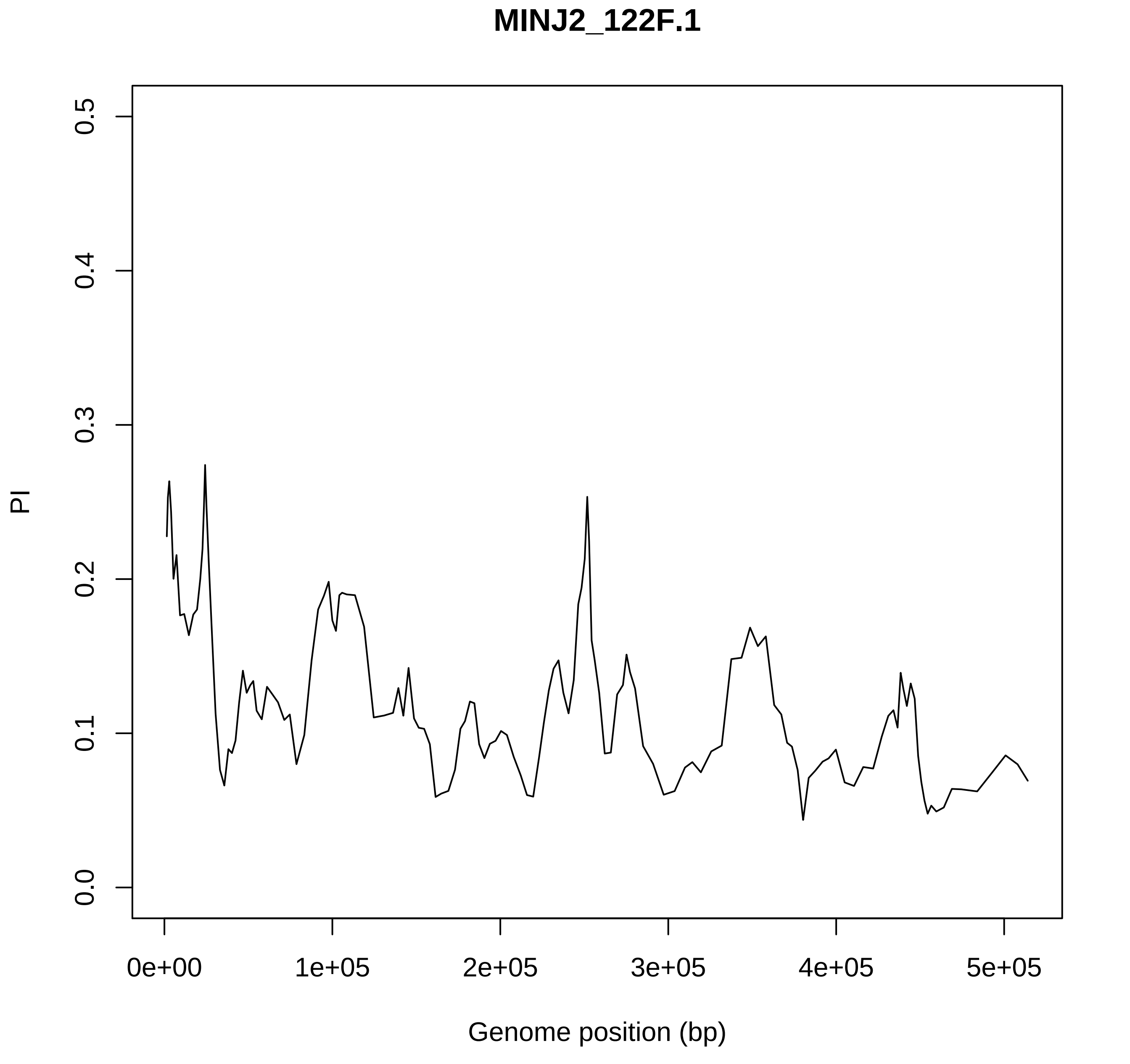

MINJ2_122F.1
PI
0.0
0.1
0.2
0.3
0.4
0.5
0e+00
1e+05
2e+05
3e+05
4e+05
5e+05
Genome position (bp)

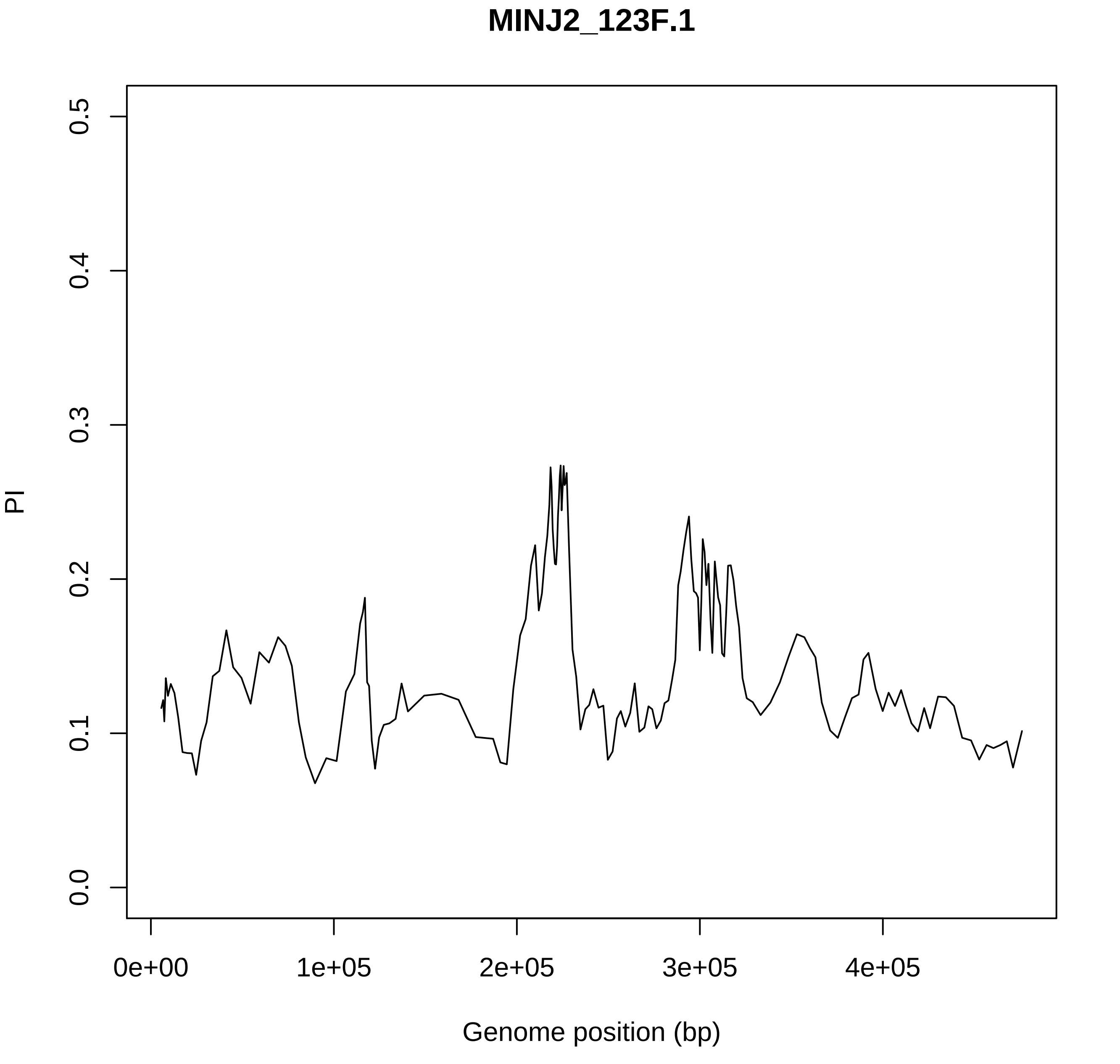

MINJ2_123F.1
PI
0.0
0.1
0.2
0.3
0.4
0.5
0e+00
1e+05
2e+05
3e+05
4e+05
Genome position (bp)

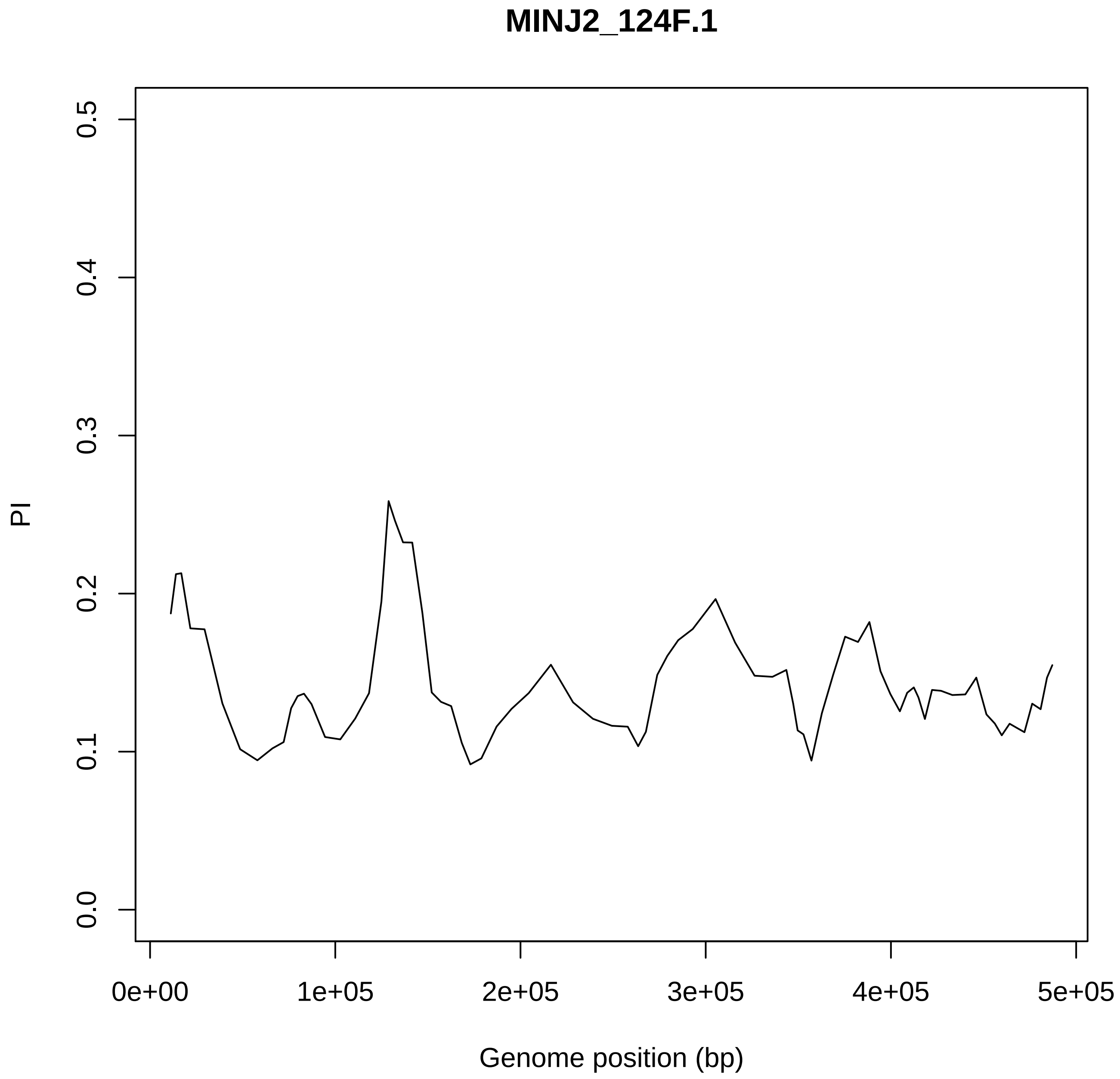

MINJ2_124F.1
PI
0.0
0.1
0.2
0.3
0.4
0.5
0e+00
1e+05
2e+05
3e+05
4e+05
5e+05
Genome position (bp)

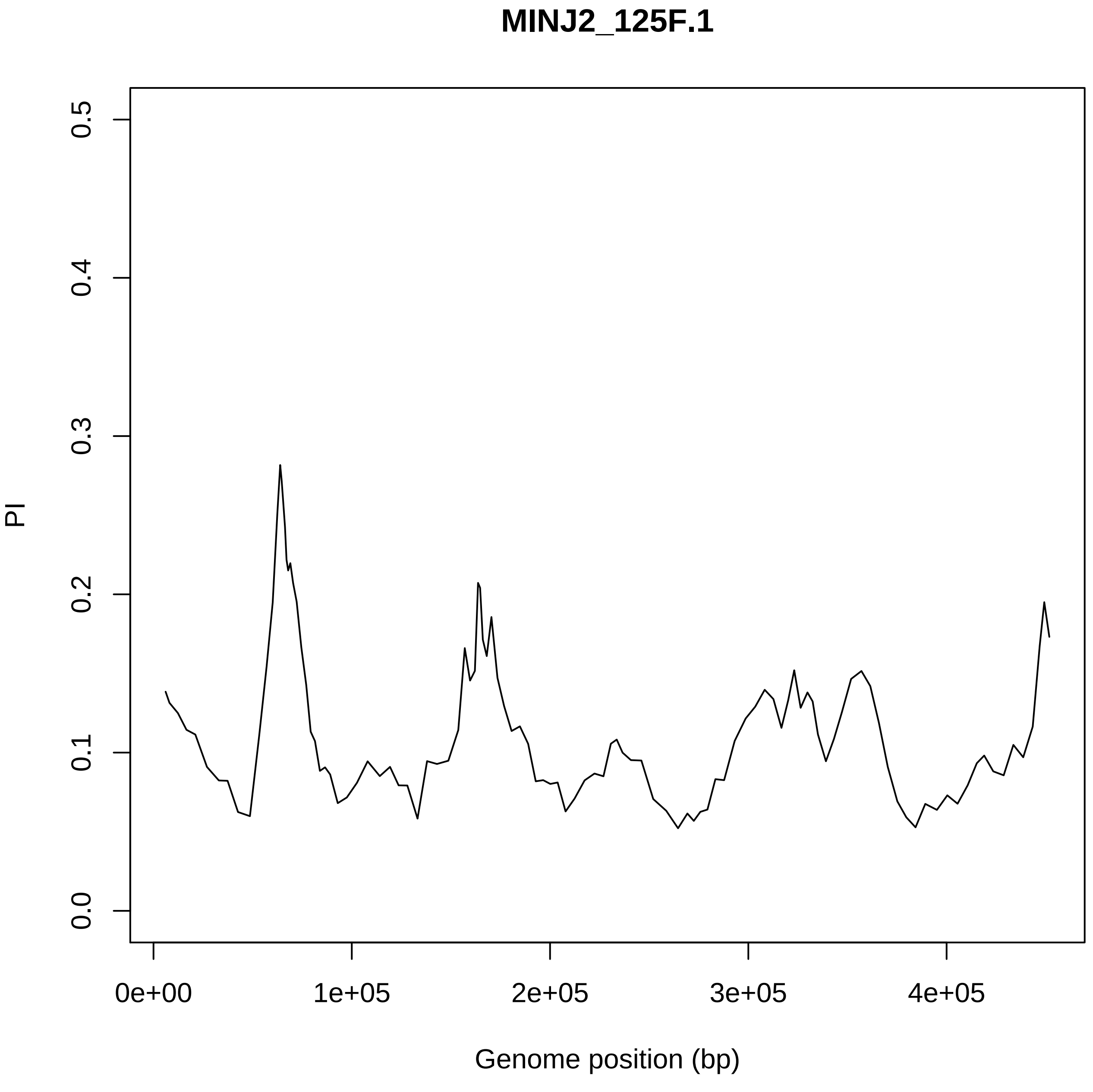
MINJ2_125F.1
PI
0.0
0.1
0.2
0.3
0.4
0.5
0e+00
1e+05
2e+05
3e+05
4e+05
Genome position (bp)

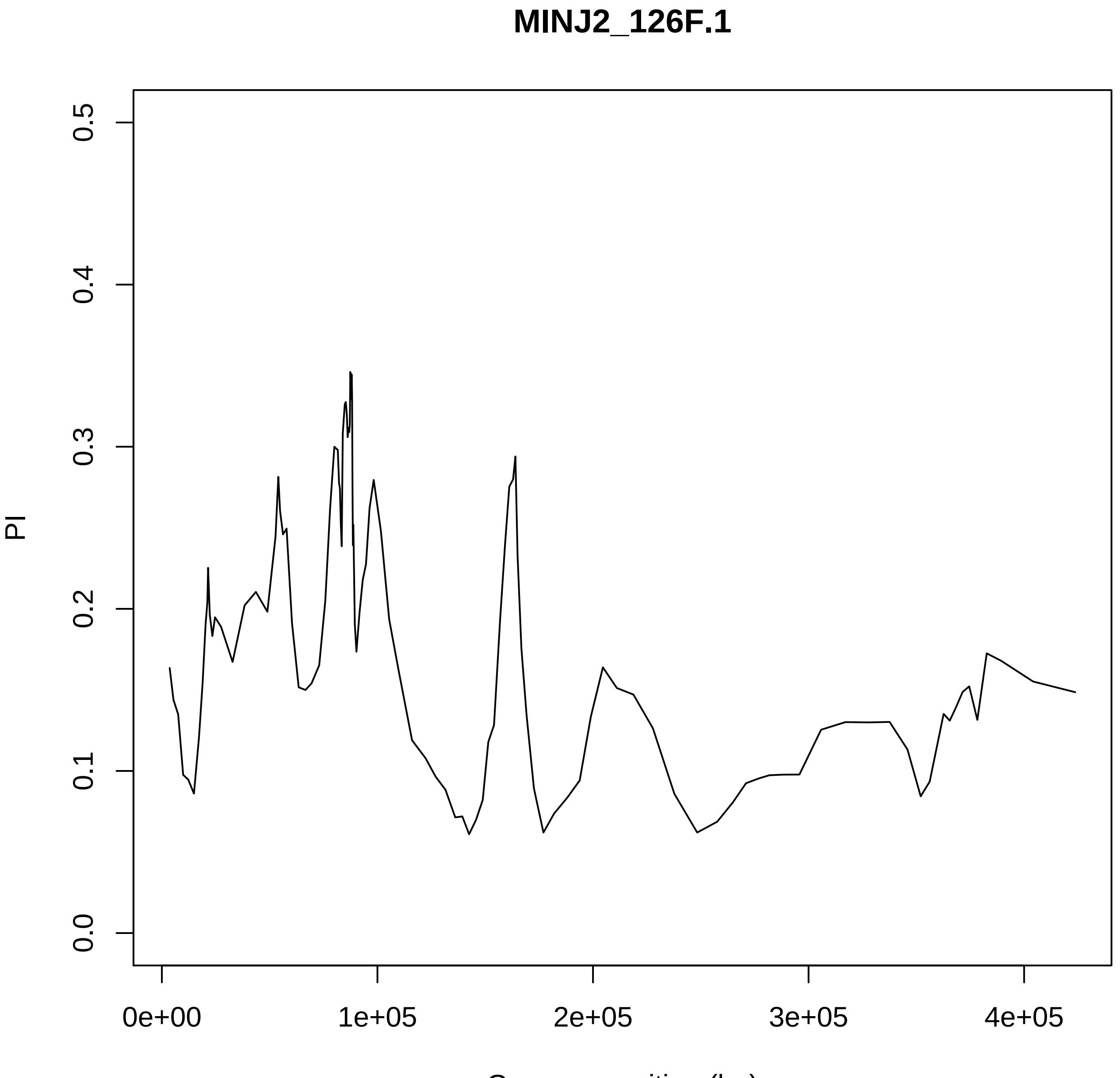
MINJ2_126F.1
PI
0.0
0.1
0.2
0.3
0.4
0.5
0e+00
1e+05
2e+05
3e+05
4e+05
Genome position (bp)

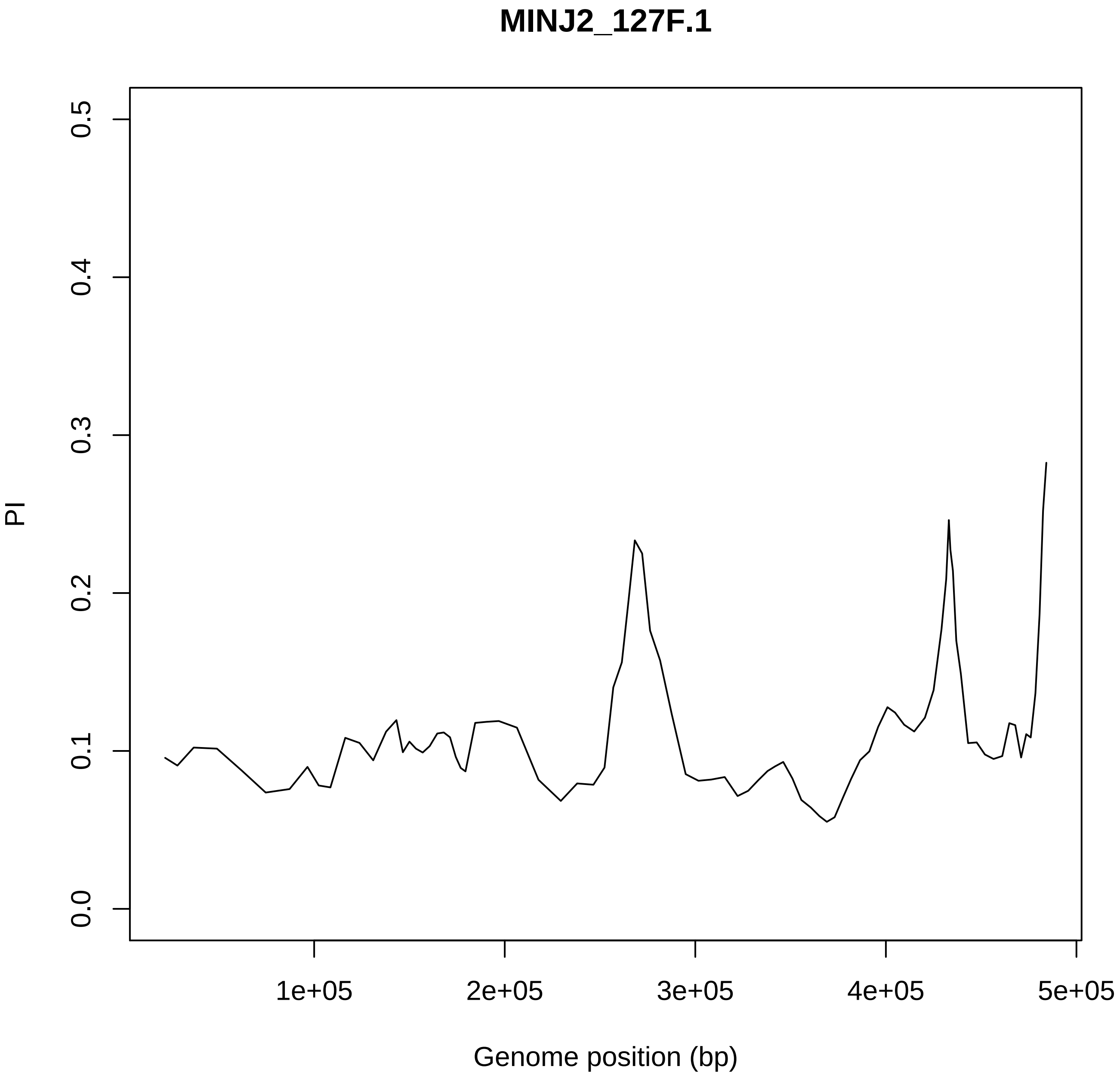
MINJ2_127F.1
PI
0.0
0.1
0.2
0.3
0.4
0.5
1e+05
2e+05
3e+05
4e+05
5e+05
Genome position (bp)

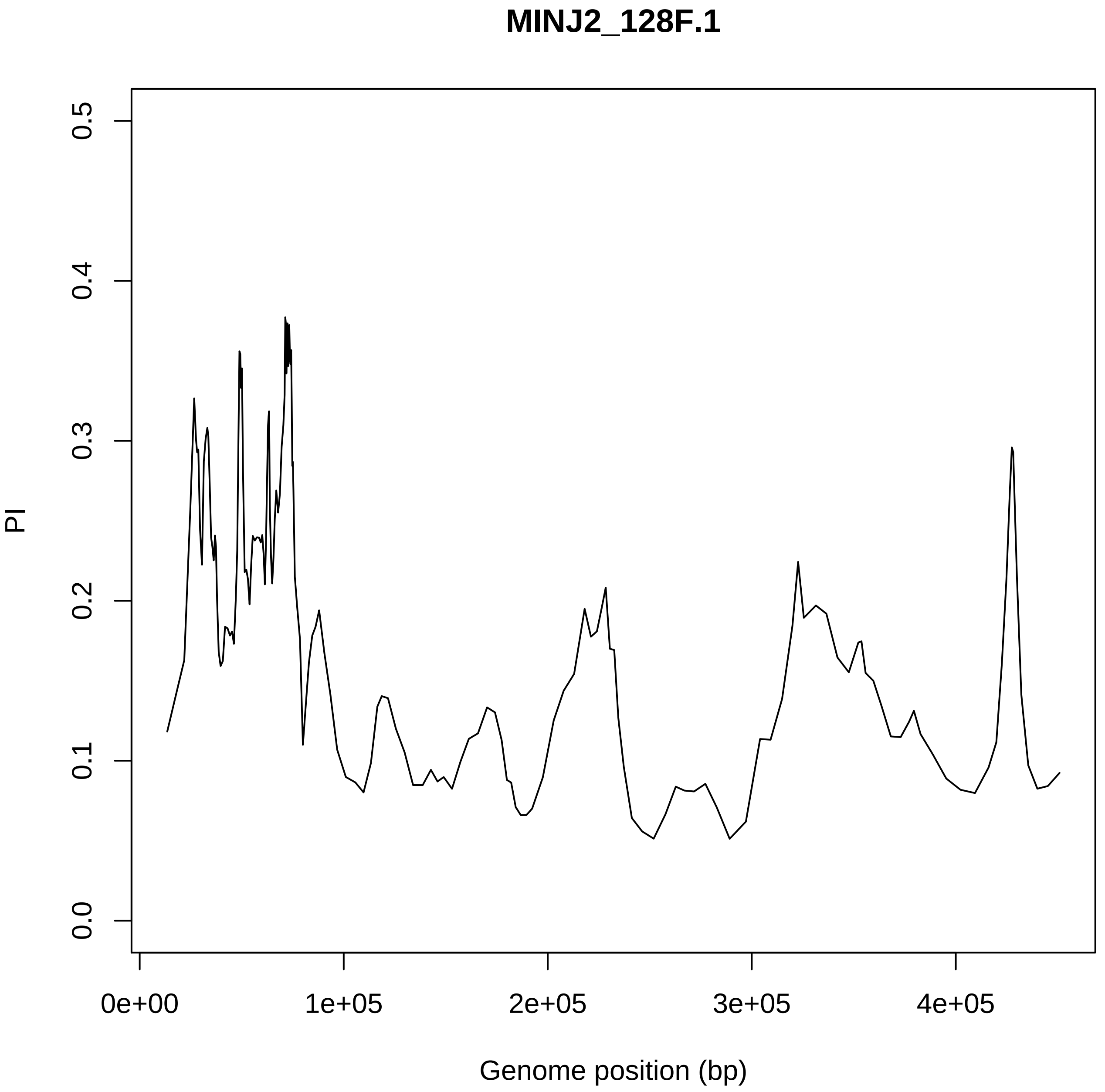

MINJ2_128F.1
PI
0.0
0.1
0.2
0.3
0.4
0.5
0e+00
1e+05
2e+05
3e+05
4e+05
Genome position (bp)

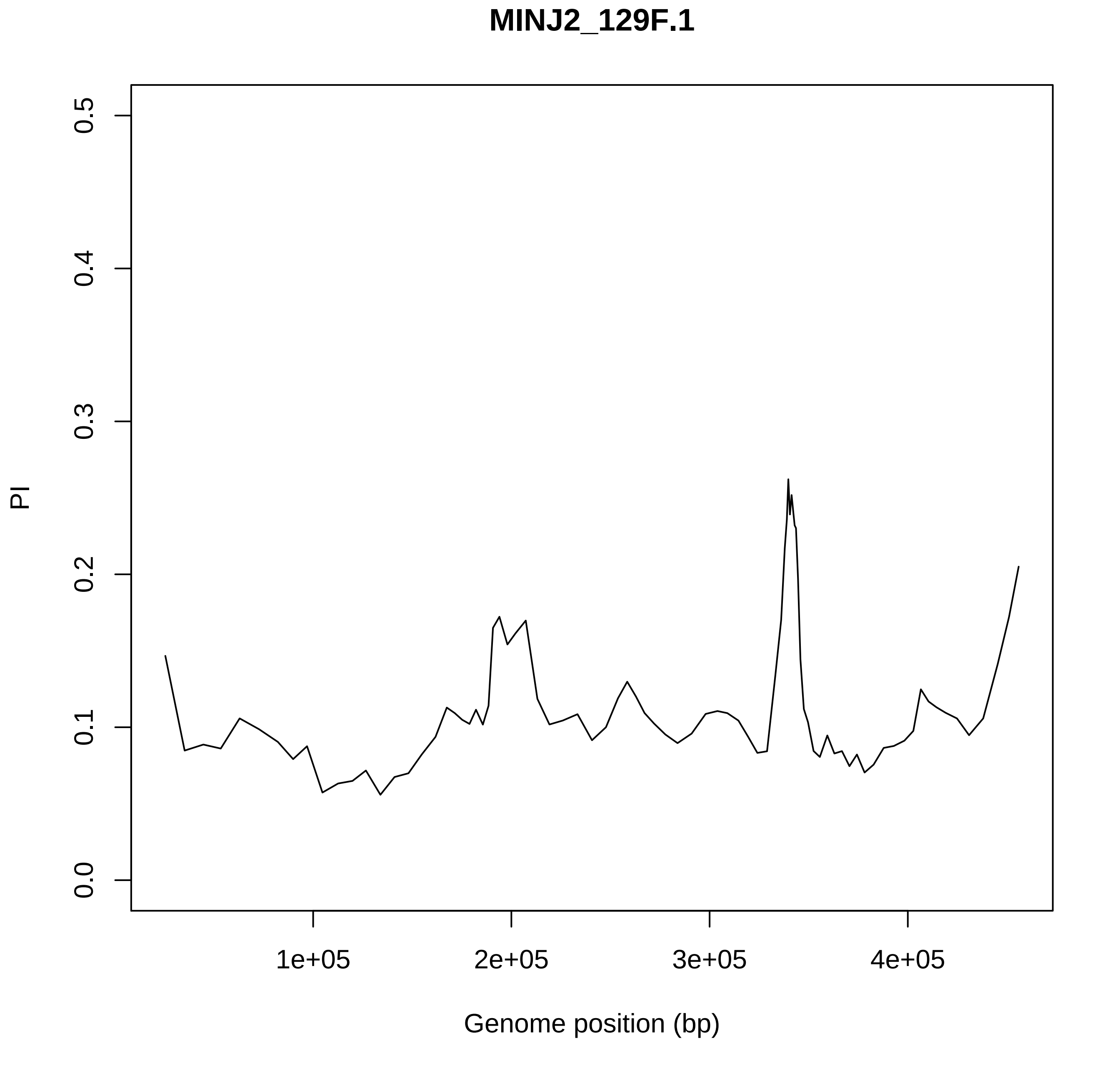

MINJ2_129F.1
PI
0.0
0.1
0.2
0.3
0.4
0.5
1e+05
2e+05
3e+05
4e+05
Genome position (bp)

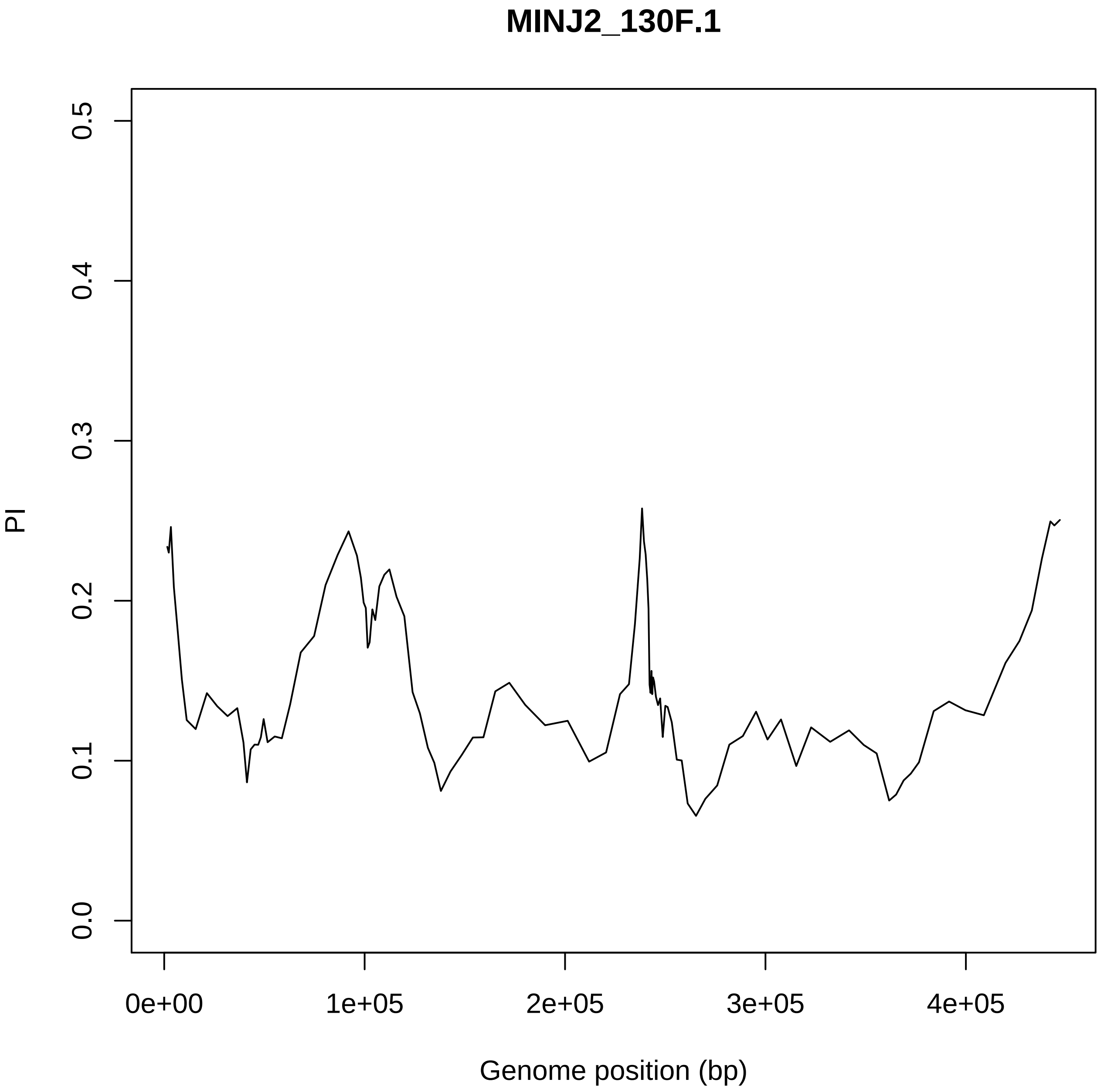

MINJ2_130F.1
PI
0.0
0.1
0.2
0.3
0.4
0.5
0e+00
1e+05
2e+05
3e+05
4e+05
Genome position (bp)

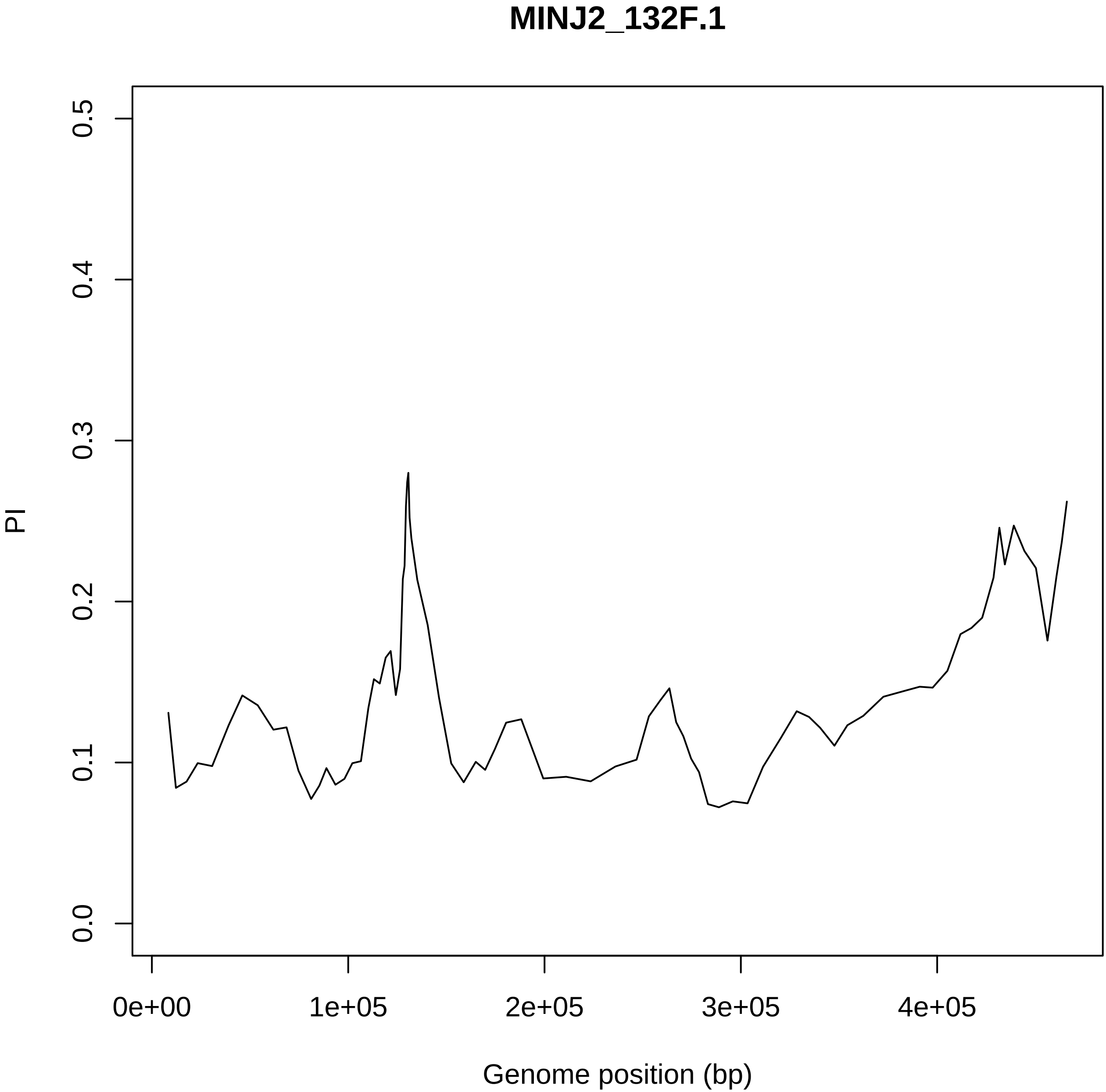
MINJ2_132F.1
PI
0.0
0.1
0.2
0.3
0.4
0.5
0e+00
1e+05
2e+05
3e+05
4e+05
Genome position (bp)

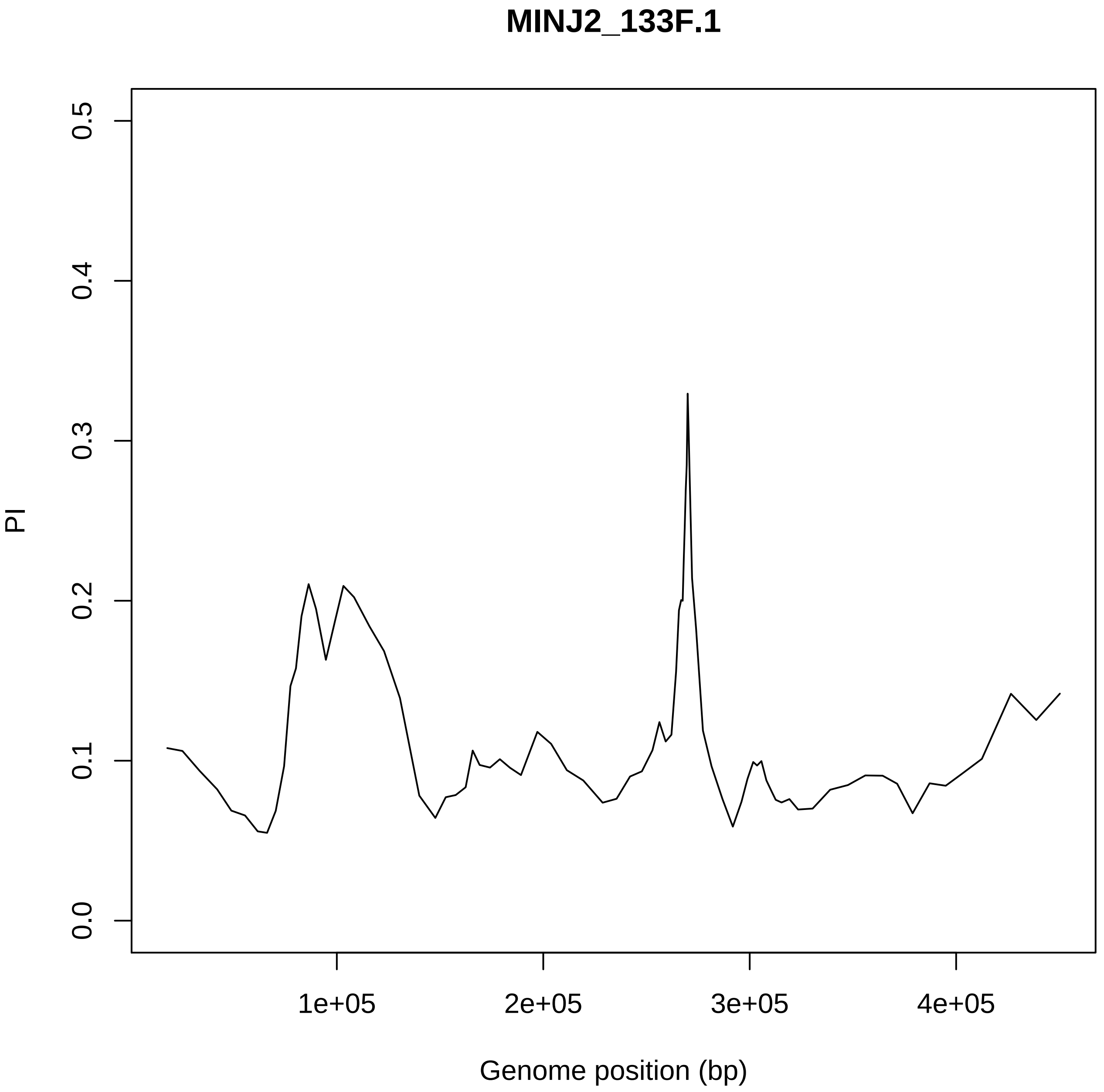
MINJ2_133F.1
PI
0.0
0.1
0.2
0.3
0.4
0.5
1e+05
2e+05
3e+05
4e+05
Genome position (bp)

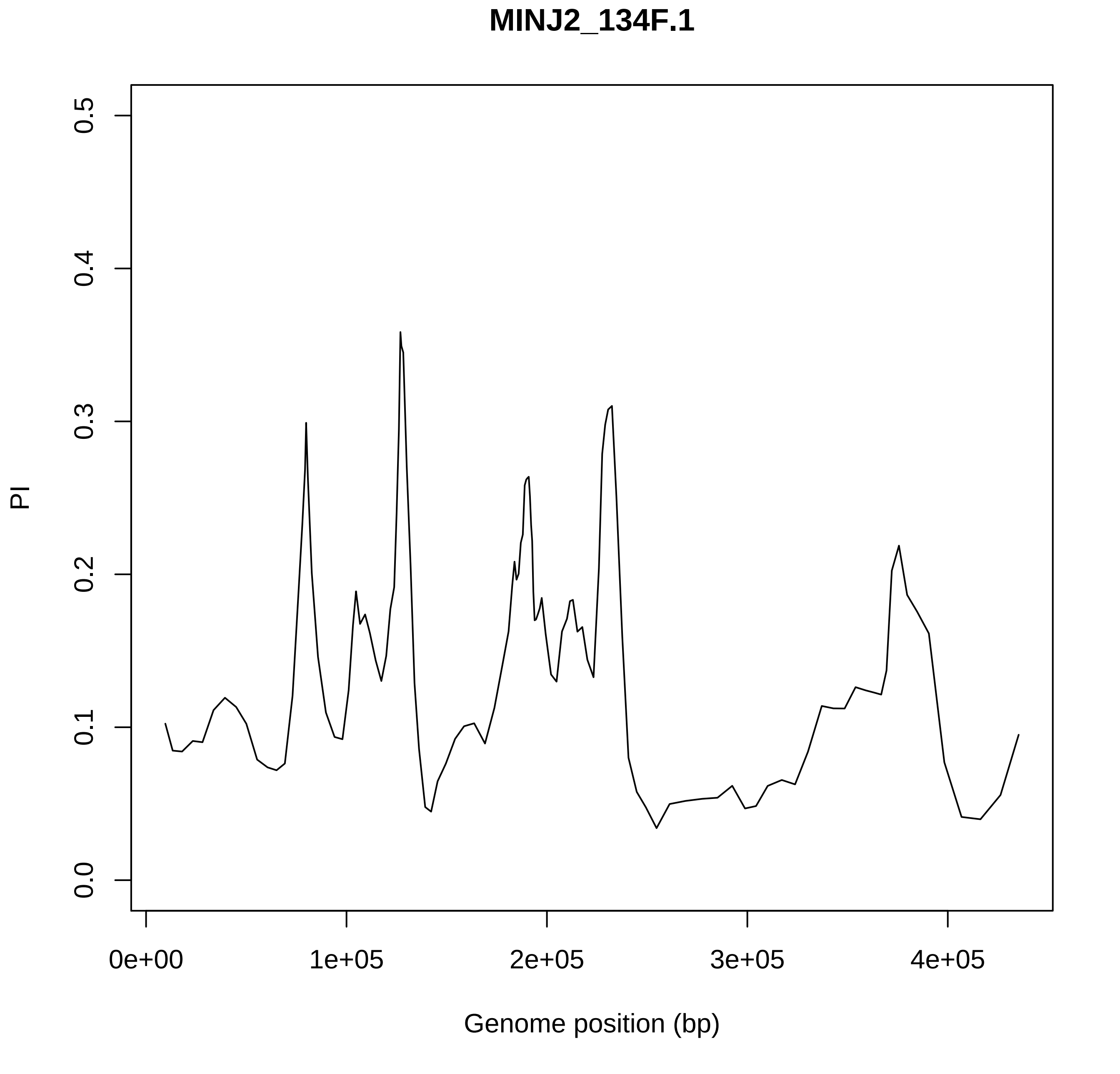

MINJ2_134F.1
PI
0.0
0.1
0.2
0.3
0.4
0.5
0e+00
1e+05
2e+05
3e+05
4e+05
Genome position (bp)

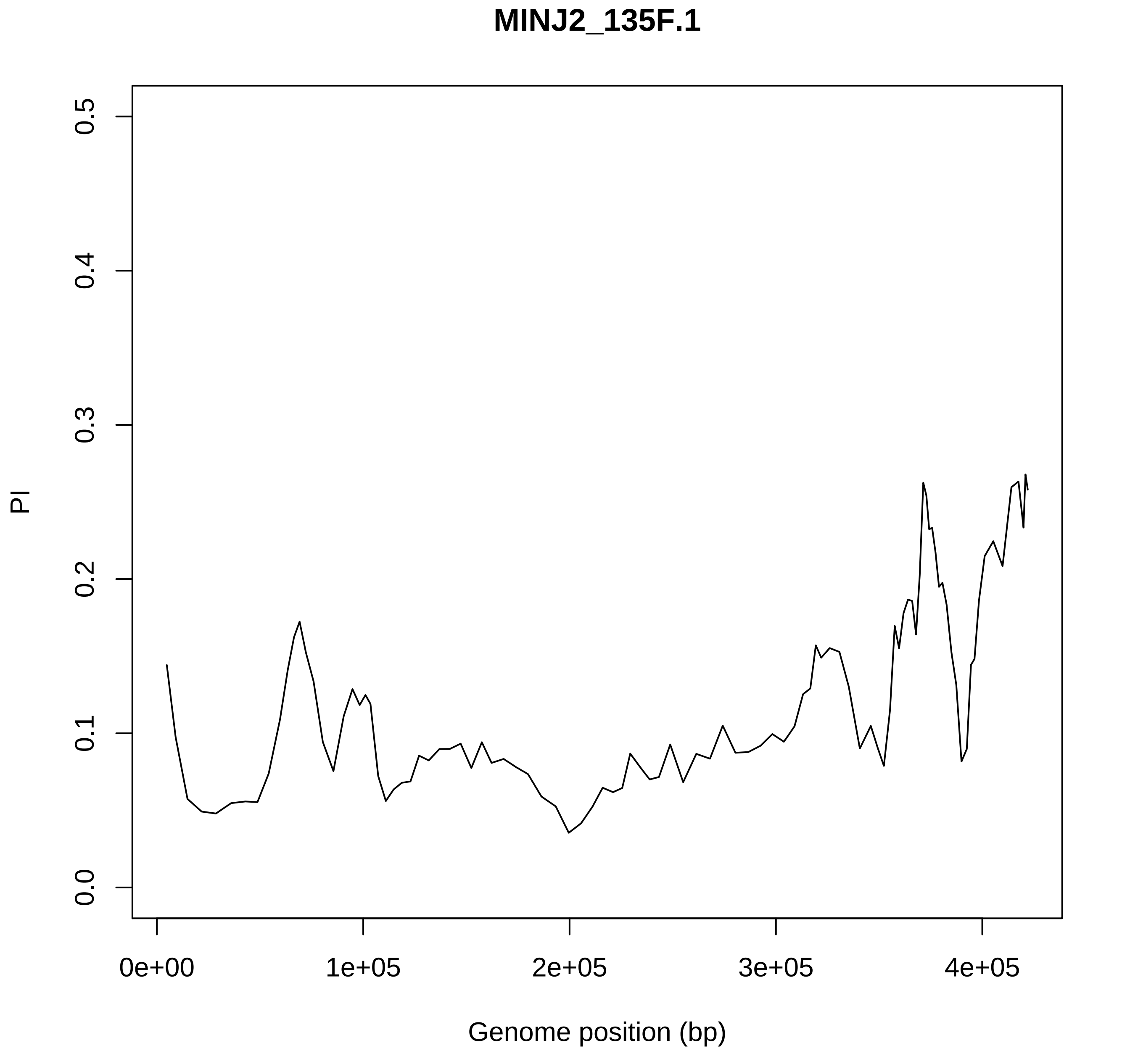

MINJ2_135F.1
PI
0.0
0.1
0.2
0.3
0.4
0.5
0e+00
1e+05
2e+05
3e+05
4e+05
Genome position (bp)

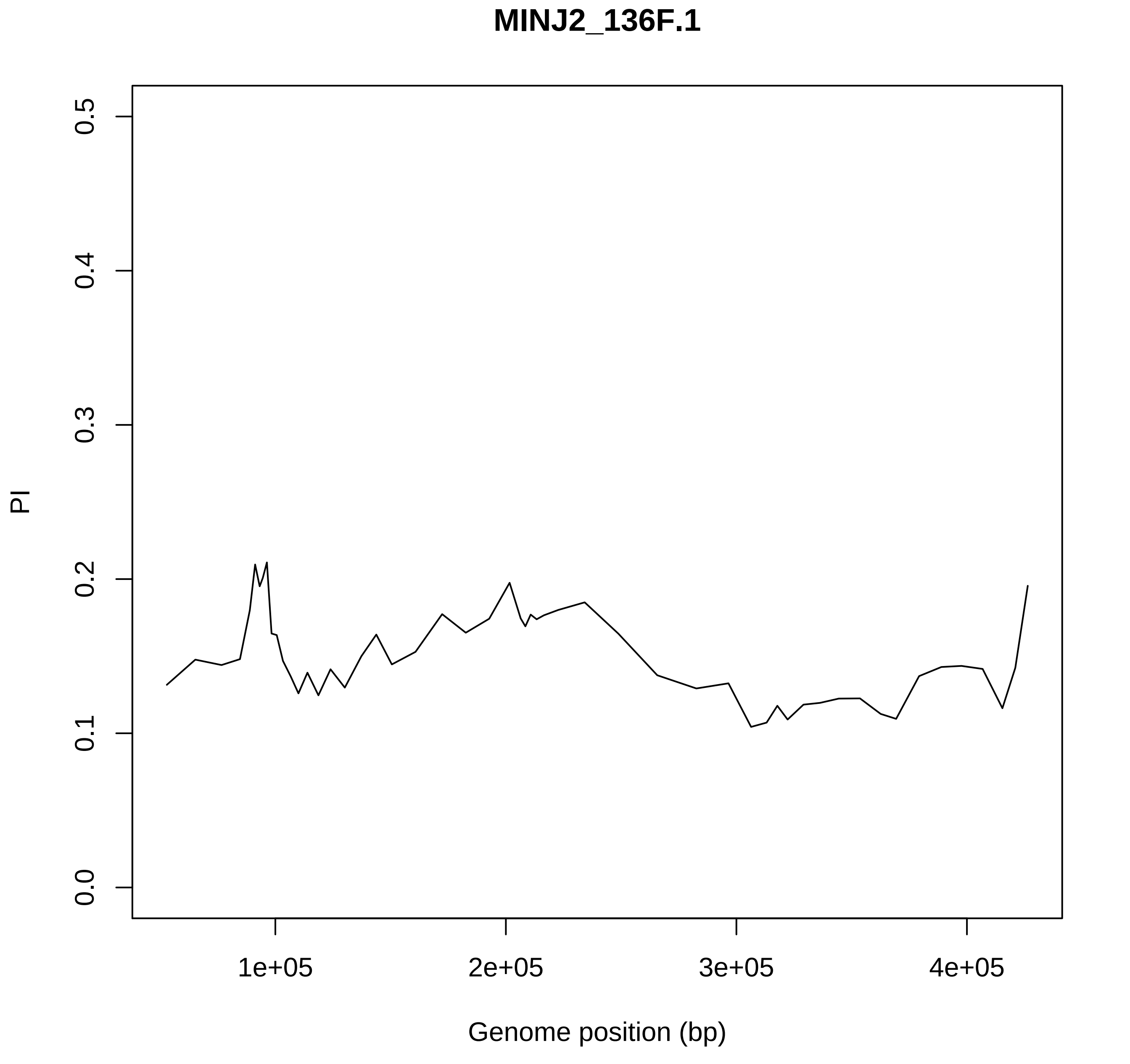

MINJ2_136F.1
PI
0.0
0.1
0.2
0.3
0.4
0.5
1e+05
2e+05
3e+05
4e+05
Genome position (bp)

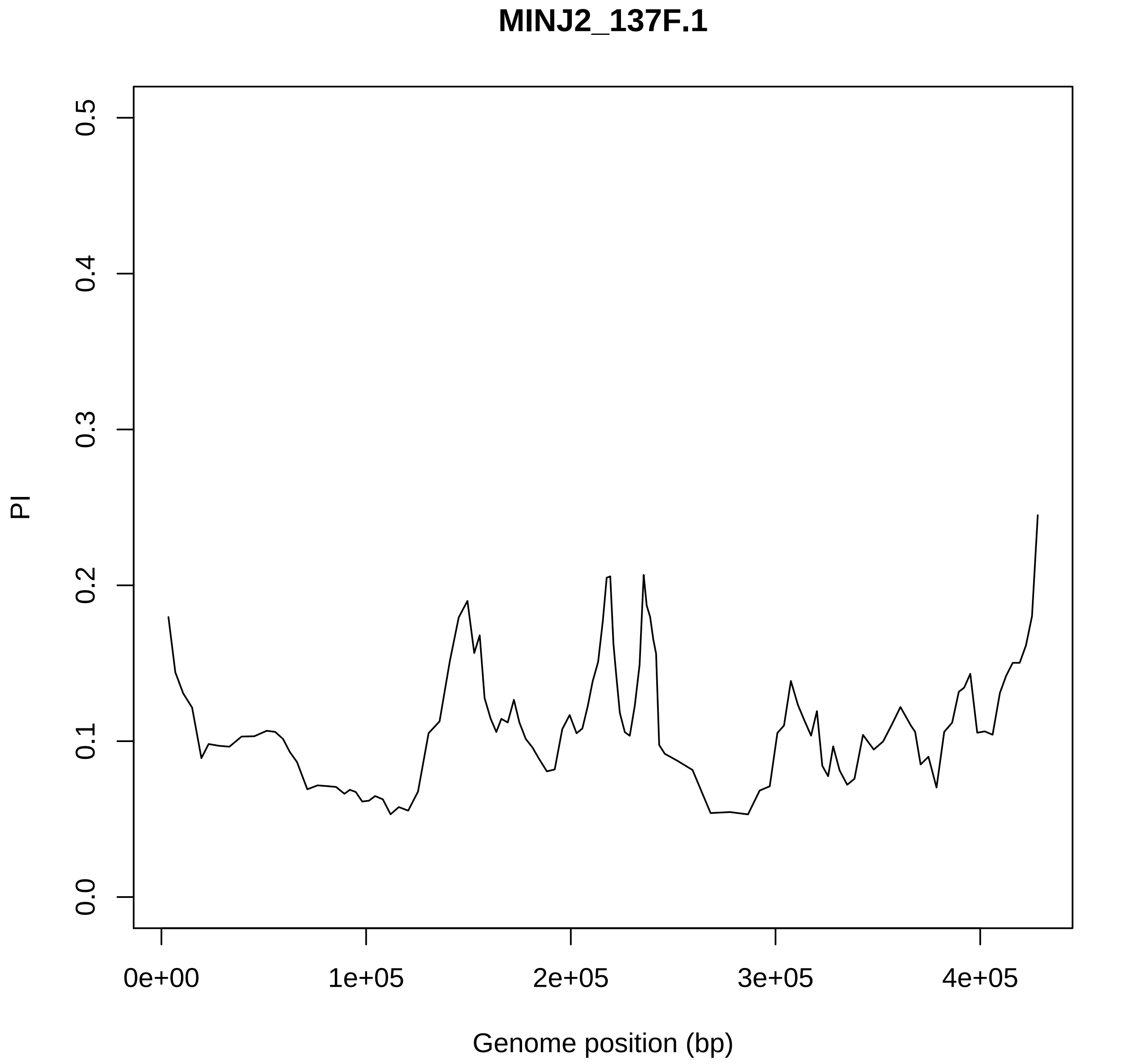
MINJ2_137F.1
PI
0.0
0.1
0.2
0.3
0.4
0.5
0e+00
1e+05
2e+05
3e+05
4e+05
Genome position (bp)

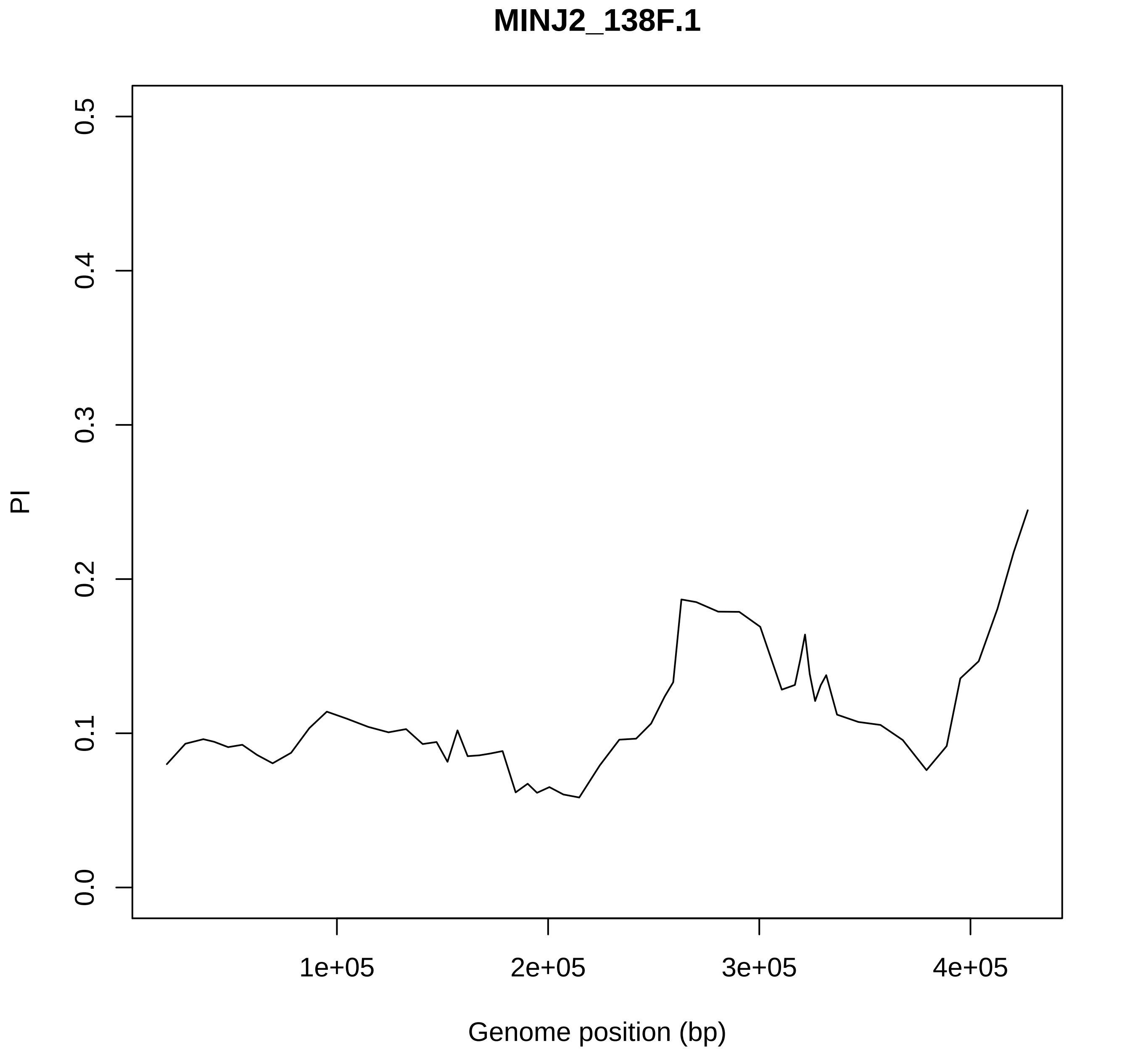

MINJ2_138F.1
PI
0.0
0.1
0.2
0.3
0.4
0.5
1e+05
2e+05
3e+05
4e+05
Genome position (bp)

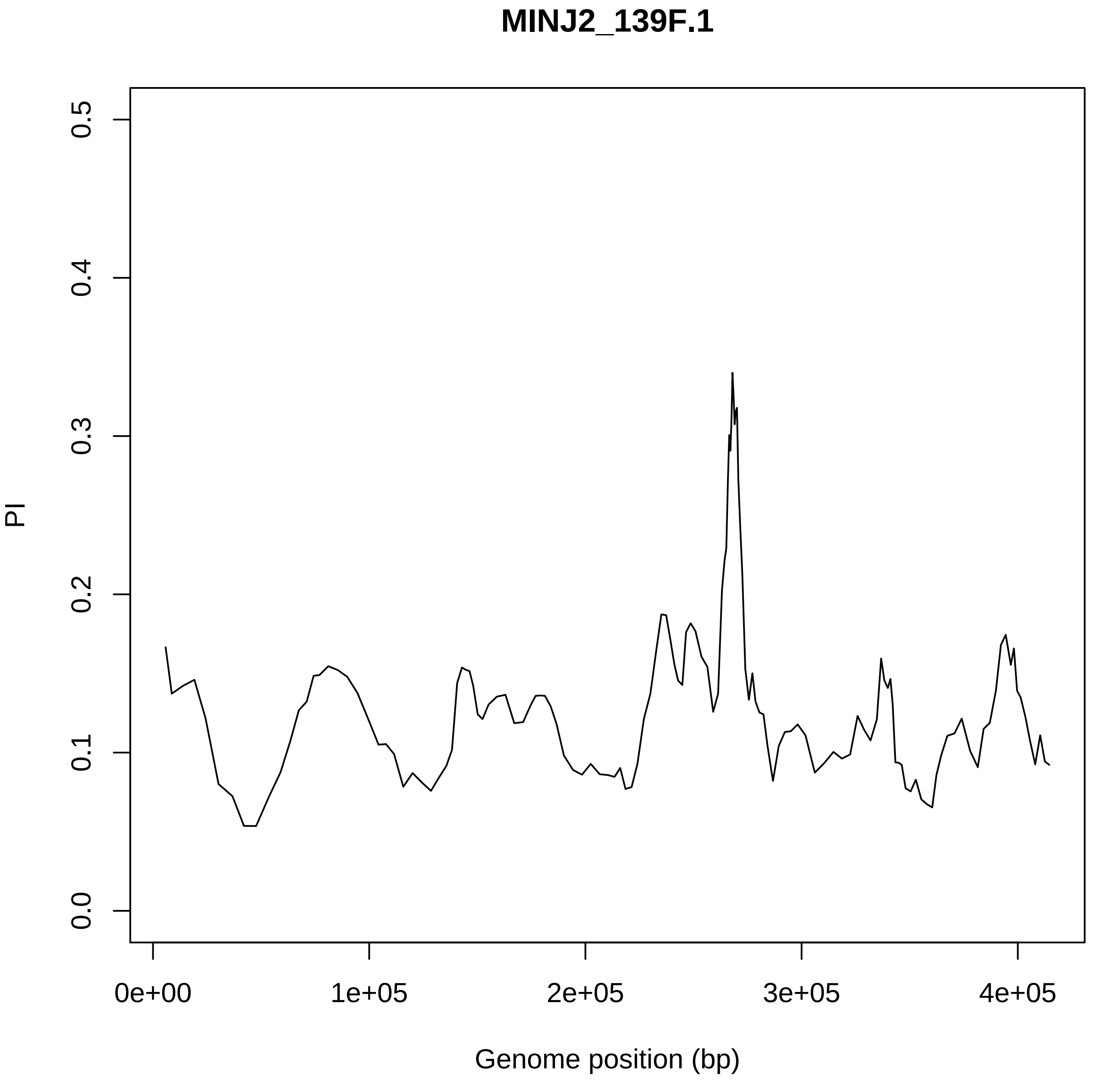
MINJ2_139F.1
PI
0.0
0.1
0.2
0.3
0.4
0.5
0e+00
1e+05
2e+05
3e+05
4e+05
Genome position (bp)

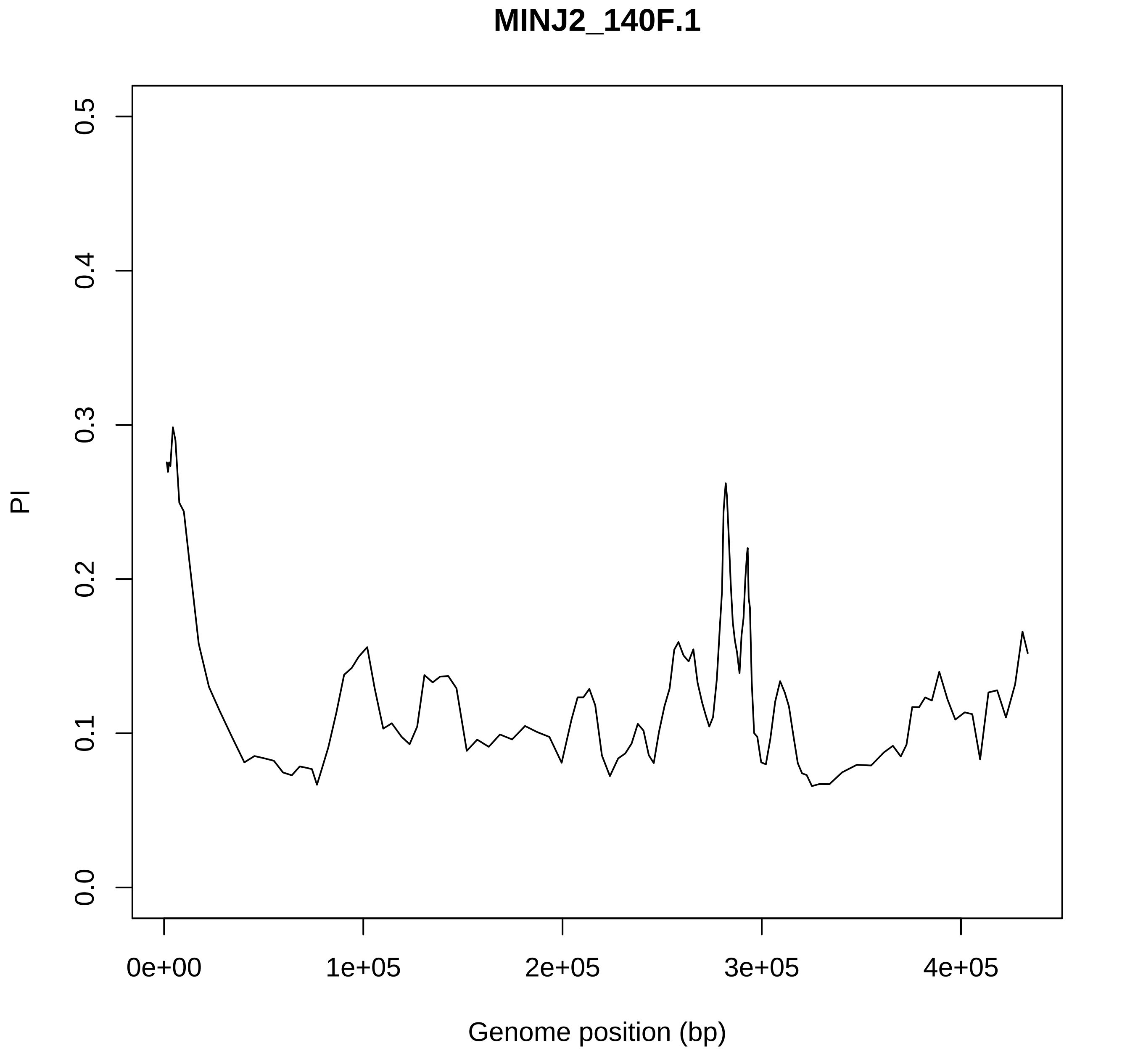
MINJ2_140F.1
PI
0.0
0.1
0.2
0.3
0.4
0.5
0e+00
1e+05
2e+05
3e+05
4e+05
Genome position (bp)

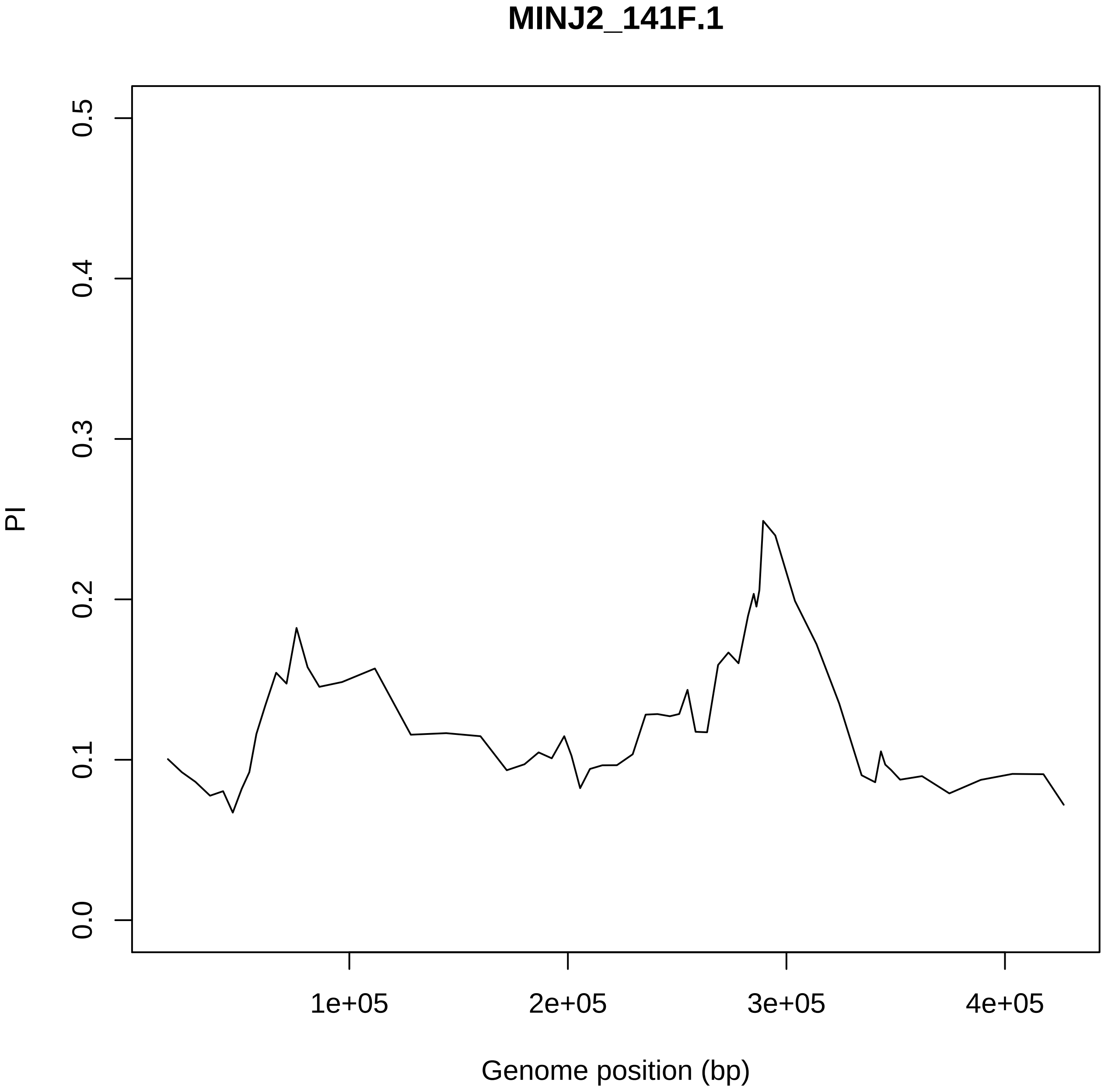
MINJ2_141F.1
PI
0.0
0.1
0.2
0.3
0.4
0.5
1e+05
2e+05
3e+05
4e+05
Genome position (bp)

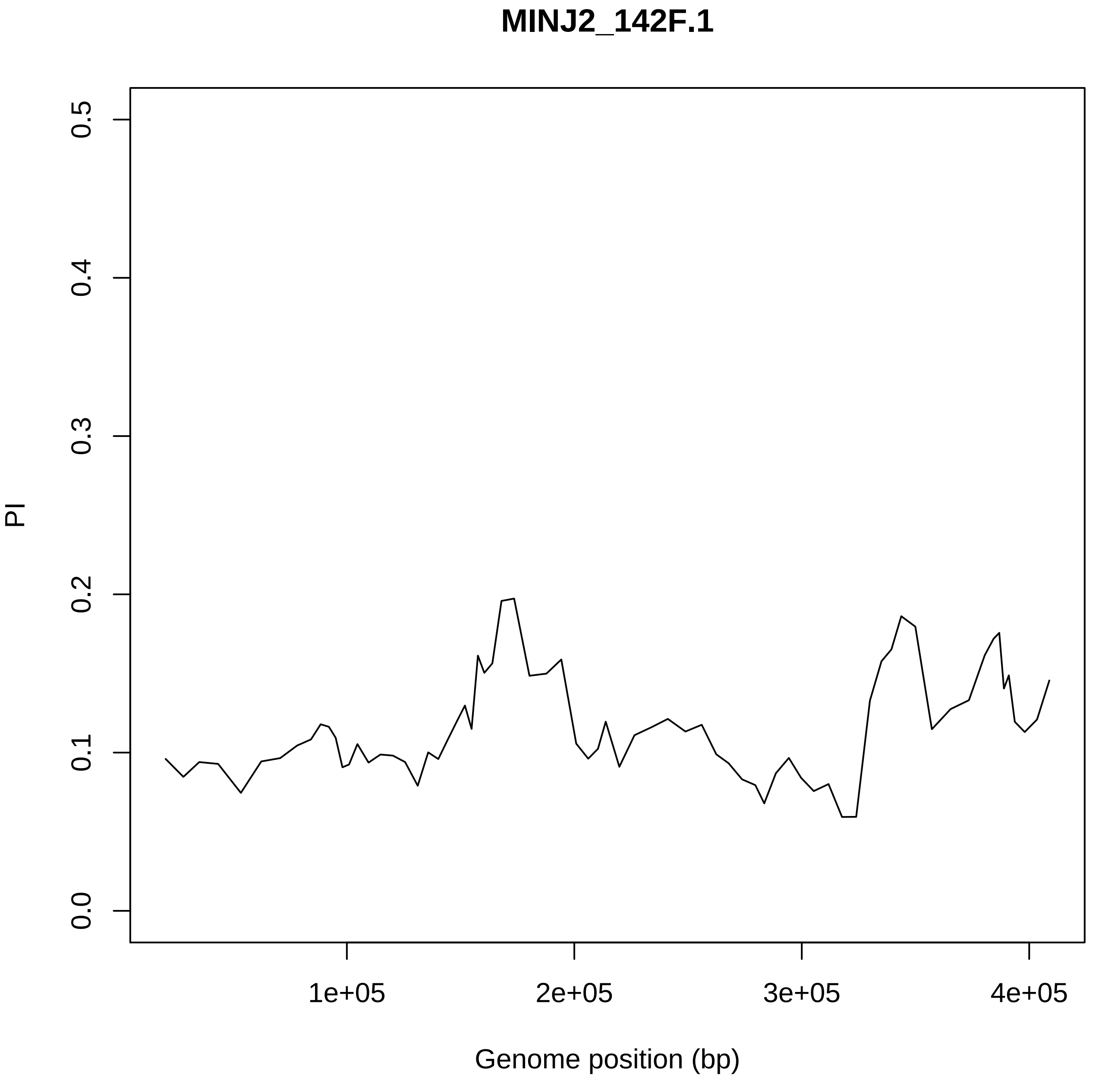
MINJ2_142F.1
PI
0.0
0.1
0.2
0.3
0.4
0.5
1e+05
2e+05
3e+05
4e+05
Genome position (bp)

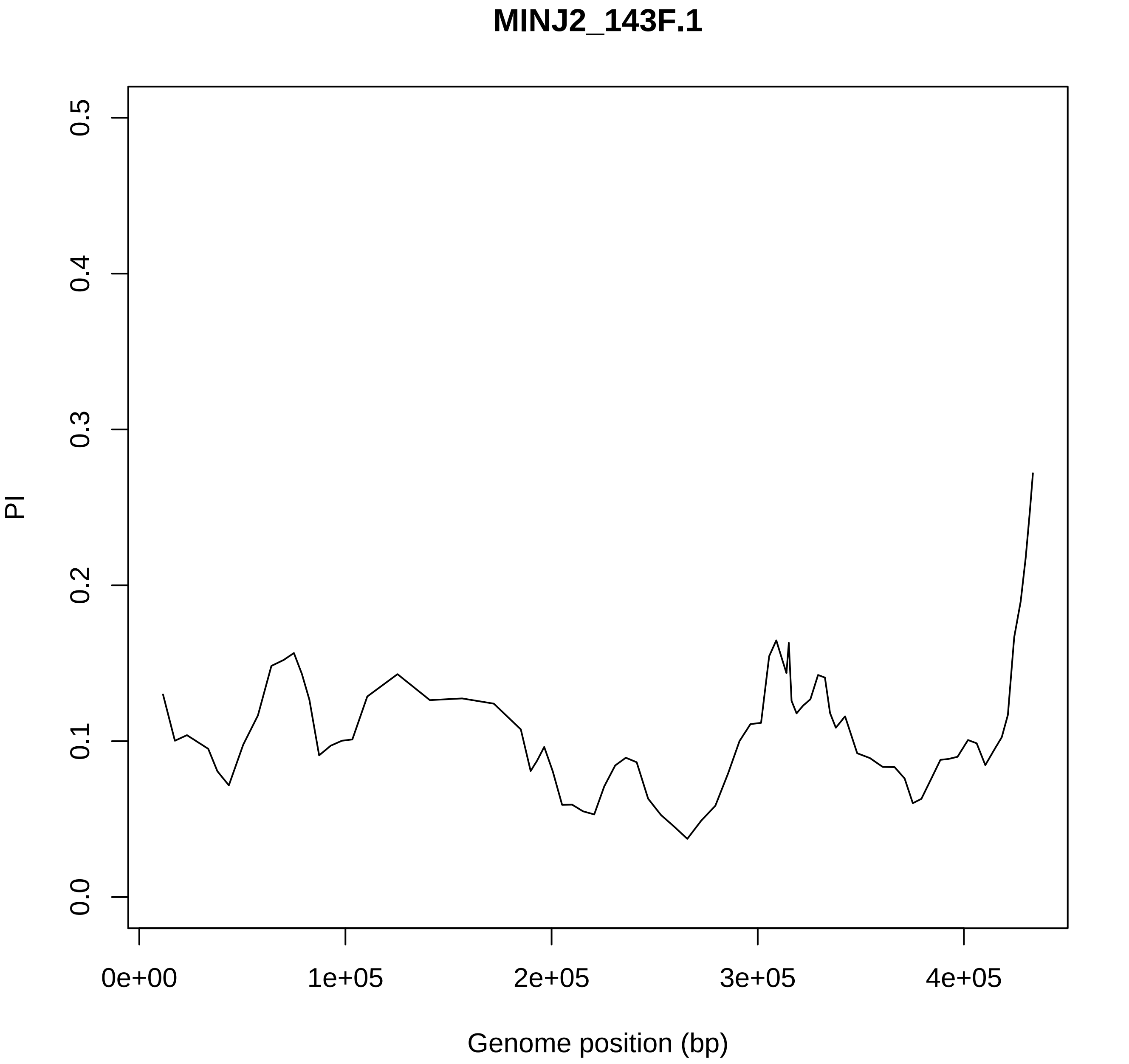

MINJ2_143F.1
PI
0.0
0.1
0.2
0.3
0.4
0.5
0e+00
1e+05
2e+05
3e+05
4e+05
Genome position (bp)

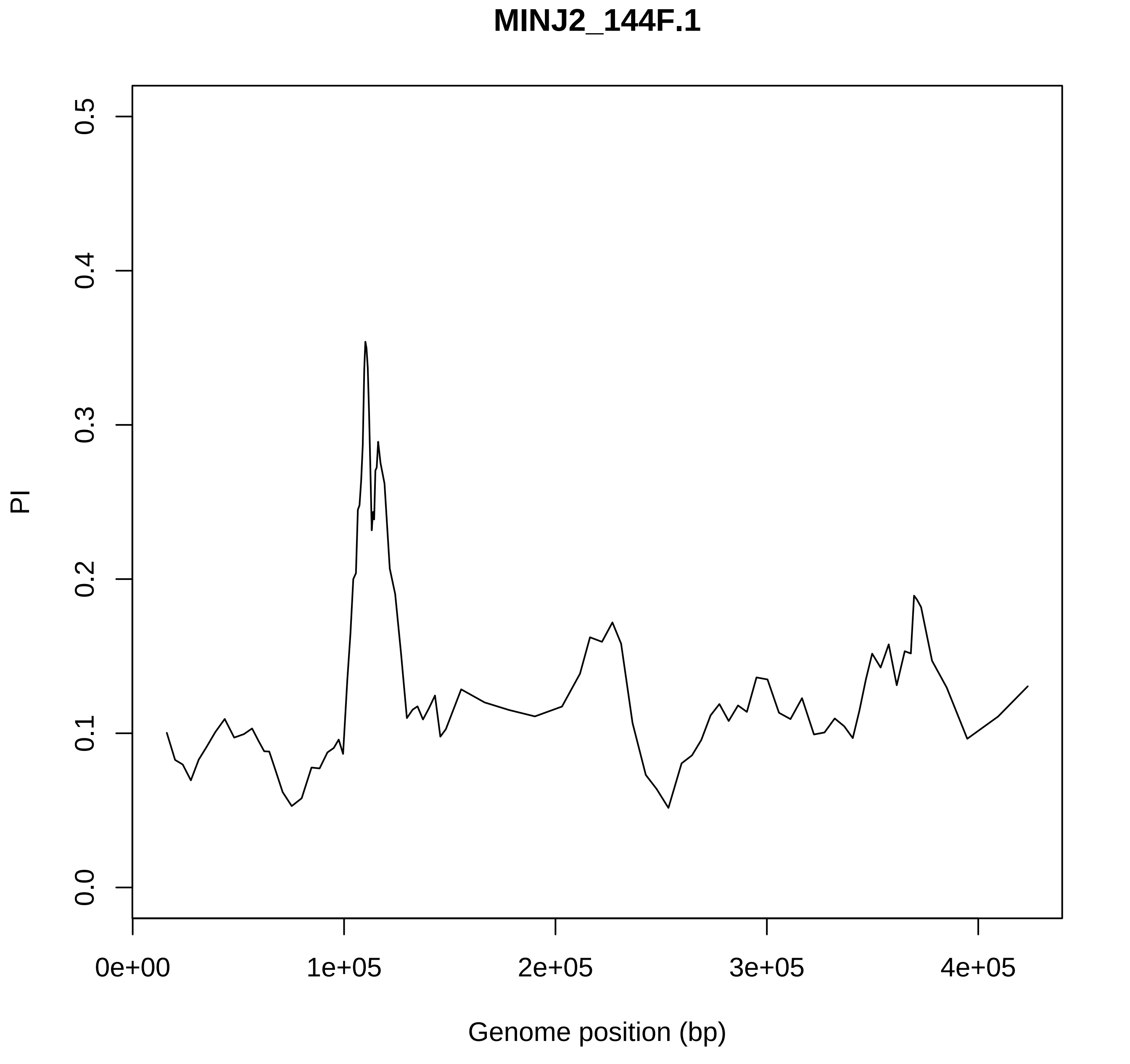

MINJ2_144F.1
PI
0.0
0.1
0.2
0.3
0.4
0.5
0e+00
1e+05
2e+05
3e+05
4e+05
Genome position (bp)

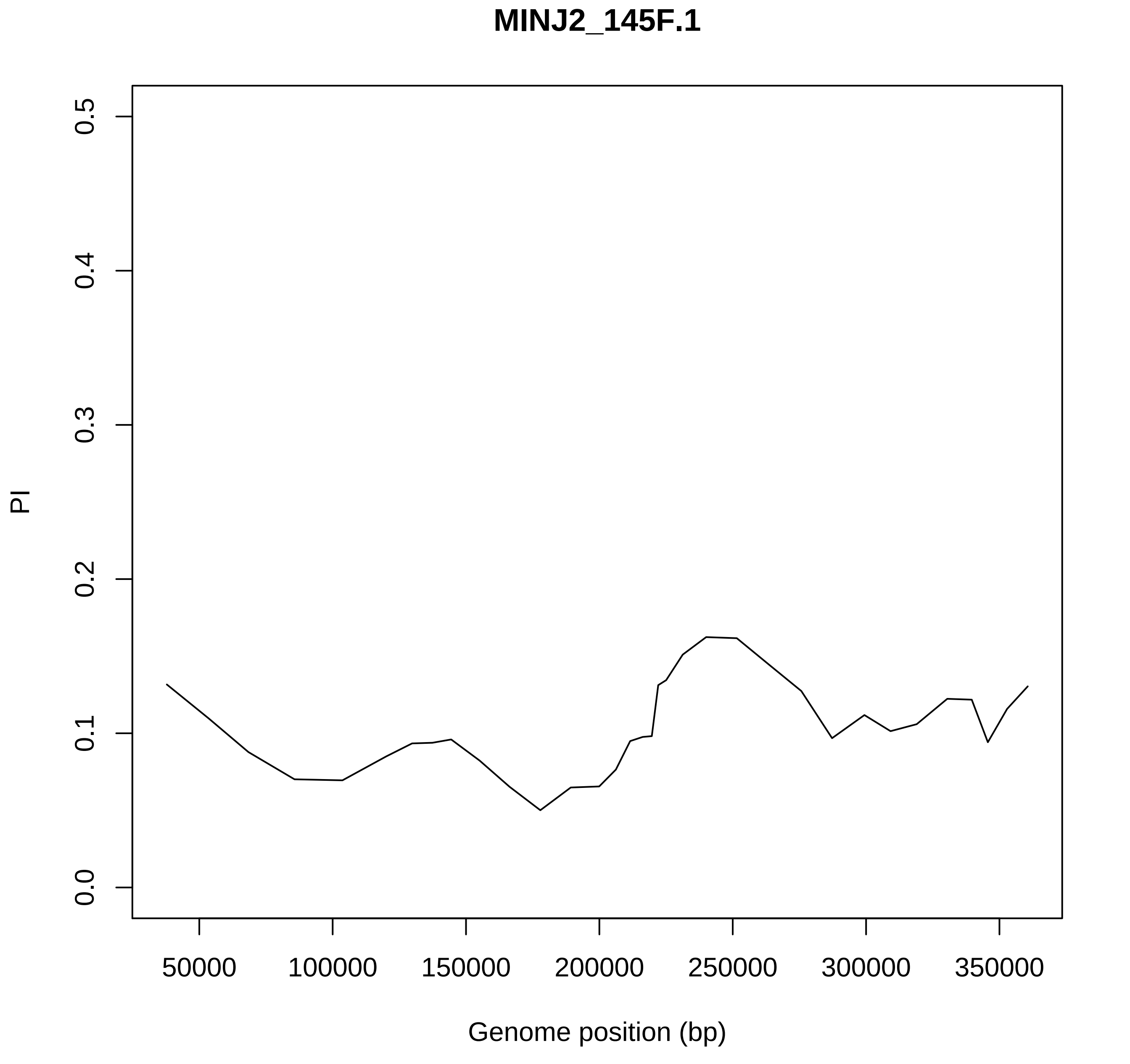
MINJ2_145F.1
PI
0.0
0.1
0.2
0.3
0.4
0.5
50000
100000
150000
200000
250000
300000
350000
Genome position (bp)

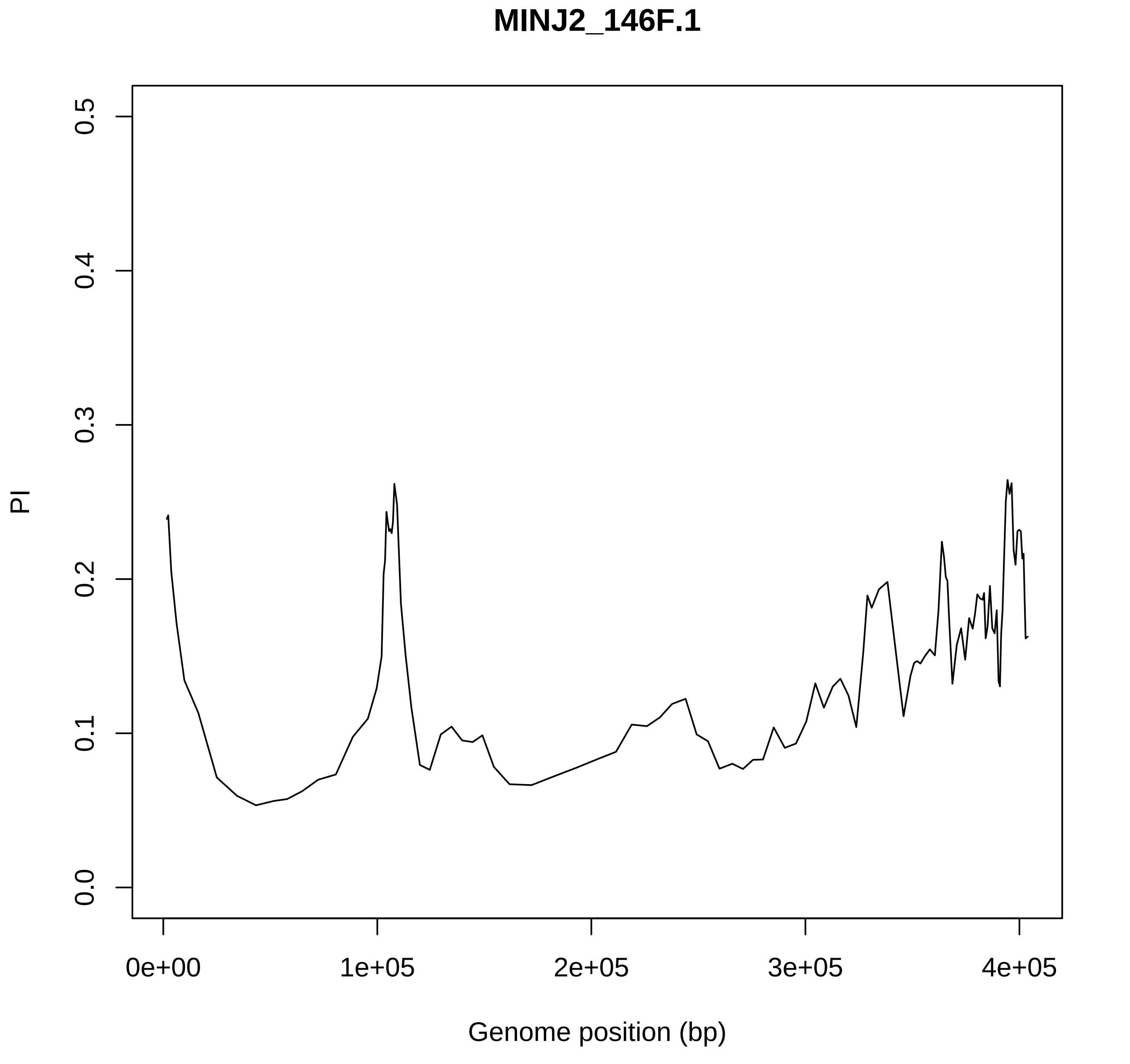
MINJ2_146F.1
PI
0.0
0.1
0.2
0.3
0.4
0.5
0e+00
1e+05
2e+05
3e+05
4e+05
Genome position (bp)

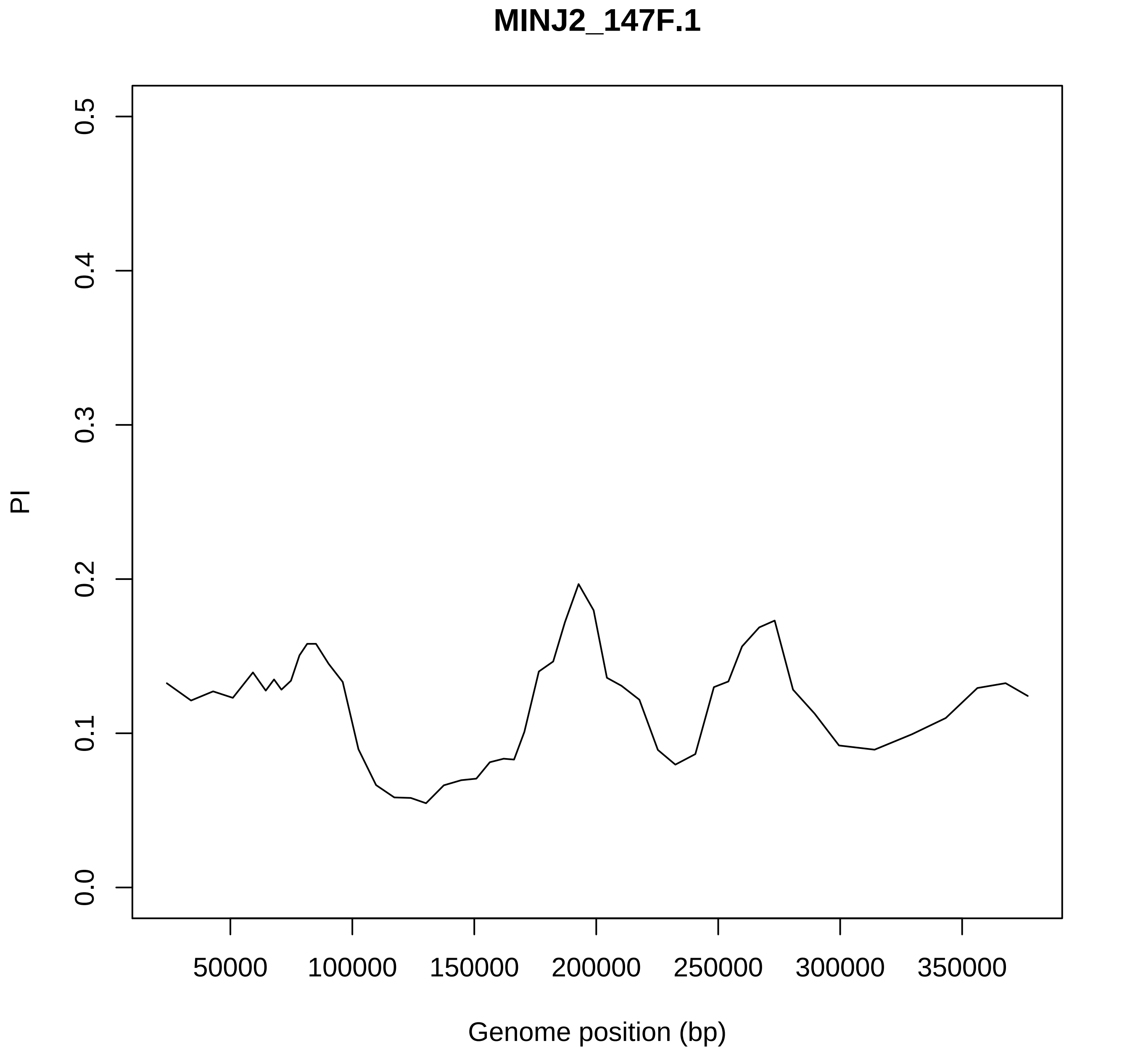

MINJ2_147F.1
PI
0.0
0.1
0.2
0.3
0.4
0.5
50000
100000
150000
200000
250000
300000
350000
Genome position (bp)

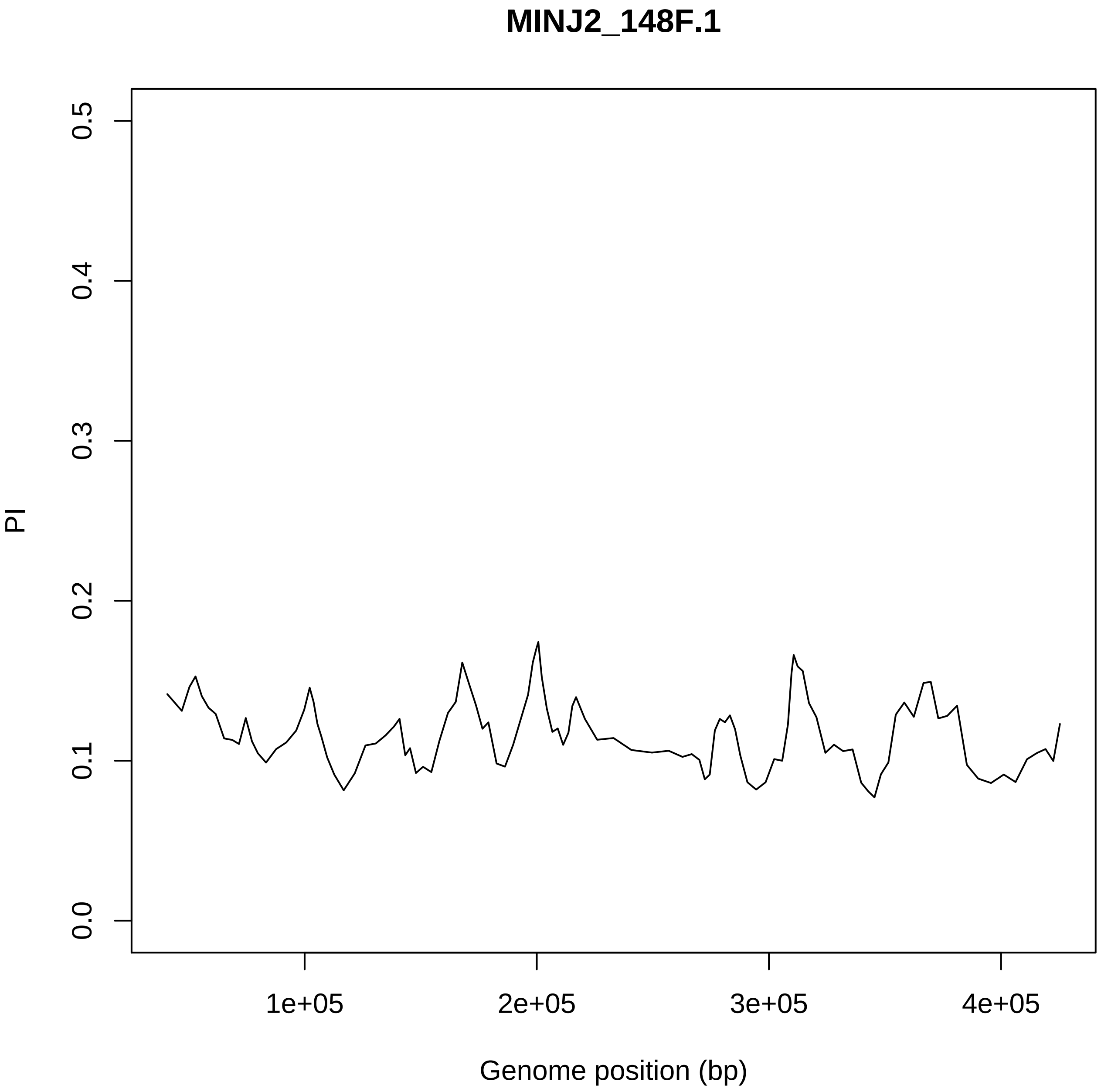
MINJ2_148F.1
PI
0.0
0.1
0.2
0.3
0.4
0.5
1e+05
2e+05
3e+05
4e+05
Genome position (bp)

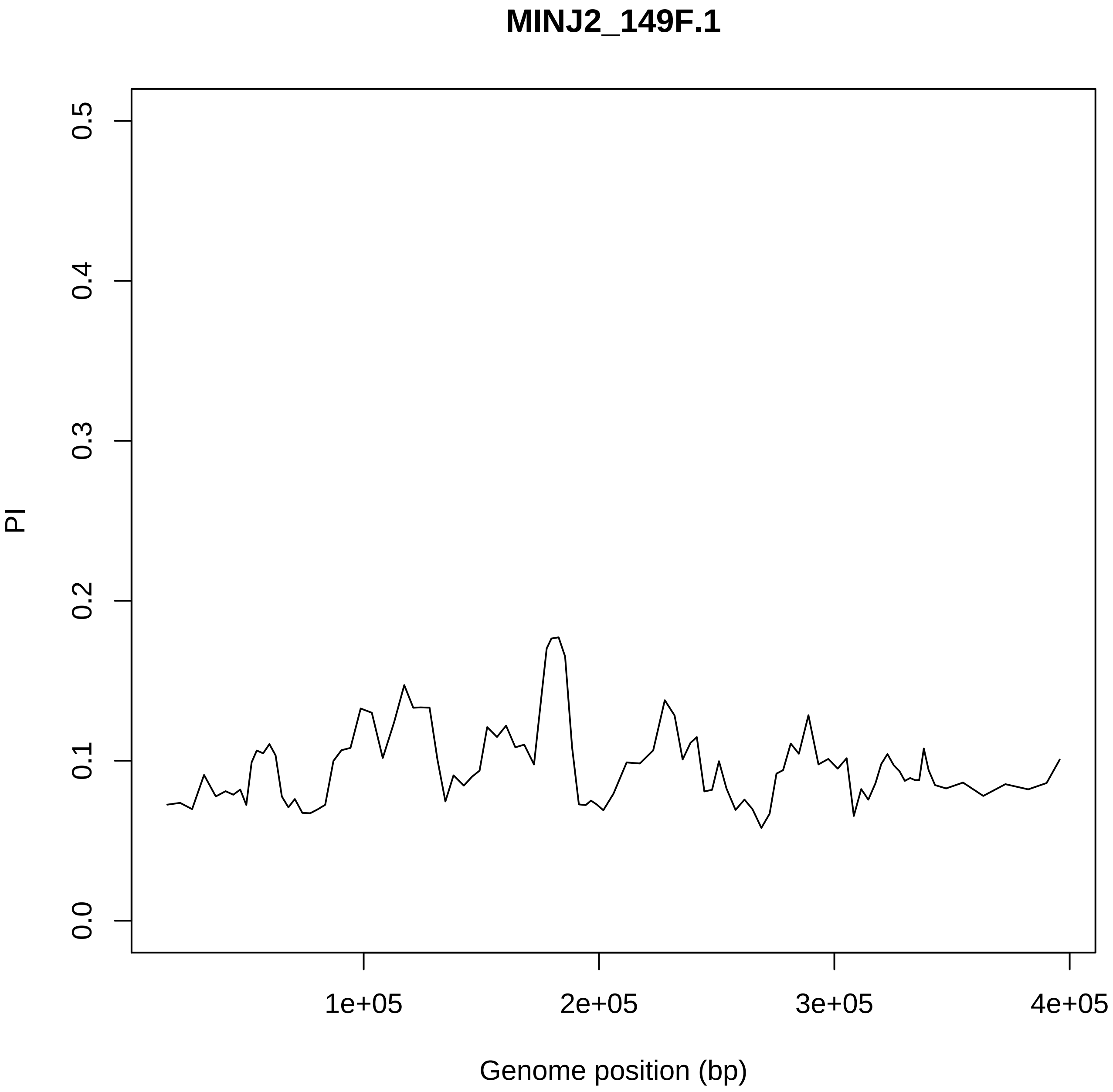
MINJ2_149F.1
PI
0.0
0.1
0.2
0.3
0.4
0.5
1e+05
2e+05
3e+05
4e+05
Genome position (bp)

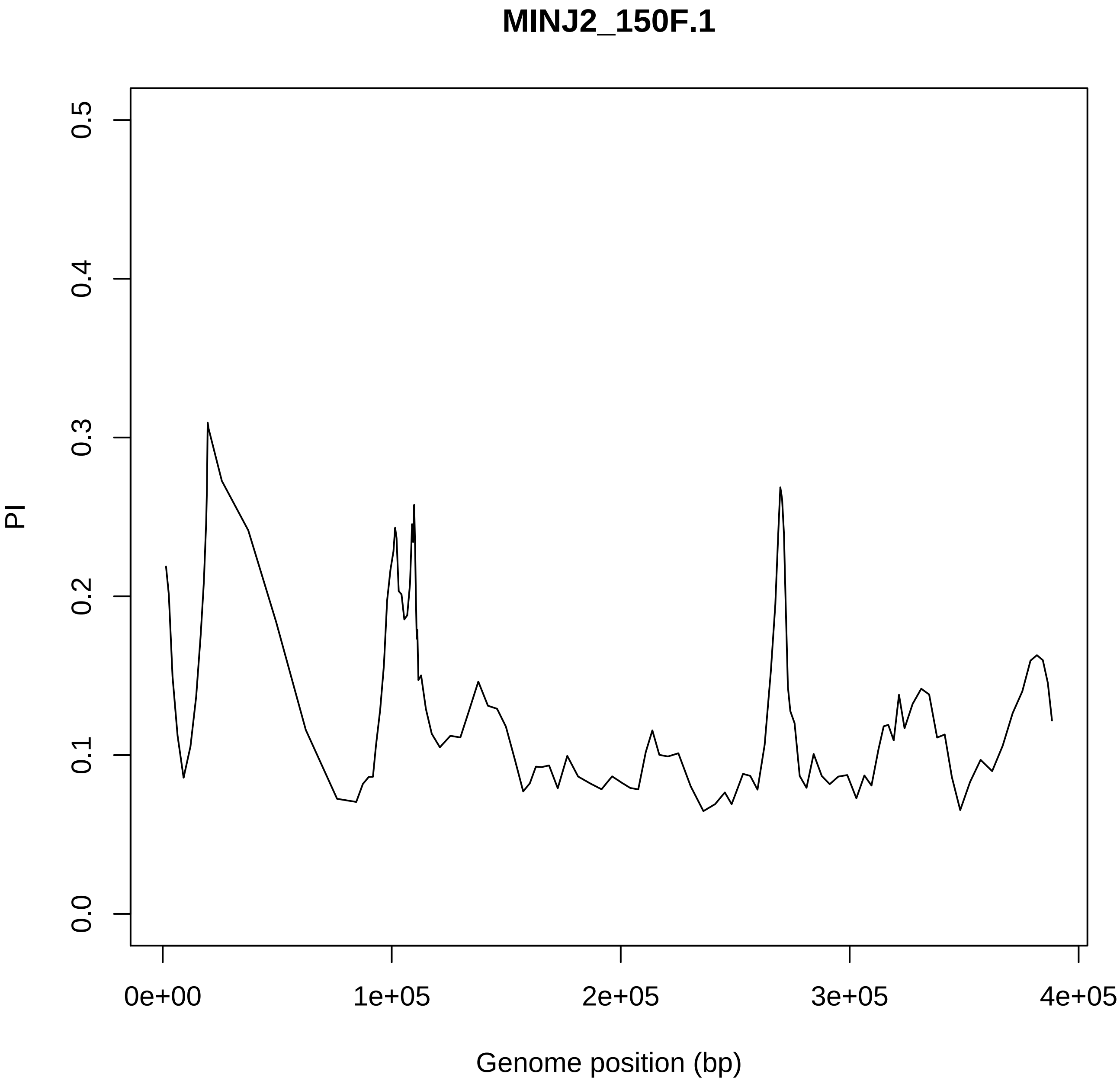

MINJ2_150F.1
PI
0.0
0.1
0.2
0.3
0.4
0.5
0e+00
1e+05
2e+05
3e+05
4e+05
Genome position (bp)

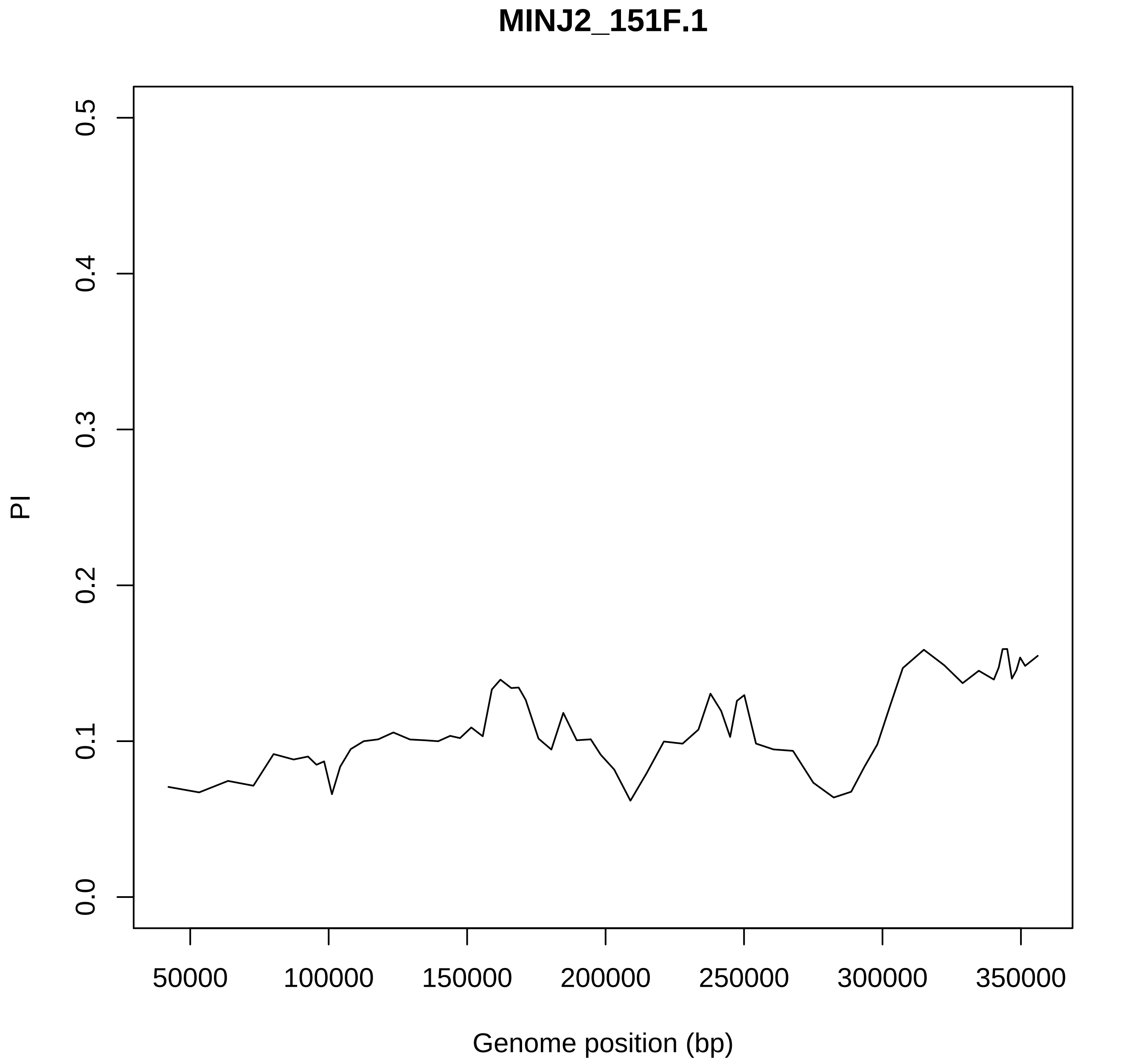

MINJ2_151F.1
PI
0.0
0.1
0.2
0.3
0.4
0.5
50000
100000
150000
200000
250000
300000
350000
Genome position (bp)

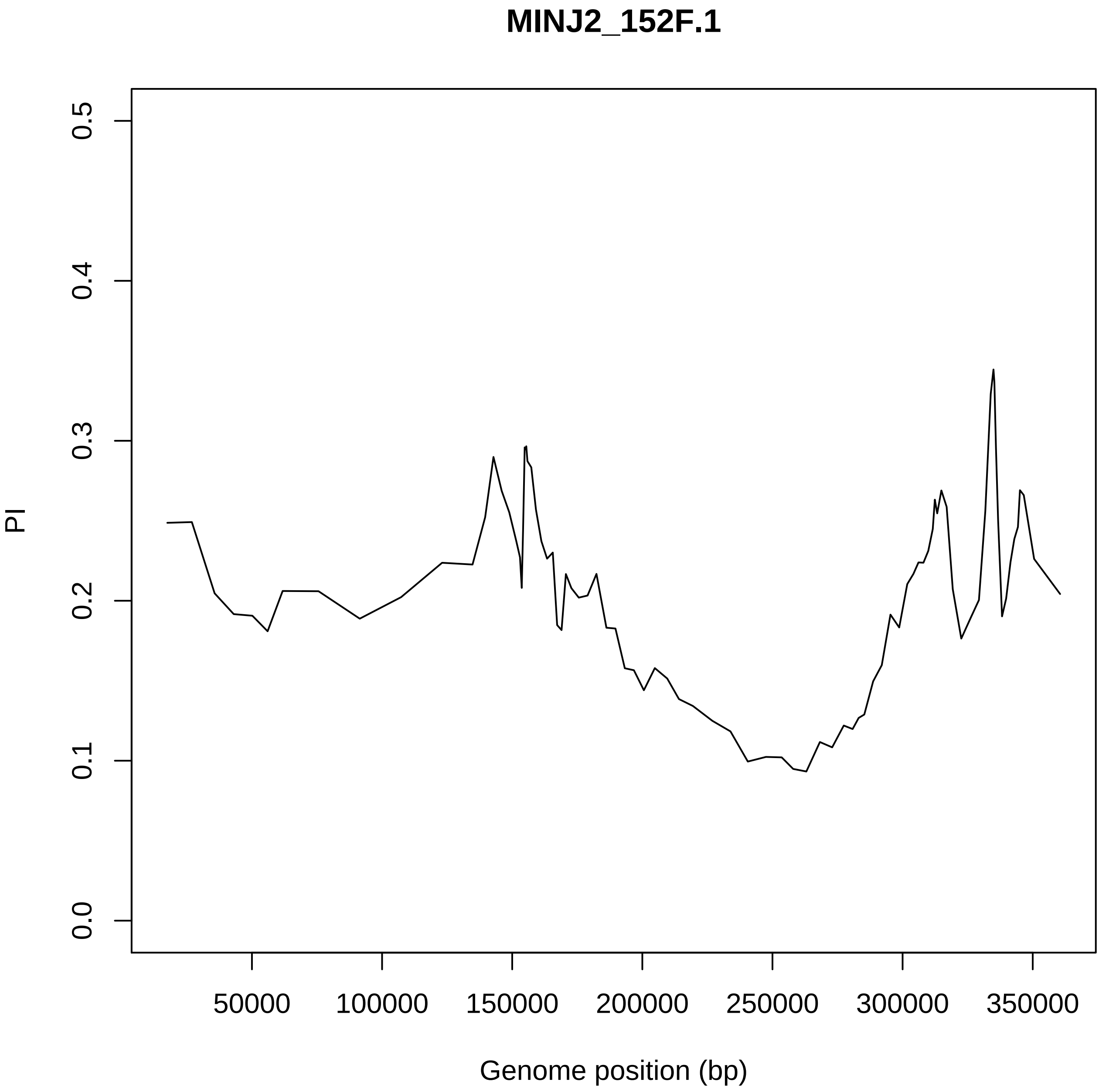

MINJ2_152F.1
PI
0.0
0.1
0.2
0.3
0.4
0.5
50000
100000
150000
200000
250000
300000
350000
Genome position (bp)

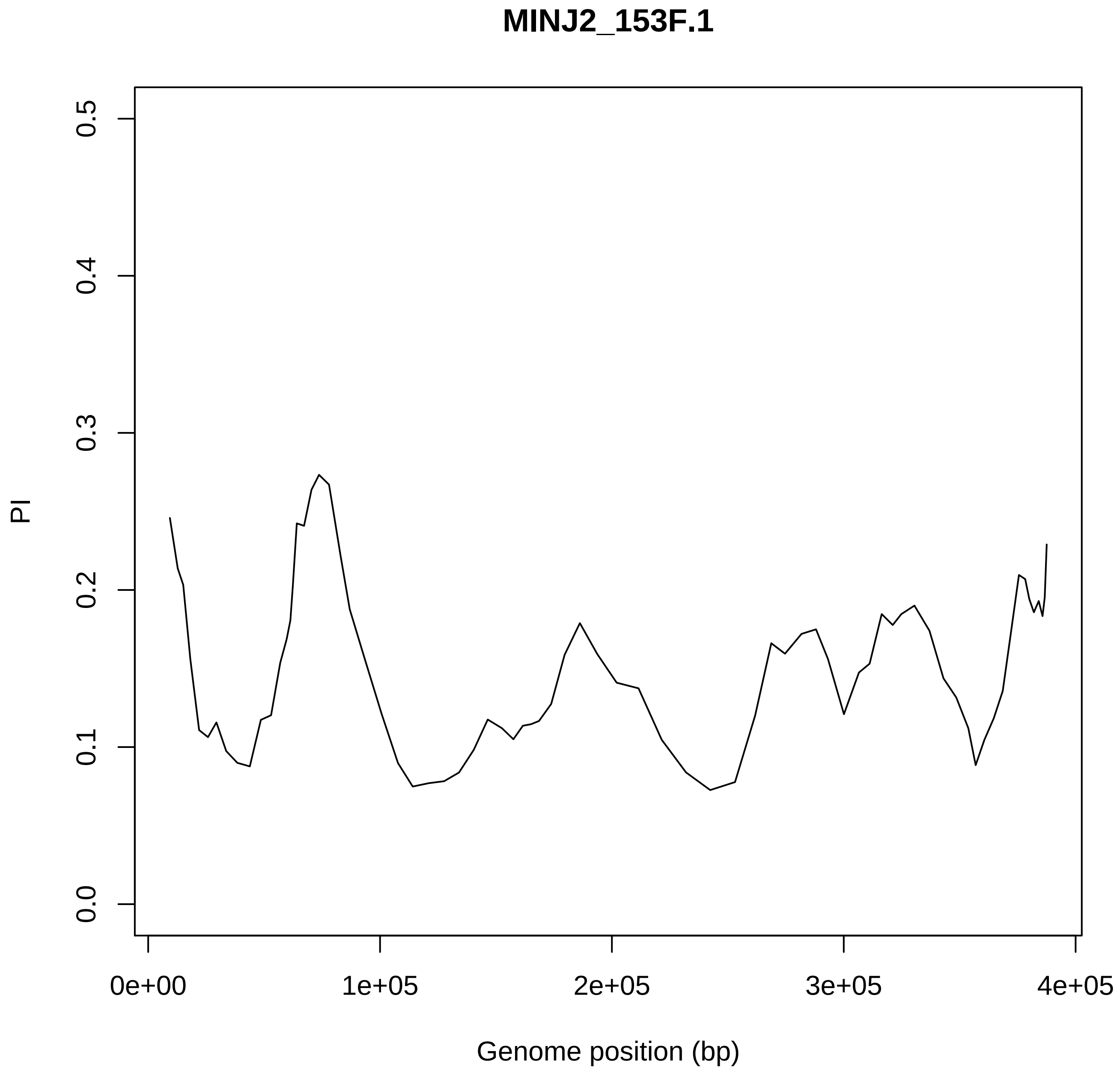
MINJ2_153F.1
PI
0.0
0.1
0.2
0.3
0.4
0.5
0e+00
1e+05
2e+05
3e+05
4e+05
Genome position (bp)

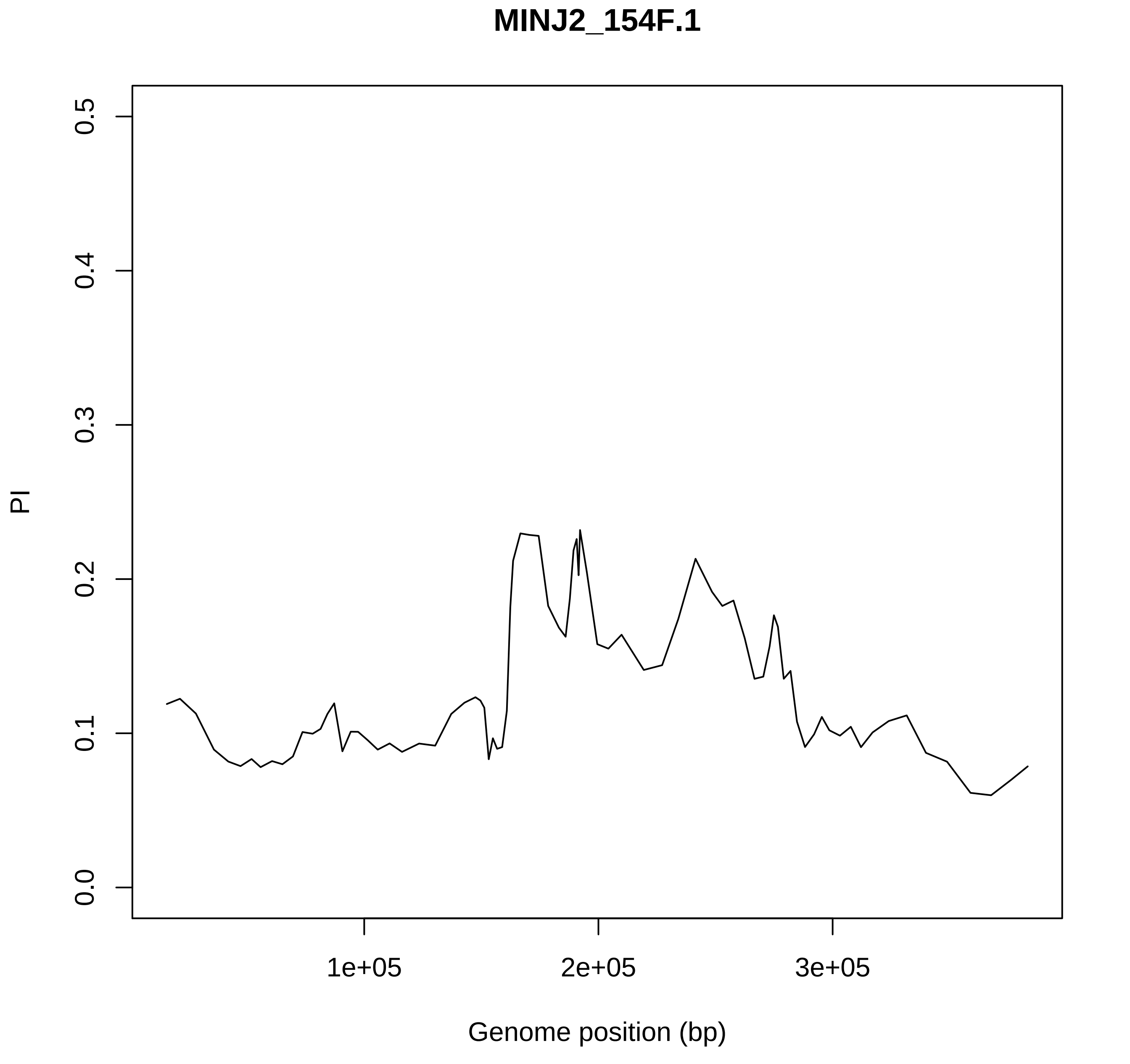
MINJ2_154F.1
PI
0.0
0.1
0.2
0.3
0.4
0.5
1e+05
2e+05
3e+05
Genome position (bp)

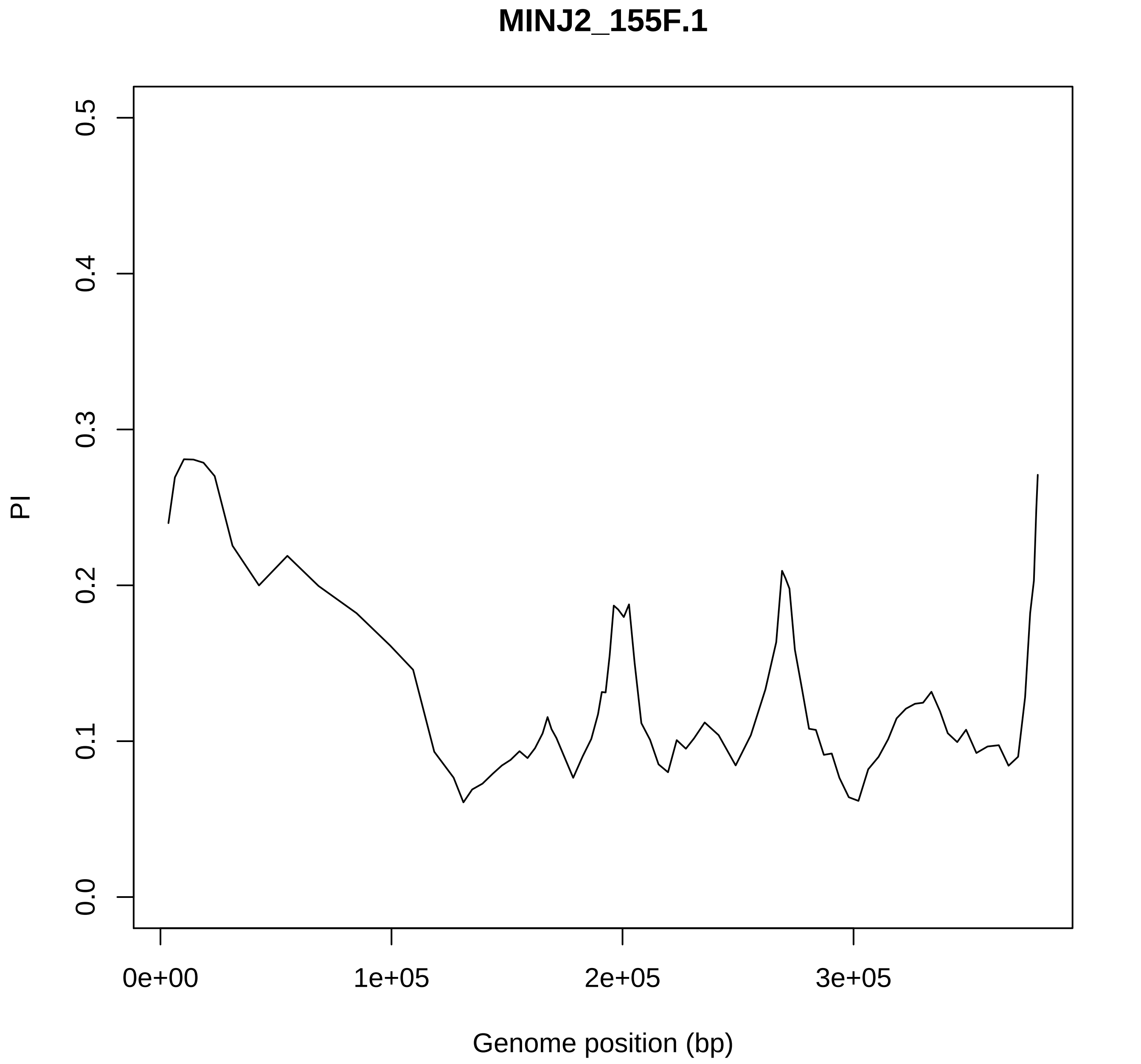
MINJ2_155F.1
PI
0.0
0.1
0.2
0.3
0.4
0.5
0e+00
1e+05
2e+05
3e+05
Genome position (bp)

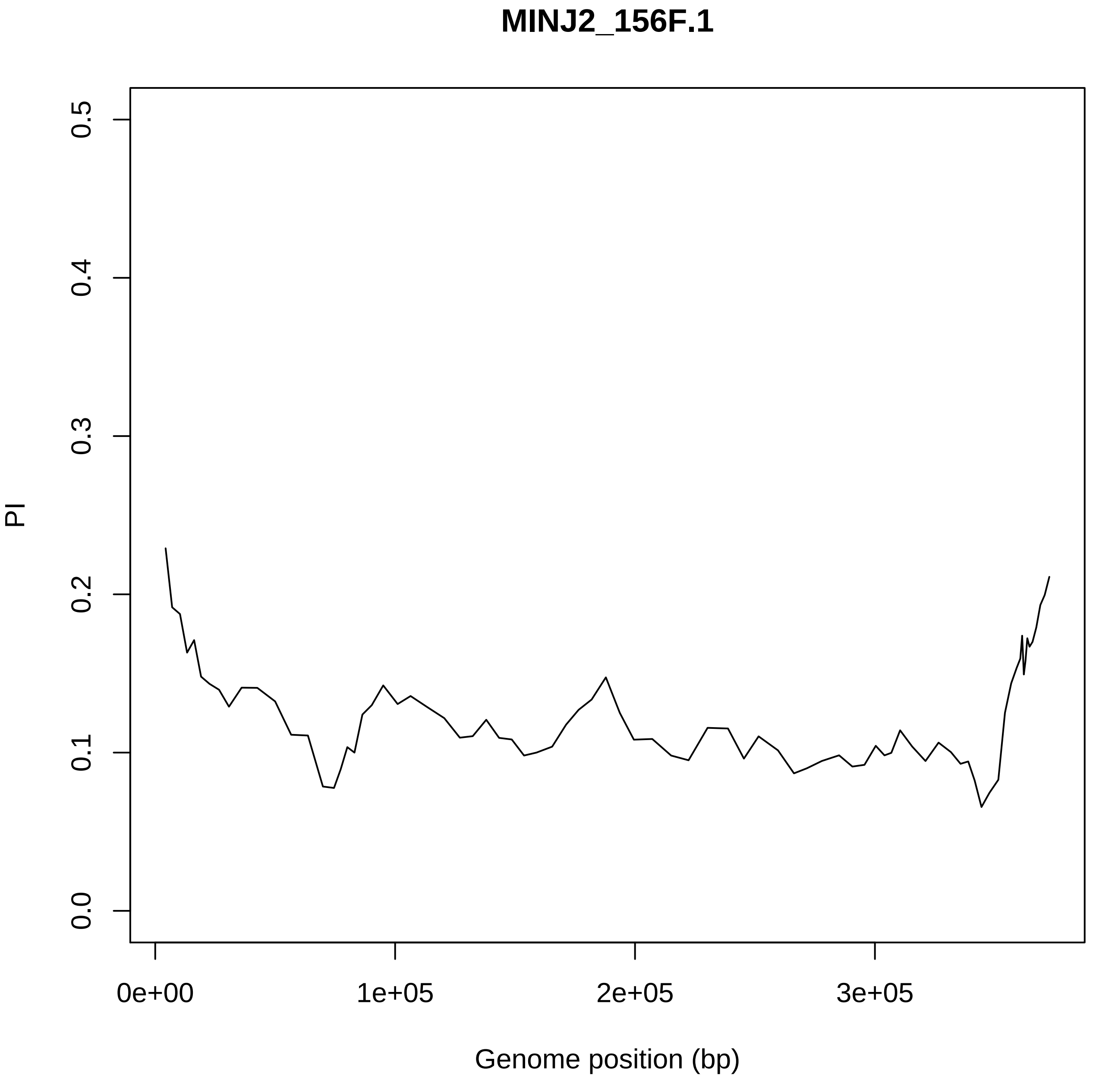

MINJ2_156F.1
PI
0.0
0.1
0.2
0.3
0.4
0.5
0e+00
1e+05
2e+05
3e+05
Genome position (bp)

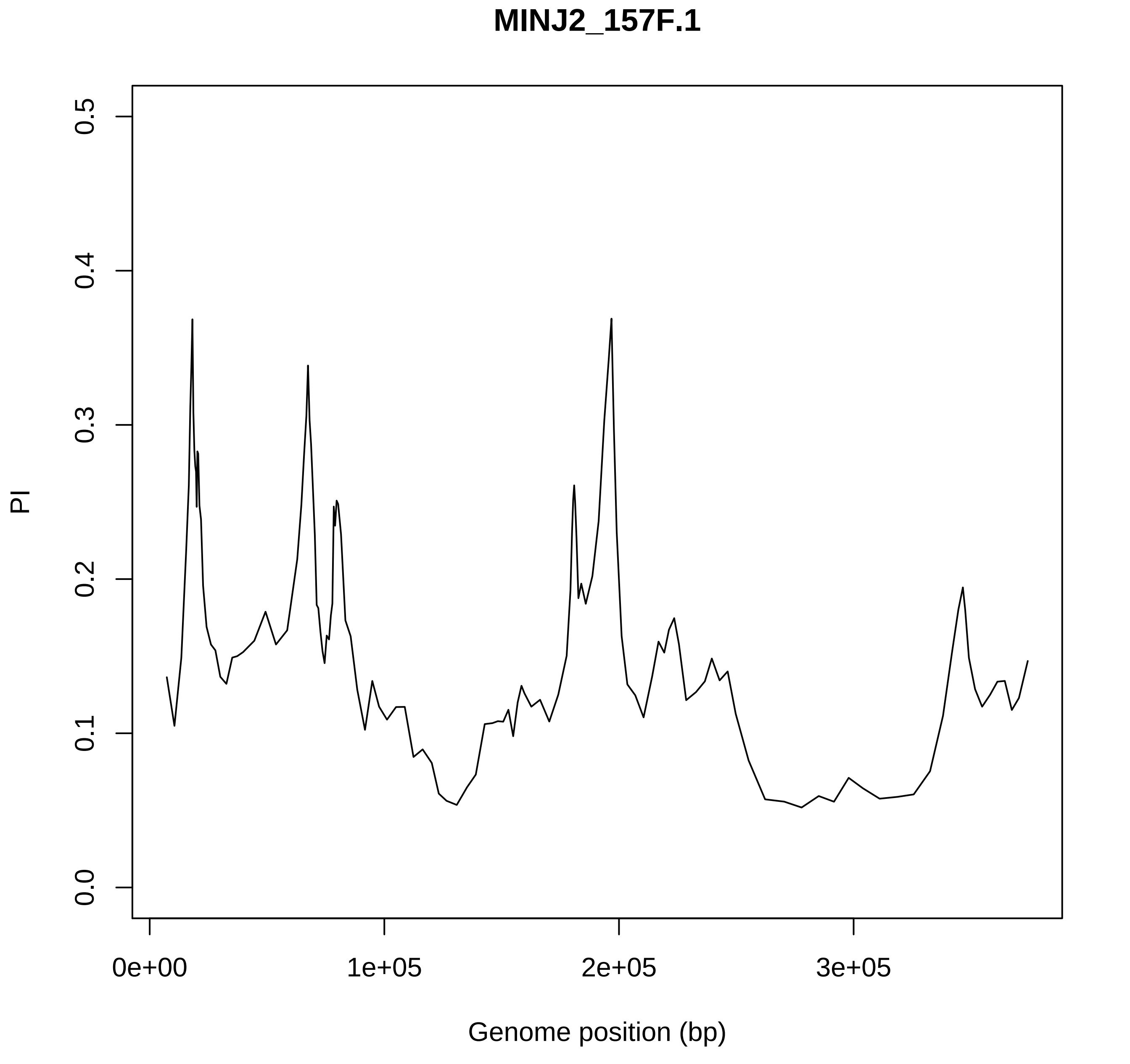
MINJ2_157F.1
PI
0.0
0.1
0.2
0.3
0.4
0.5
0e+00
1e+05
2e+05
3e+05
Genome position (bp)

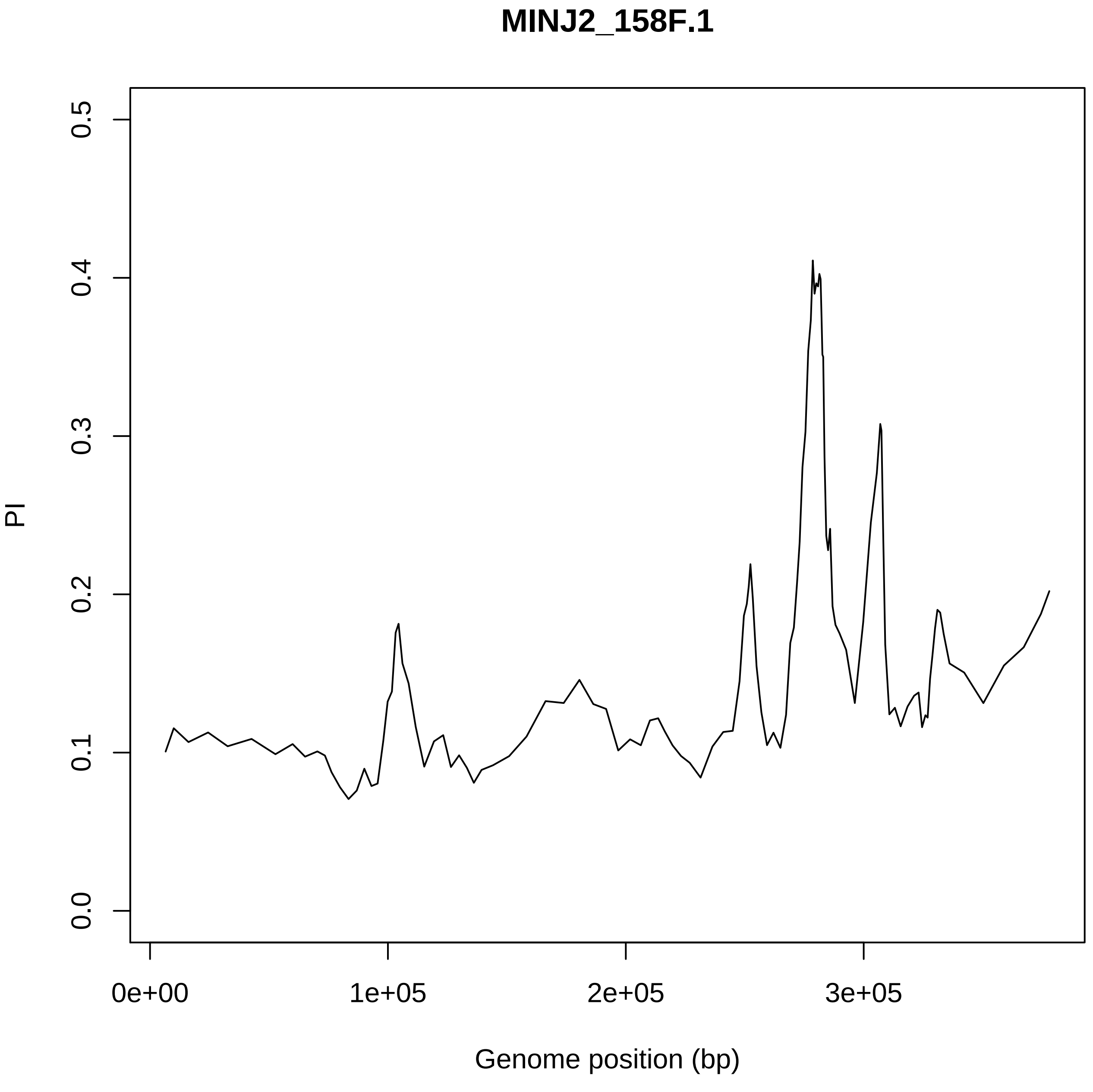
MINJ2_158F.1
PI
0.0
0.1
0.2
0.3
0.4
0.5
0e+00
1e+05
2e+05
3e+05
Genome position (bp)

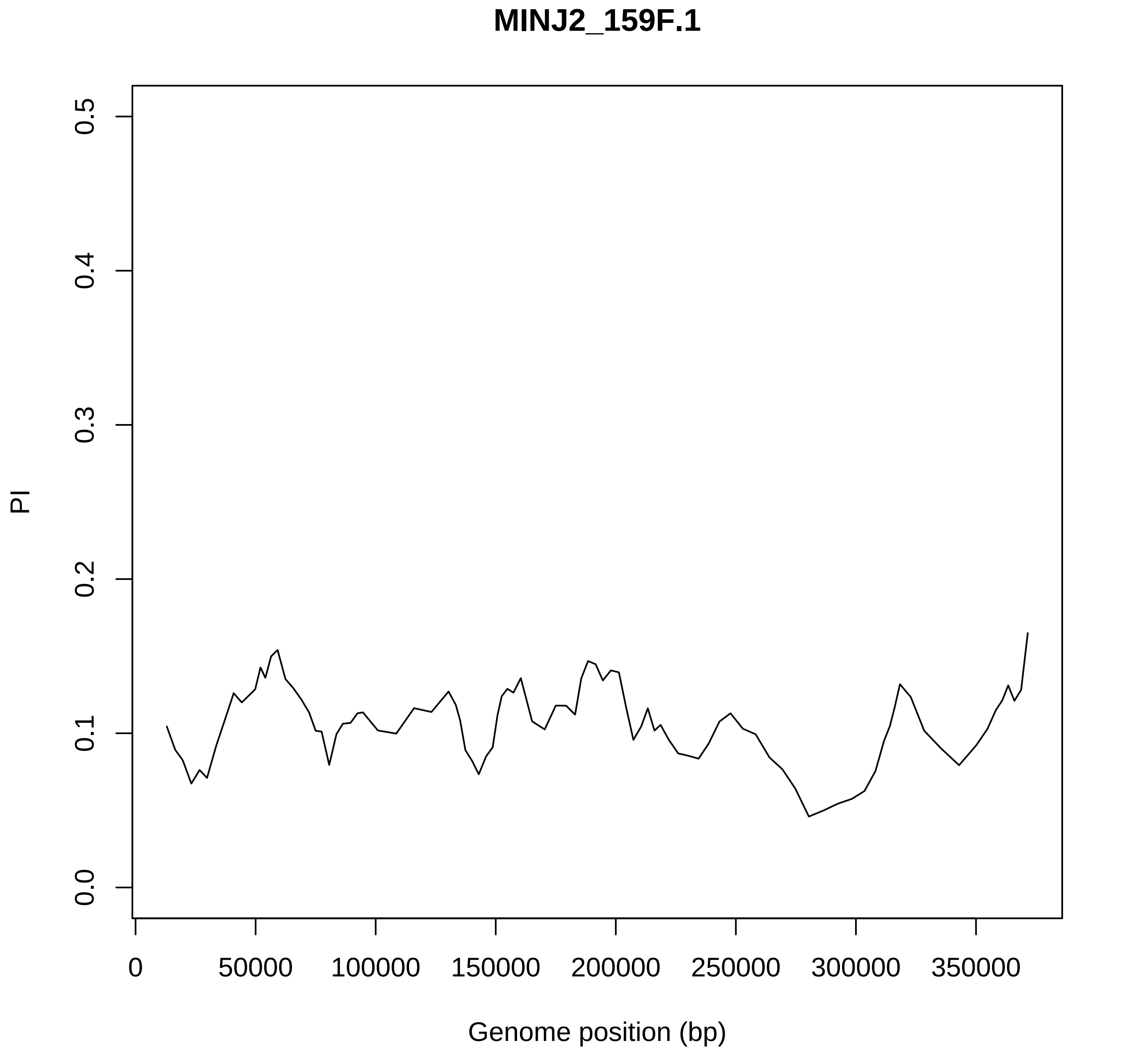
MINJ2_159F.1
PI
0.0
0.1
0.2
0.3
0.4
0.5
0
50000
100000
150000
200000
250000
300000
350000
Genome position (bp)

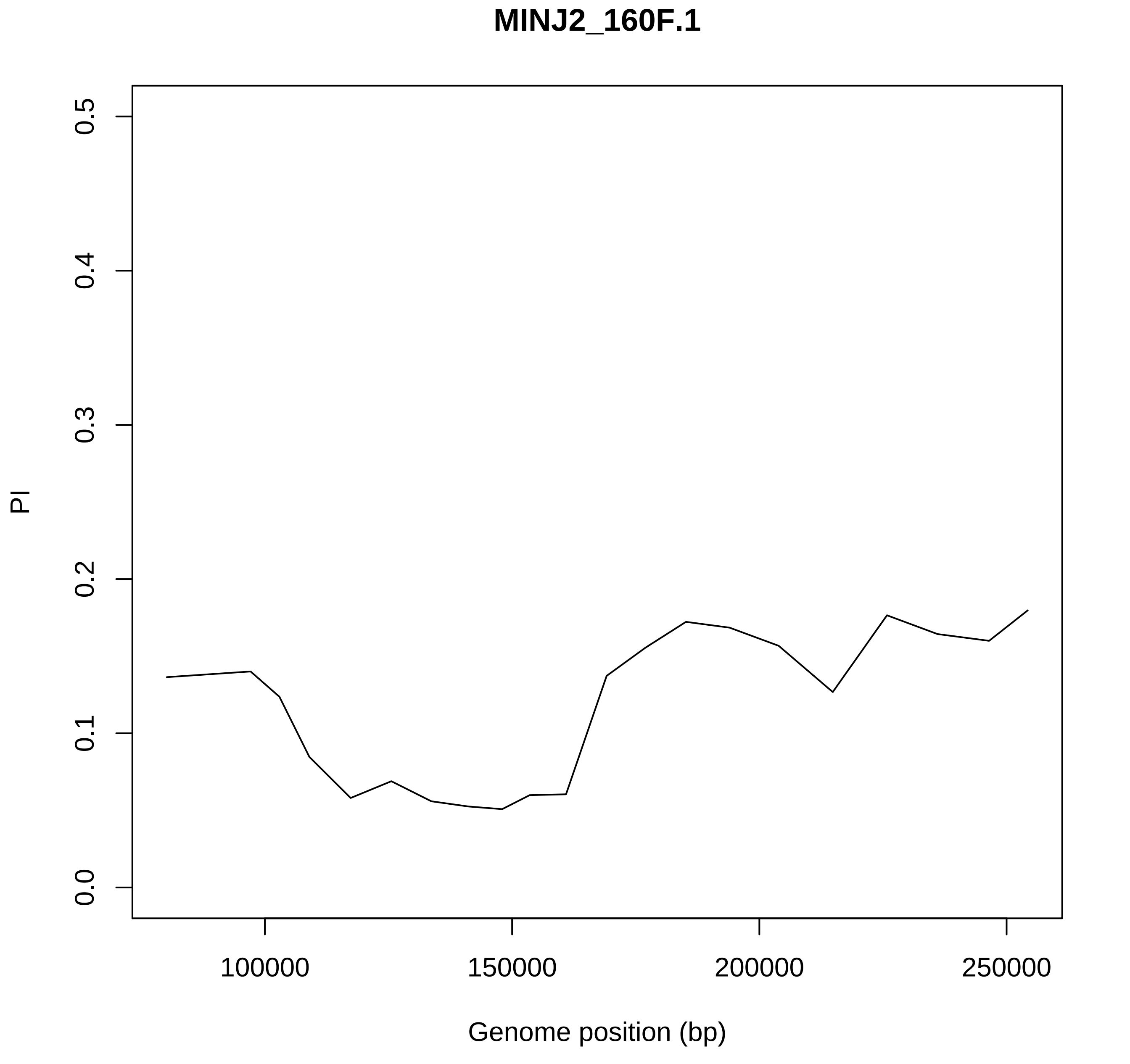

MINJ2_160F.1
PI
0.0
0.1
0.2
0.3
0.4
0.5
100000
150000
200000
250000
Genome position (bp)

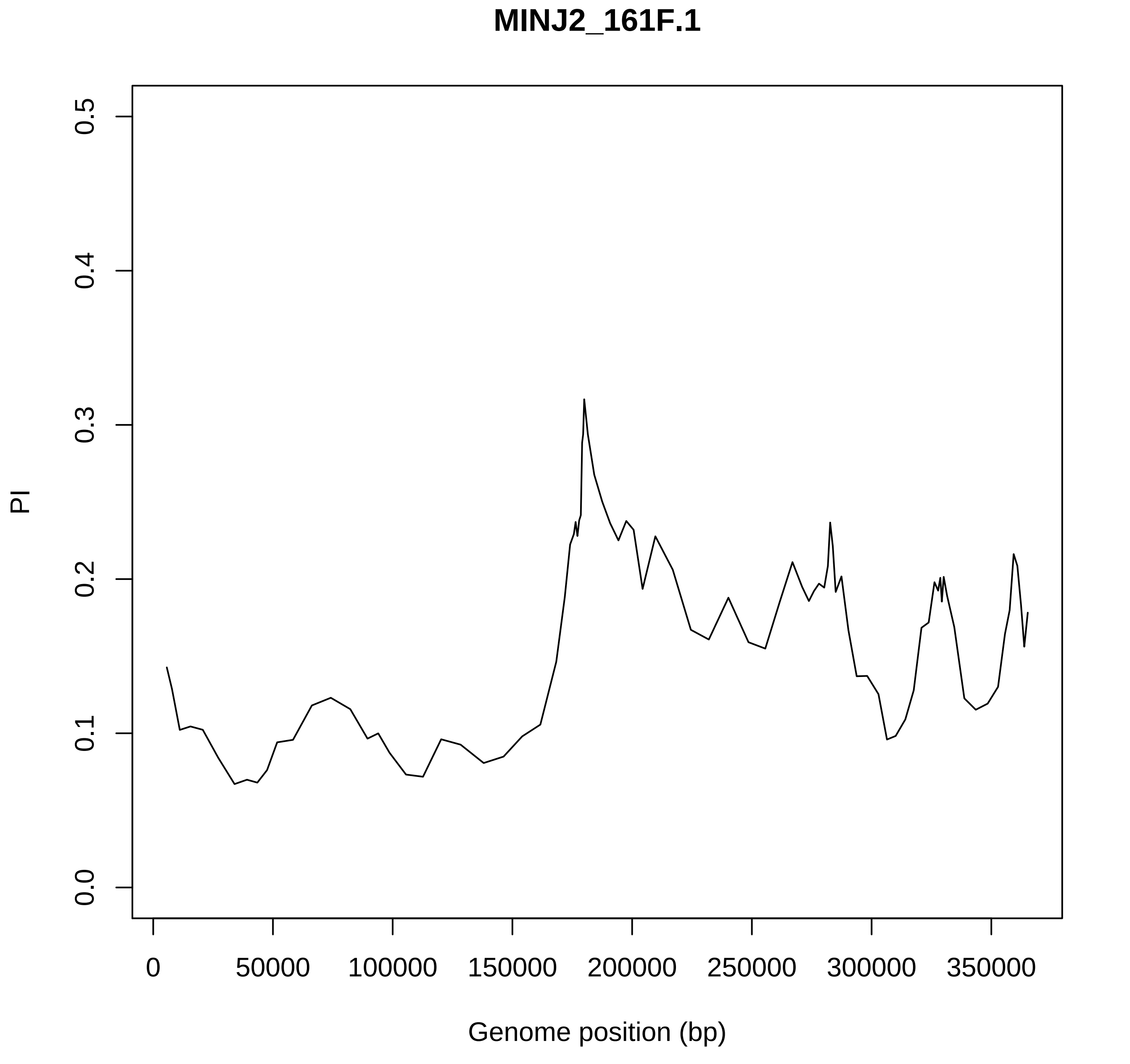
MINJ2_161F.1
PI
0.0
0.1
0.2
0.3
0.4
0.5
0
50000
100000
150000
200000
250000
300000
350000
Genome position (bp)

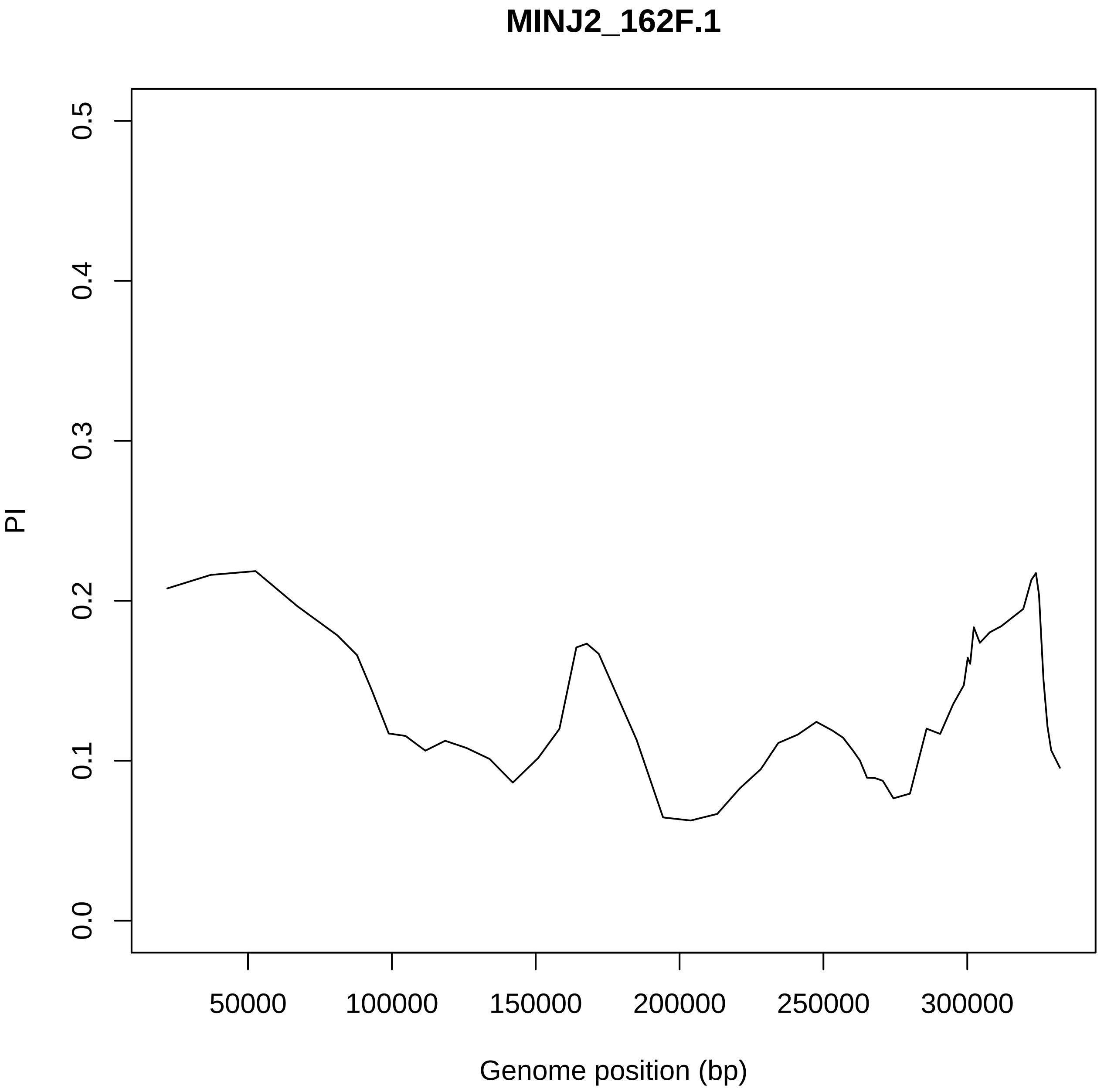
MINJ2_162F.1
PI
0.0
0.1
0.2
0.3
0.4
0.5
50000
100000
150000
200000
250000
300000
Genome position (bp)

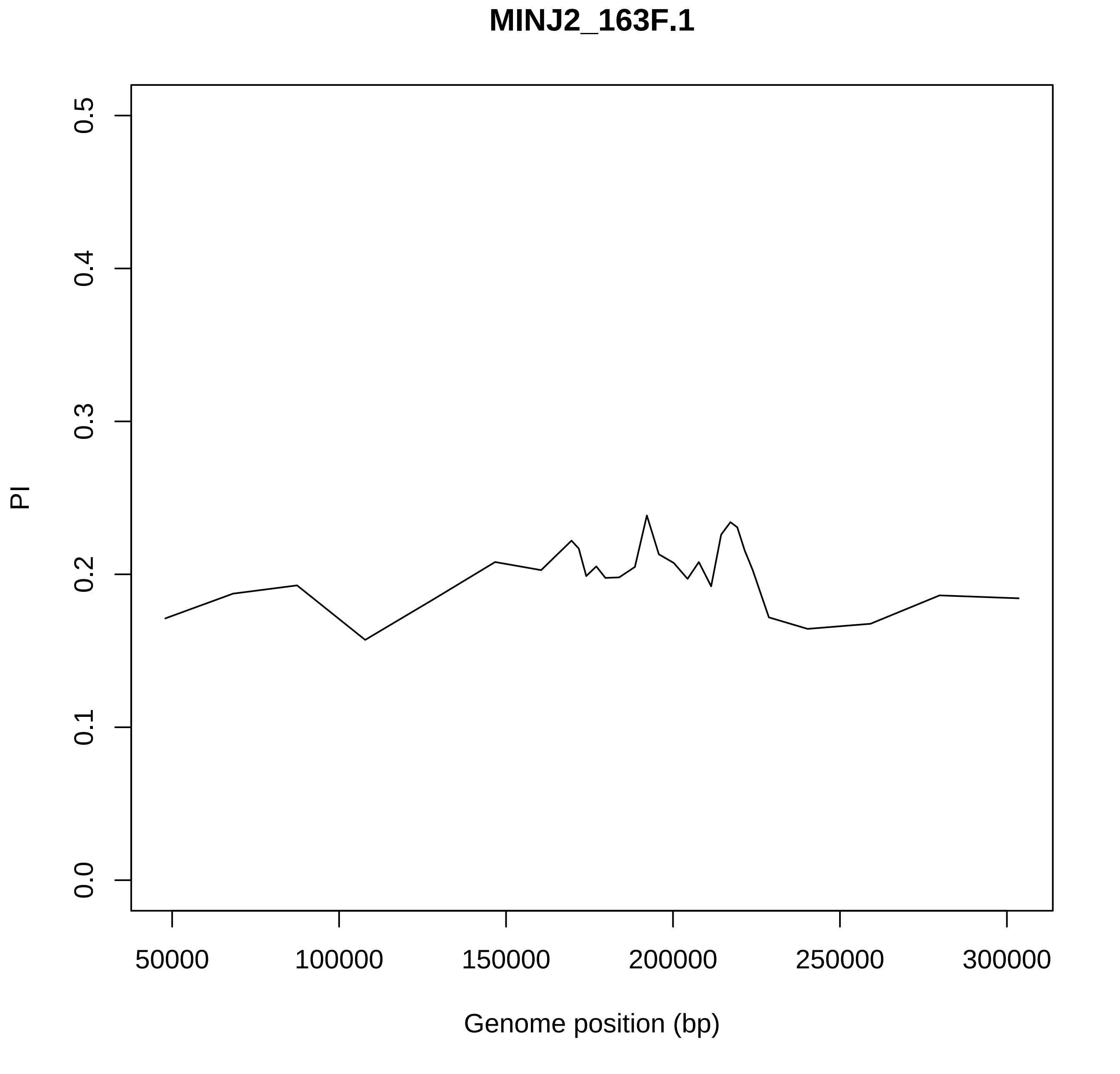
MINJ2_163F.1
PI
0.0
0.1
0.2
0.3
0.4
0.5
50000
100000
150000
200000
250000
300000
Genome position (bp)

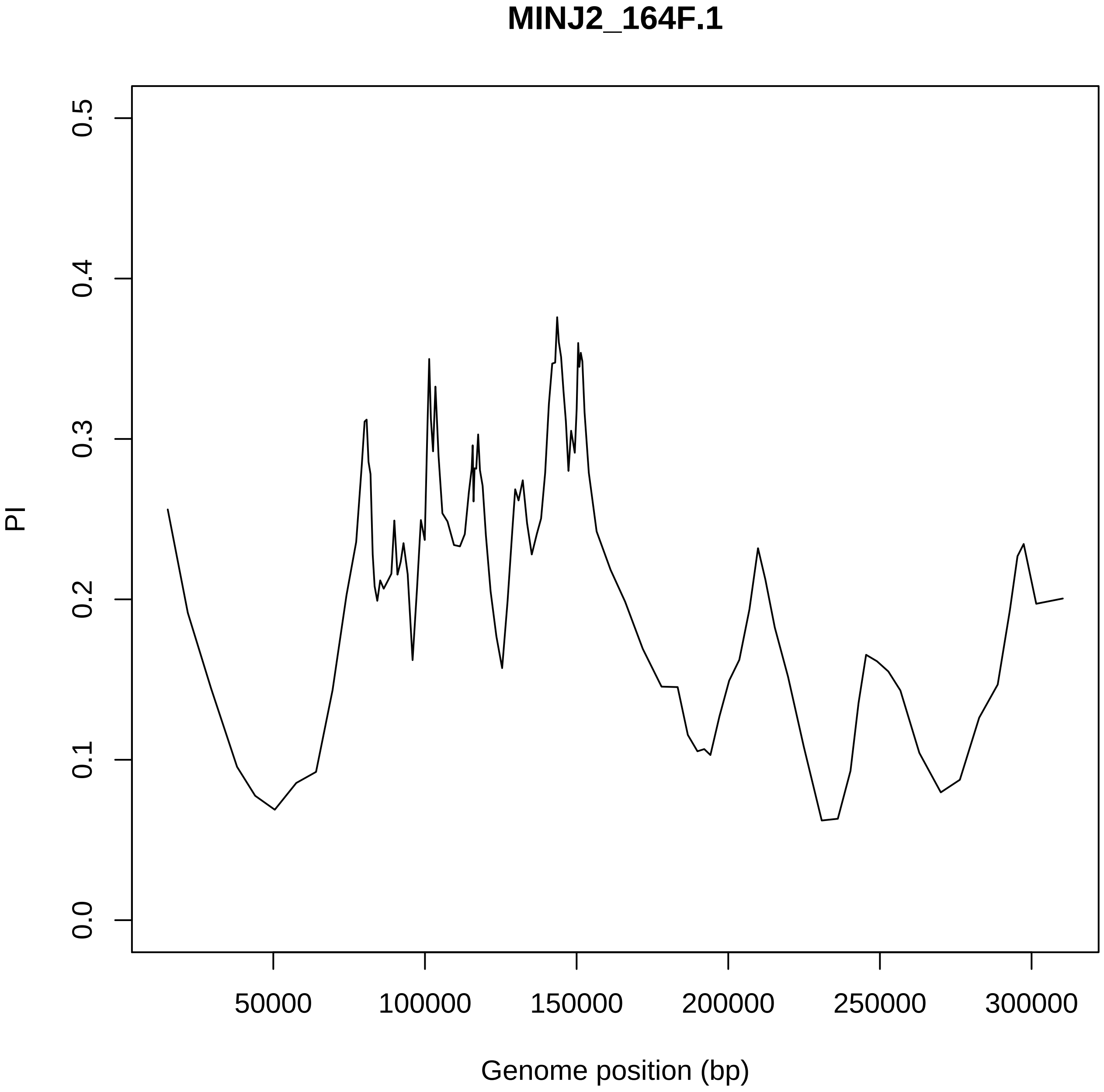
MINJ2_164F.1
PI
0.0
0.1
0.2
0.3
0.4
0.5
50000
100000
150000
200000
250000
300000
Genome position (bp)

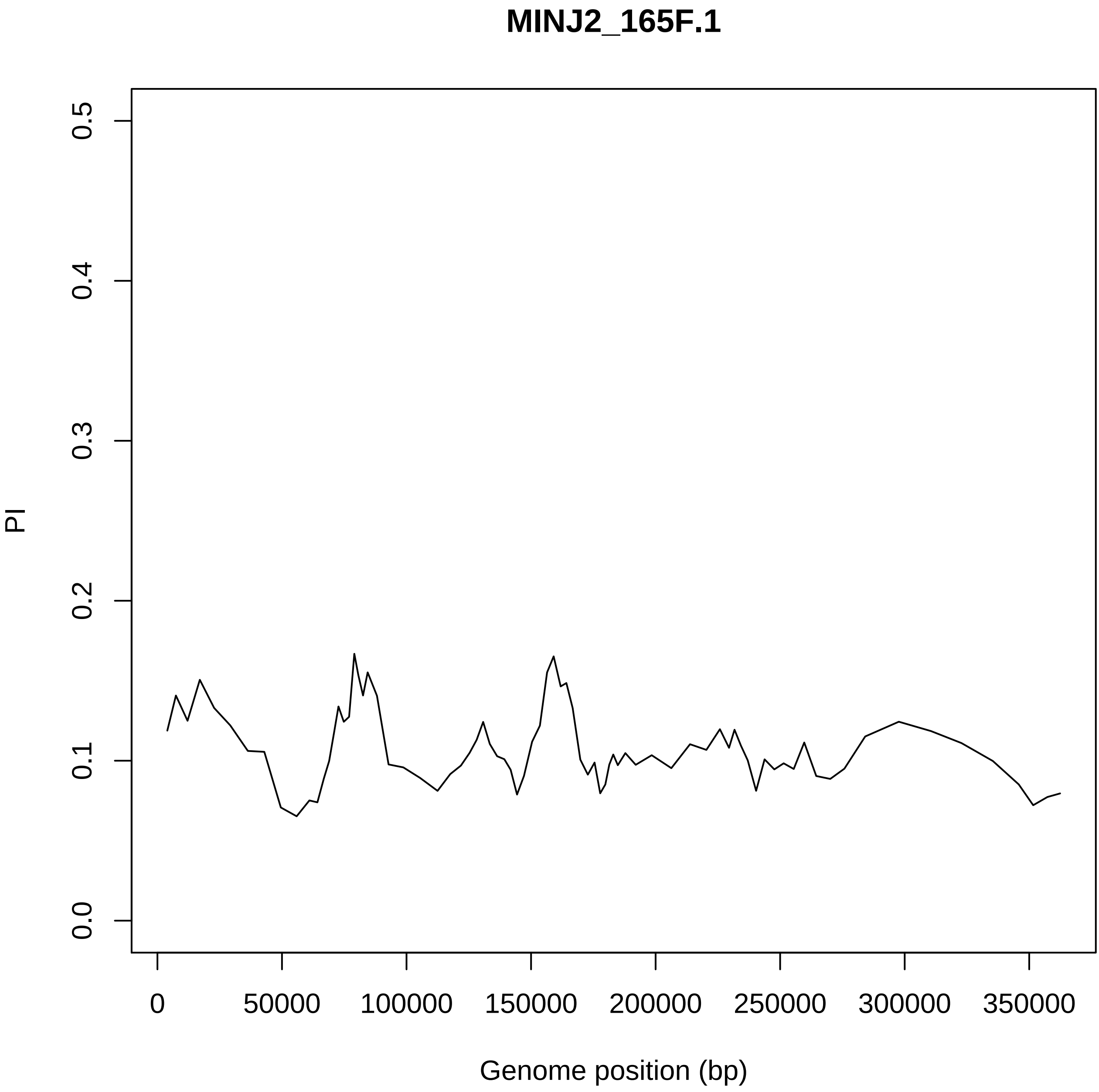

MINJ2_165F.1
PI
0.0
0.1
0.2
0.3
0.4
0.5
0
50000
100000
150000
200000
250000
300000
350000
Genome position (bp)

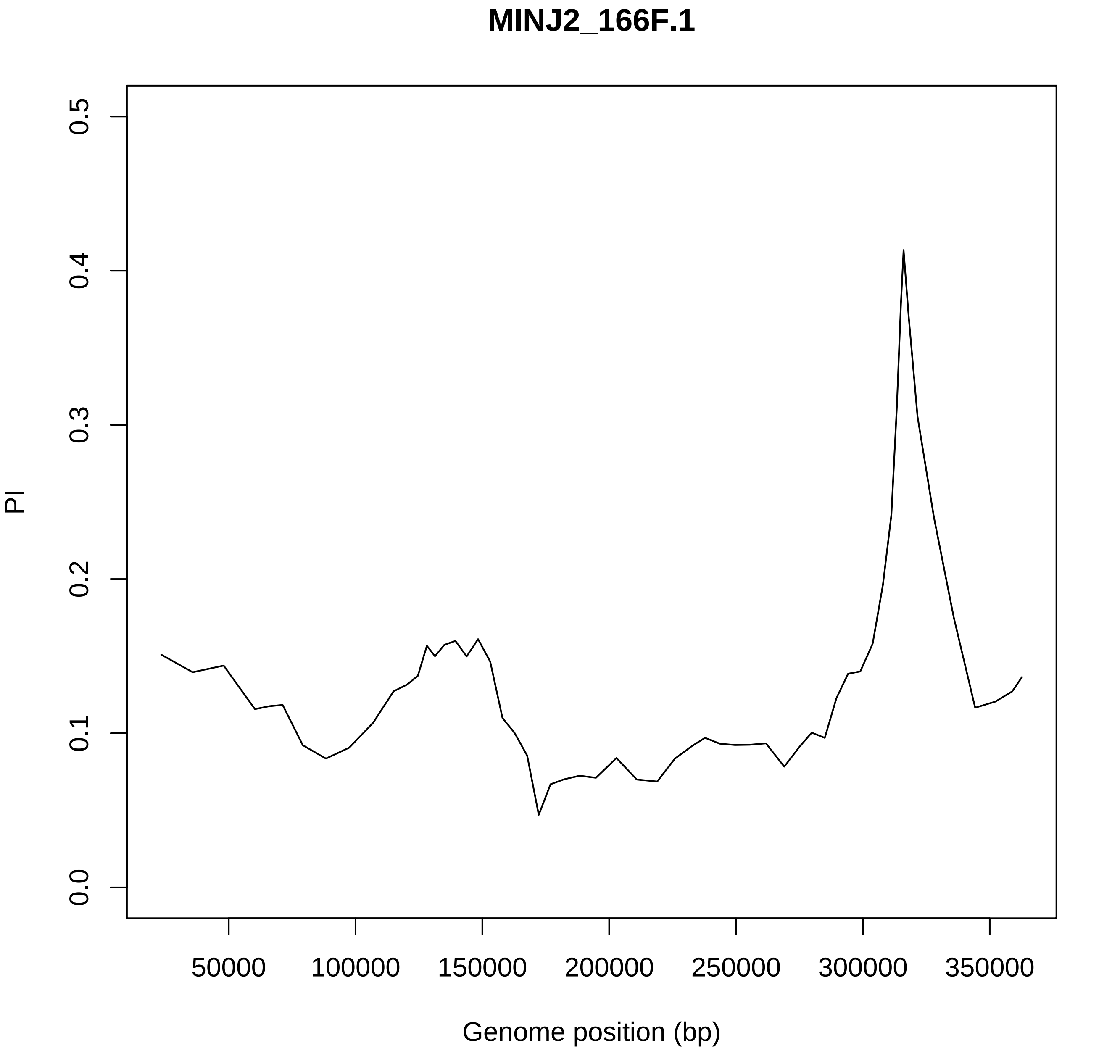
MINJ2_166F.1
PI
0.0
0.1
0.2
0.3
0.4
0.5
50000
100000
150000
200000
250000
300000
350000
Genome position (bp)

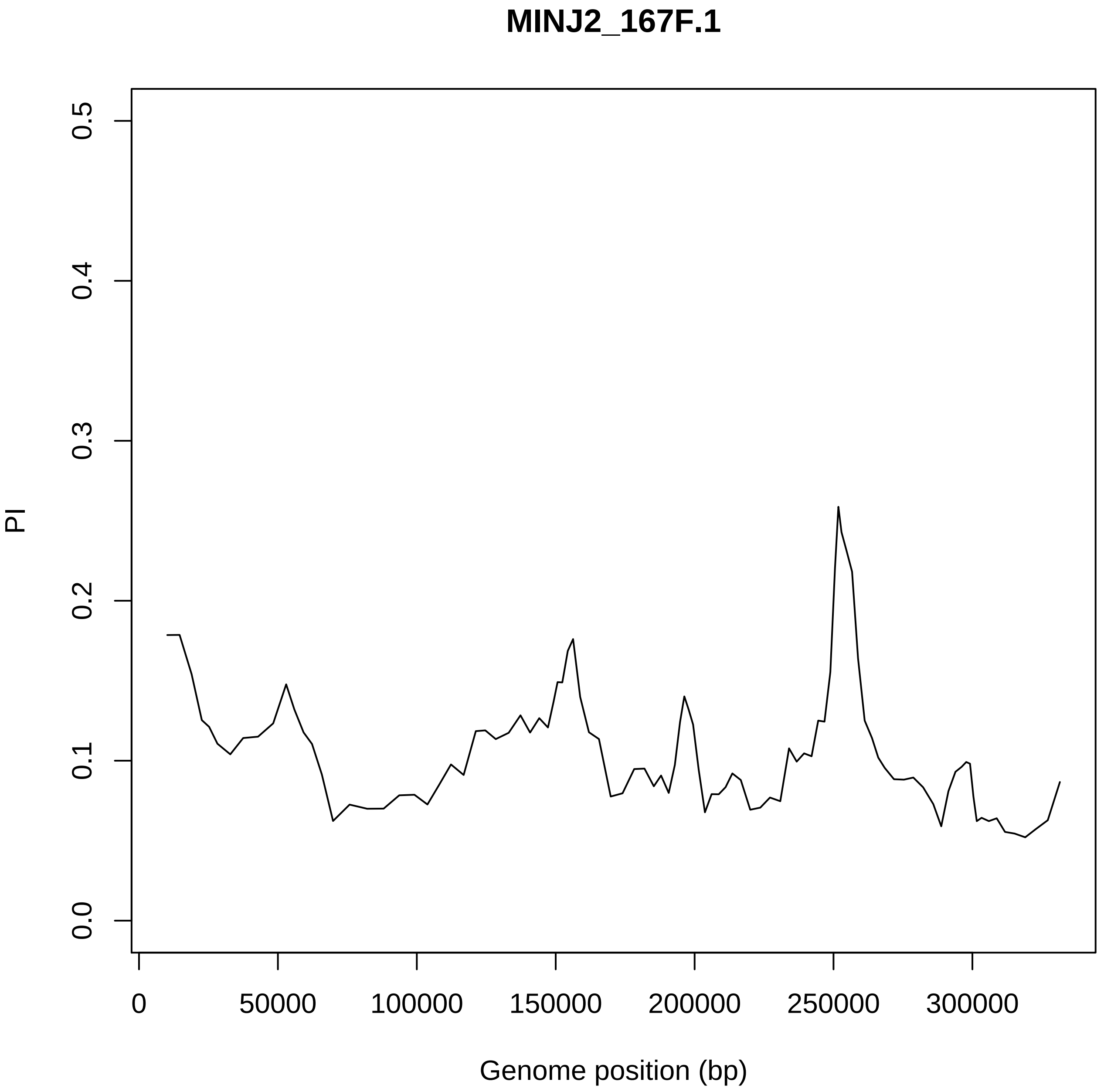

MINJ2_167F.1
PI
0.0
0.1
0.2
0.3
0.4
0.5
0
50000
100000
150000
200000
250000
300000
Genome position (bp)

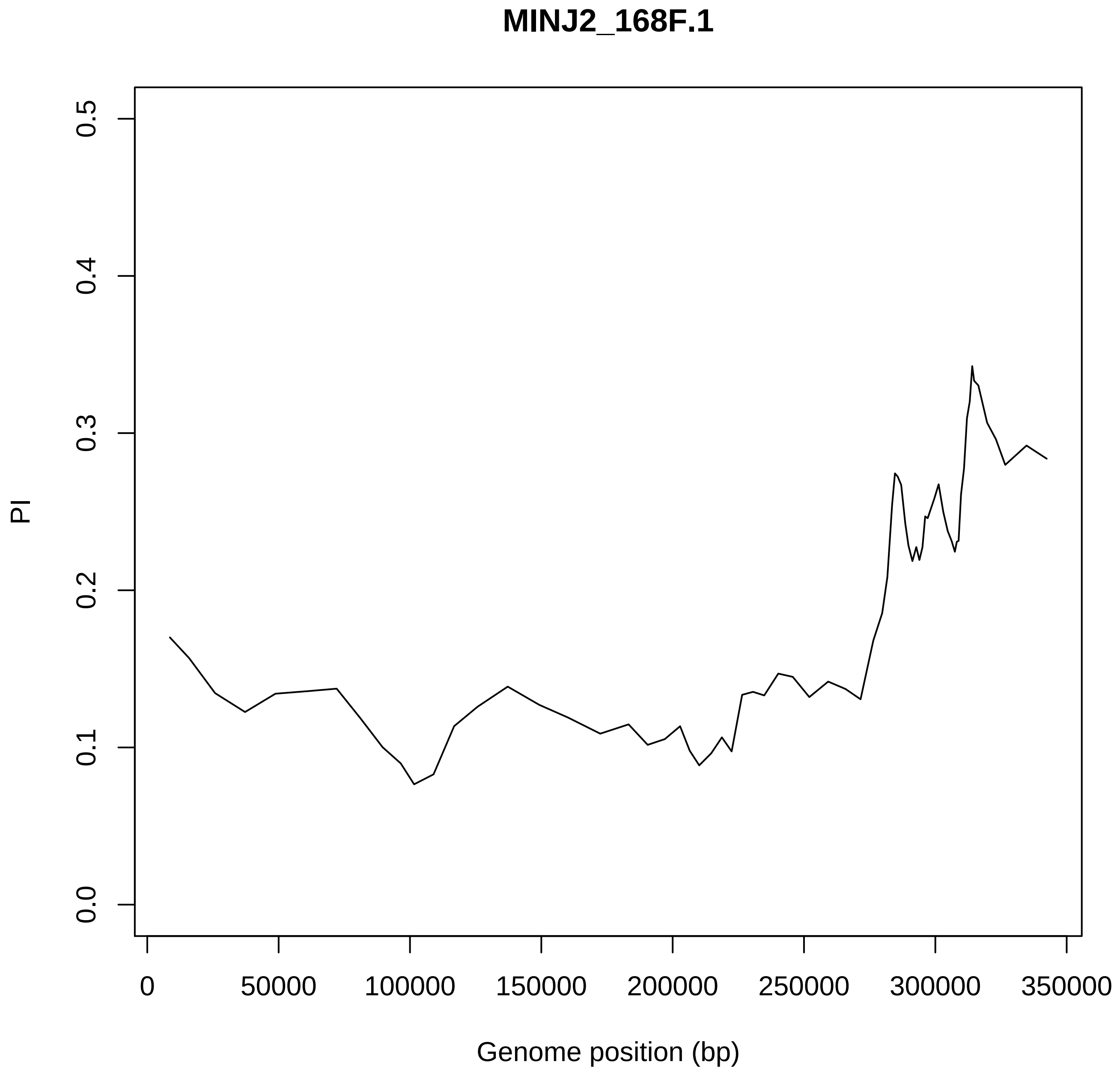
MINJ2_168F.1
PI
0.0
0.1
0.2
0.3
0.4
0.5
0
50000
100000
150000
200000
250000
300000
350000
Genome position (bp)

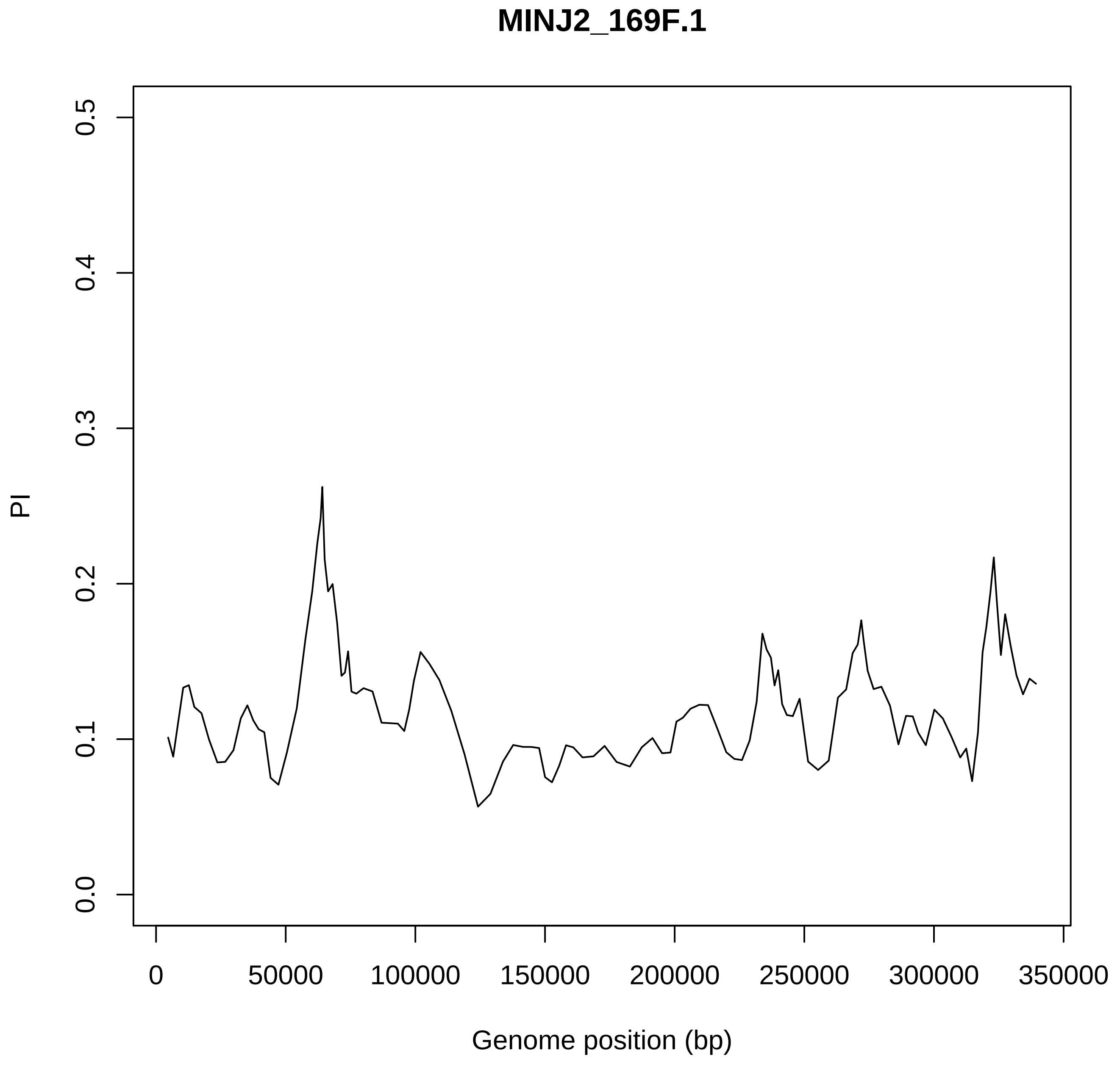
MINJ2_169F.1
PI
0.0
0.1
0.2
0.3
0.4
0.5
0
50000
100000
150000
200000
250000
300000
350000
Genome position (bp)

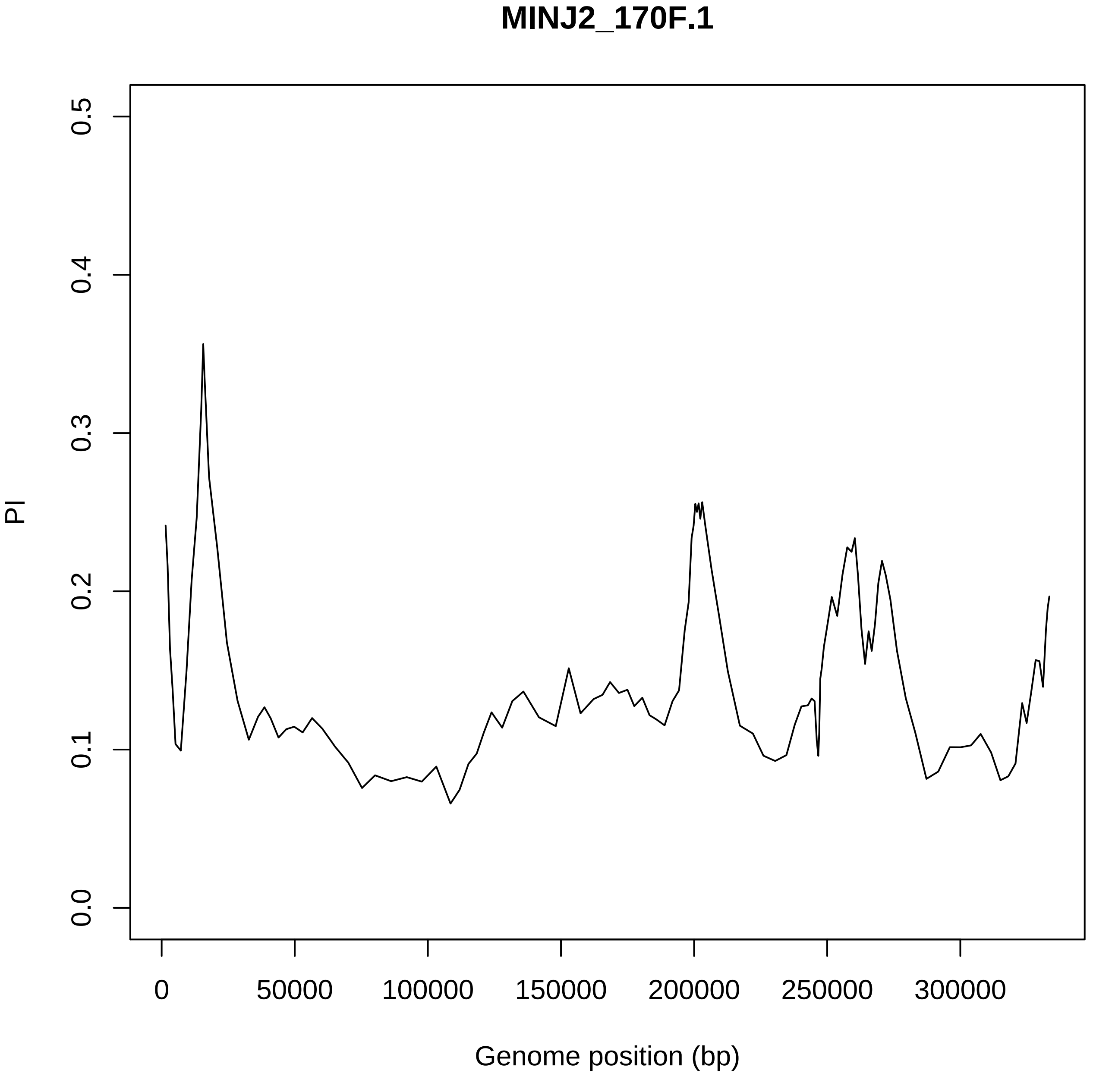
MINJ2_170F.1
PI
0.0
0.1
0.2
0.3
0.4
0.5
0
50000
100000
150000
200000
250000
300000
Genome position (bp)

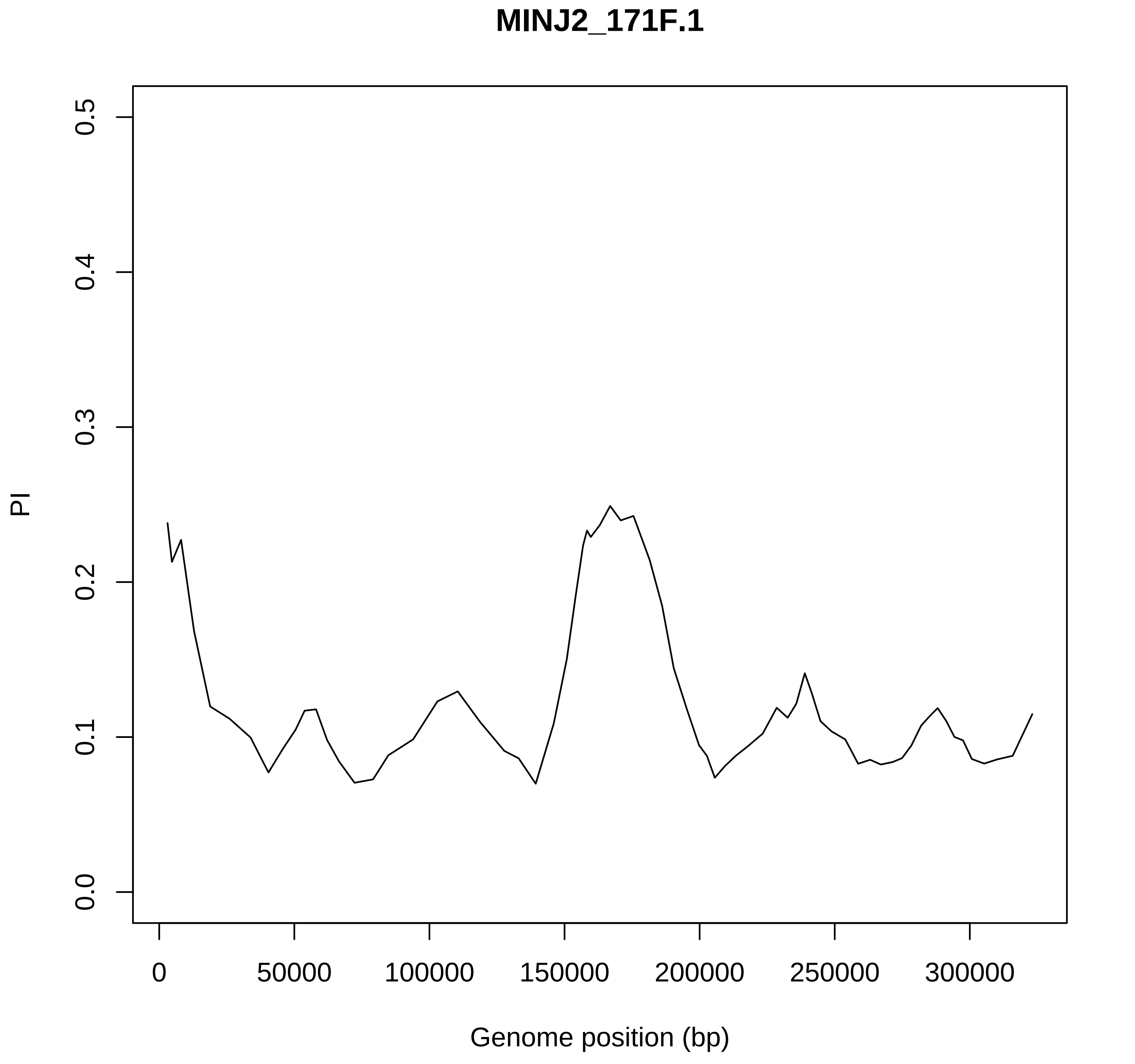

MINJ2_171F.1
PI
0.0
0.1
0.2
0.3
0.4
0.5
0
50000
100000
150000
200000
250000
300000
Genome position (bp)

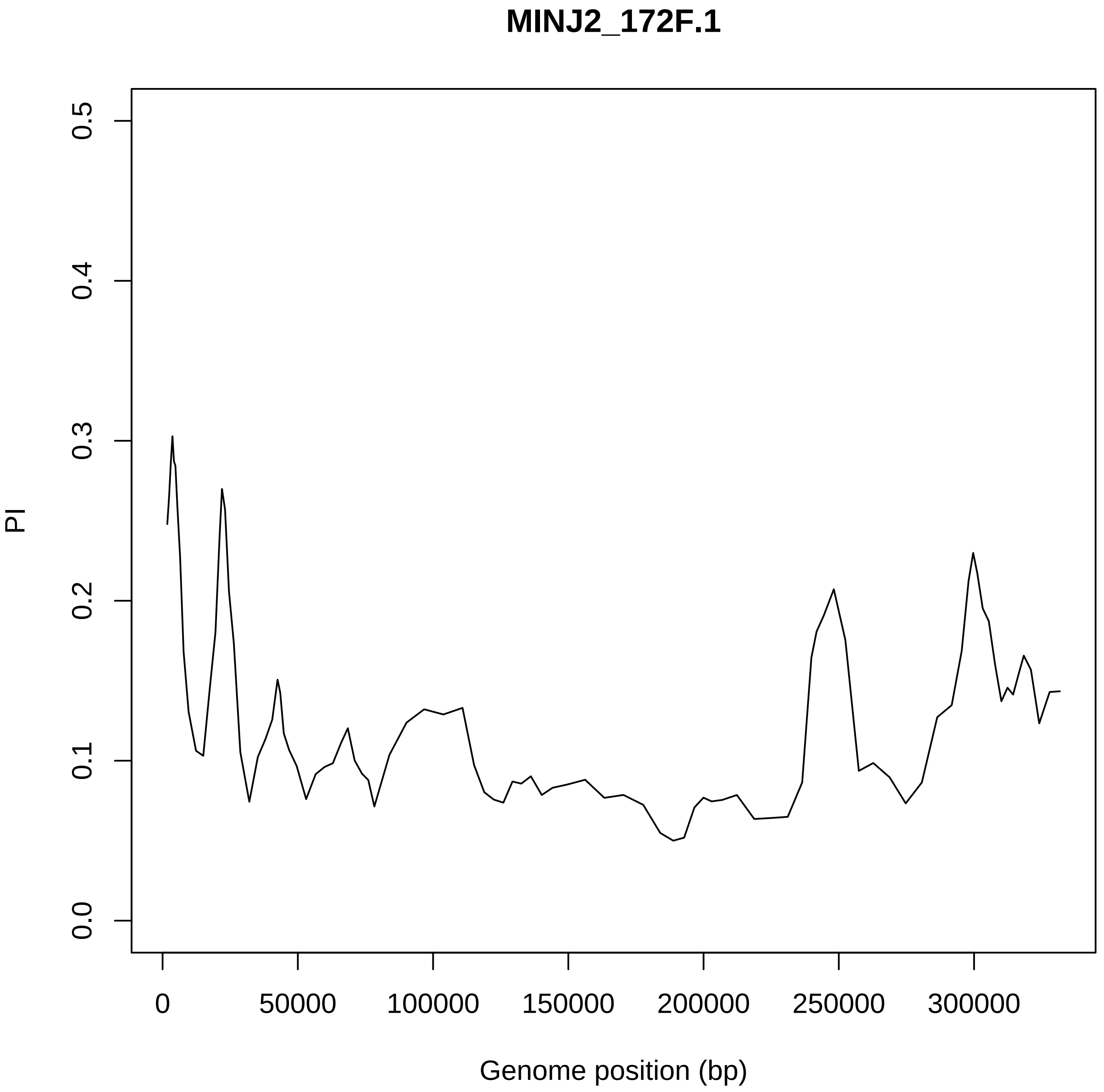
MINJ2_172F.1
PI
0.0
0.1
0.2
0.3
0.4
0.5
0
50000
100000
150000
200000
250000
300000
Genome position (bp)

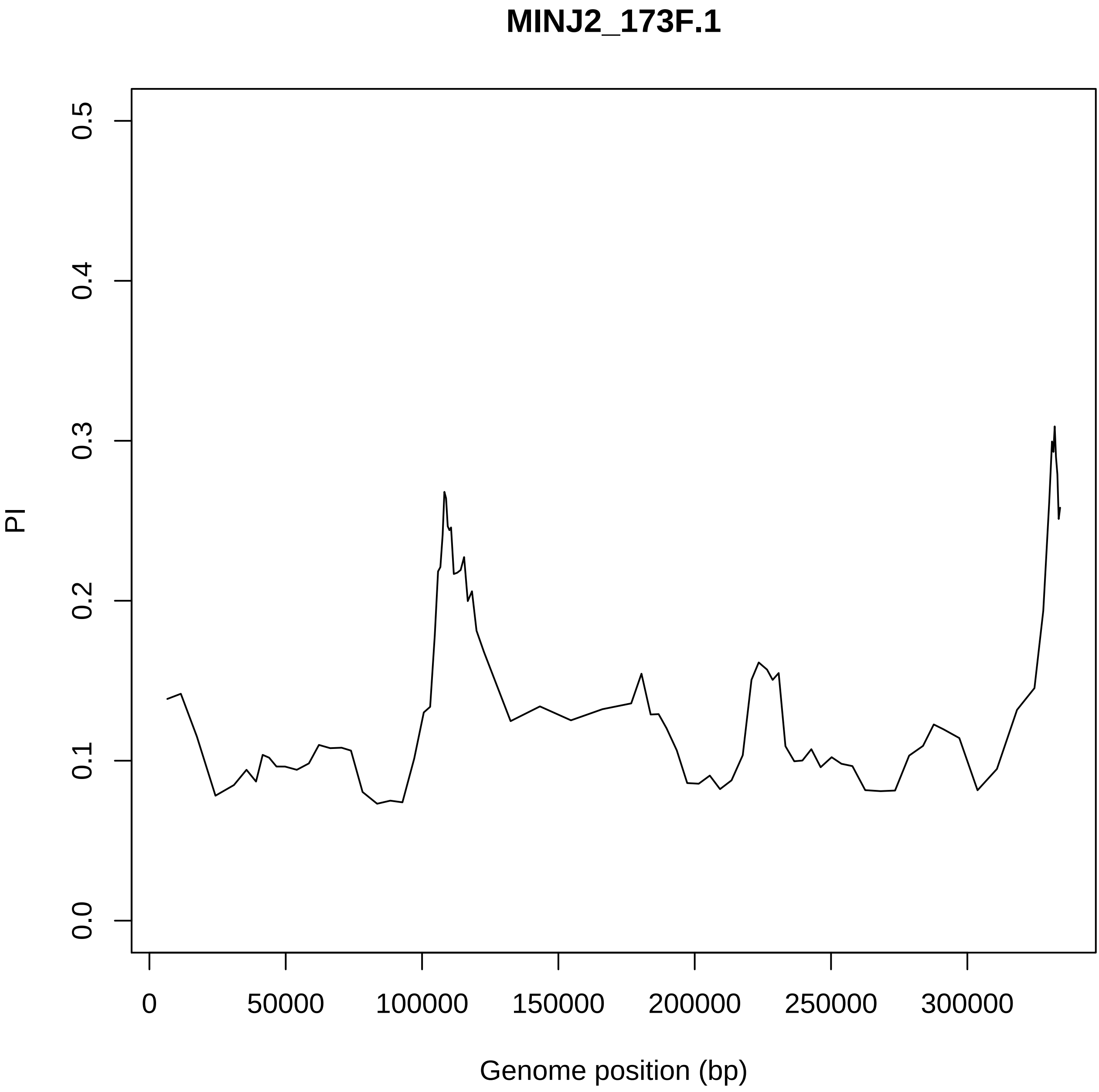
MINJ2_173F.1
PI
0.0
0.1
0.2
0.3
0.4
0.5
0
50000
100000
150000
200000
250000
300000
Genome position (bp)

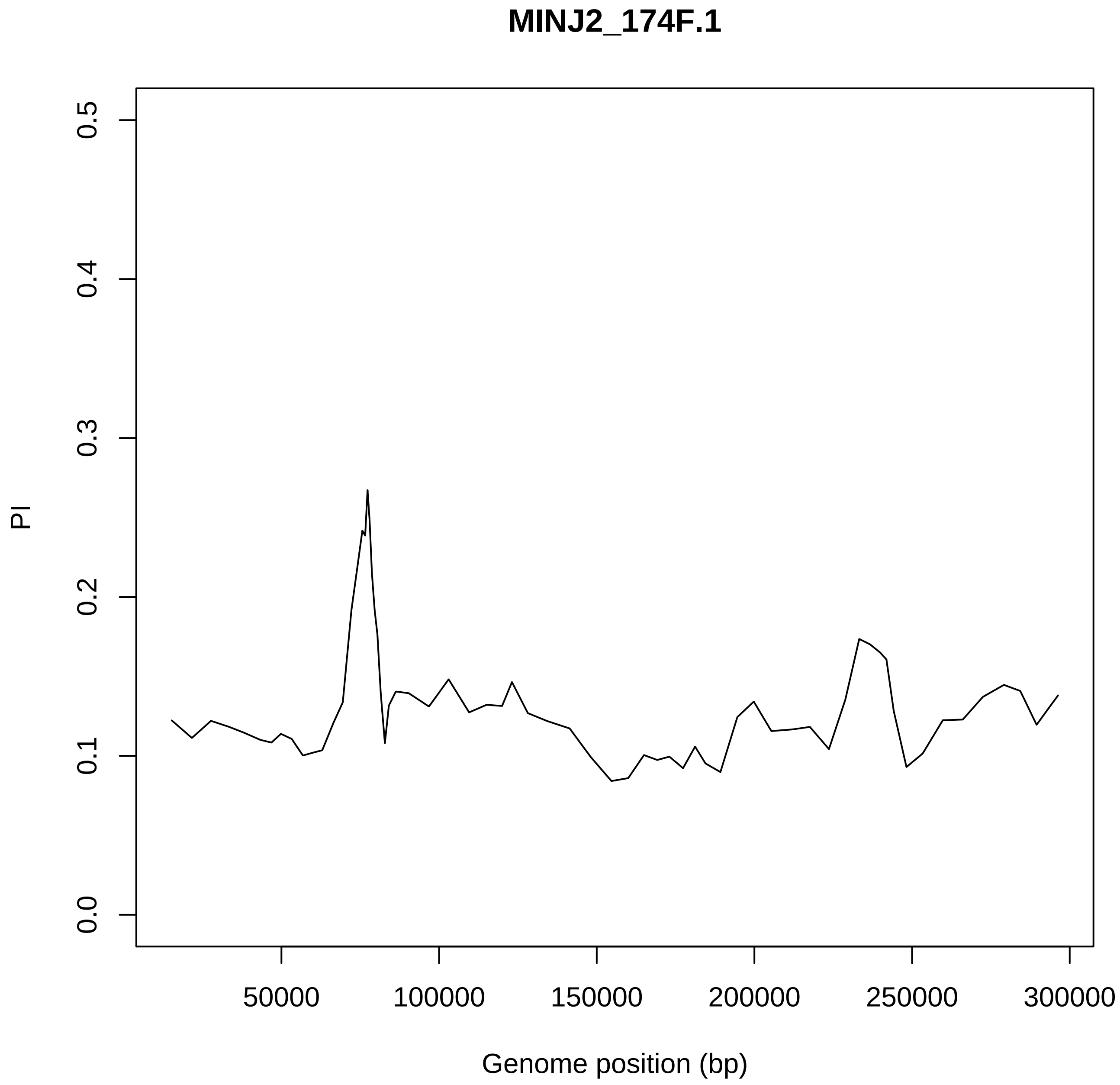

MINJ2_174F.1
PI
0.0
0.1
0.2
0.3
0.4
0.5
50000
100000
150000
200000
250000
300000
Genome position (bp)

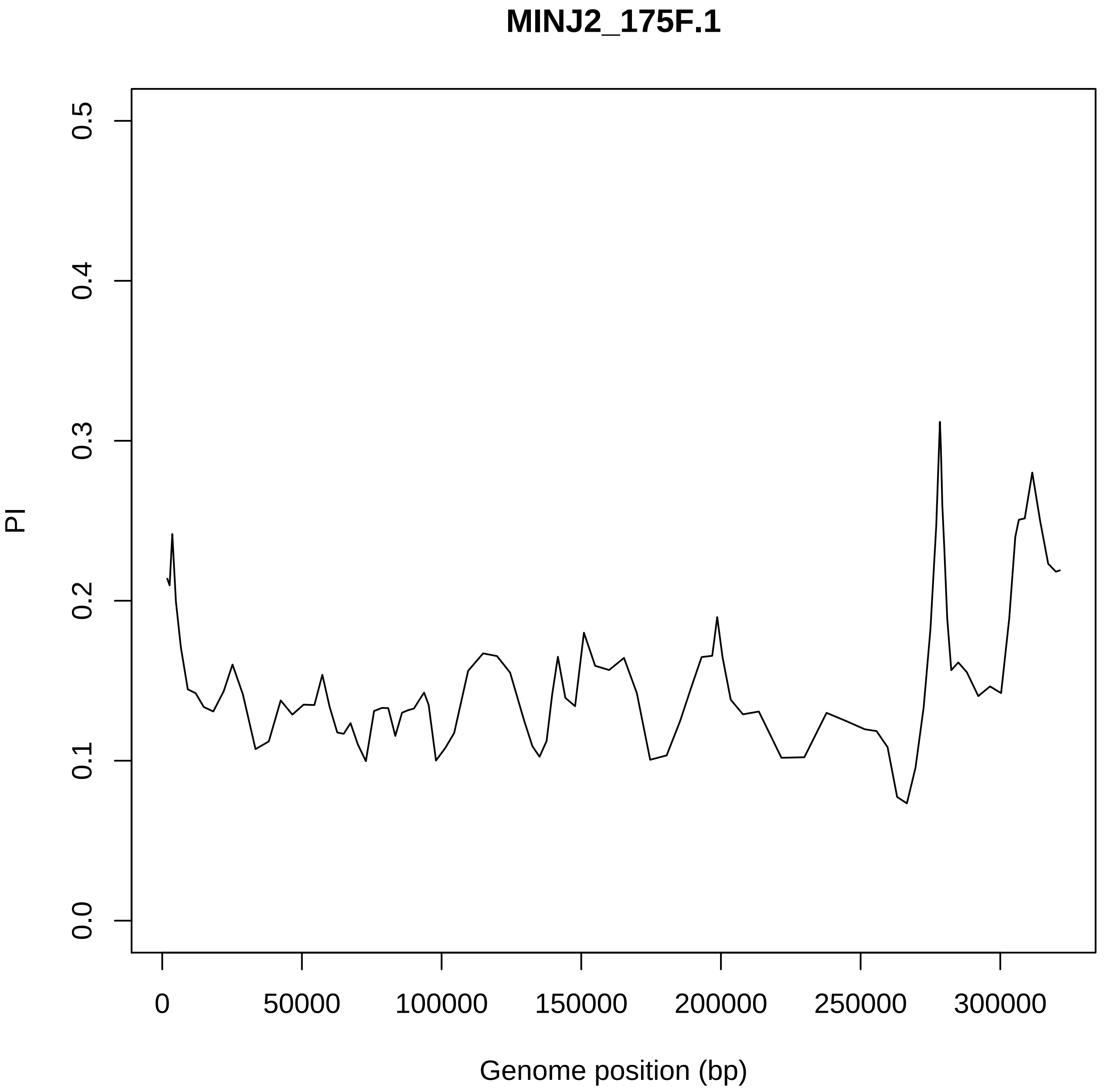

MINJ2_175F.1
PI
0.0
0.1
0.2
0.3
0.4
0.5
0
50000
100000
150000
200000
250000
300000
Genome position (bp)

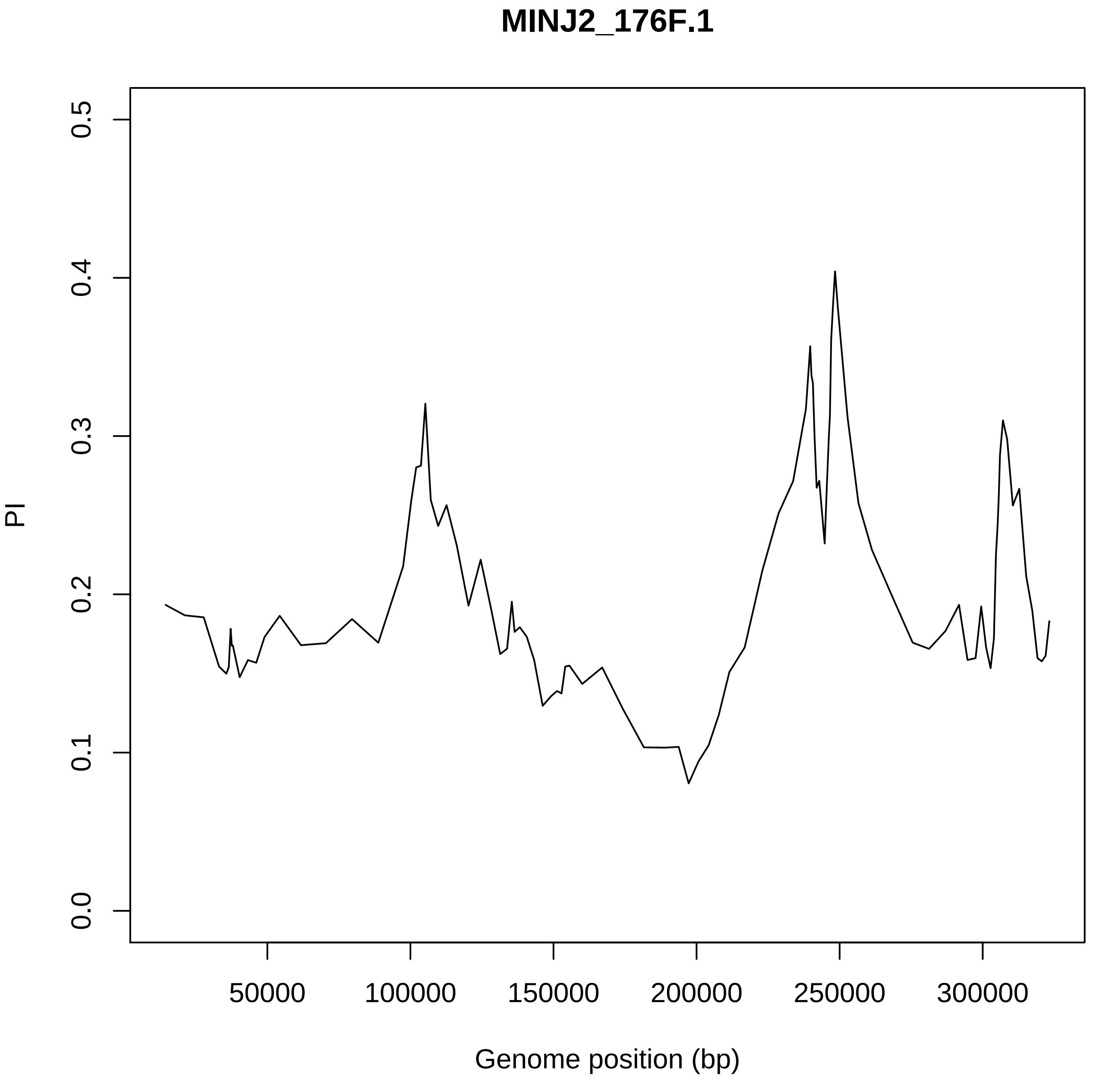

MINJ2_176F.1
PI
0.0
0.1
0.2
0.3
0.4
0.5
50000
100000
150000
200000
250000
300000
Genome position (bp)

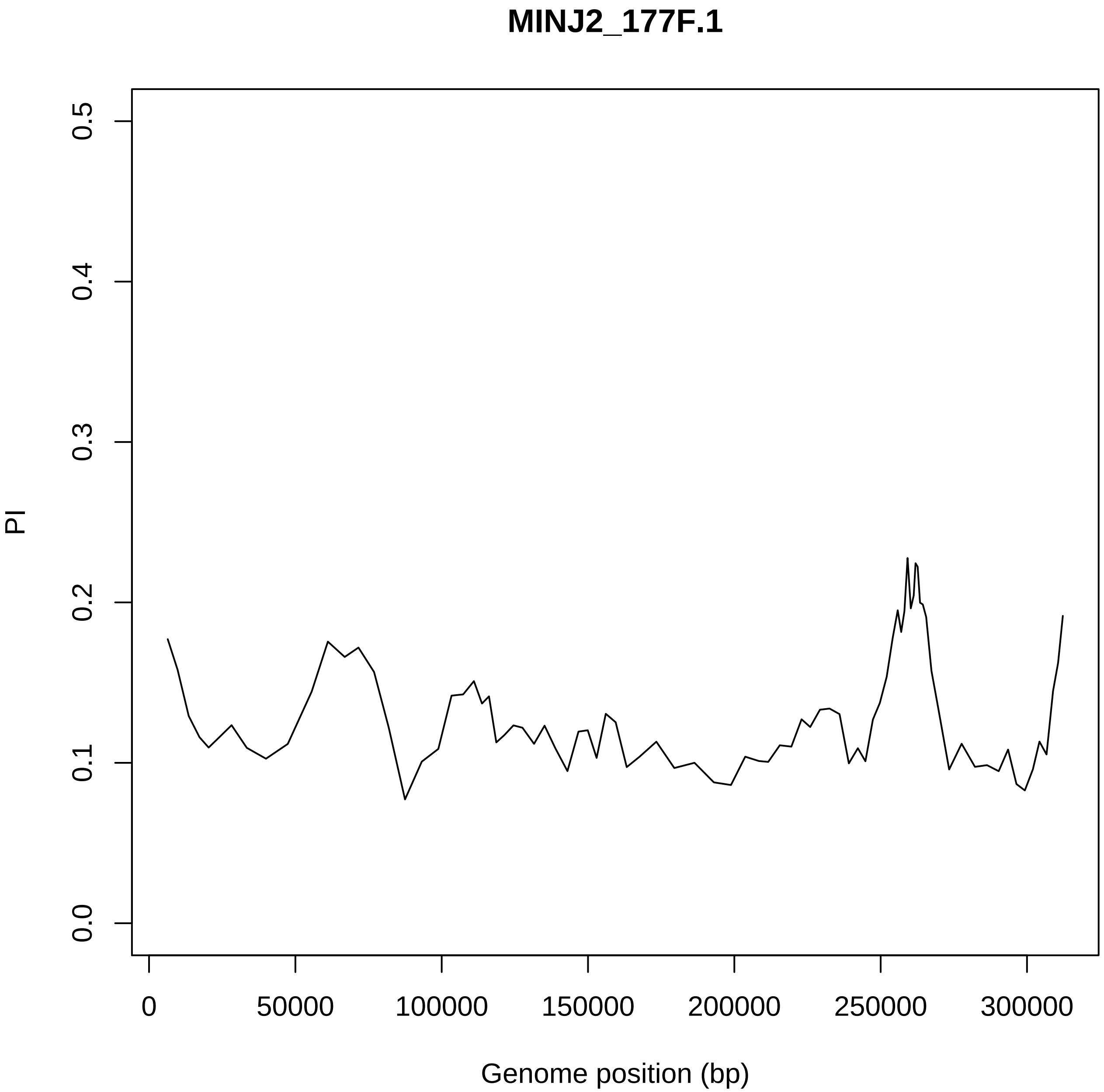

MINJ2_177F.1
PI
0.0
0.1
0.2
0.3
0.4
0.5
0
50000
100000
150000
200000
250000
300000
Genome position (bp)

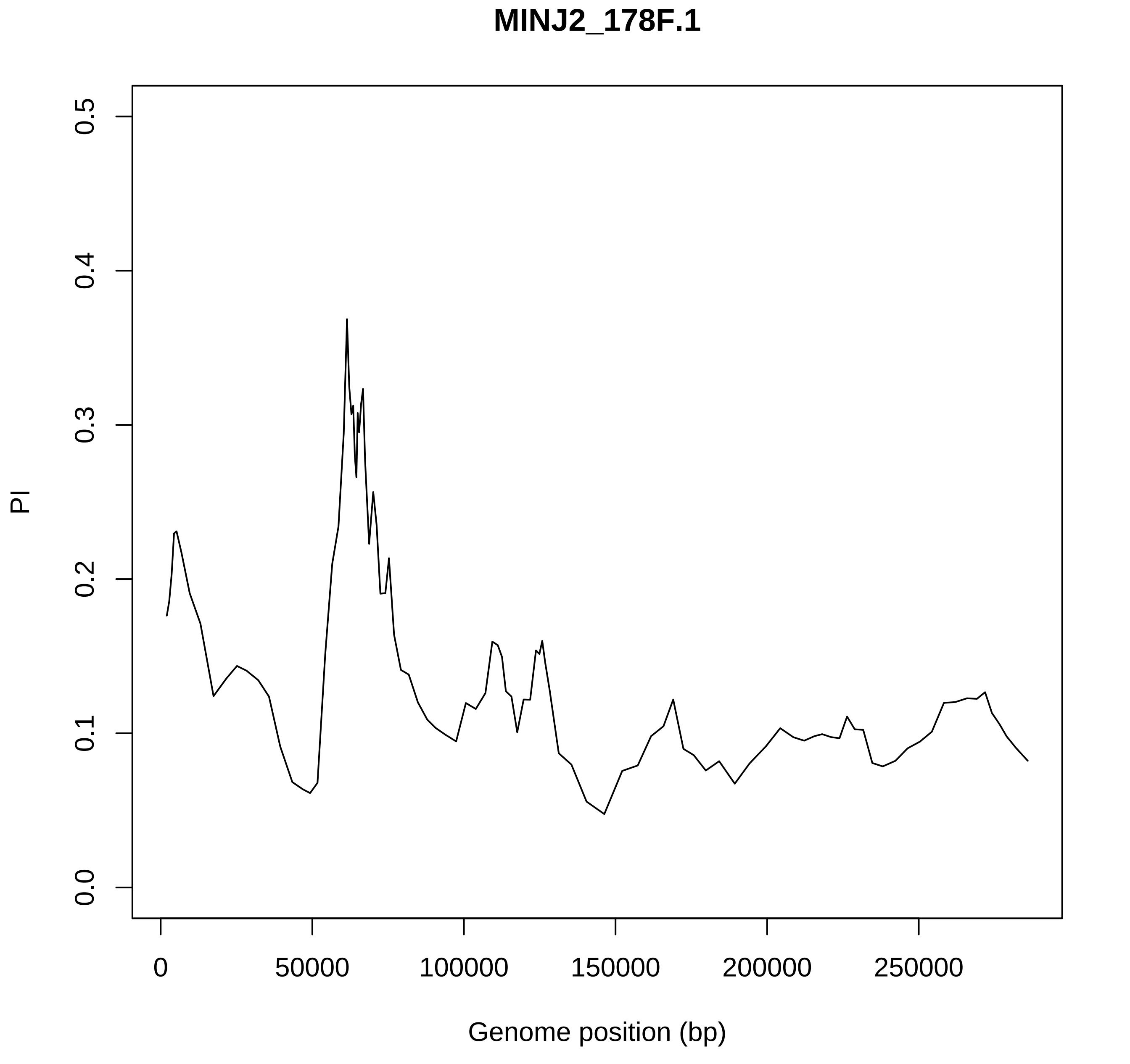

MINJ2_178F.1
PI
0.0
0.1
0.2
0.3
0.4
0.5
0
50000
100000
150000
200000
250000
Genome position (bp)

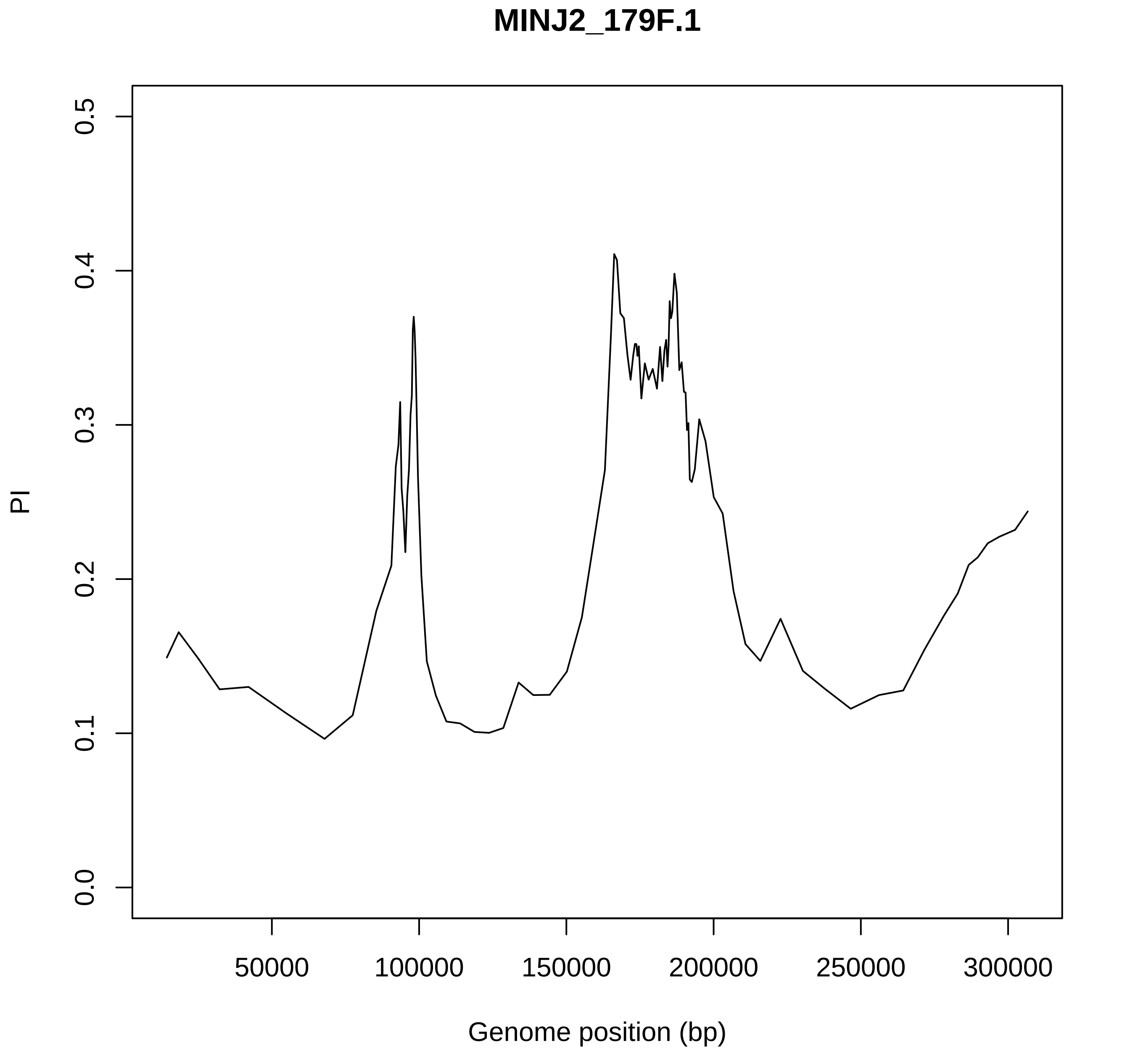

MINJ2_179F.1
PI
0.0
0.1
0.2
0.3
0.4
0.5
50000
100000
150000
200000
250000
300000
Genome position (bp)

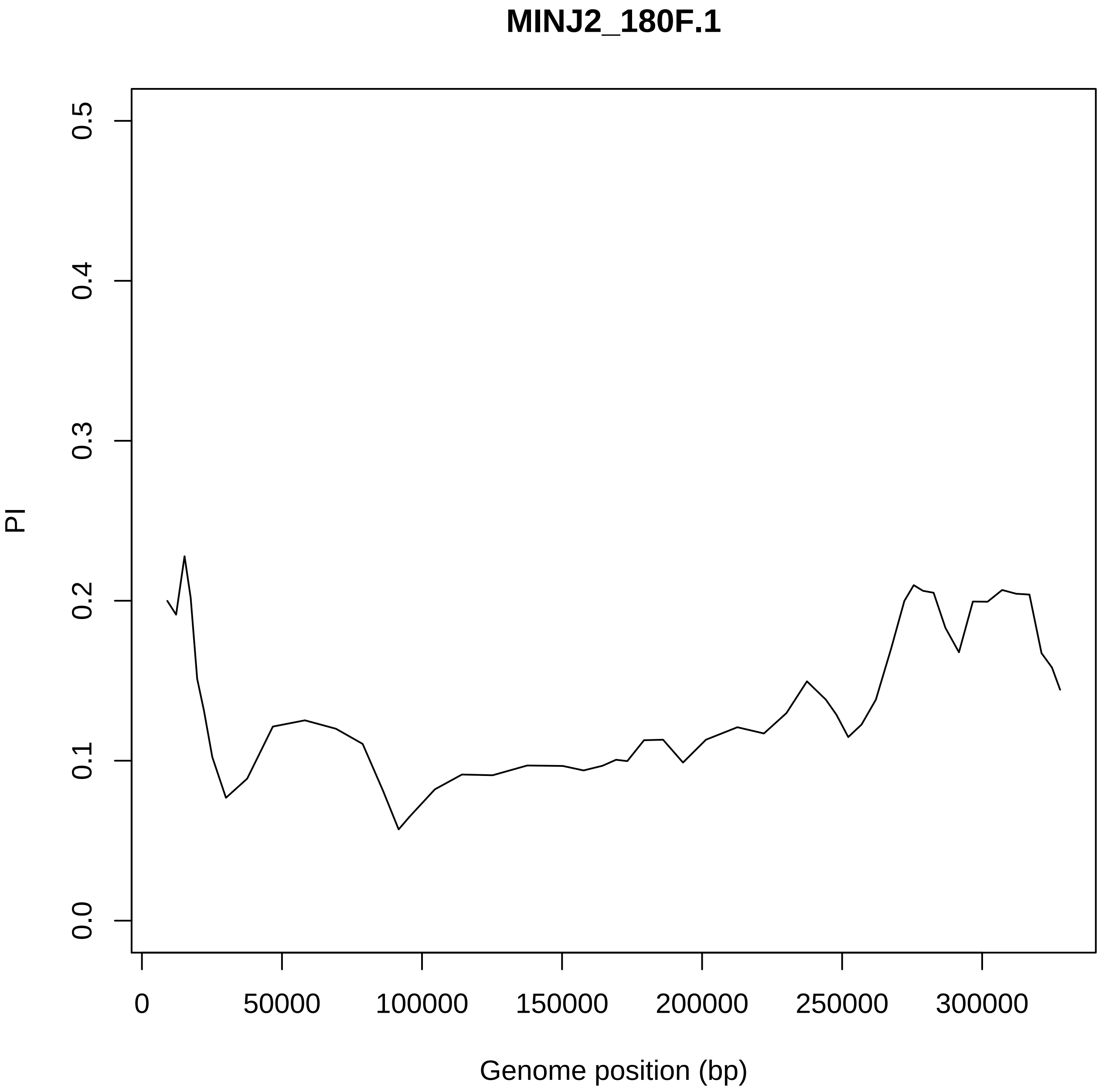
MINJ2_180F.1
PI
0.0
0.1
0.2
0.3
0.4
0.5
0
50000
100000
150000
200000
250000
300000
Genome position (bp)

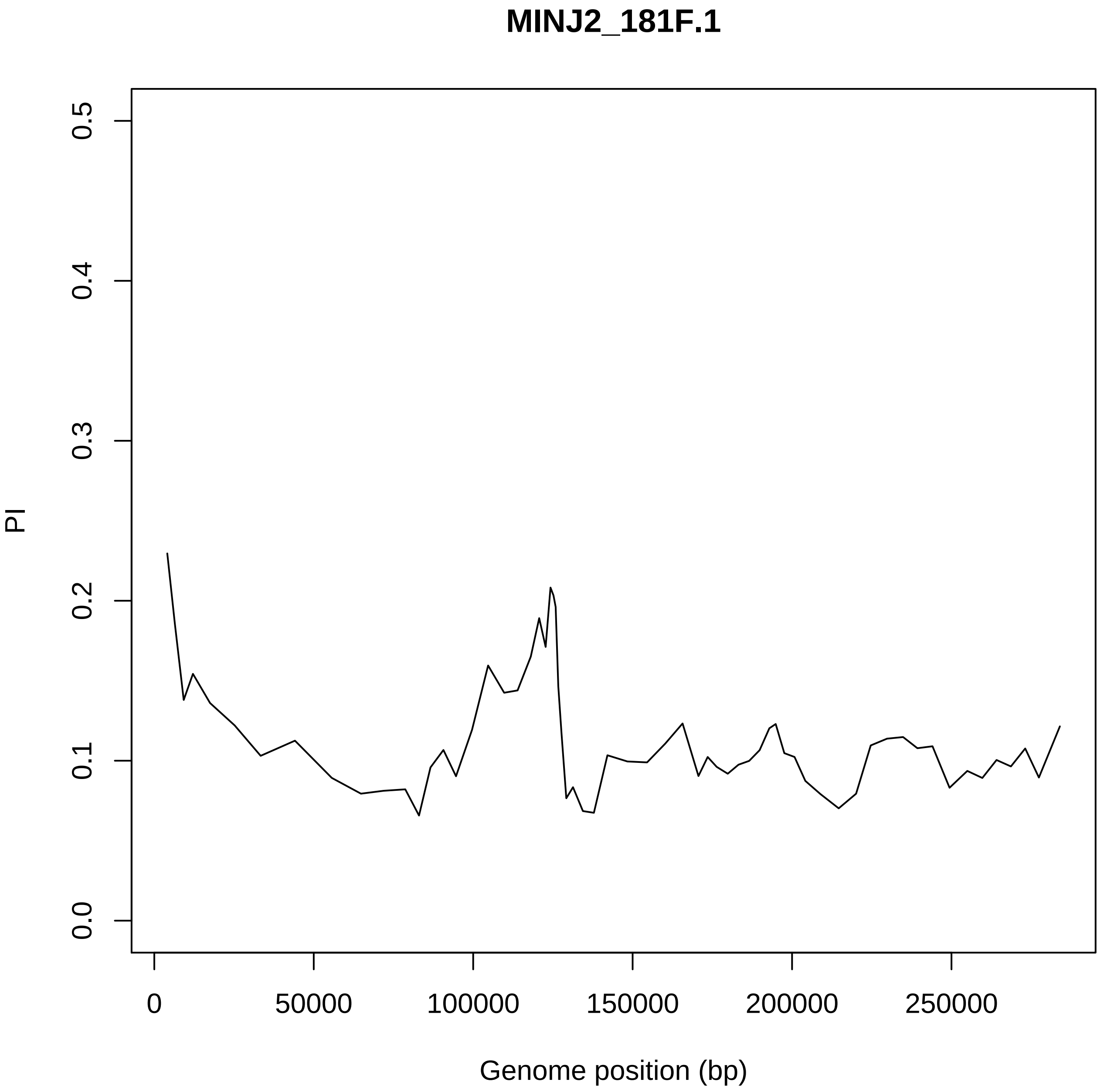
MINJ2_181F.1
PI
0.0
0.1
0.2
0.3
0.4
0.5
0
50000
100000
150000
200000
250000
Genome position (bp)

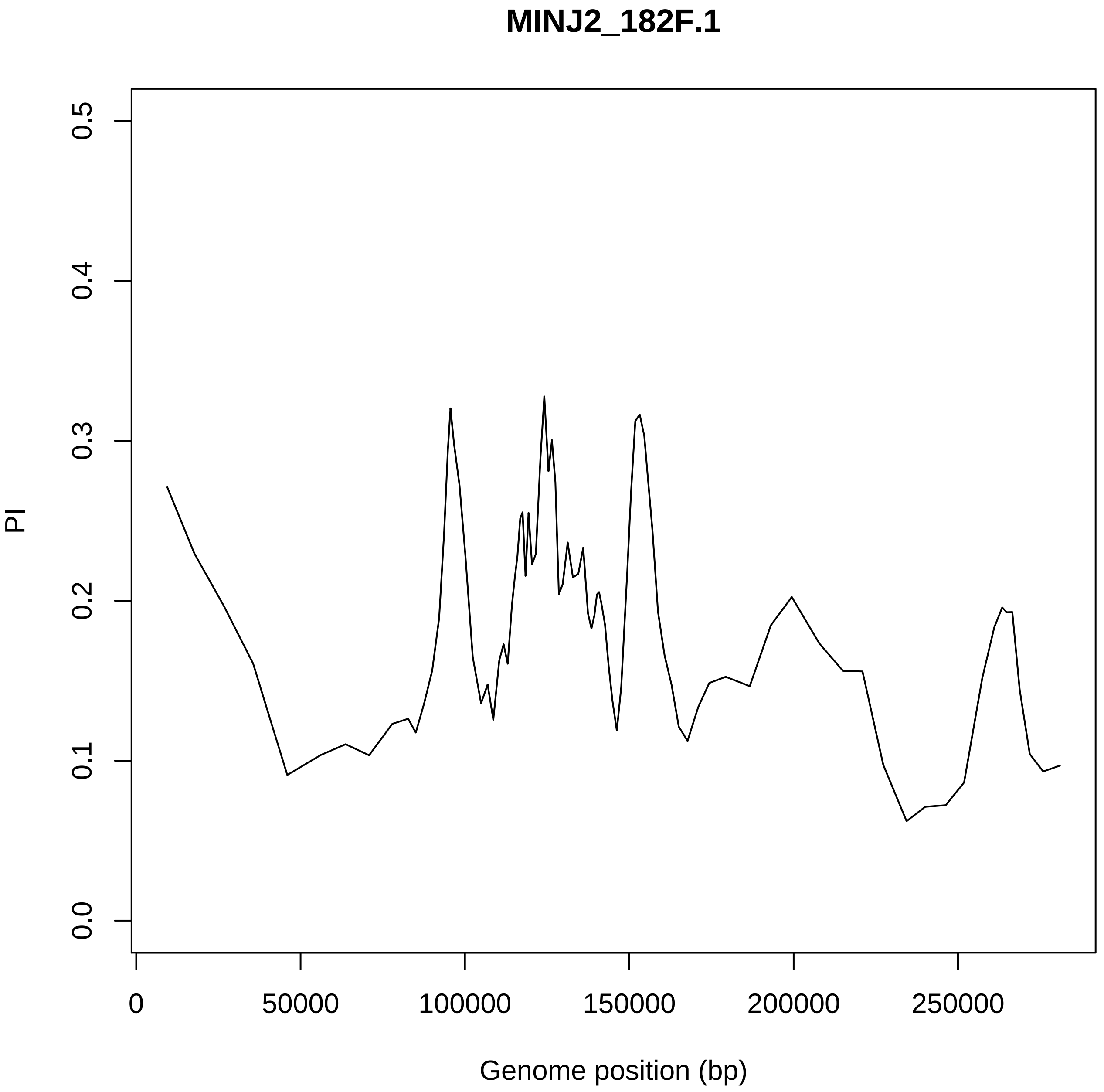
MINJ2_182F.1
PI
0.0
0.1
0.2
0.3
0.4
0.5
0
50000
100000
150000
200000
250000
Genome position (bp)

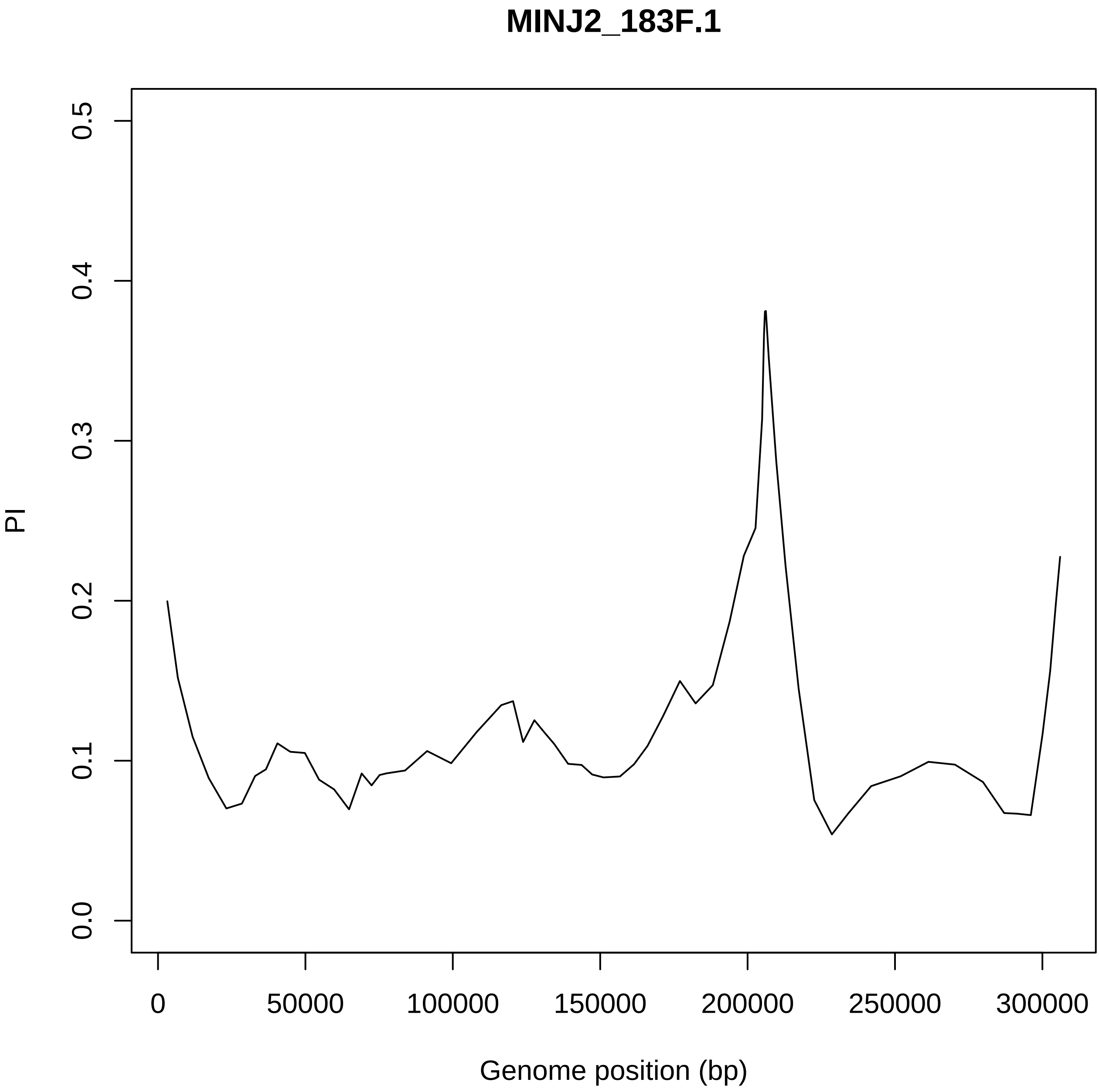

MINJ2_183F.1
PI
0.0
0.1
0.2
0.3
0.4
0.5
0
50000
100000
150000
200000
250000
300000
Genome position (bp)

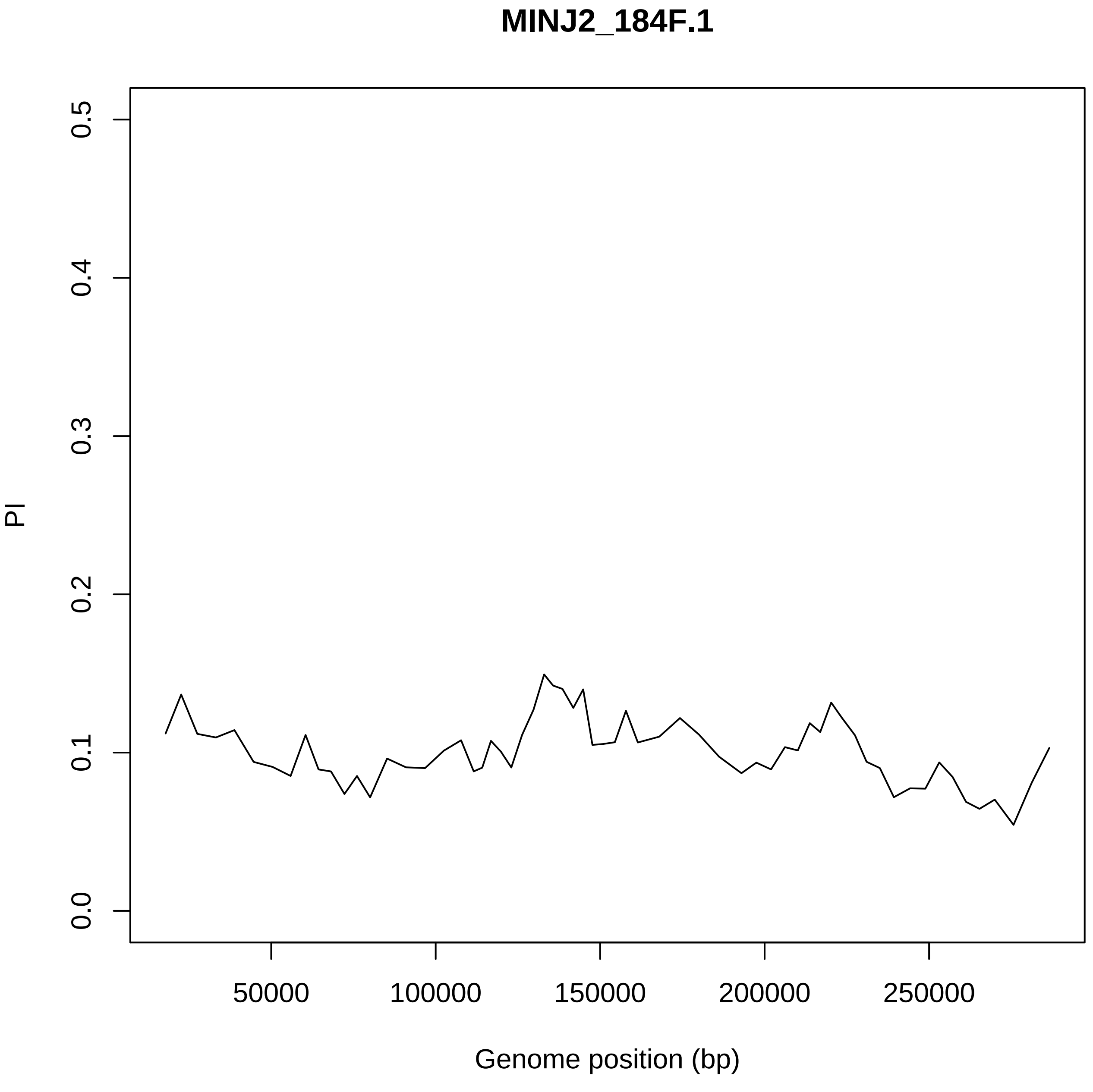

MINJ2_184F.1
PI
0.0
0.1
0.2
0.3
0.4
0.5
50000
100000
150000
200000
250000
Genome position (bp)

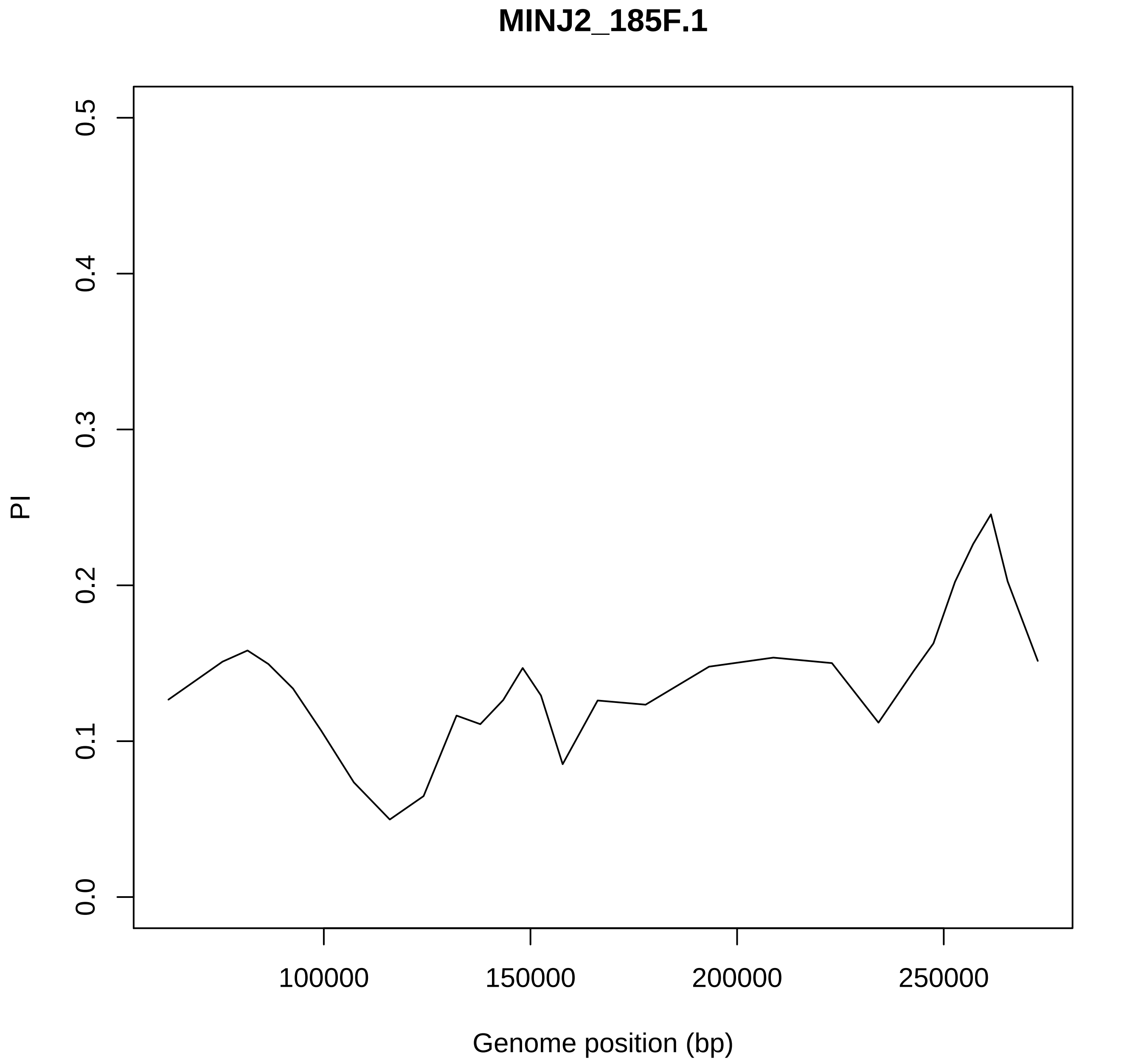
MINJ2_185F.1
PI
0.0
0.1
0.2
0.3
0.4
0.5
100000
150000
200000
250000
Genome position (bp)

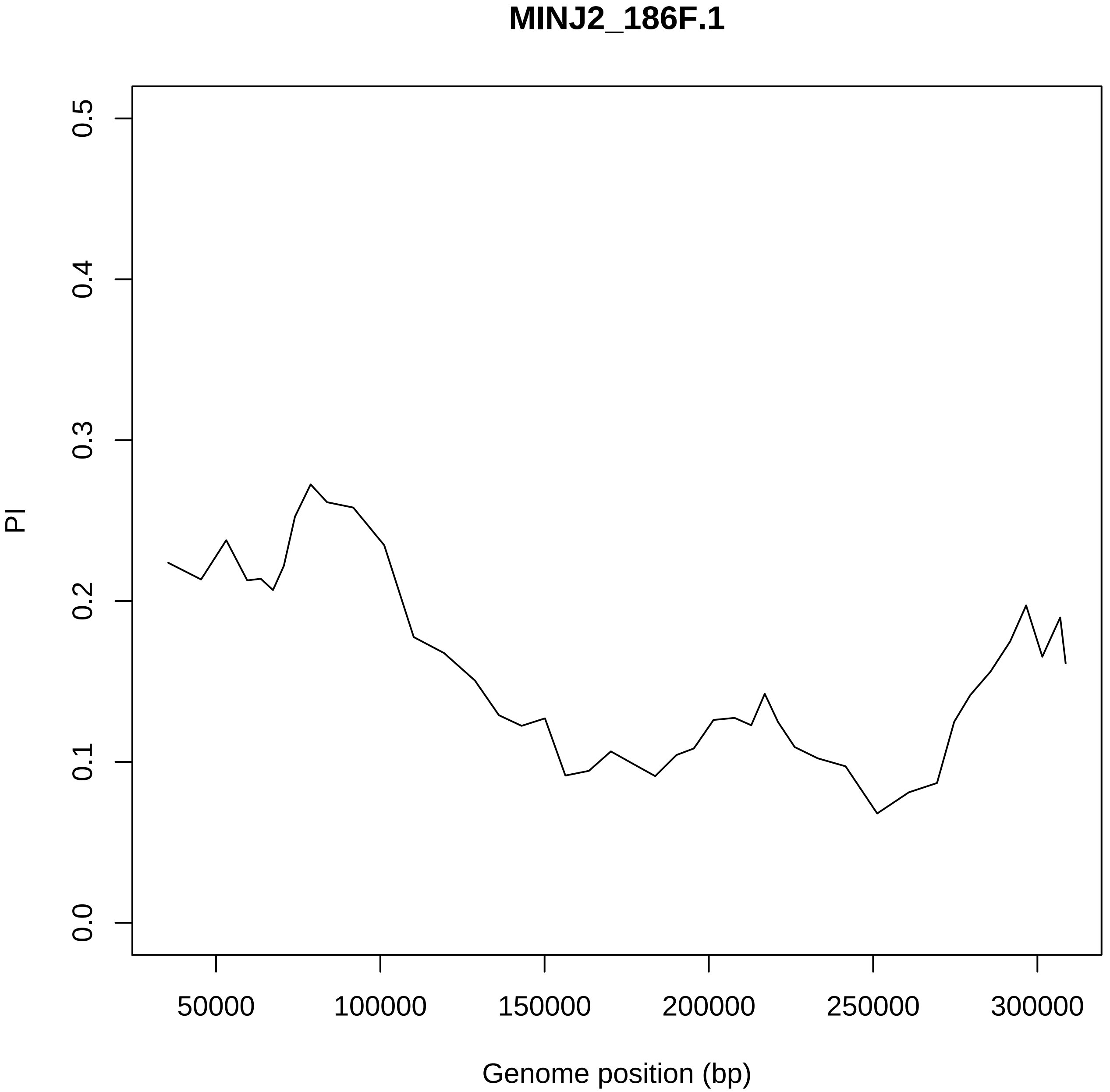
MINJ2_186F.1
PI
0.0
0.1
0.2
0.3
0.4
0.5
50000
100000
150000
200000
250000
300000
Genome position (bp)

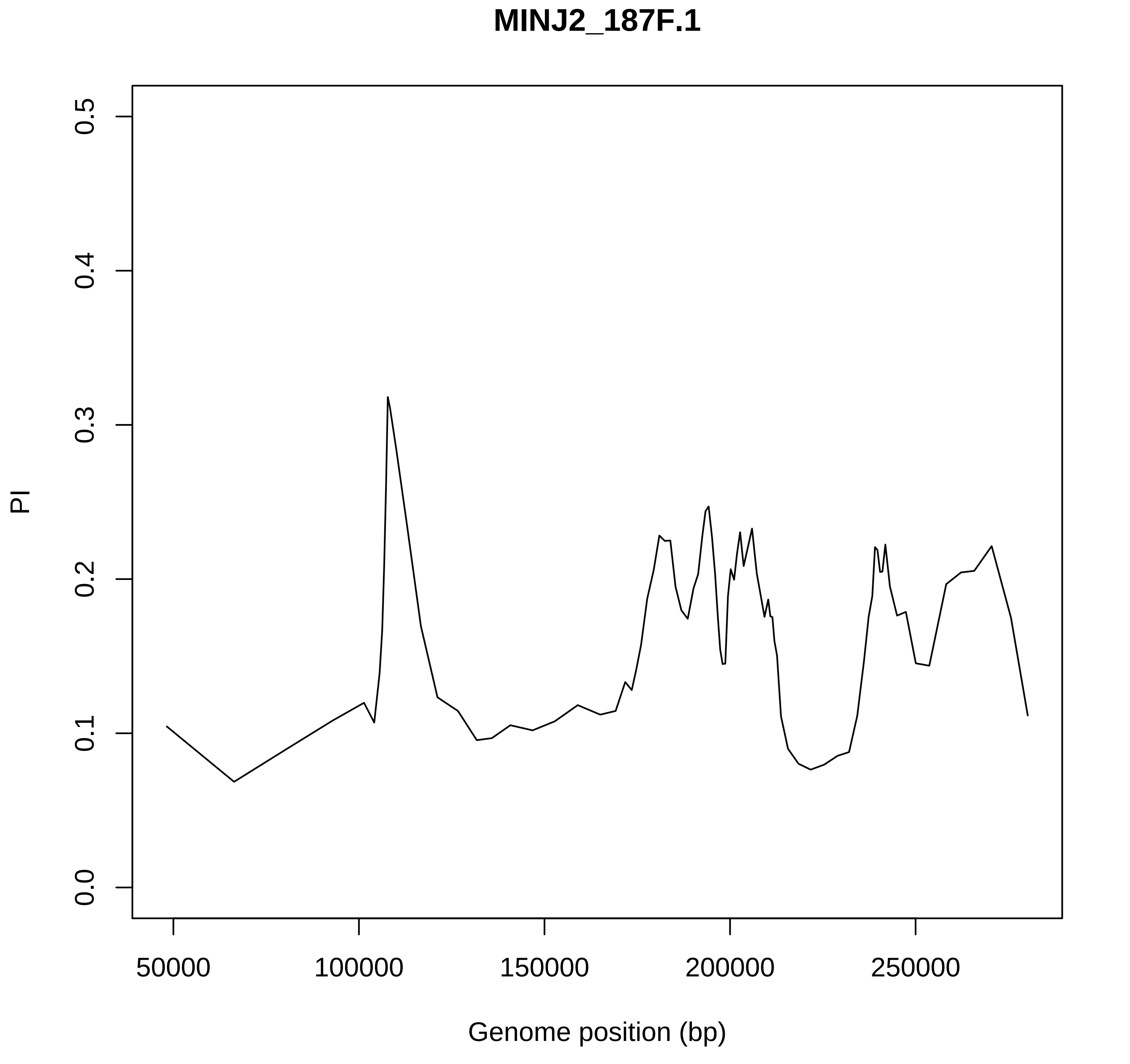
MINJ2_187F.1
PI
0.0
0.1
0.2
0.3
0.4
0.5
50000
100000
150000
200000
250000
Genome position (bp)

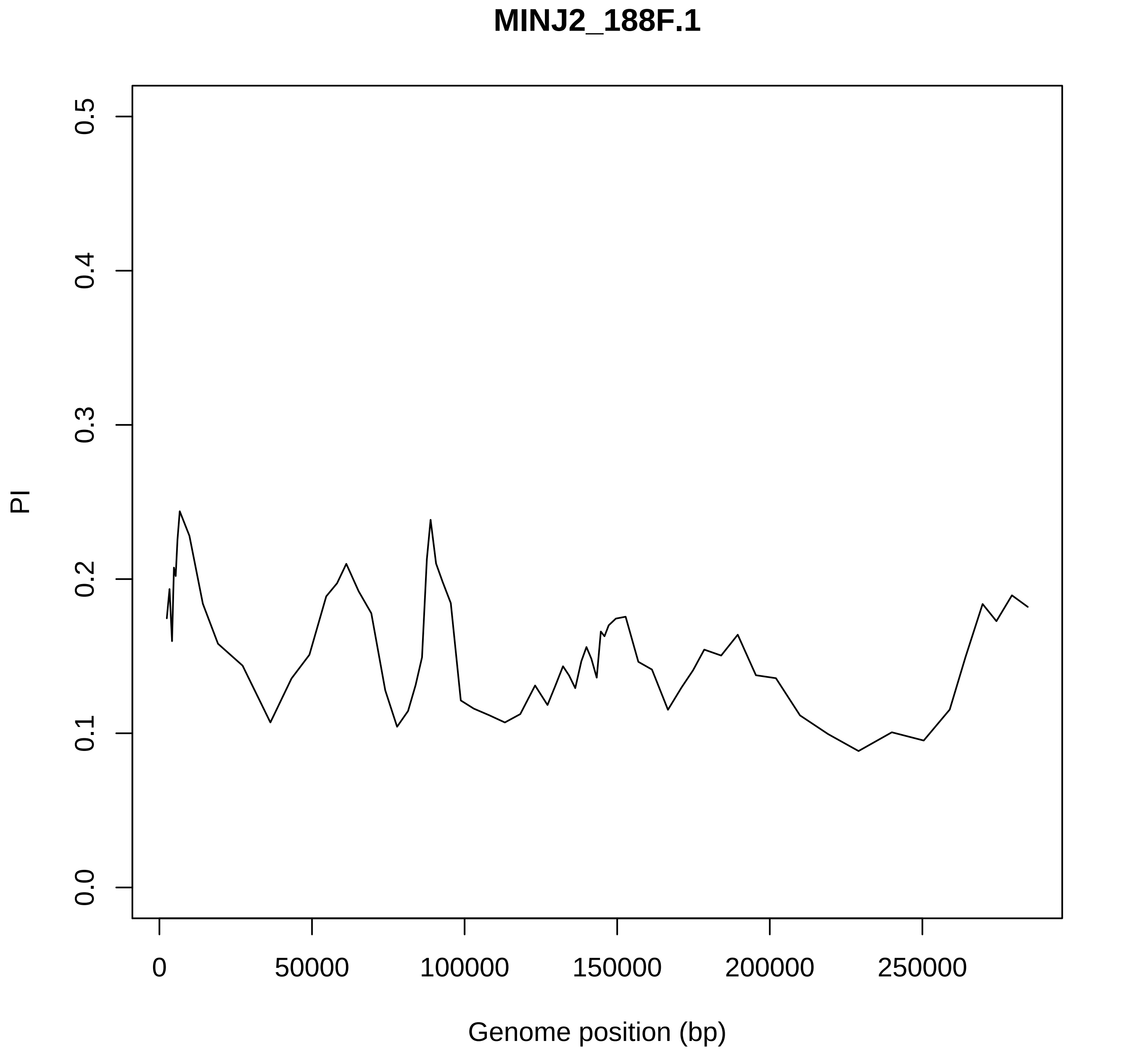

MINJ2_188F.1
PI
0.0
0.1
0.2
0.3
0.4
0.5
0
50000
100000
150000
200000
250000
Genome position (bp)

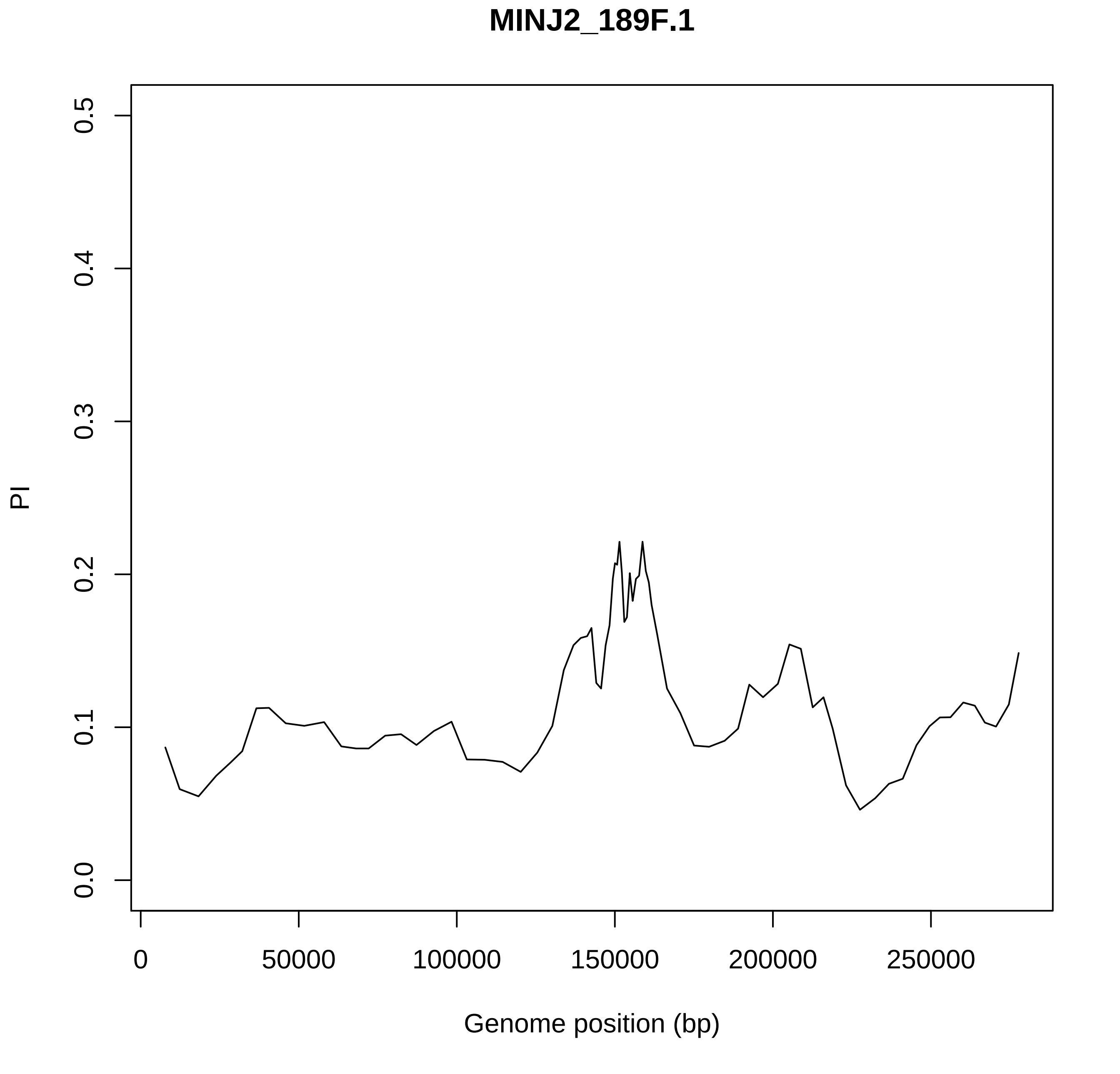
MINJ2_189F.1
PI
0.0
0.1
0.2
0.3
0.4
0.5
0
50000
100000
150000
200000
250000
Genome position (bp)

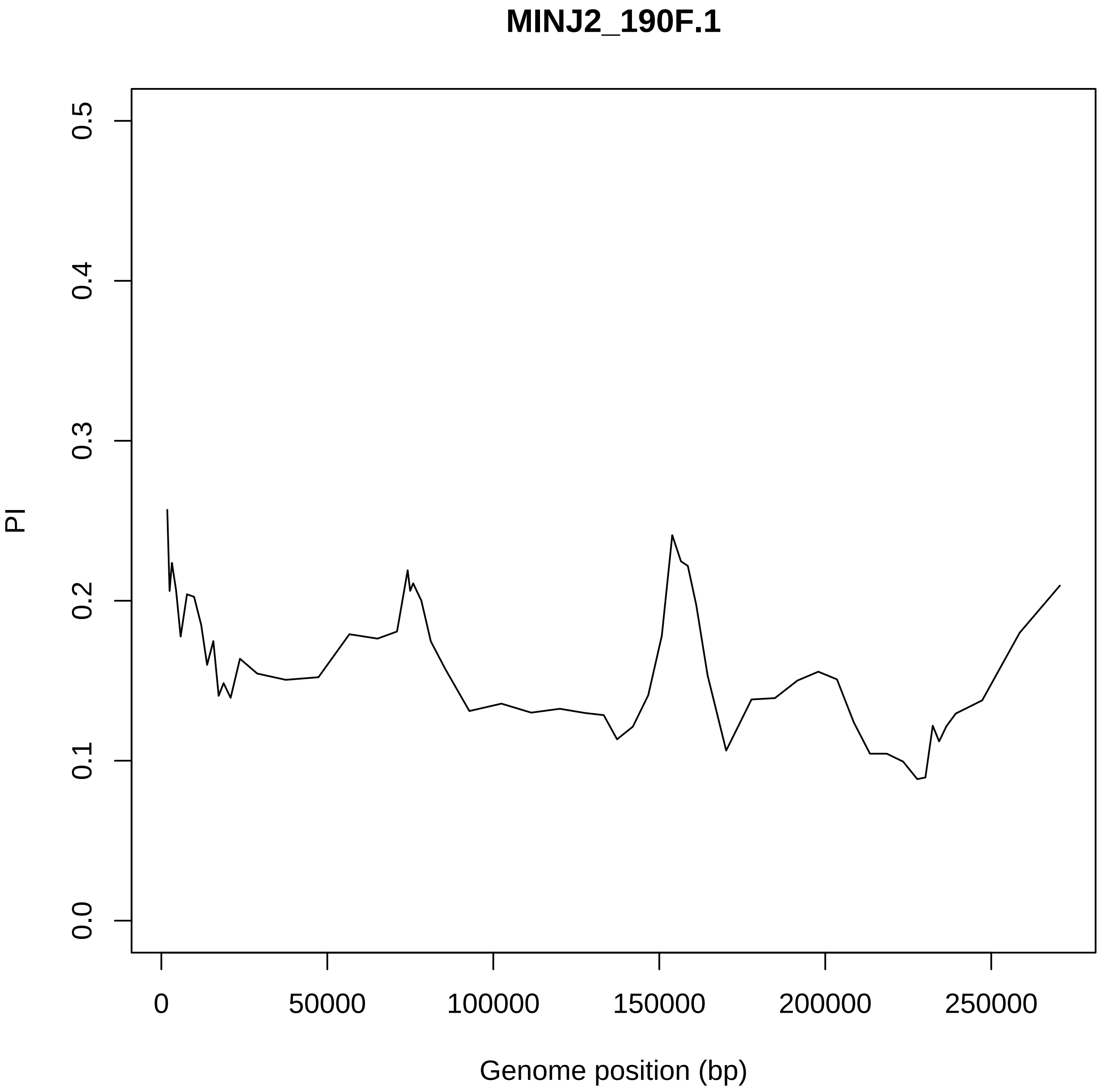

MINJ2_190F.1
0.0
0.1
0.2
0.3
0.4
0.5
PI
0
50000
100000
150000
200000
250000
Genome position (bp)

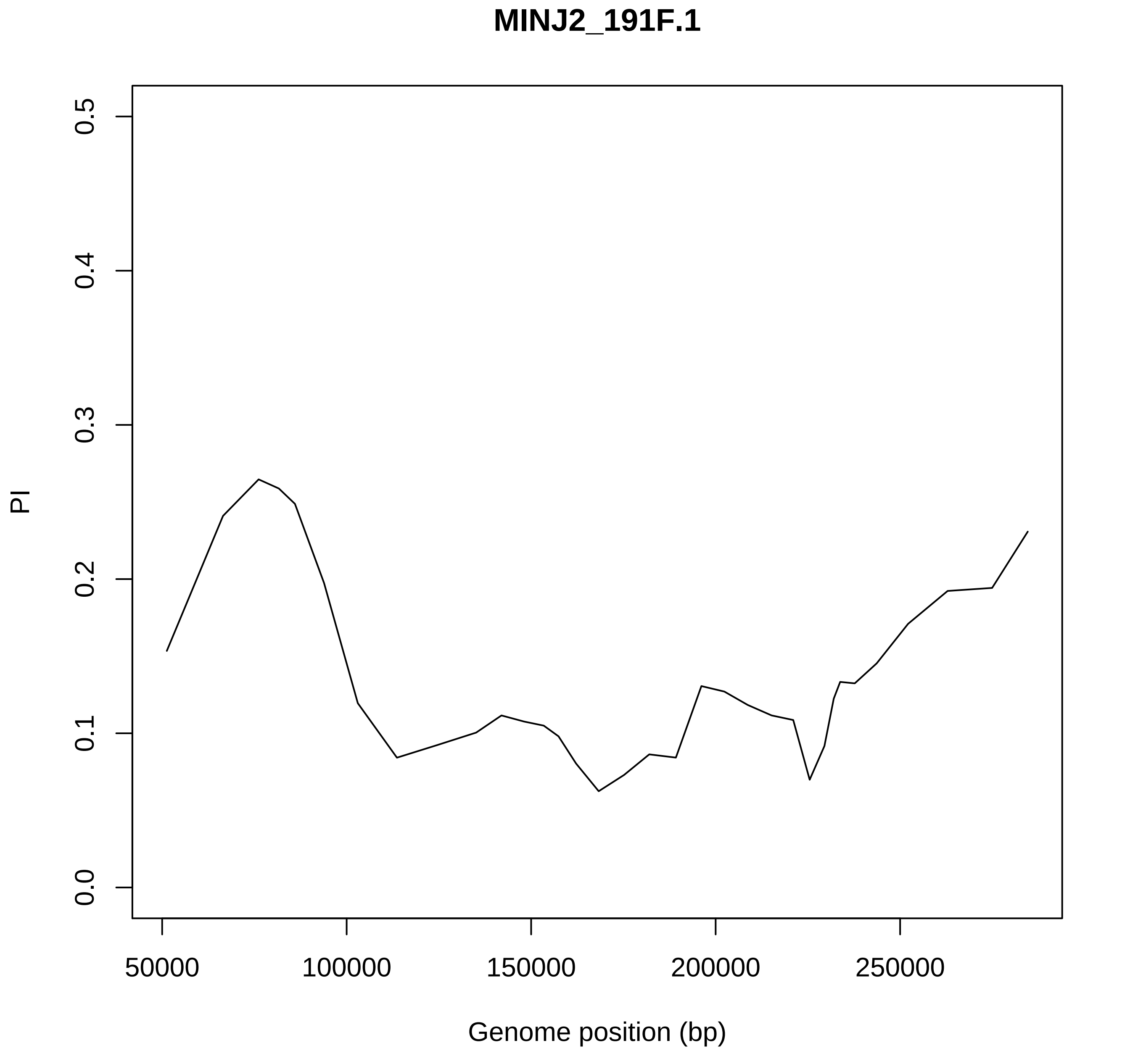

MINJ2_191F.1
PI
0.0
0.1
0.2
0.3
0.4
0.5
50000
100000
150000
200000
250000
Genome position (bp)

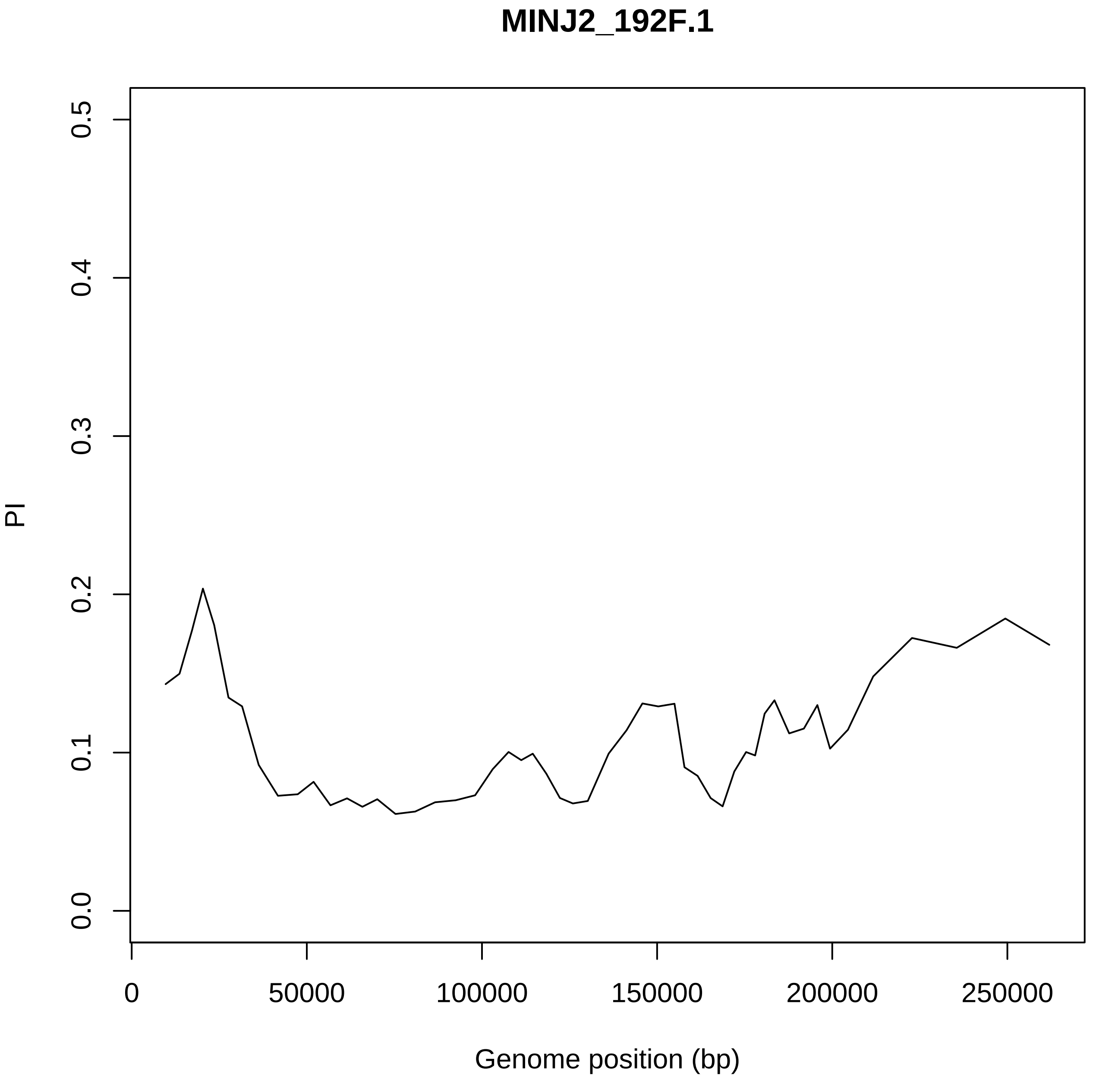
MINJ2_192F.1
0.0
0.1
0.2
0.3
0.4
0.5
PI
0
50000
100000
150000
200000
250000
Genome position (bp)

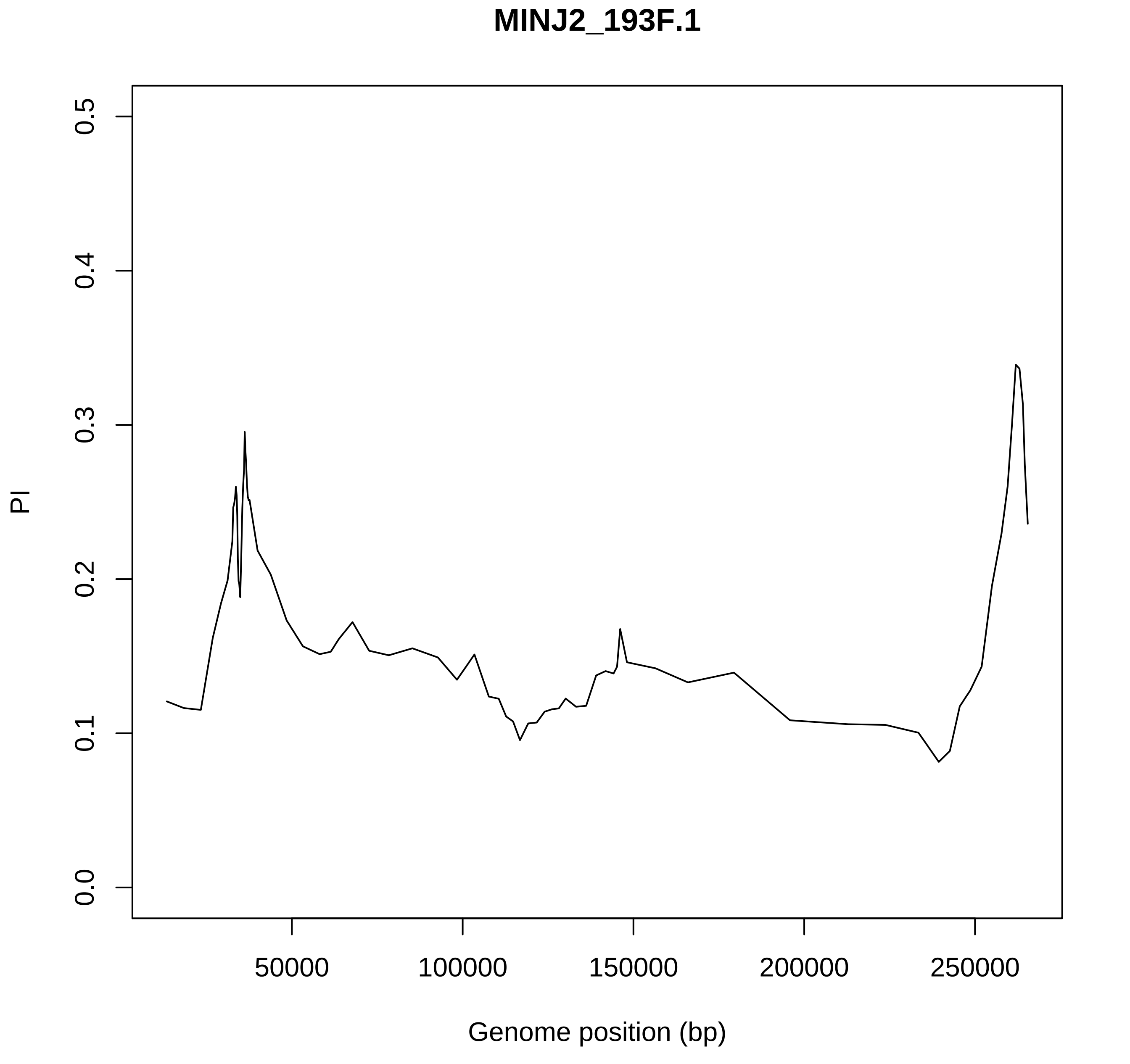
MINJ2_193F.1
PI
0.0
0.1
0.2
0.3
0.4
0.5
50000
100000
150000
200000
250000
Genome position (bp)

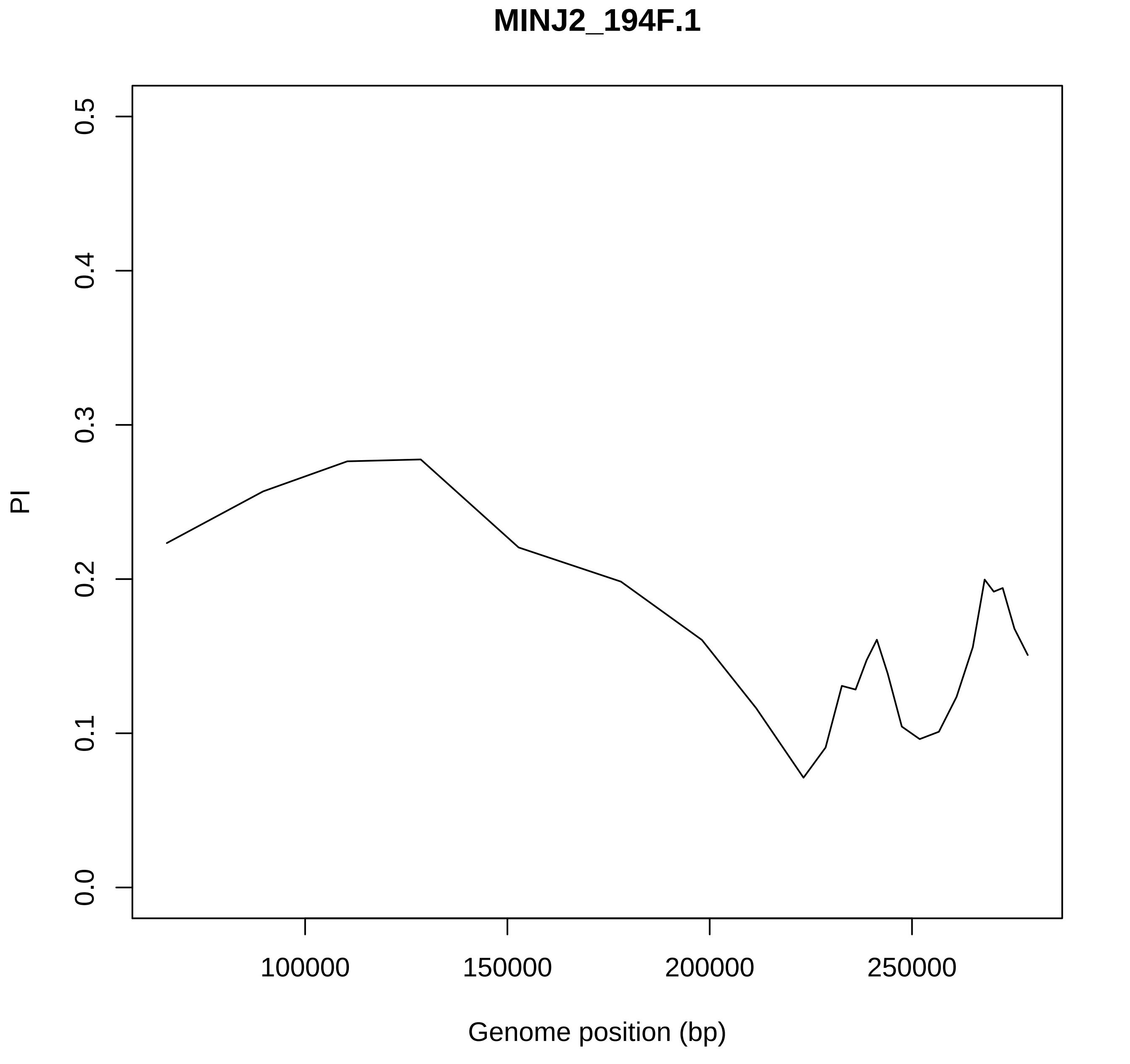
MINJ2_194F.1
PI
0.0
0.1
0.2
0.3
0.4
0.5
100000
150000
200000
250000
Genome position (bp)

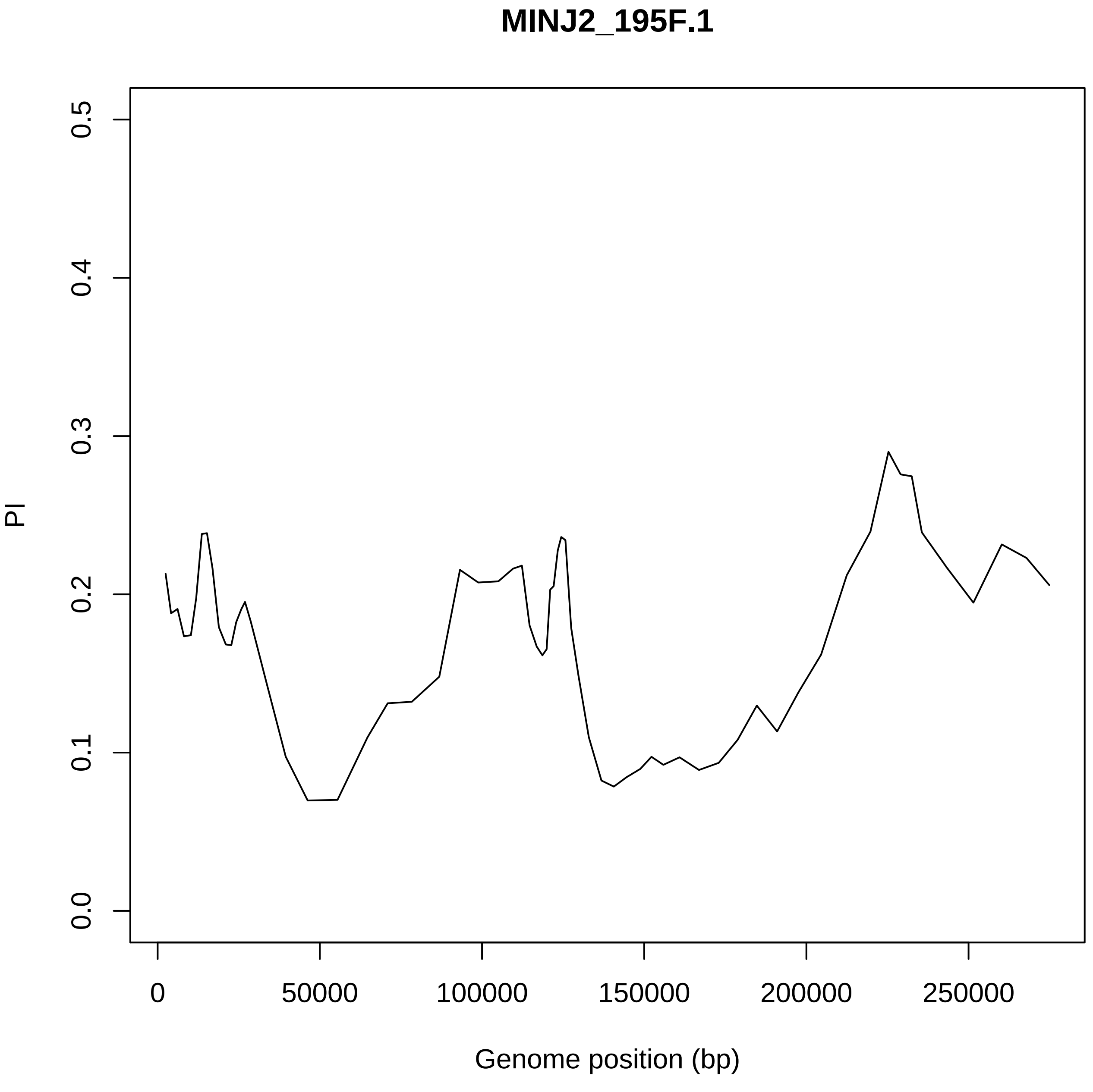
MINJ2_195F.1
PI
0.0
0.1
0.2
0.3
0.4
0.5
0
50000
100000
150000
200000
250000
Genome position (bp)

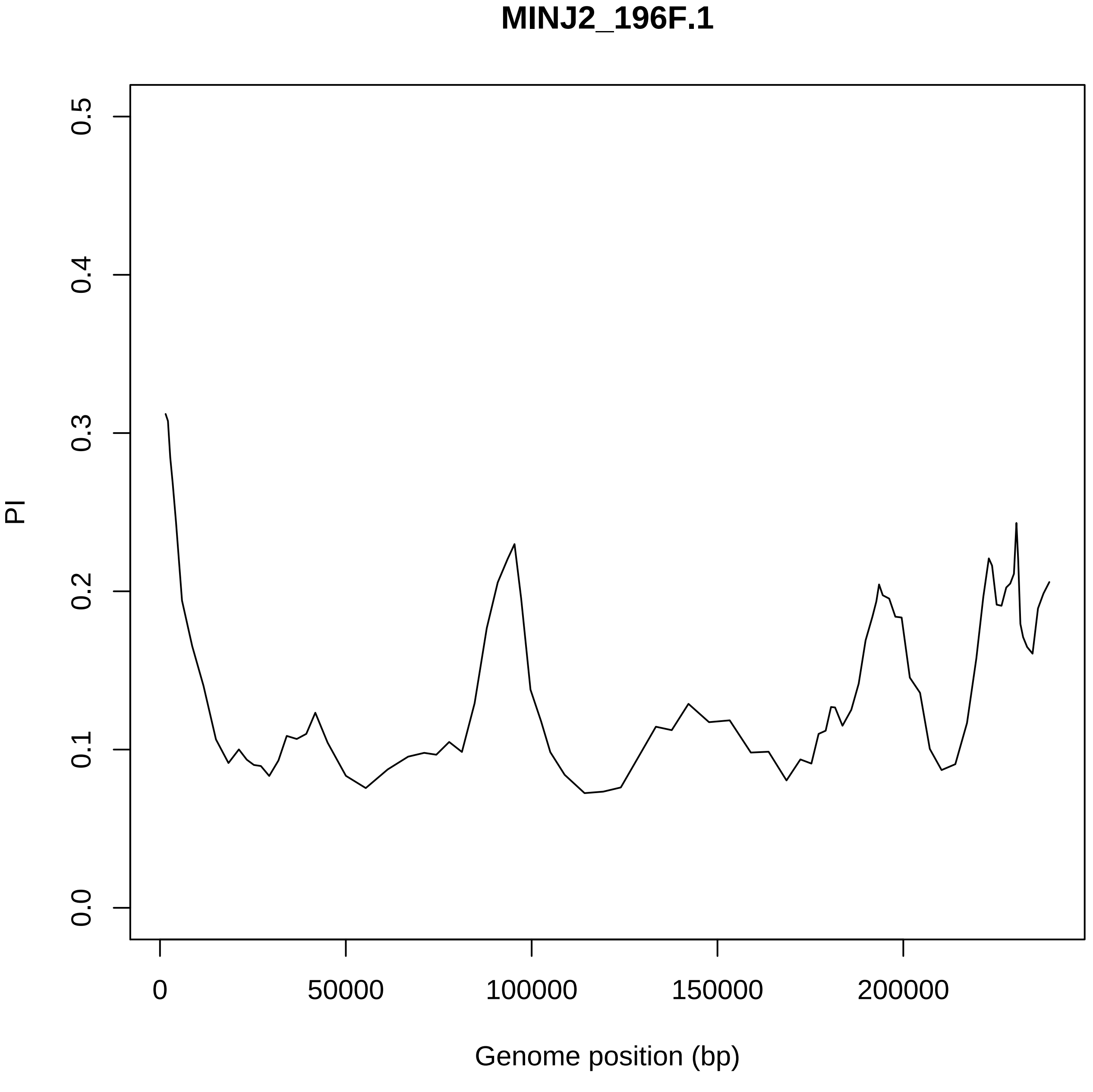
MINJ2_196F.1
PI
0.0
0.1
0.2
0.3
0.4
0.5
0
50000
100000
150000
200000
Genome position (bp)

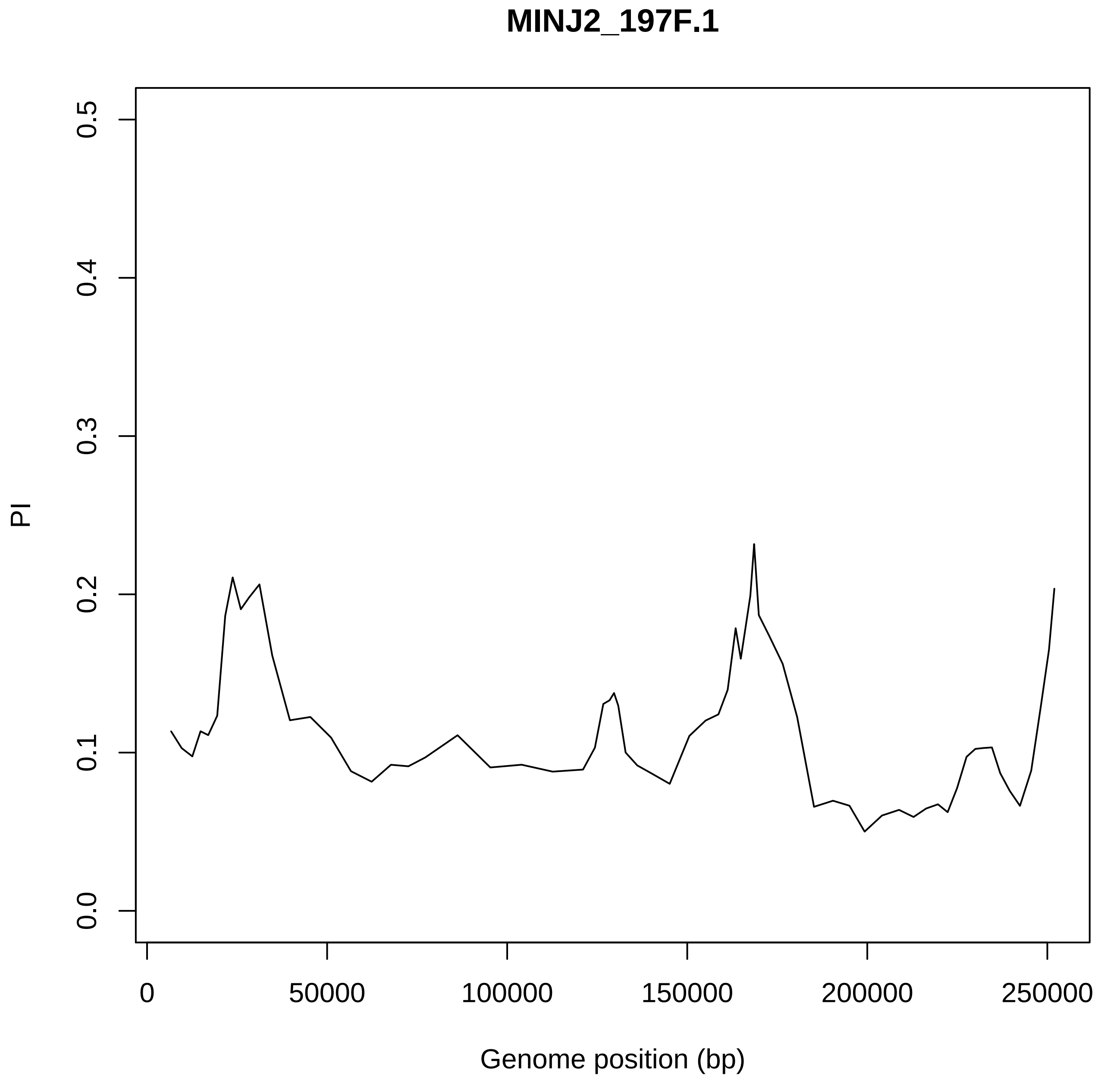
MINJ2_197F.1
PI
0.0
0.1
0.2
0.3
0.4
0.5
0
50000
100000
150000
200000
250000
Genome position (bp)

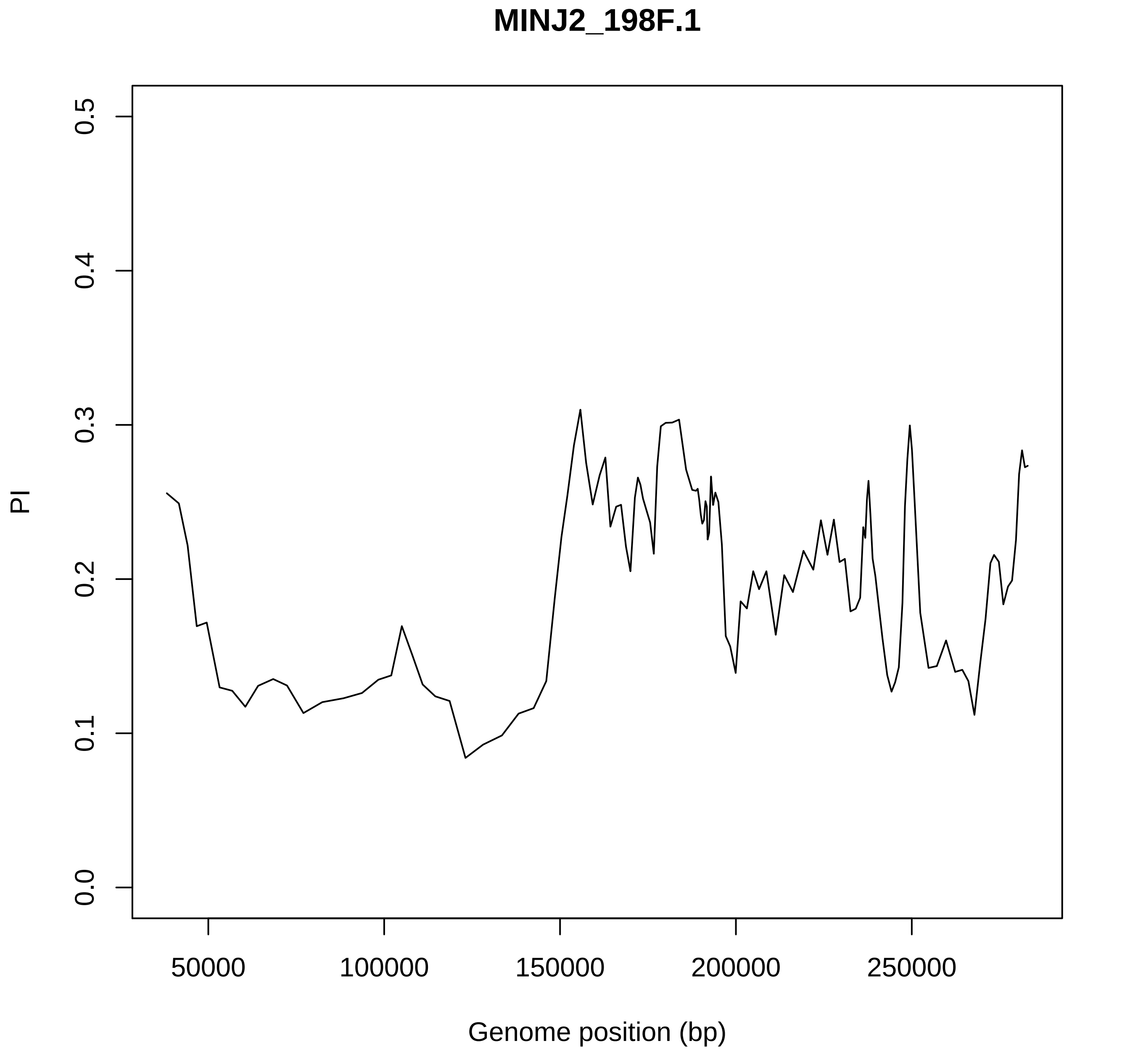
MINJ2_198F.1
PI
0.0
0.1
0.2
0.3
0.4
0.5
50000
100000
150000
200000
250000
Genome position (bp)

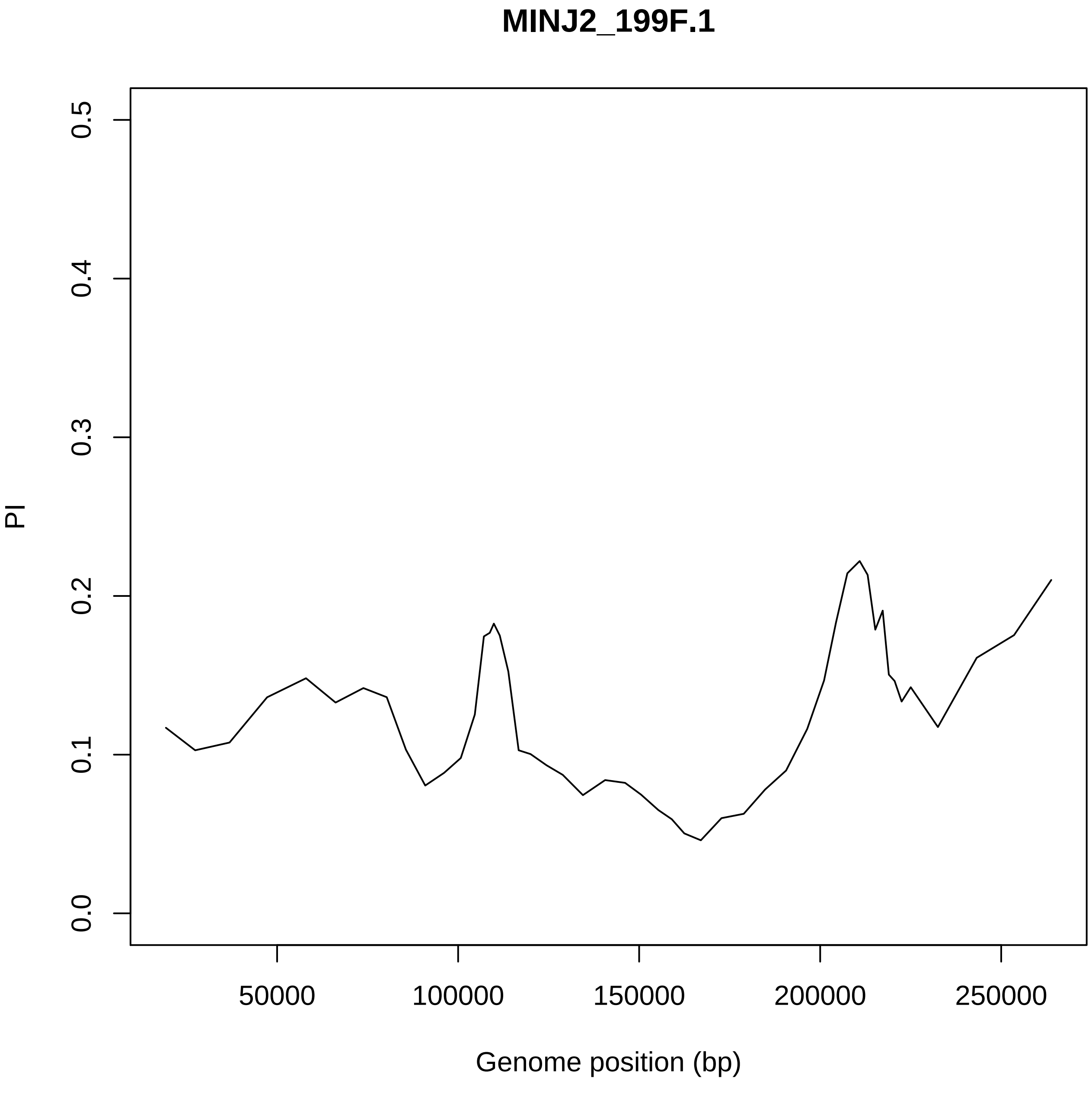
MINJ2_199F.1
PI
0.0
0.1
0.2
0.3
0.4
0.5
50000
100000
150000
200000
250000
Genome position (bp)

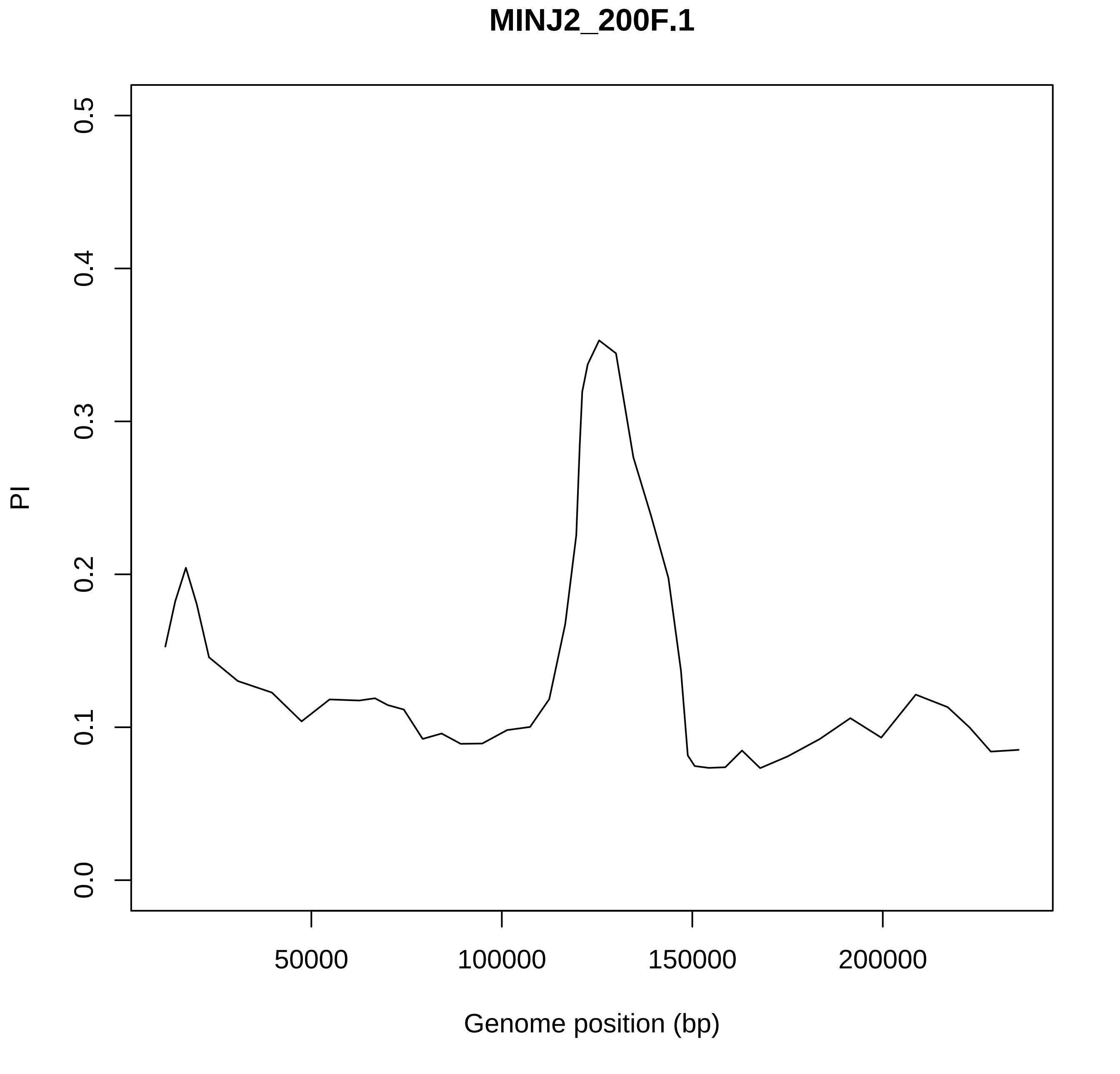
MINJ2_200F.1
PI
0.0
0.1
0.2
0.3
0.4
0.5
50000
100000
150000
200000
Genome position (bp)

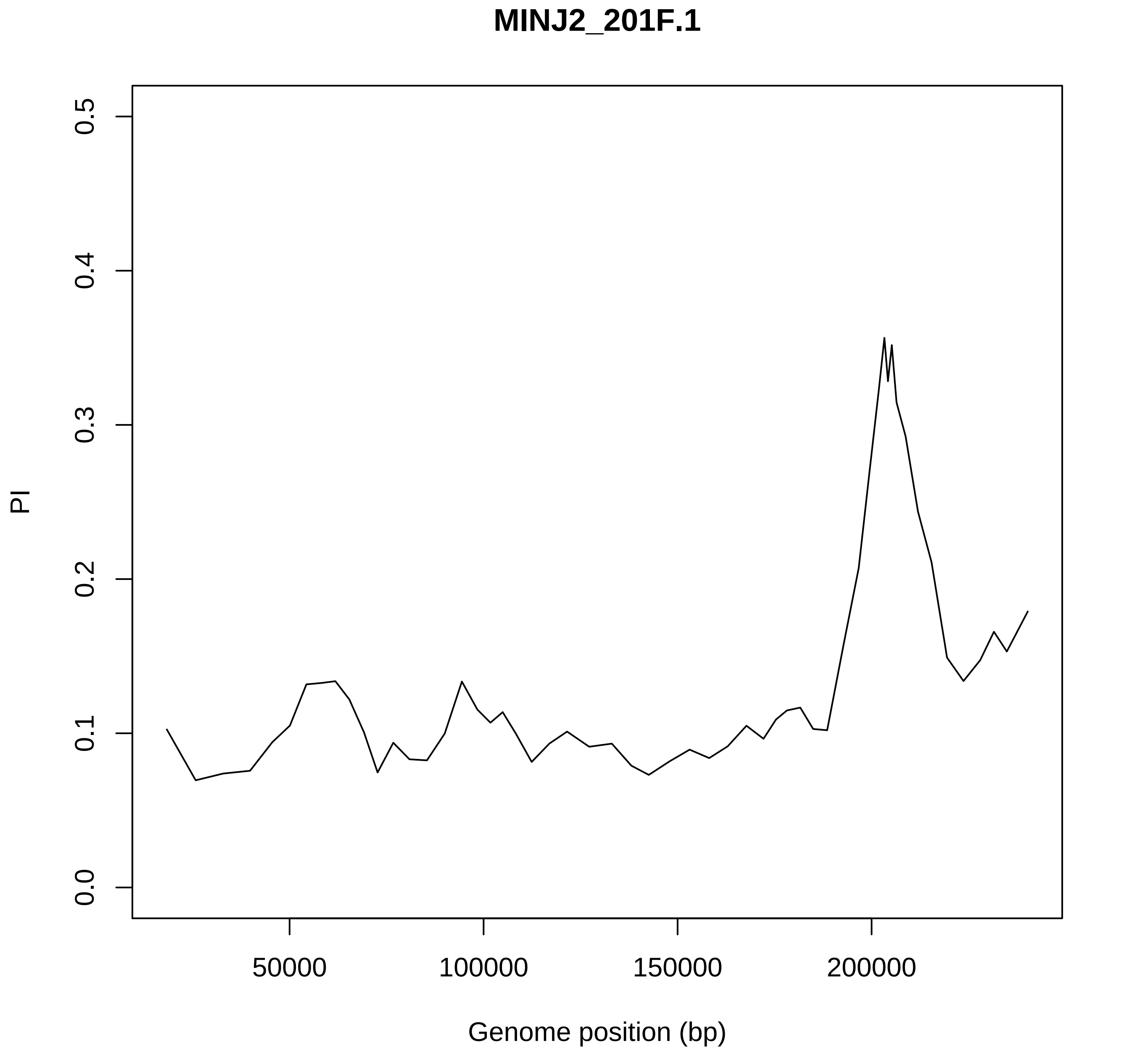
MINJ2_201F.1
PI
0.0
0.1
0.2
0.3
0.4
0.5
50000
100000
150000
200000
Genome position (bp)

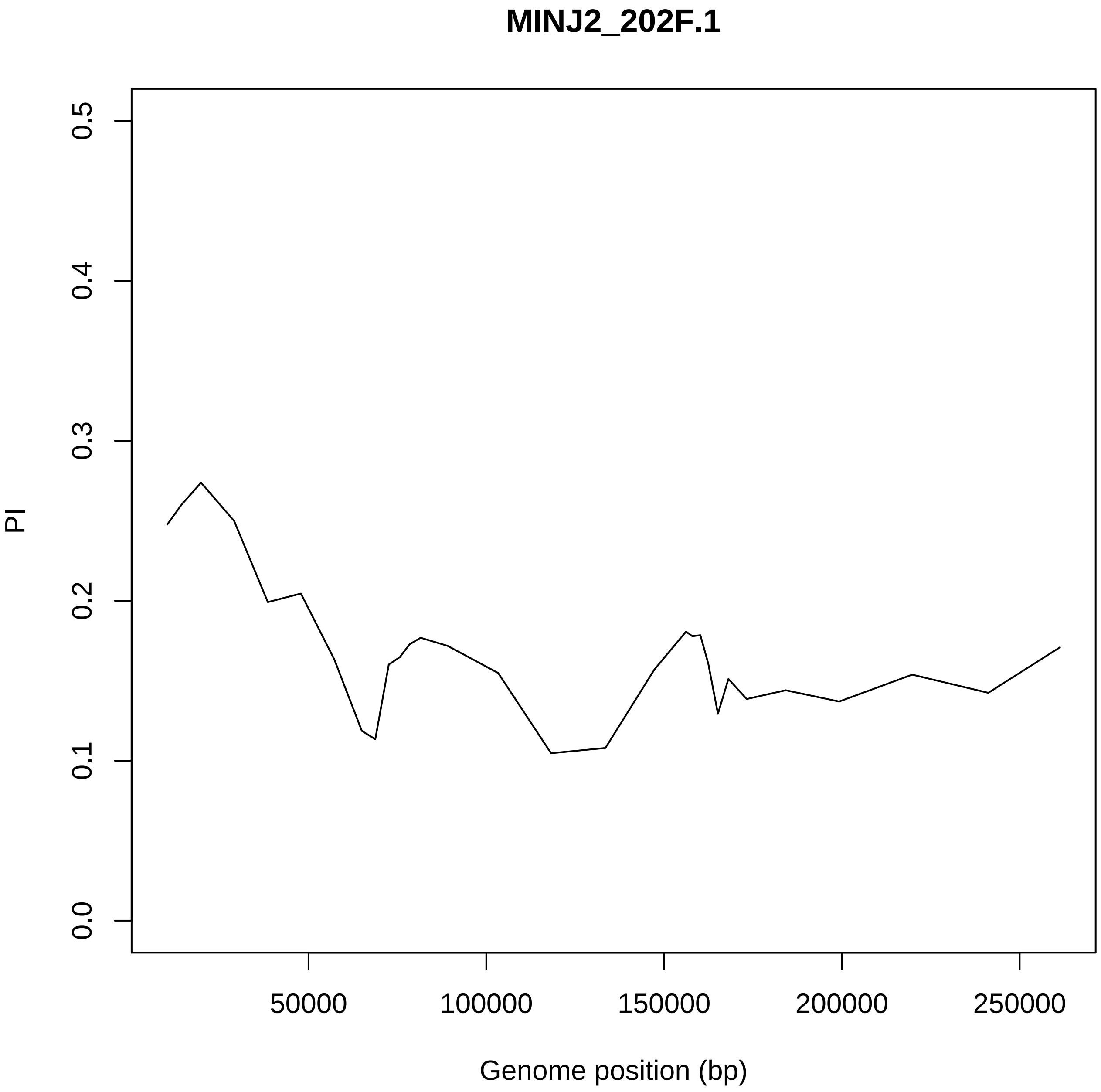
MINJ2_202F.1
PI
0.0
0.1
0.2
0.3
0.4
0.5
50000
100000
150000
200000
250000
Genome position (bp)

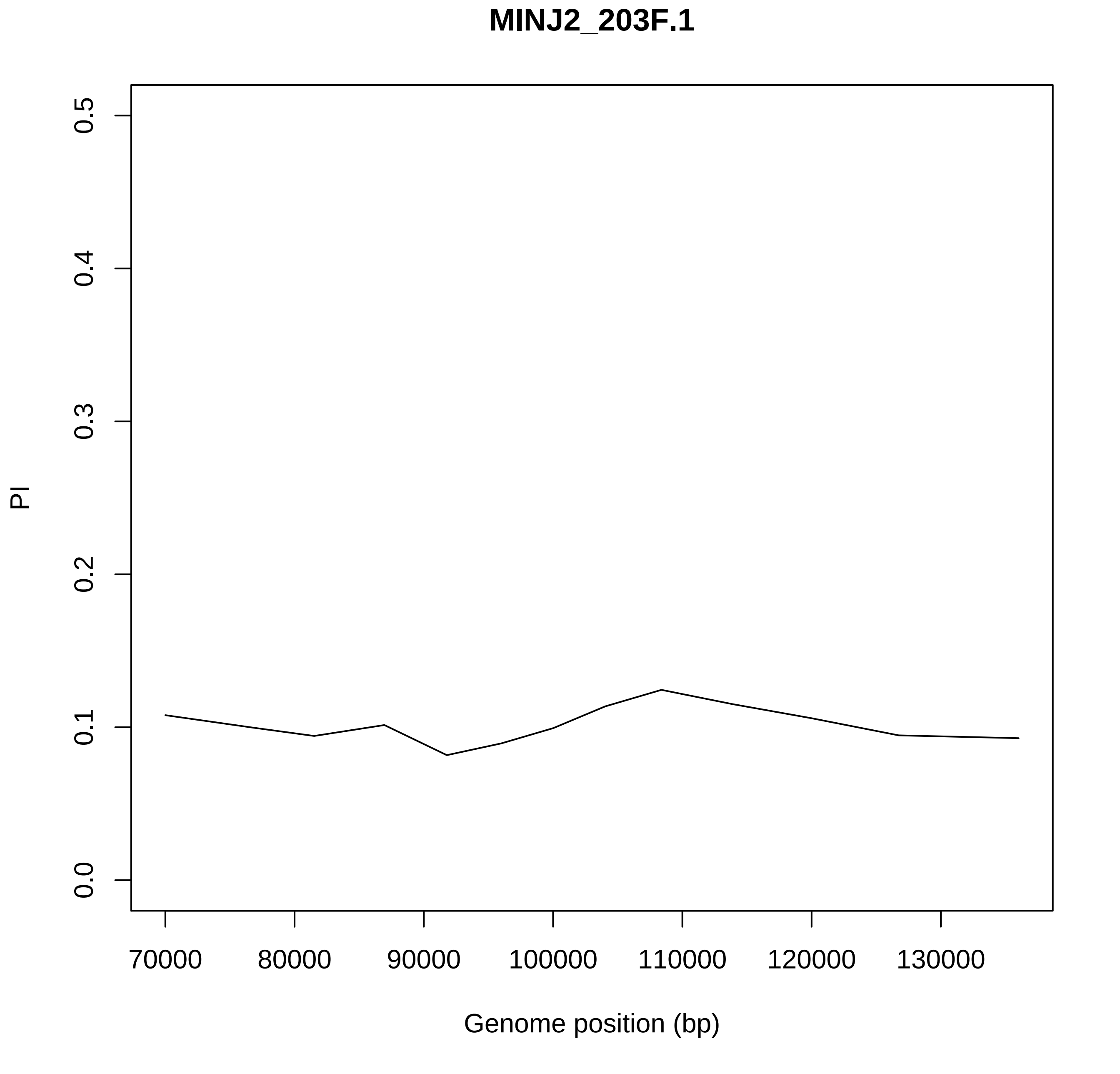
MINJ2_203F.1
PI
0.0
0.1
0.2
0.3
0.4
0.5
70000
80000
90000
100000
110000
120000
130000
Genome position (bp)

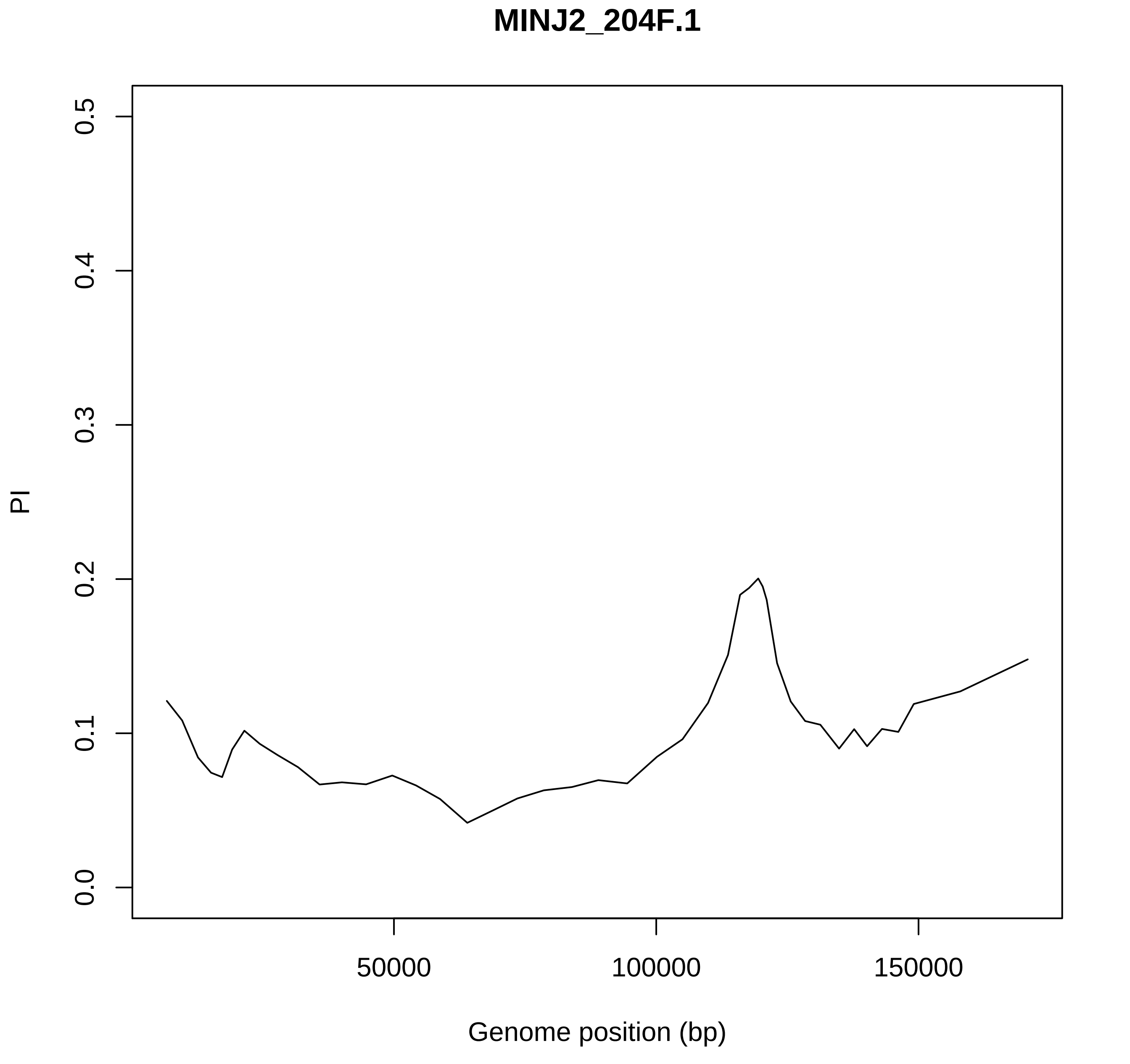
MINJ2_204F.1
PI
0.0
0.1
0.2
0.3
0.4
0.5
50000
100000
150000
Genome position (bp)

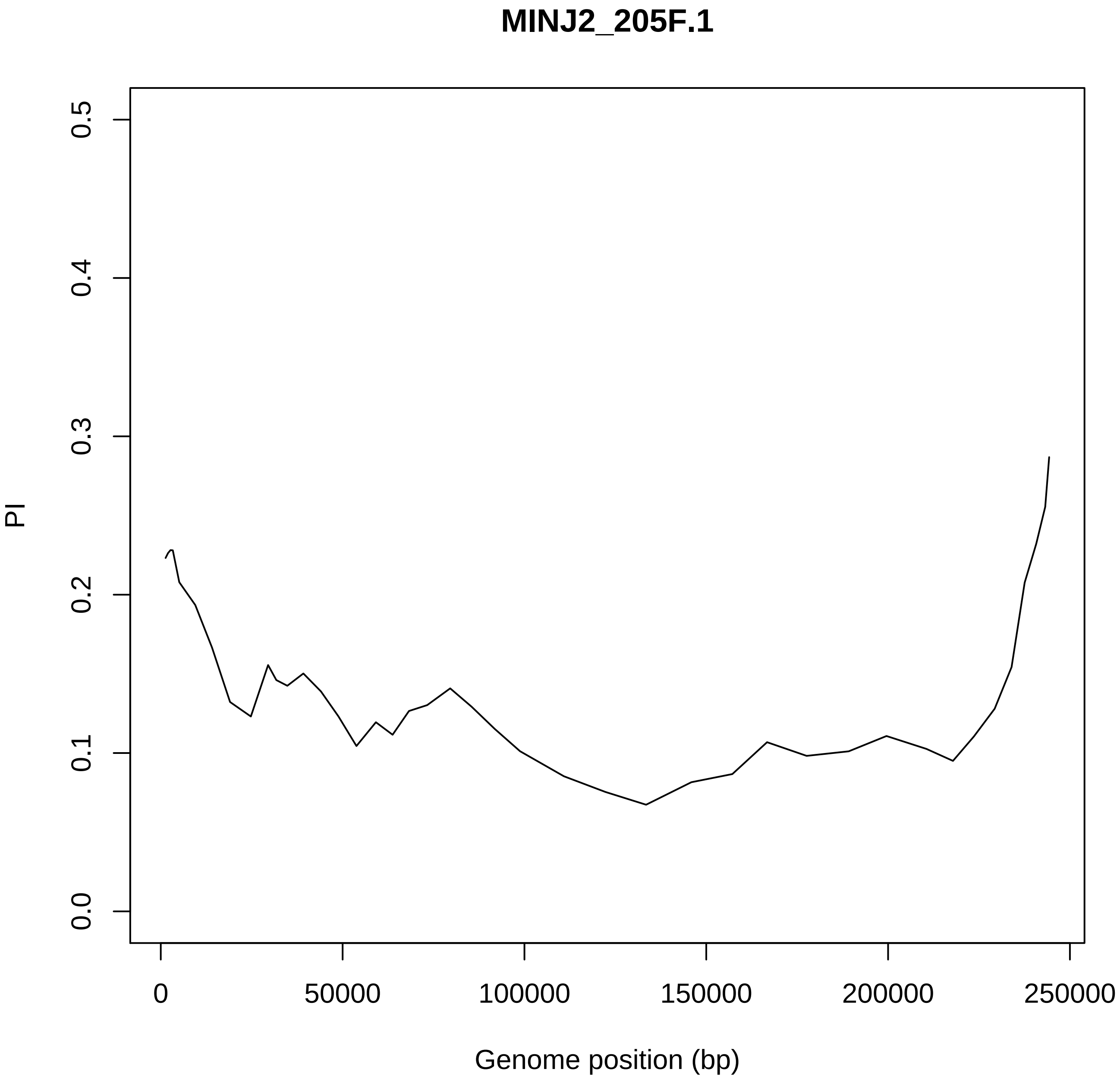
MINJ2_205F.1
PI
0.0
0.1
0.2
0.3
0.4
0.5
0
50000
100000
150000
200000
250000
Genome position (bp)

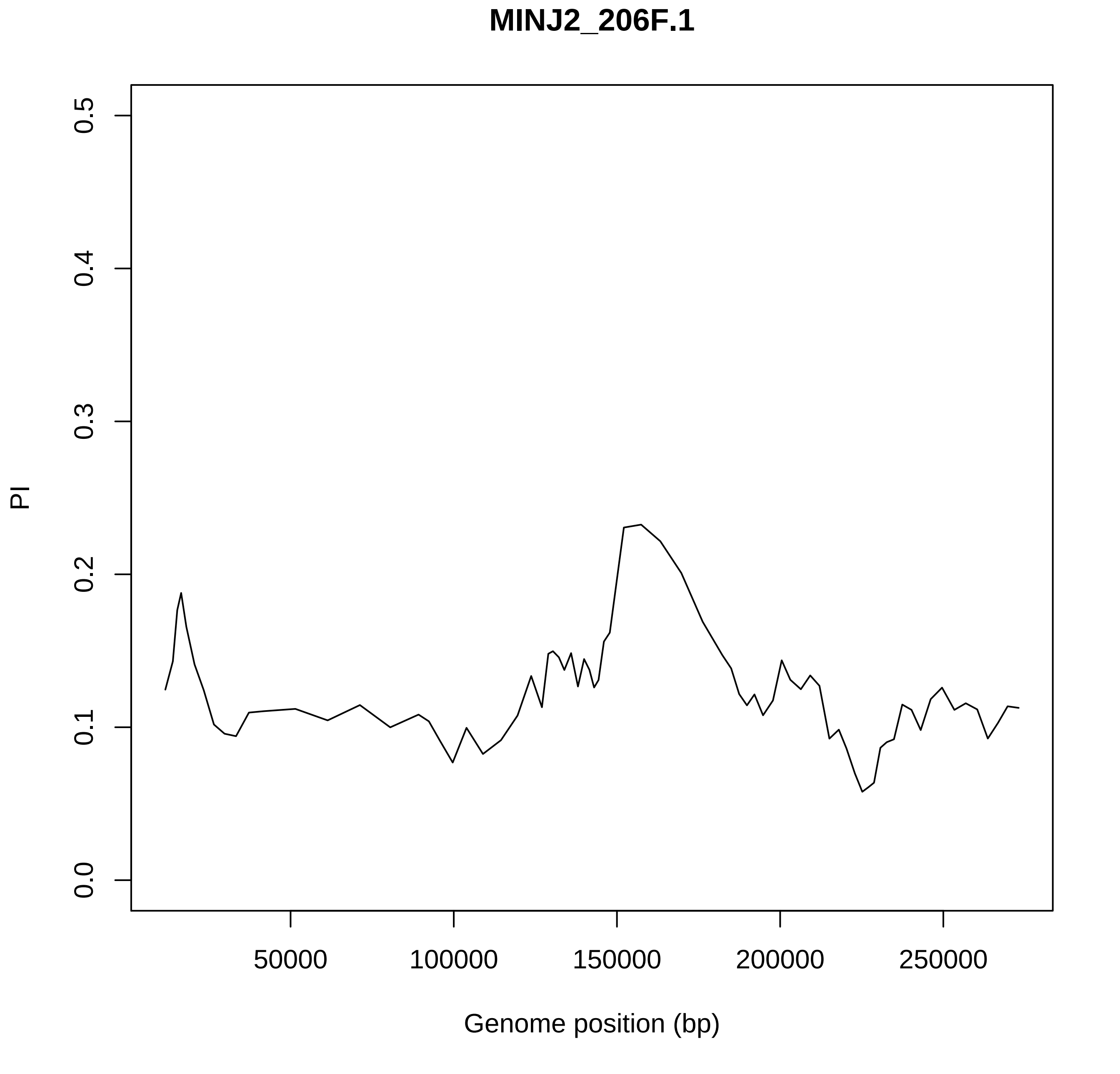
MINJ2_206F.1
PI
0.0
0.1
0.2
0.3
0.4
0.5
50000
100000
150000
200000
250000
Genome position (bp)

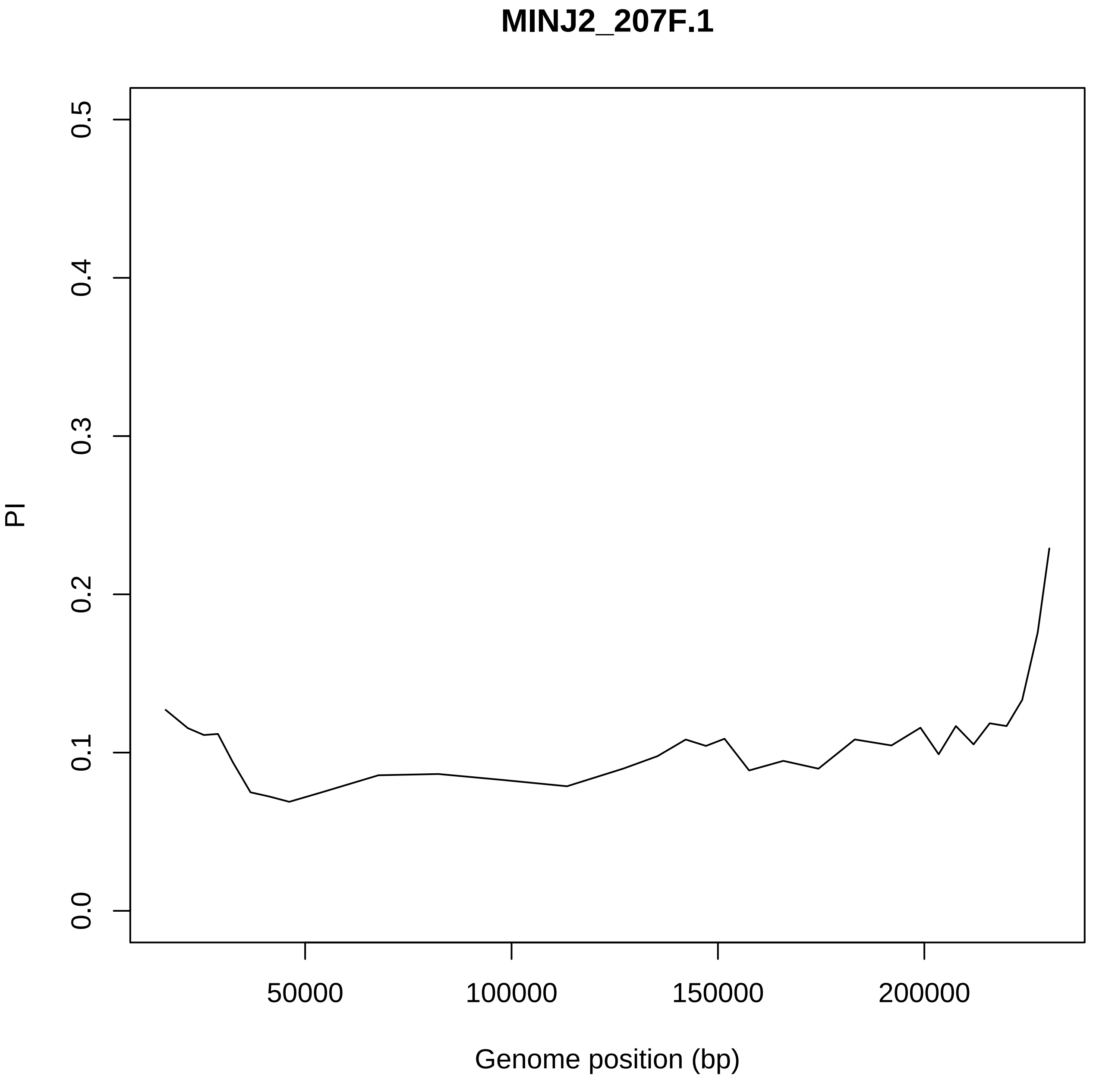
MINJ2_207F.1
PI
0.0
0.1
0.2
0.3
0.4
0.5
50000
100000
150000
200000
Genome position (bp)

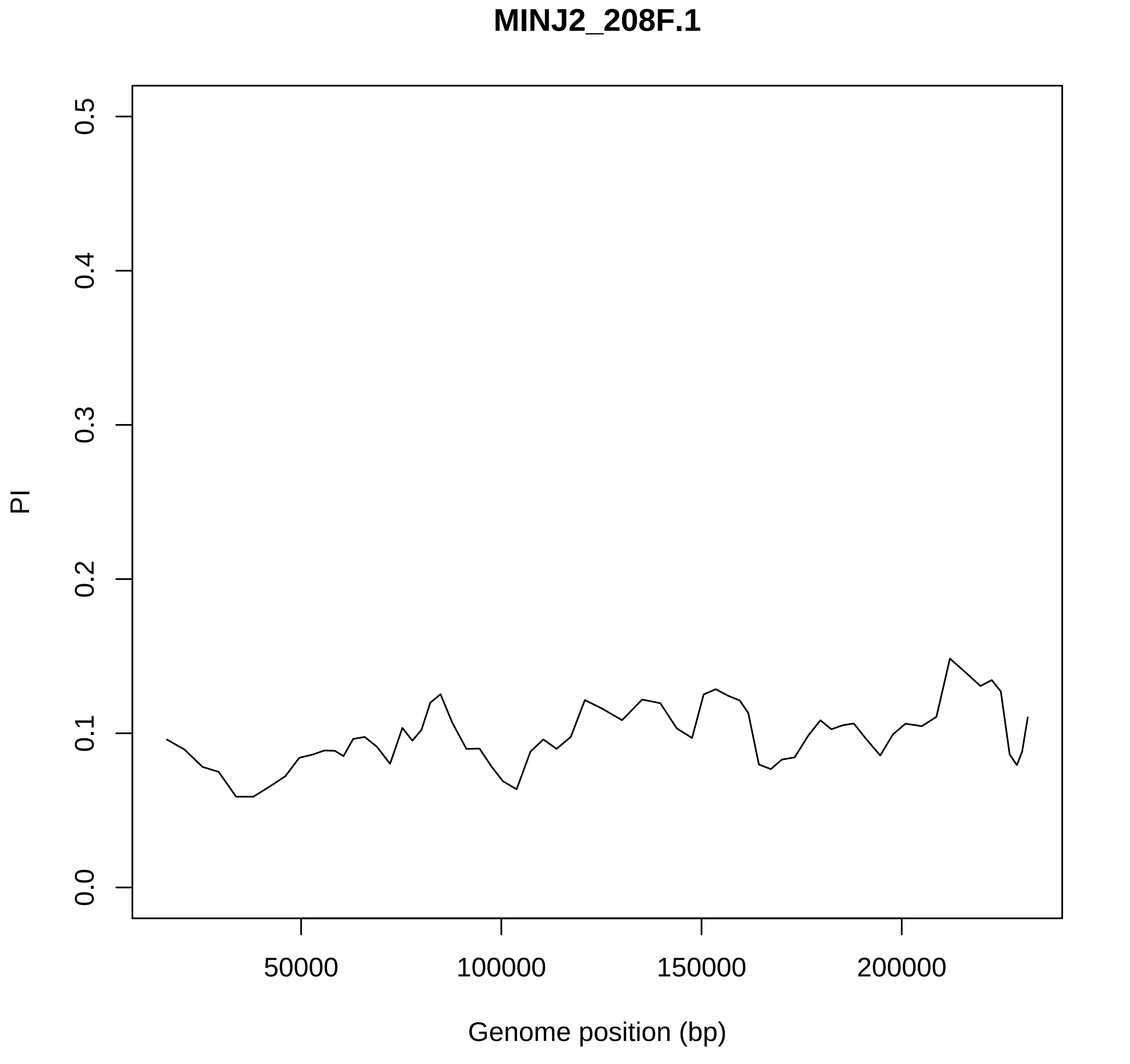
MINJ2_208F.1
PI
0.0
0.1
0.2
0.3
0.4
0.5
50000
100000
150000
200000
Genome position (bp)

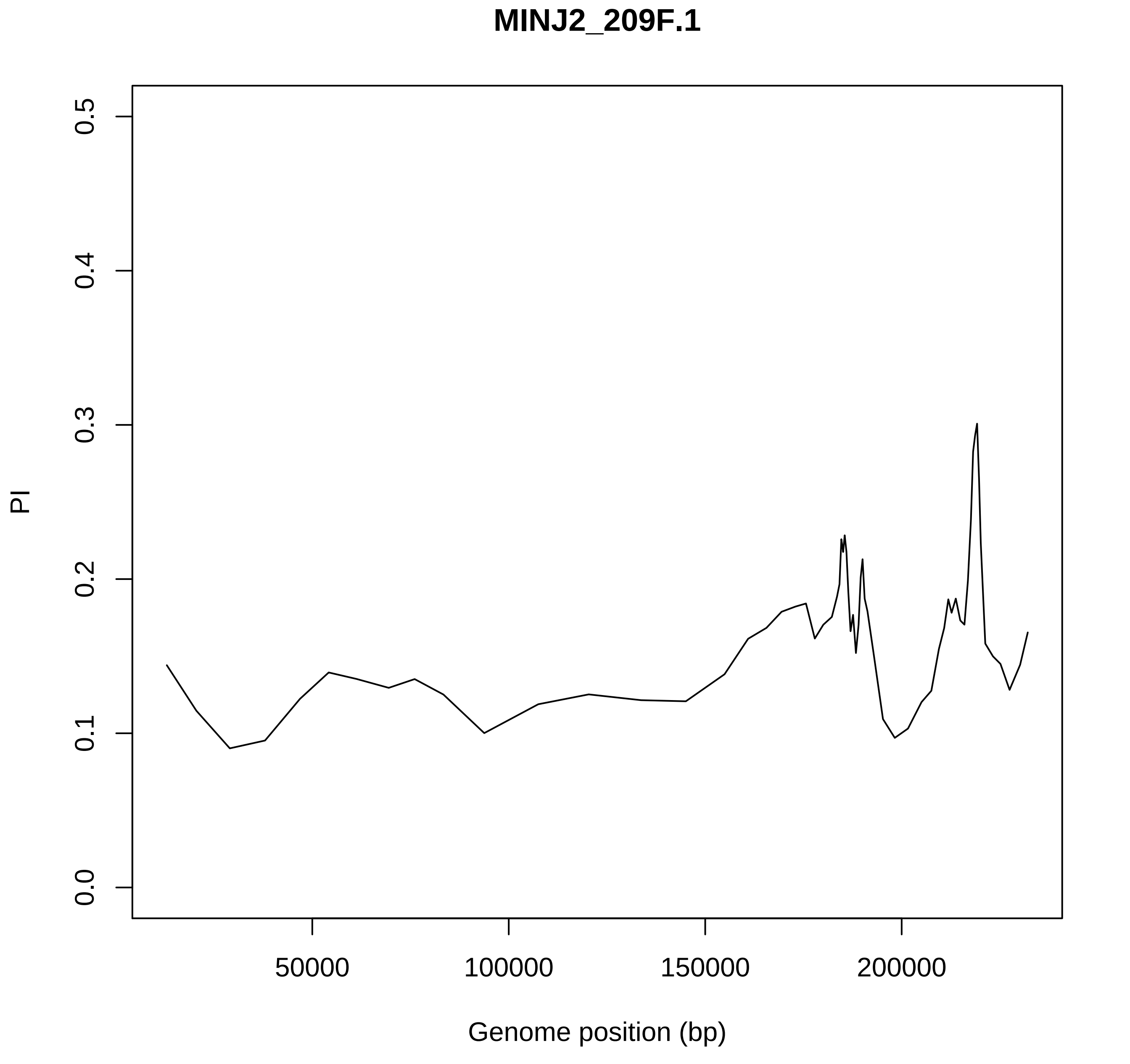
MINJ2_209F.1
PI
0.0
0.1
0.2
0.3
0.4
0.5
50000
100000
150000
200000
Genome position (bp)

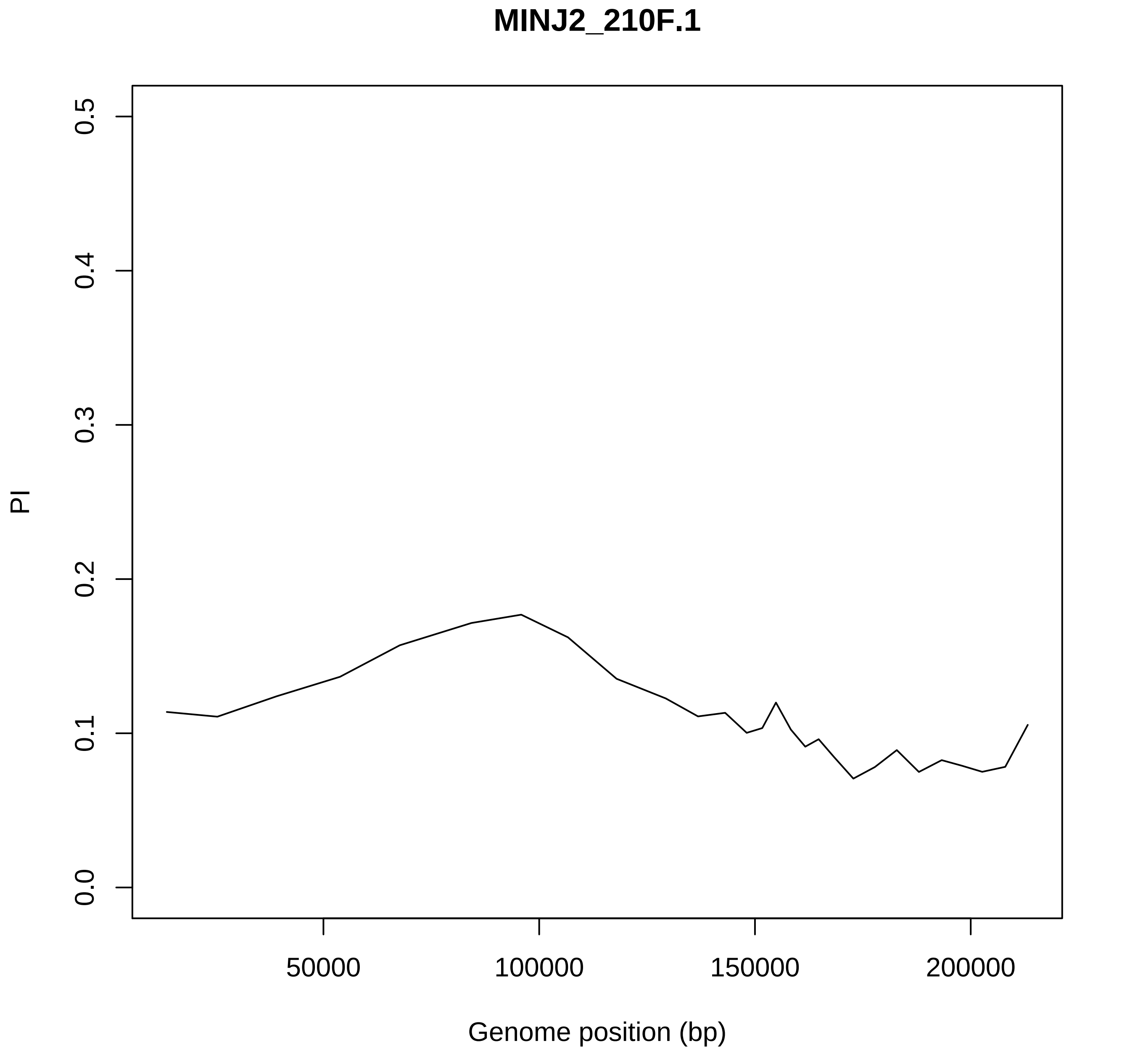
MINJ2_210F.1
PI
0.0
0.1
0.2
0.3
0.4
0.5
50000
100000
150000
200000
Genome position (bp)

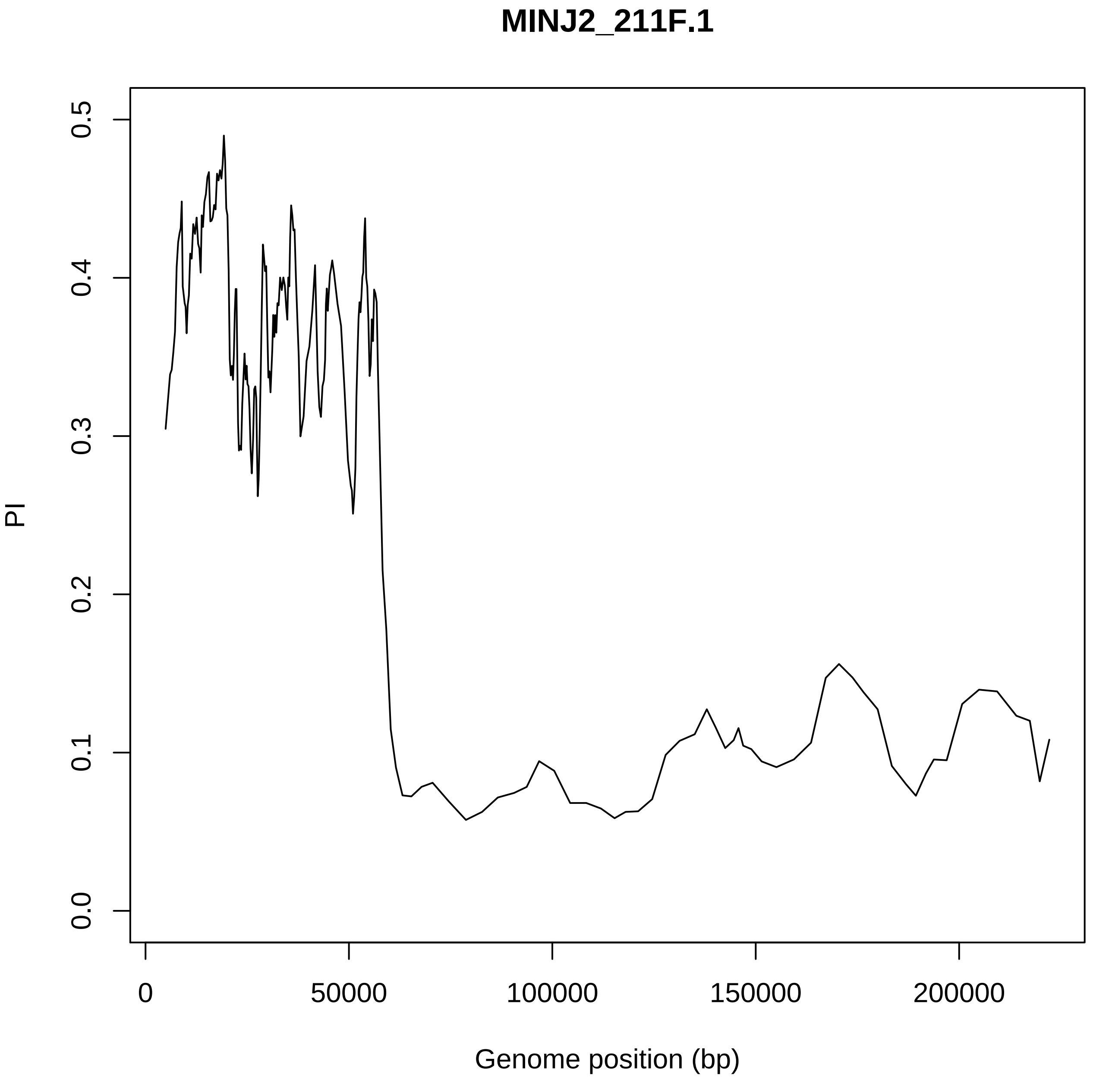

MINJ2_211F.1
PI
0.0
0.1
0.2
0.3
0.4
0.5
0
50000
100000
150000
200000
Genome position (bp)

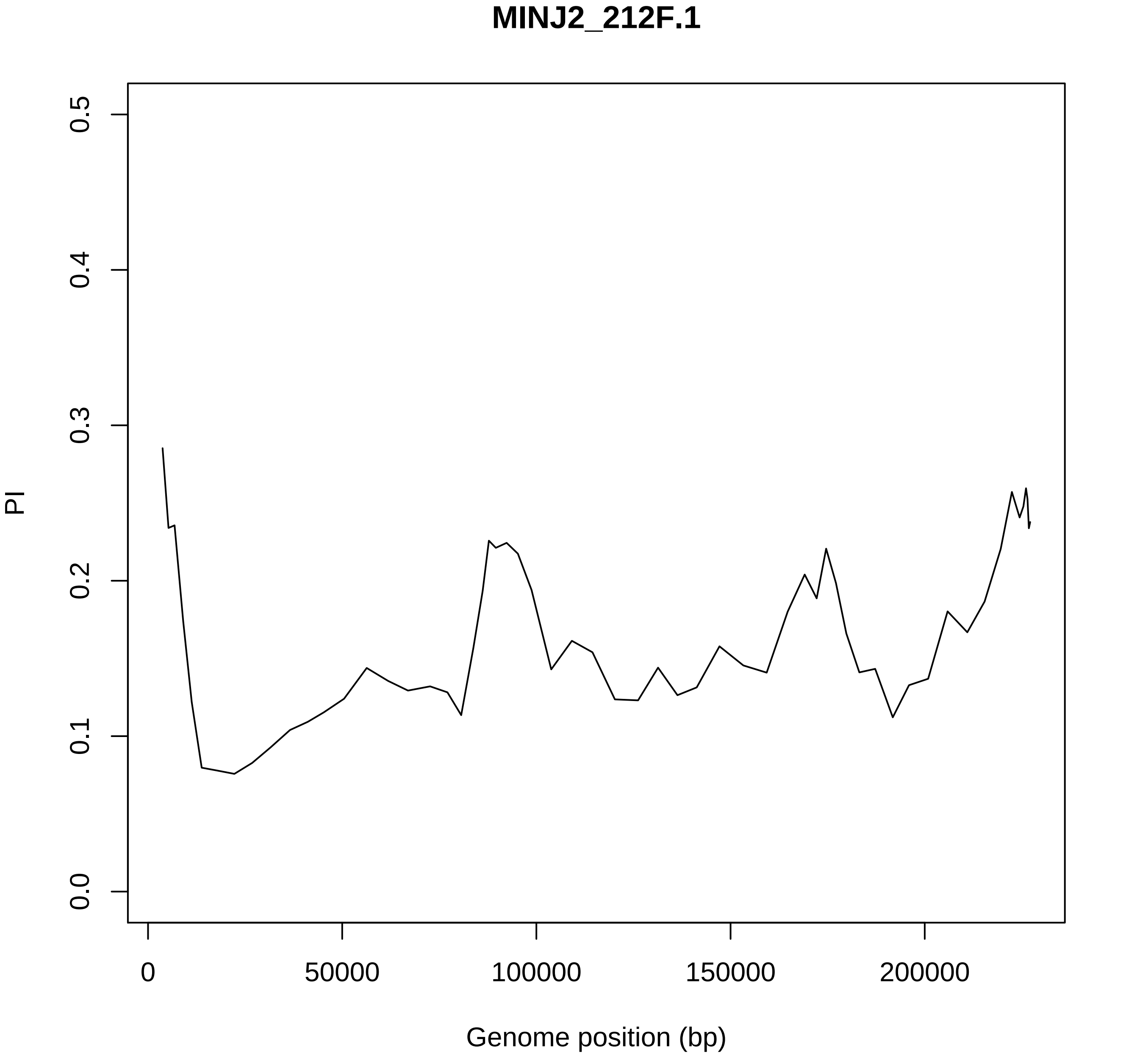

MINJ2_212F.1
PI
0.0
0.1
0.2
0.3
0.4
0.5
0
50000
100000
150000
200000
Genome position (bp)

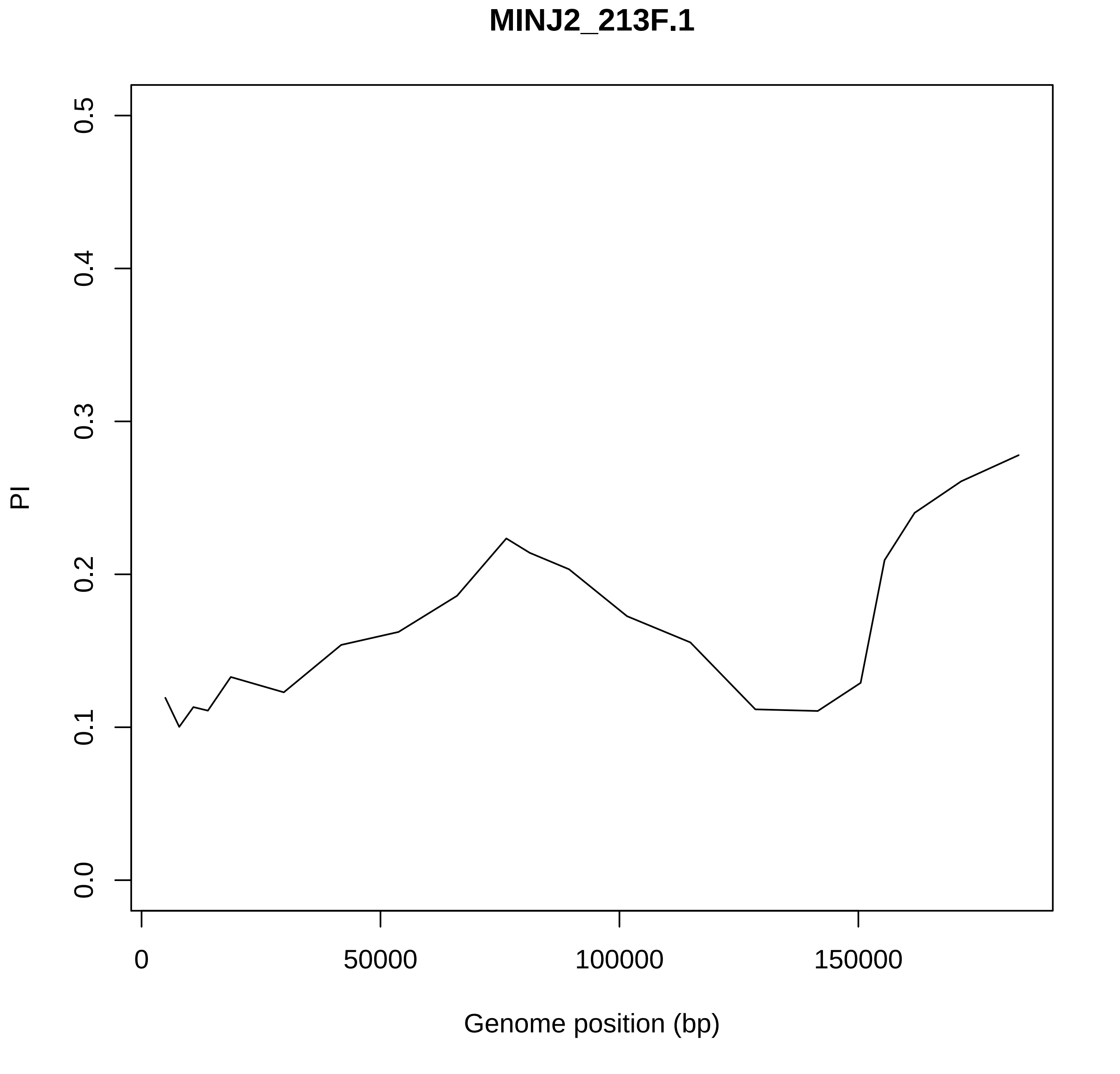
MINJ2_213F.1
PI
0.0
0.1
0.2
0.3
0.4
0.5
0
50000
100000
150000
Genome position (bp)

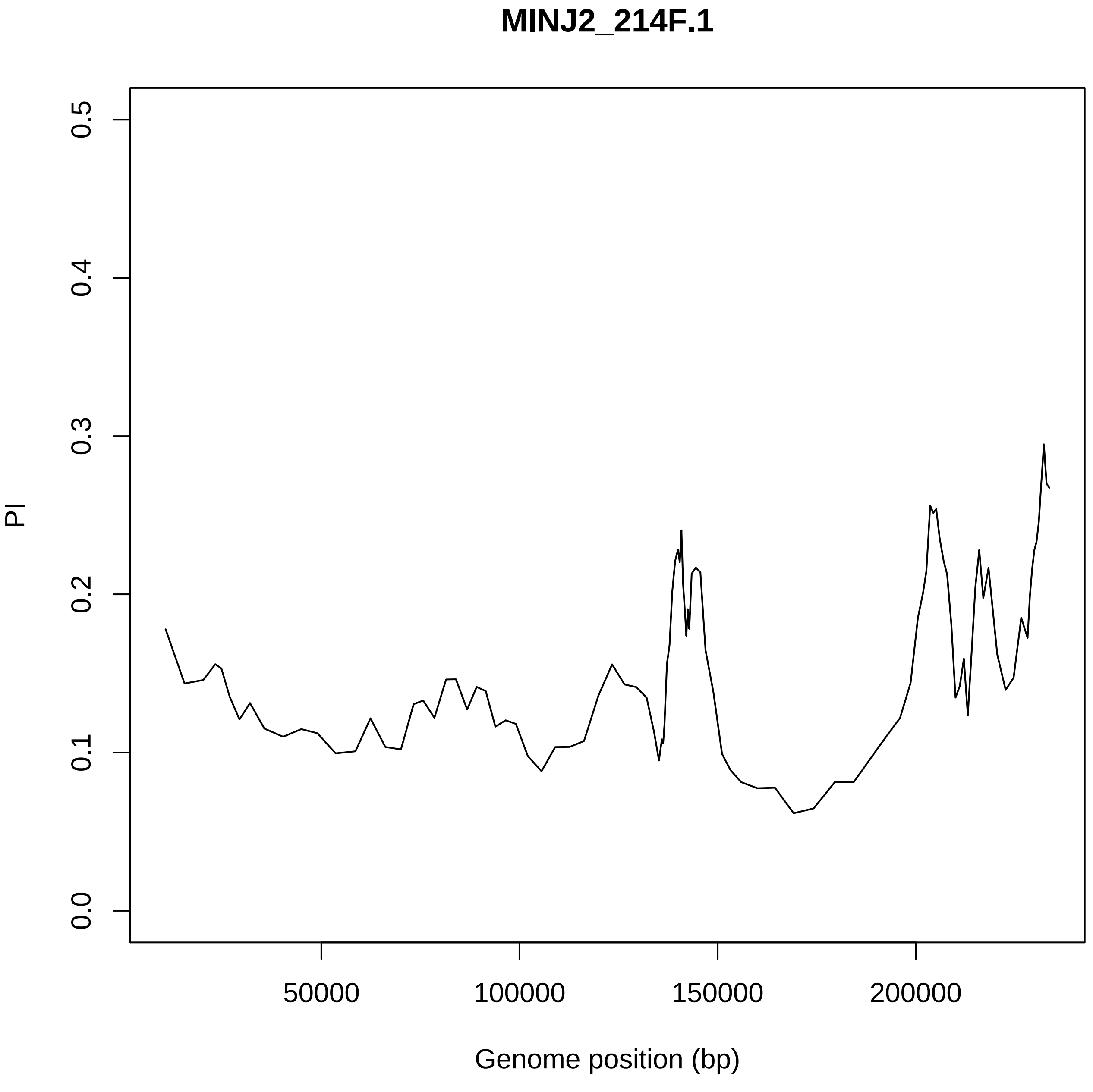

MINJ2_214F.1
PI
0.0
0.1
0.2
0.3
0.4
0.5
50000
100000
150000
200000
Genome position (bp)

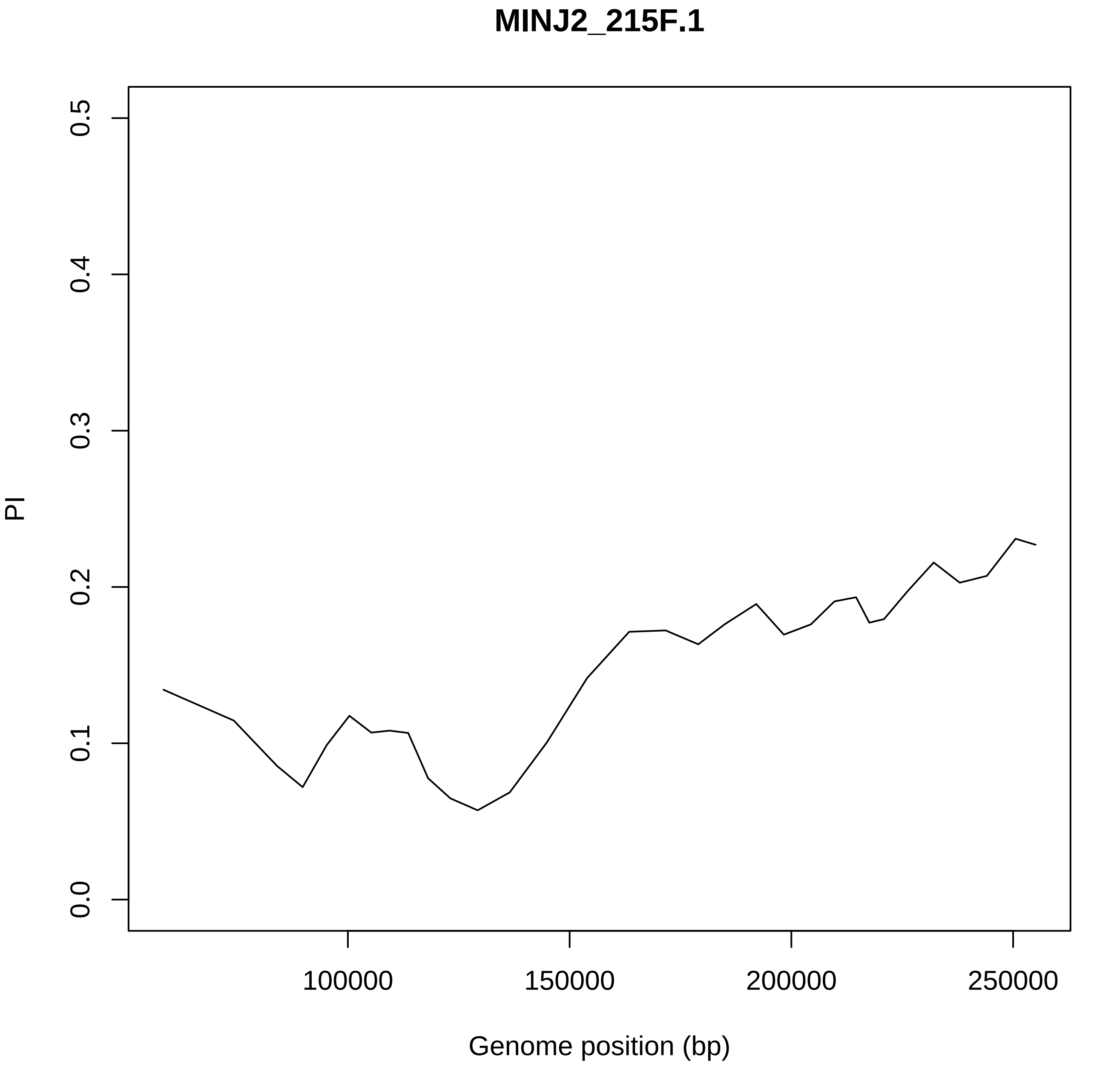

MINJ2_215F.1
PI
0.0
0.1
0.2
0.3
0.4
0.5
100000
150000
200000
250000
Genome position (bp)

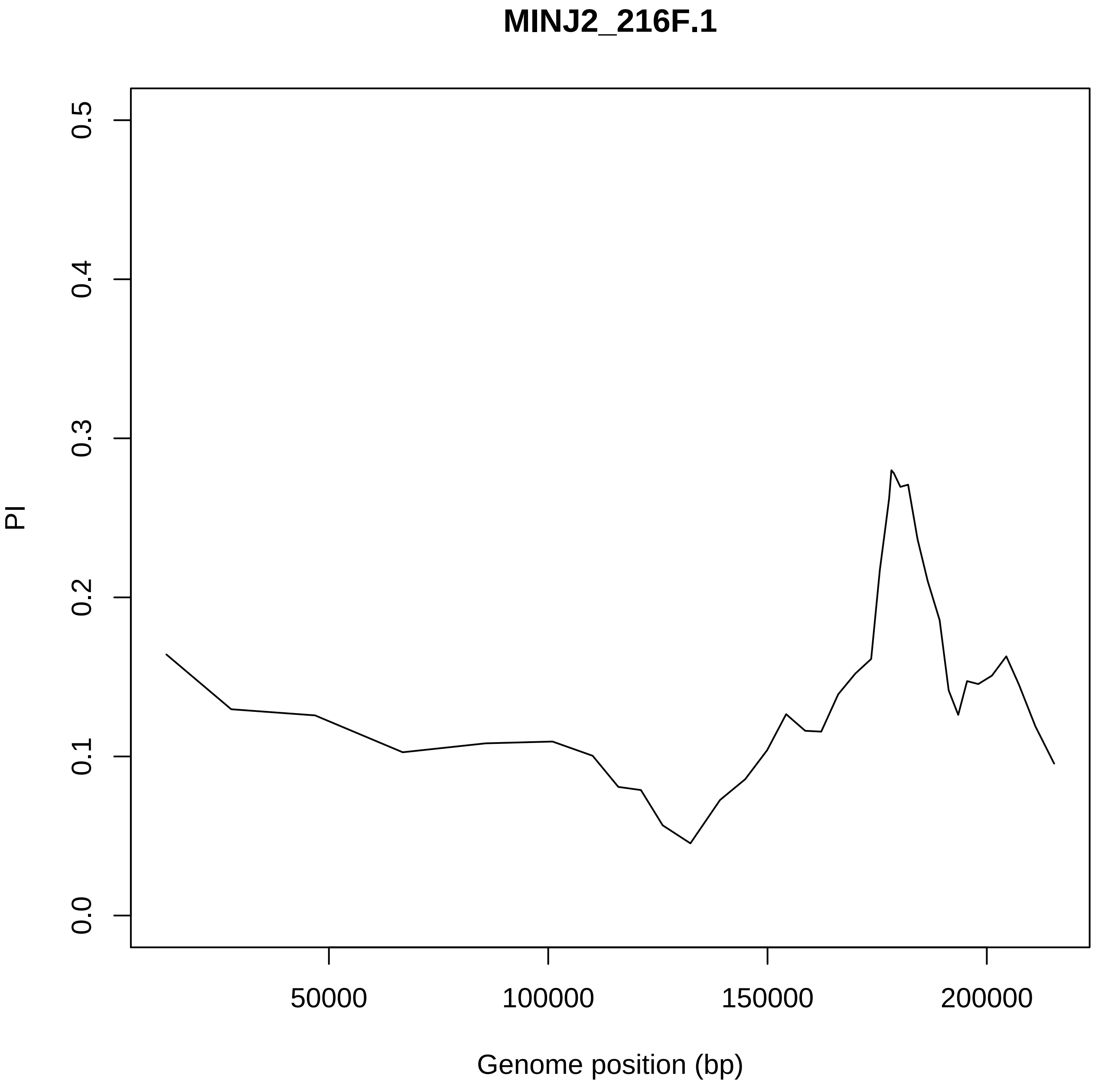

MINJ2_216F.1
PI
0.0
0.1
0.2
0.3
0.4
0.5
50000
100000
150000
200000
Genome position (bp)

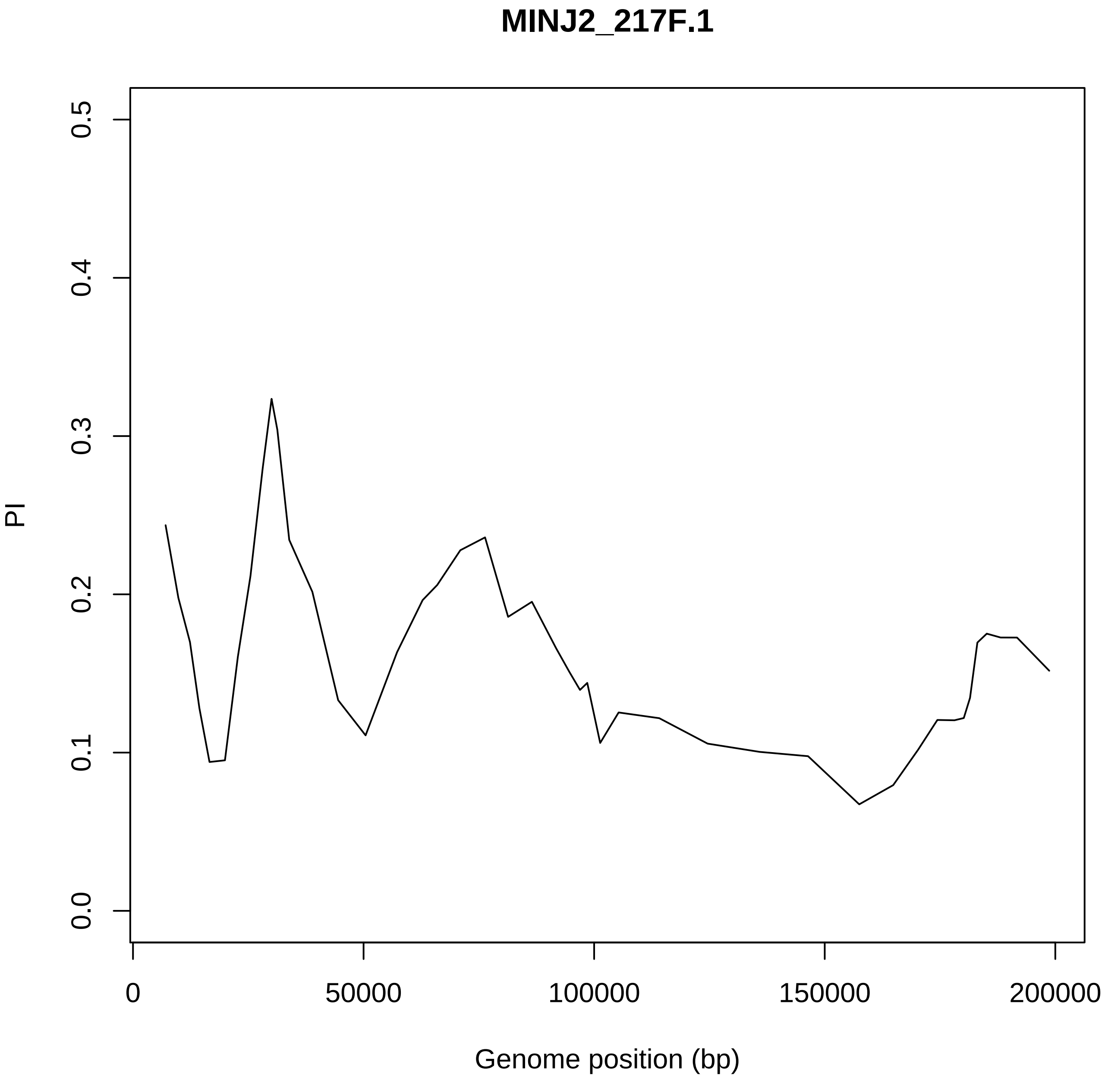
MINJ2_217F.1
PI
0.0
0.1
0.2
0.3
0.4
0.5
0
50000
100000
150000
200000
Genome position (bp)

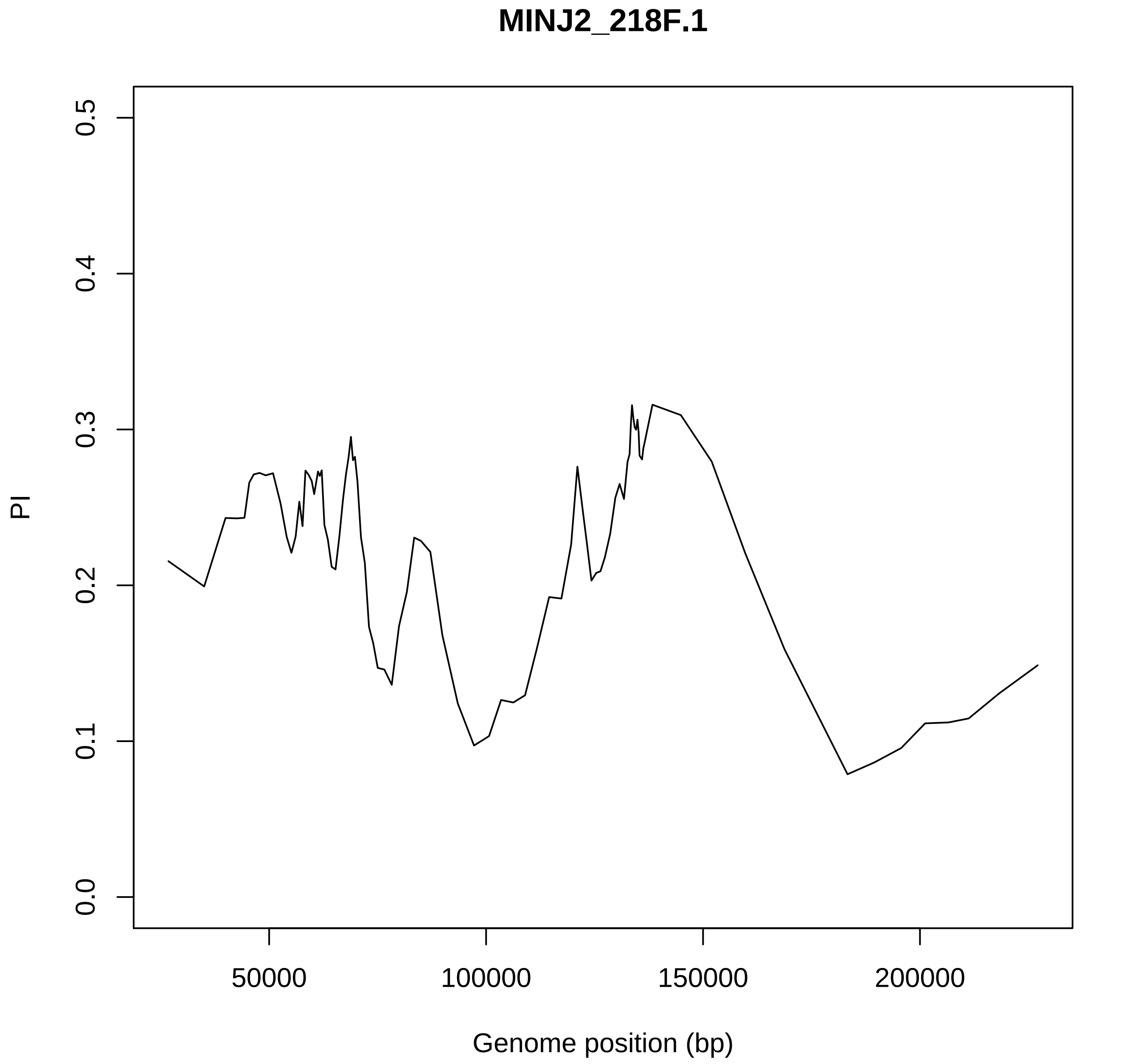
MINJ2_218F.1
PI
0.0
0.1
0.2
0.3
0.4
0.5
50000
100000
150000
200000
Genome position (bp)

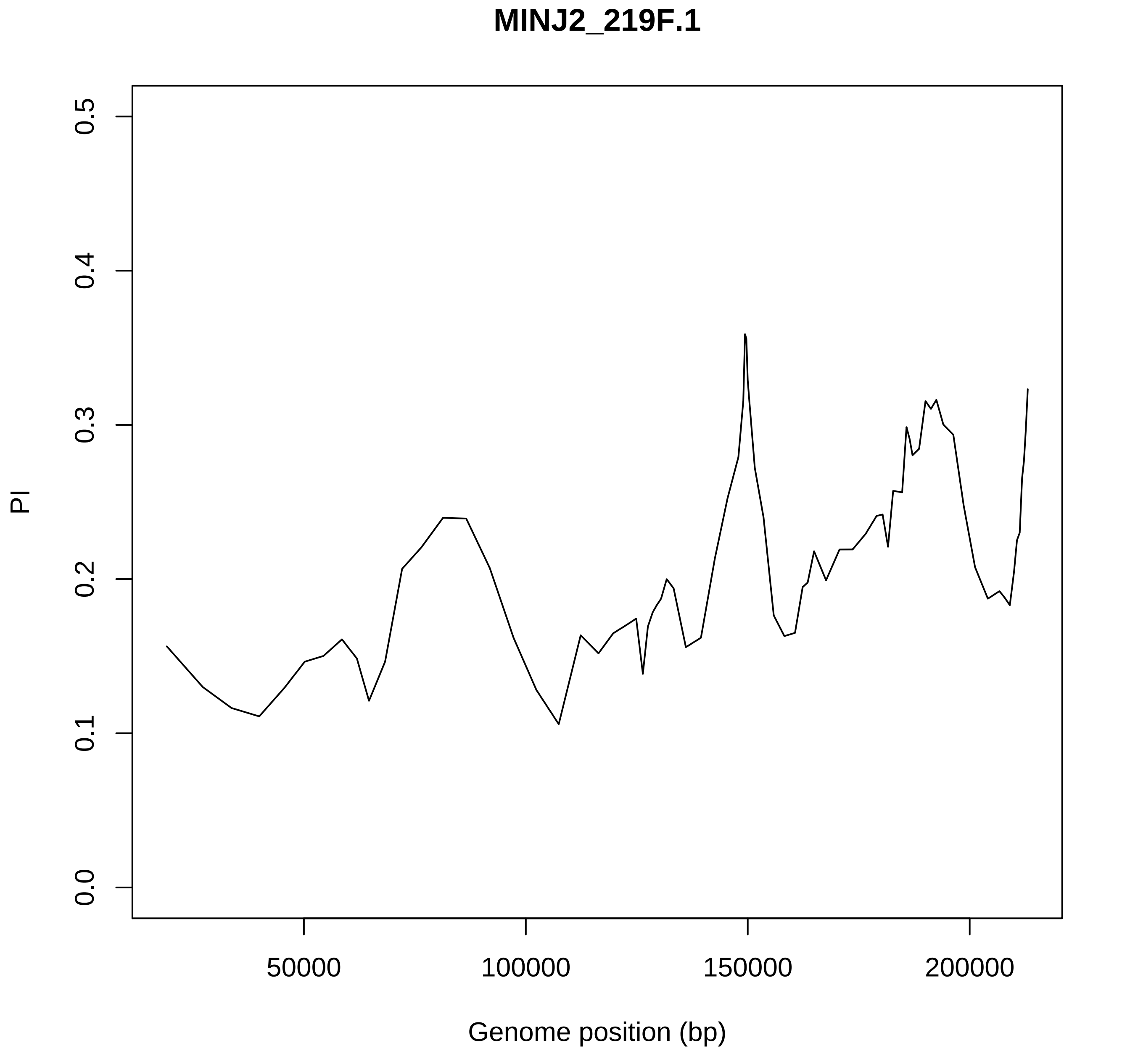
MINJ2_219F.1
PI
0.0
0.1
0.2
0.3
0.4
0.5
50000
100000
150000
200000
Genome position (bp)

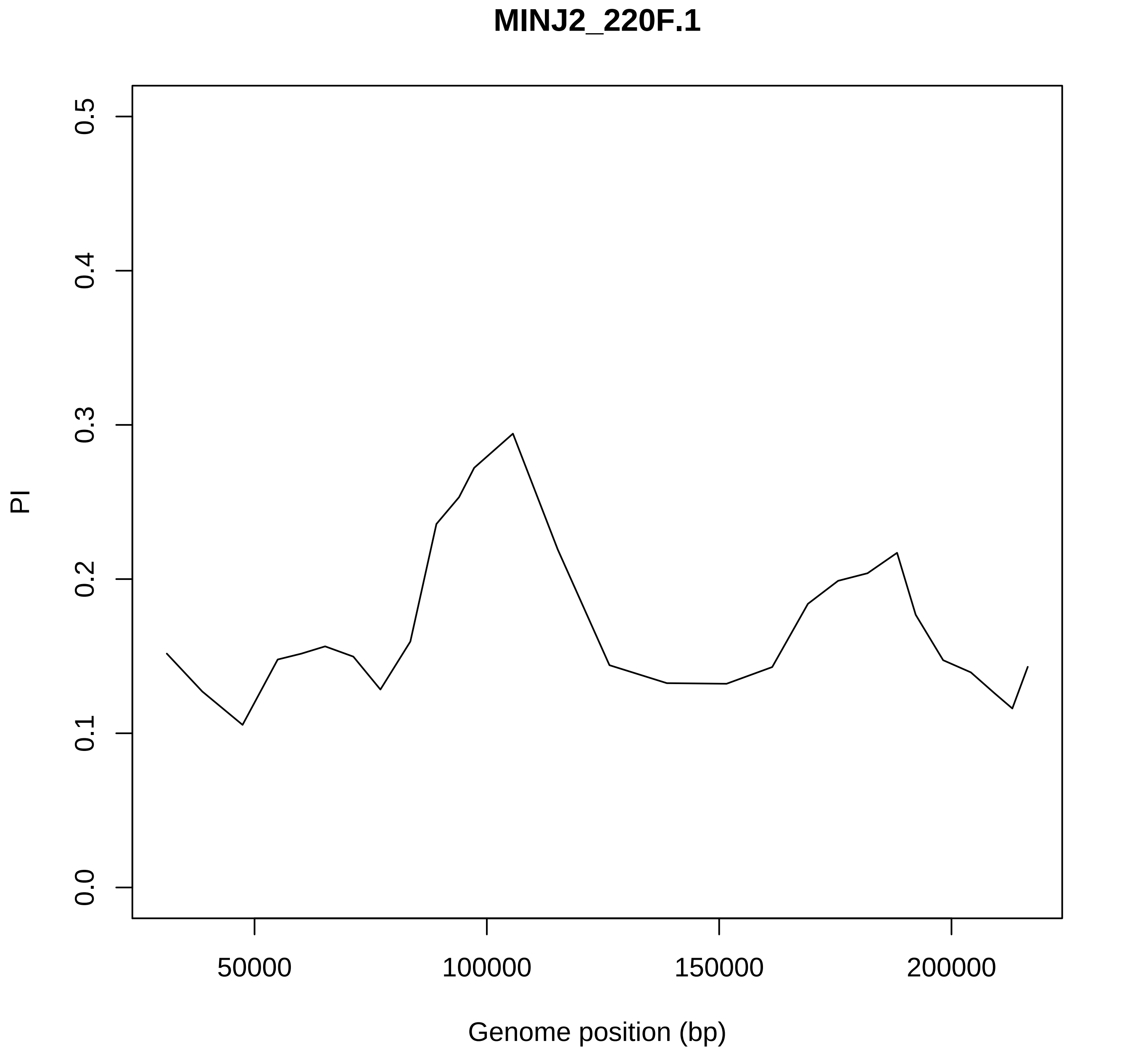
MINJ2_220F.1
PI
0.0
0.1
0.2
0.3
0.4
0.5
50000
100000
150000
200000
Genome position (bp)

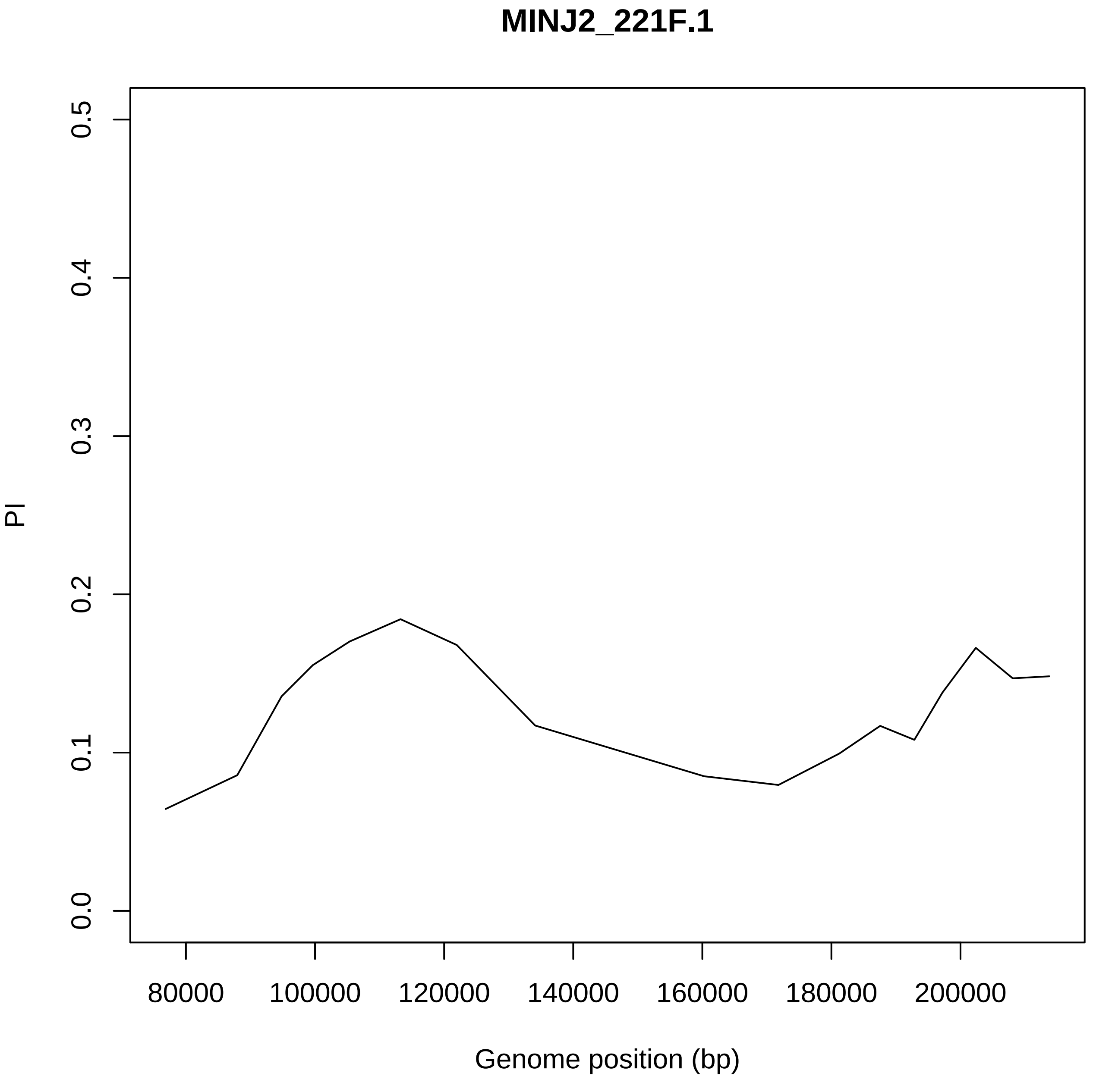
MINJ2_221F.1
PI
0.0
0.1
0.2
0.3
0.4
0.5
80000
100000
120000
140000
160000
180000
200000
Genome position (bp)

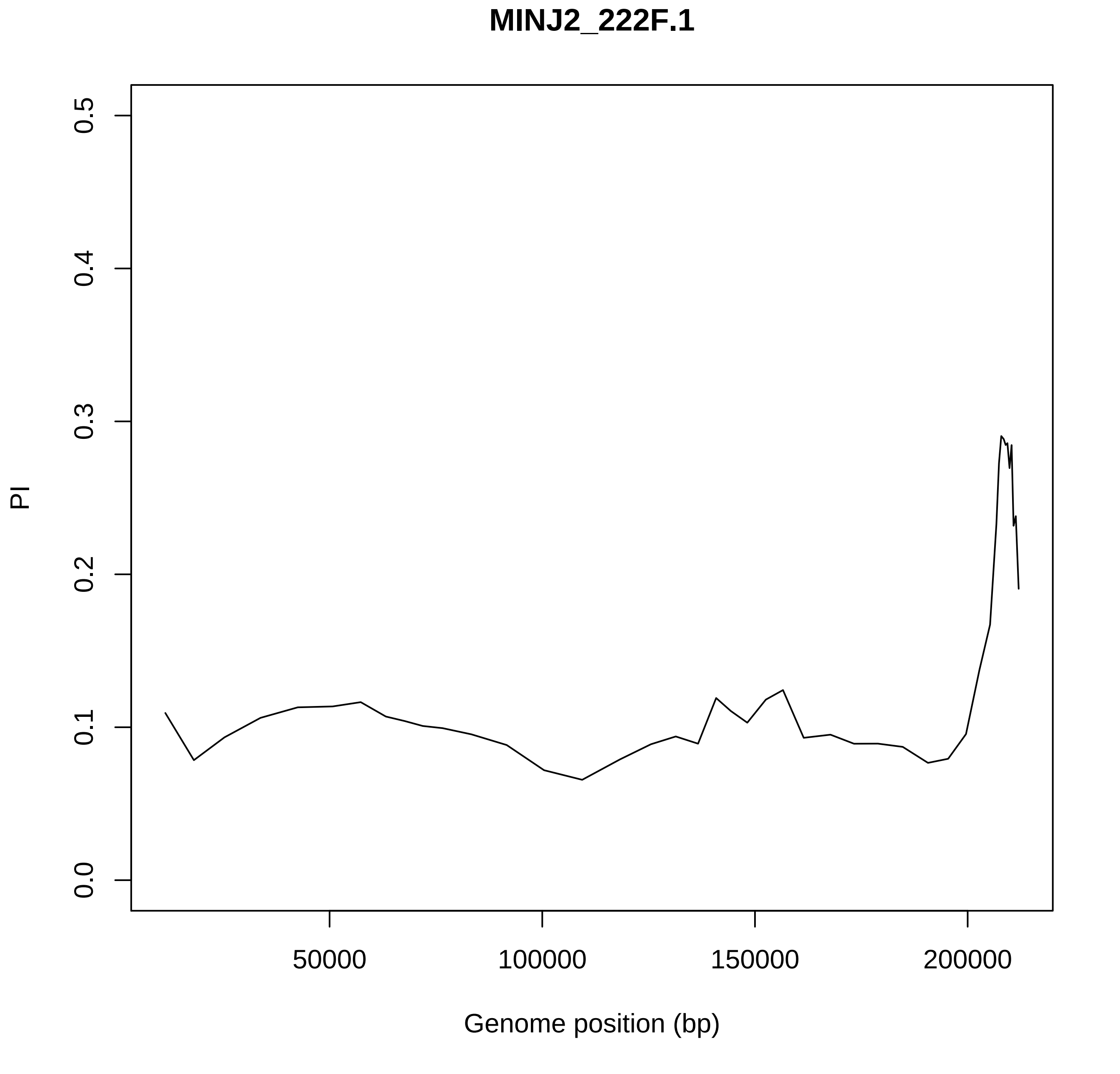

MINJ2_222F.1
PI
0.0
0.1
0.2
0.3
0.4
0.5
50000
100000
150000
200000
Genome position (bp)

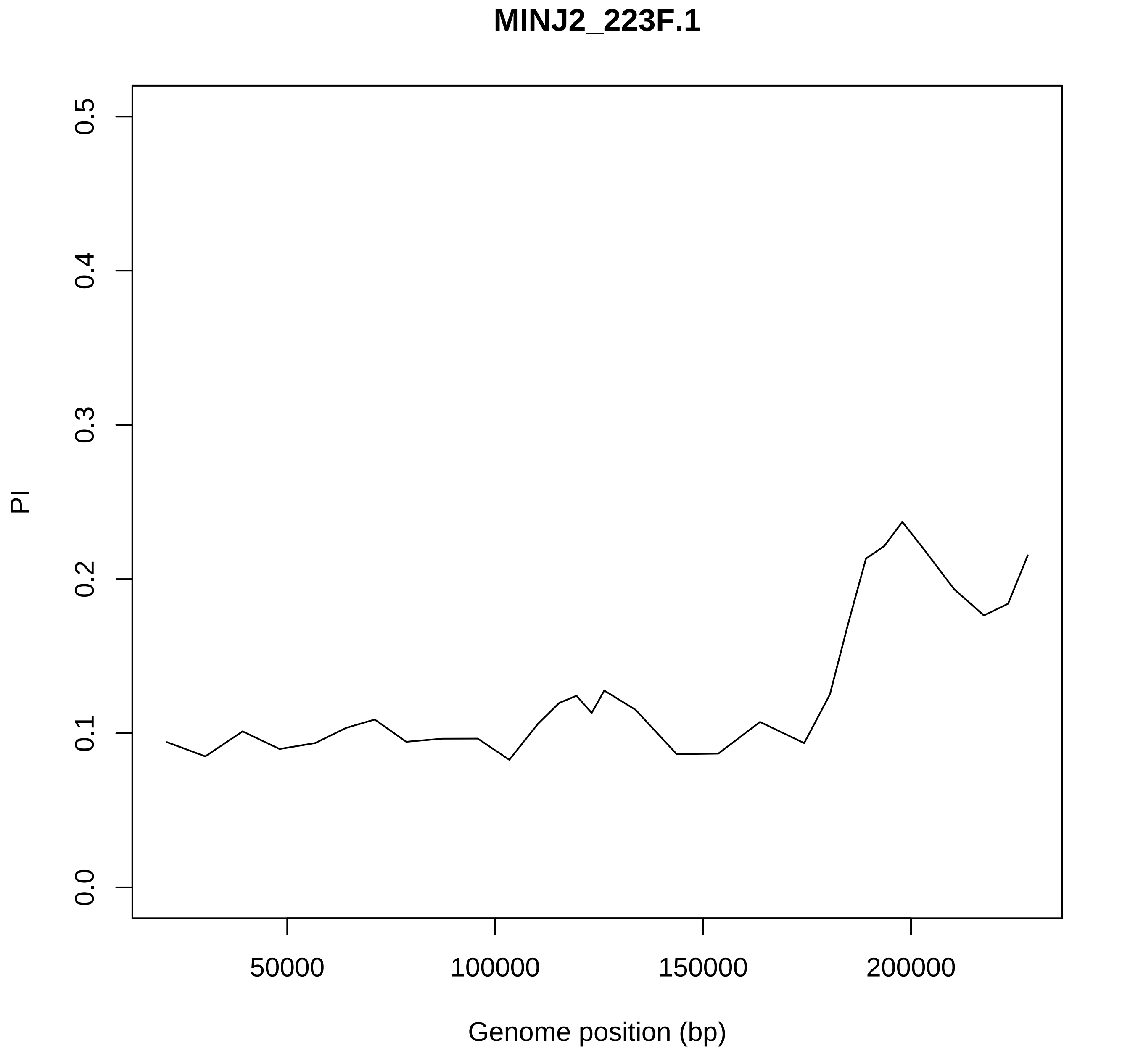
MINJ2_223F.1
PI
0.0
0.1
0.2
0.3
0.4
0.5
50000
100000
150000
200000
Genome position (bp)

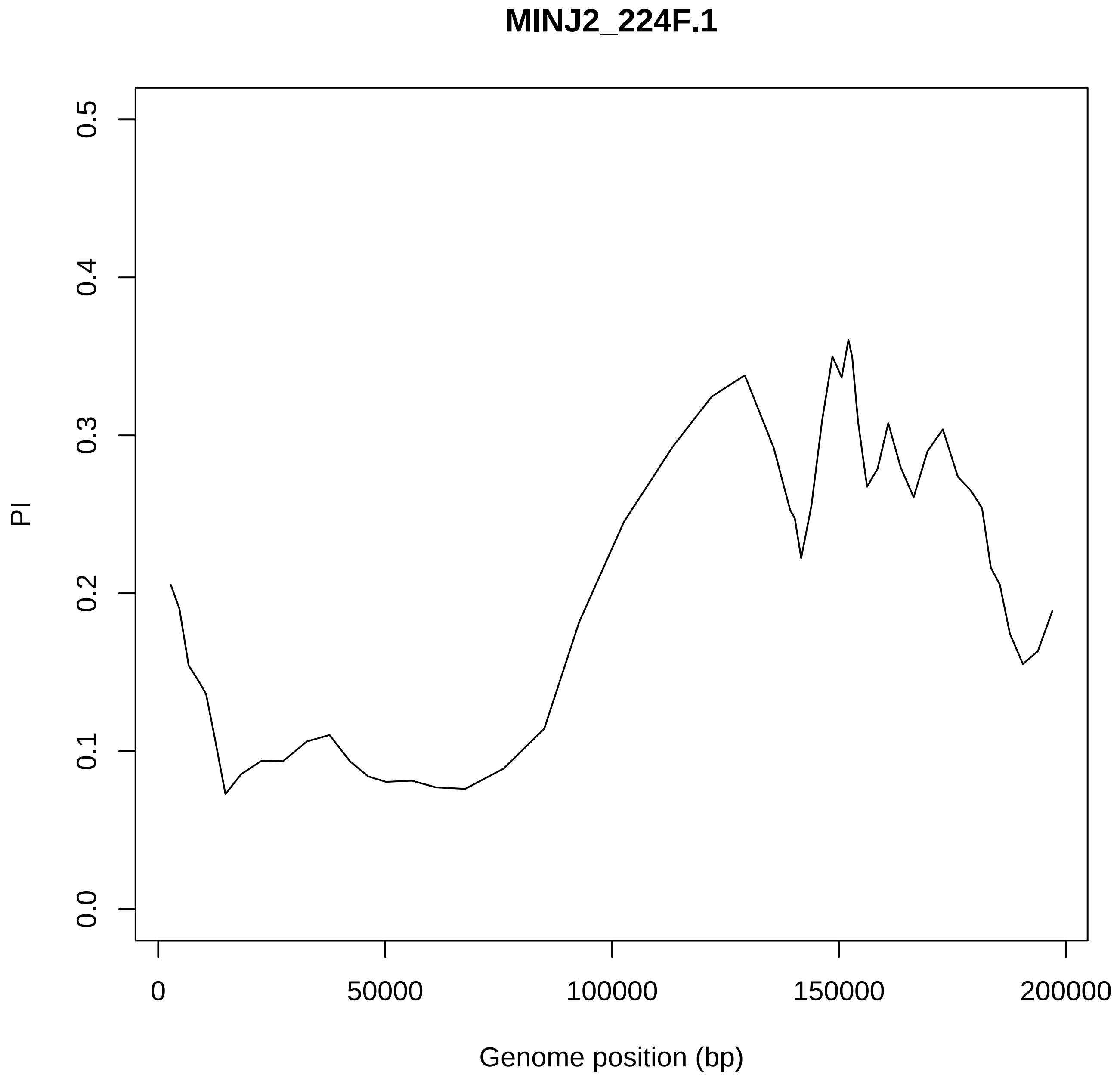
MINJ2_224F.1
PI
0.0
0.1
0.2
0.3
0.4
0.5
0
50000
100000
150000
200000
Genome position (bp)

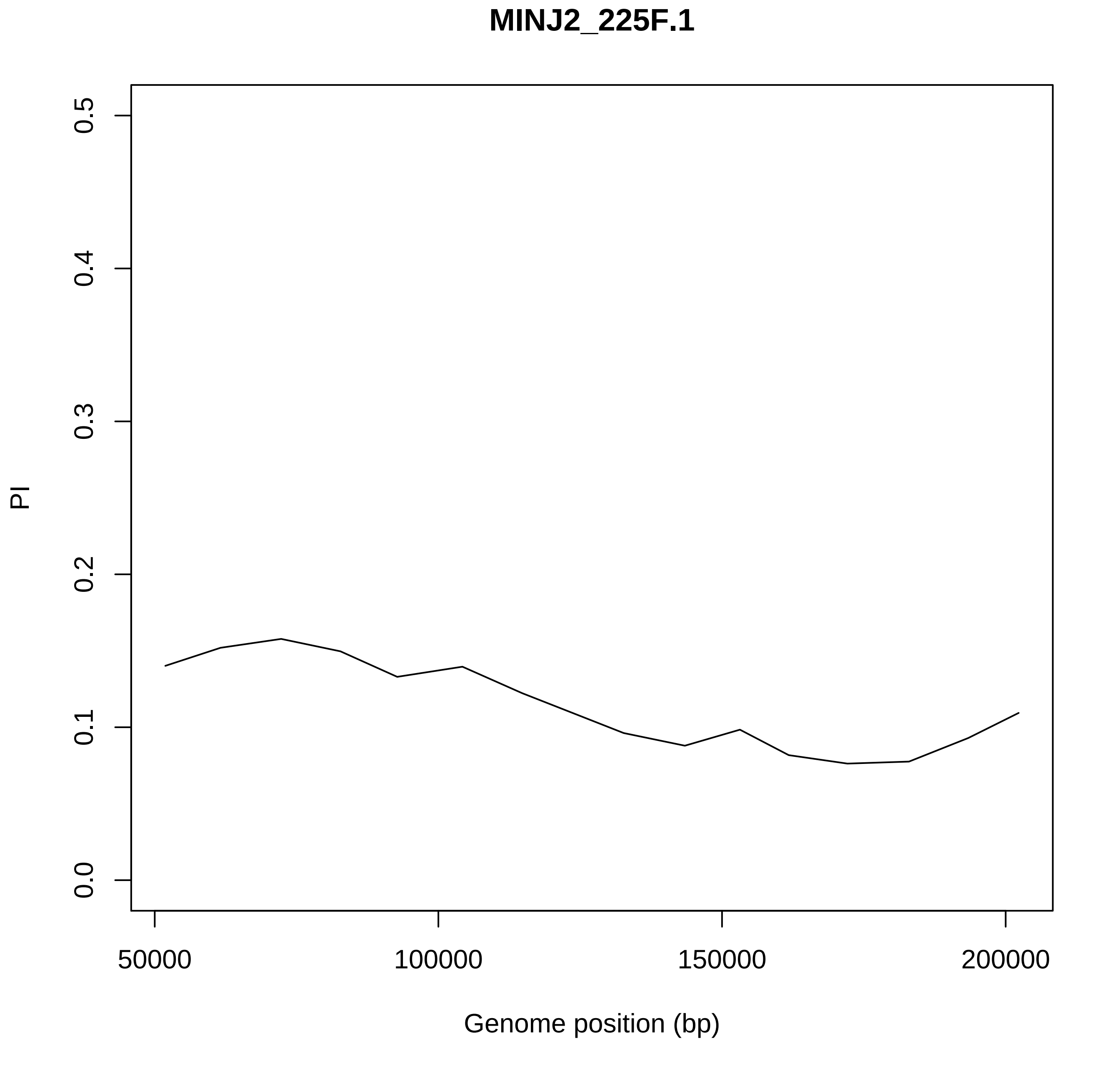
MINJ2_225F.1
PI
0.0
0.1
0.2
0.3
0.4
0.5
50000
100000
150000
200000
Genome position (bp)

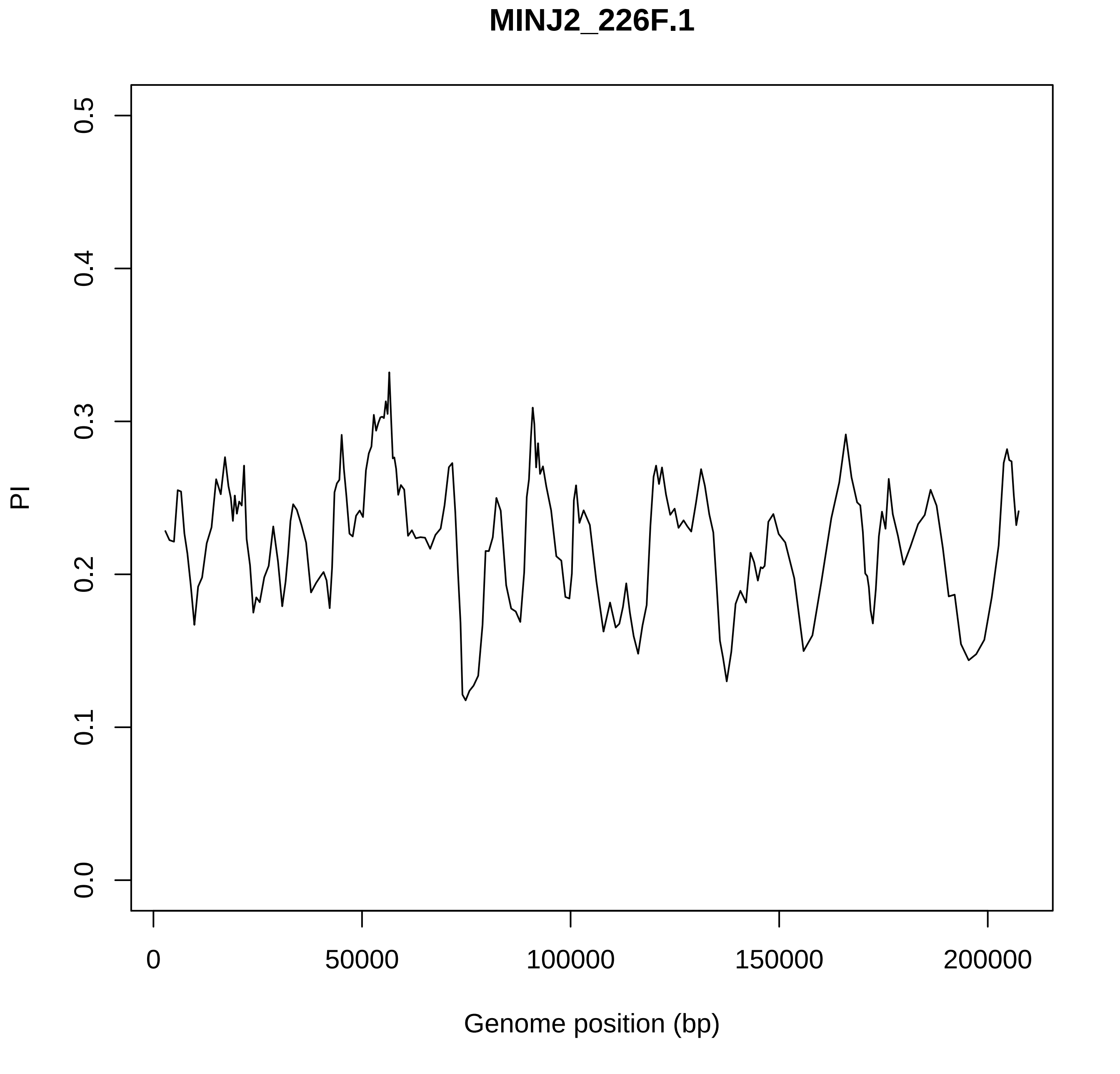
MINJ2_226F.1
PI
0.0
0.1
0.2
0.3
0.4
0.5
0
50000
100000
150000
200000
Genome position (bp)

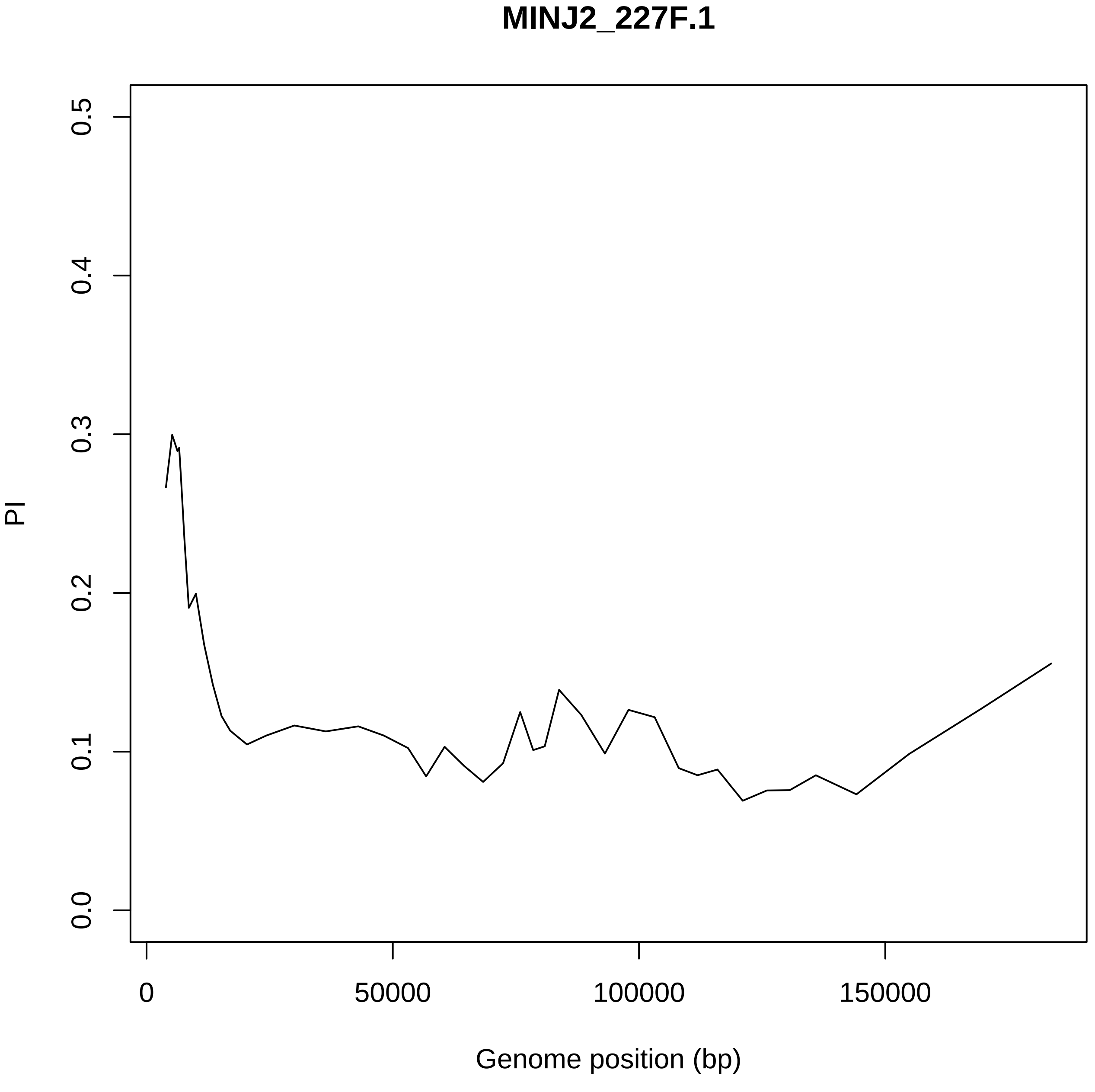
MINJ2_227F.1
PI
0.0
0.1
0.2
0.3
0.4
0.5
0
50000
100000
150000
Genome position (bp)

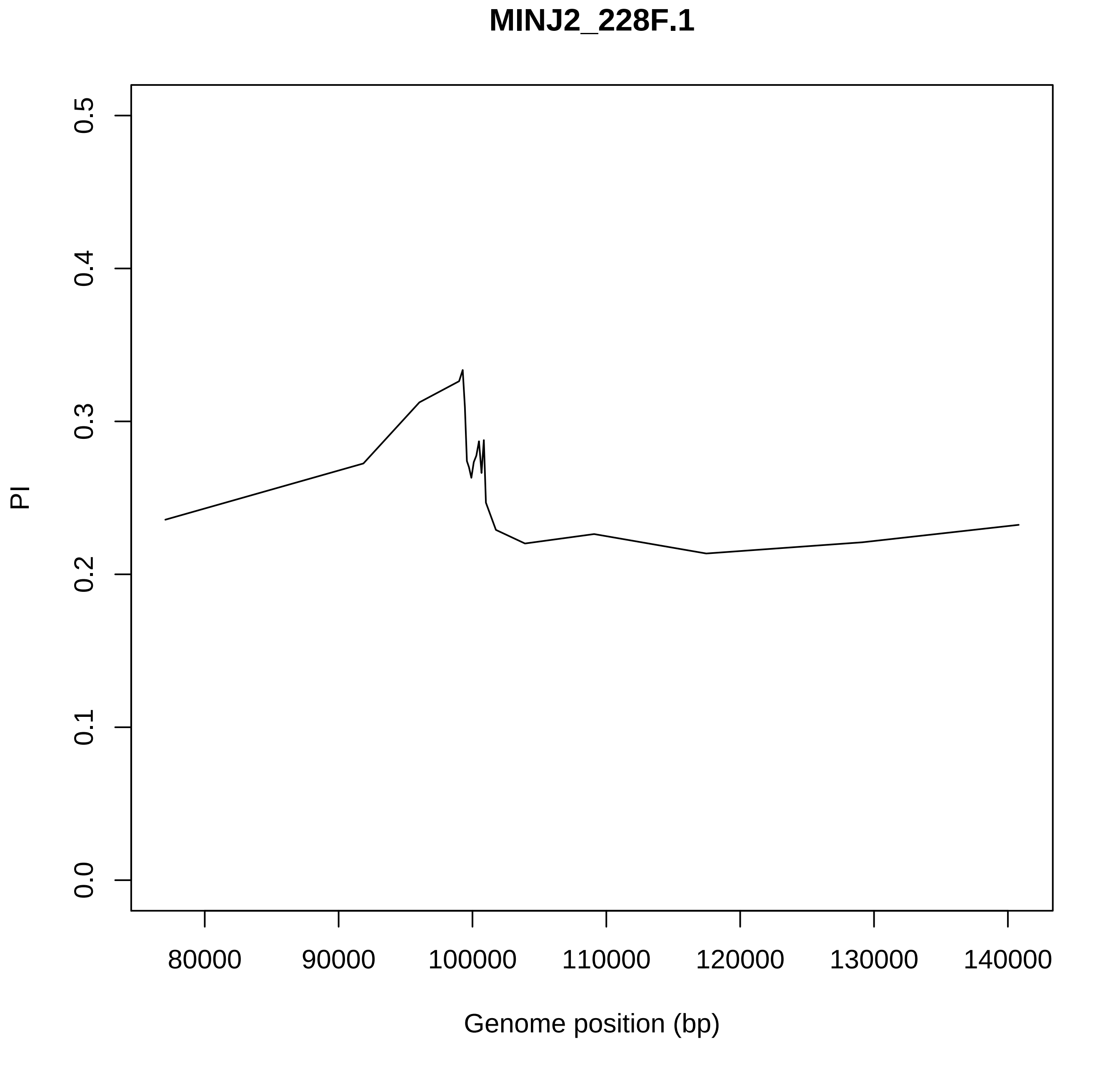
MINJ2_228F.1
PI
0.0
0.1
0.2
0.3
0.4
0.5
80000
90000
100000
110000
120000
130000
140000
Genome position (bp)

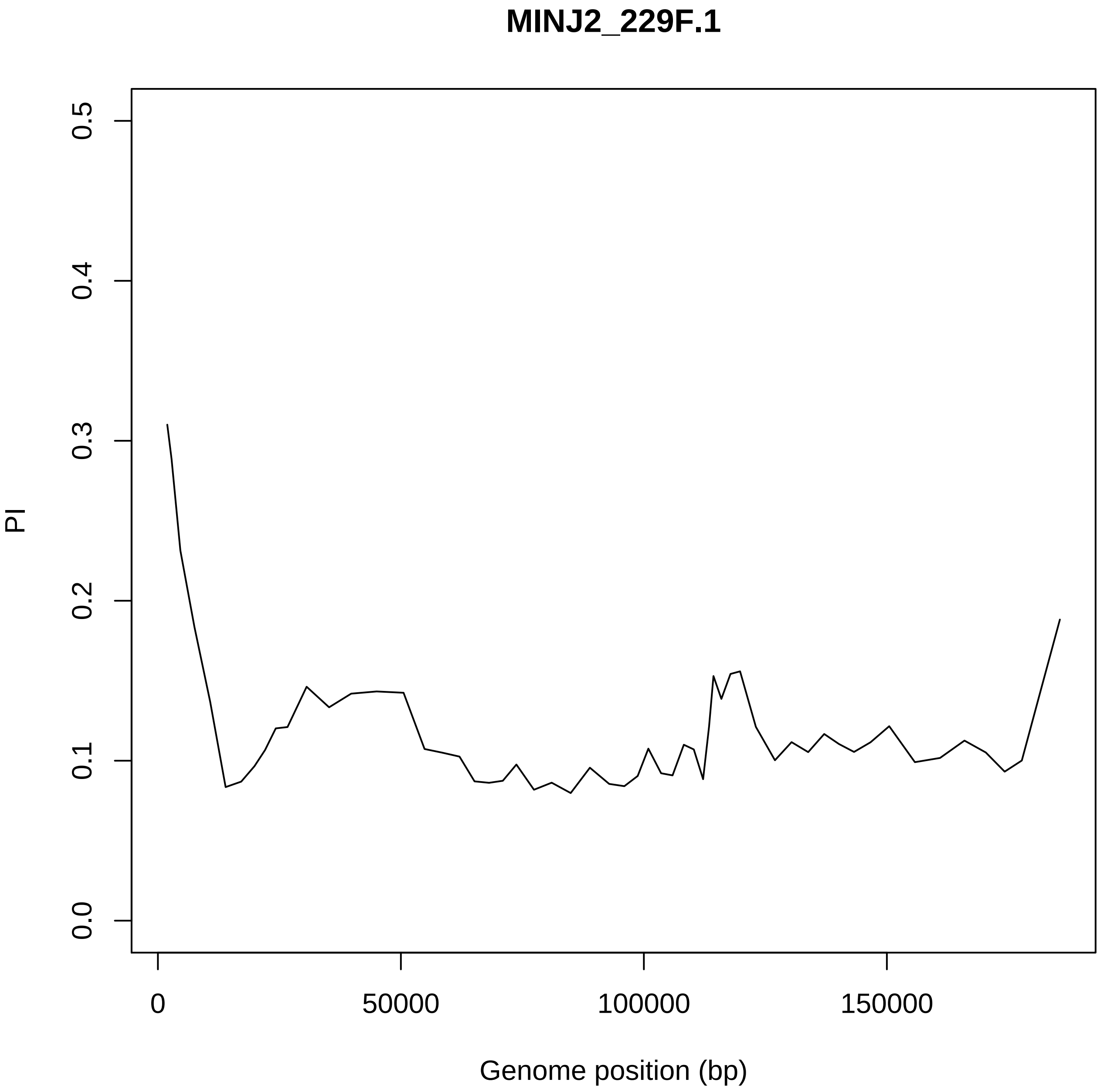
MINJ2_229F.1
PI
0.0
0.1
0.2
0.3
0.4
0.5
0
50000
100000
150000
Genome position (bp)

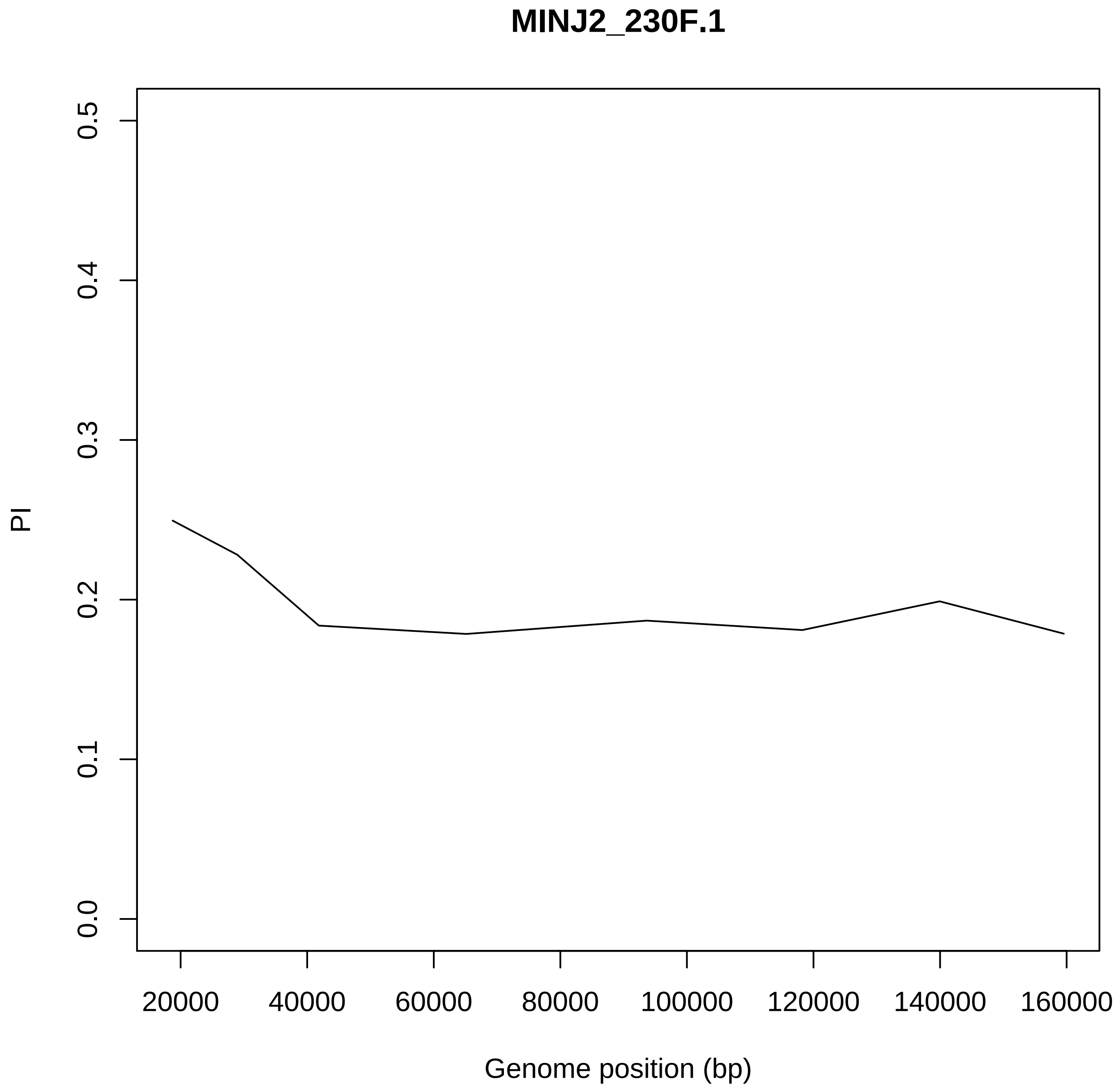
MINJ2_230F.1
PI
0.0
0.1
0.2
0.3
0.4
0.5
20000
40000
60000
80000
100000
120000
140000
160000
Genome position (bp)

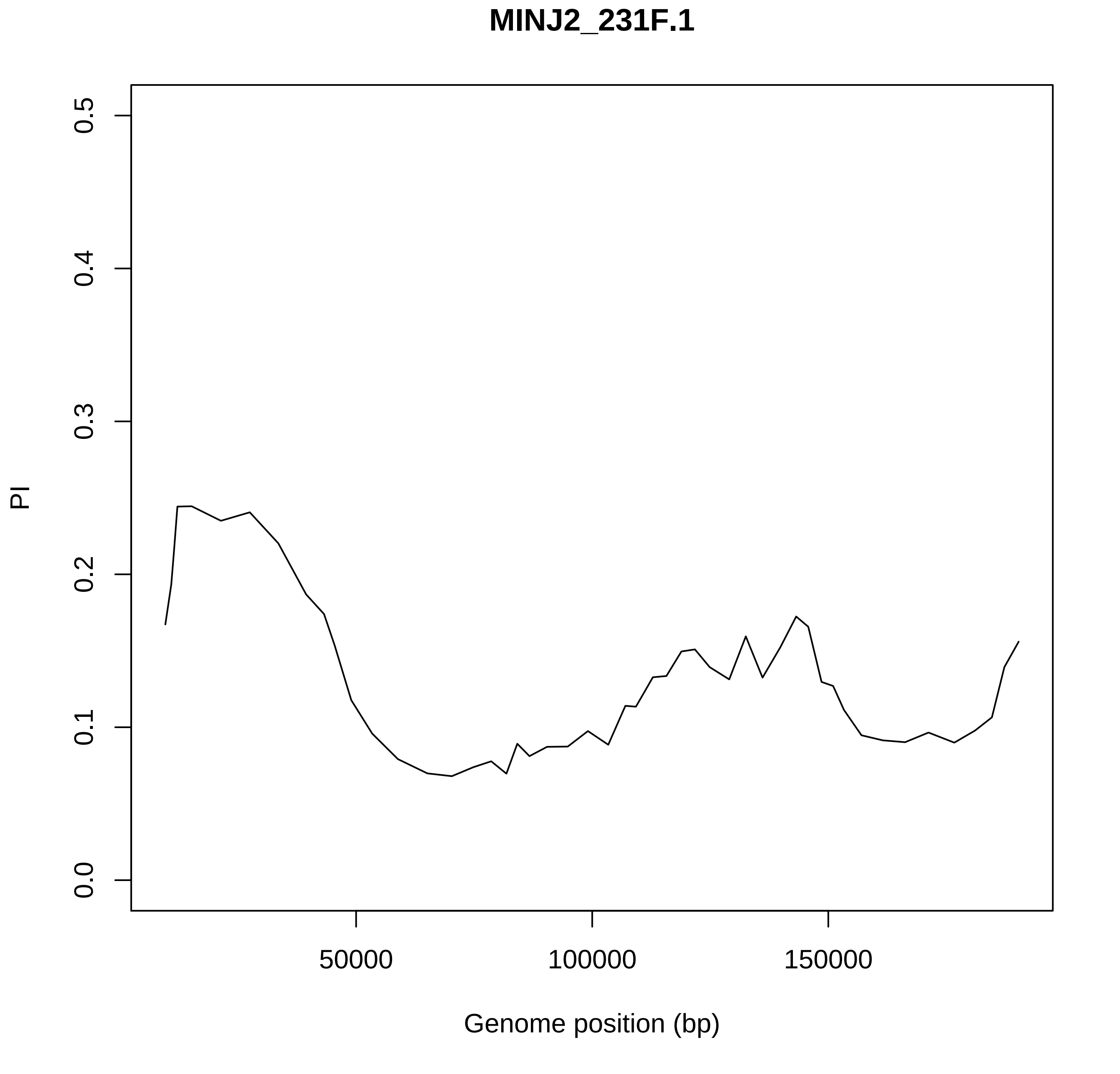
MINJ2_231F.1
PI
0.0
0.1
0.2
0.3
0.4
0.5
50000
100000
150000
Genome position (bp)

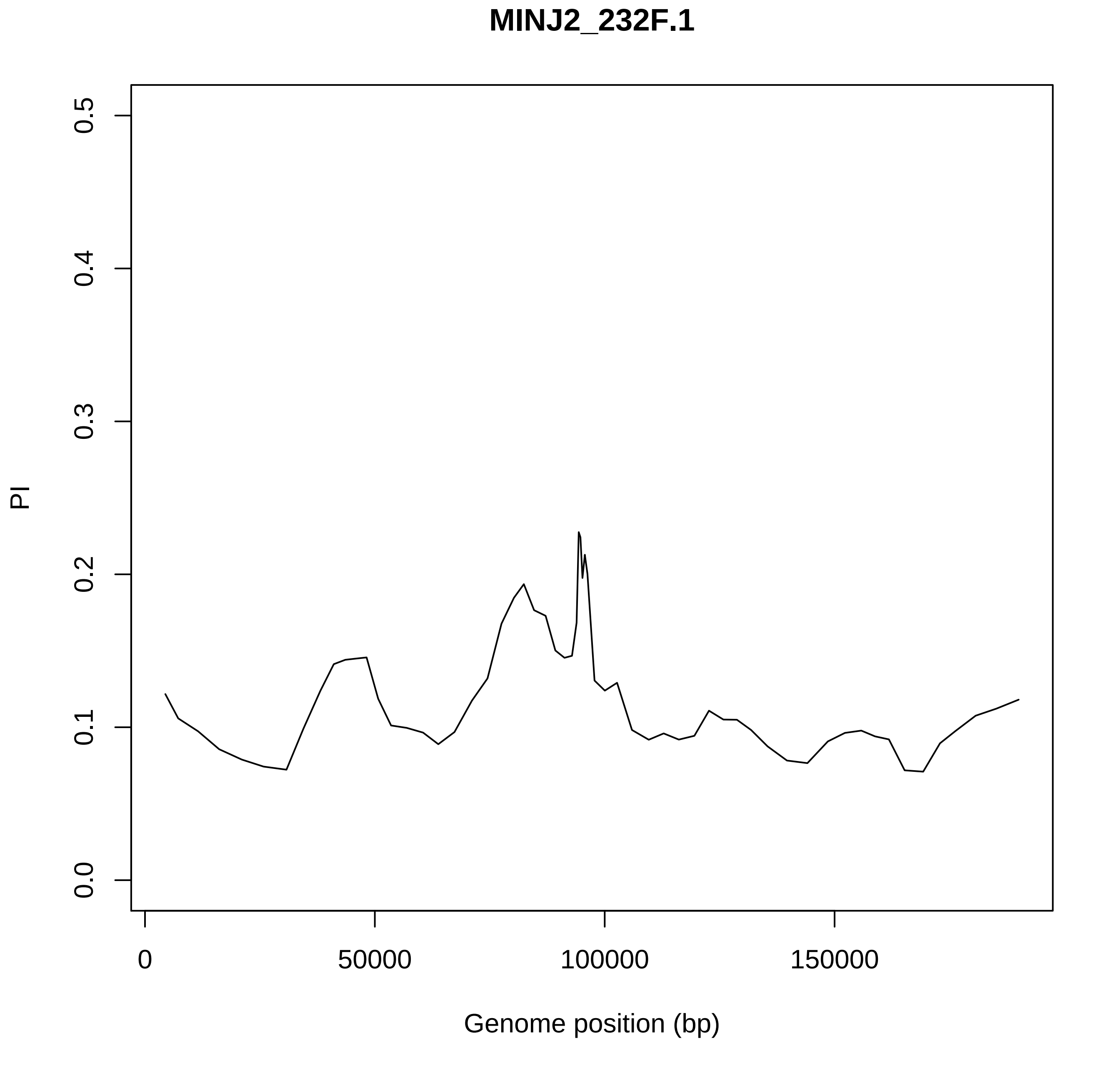
MINJ2_232F.1
PI
0.0
0.1
0.2
0.3
0.4
0.5
0
50000
100000
150000
Genome position (bp)

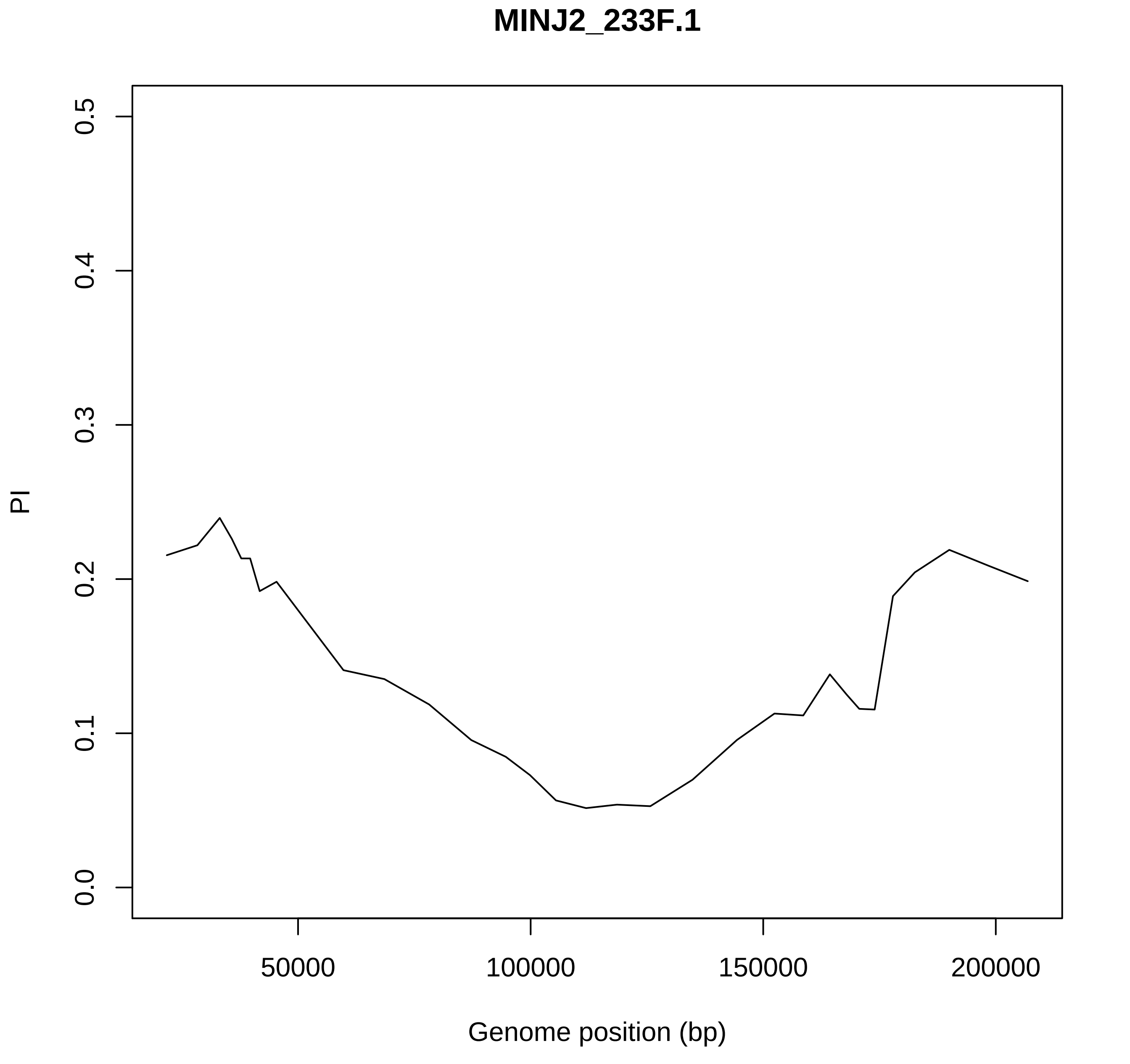

MINJ2_233F.1
PI
0.0
0.1
0.2
0.3
0.4
0.5
50000
100000
150000
200000
Genome position (bp)

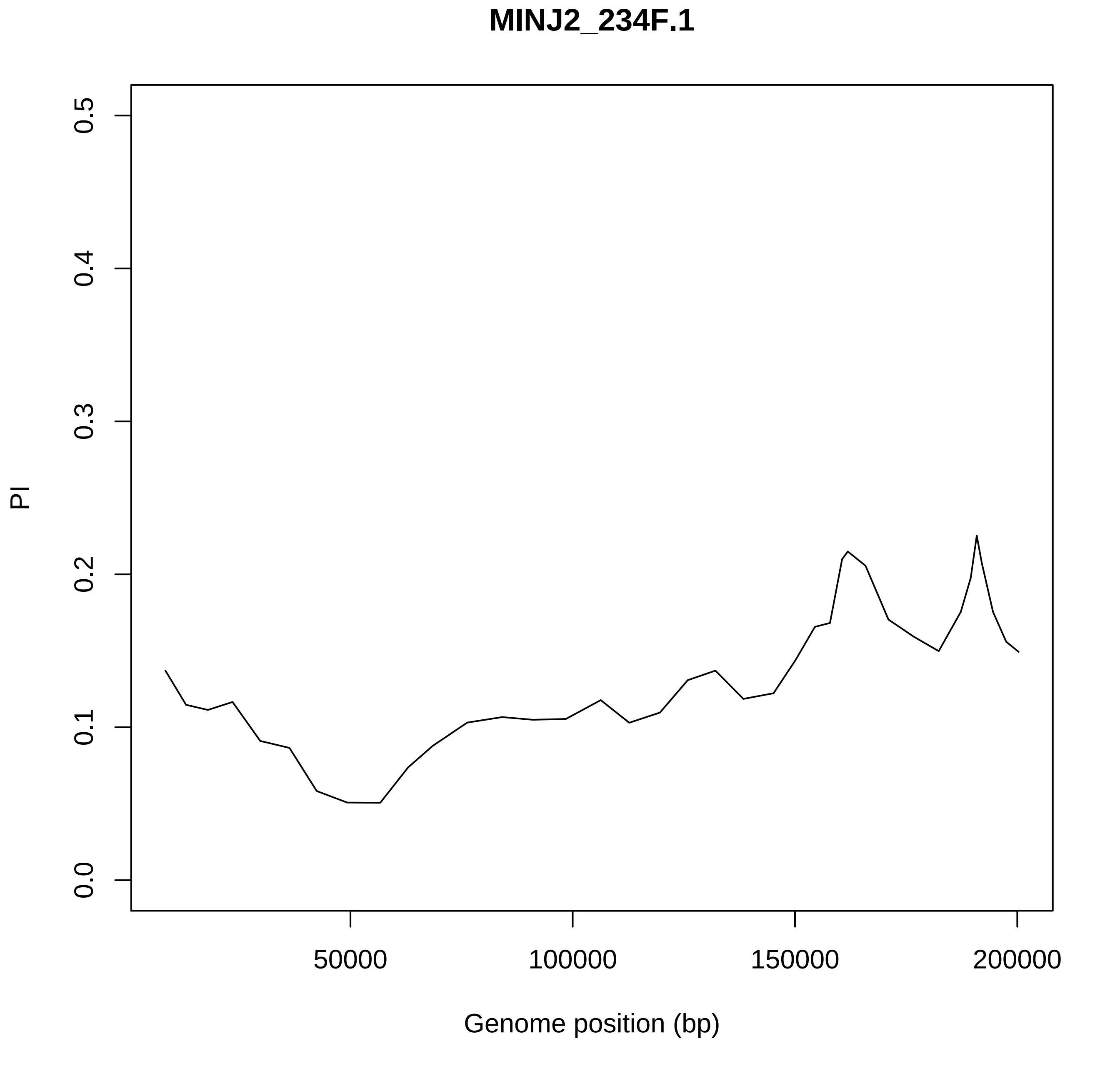
MINJ2_234F.1
PI
0.0
0.1
0.2
0.3
0.4
0.5
50000
100000
150000
200000
Genome position (bp)

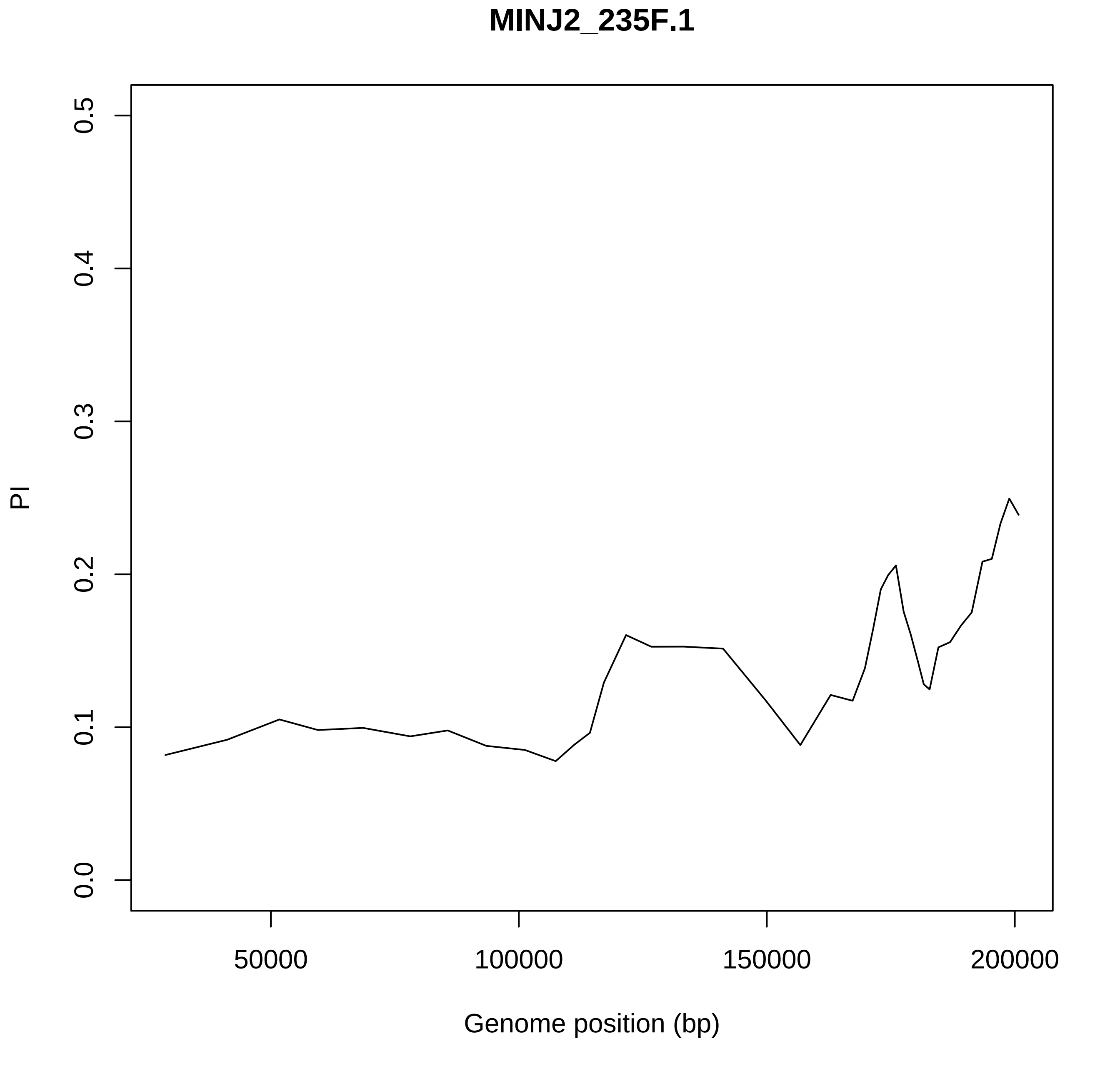
MINJ2_235F.1
PI
0.0
0.1
0.2
0.3
0.4
0.5
50000
100000
150000
200000
Genome position (bp)

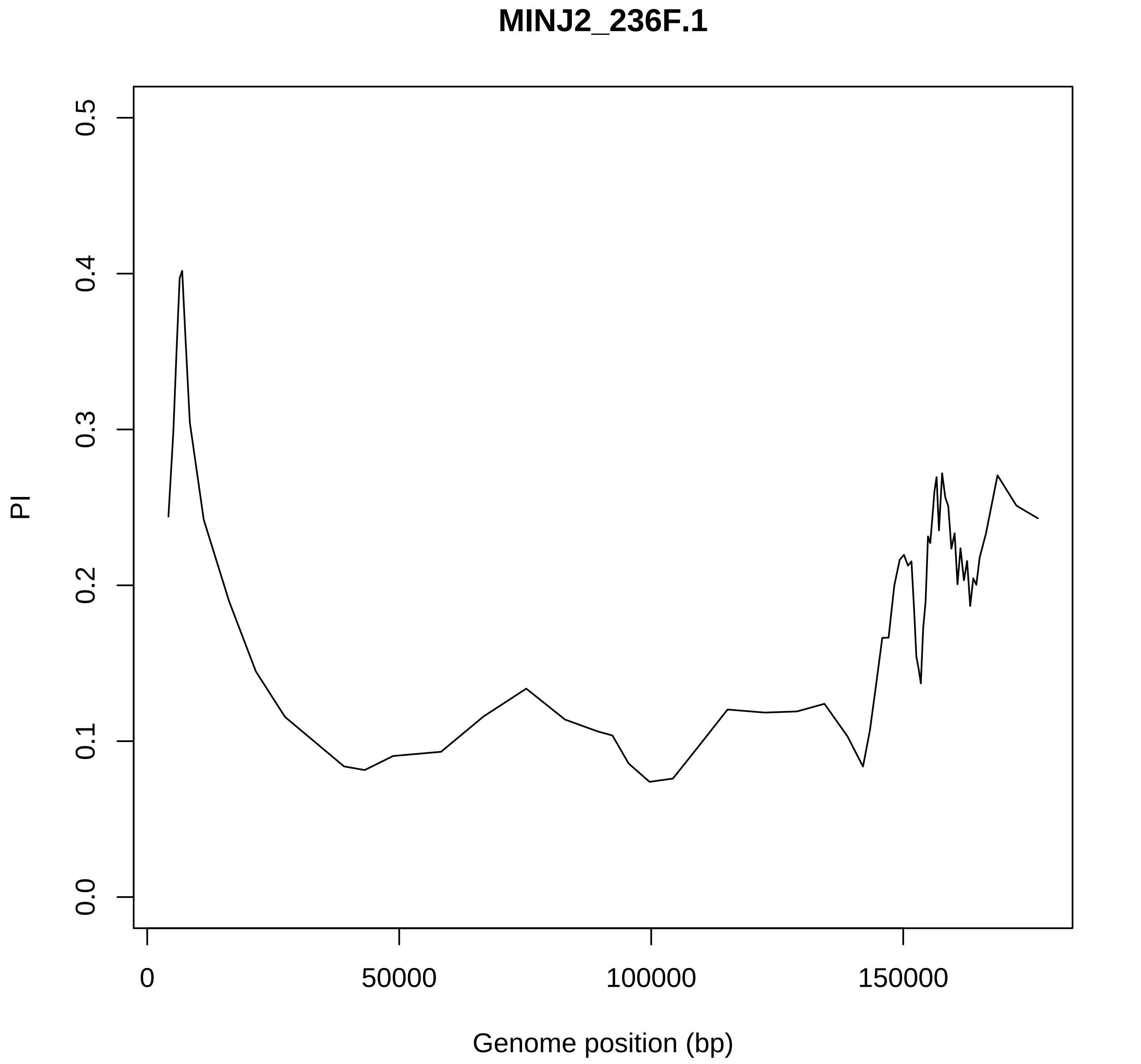
MINJ2_236F.1
PI
0.0
0.1
0.2
0.3
0.4
0.5
0
50000
100000
150000
Genome position (bp)

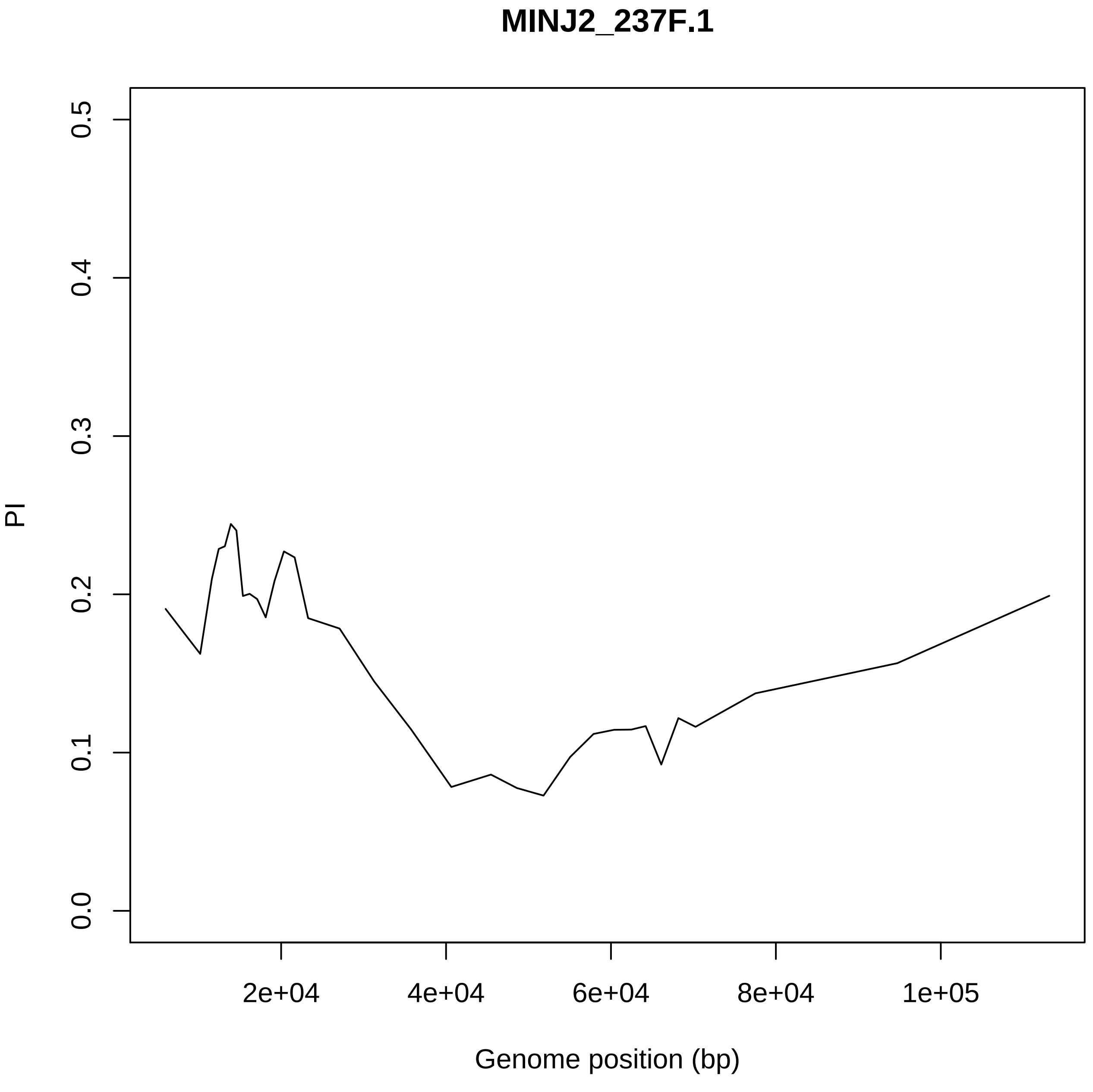
MINJ2_237F.1
PI
0.0
0.1
0.2
0.3
0.4
0.5
2e+04
4e+04
6e+04
8e+04
1e+05
Genome position (bp)

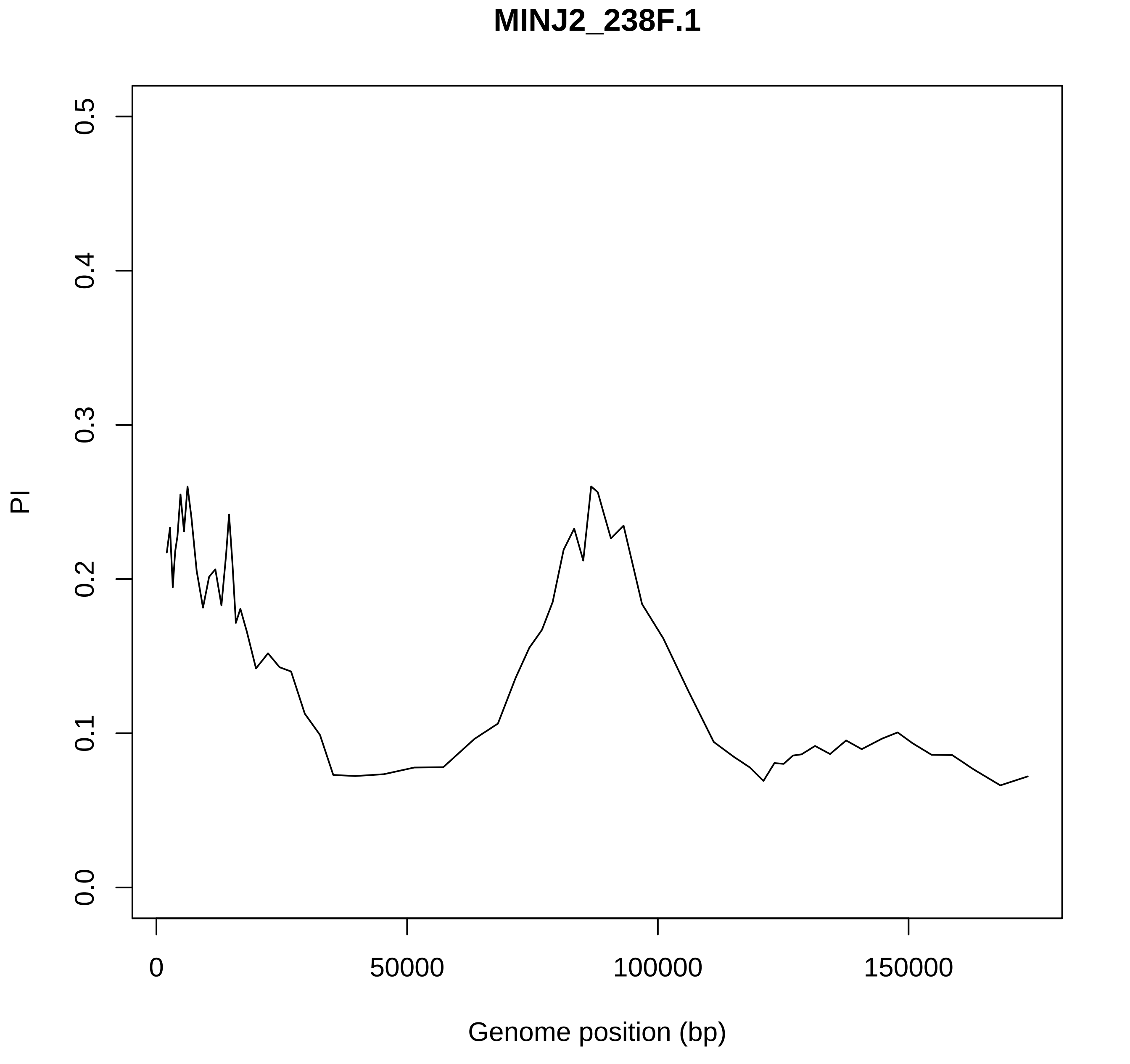

MINJ2_238F.1
PI
0.0
0.1
0.2
0.3
0.4
0.5
0
50000
100000
150000
Genome position (bp)

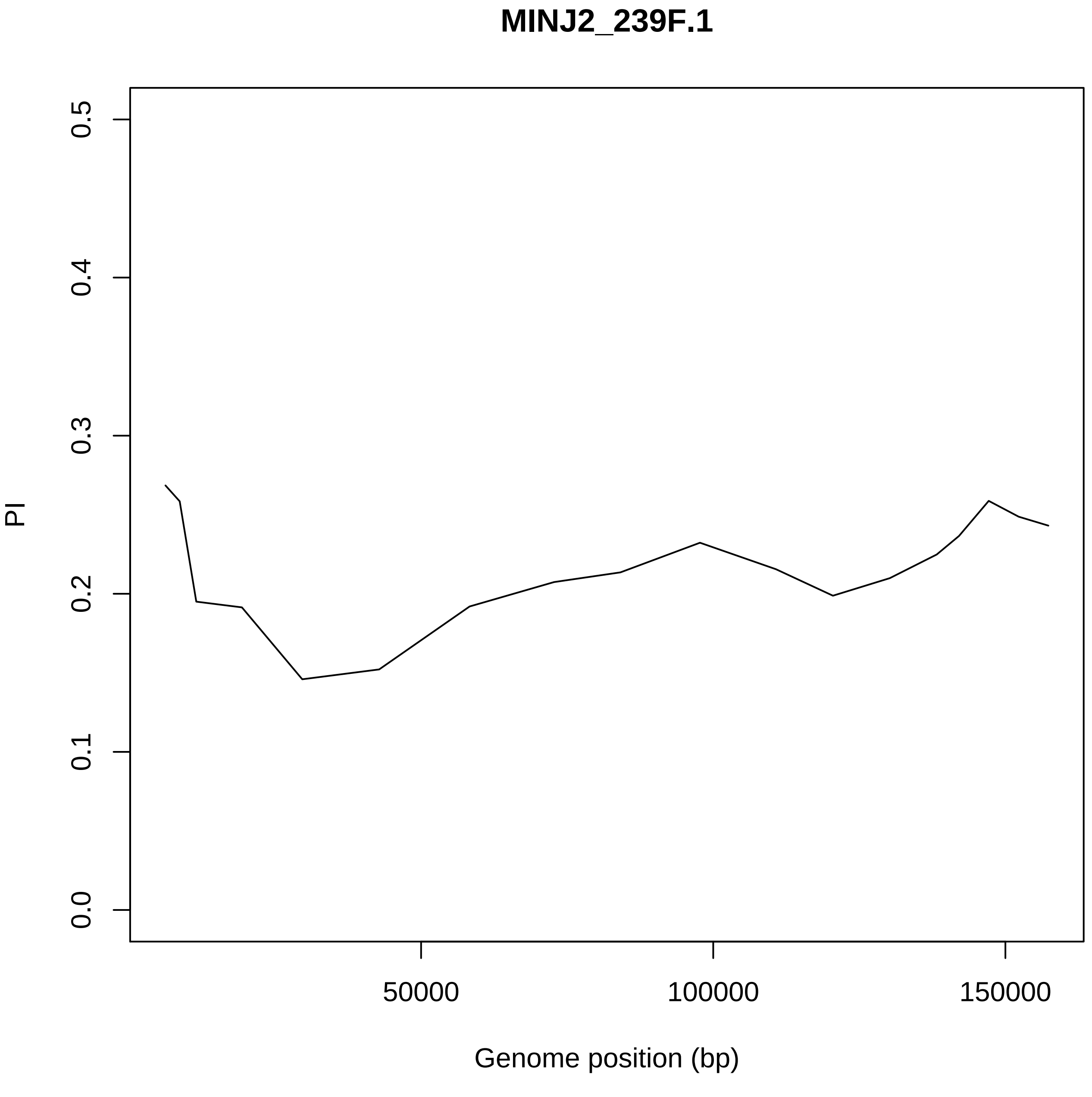

MINJ2_239F.1
PI
0.0
0.1
0.2
0.3
0.4
0.5
50000
100000
150000
Genome position (bp)

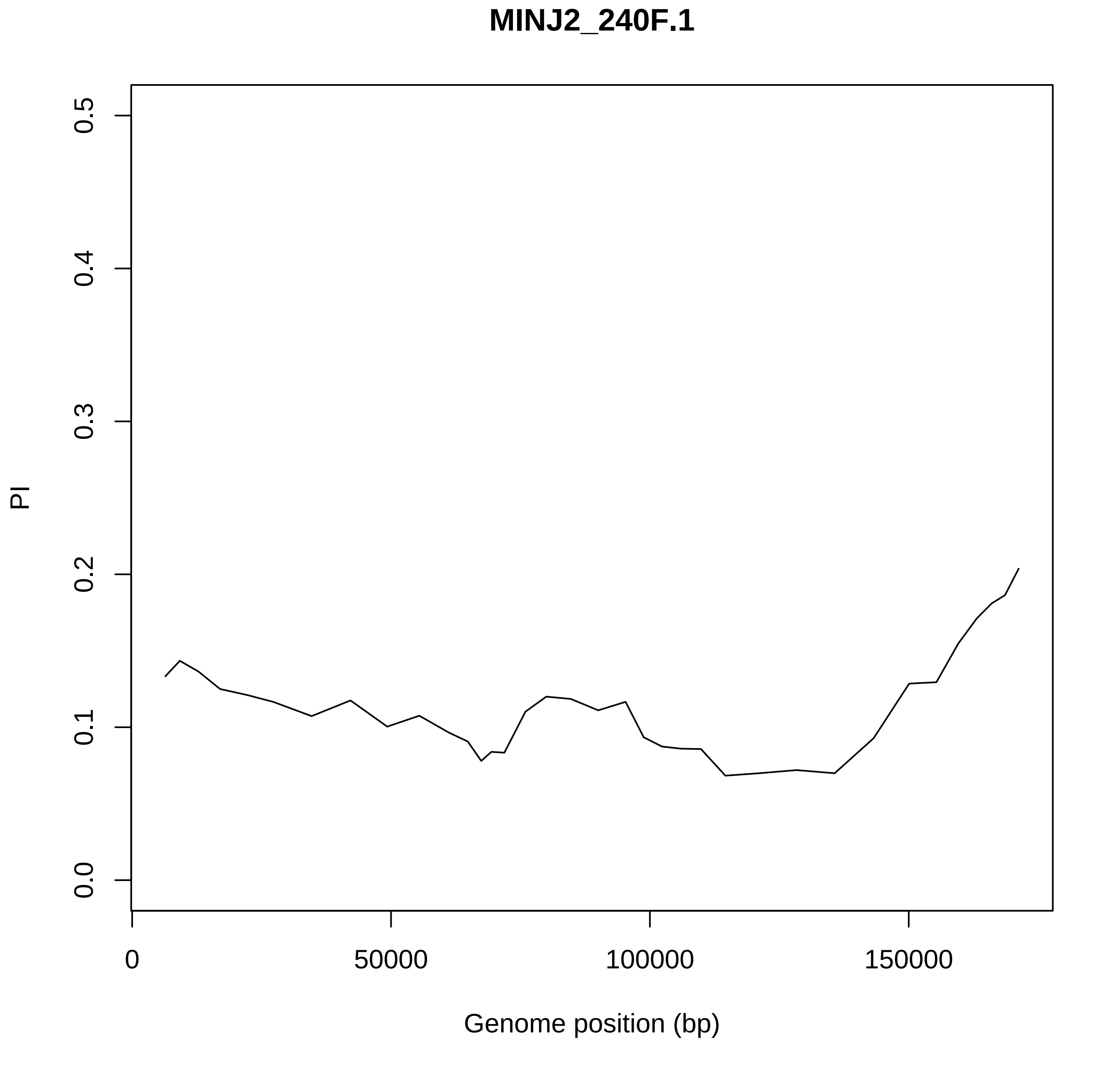
MINJ2_240F.1
PI
0.0
0.1
0.2
0.3
0.4
0.5
0
50000
100000
150000
Genome position (bp)

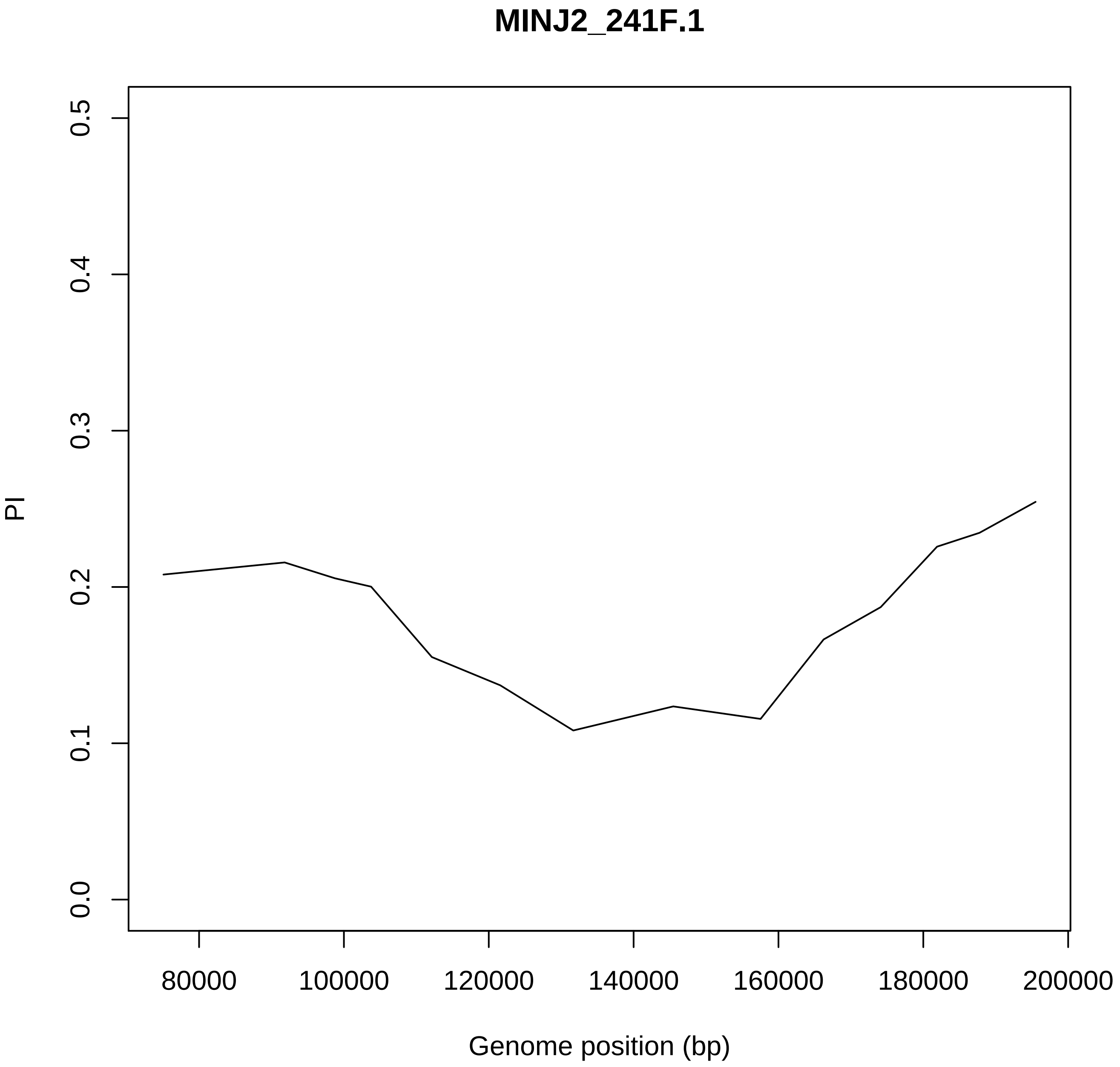
MINJ2_241F.1
PI
0.0
0.1
0.2
0.3
0.4
0.5
80000
100000
120000
140000
160000
180000
200000
Genome position (bp)

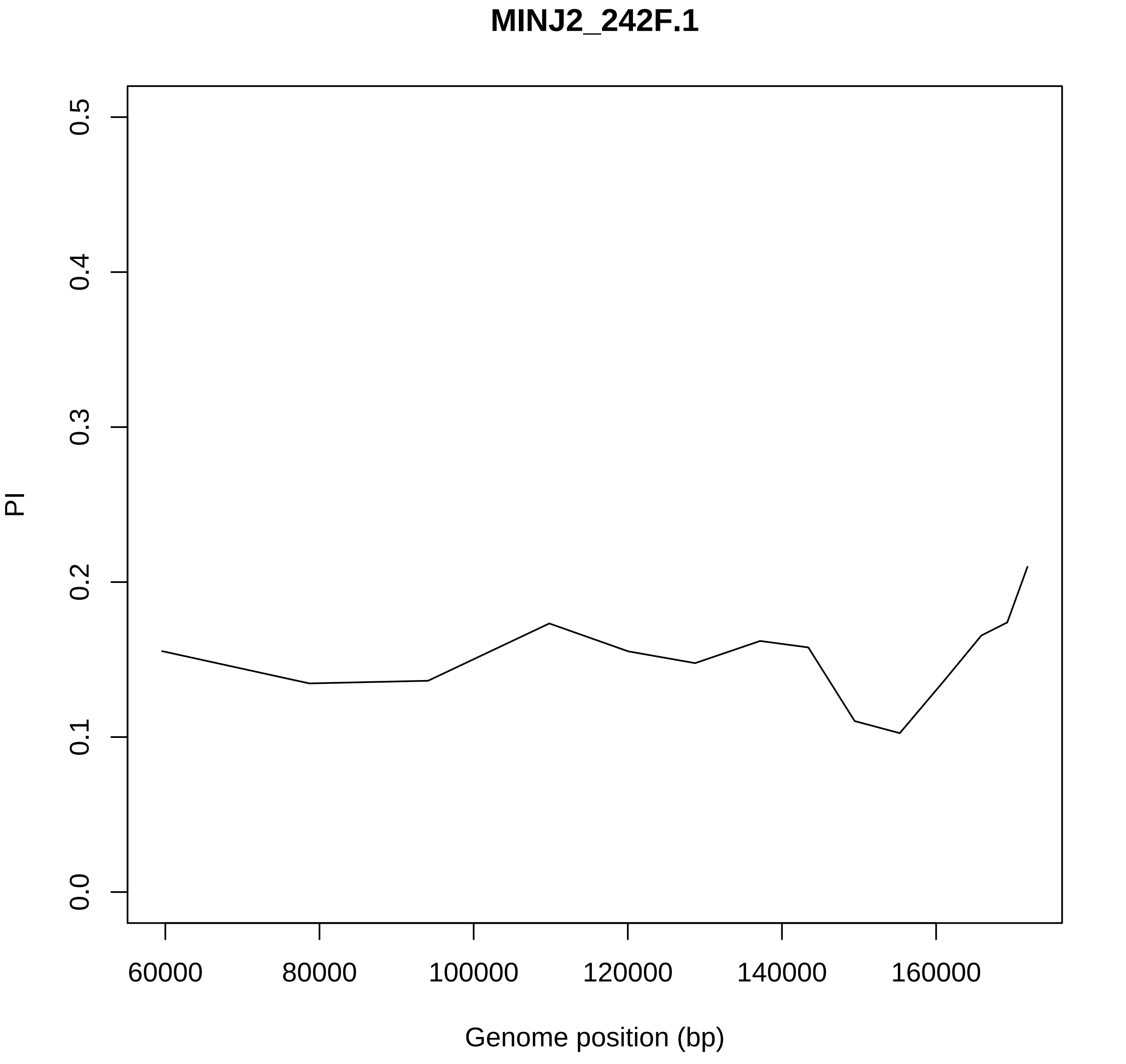

MINJ2_242F.1
PI
0.0
0.1
0.2
0.3
0.4
0.5
60000
80000
100000
120000
140000
160000
Genome position (bp)

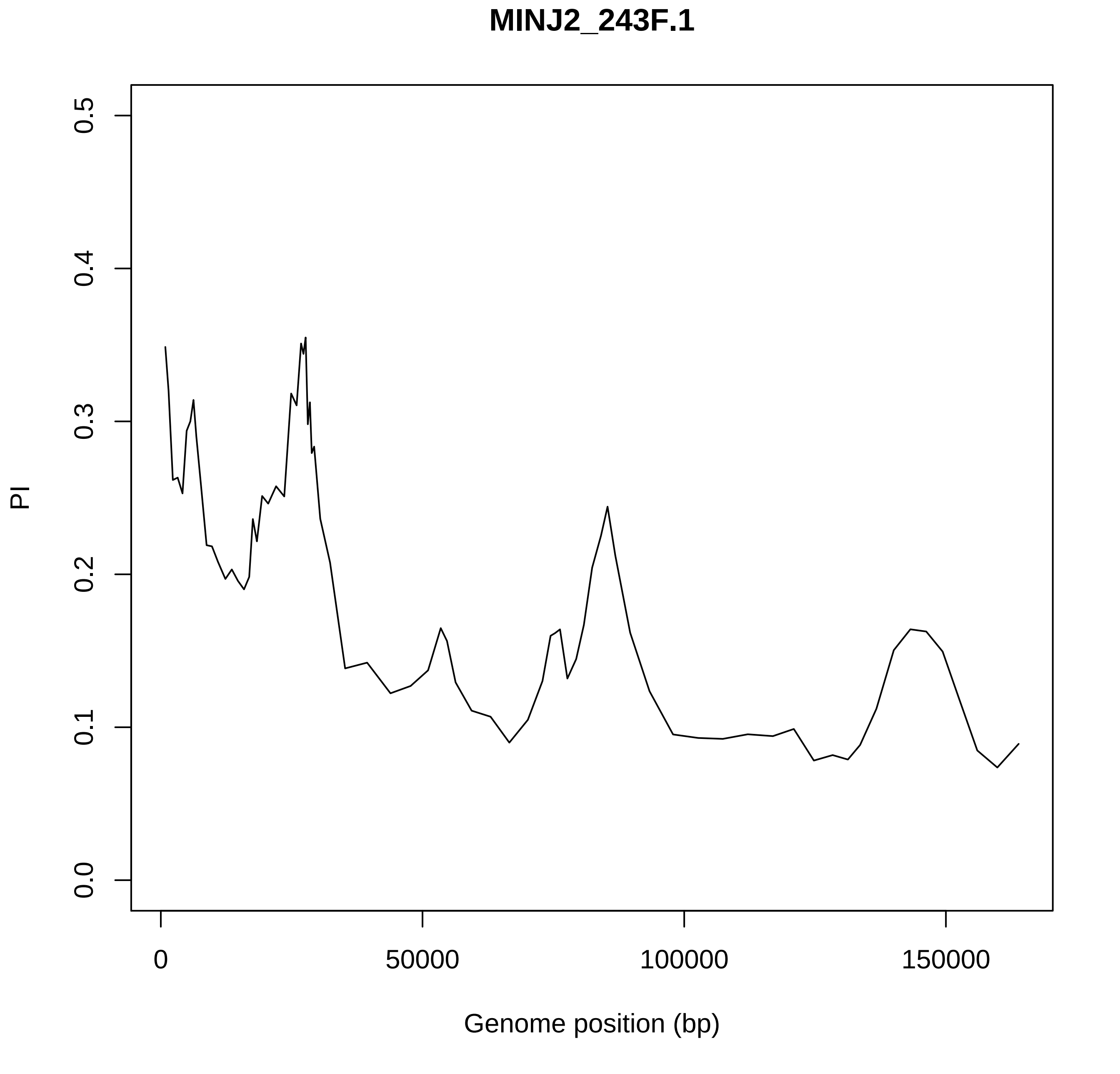
MINJ2_243F.1
PI
0.0
0.1
0.2
0.3
0.4
0.5
0
50000
100000
150000
Genome position (bp)

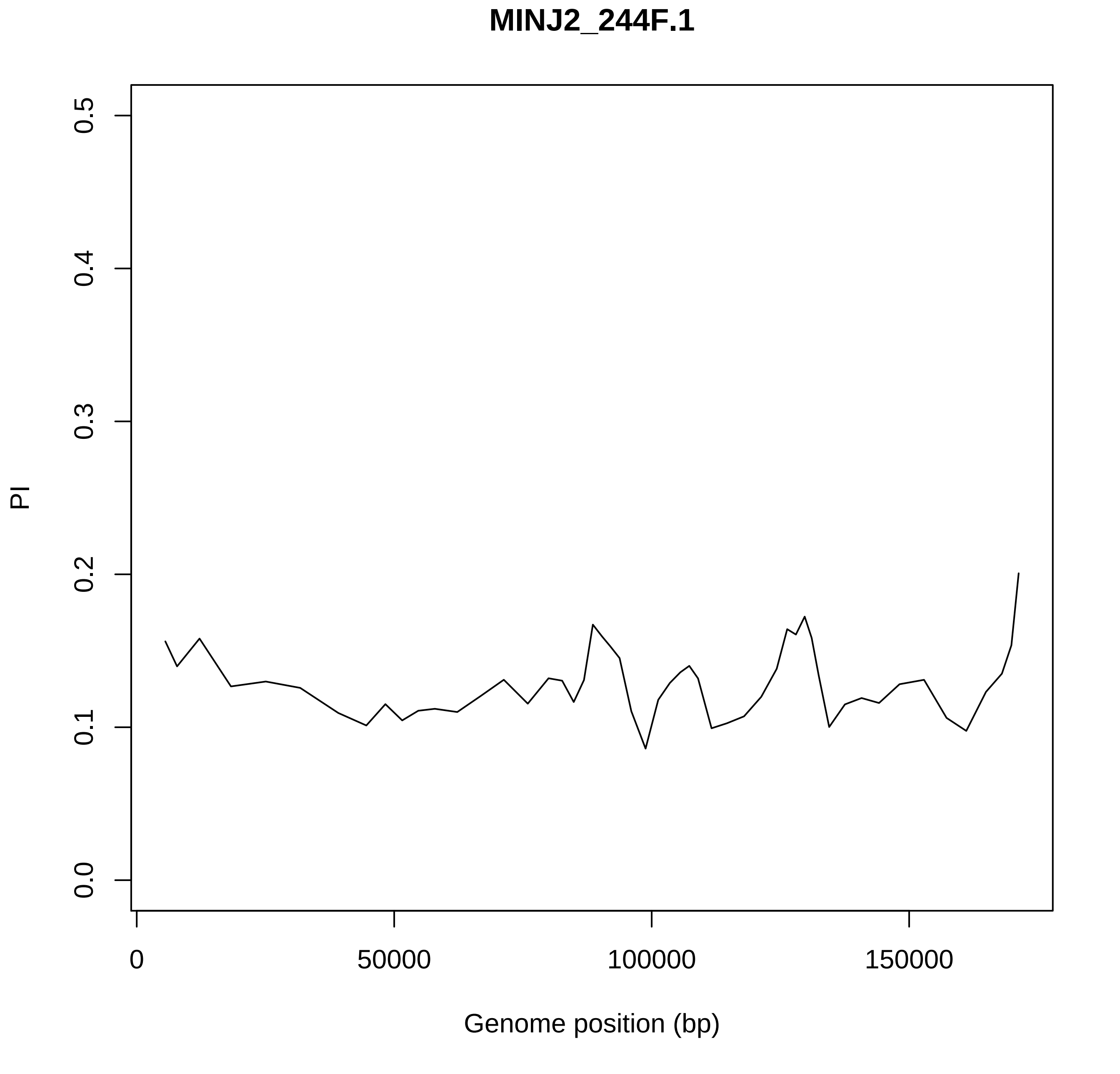
MINJ2_244F.1
PI
0.0
0.1
0.2
0.3
0.4
0.5
0
50000
100000
150000
Genome position (bp)

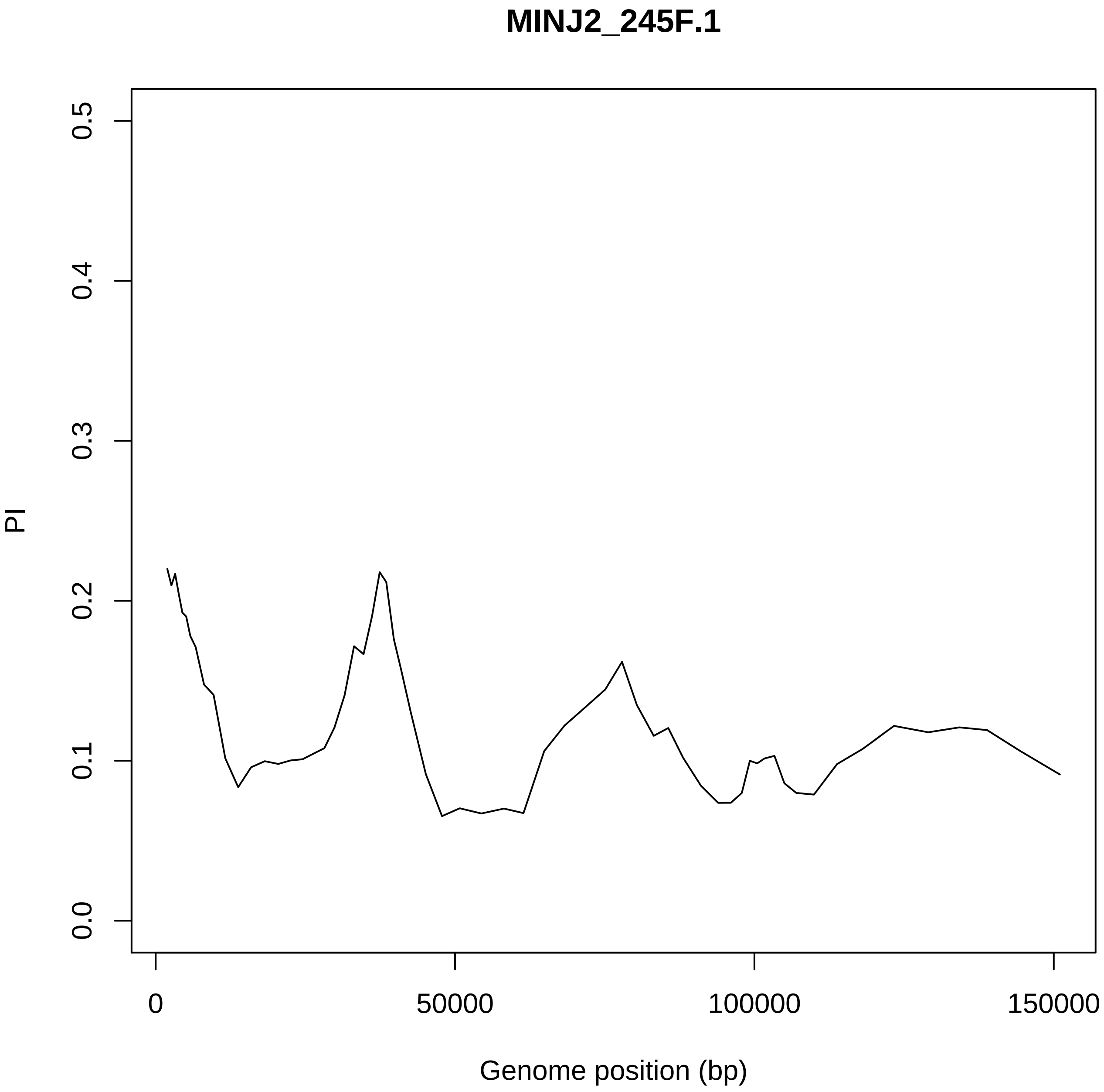
MINJ2_245F.1
PI
0.0
0.1
0.2
0.3
0.4
0.5
0
50000
100000
150000
Genome position (bp)

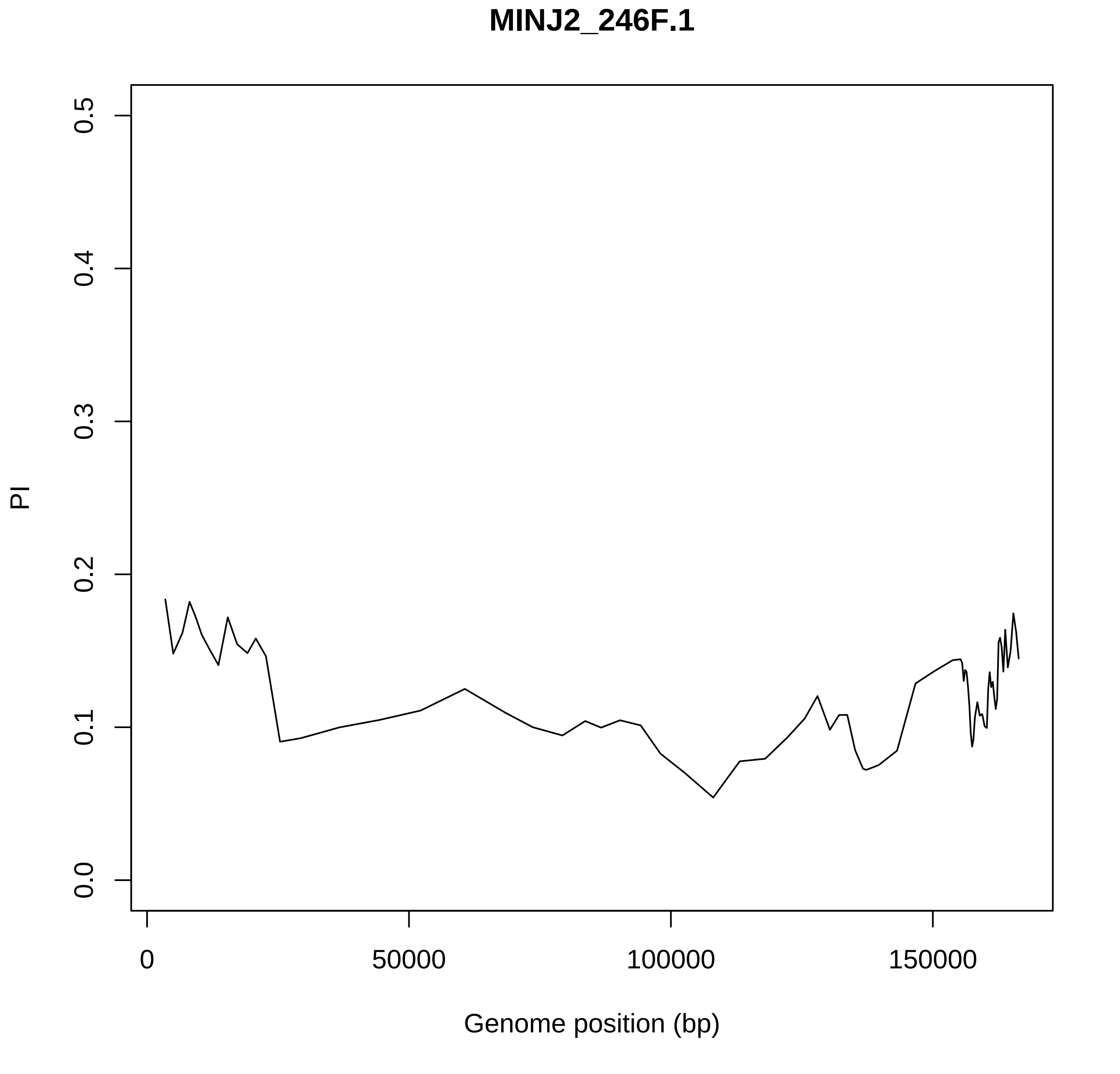
MINJ2_246F.1
PI
0.0
0.1
0.2
0.3
0.4
0.5
0
50000
100000
150000
Genome position (bp)

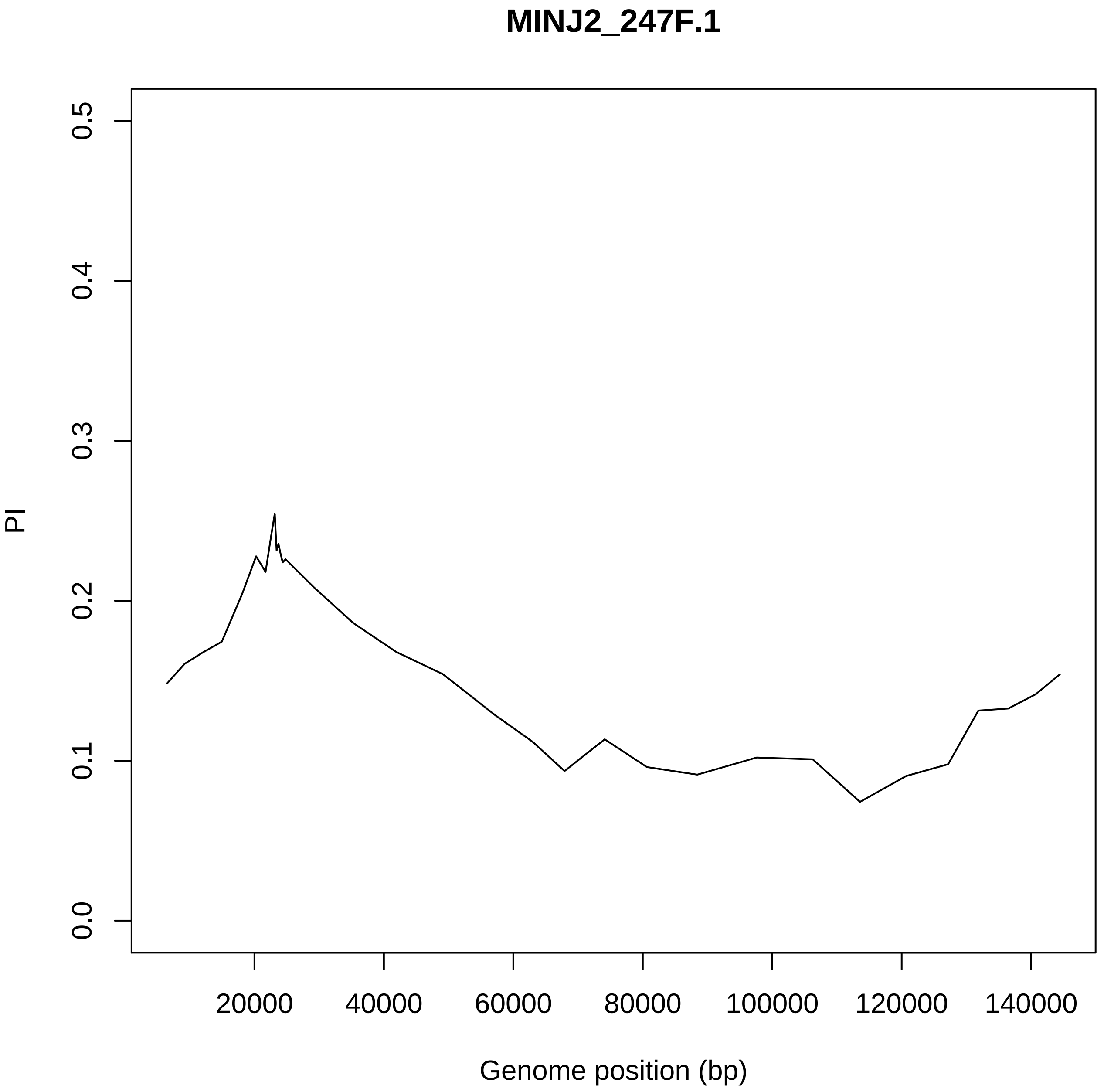
MINJ2_247F.1
PI
0.0
0.1
0.2
0.3
0.4
0.5
20000
40000
60000
80000
100000
120000
140000
Genome position (bp)

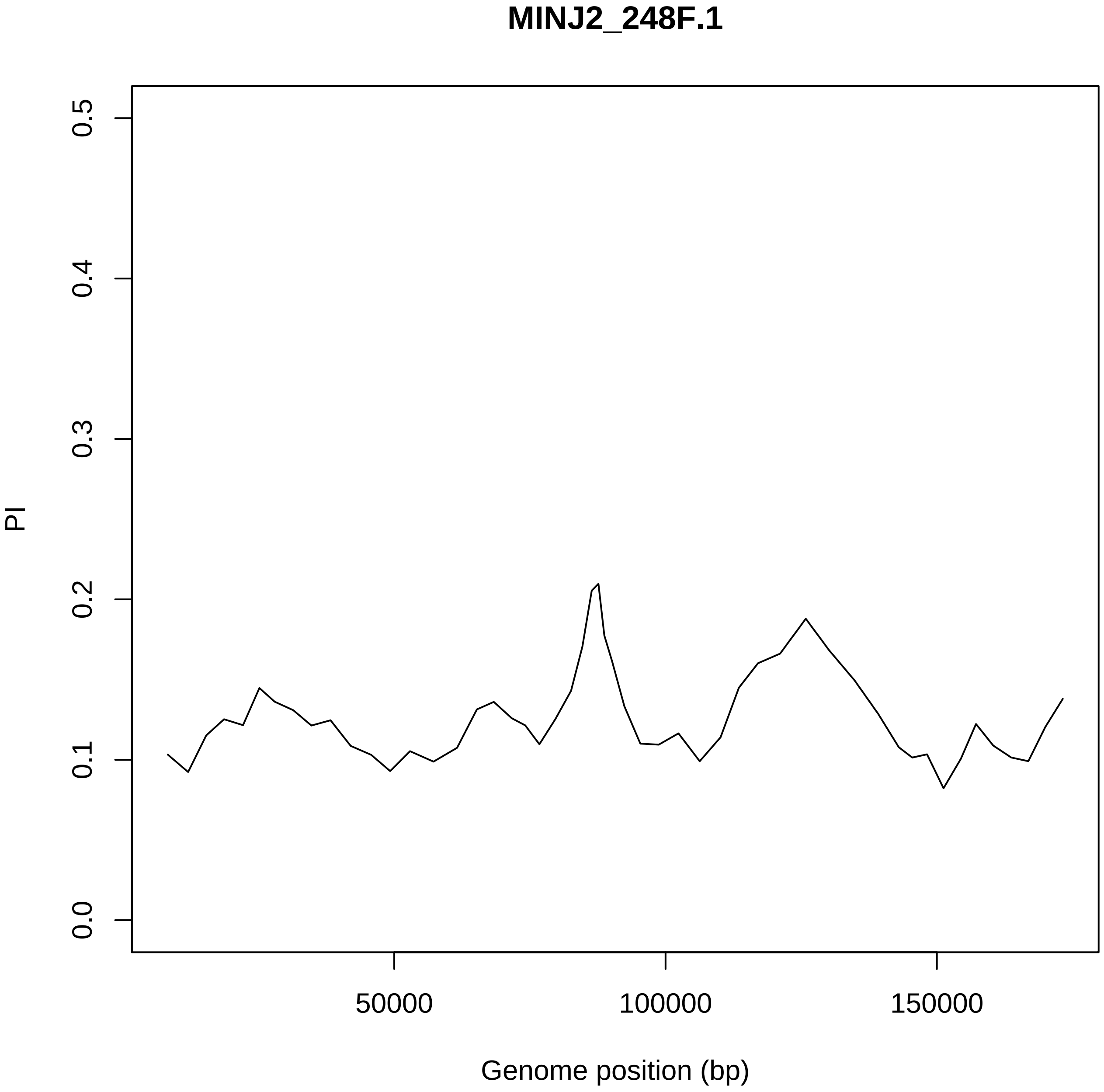
MINJ2_248F.1
PI
0.0
0.1
0.2
0.3
0.4
0.5
50000
100000
150000
Genome position (bp)

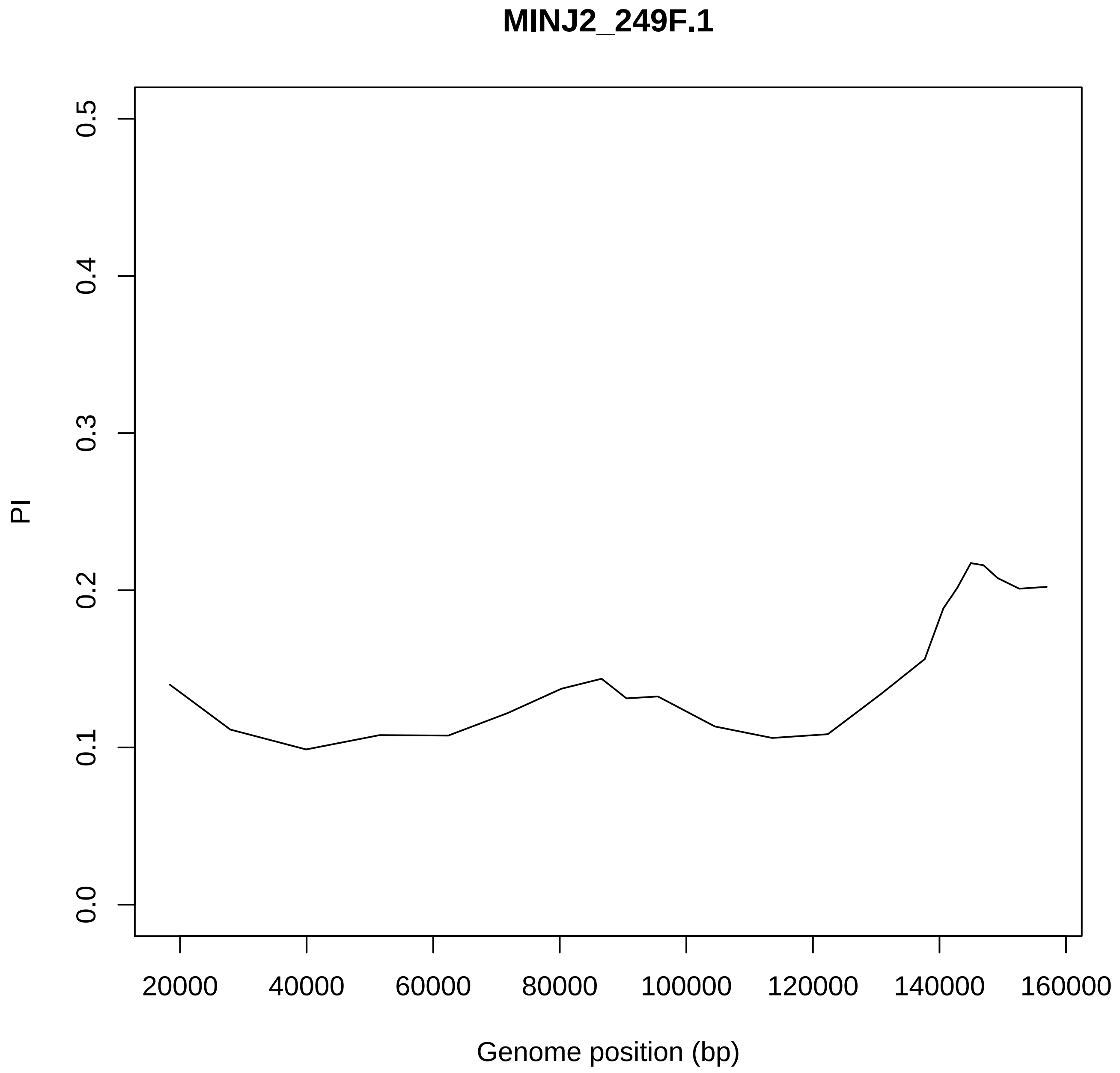

MINJ2_249F.1
PI
0.0
0.1
0.2
0.3
0.4
0.5
20000
40000
60000
80000
100000
120000
140000
160000
Genome position (bp)

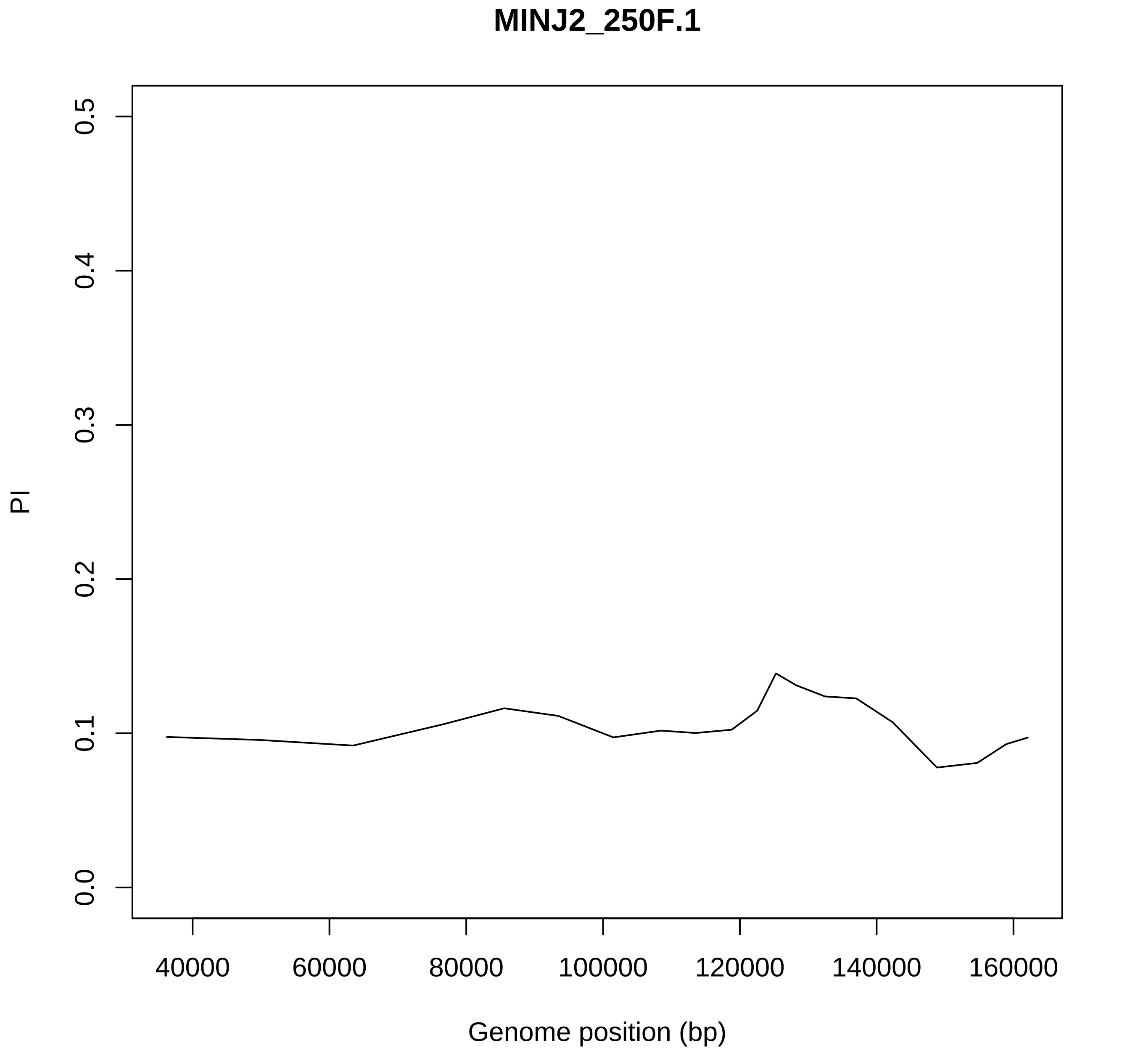

MINJ2_250F.1
PI
0.0
0.1
0.2
0.3
0.4
0.5
40000
60000
80000
100000
120000
140000
160000
Genome position (bp)

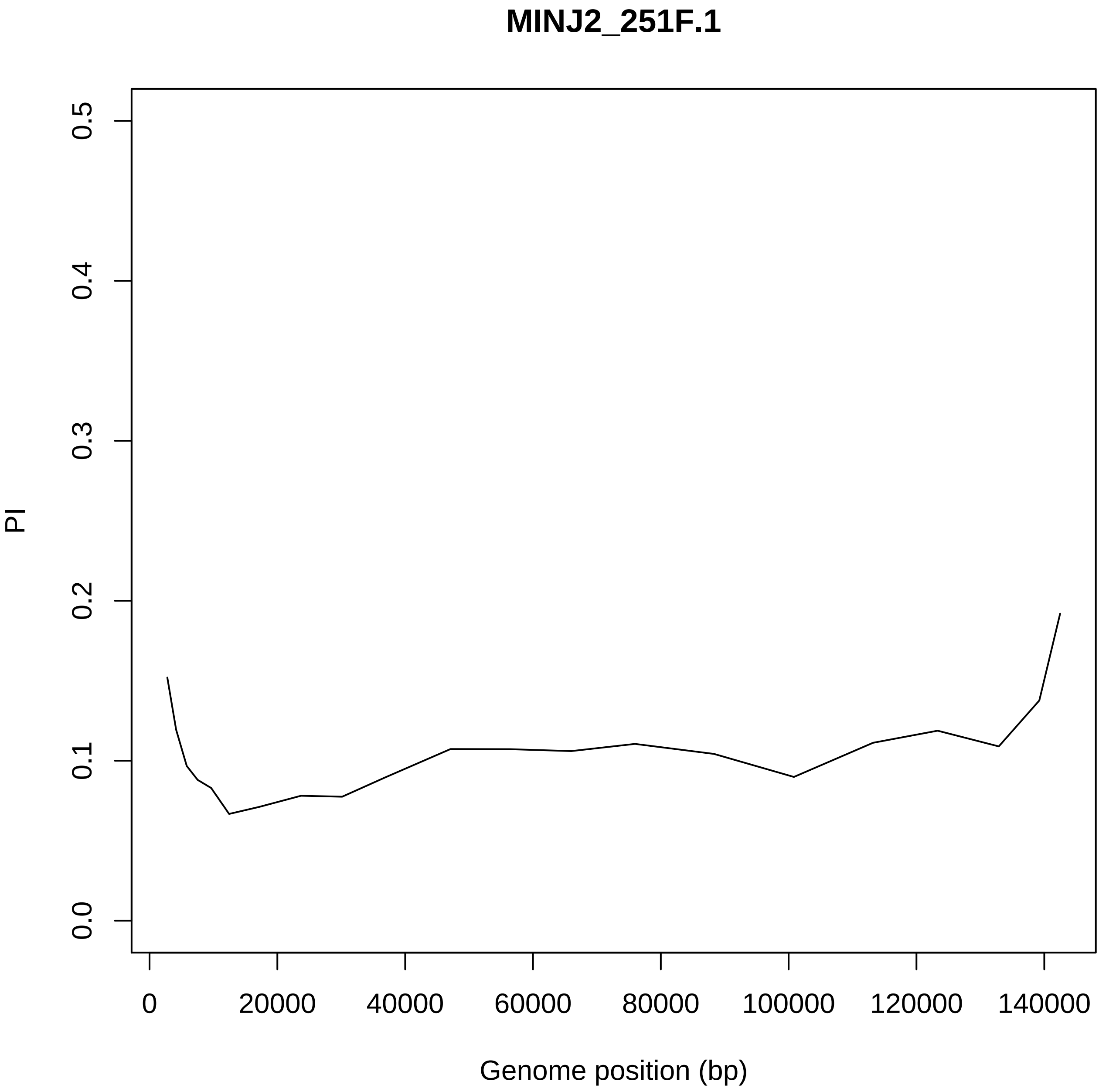

MINJ2_251F.1
PI
0.0
0.1
0.2
0.3
0.4
0.5
0
20000
40000
60000
80000
100000
120000
140000
Genome position (bp)

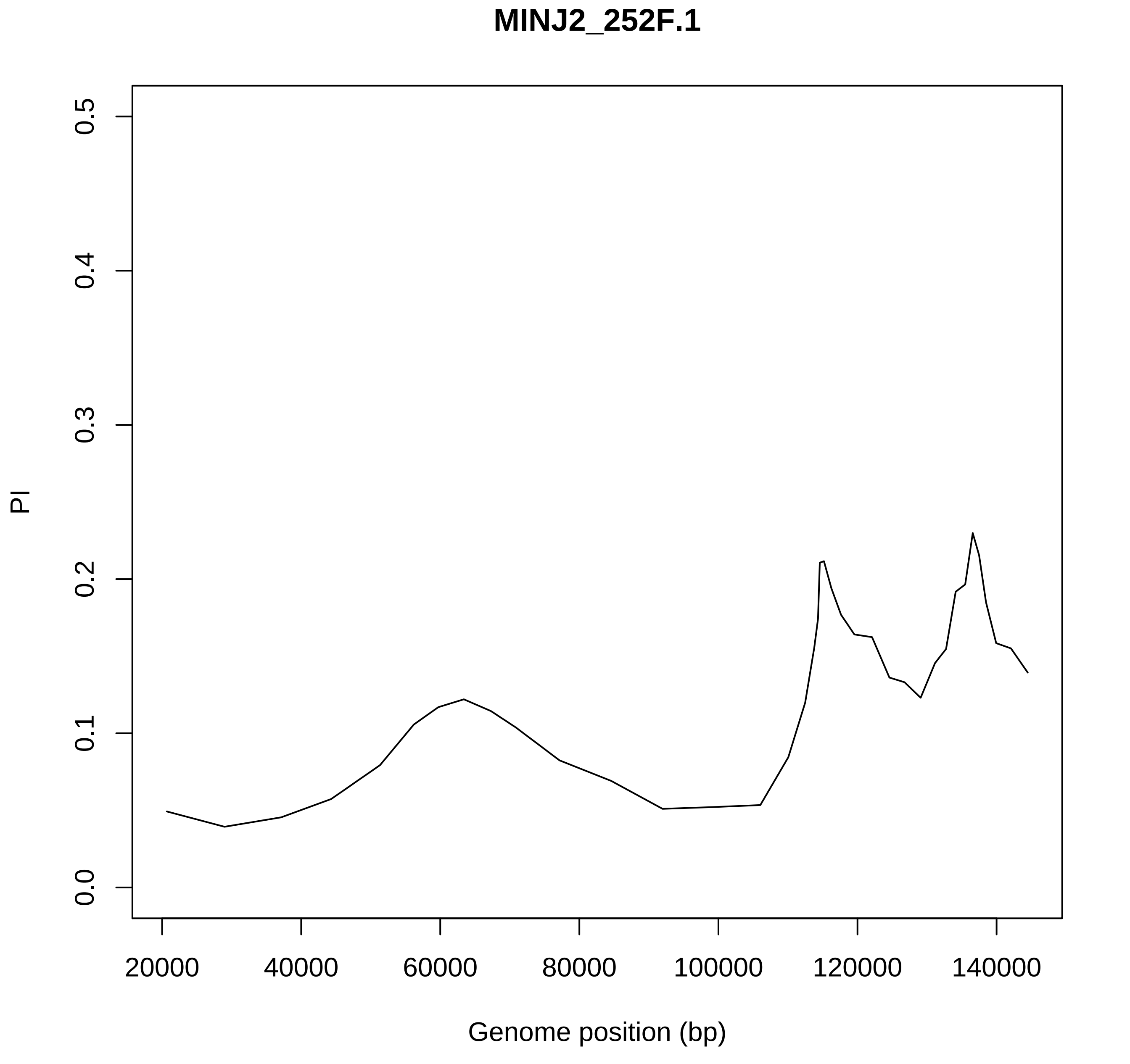
MINJ2_252F.1
PI
0.0
0.1
0.2
0.3
0.4
0.5
20000
40000
60000
80000
100000
120000
140000
Genome position (bp)

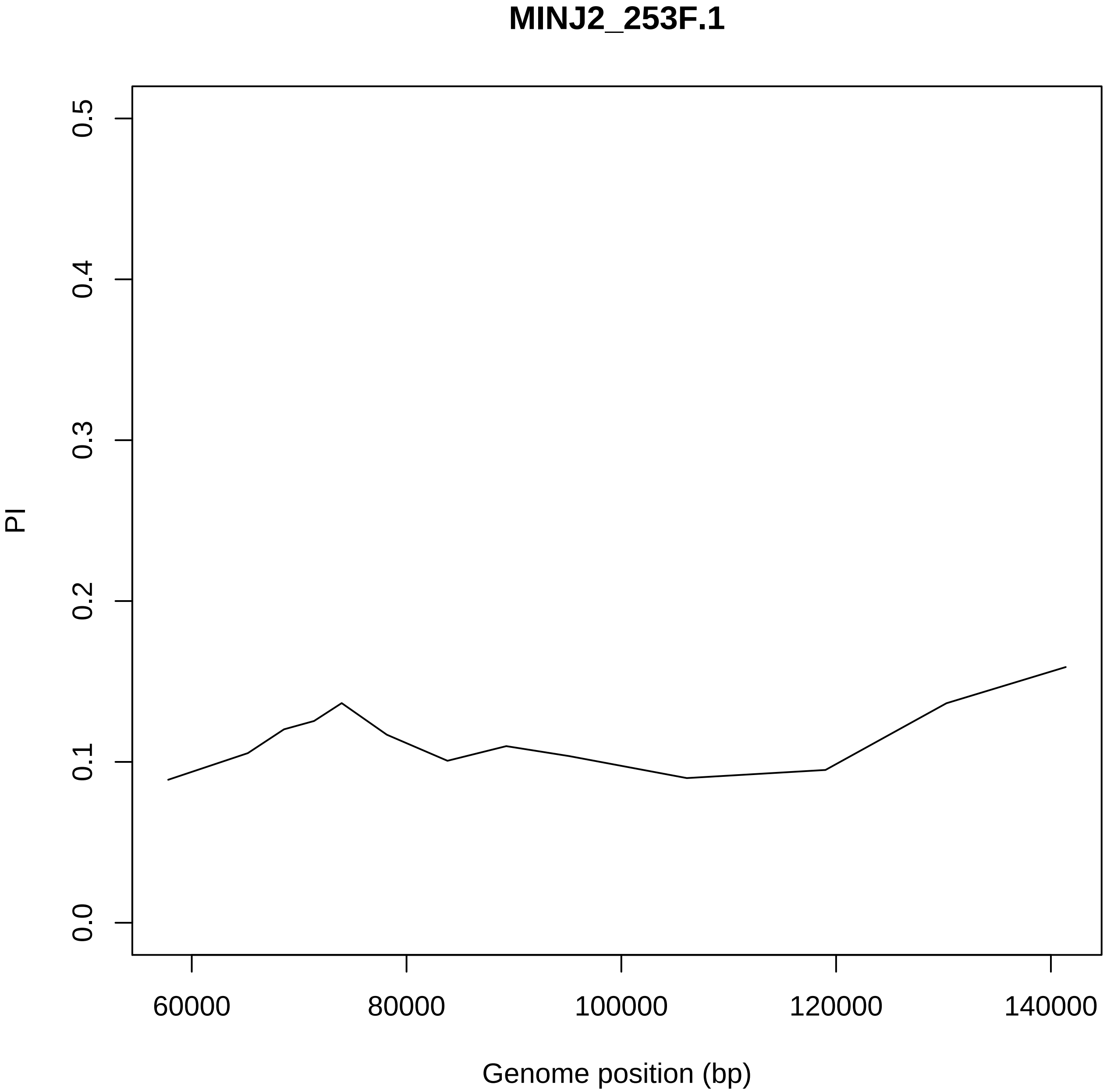
MINJ2_253F.1
PI
0.0
0.1
0.2
0.3
0.4
0.5
60000
80000
100000
120000
140000
Genome position (bp)

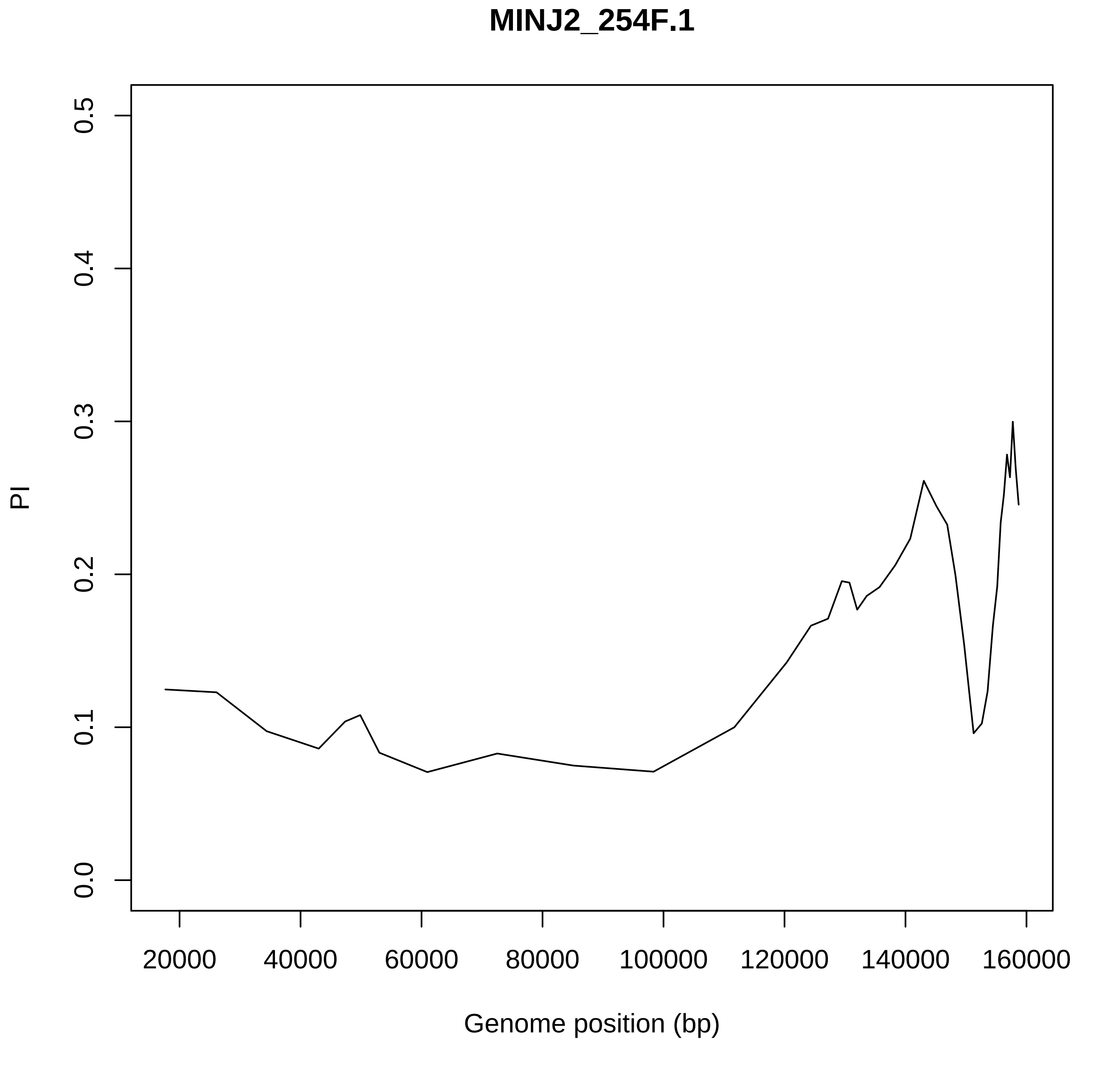

MINJ2_254F.1
PI
0.0
0.1
0.2
0.3
0.4
0.5
20000
40000
60000
80000
100000
120000
140000
160000
Genome position (bp)

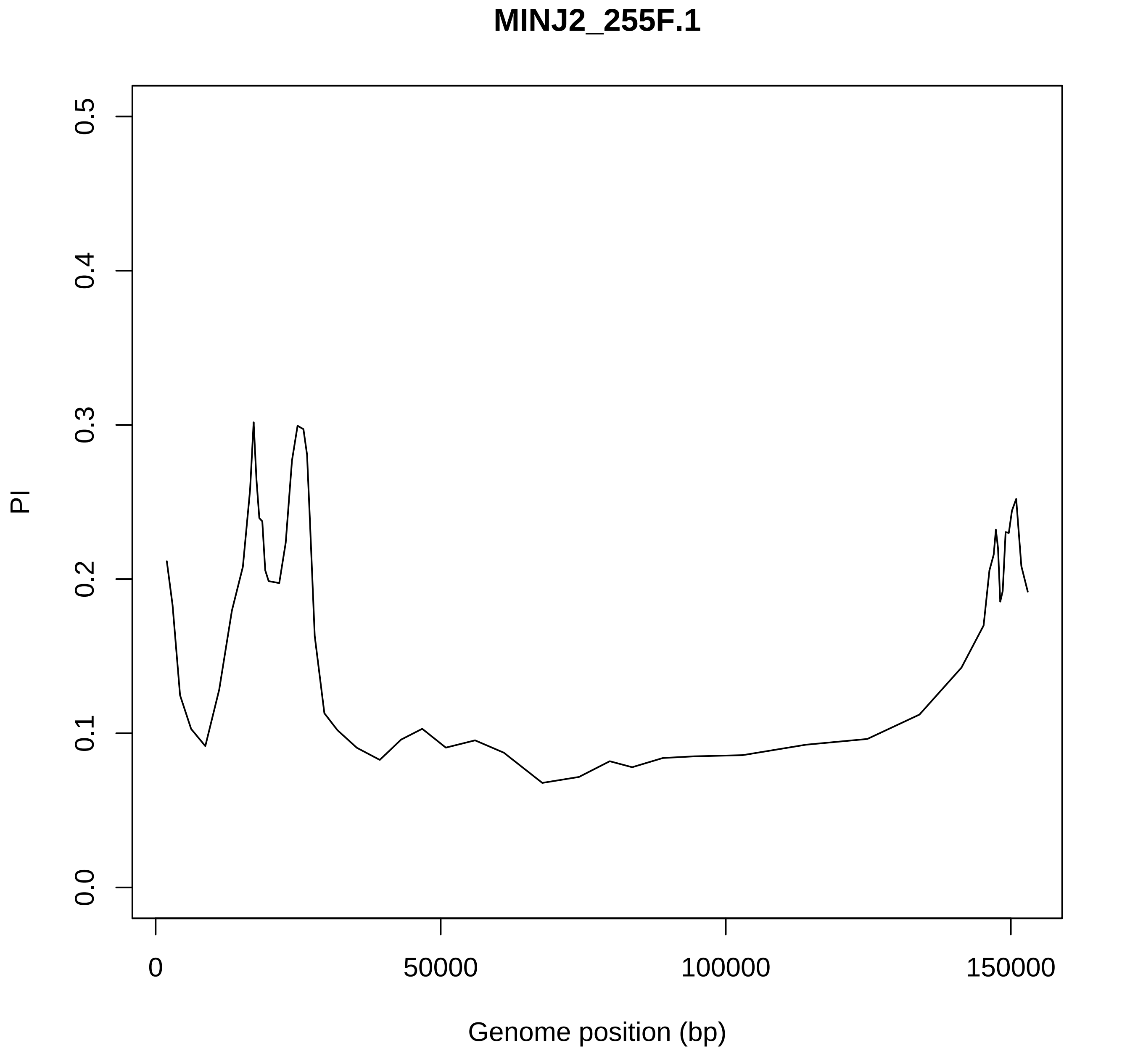
MINJ2_255F.1
PI
0.0
0.1
0.2
0.3
0.4
0.5
0
50000
100000
150000
Genome position (bp)

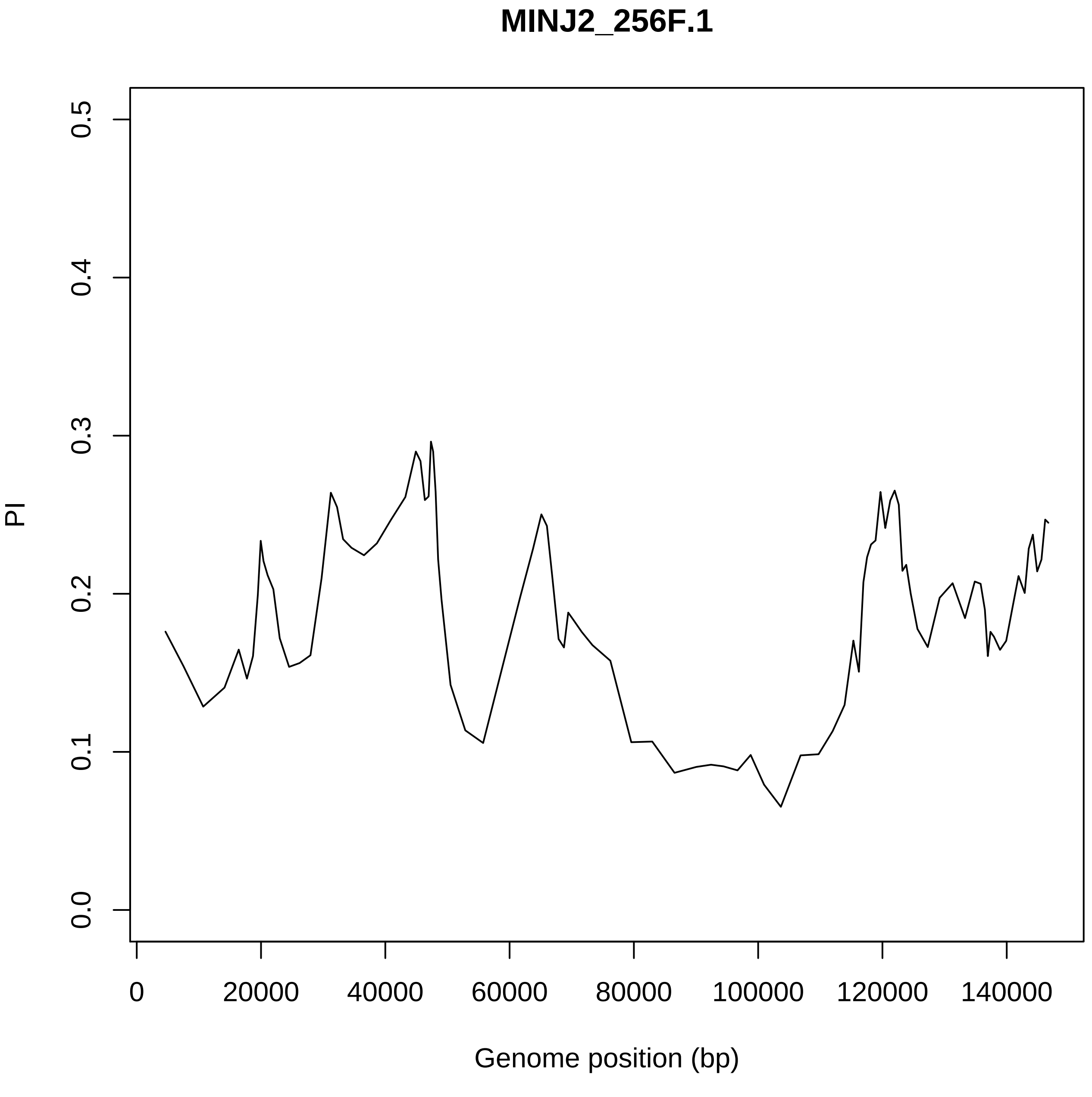
MINJ2_256F.1
PI
0.0
0.1
0.2
0.3
0.4
0.5
0
20000
40000
60000
80000
100000
120000
140000
Genome position (bp)

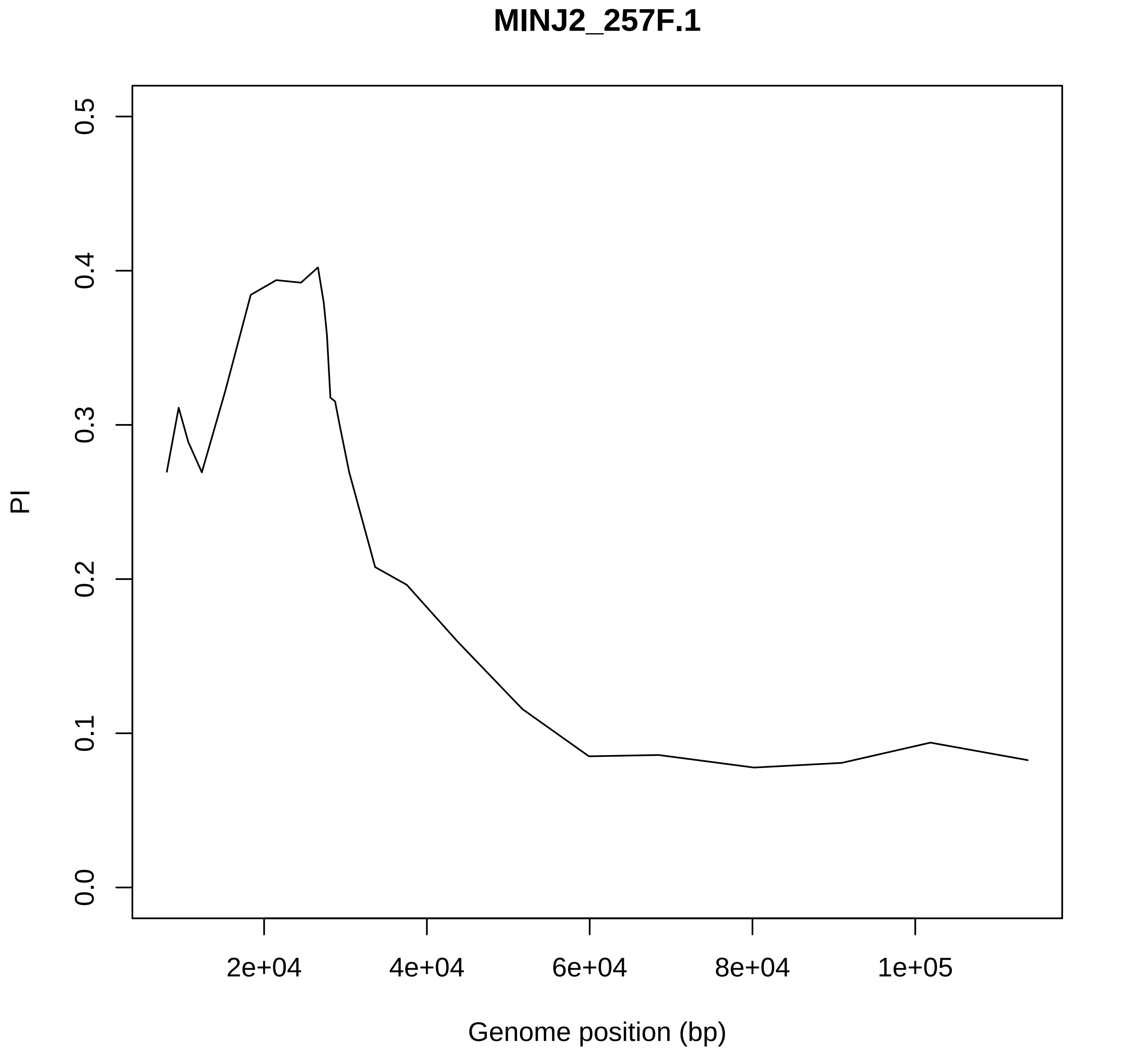

MINJ2_257F.1
PI
0.0
0.1
0.2
0.3
0.4
0.5
2e+04
4e+04
6e+04
8e+04
1e+05
Genome position (bp)

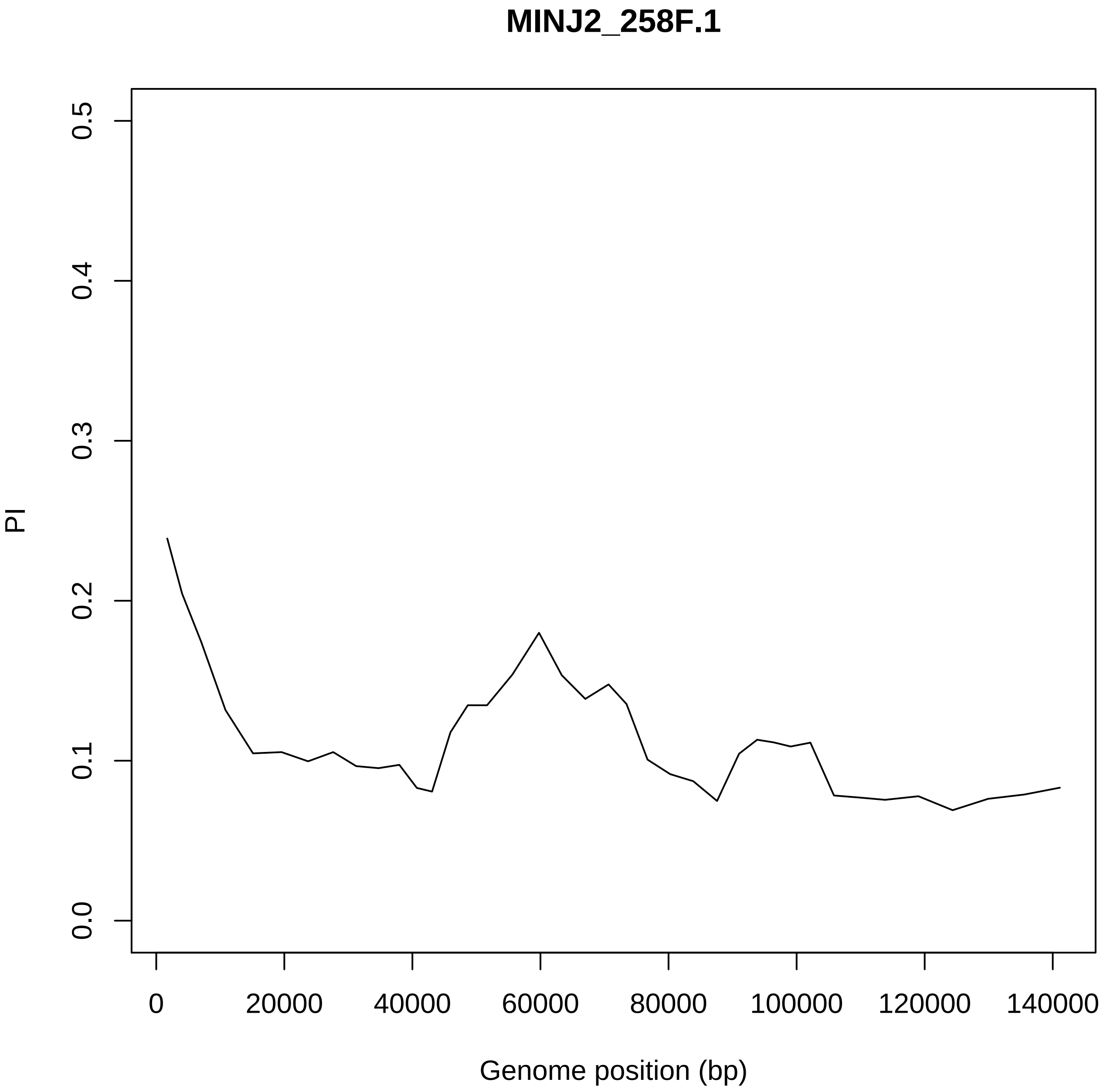
MINJ2_258F.1
PI
0.0
0.1
0.2
0.3
0.4
0.5
0
20000
40000
60000
80000
100000
120000
140000
Genome position (bp)

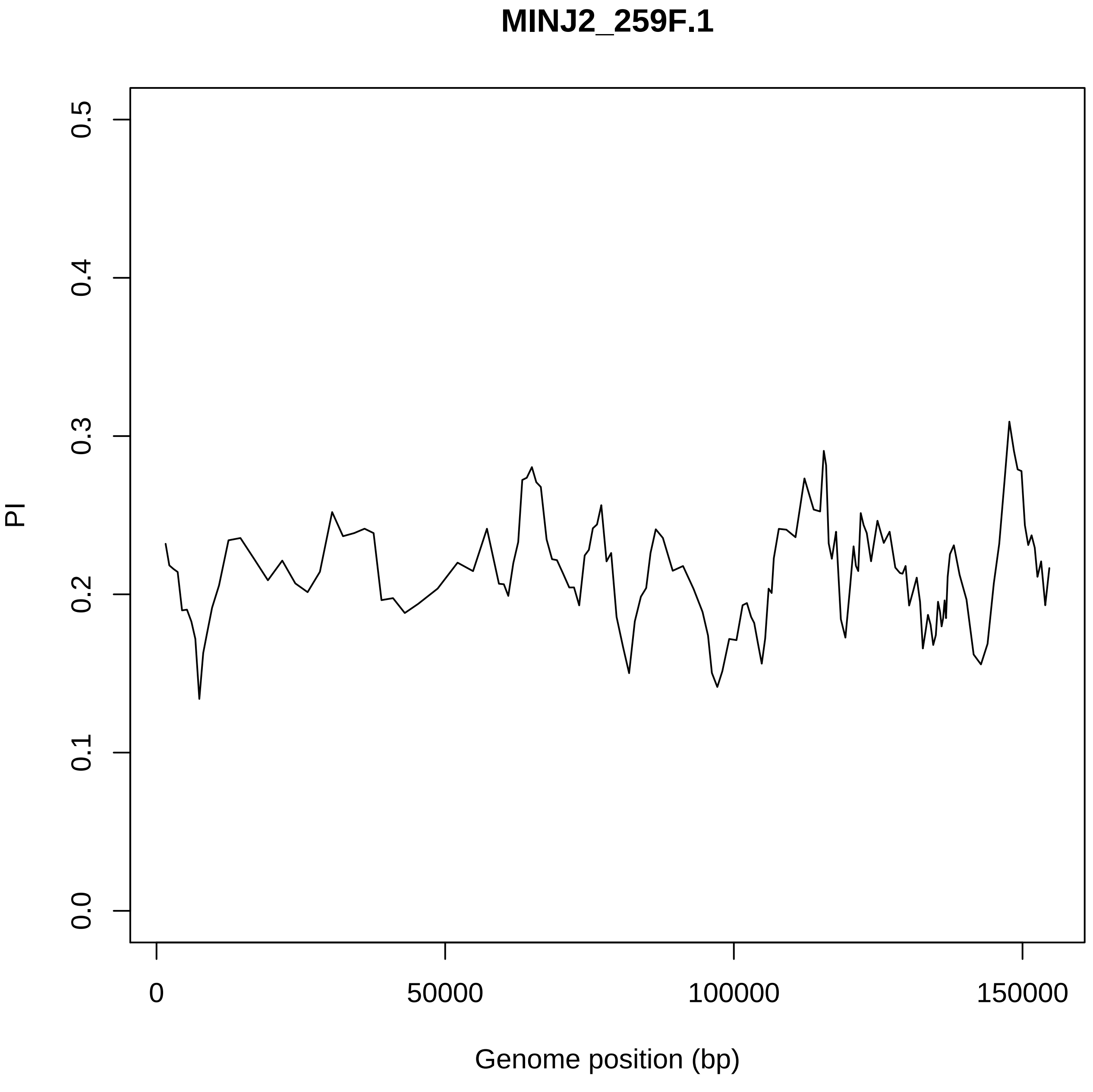
MINJ2_259F.1
PI
0.0
0.1
0.2
0.3
0.4
0.5
0
50000
100000
150000
Genome position (bp)

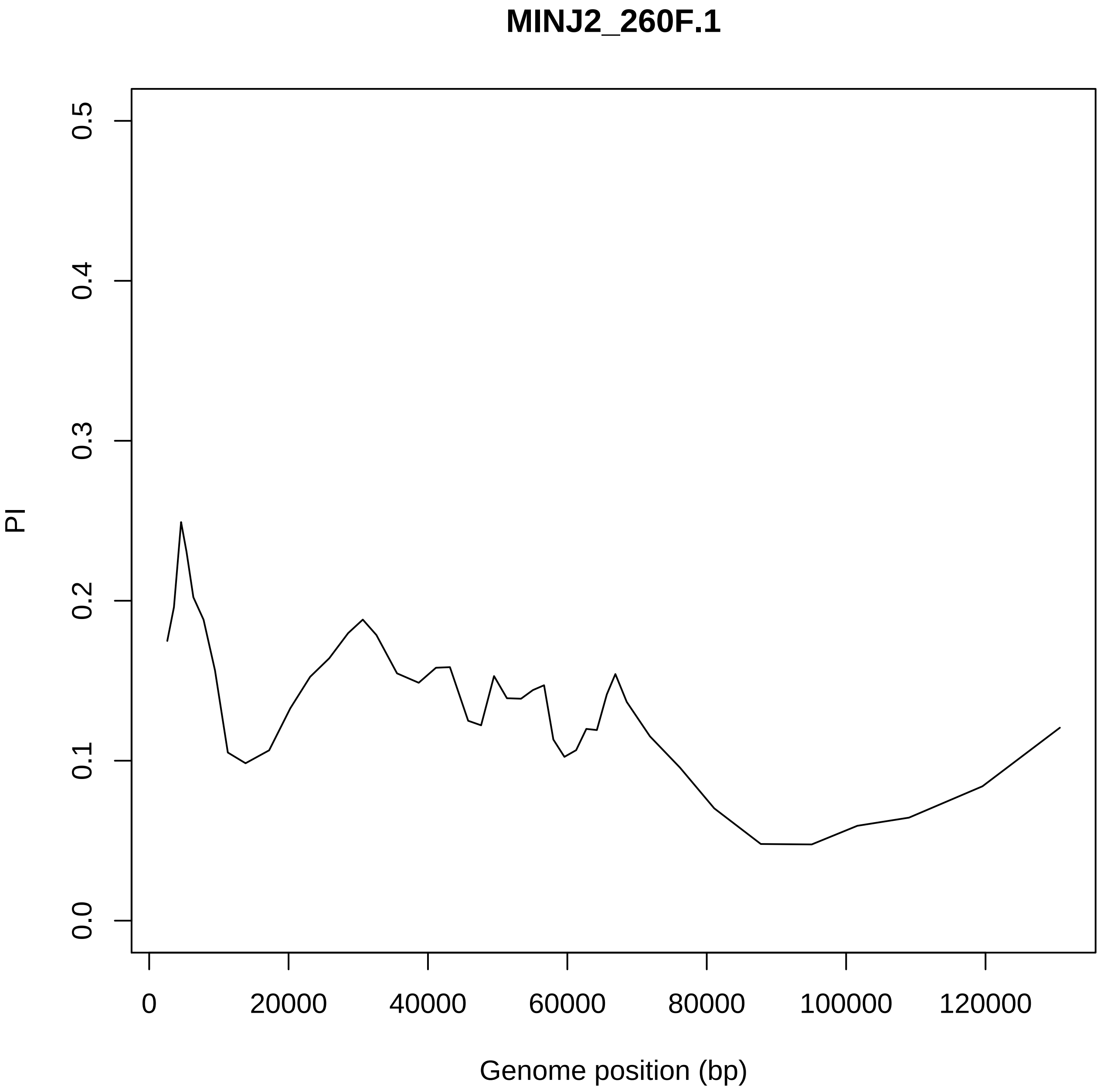

MINJ2_260F.1
PI
0.0
0.1
0.2
0.3
0.4
0.5
0
20000
40000
60000
80000
100000
120000
Genome position (bp)

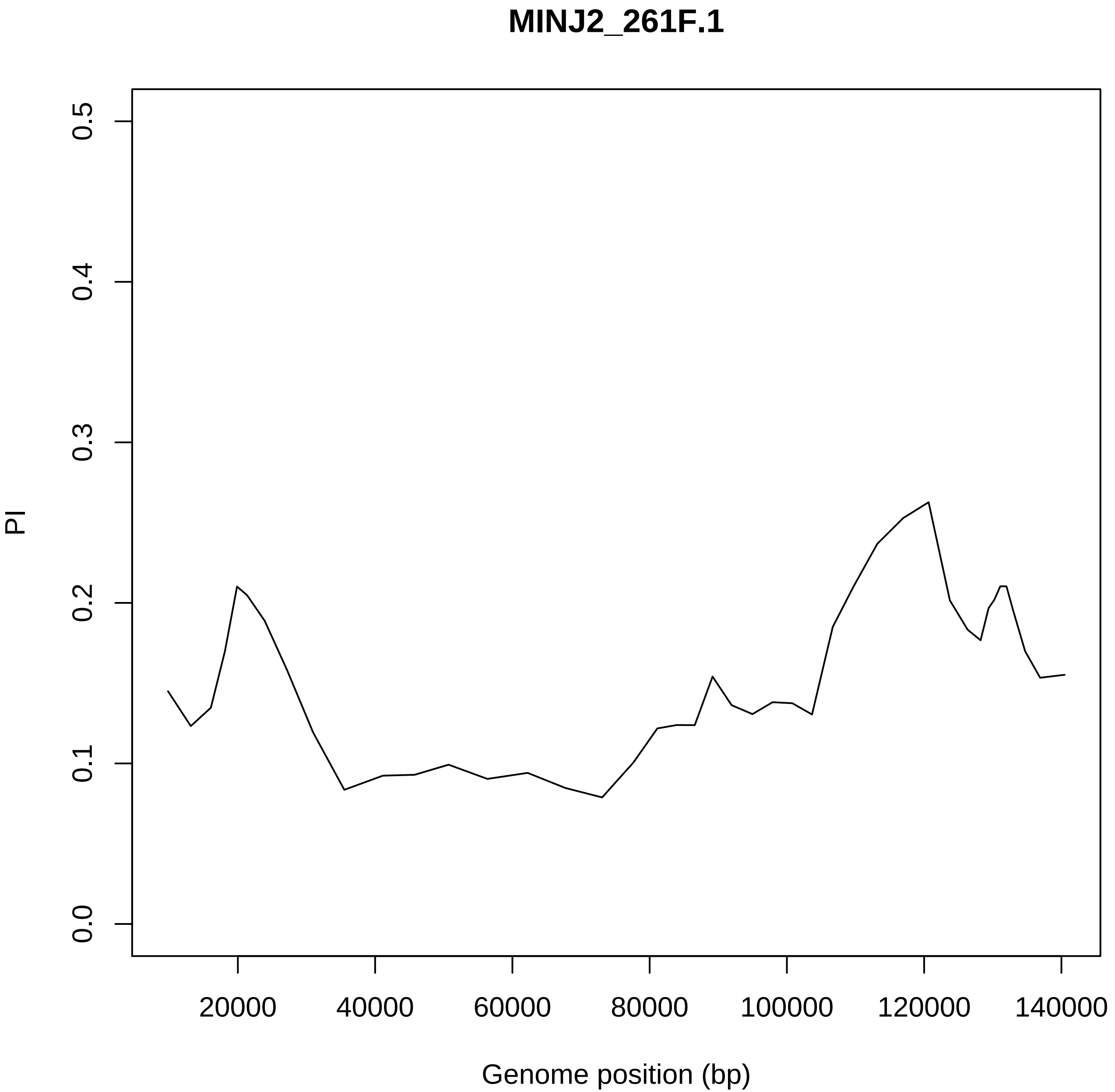

MINJ2_261F.1
PI
0.0
0.1
0.2
0.3
0.4
0.5
20000
40000
60000
80000
100000
120000
140000
Genome position (bp)

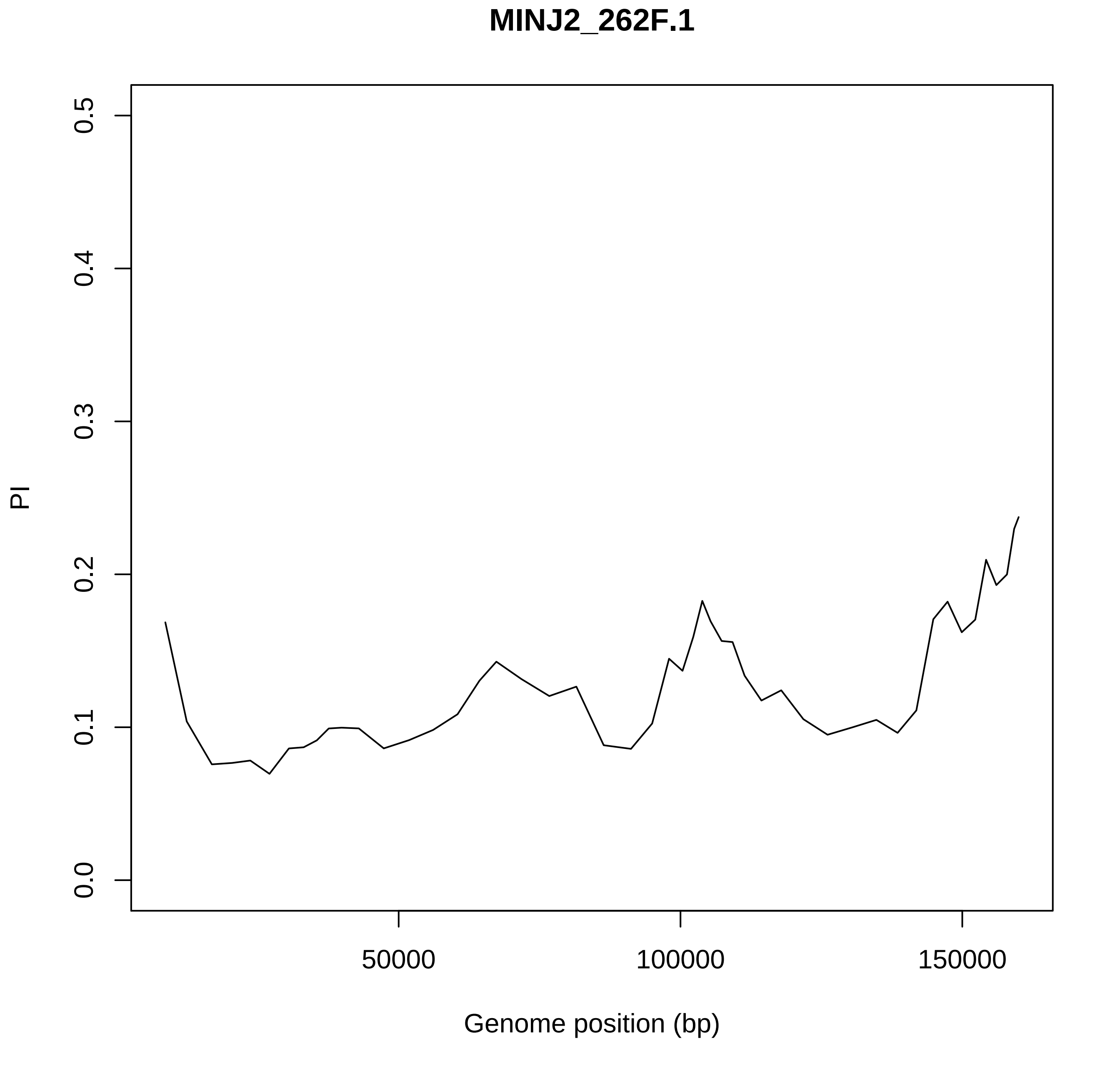
MINJ2_262F.1
PI
0.0
0.1
0.2
0.3
0.4
0.5
50000
100000
150000
Genome position (bp)

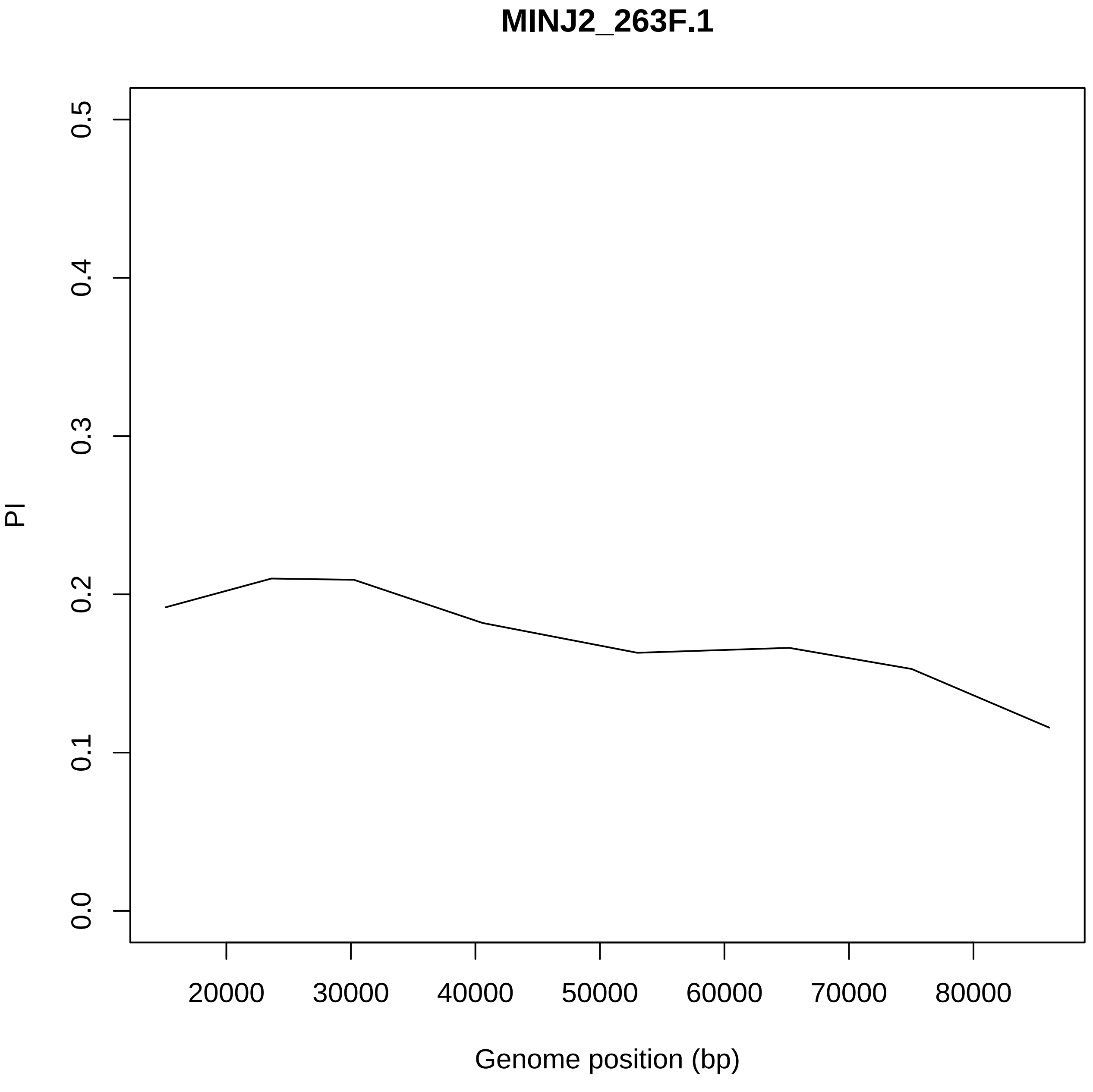
MINJ2_263F.1
PI
0.0
0.1
0.2
0.3
0.4
0.5
20000
30000
40000
50000
60000
70000
80000
Genome position (bp)

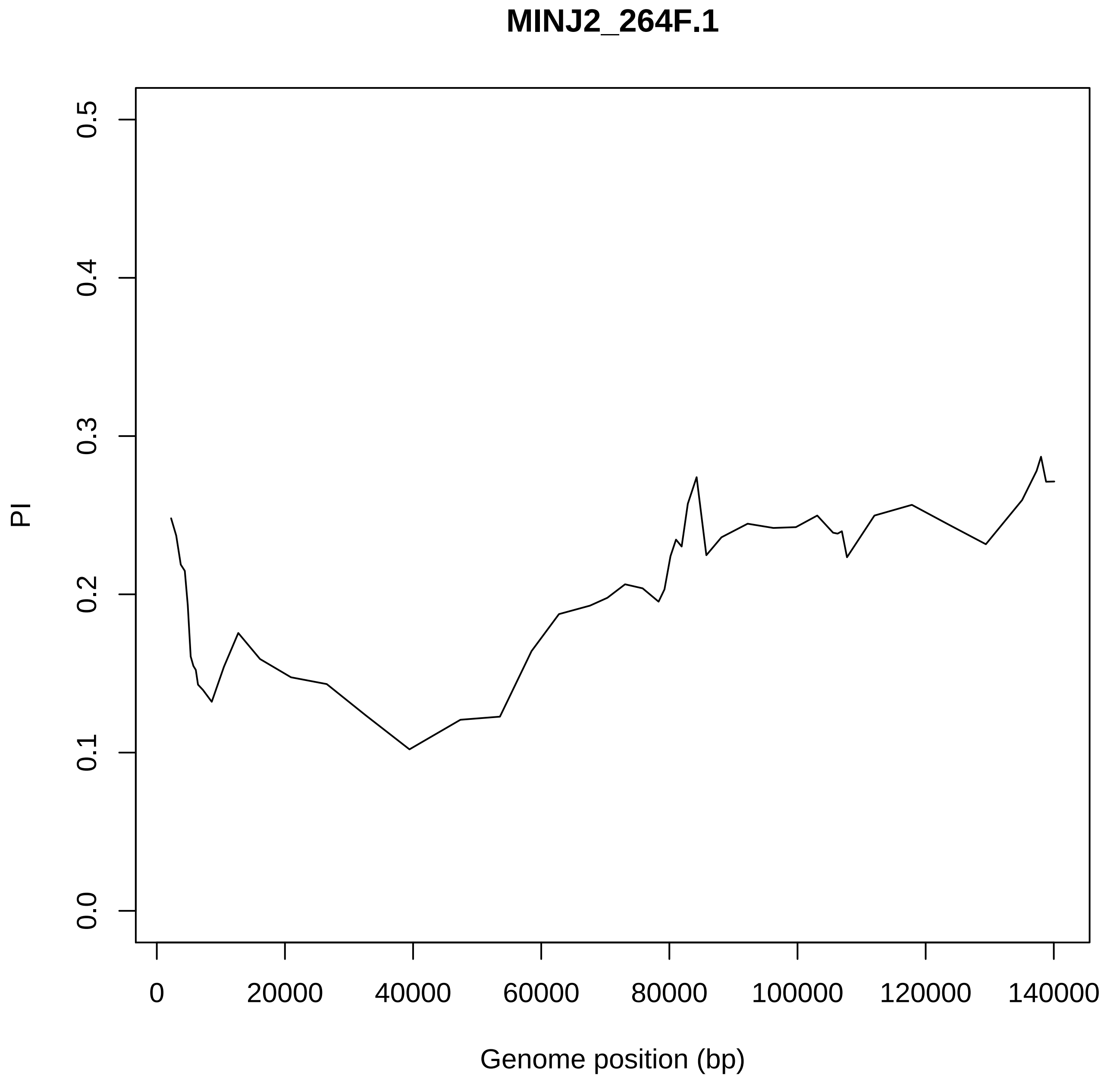

MINJ2_264F.1
PI
0.0
0.1
0.2
0.3
0.4
0.5
Genome position (bp)
0
20000
40000
60000
80000
100000
120000
140000

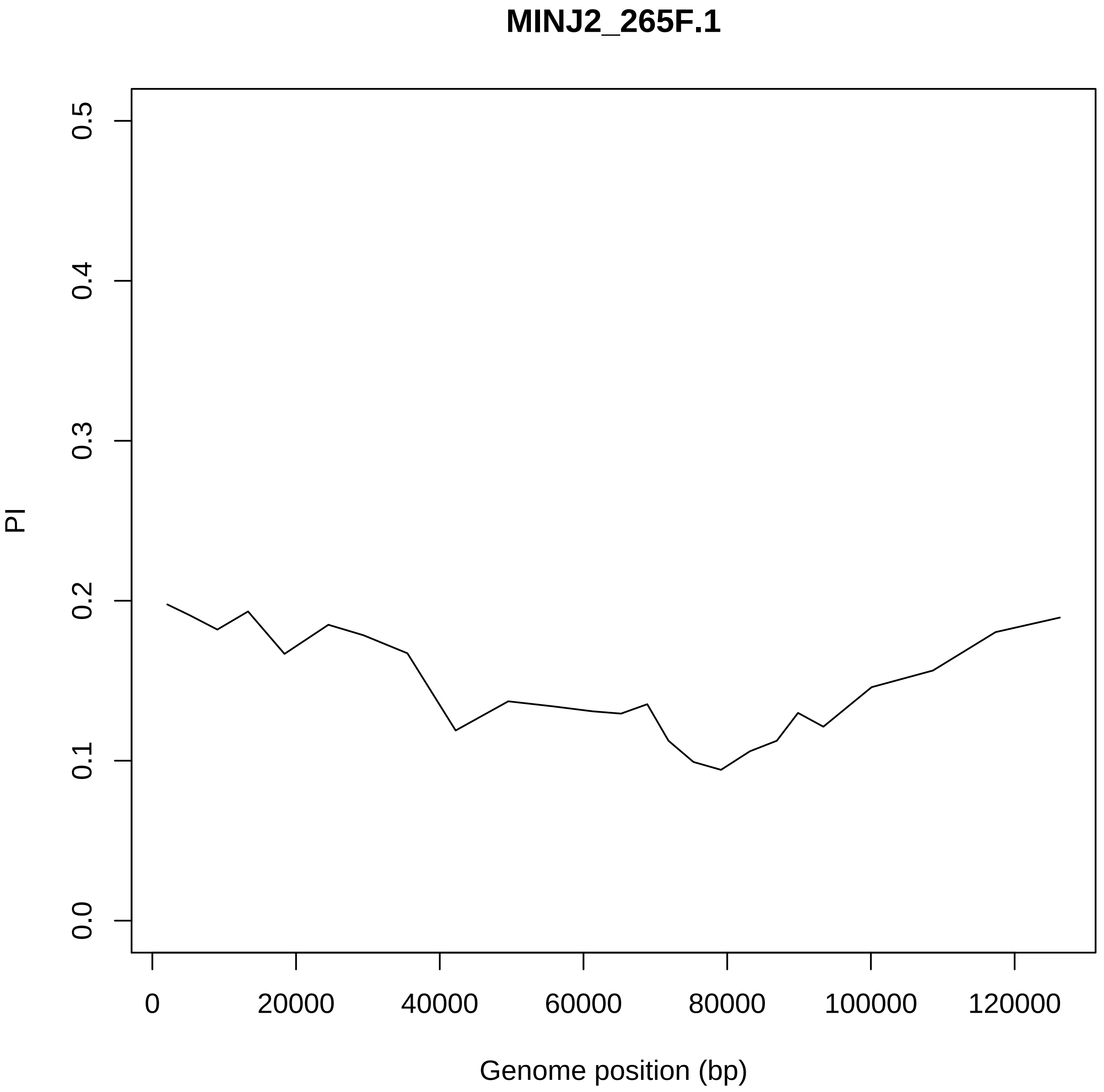
MINJ2_265F.1
PI
0.0
0.1
0.2
0.3
0.4
0.5
0
20000
40000
60000
80000
100000
120000
Genome position (bp)

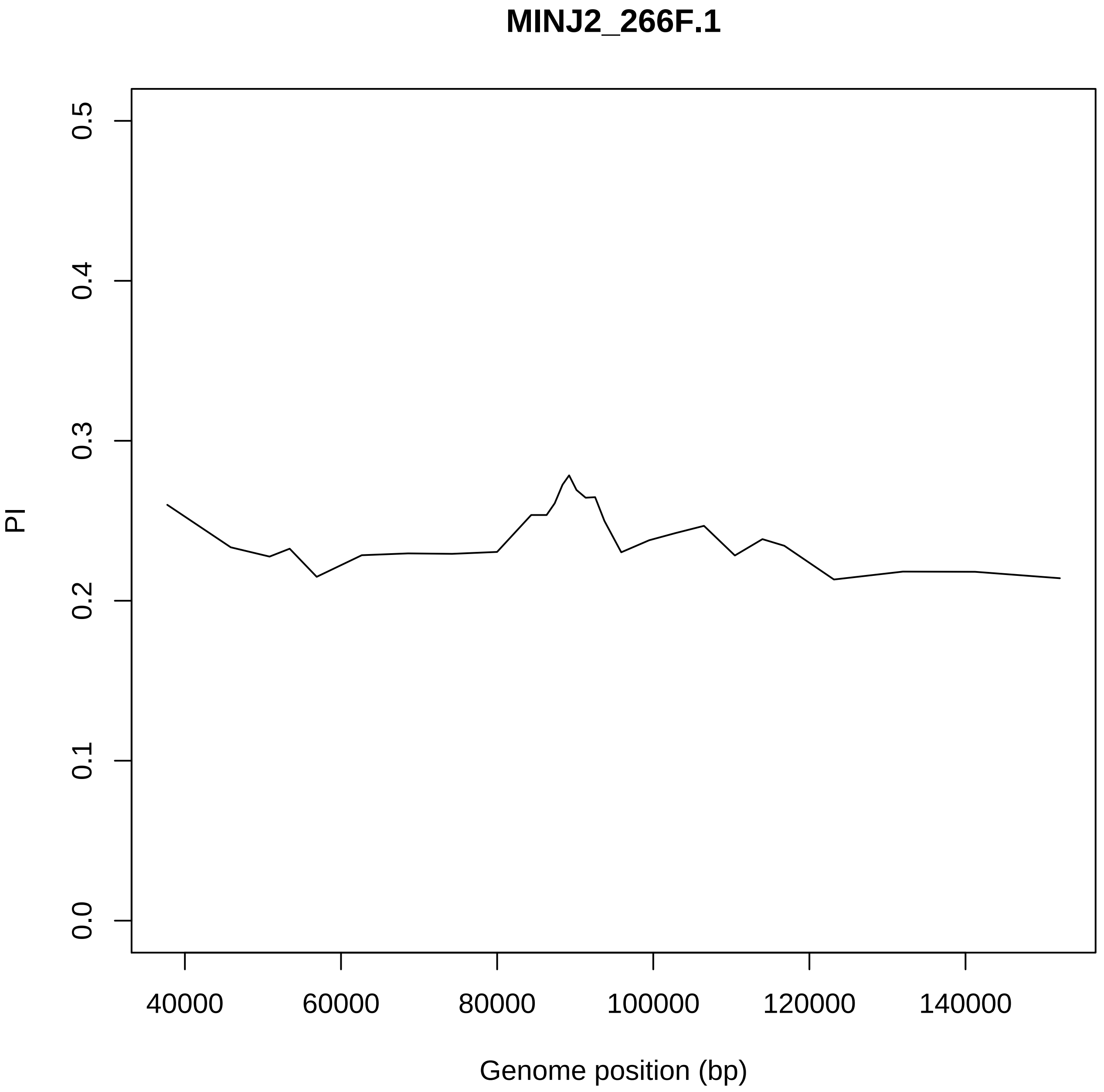
MINJ2_266F.1
PI
0.0
0.1
0.2
0.3
0.4
0.5
40000
60000
80000
100000
120000
140000
Genome position (bp)

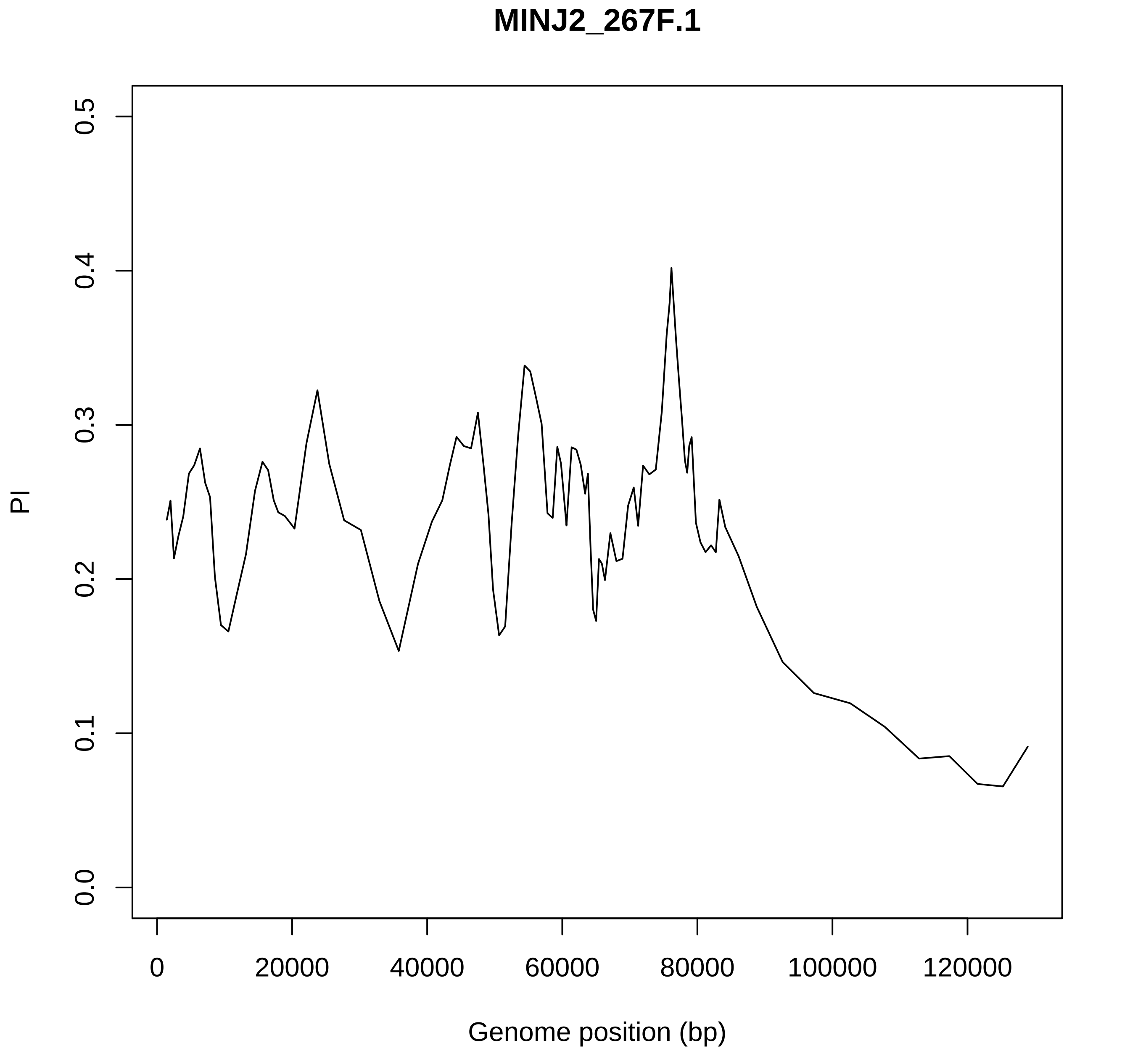
MINJ2_267F.1
PI
0.0
0.1
0.2
0.3
0.4
0.5
0
20000
40000
60000
80000
100000
120000
Genome position (bp)

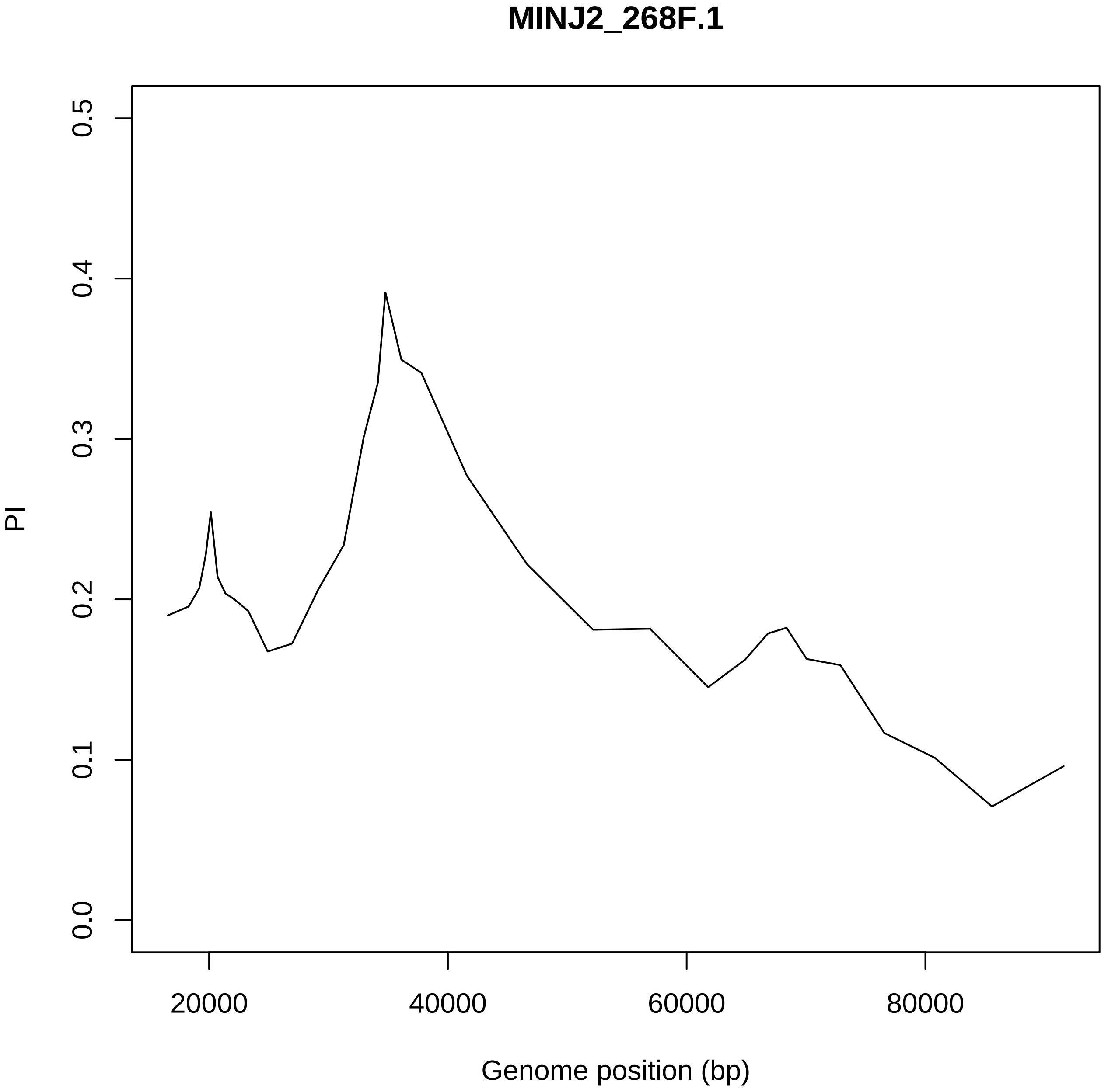
MINJ2_268F.1
PI
0.0
0.1
0.2
0.3
0.4
0.5
20000
40000
60000
80000
Genome position (bp)

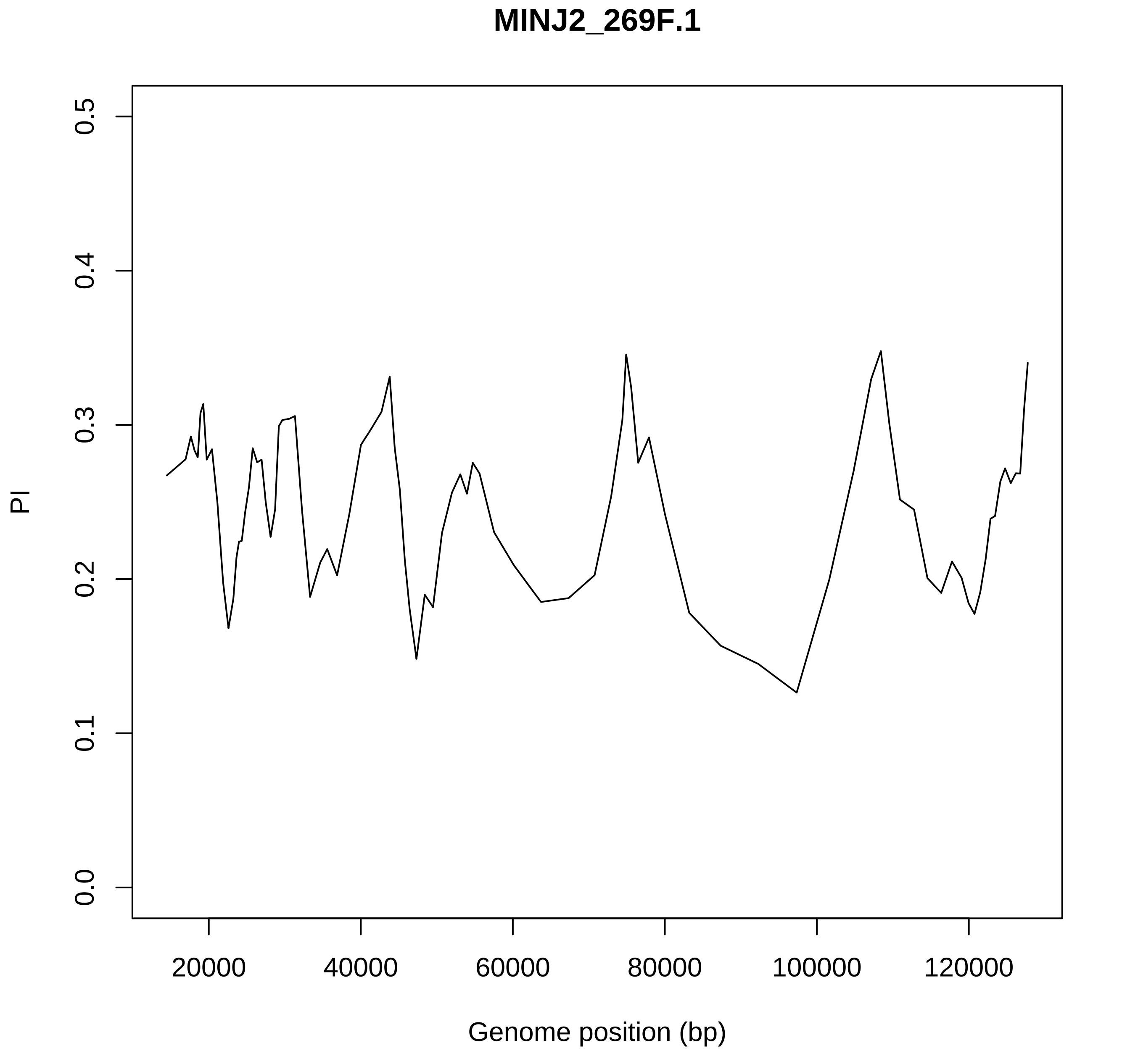
MINJ2_269F.1
PI
0.0
0.1
0.2
0.3
0.4
0.5
20000
40000
60000
80000
100000
120000
Genome position (bp)

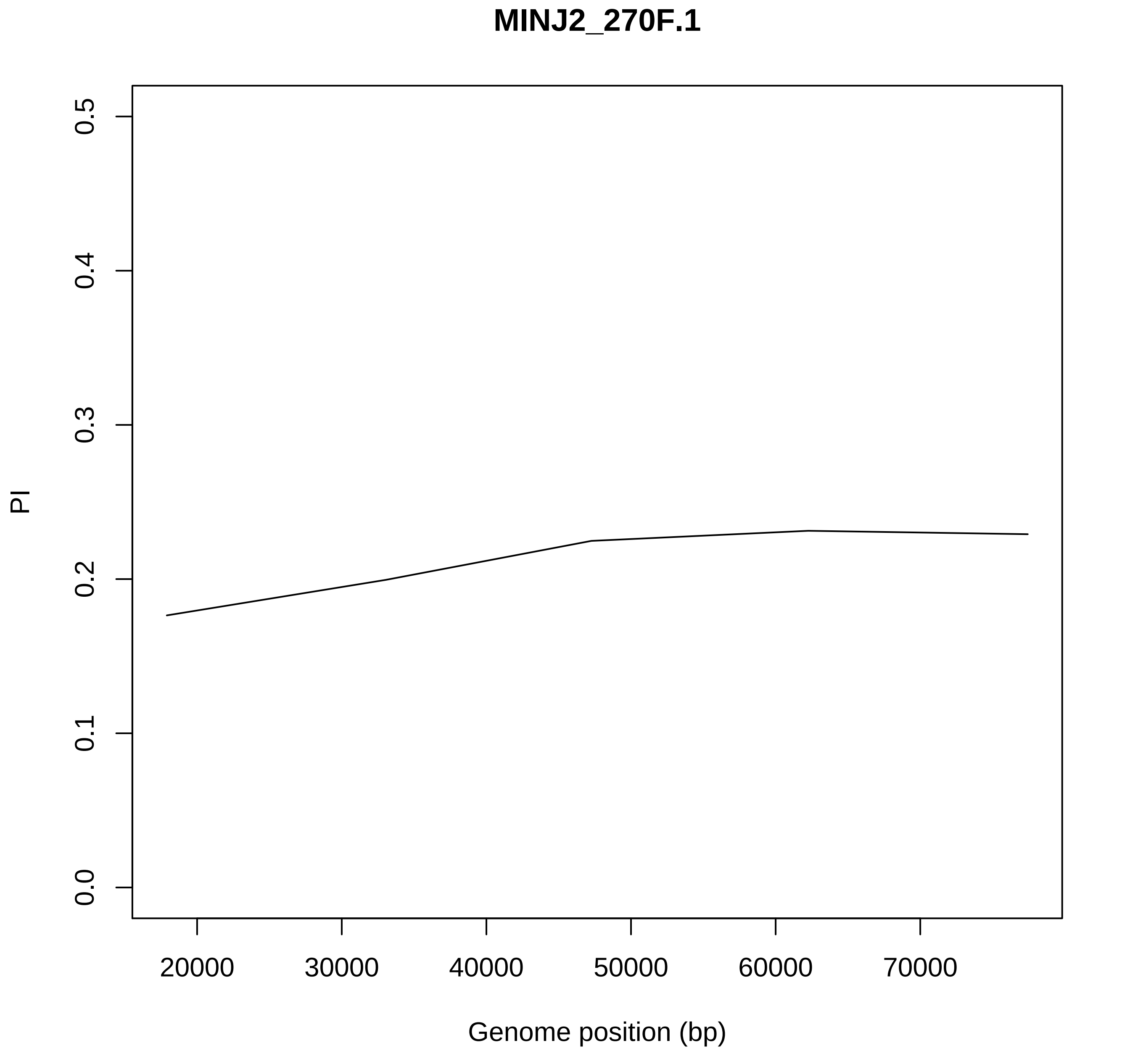
MINJ2_270F.1
PI
0.0
0.1
0.2
0.3
0.4
0.5
20000
30000
40000
50000
60000
70000
Genome position (bp)

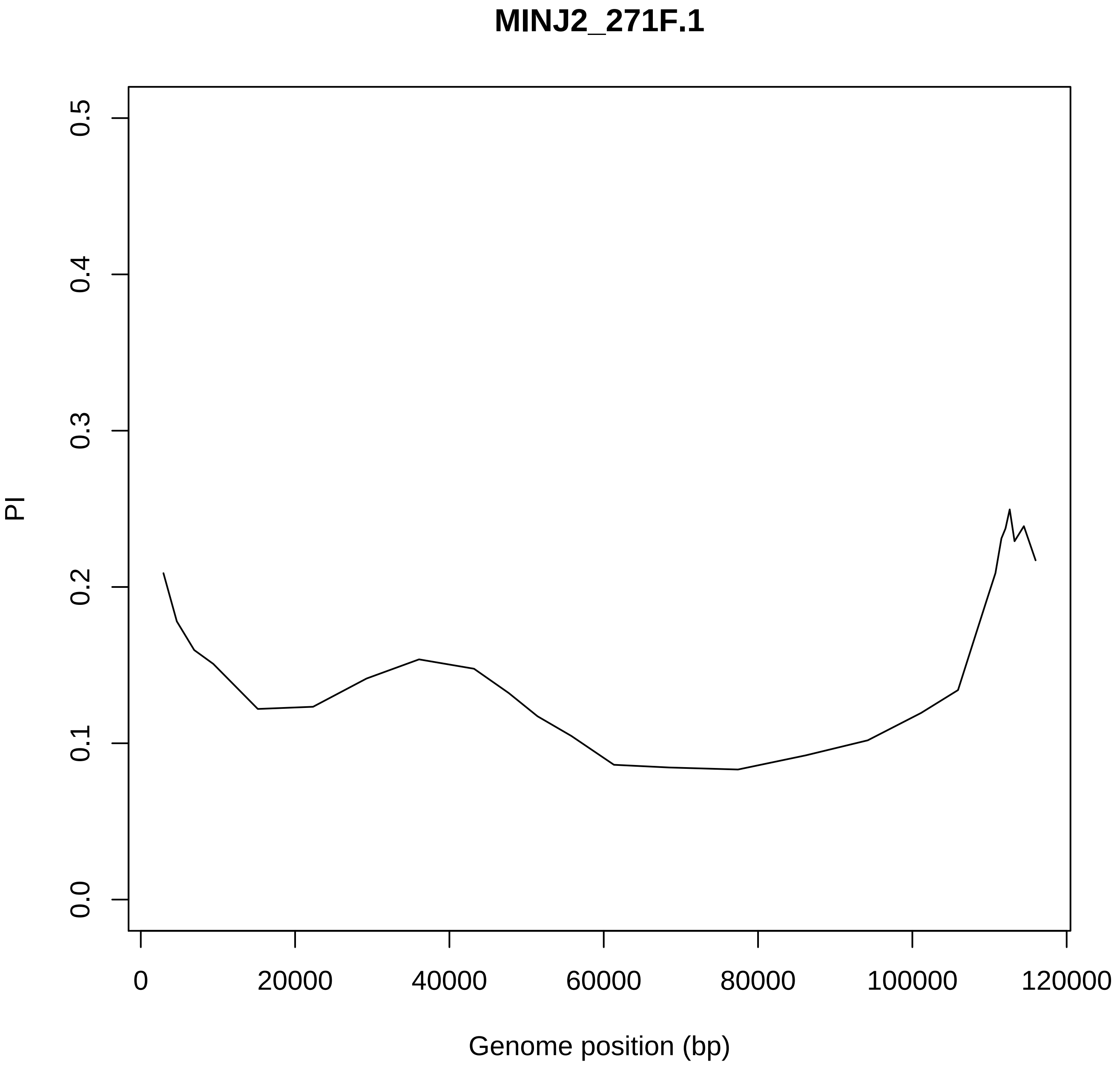

MINJ2_271F.1
PI
0.0
0.1
0.2
0.3
0.4
0.5
0
20000
40000
60000
80000
100000
120000
Genome position (bp)

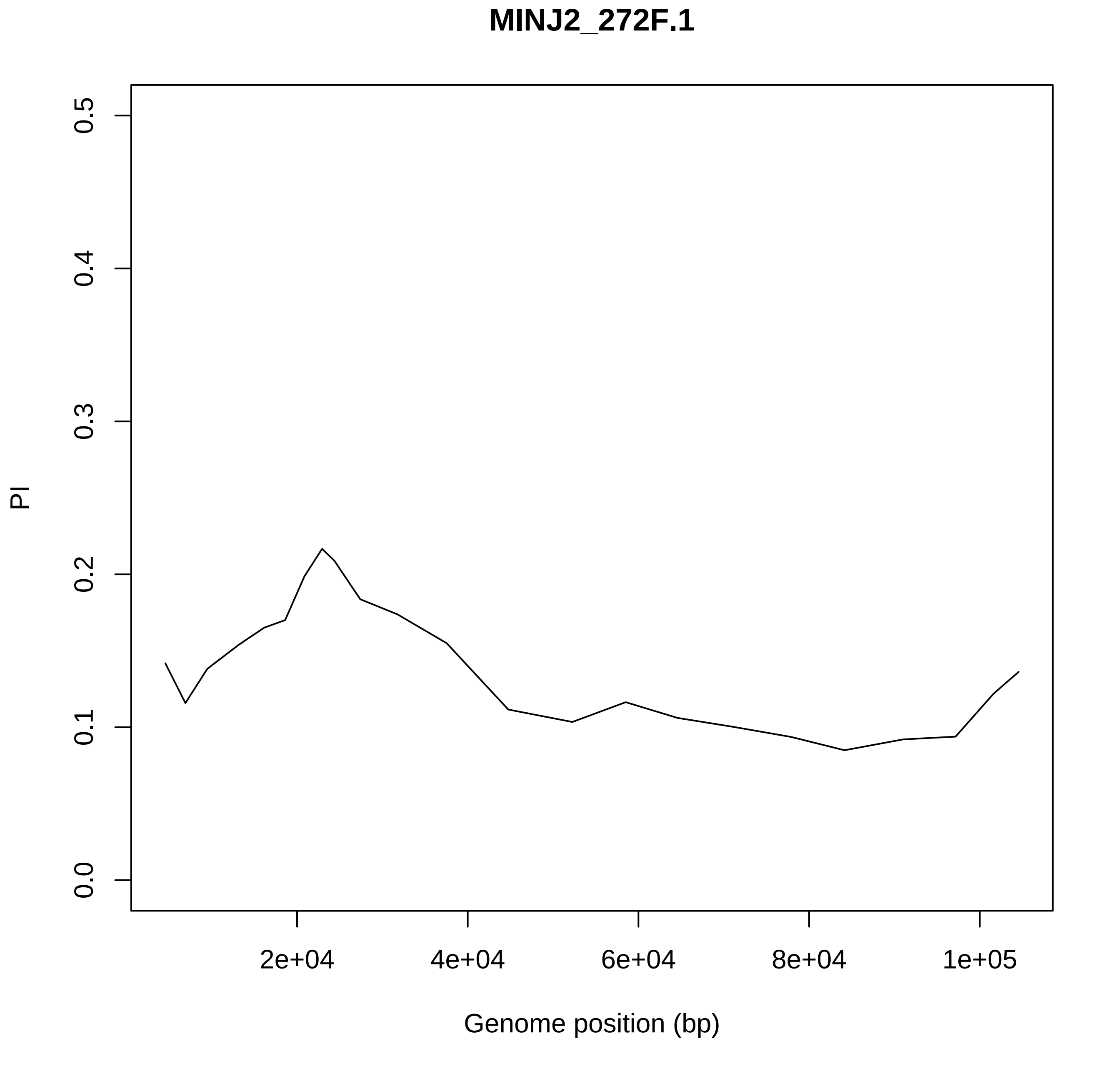
MINJ2_272F.1
PI
0.0
0.1
0.2
0.3
0.4
0.5
2e+04
4e+04
6e+04
8e+04
1e+05
Genome position (bp)

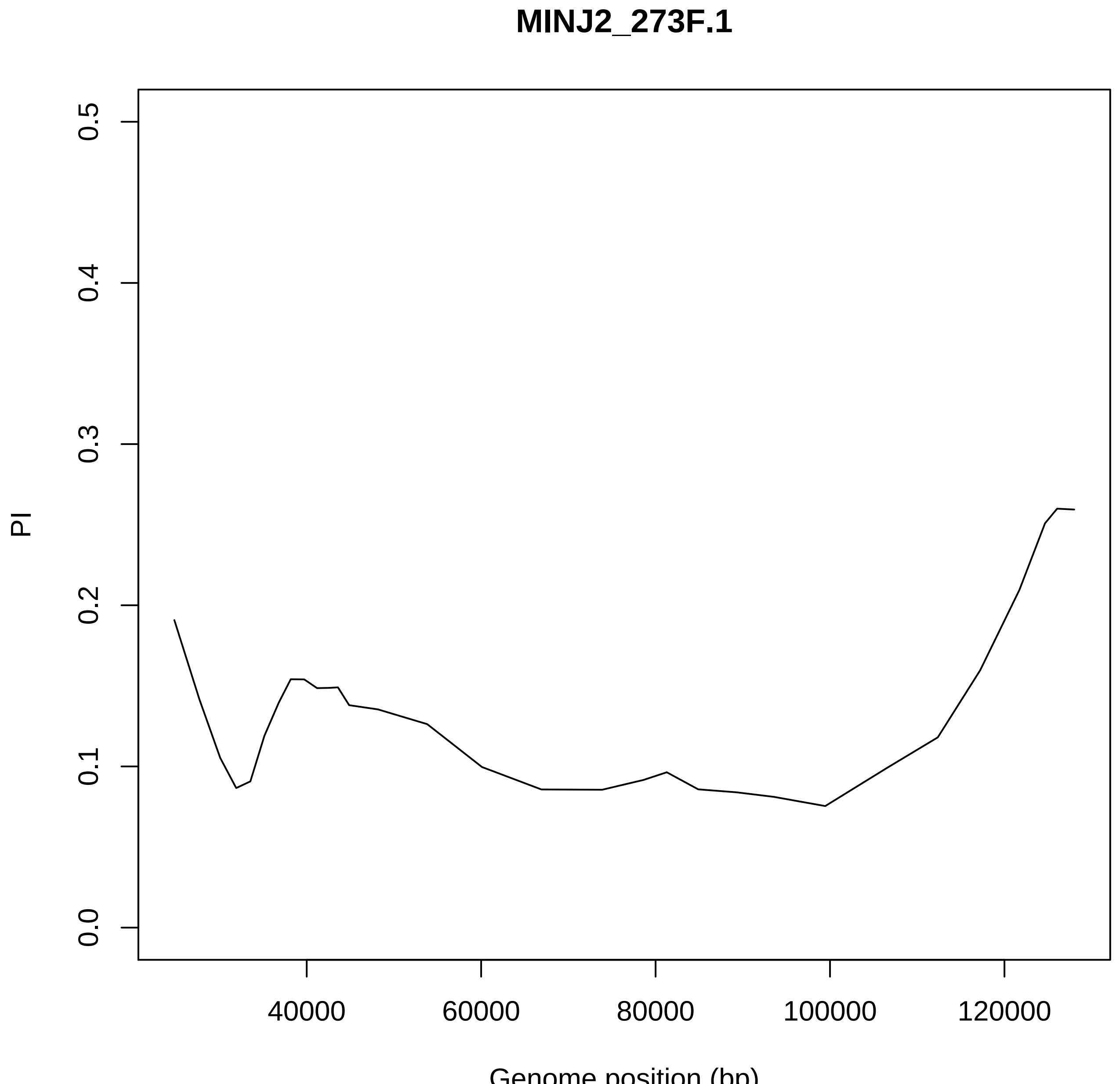

MINJ2_273F.1
PI
0.0
0.1
0.2
0.3
0.4
0.5
40000
60000
80000
100000
120000
Genome position (bp)

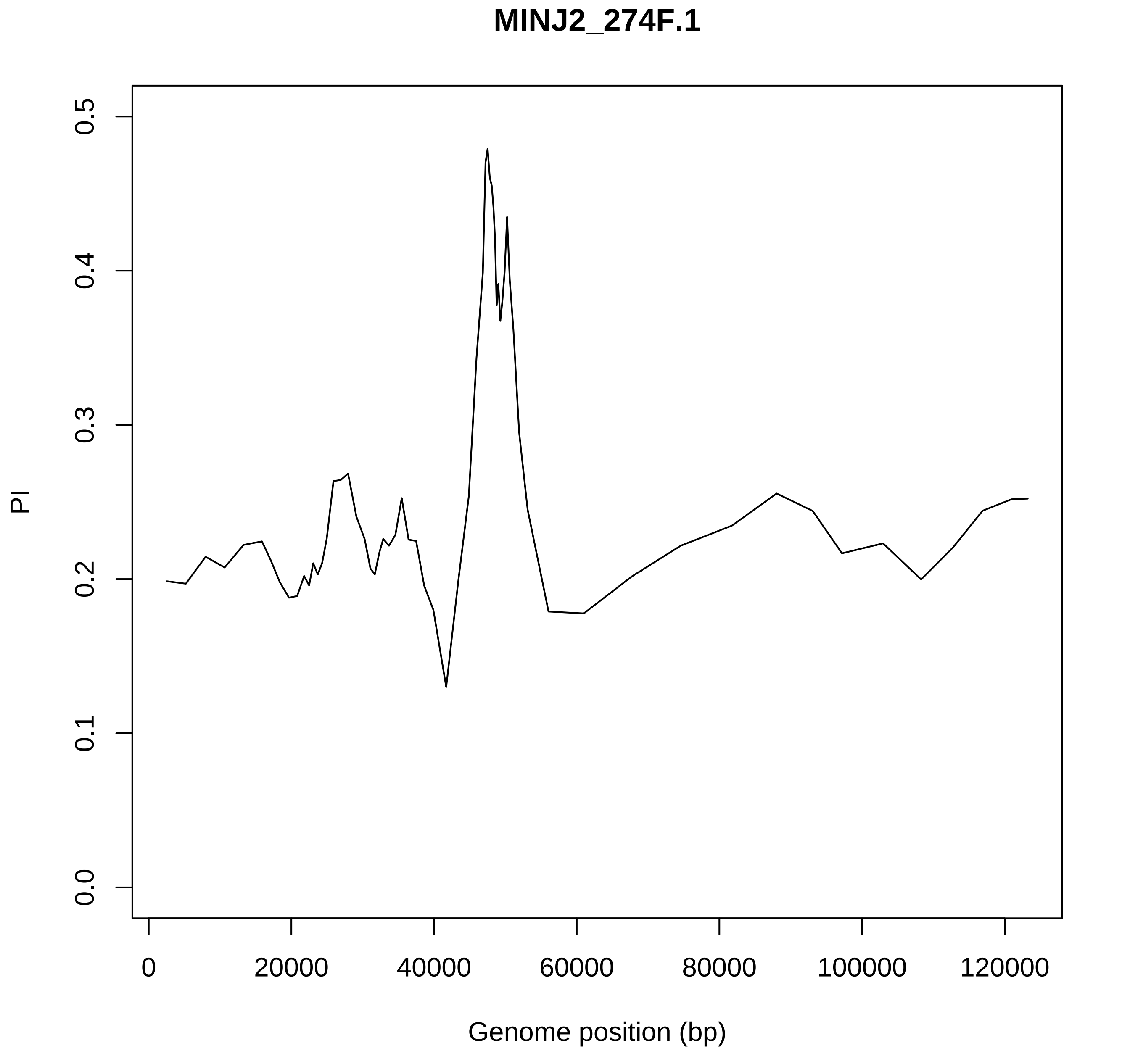

MINJ2_274F.1
PI
0.0
0.1
0.2
0.3
0.4
0.5
0
20000
40000
60000
80000
100000
120000
Genome position (bp)

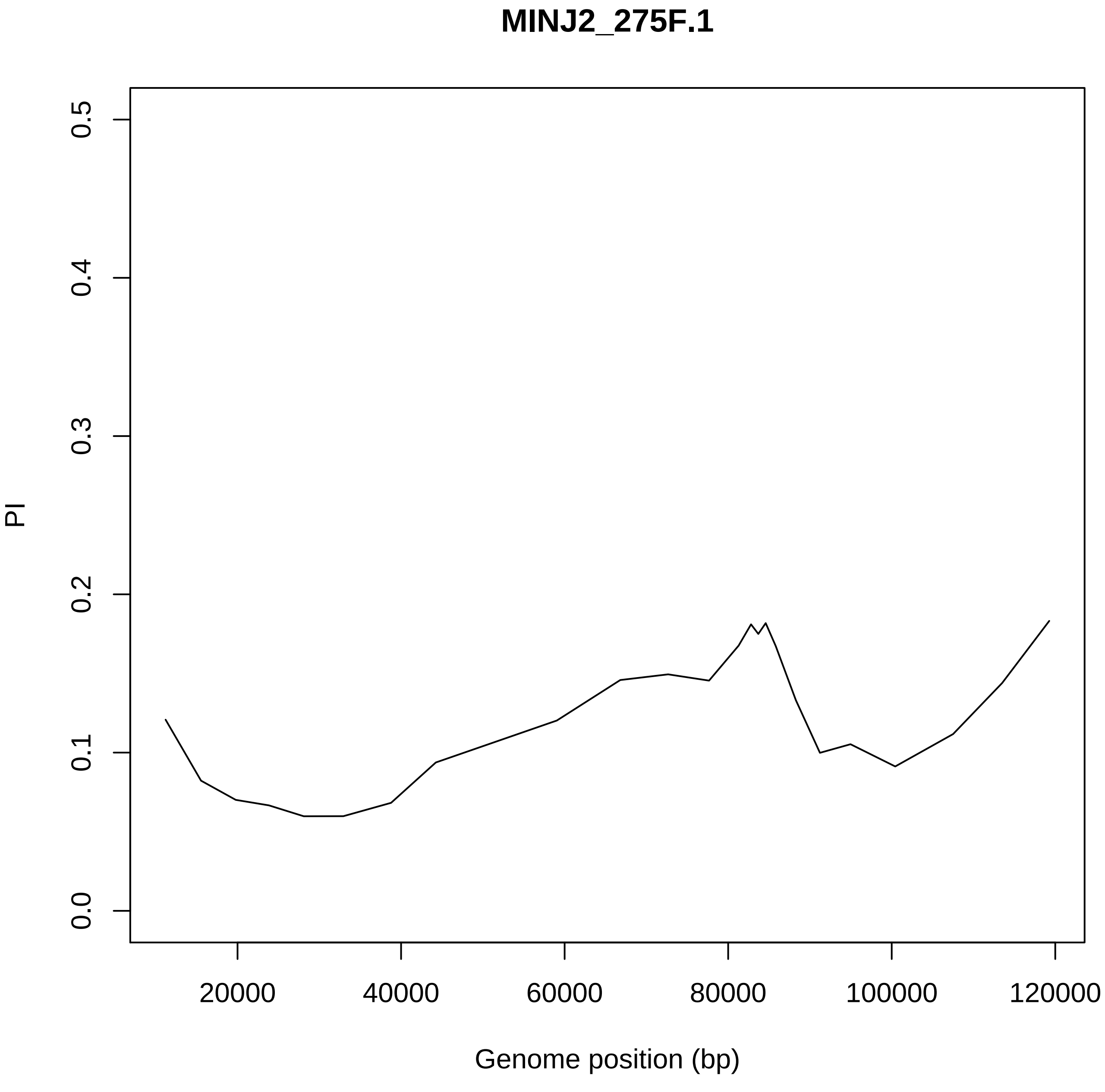
MINJ2_275F.1
PI
0.0
0.1
0.2
0.3
0.4
0.5
20000
40000
60000
80000
100000
120000
Genome position (bp)

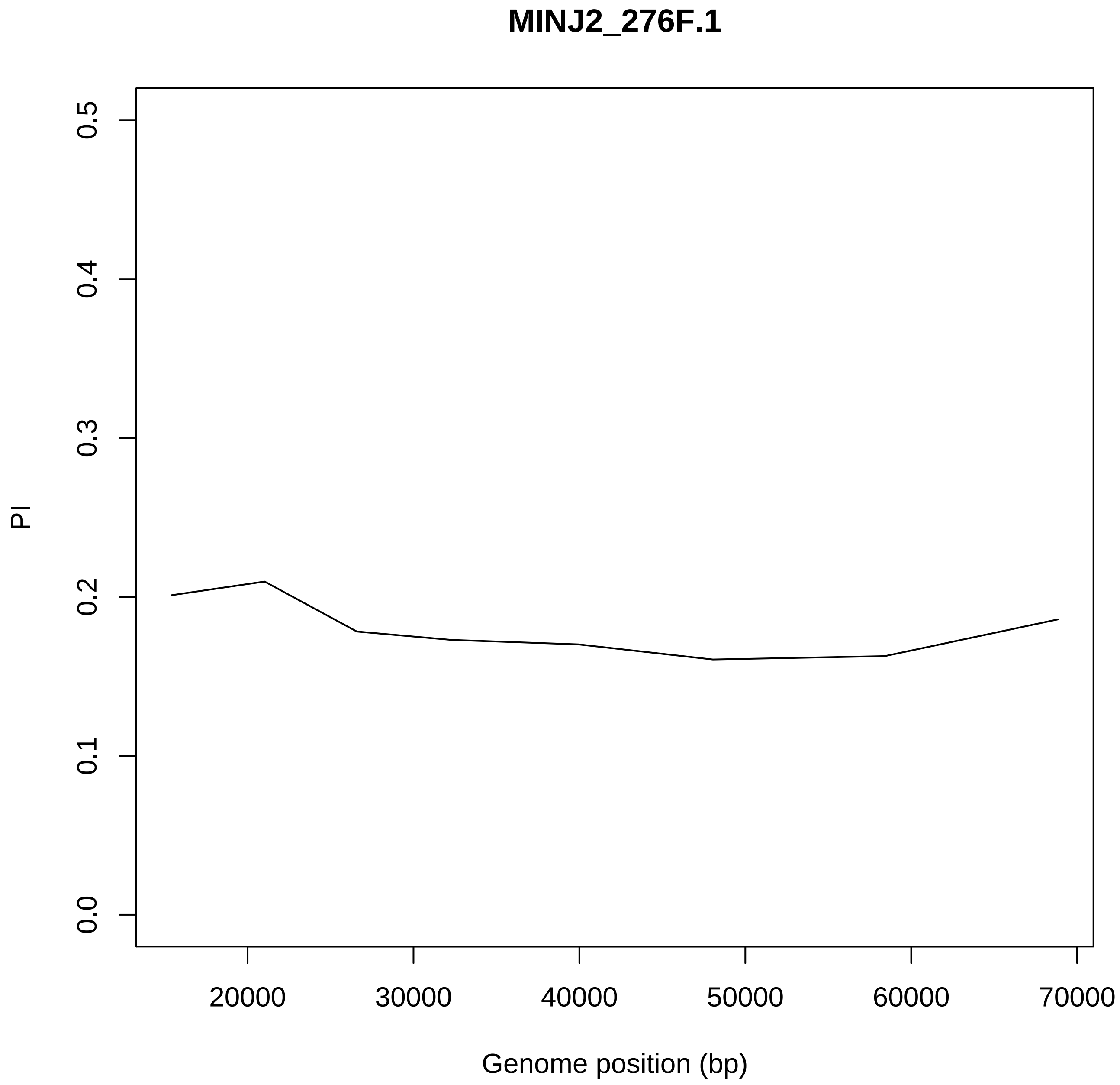
MINJ2_276F.1
PI
0.0
0.1
0.2
0.3
0.4
0.5
20000
30000
40000
50000
60000
70000
Genome position (bp)

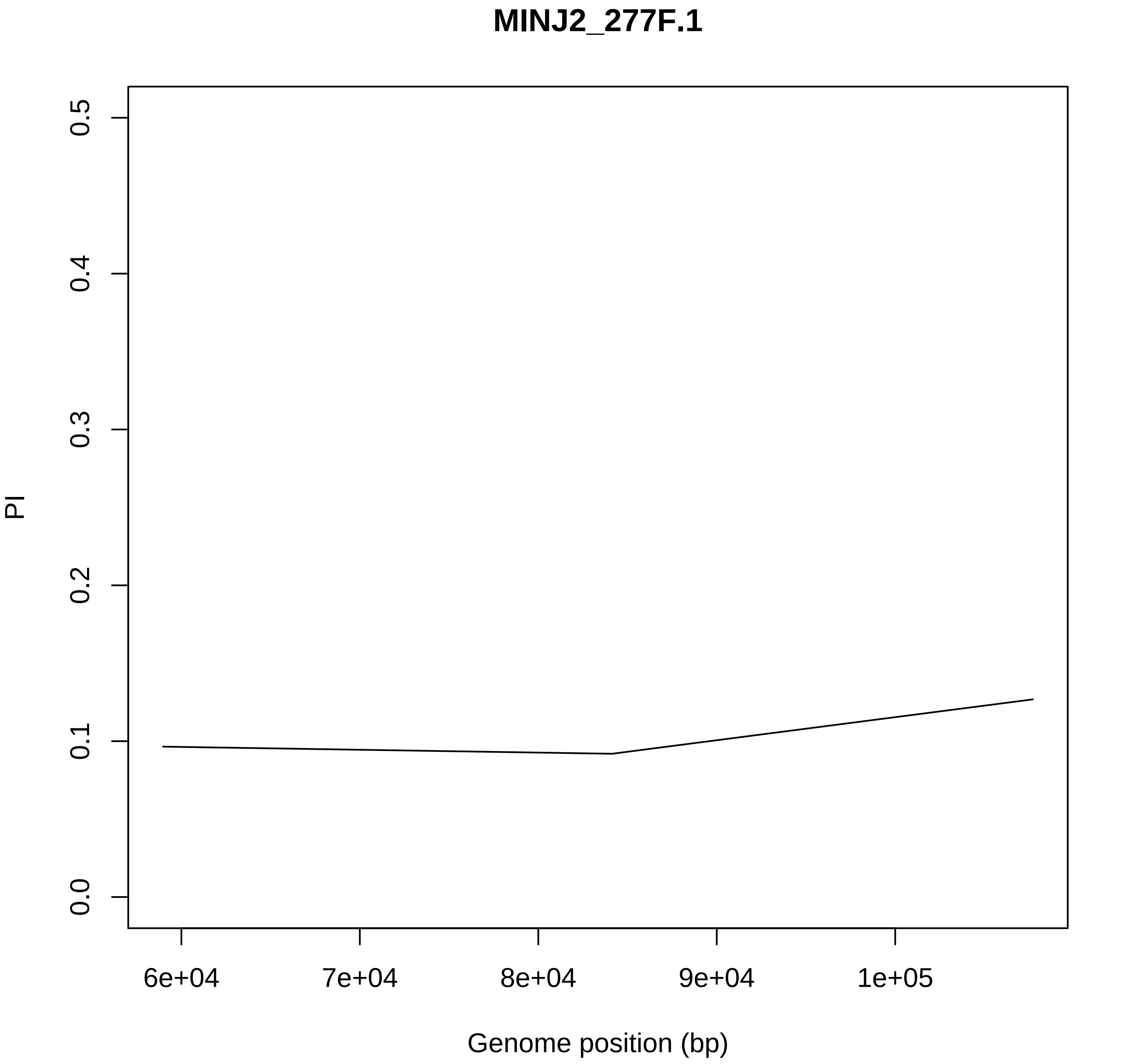
MINJ2_277F.1
PI
0.0
0.1
0.2
0.3
0.4
0.5
6e+04
7e+04
8e+04
9e+04
1e+05
Genome position (bp)

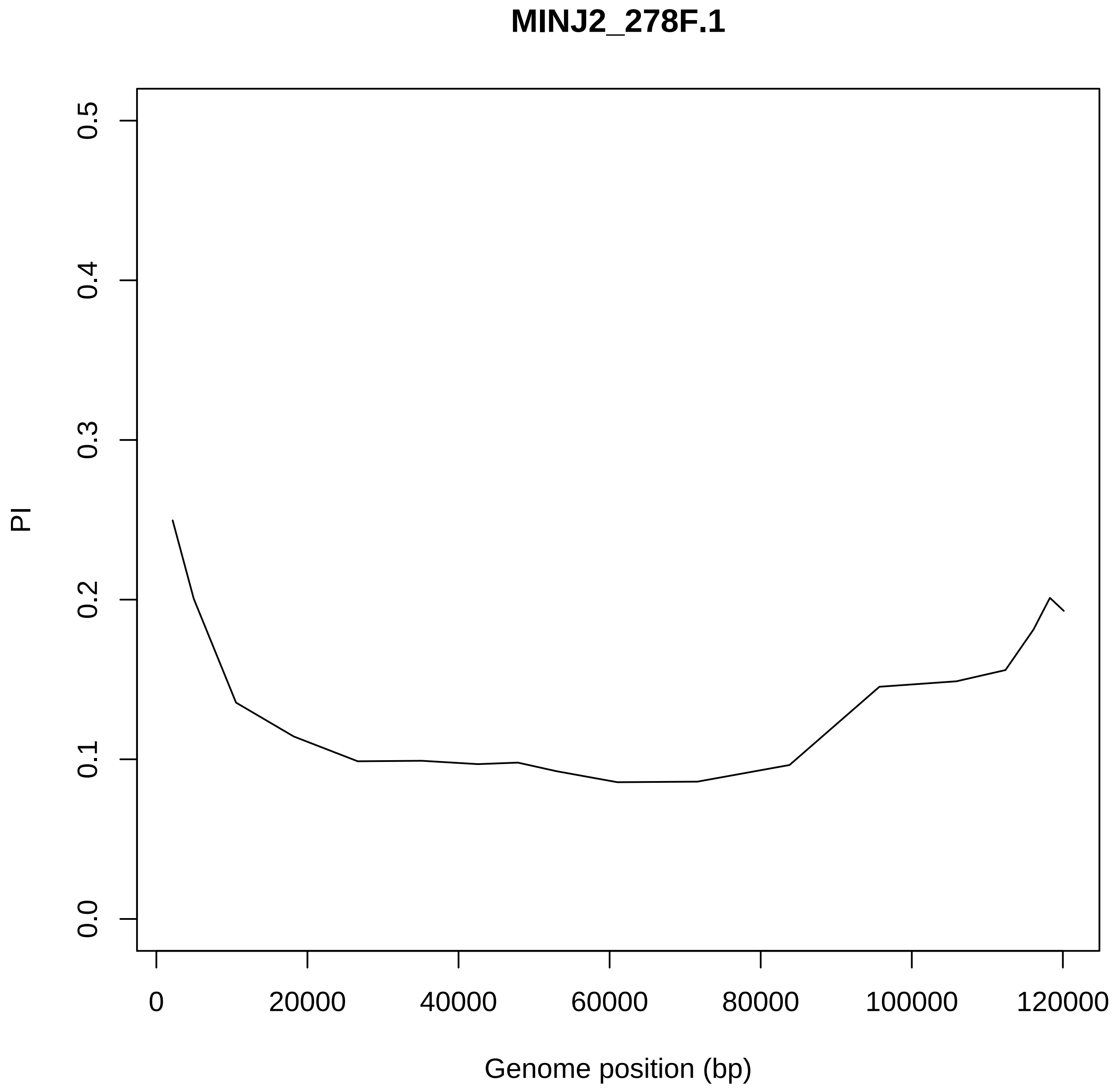

MINJ2_278F.1
PI
0.0
0.1
0.2
0.3
0.4
0.5
0
20000
40000
60000
80000
100000
120000
Genome position (bp)

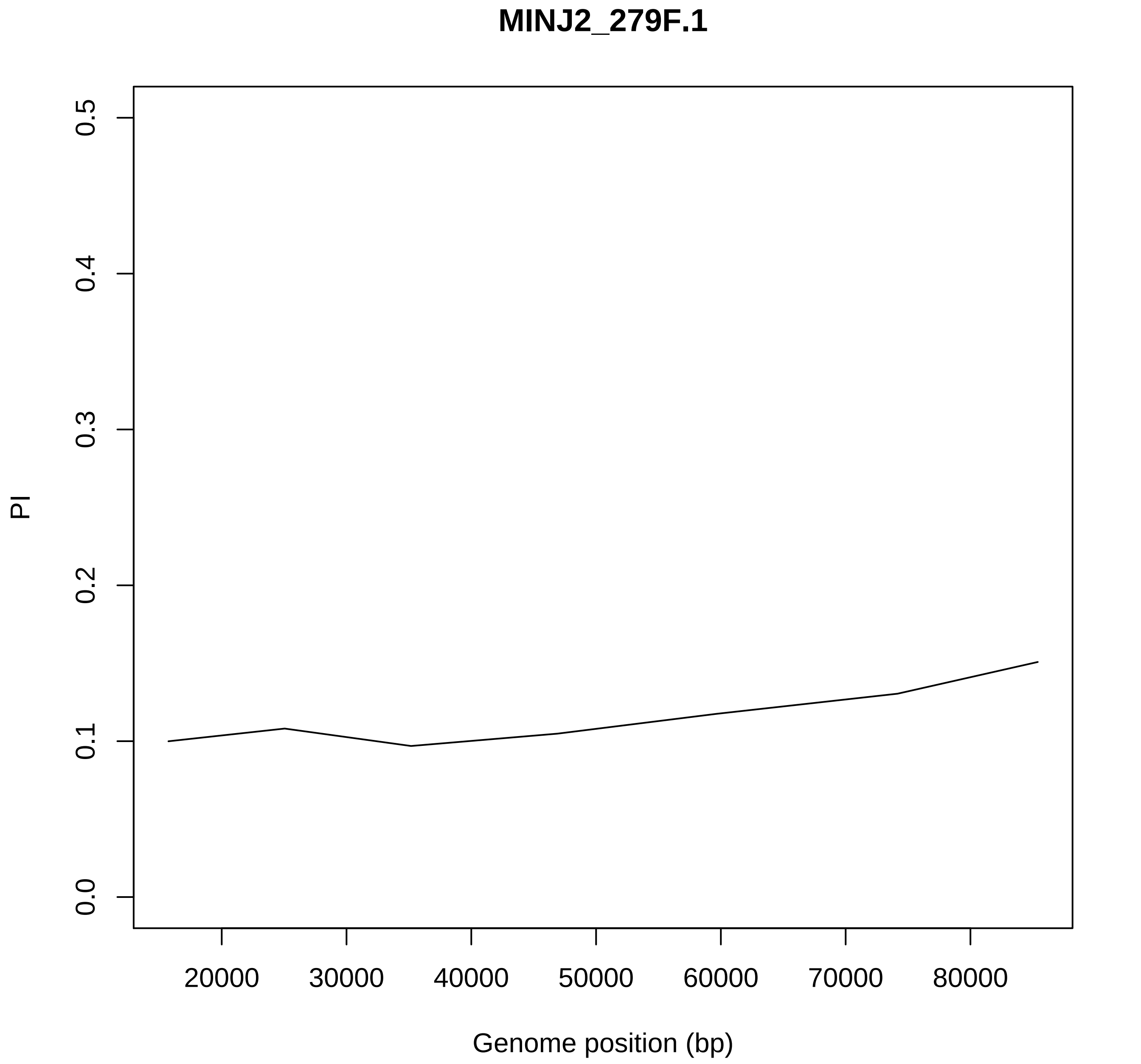

MINJ2_279F.1
PI
0.0
0.1
0.2
0.3
0.4
0.5
20000
30000
40000
50000
60000
70000
80000
Genome position (bp)

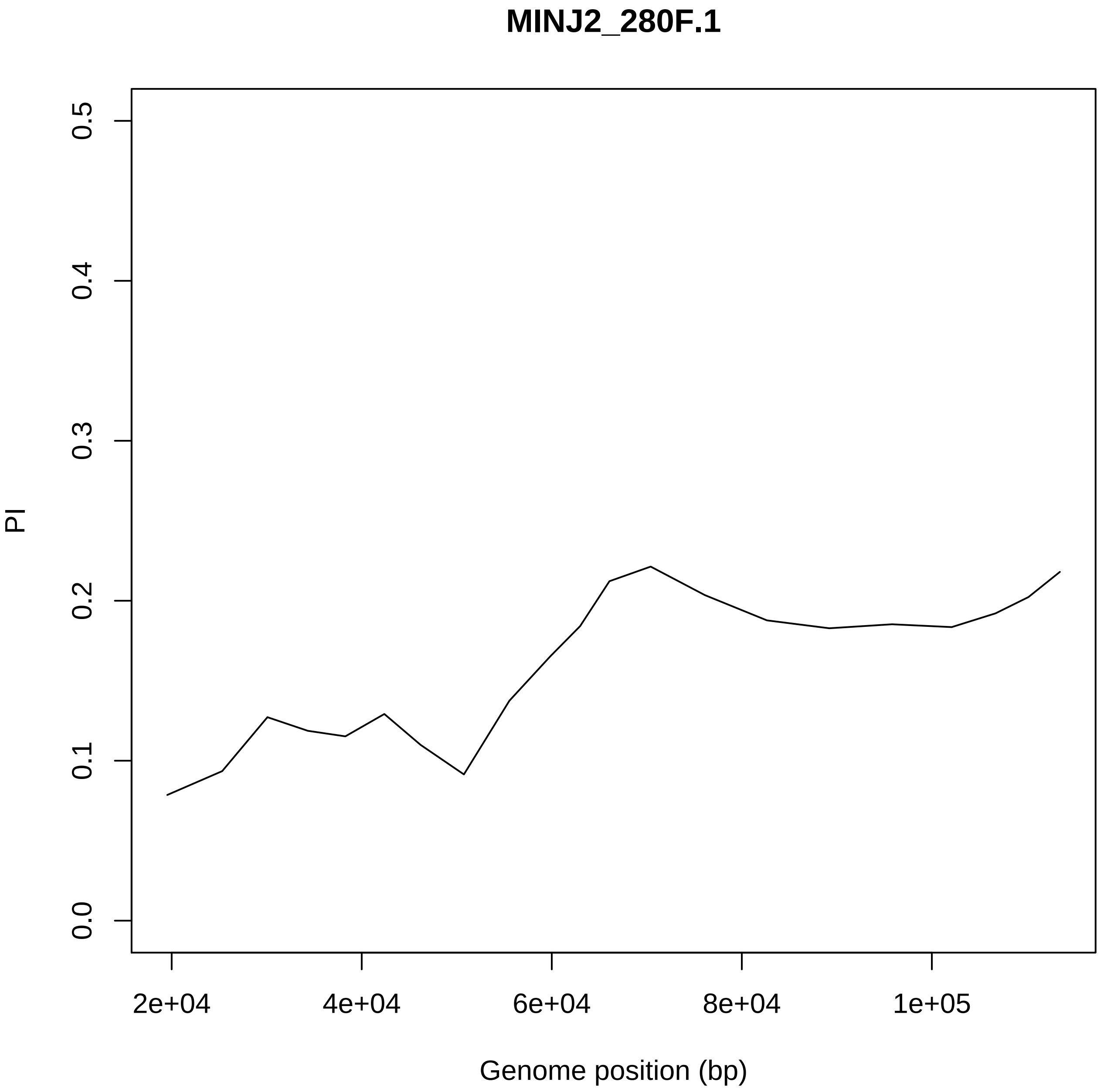
MINJ2_280F.1
PI
0.0
0.1
0.2
0.3
0.4
0.5
2e+04
4e+04
6e+04
8e+04
1e+05
Genome position (bp)

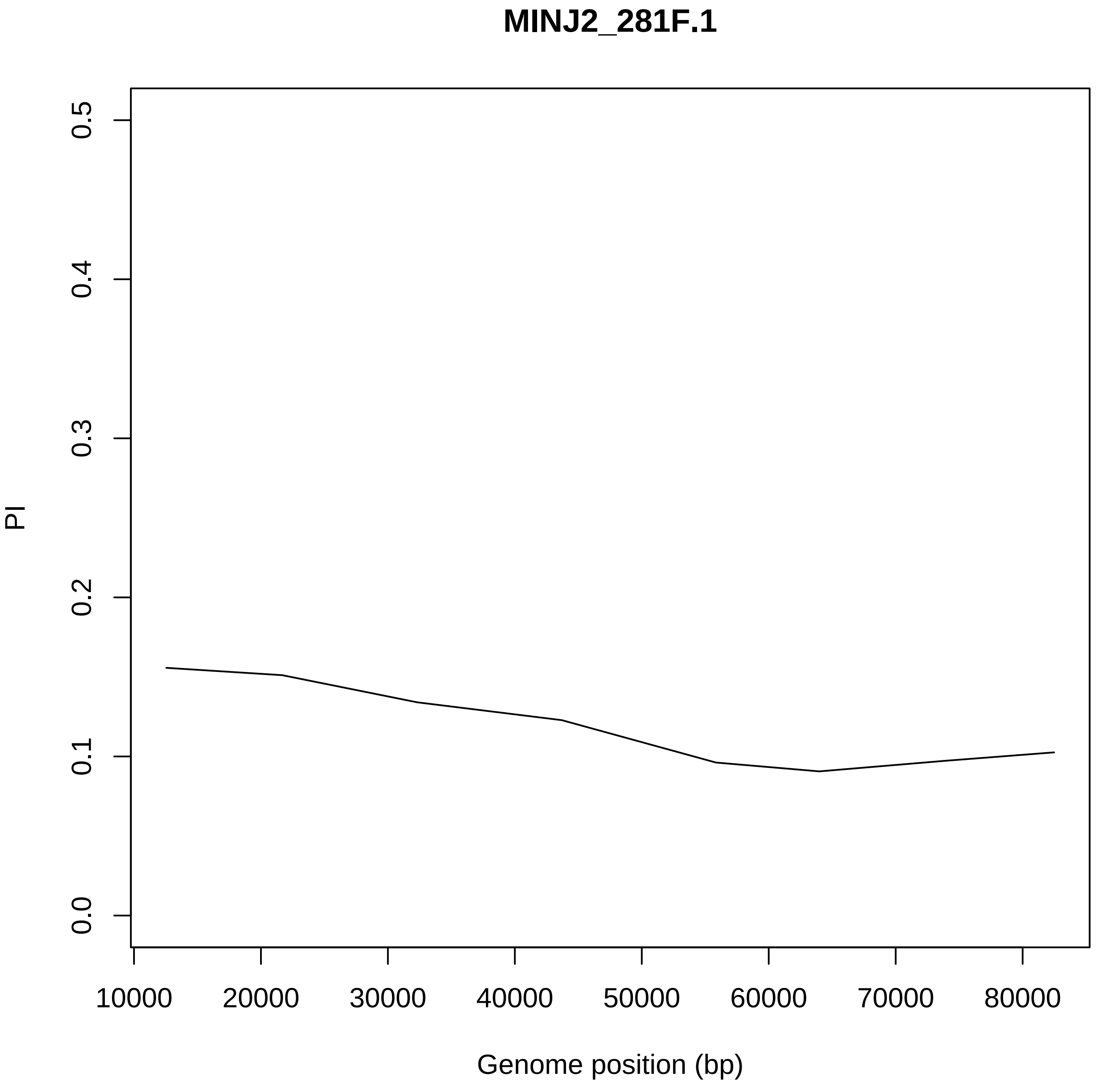
MINJ2_281F.1
PI
0.0
0.1
0.2
0.3
0.4
0.5
10000
20000
30000
40000
50000
60000
70000
80000
Genome position (bp)

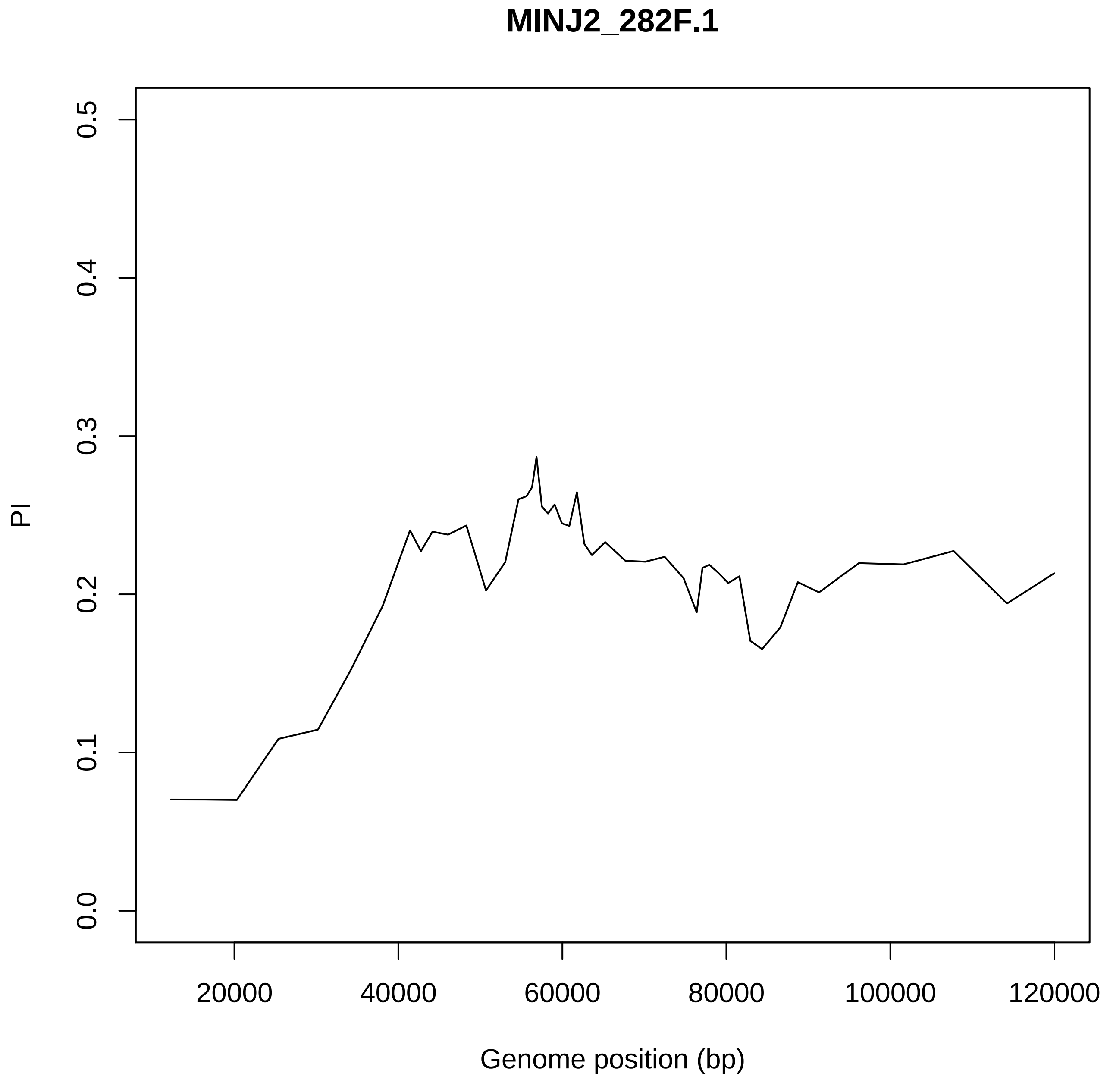
MINJ2_282F.1
PI
0.0
0.1
0.2
0.3
0.4
0.5
20000
40000
60000
80000
100000
120000
Genome position (bp)

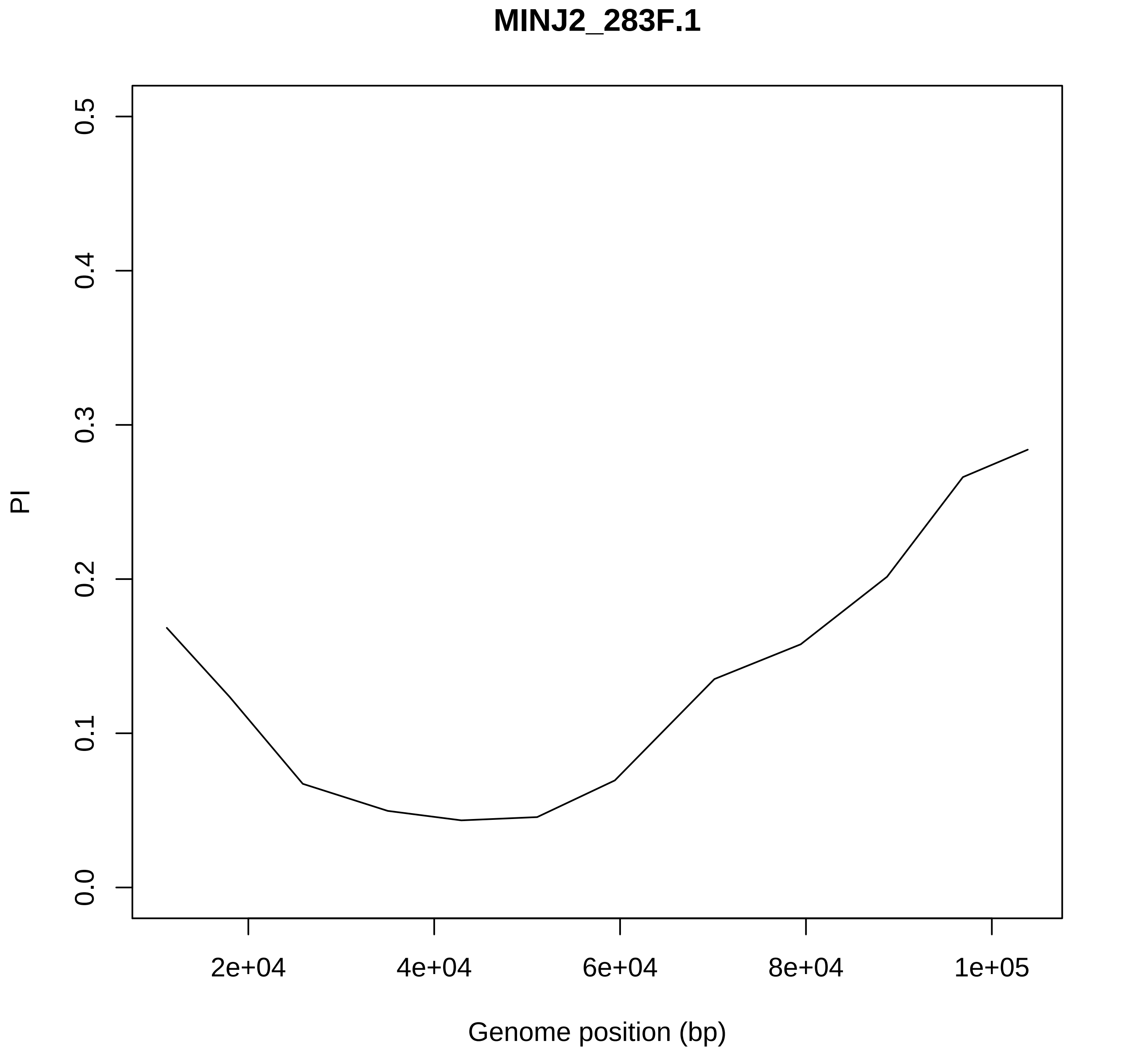
MINJ2_283F.1
PI
0.0
0.1
0.2
0.3
0.4
0.5
2e+04
4e+04
6e+04
8e+04
1e+05
Genome position (bp)

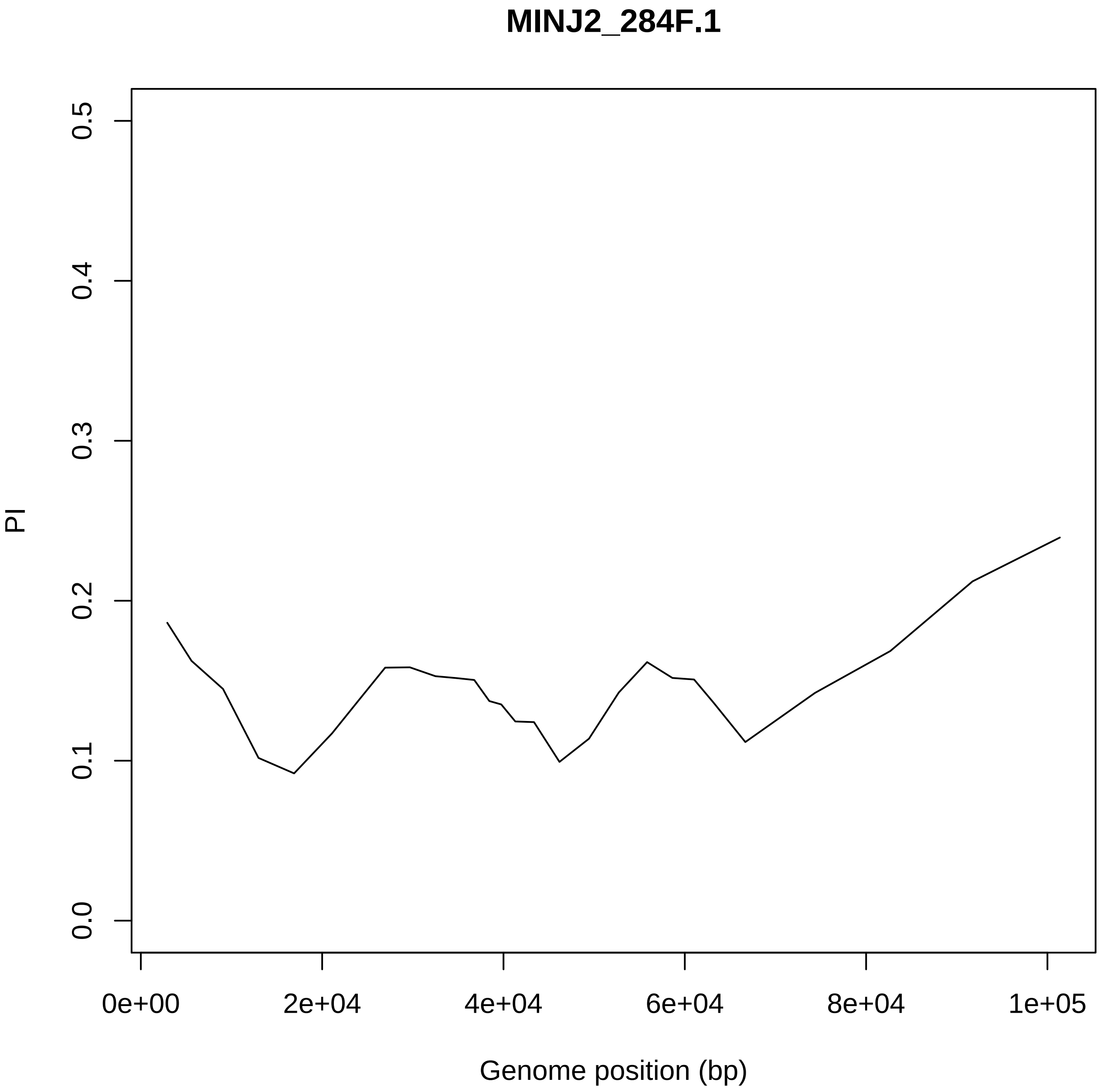
MINJ2_284F.1
PI
0.0
0.1
0.2
0.3
0.4
0.5
0e+00
2e+04
4e+04
6e+04
8e+04
1e+05
Genome position (bp)

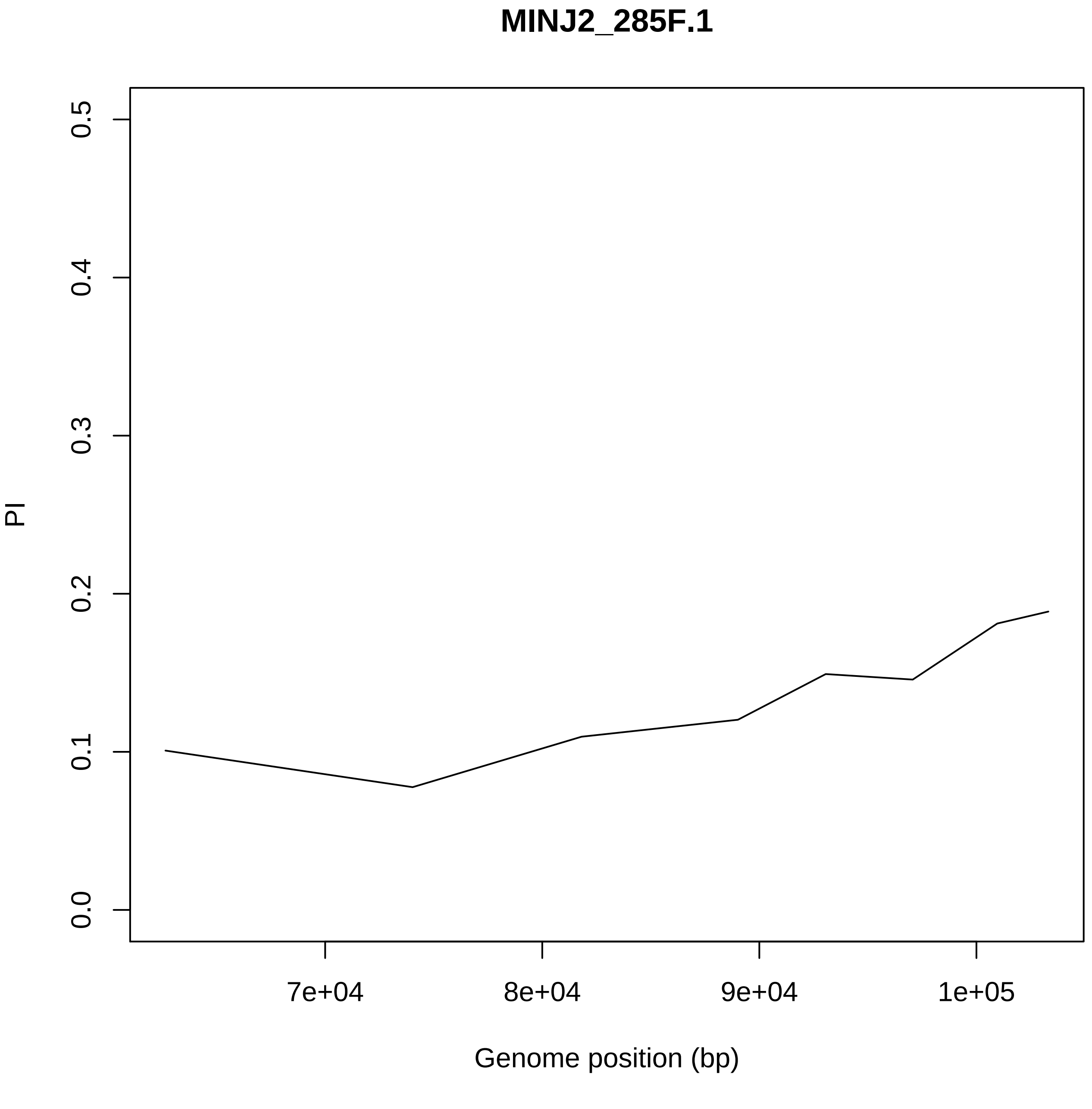
MINJ2_285F.1
PI
0.0
0.1
0.2
0.3
0.4
0.5
7e+04
8e+04
9e+04
1e+05
Genome position (bp)

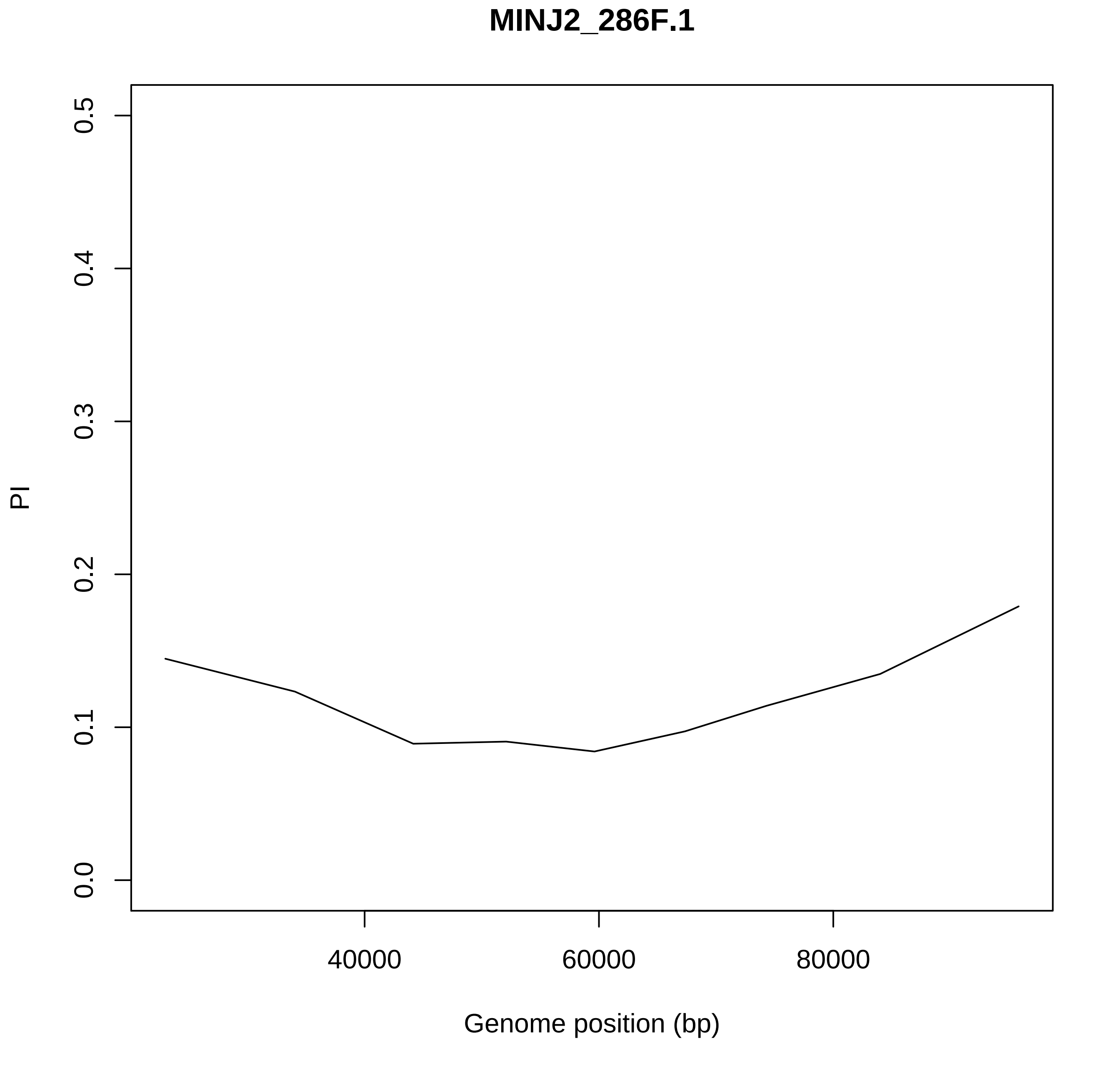
MINJ2_286F.1
PI
0.0
0.1
0.2
0.3
0.4
0.5
40000
60000
80000
Genome position (bp)

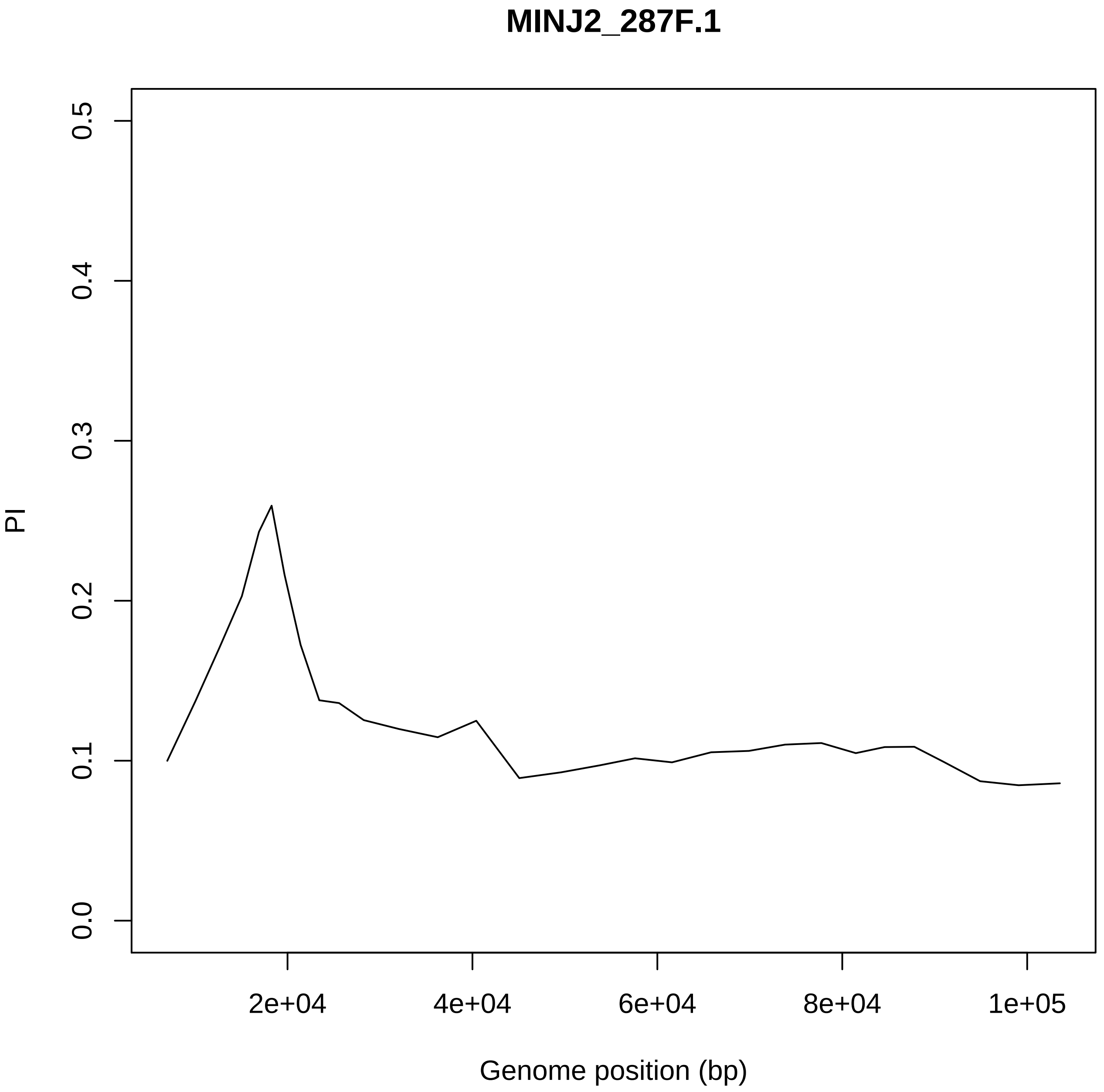
MINJ2_287F.1
PI
0.0
0.1
0.2
0.3
0.4
0.5
2e+04
4e+04
6e+04
8e+04
1e+05
Genome position (bp)

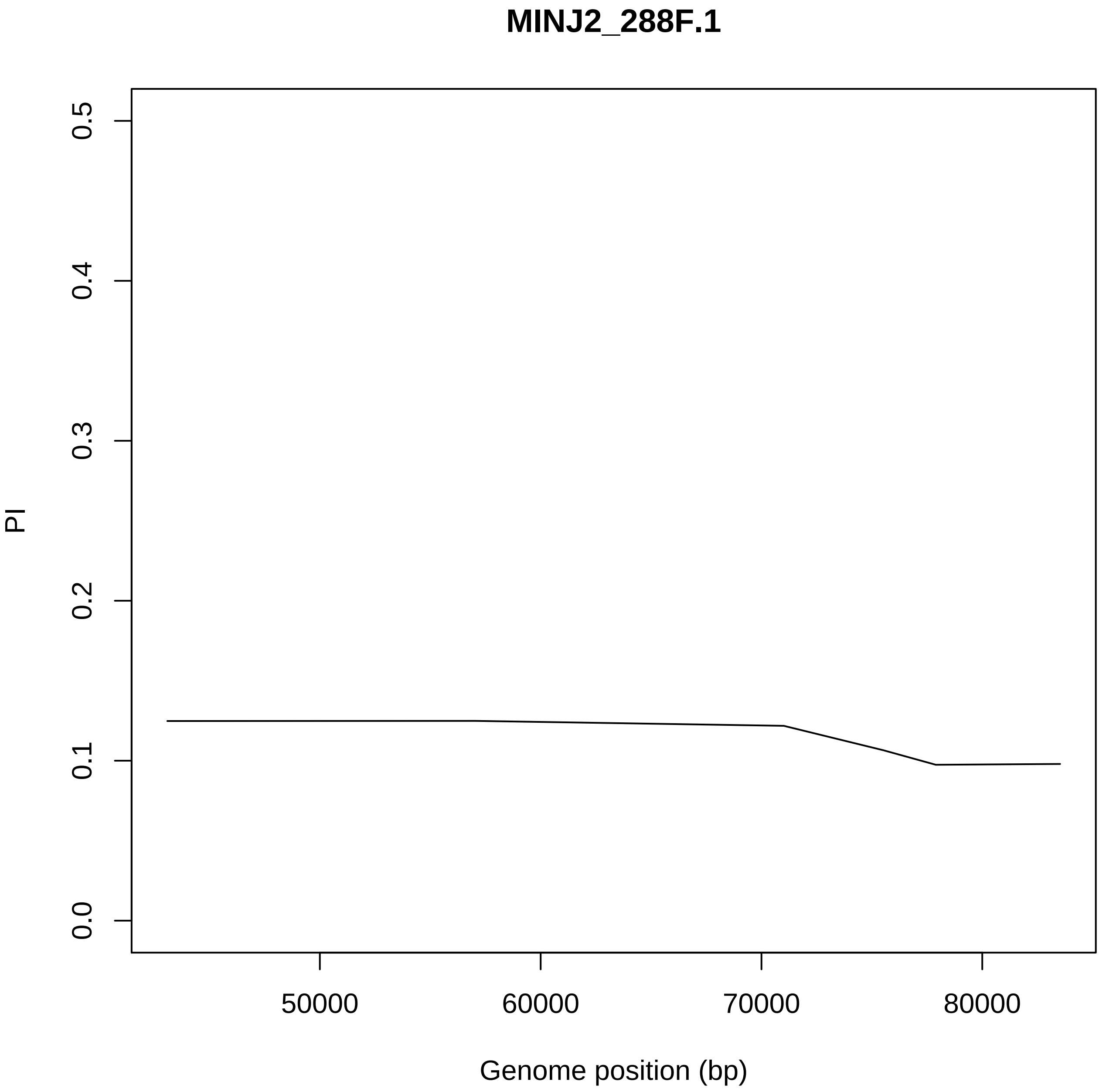
MINJ2_288F.1
0.0
0.1
0.2
0.3
0.4
0.5
PI
50000
60000
70000
80000
Genome position (bp)

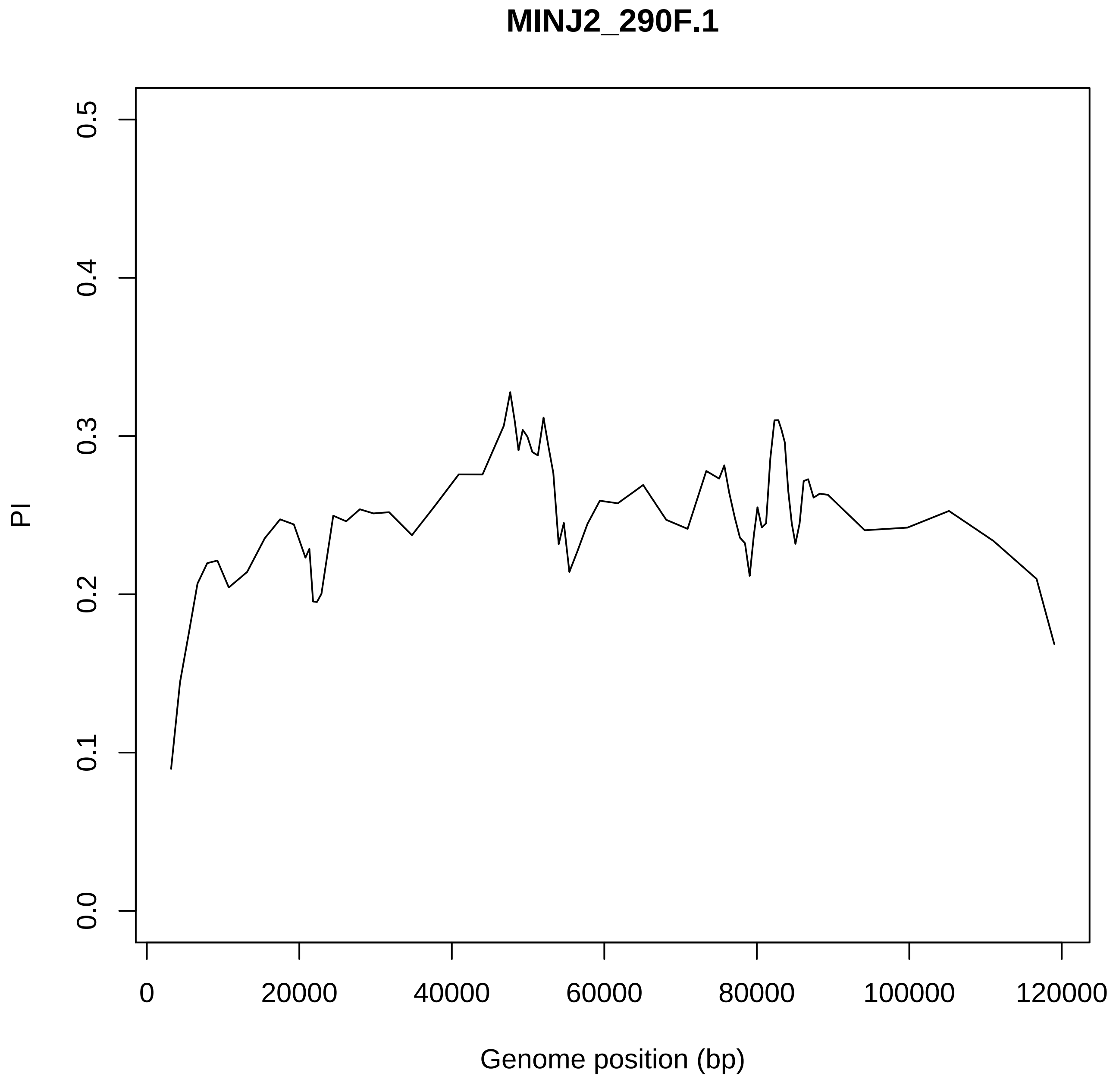
MINJ2_290F.1
PI
0.0
0.1
0.2
0.3
0.4
0.5
0
20000
40000
60000
80000
100000
120000
Genome position (bp)

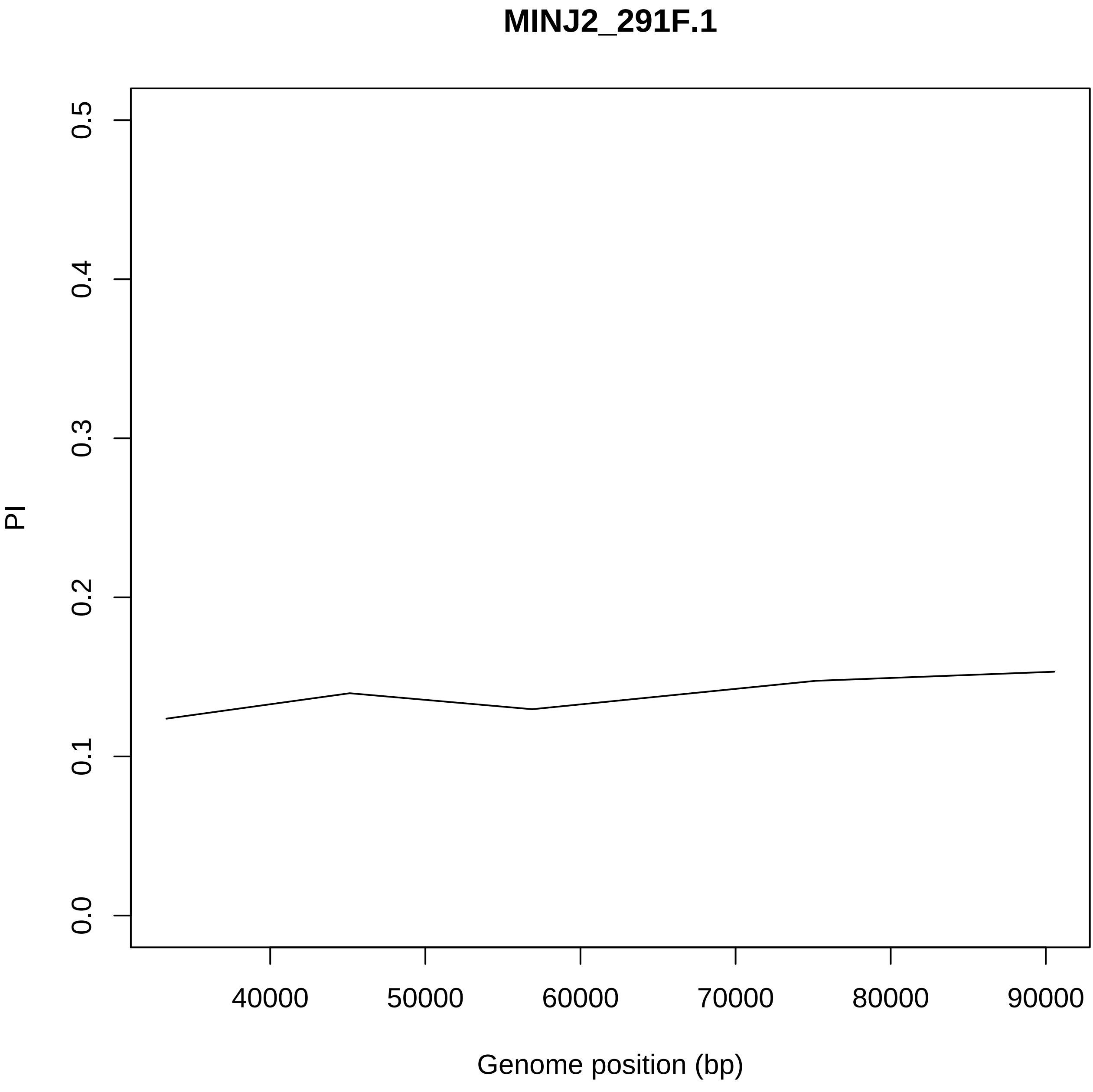
MINJ2_291F.1
PI
0.0
0.1
0.2
0.3
0.4
0.5
40000
50000
60000
70000
80000
90000
Genome position (bp)

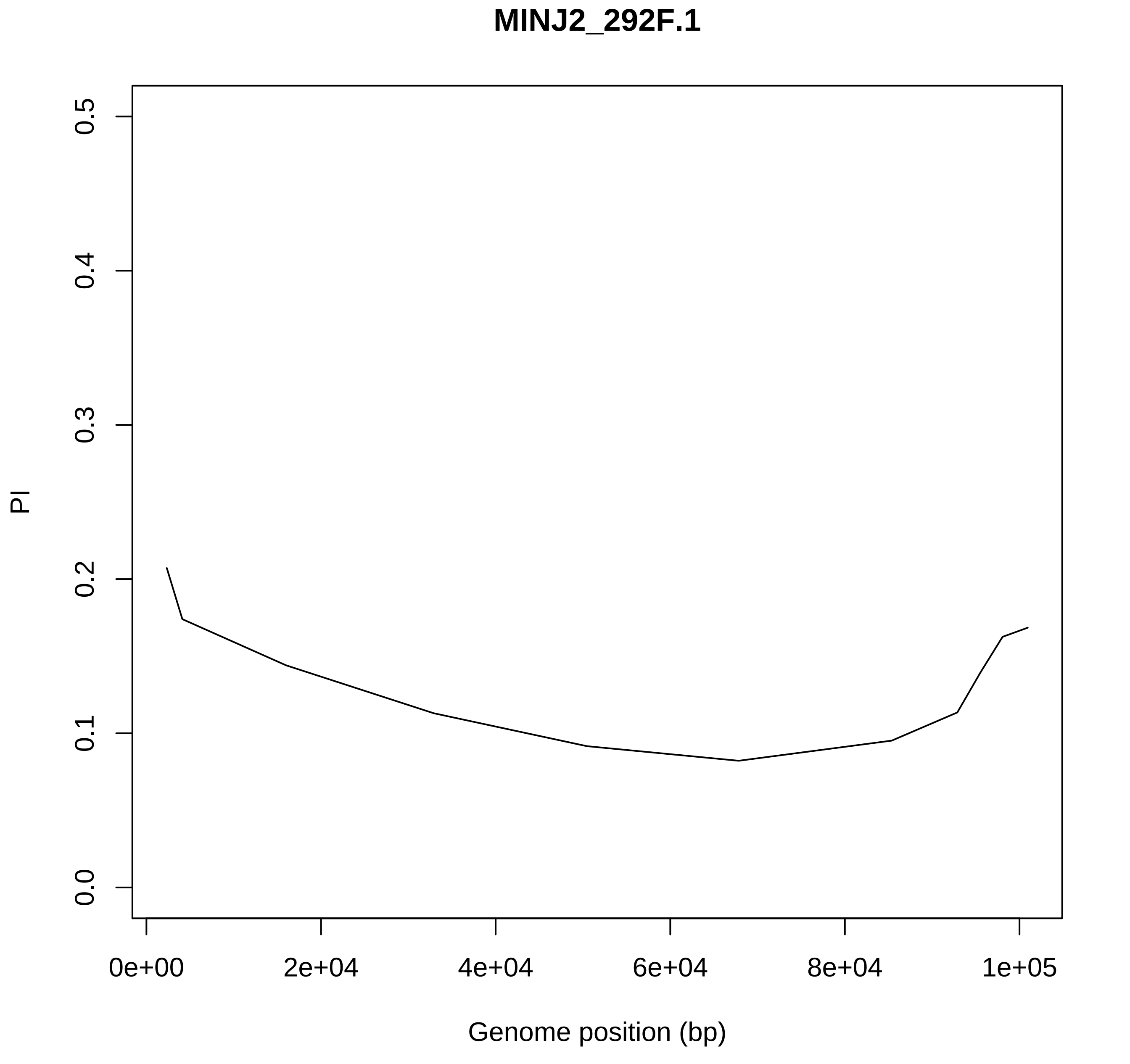
MINJ2_292F.1
PI
0.0
0.1
0.2
0.3
0.4
0.5
0e+00
2e+04
4e+04
6e+04
8e+04
1e+05
Genome position (bp)

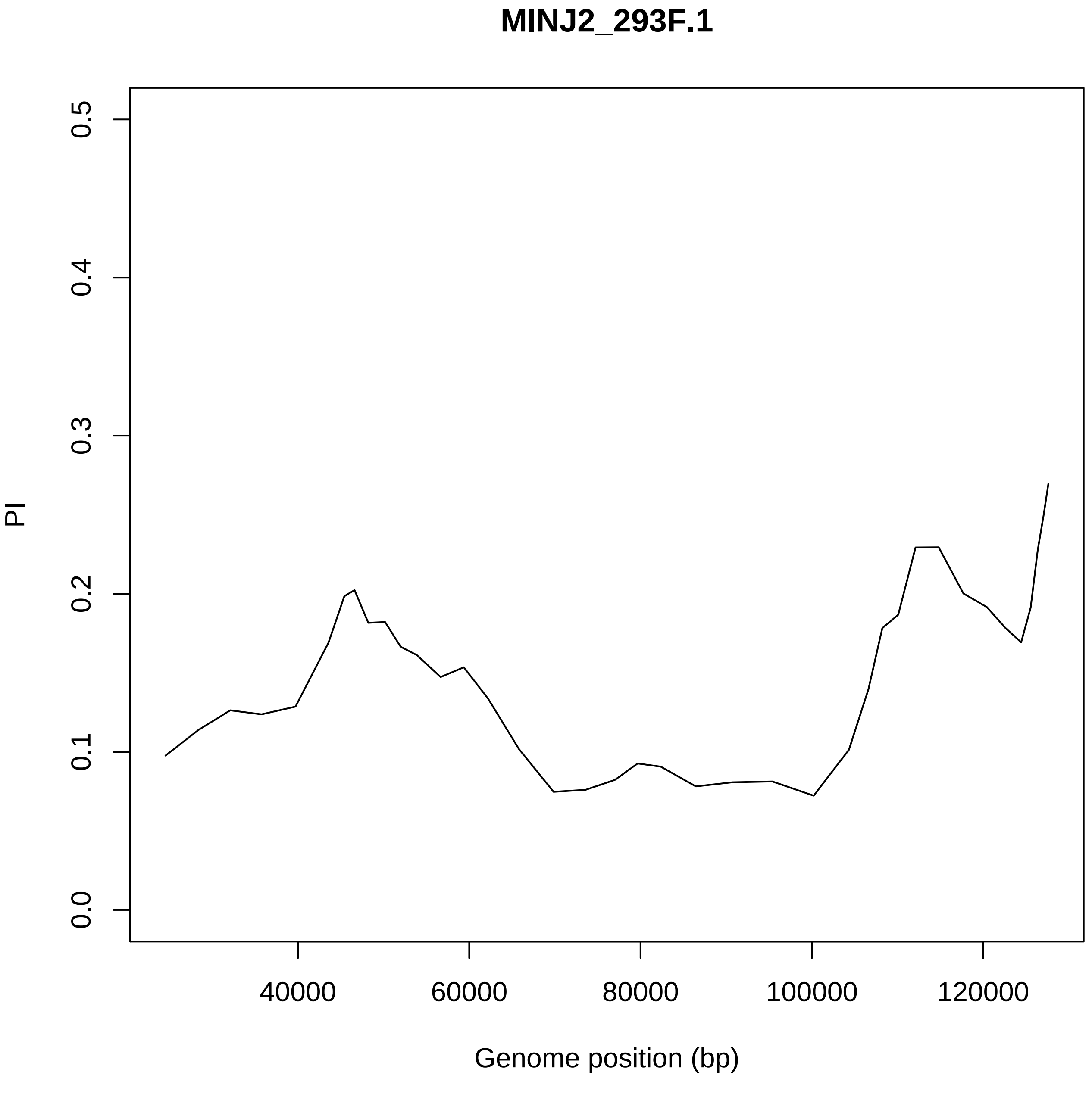
MINJ2_293F.1
PI
0.0
0.1
0.2
0.3
0.4
0.5
40000
60000
80000
100000
120000
Genome position (bp)

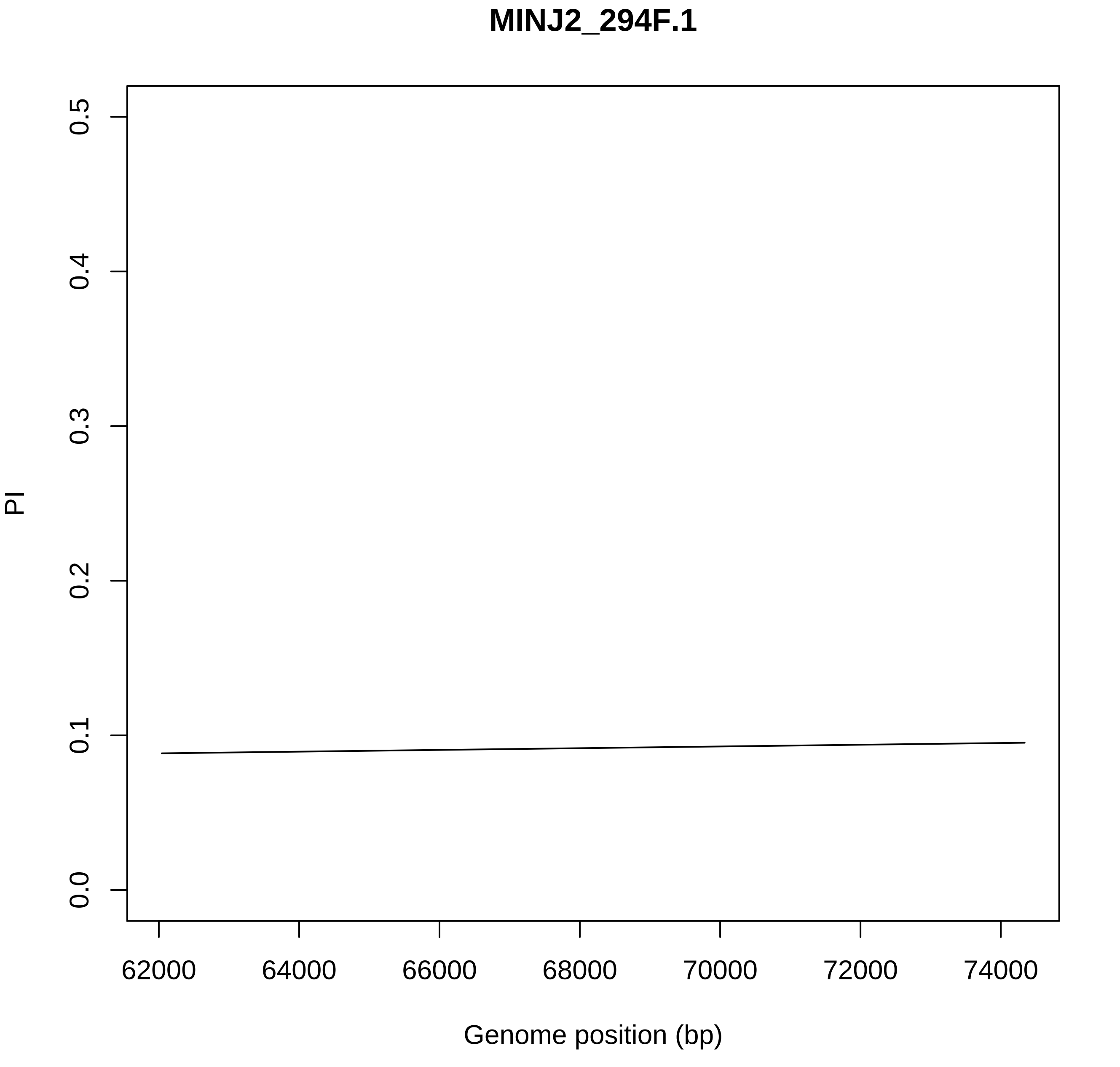
MINJ2_294F.1
PI
0.0
0.1
0.2
0.3
0.4
0.5
62000
64000
66000
68000
70000
72000
74000
Genome position (bp)

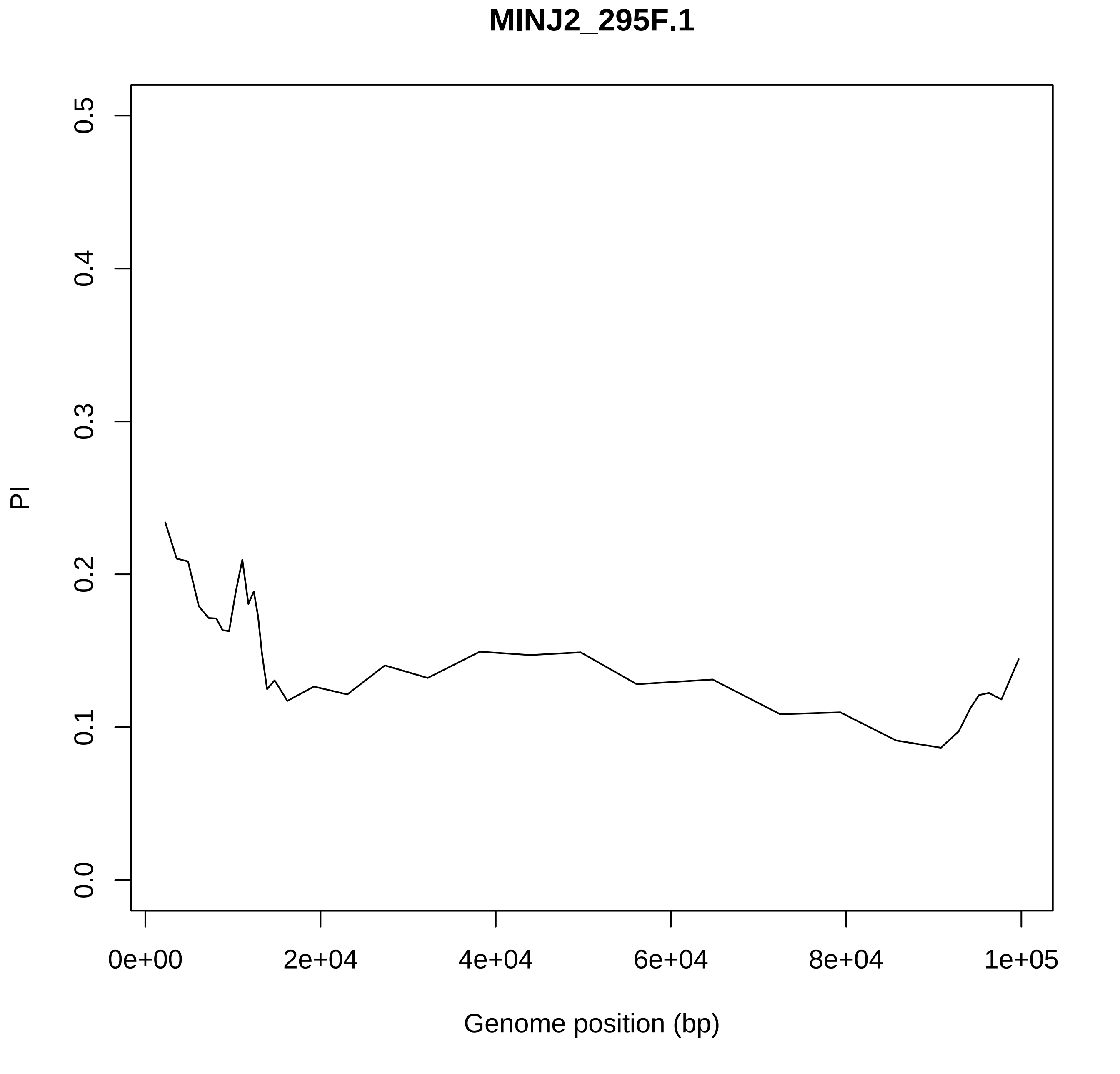
MINJ2_295F.1
PI
0.0
0.1
0.2
0.3
0.4
0.5
0e+00
2e+04
4e+04
6e+04
8e+04
1e+05
Genome position (bp)

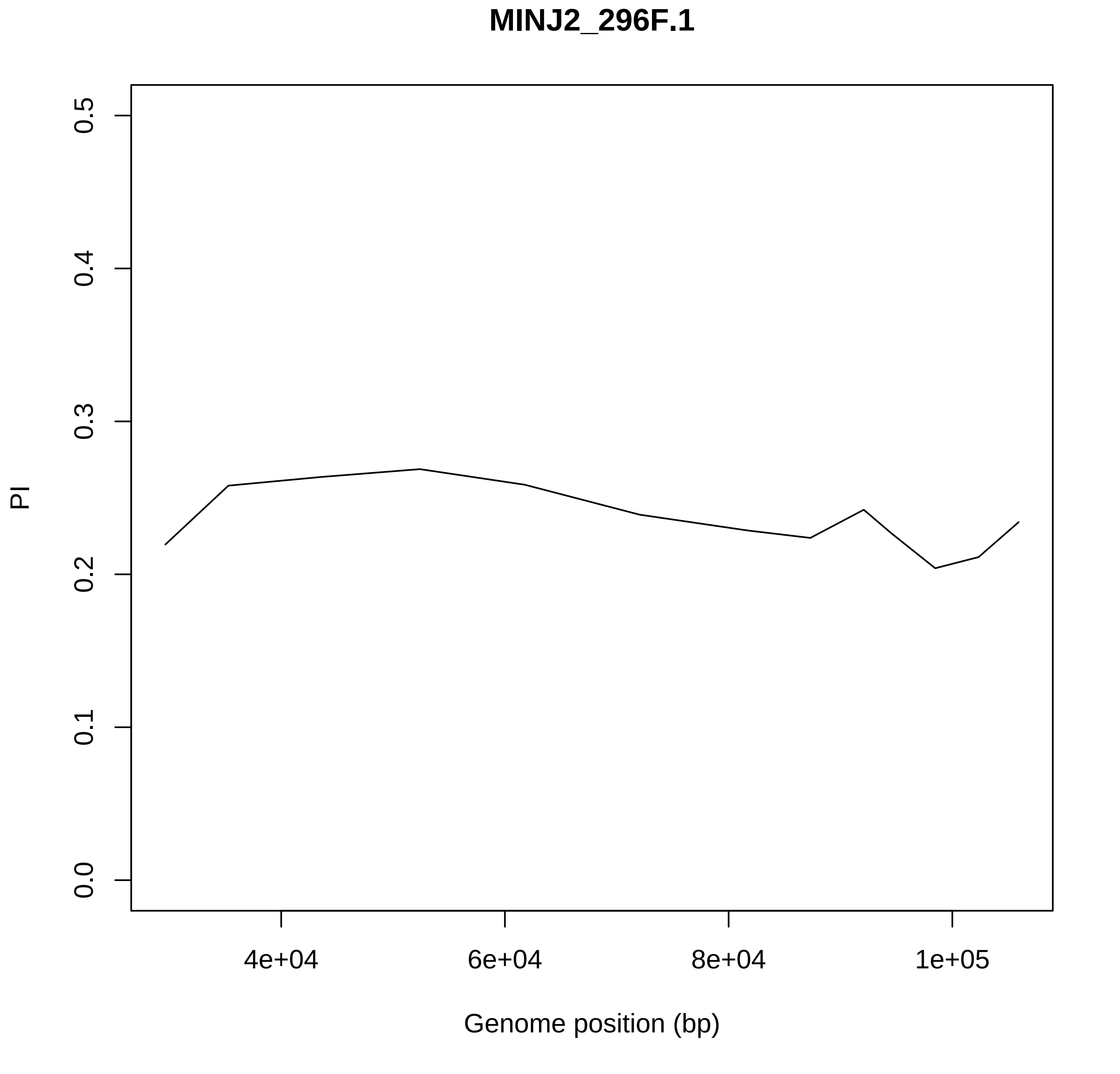

MINJ2_296F.1
0.0
0.1
0.2
0.3
0.4
0.5
PI
4e+04
6e+04
8e+04
1e+05
Genome position (bp)

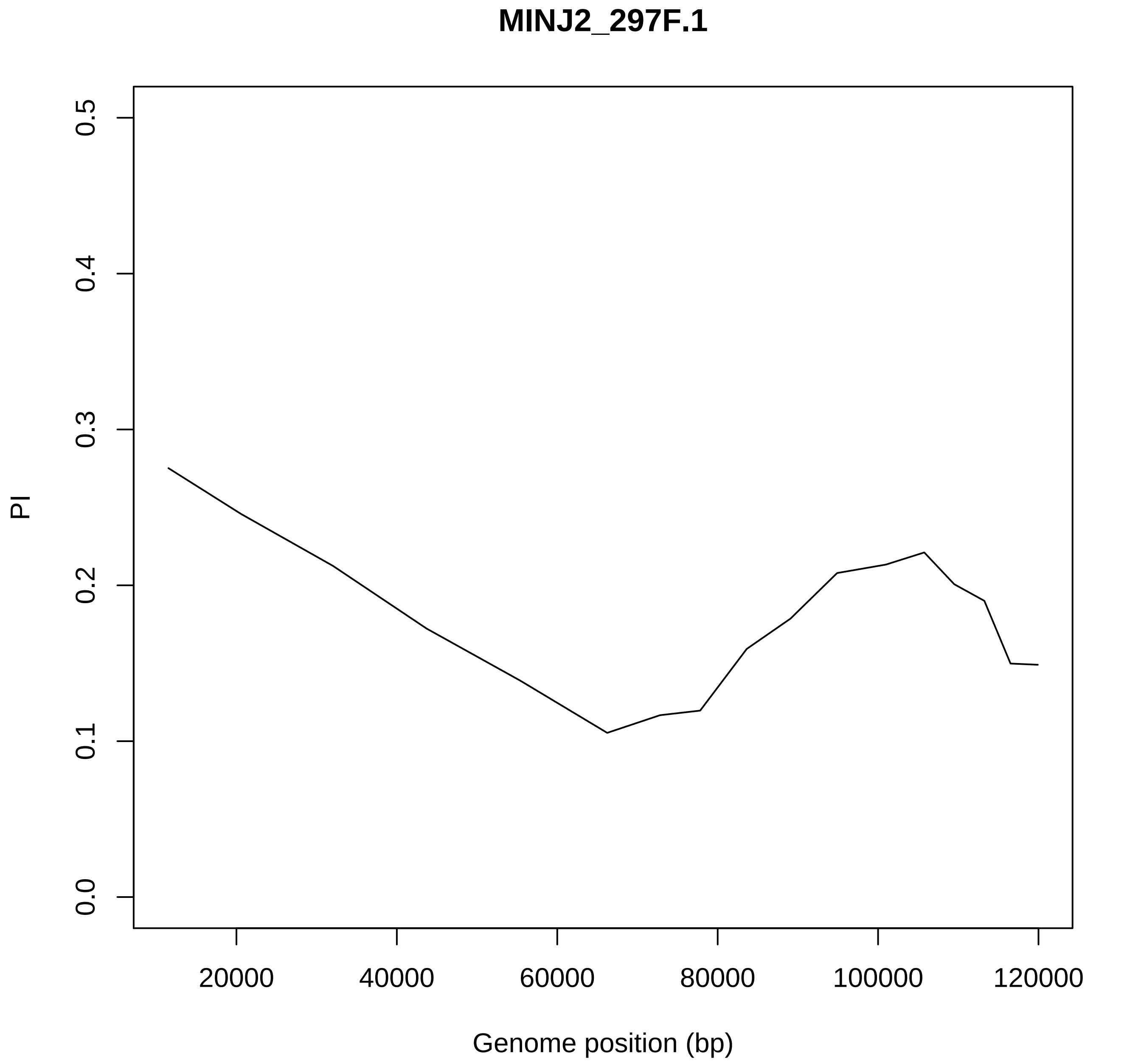
MINJ2_297F.1
PI
0.0
0.1
0.2
0.3
0.4
0.5
20000
40000
60000
80000
100000
120000
Genome position (bp)

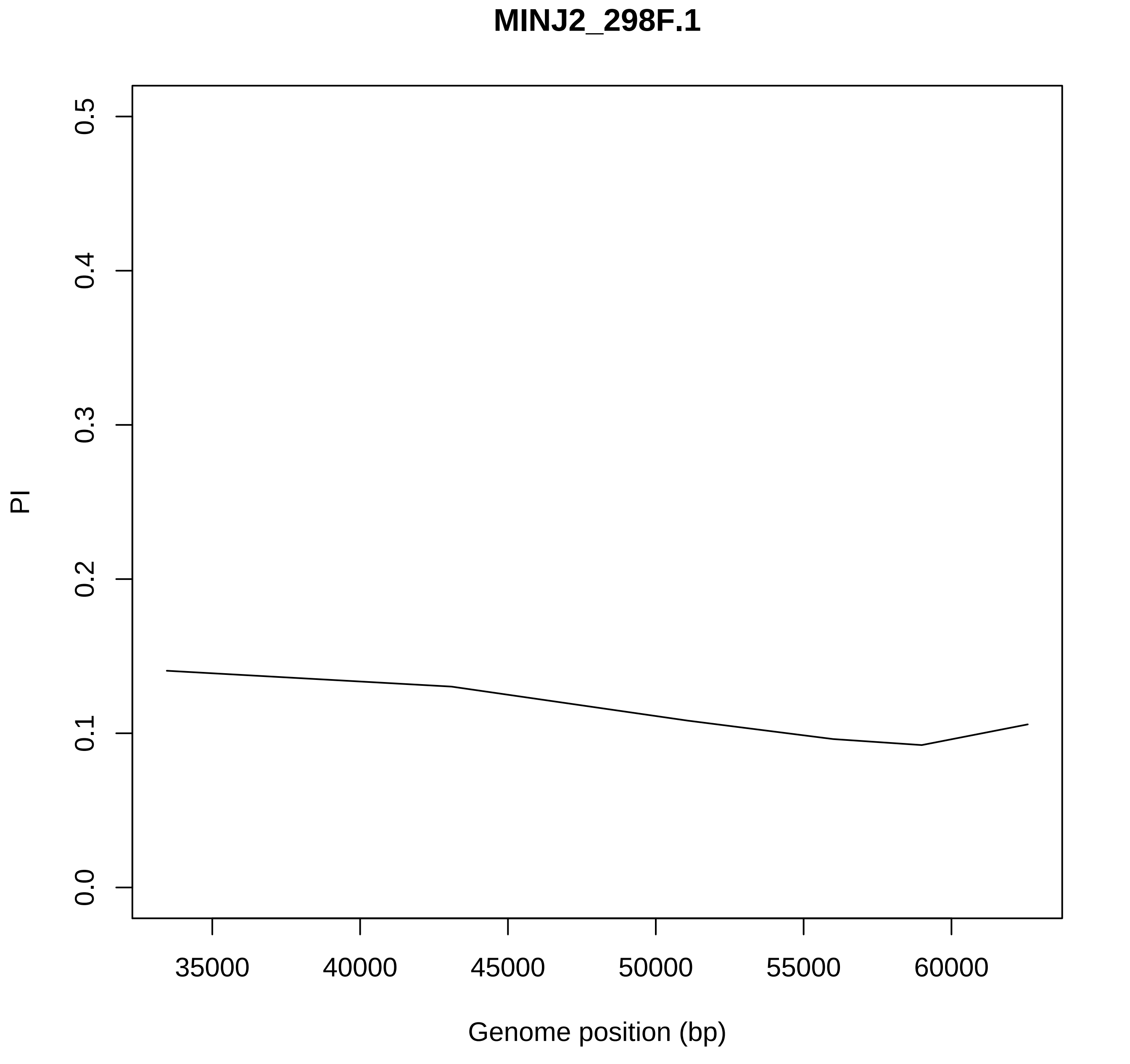

MINJ2_298F.1
PI
0.0
0.1
0.2
0.3
0.4
0.5
35000
40000
45000
50000
55000
60000
Genome position (bp)

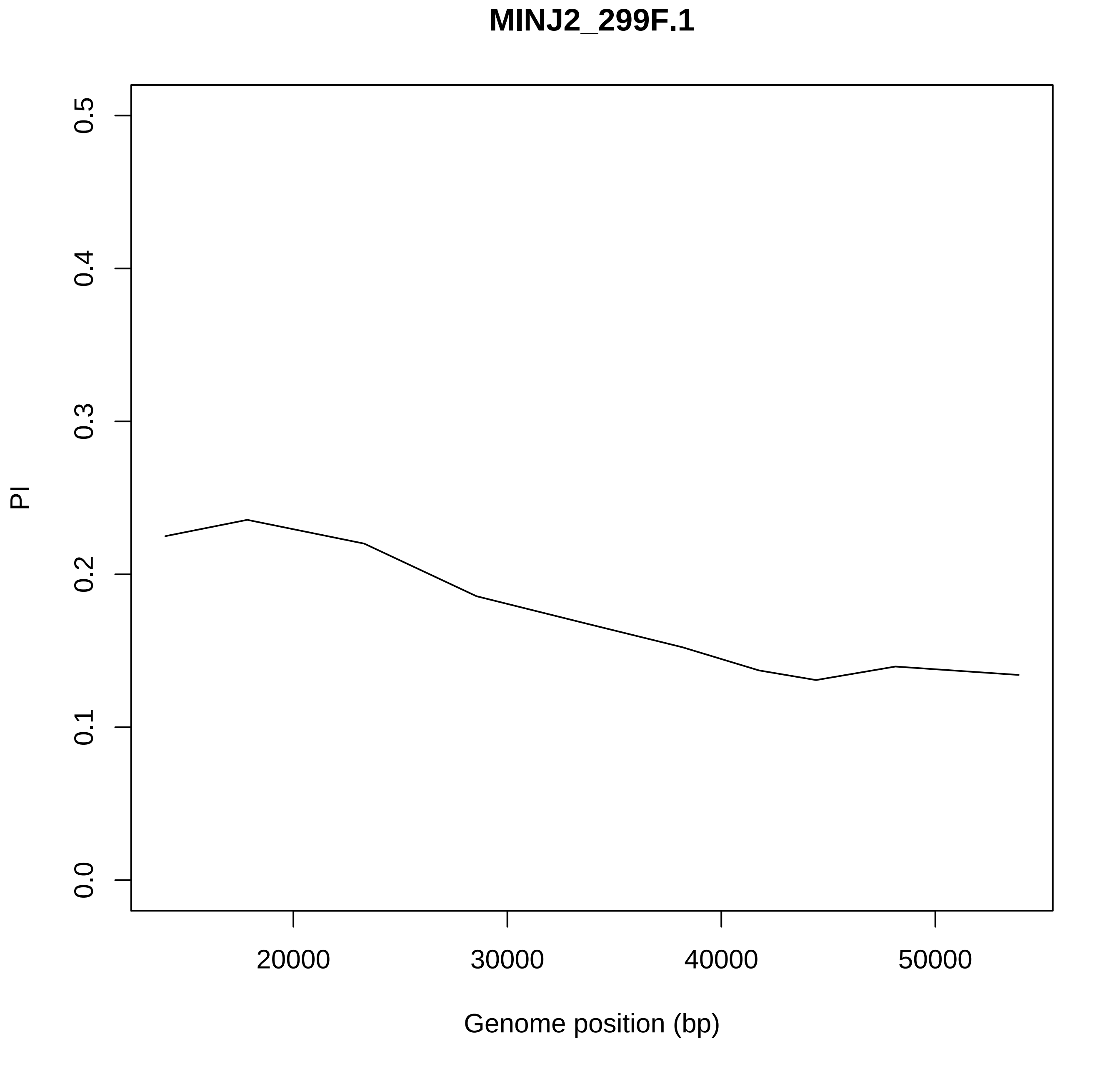

MINJ2_299F.1
PI
0.0
0.1
0.2
0.3
0.4
0.5
20000
30000
40000
50000
Genome position (bp)

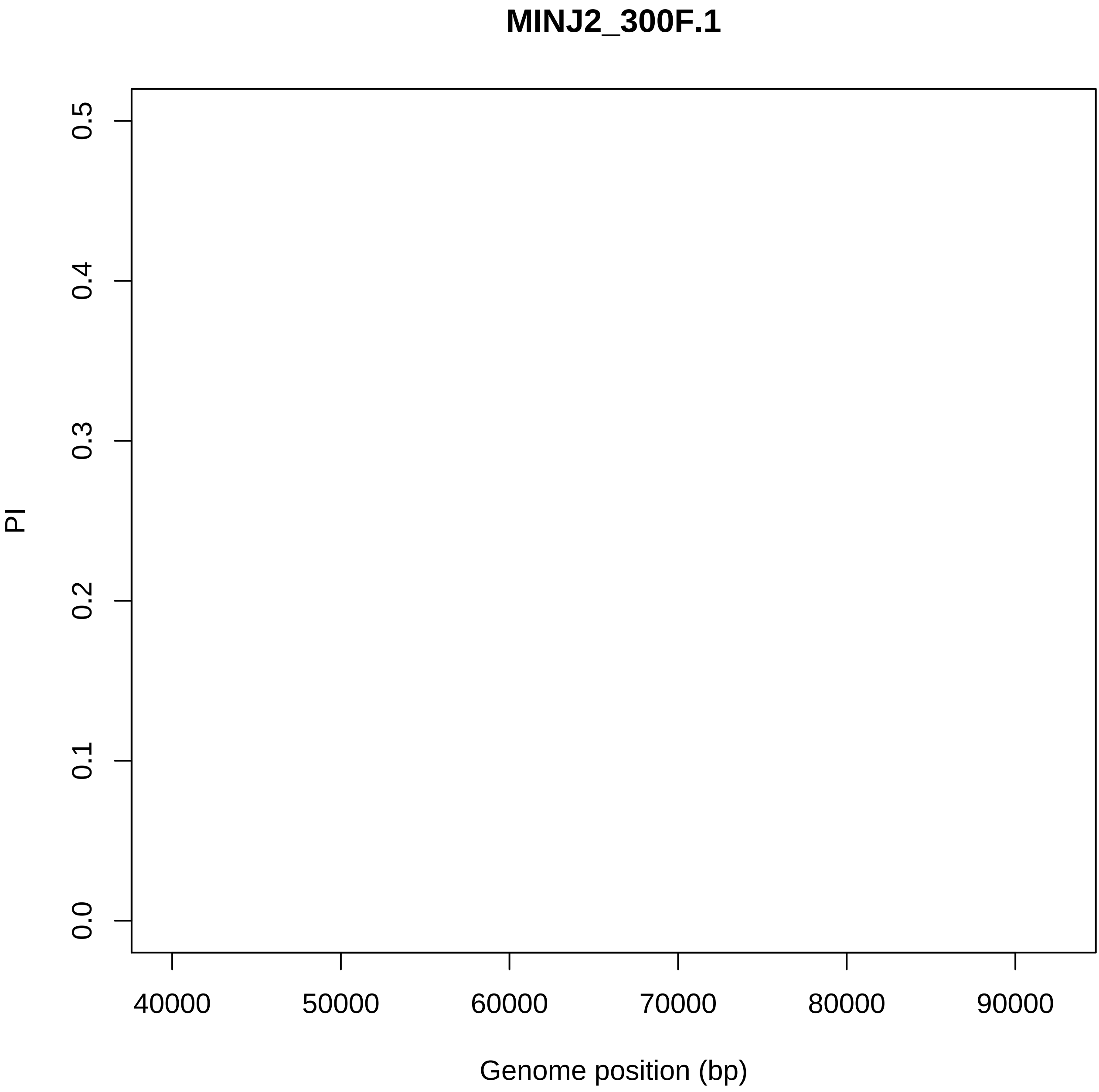

MINJ2_300F.1
PI
0.0
0.1
0.2
0.3
0.4
0.5
40000
50000
60000
70000
80000
90000
Genome position (bp)

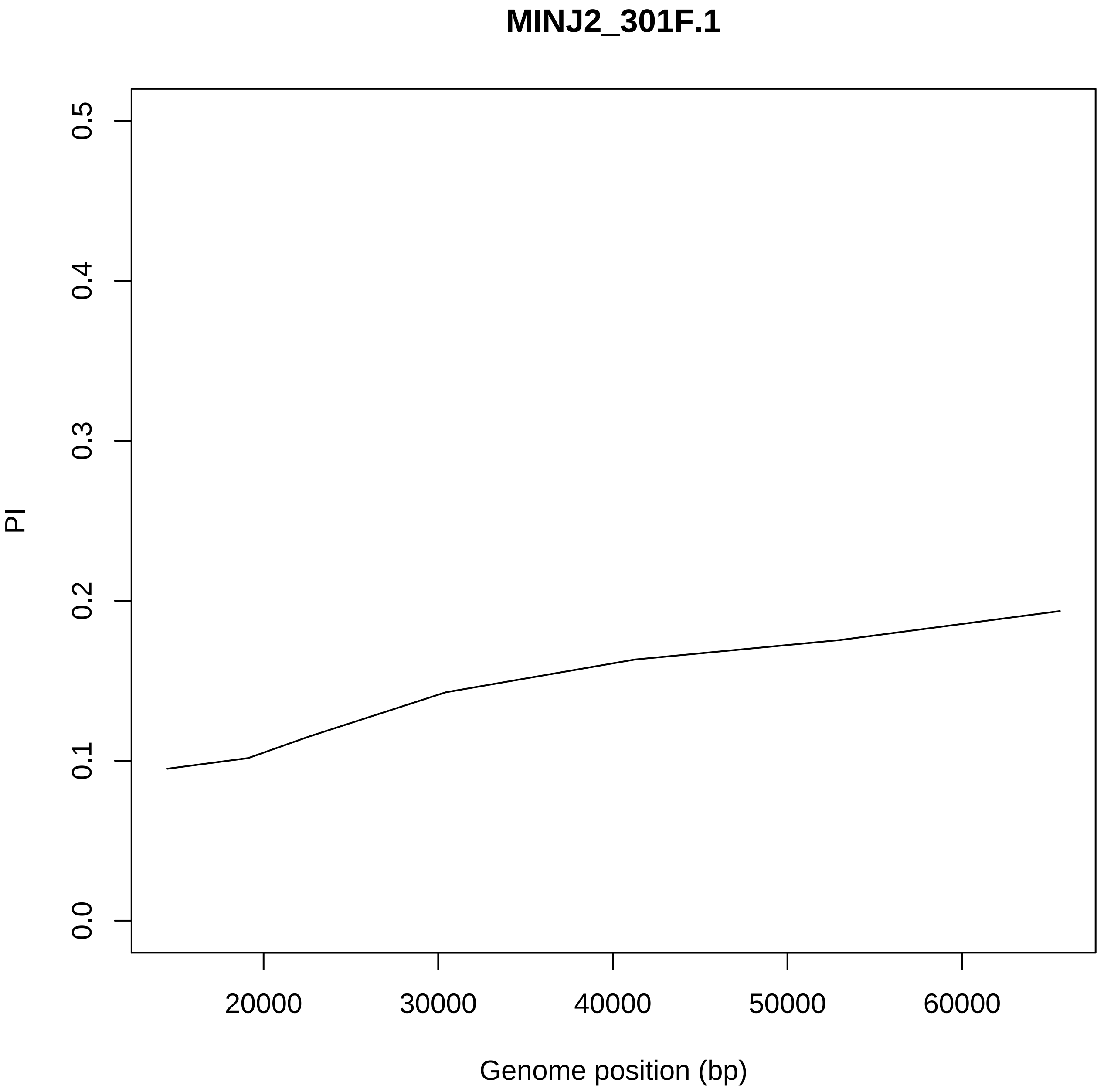

MINJ2_301F.1
PI
0.0
0.1
0.2
0.3
0.4
0.5
20000
30000
40000
50000
60000
Genome position (bp)

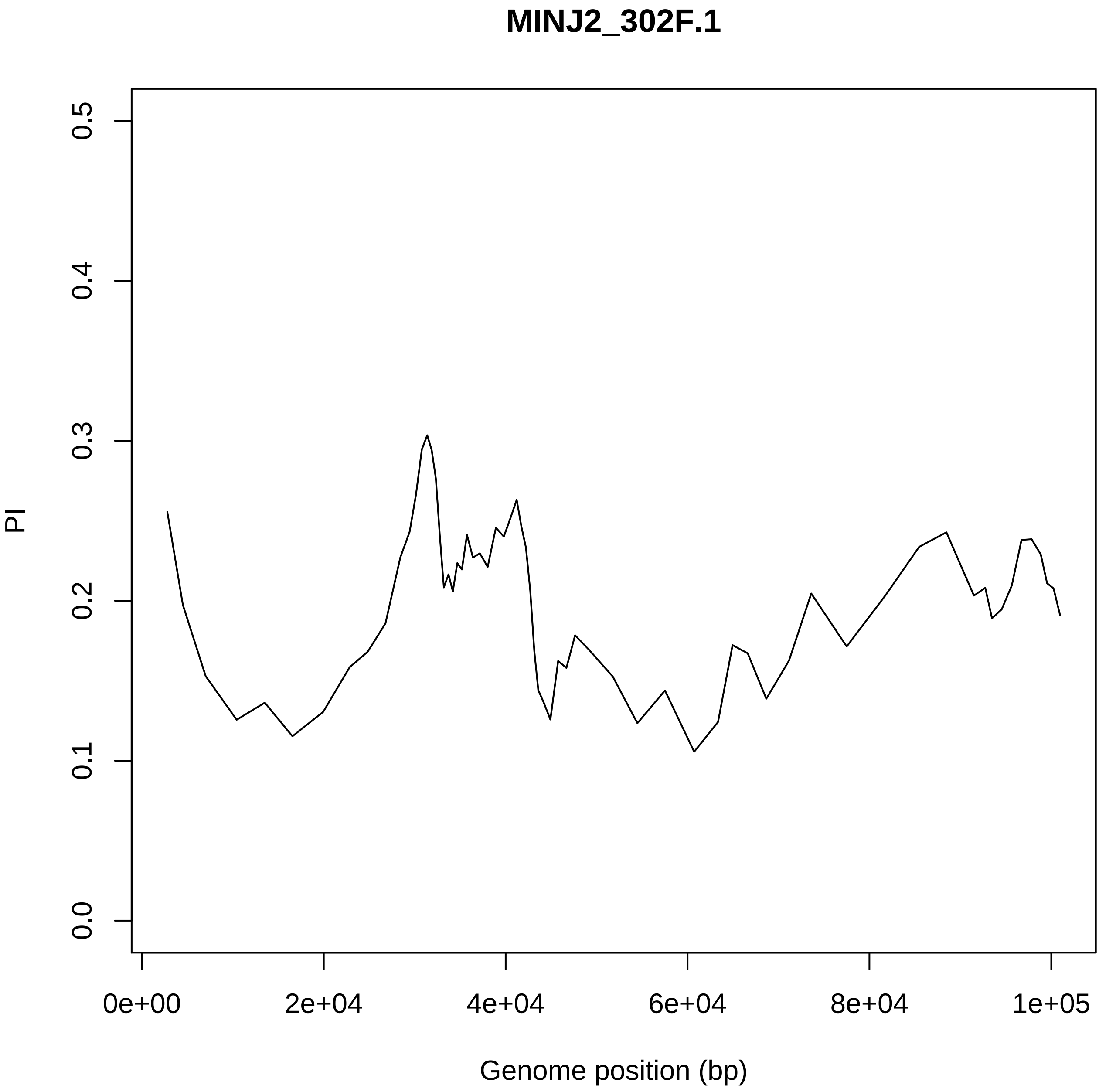
MINJ2_302F.1
PI
0.0
0.1
0.2
0.3
0.4
0.5
0e+00
2e+04
4e+04
6e+04
8e+04
1e+05
Genome position (bp)

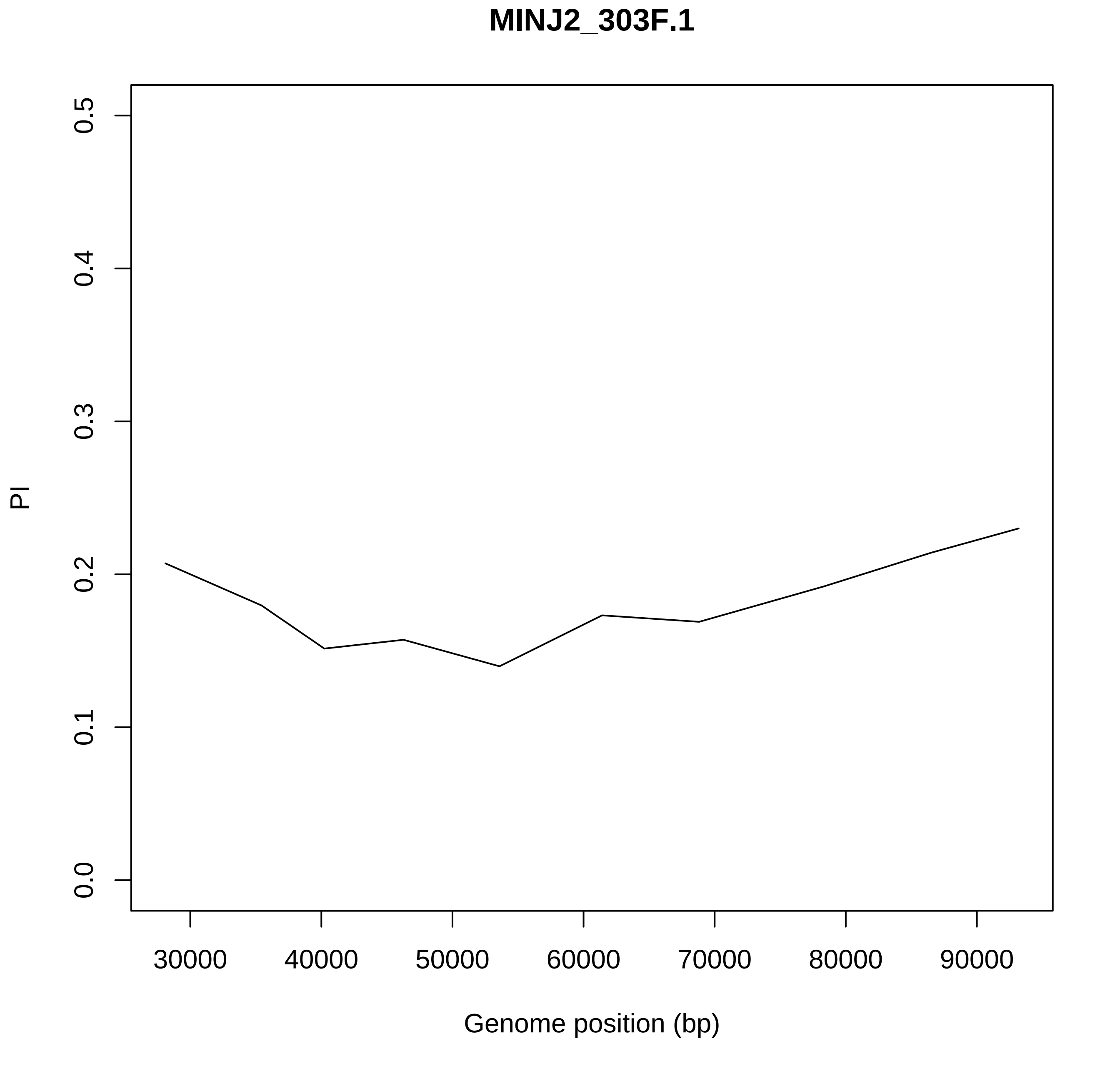
MINJ2_303F.1
PI
0.0
0.1
0.2
0.3
0.4
0.5
30000
40000
50000
60000
70000
80000
90000
Genome position (bp)

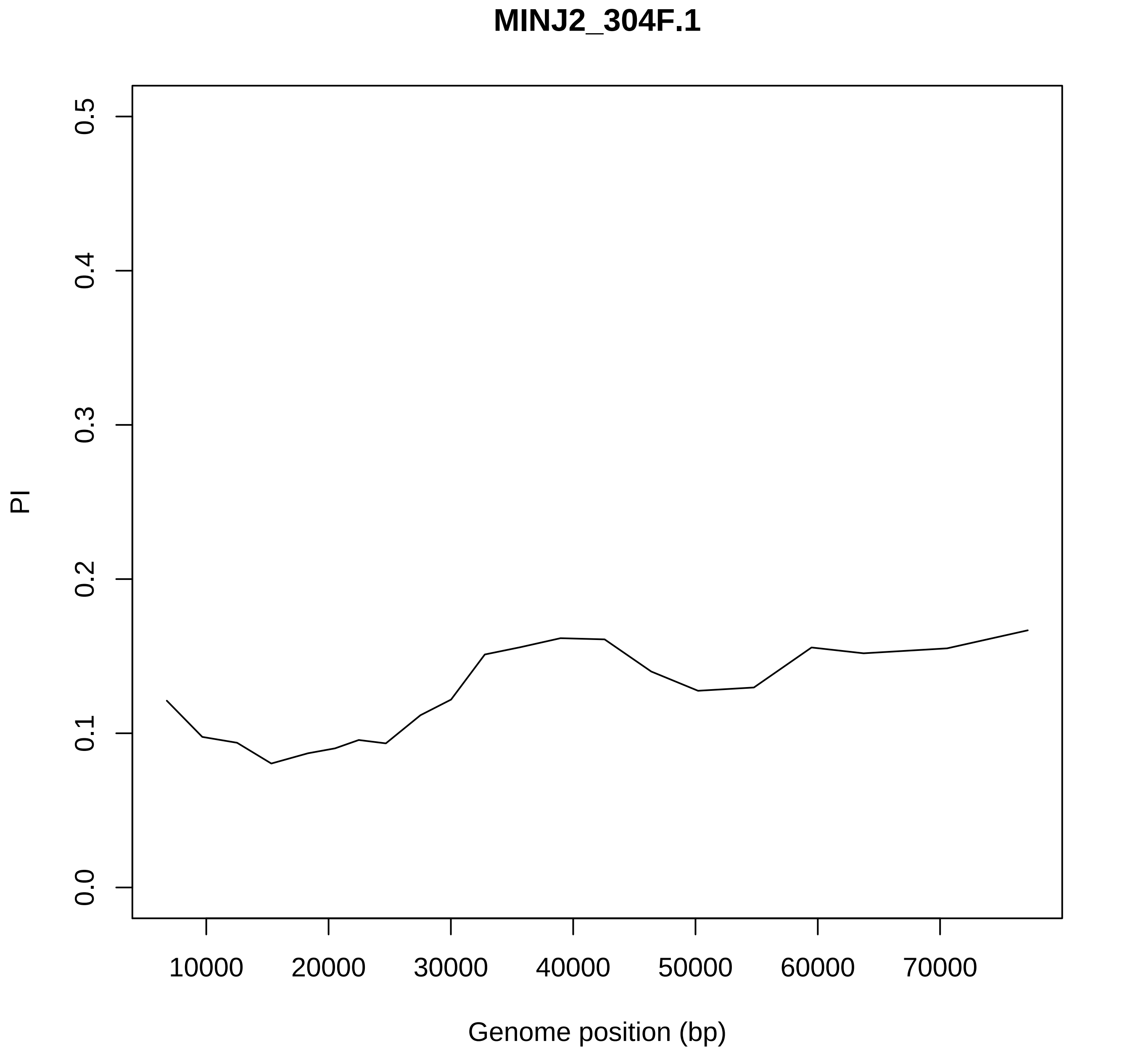
MINJ2_304F.1
PI
0.0
0.1
0.2
0.3
0.4
0.5
10000
20000
30000
40000
50000
60000
70000
Genome position (bp)

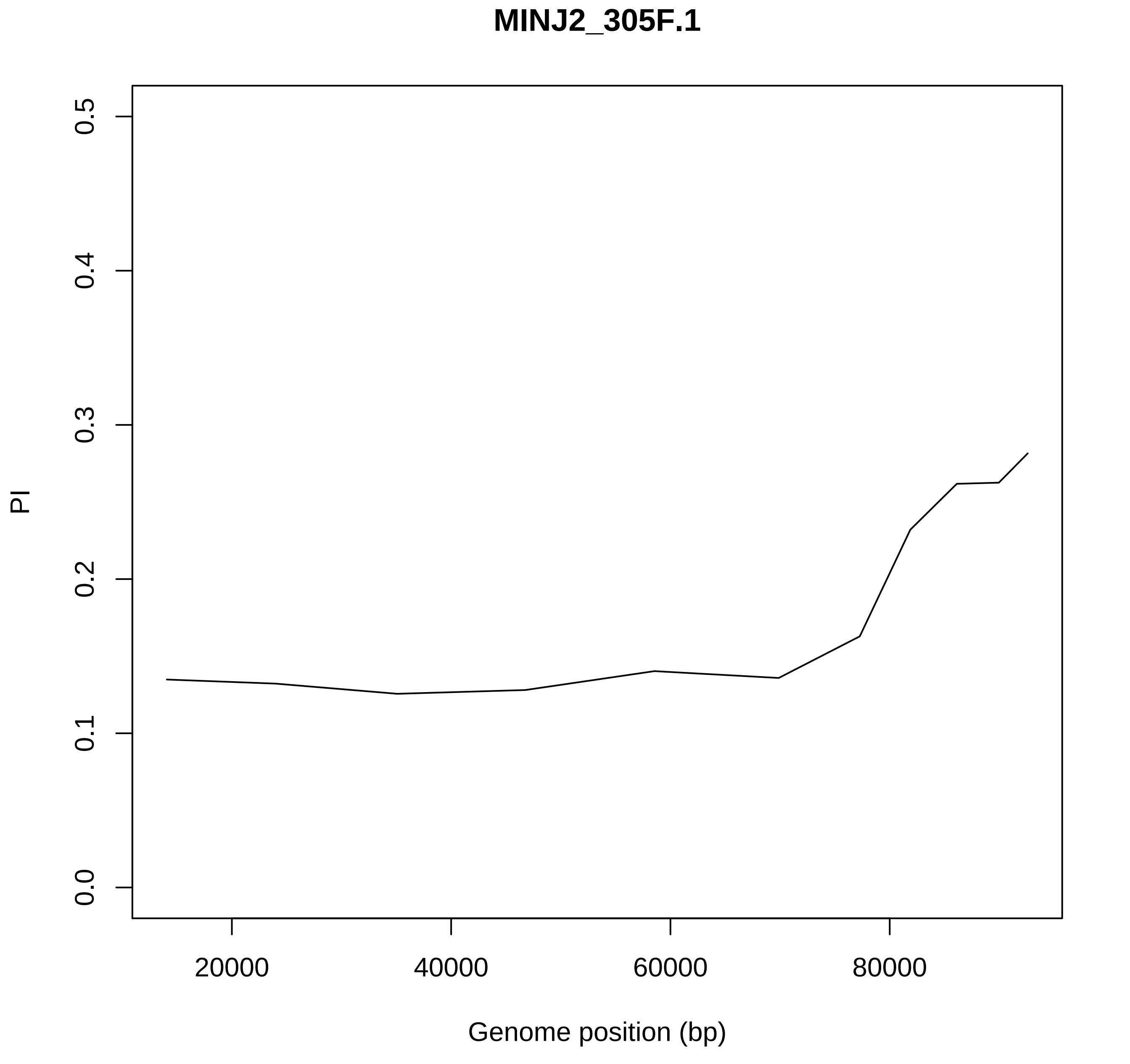
MINJ2_305F.1
PI
0.0
0.1
0.2
0.3
0.4
0.5
20000
40000
60000
80000
Genome position (bp)

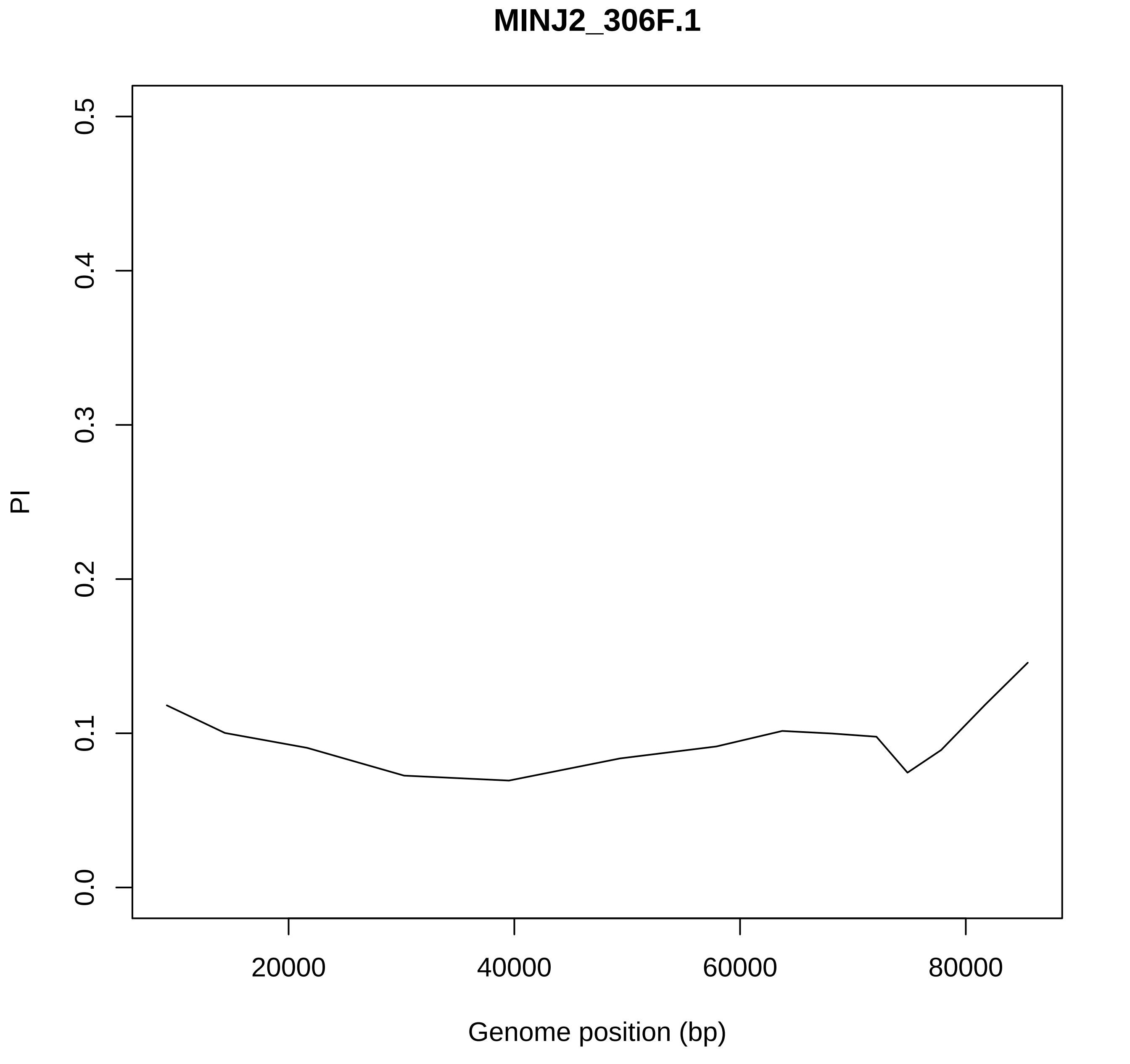

MINJ2_306F.1
PI
0.0
0.1
0.2
0.3
0.4
0.5
20000
40000
60000
80000
Genome position (bp)

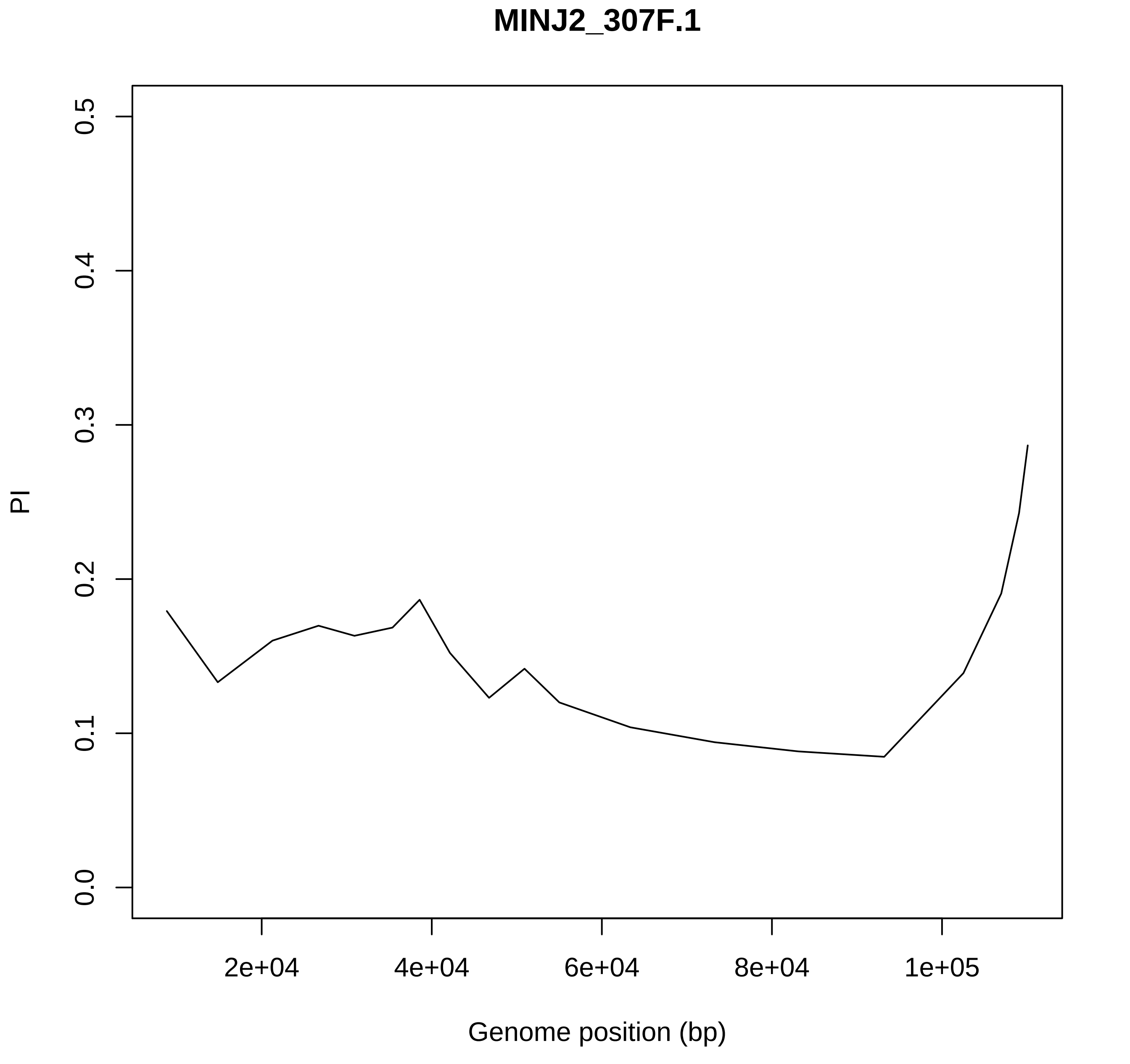

MINJ2_307F.1
PI
0.0
0.1
0.2
0.3
0.4
0.5
2e+04
4e+04
6e+04
8e+04
1e+05
Genome position (bp)

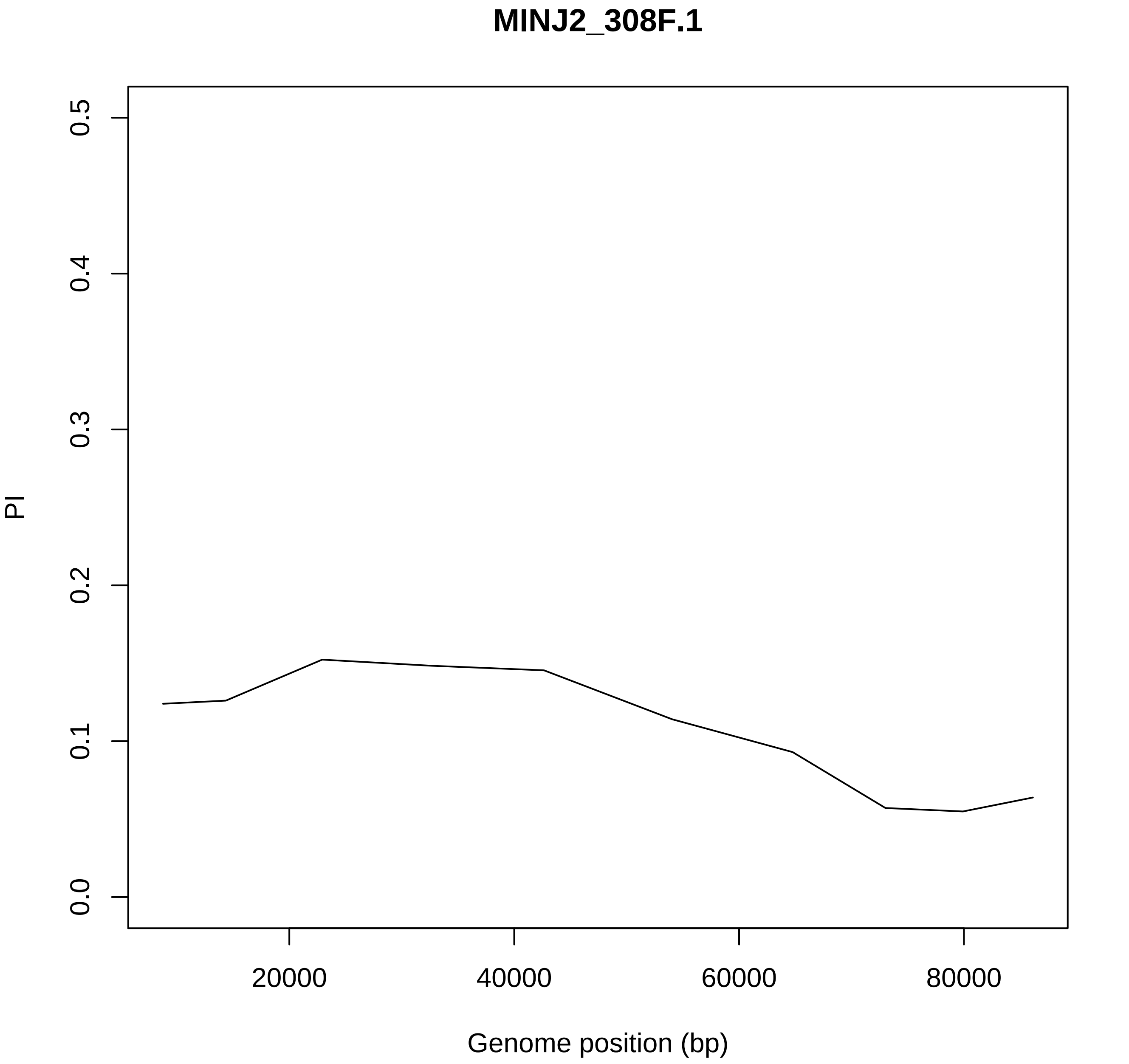
MINJ2_308F.1
PI
0.0
0.1
0.2
0.3
0.4
0.5
20000
40000
60000
80000
Genome position (bp)

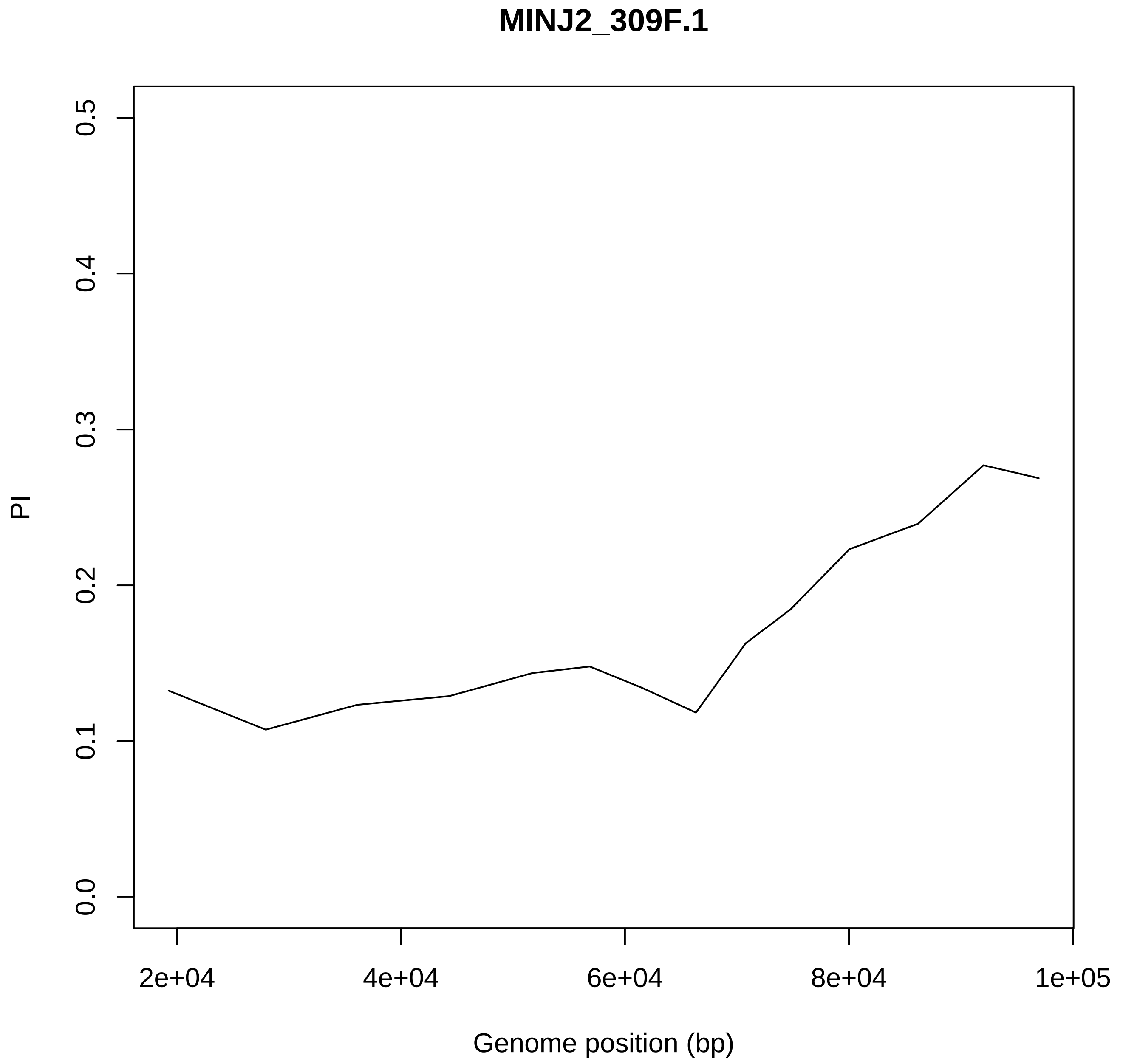

MINJ2_309F.1
PI
0.0
0.1
0.2
0.3
0.4
0.5
2e+04
4e+04
6e+04
8e+04
1e+05
Genome position (bp)

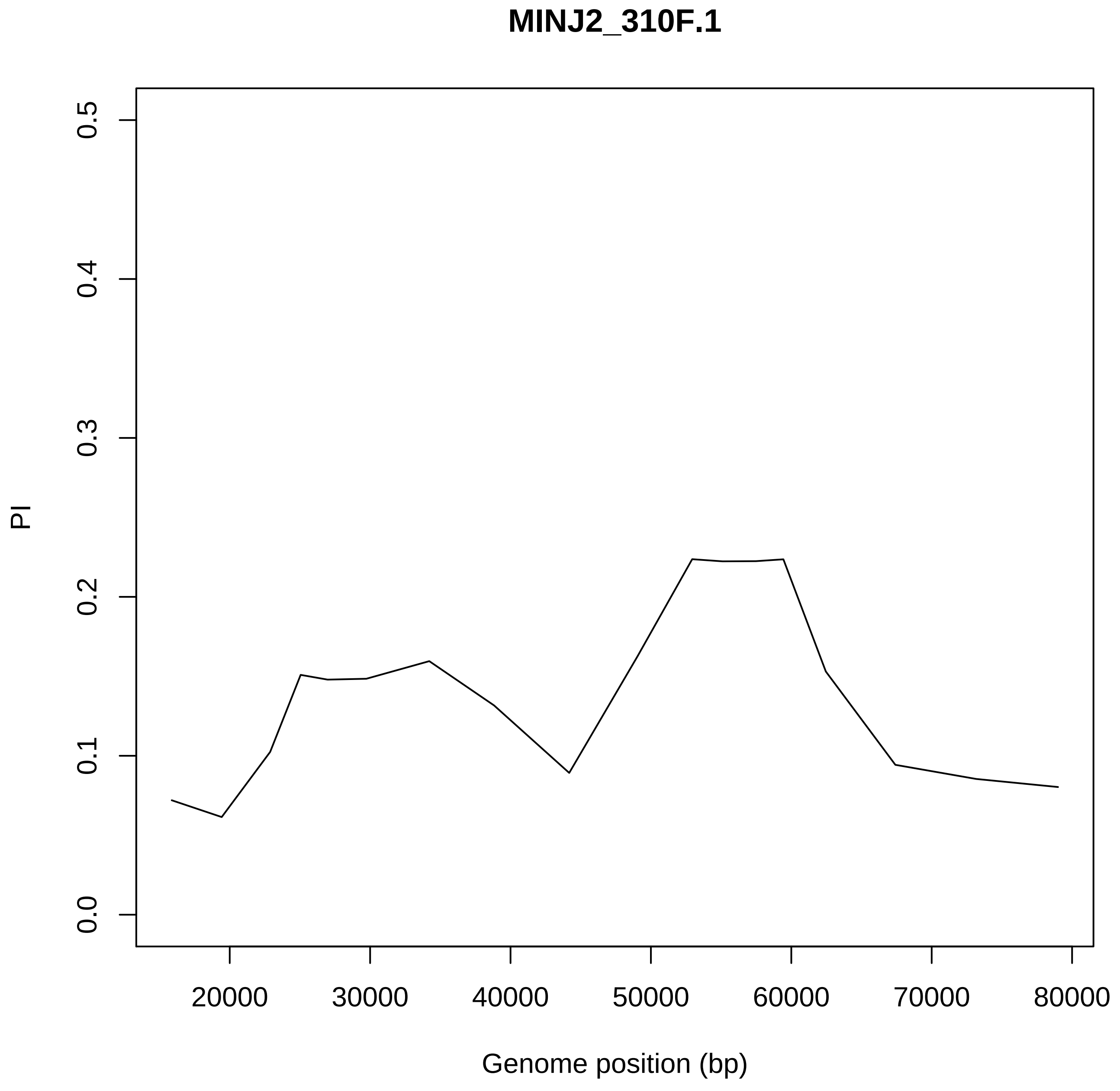

MINJ2_310F.1
PI
0.0
0.1
0.2
0.3
0.4
0.5
20000
30000
40000
50000
60000
70000
80000
Genome position (bp)

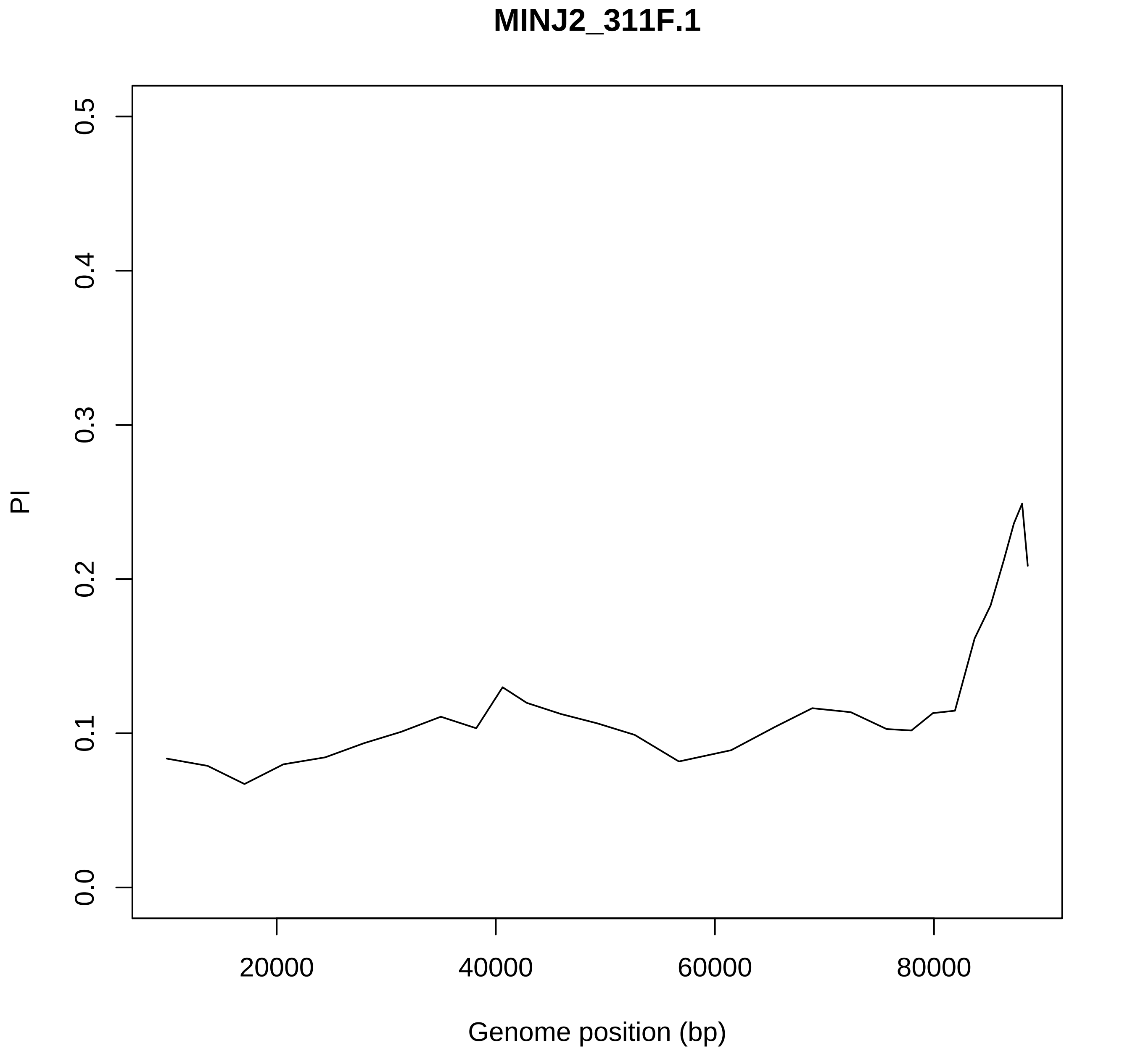

MINJ2_311F.1
PI
0.0
0.1
0.2
0.3
0.4
0.5
20000
40000
60000
80000
Genome position (bp)

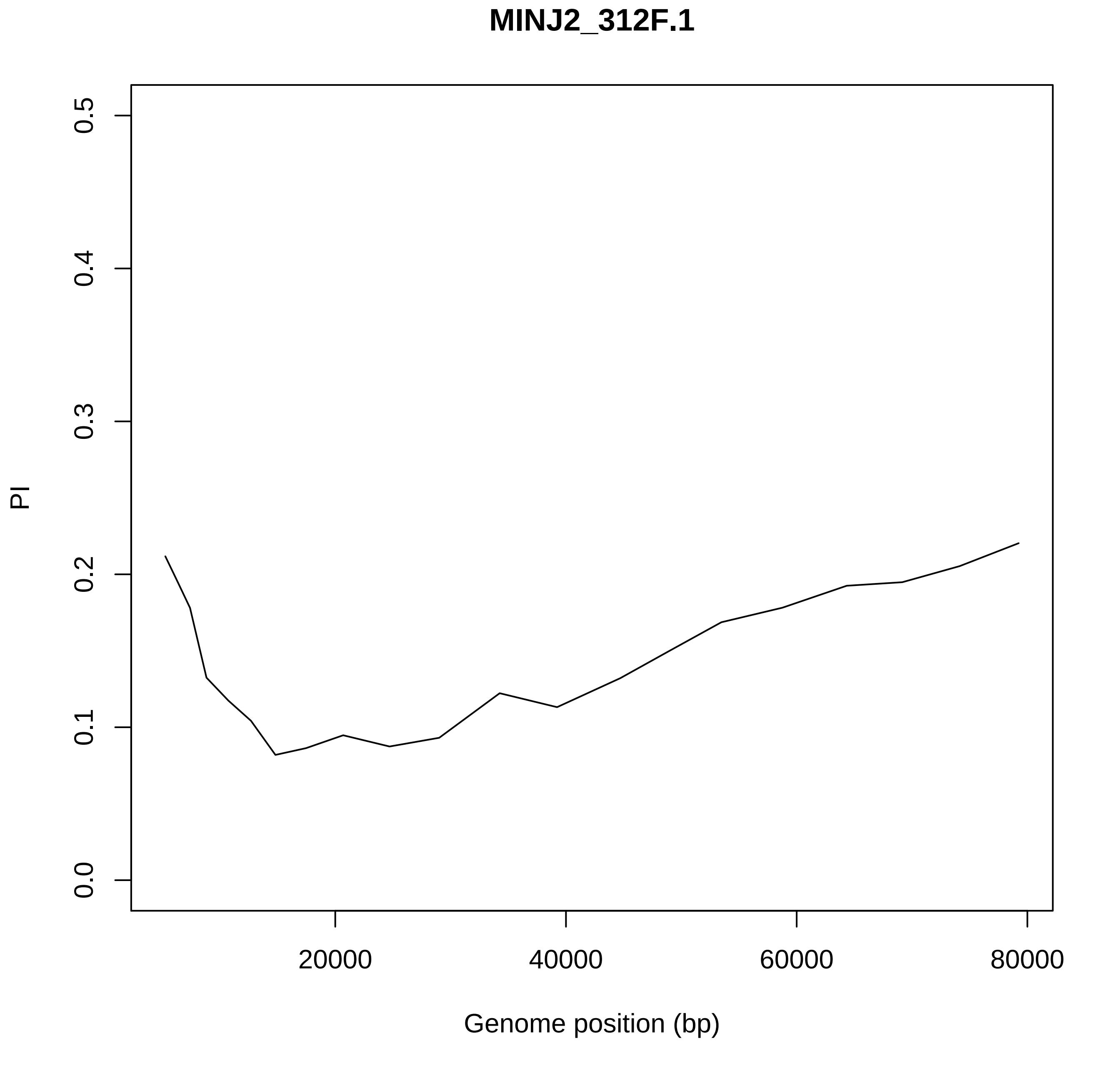

MINJ2_312F.1
PI
0.0
0.1
0.2
0.3
0.4
0.5
20000
40000
60000
80000
Genome position (bp)

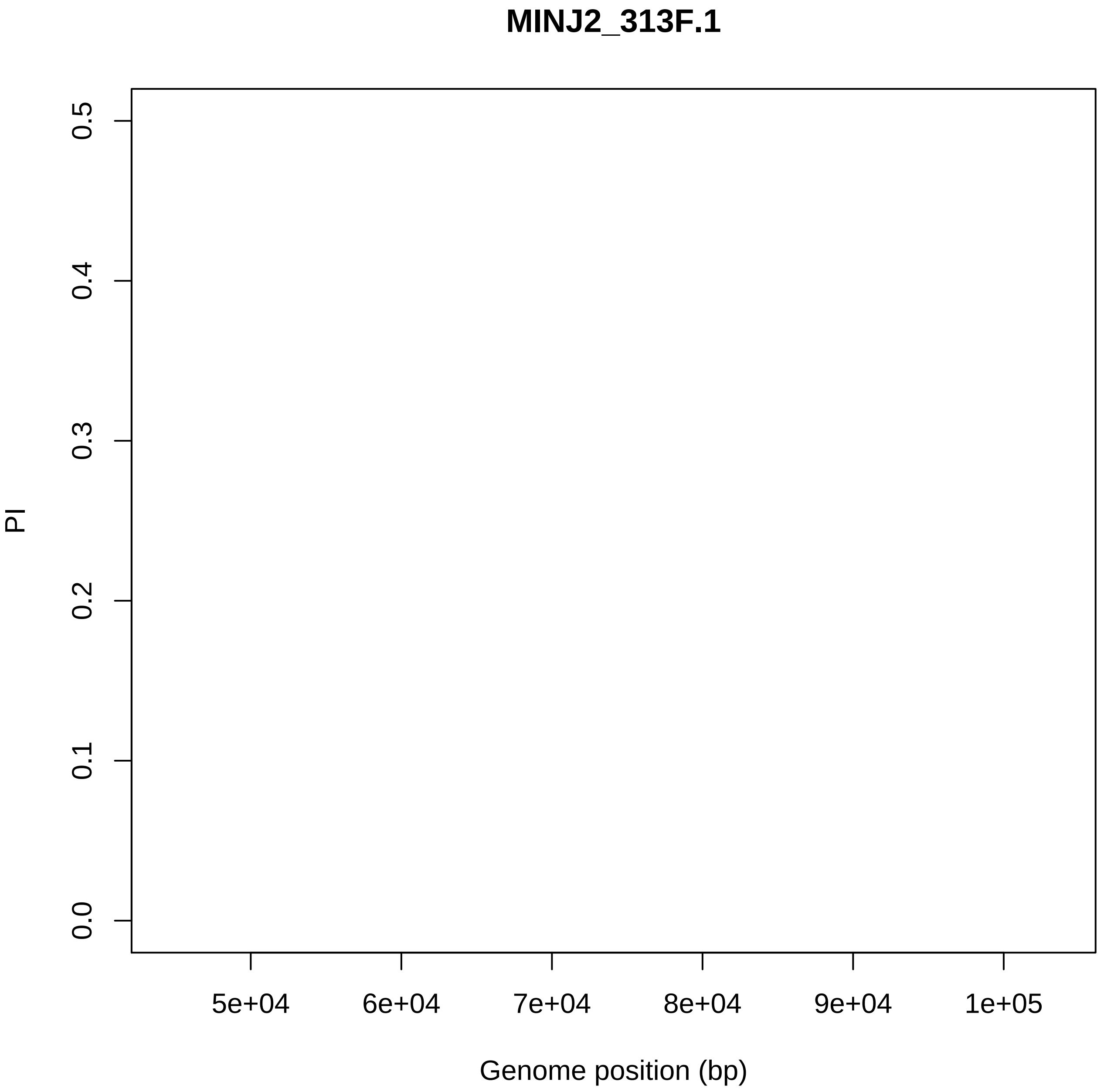
MINJ2_313F.1
PI
0.0
0.1
0.2
0.3
0.4
0.5
5e+04
6e+04
7e+04
8e+04
9e+04
1e+05
Genome position (bp)

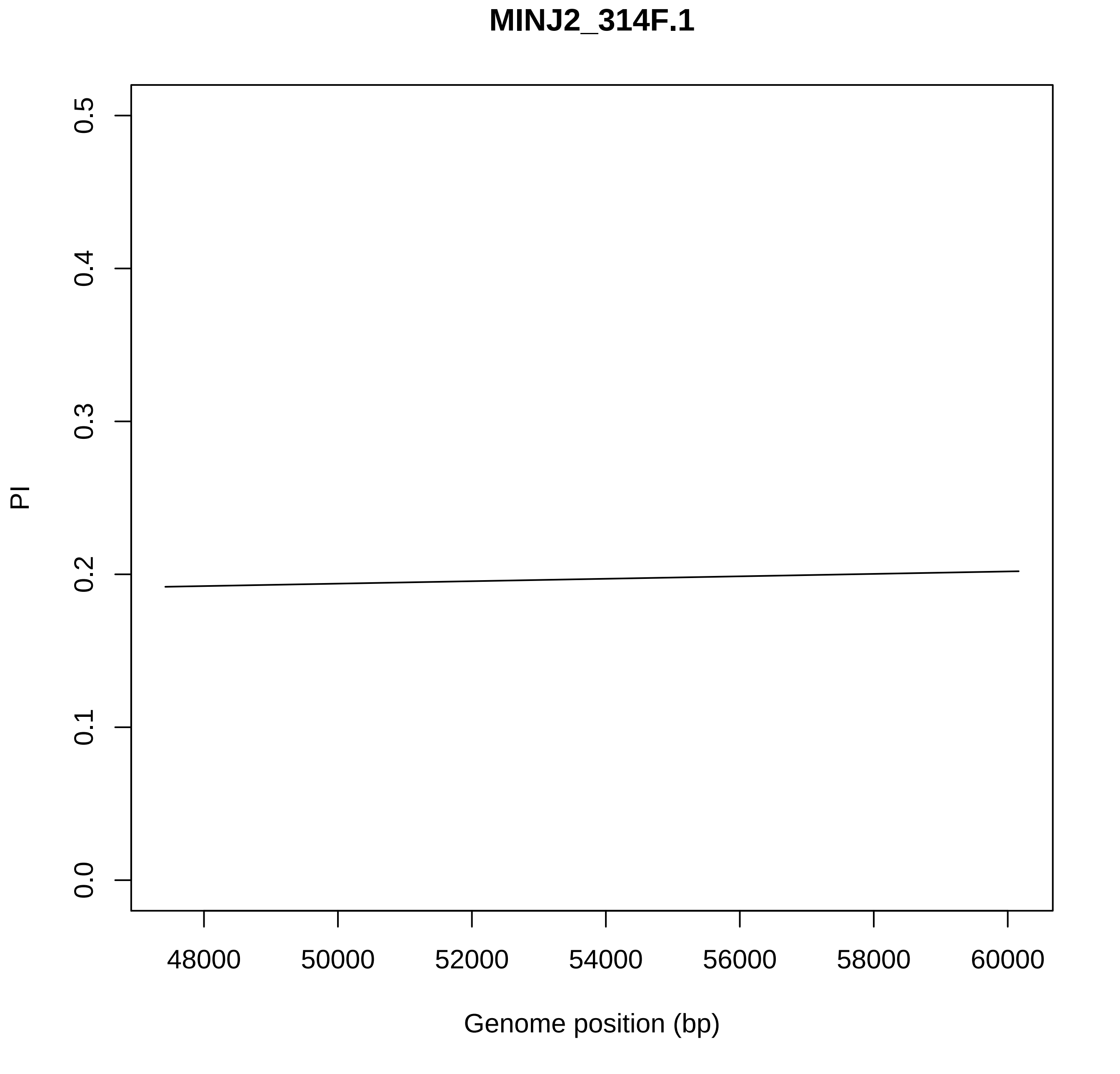
MINJ2_314F.1
PI
0.0
0.1
0.2
0.3
0.4
0.5
48000
50000
52000
54000
56000
58000
60000
Genome position (bp)

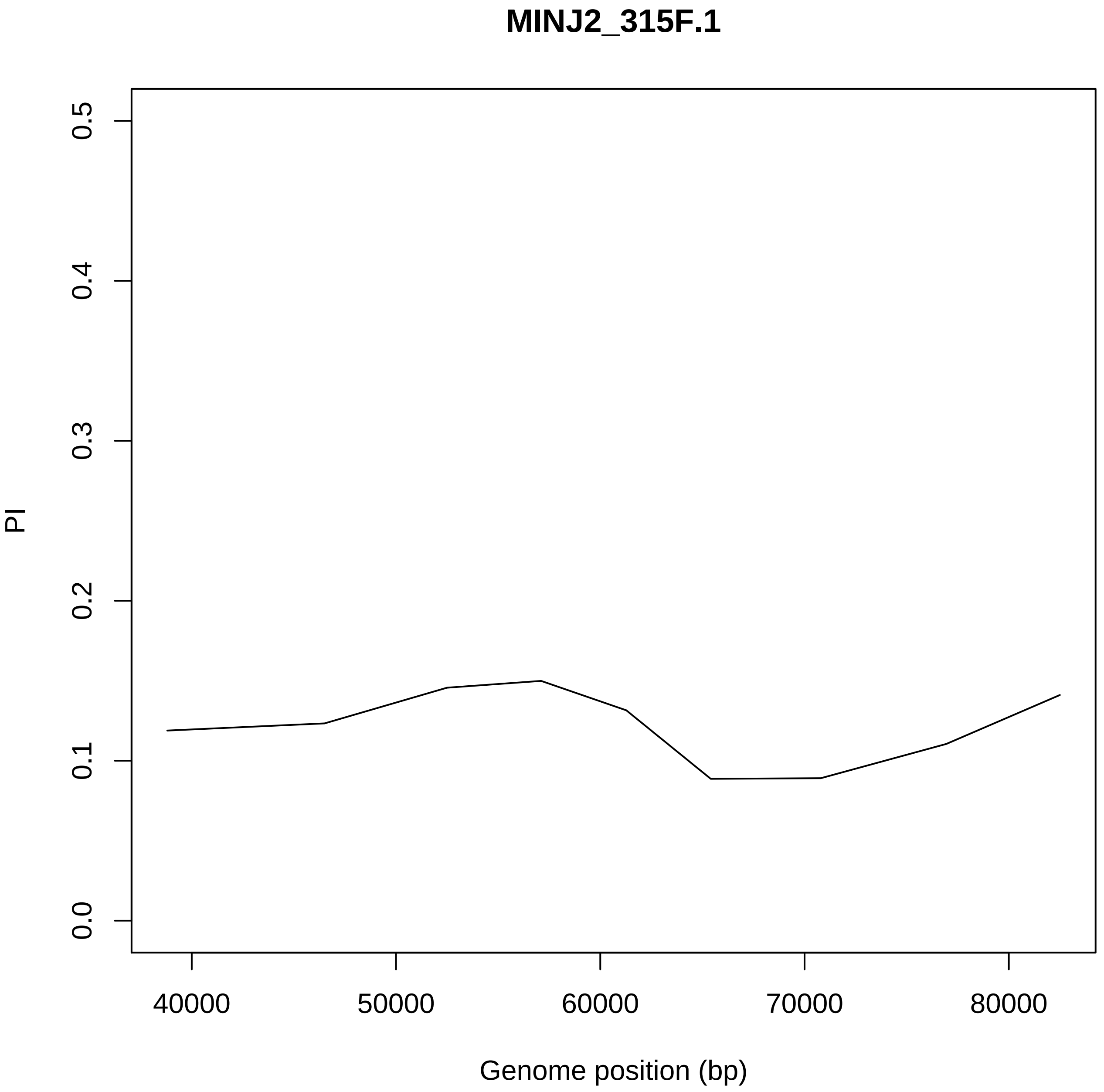
MINJ2_315F.1
PI
0.0
0.1
0.2
0.3
0.4
0.5
40000
50000
60000
70000
80000
Genome position (bp)

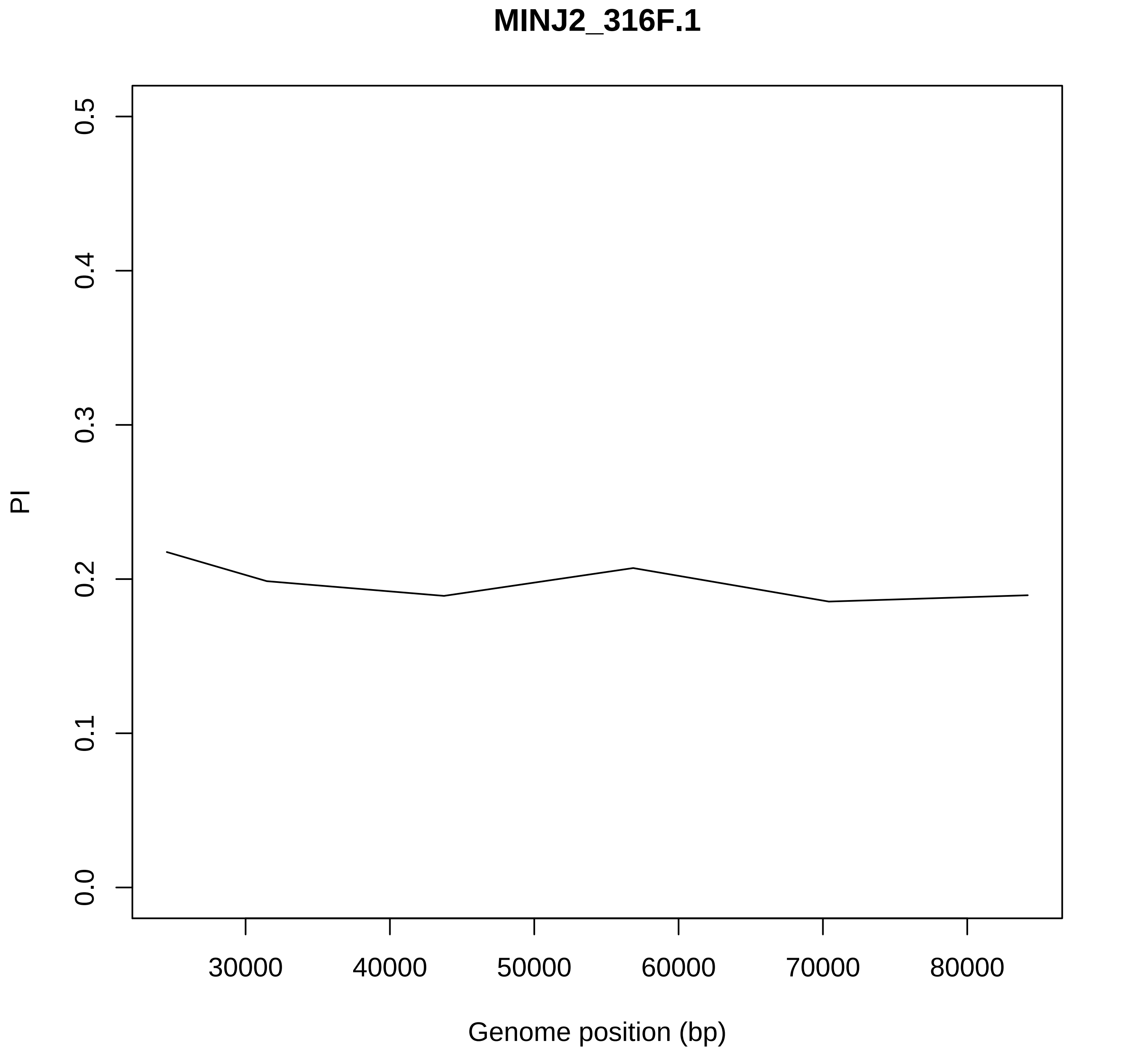

MINJ2_316F.1
PI
0.0
0.1
0.2
0.3
0.4
0.5
30000
40000
50000
60000
70000
80000
Genome position (bp)

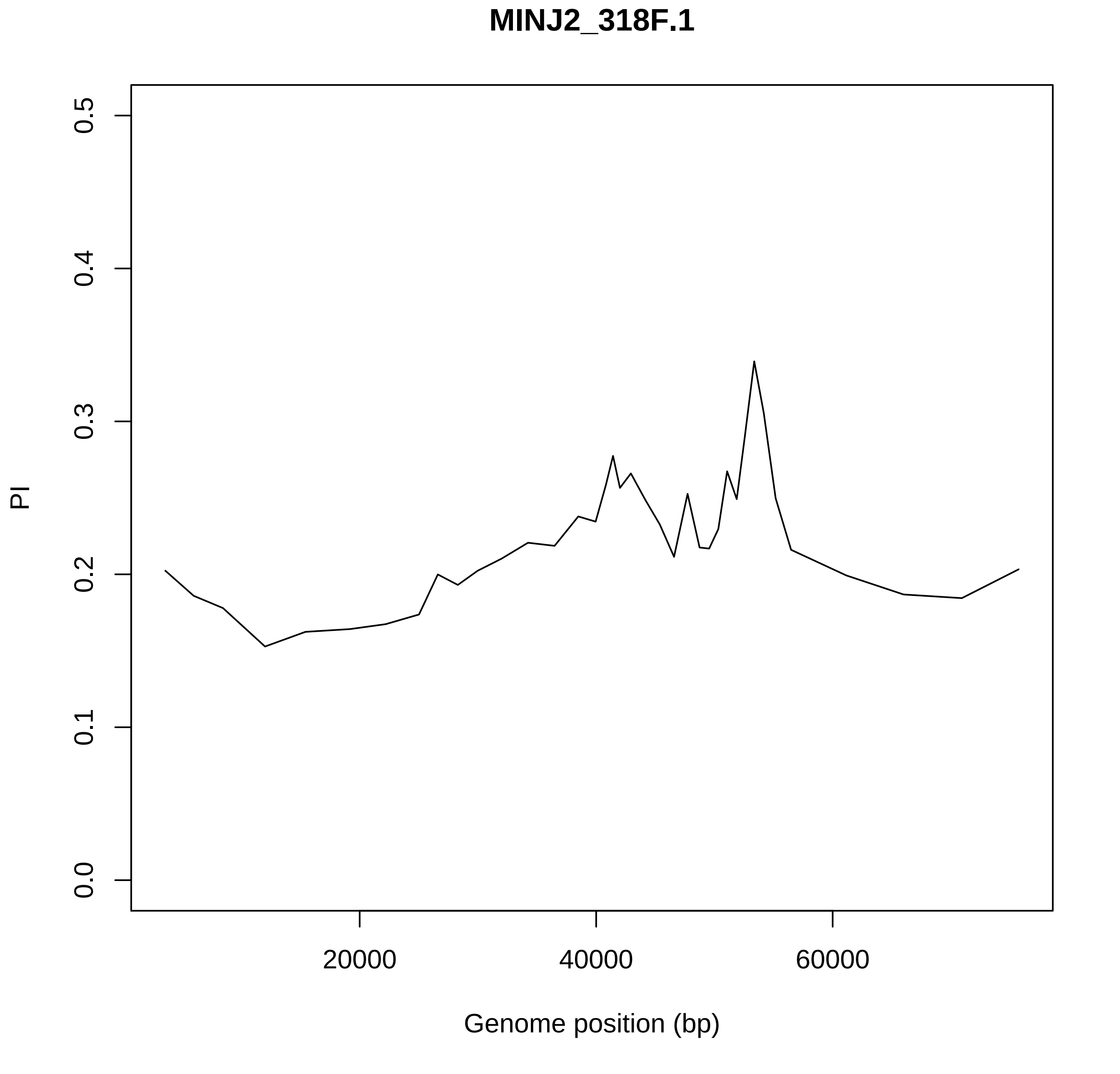
MINJ2_318F.1
PI
0.0
0.1
0.2
0.3
0.4
0.5
20000
40000
60000
Genome position (bp)

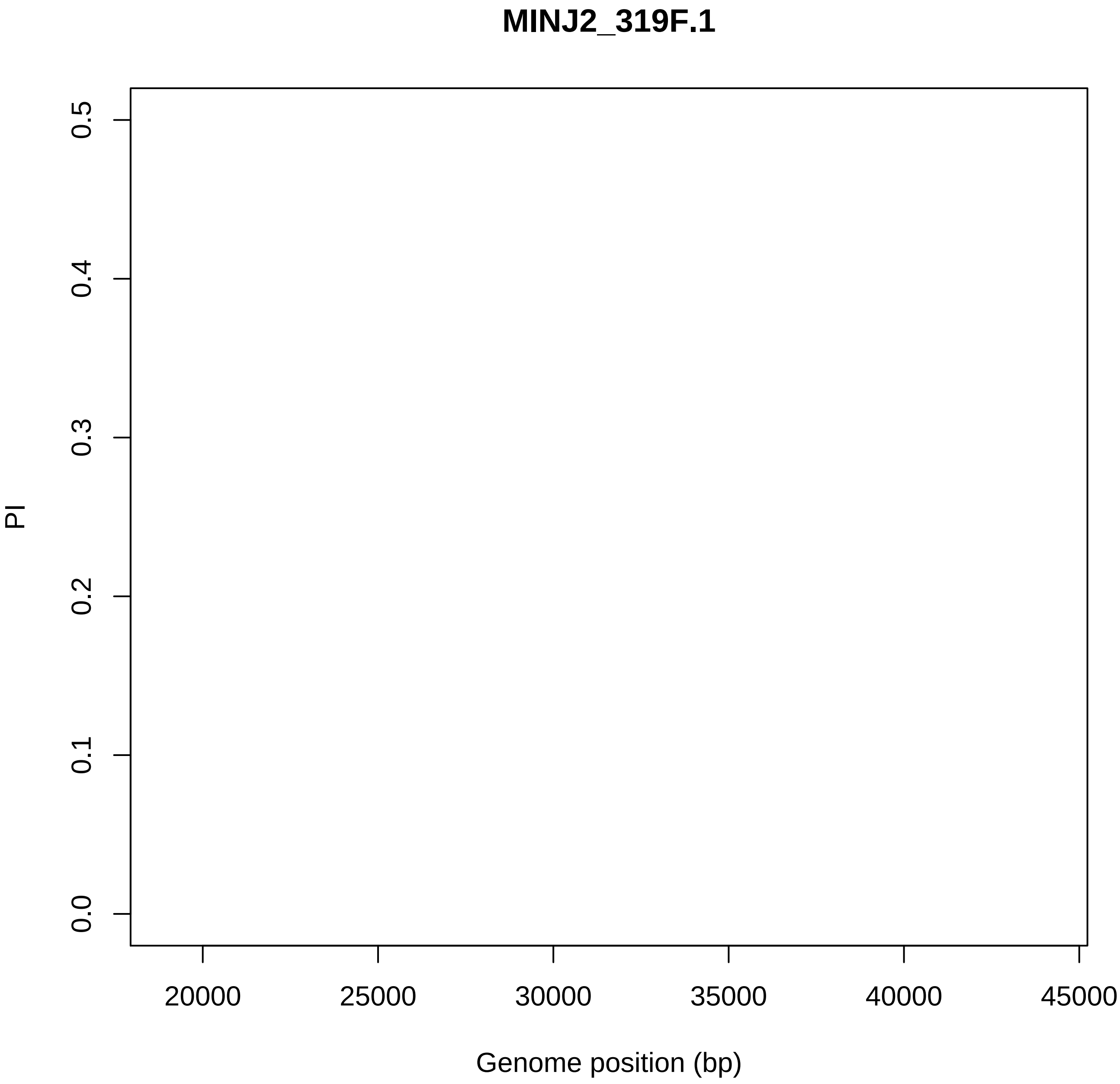
MINJ2_319F.1
PI
0.0
0.1
0.2
0.3
0.4
0.5
20000
25000
30000
35000
40000
45000
Genome position (bp)

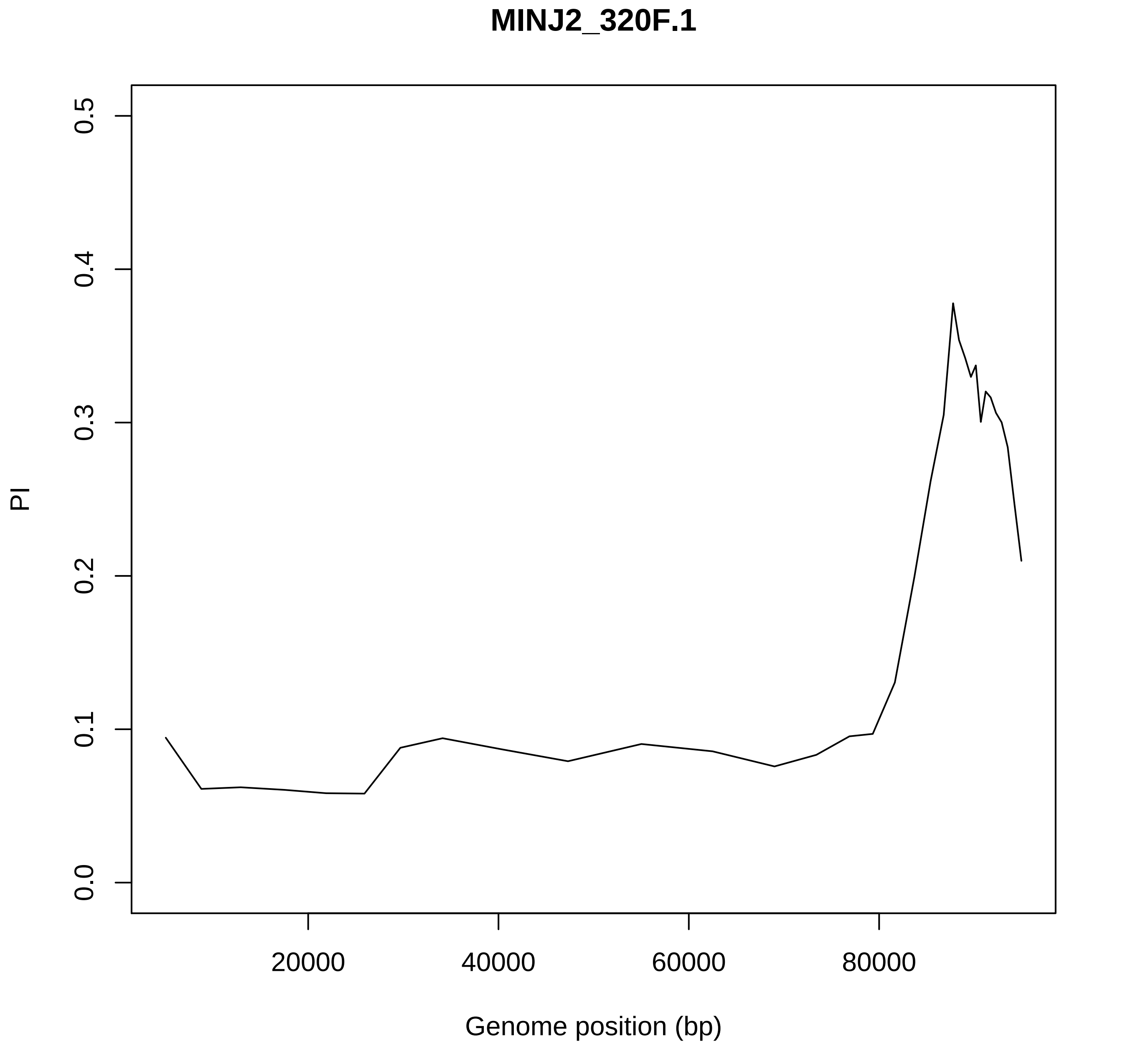
MINJ2_320F.1
PI
0.0
0.1
0.2
0.3
0.4
0.5
20000
40000
60000
80000
Genome position (bp)

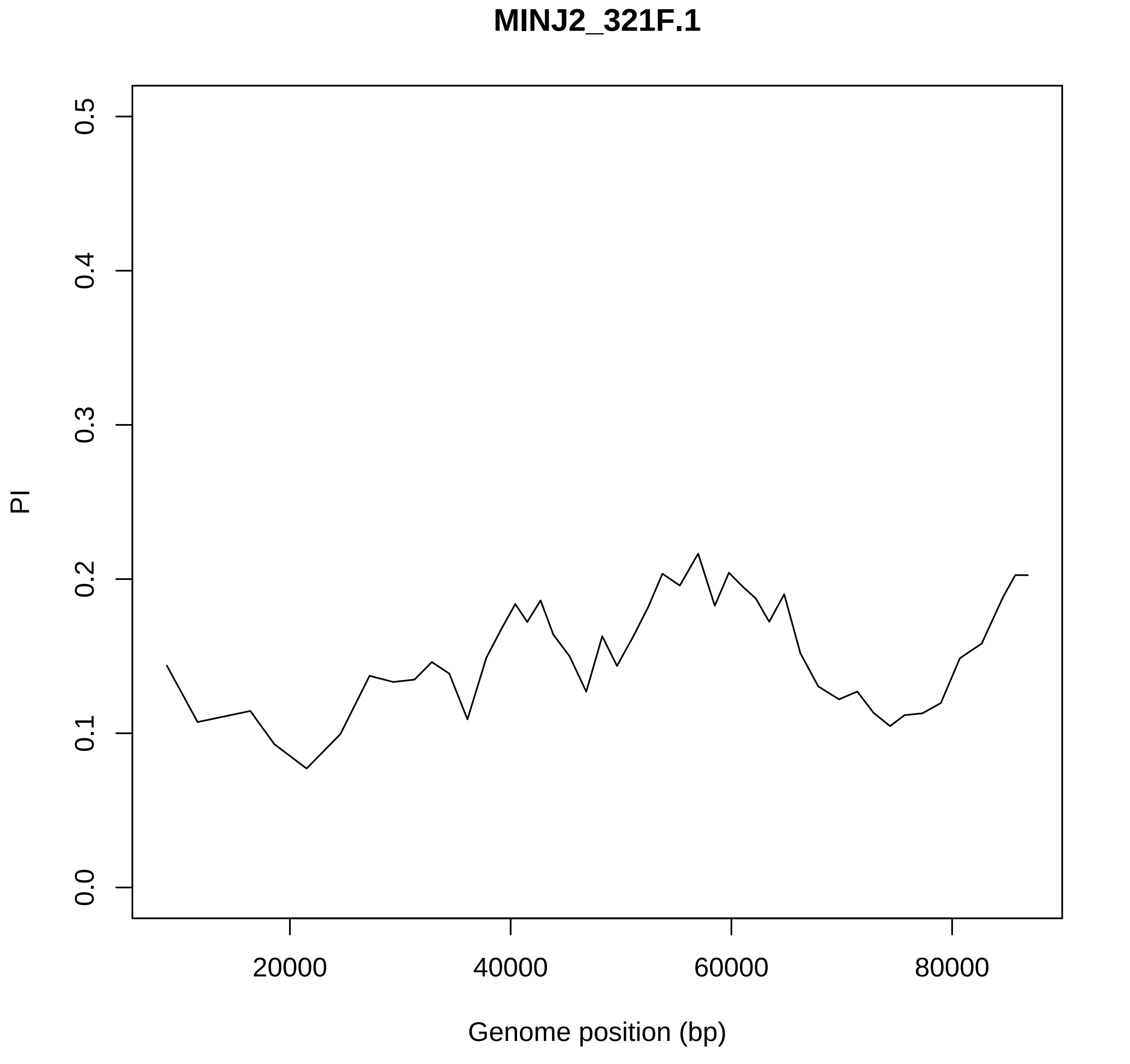

MINJ2_321F.1
PI
0.0
0.1
0.2
0.3
0.4
0.5
20000
40000
60000
80000
Genome position (bp)

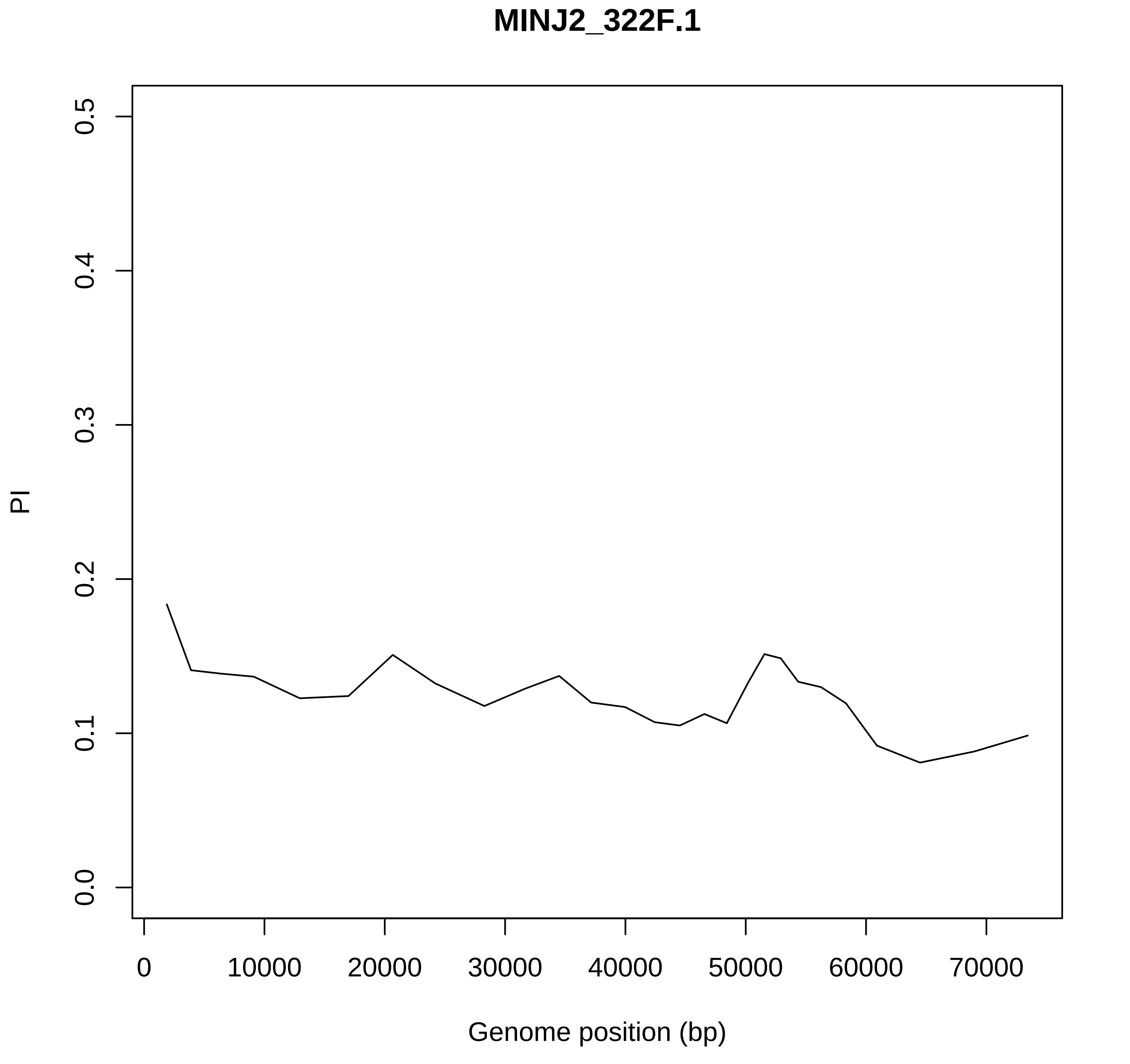
MINJ2_322F.1
PI
0.0
0.1
0.2
0.3
0.4
0.5
0
10000
20000
30000
40000
50000
60000
70000
Genome position (bp)

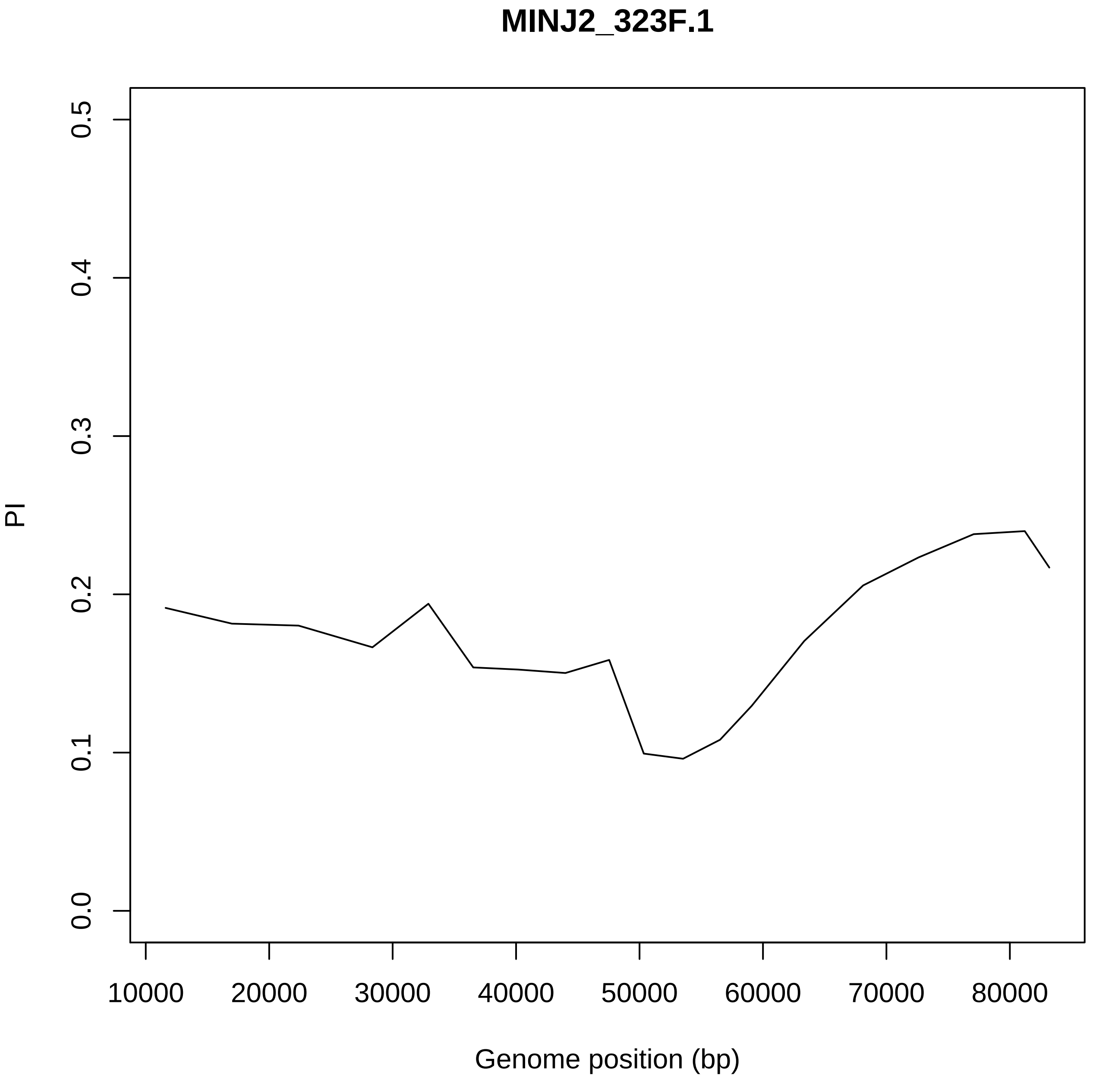
MINJ2_323F.1
PI
0.0
0.1
0.2
0.3
0.4
0.5
10000
20000
30000
40000
50000
60000
70000
80000
Genome position (bp)

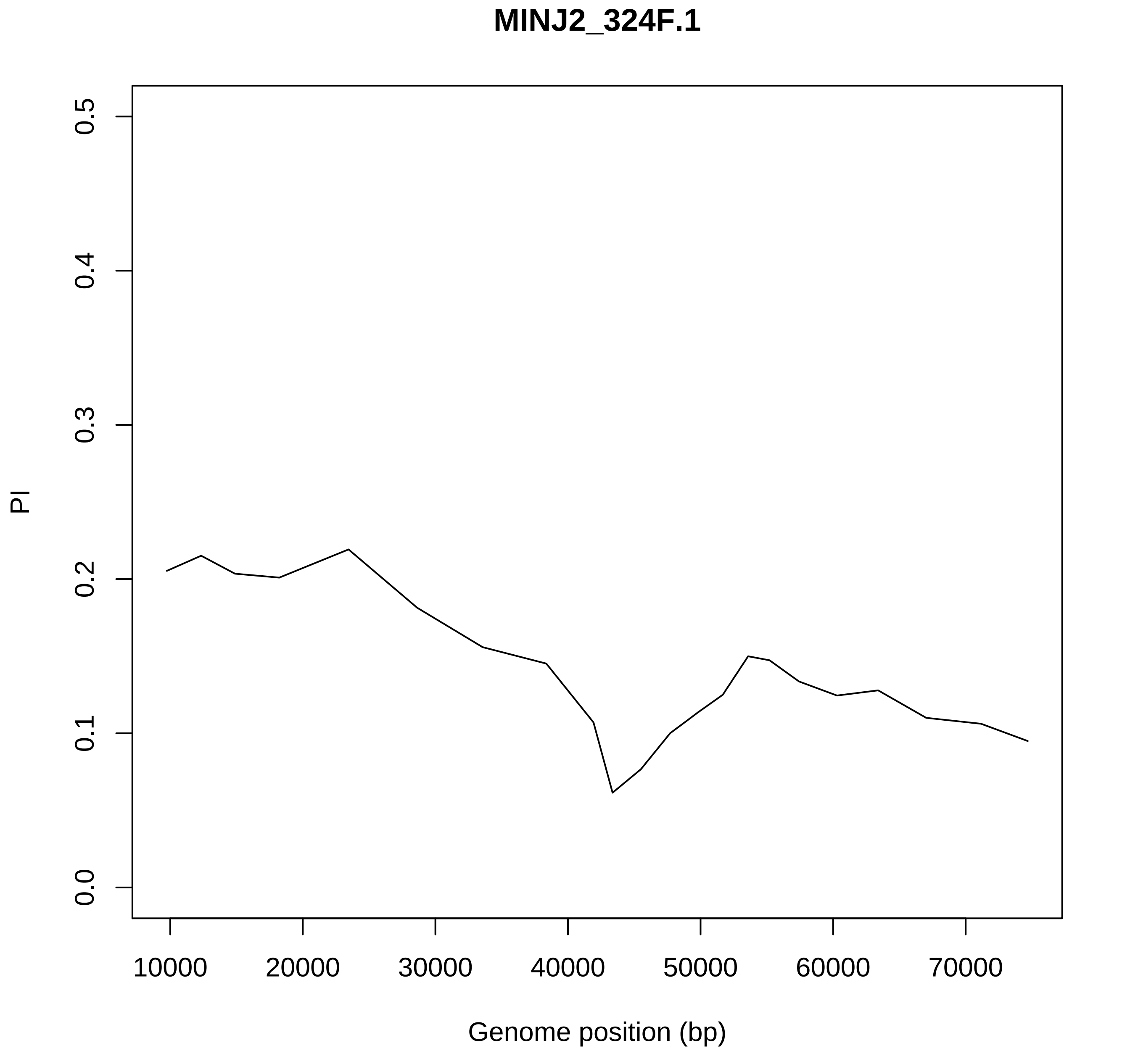
MINJ2_324F.1
PI
0.0
0.1
0.2
0.3
0.4
0.5
10000
20000
30000
40000
50000
60000
70000
Genome position (bp)

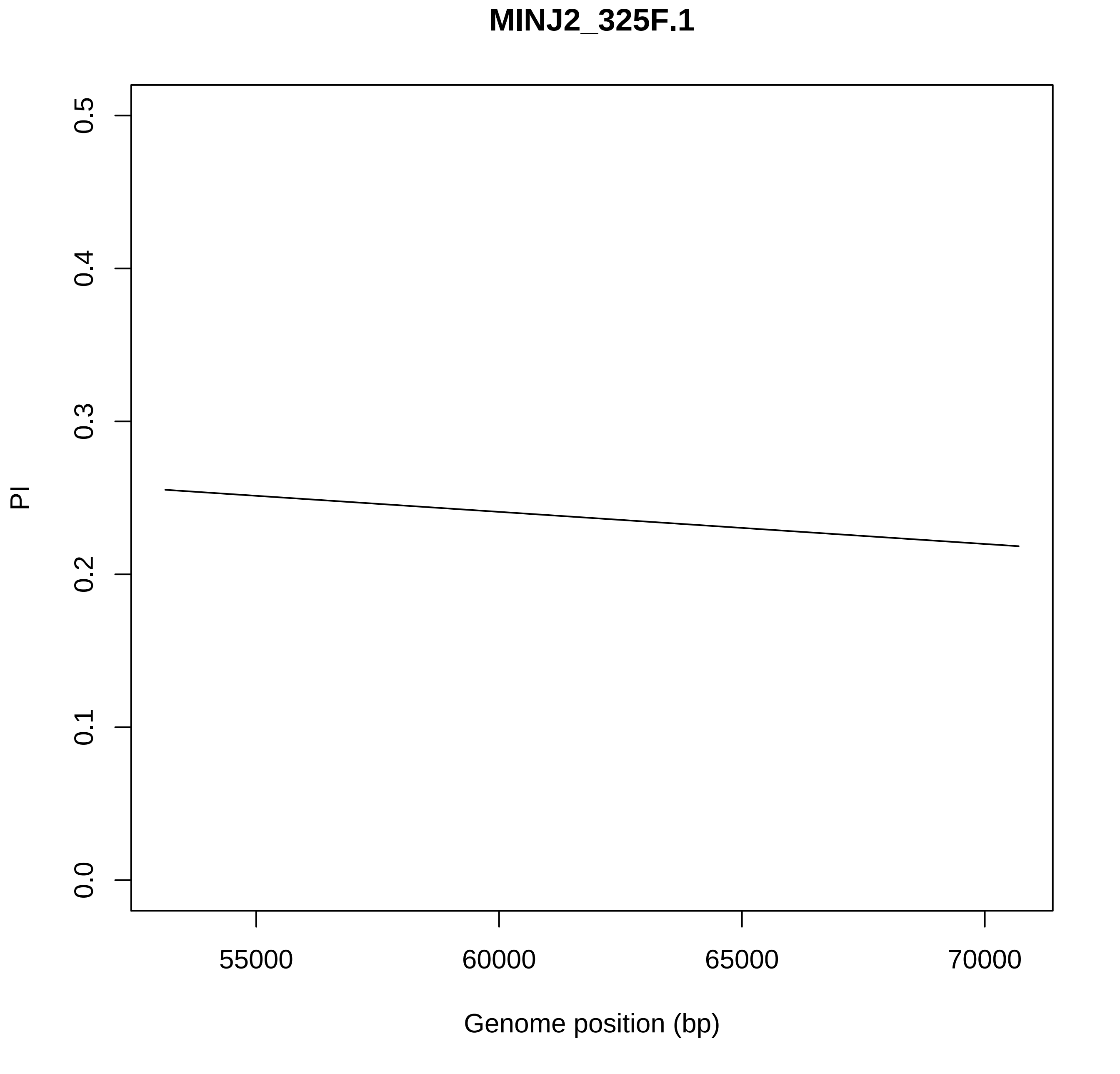
MINJ2_325F.1
PI
0.0
0.1
0.2
0.3
0.4
0.5
55000
60000
65000
70000
Genome position (bp)

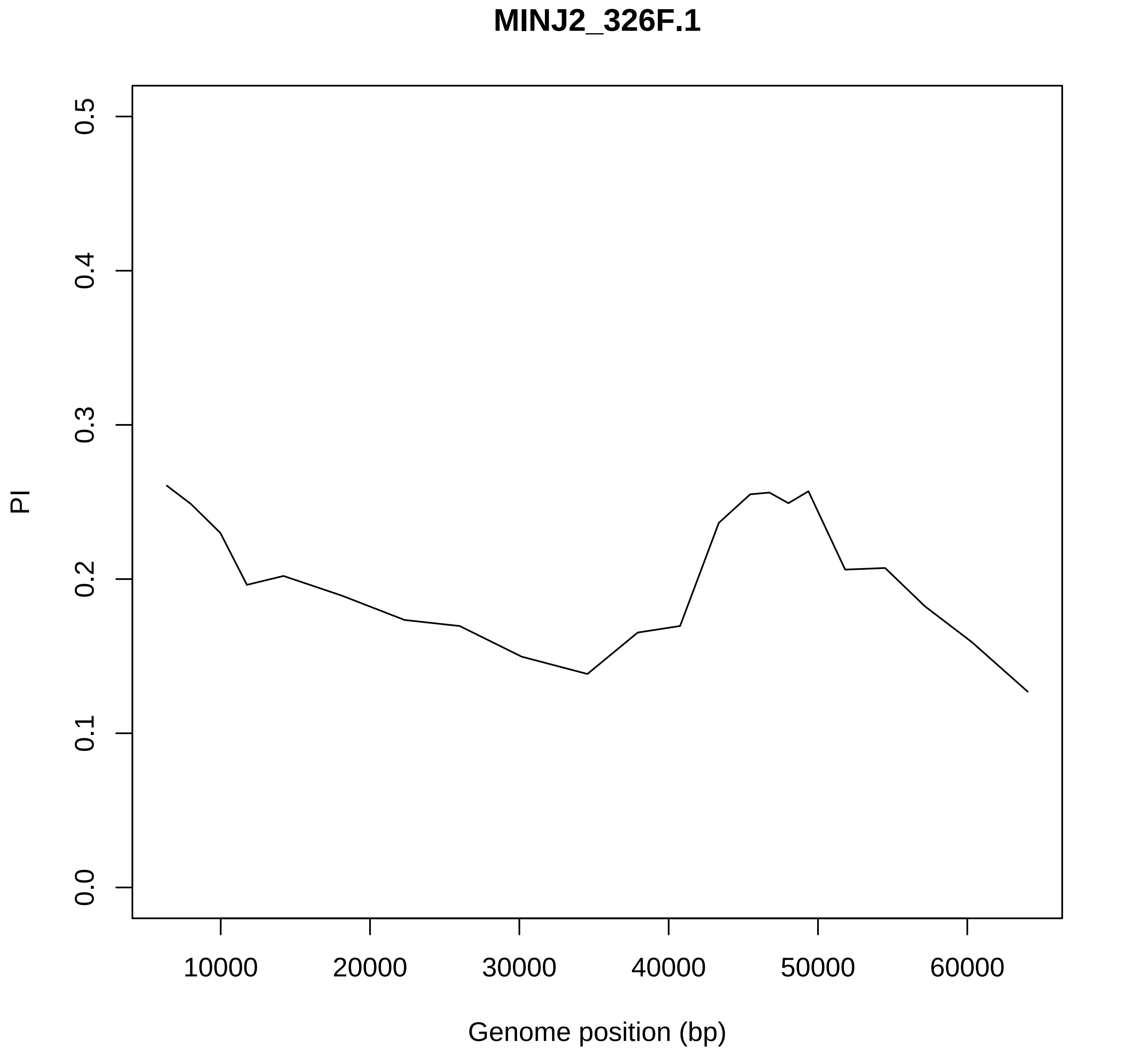
MINJ2_326F.1
PI
0.0
0.1
0.2
0.3
0.4
0.5
10000
20000
30000
40000
50000
60000
Genome position (bp)

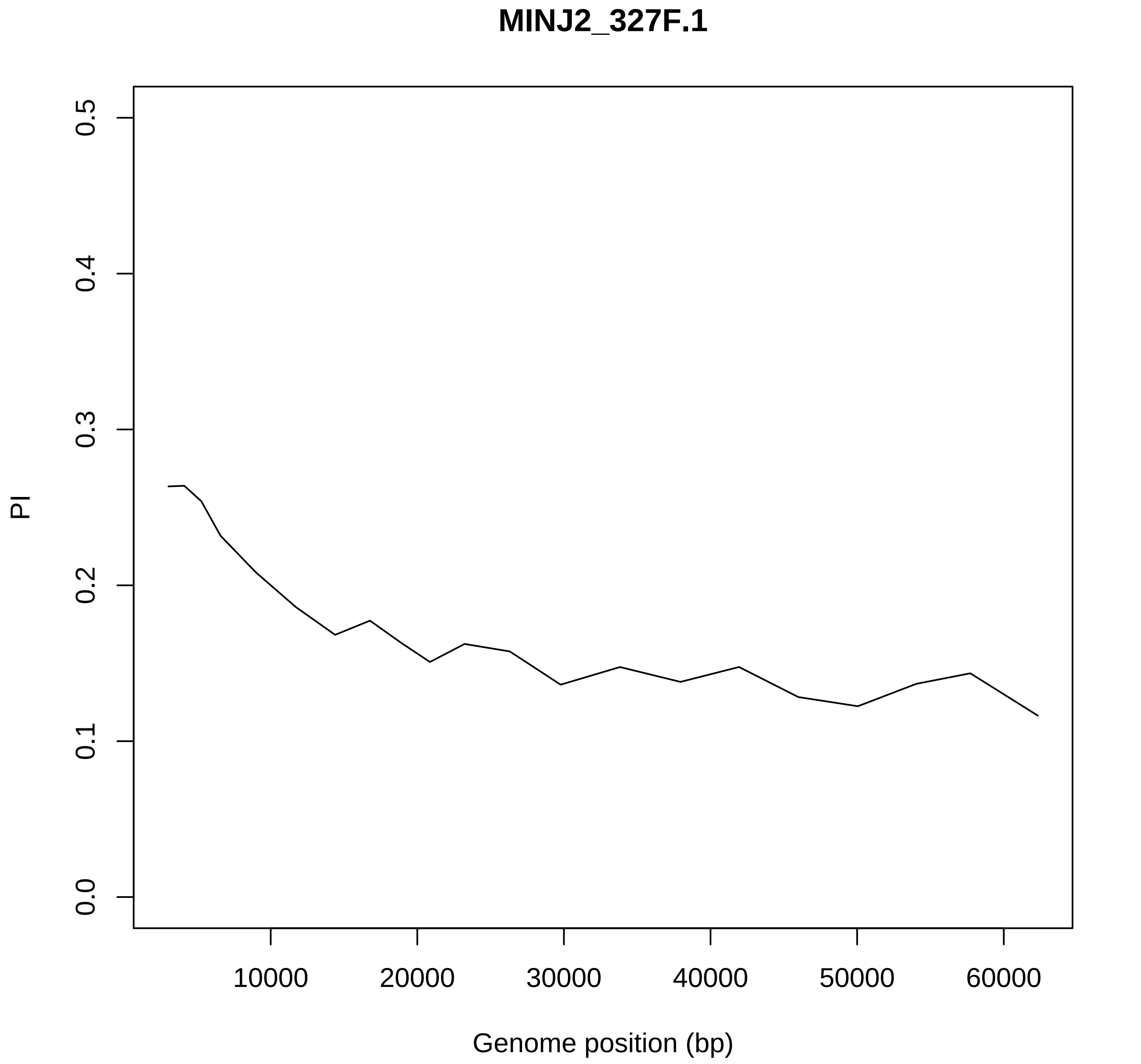

MINJ2_327F.1
PI
0.0
0.1
0.2
0.3
0.4
0.5
10000
20000
30000
40000
50000
60000
Genome position (bp)

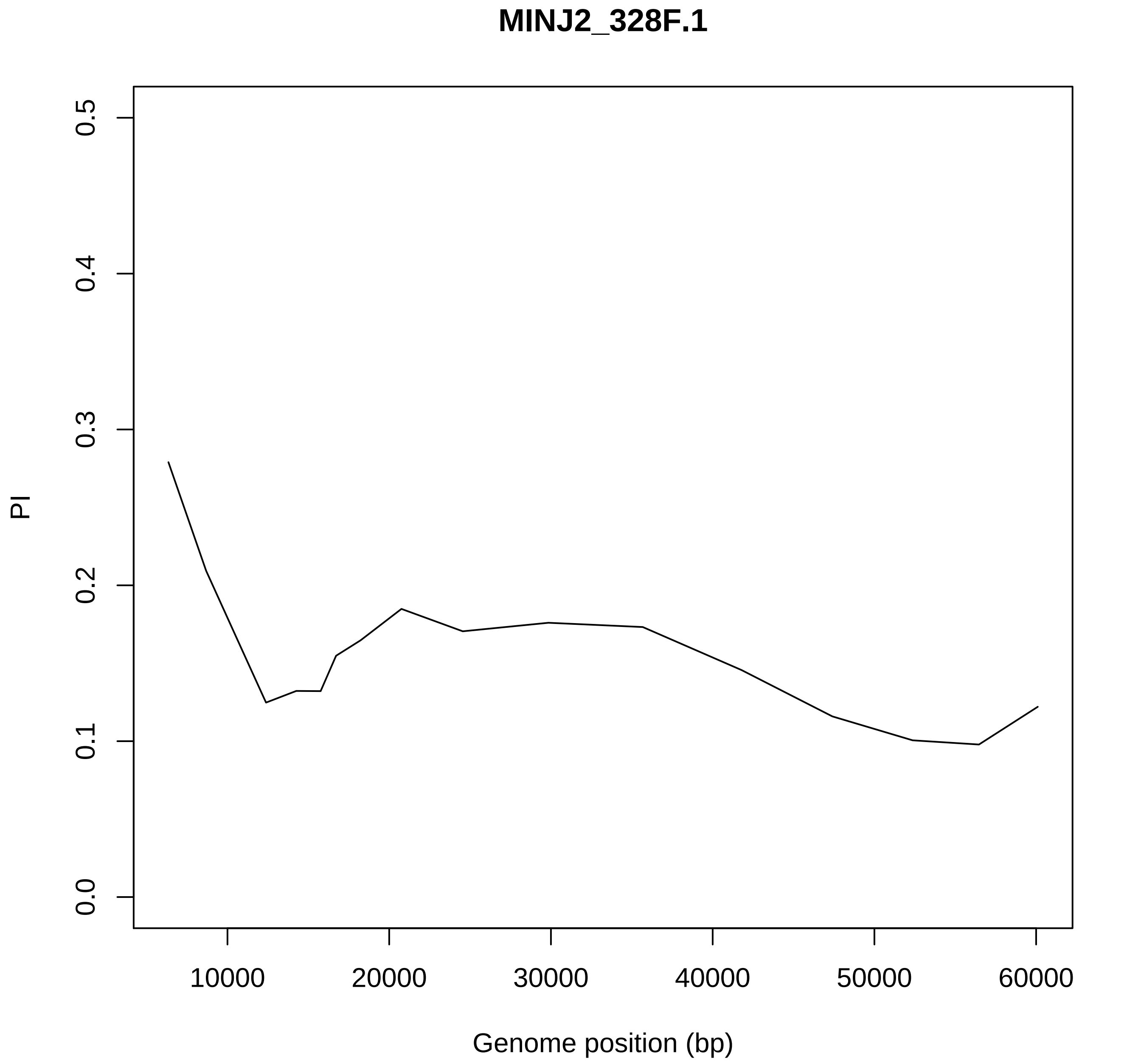

MINJ2_328F.1
PI
0.0
0.1
0.2
0.3
0.4
0.5
10000
20000
30000
40000
50000
60000
Genome position (bp)

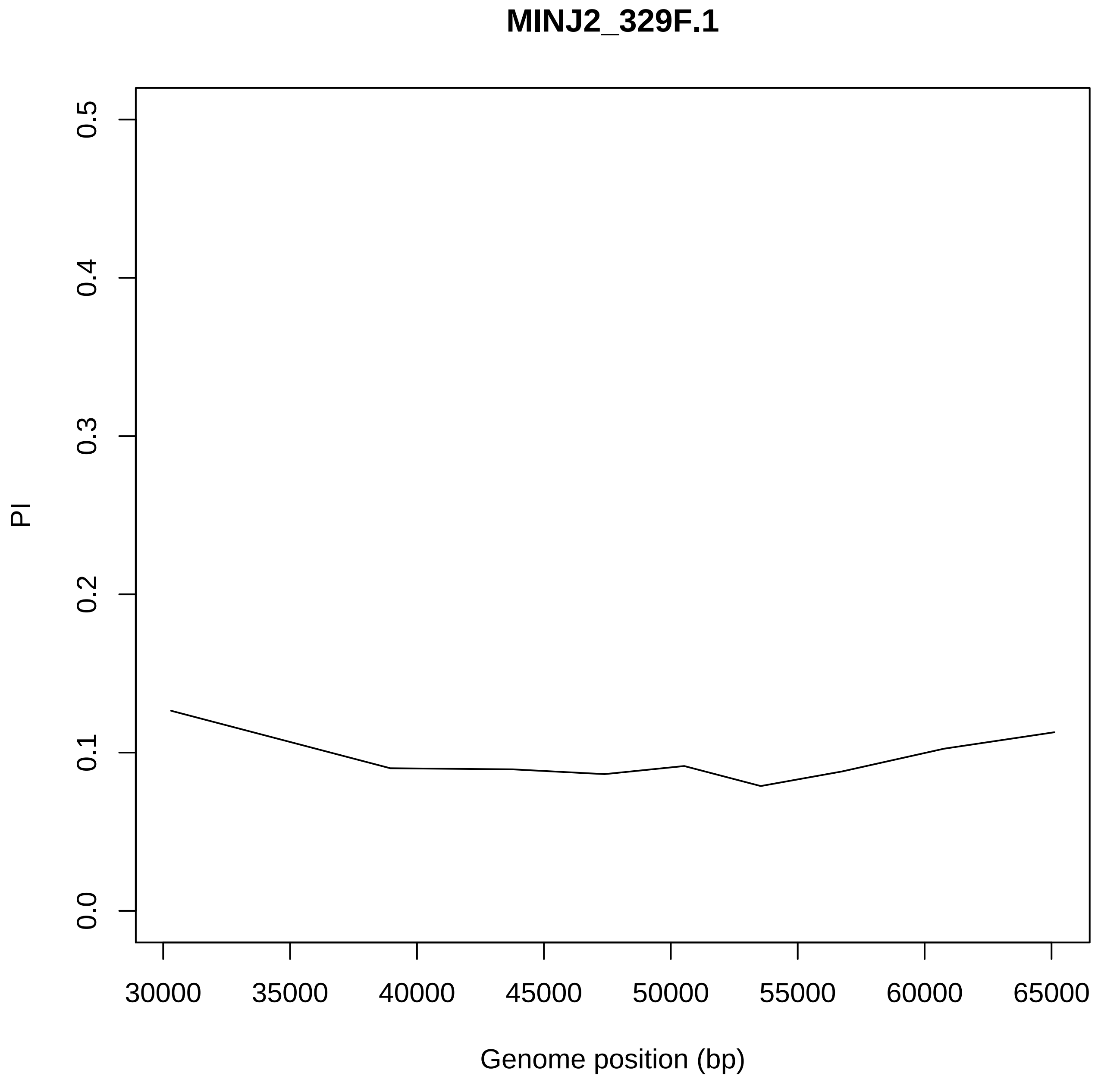
MINJ2_329F.1
PI
0.0
0.1
0.2
0.3
0.4
0.5
30000
35000
40000
45000
50000
55000
60000
65000
Genome position (bp)

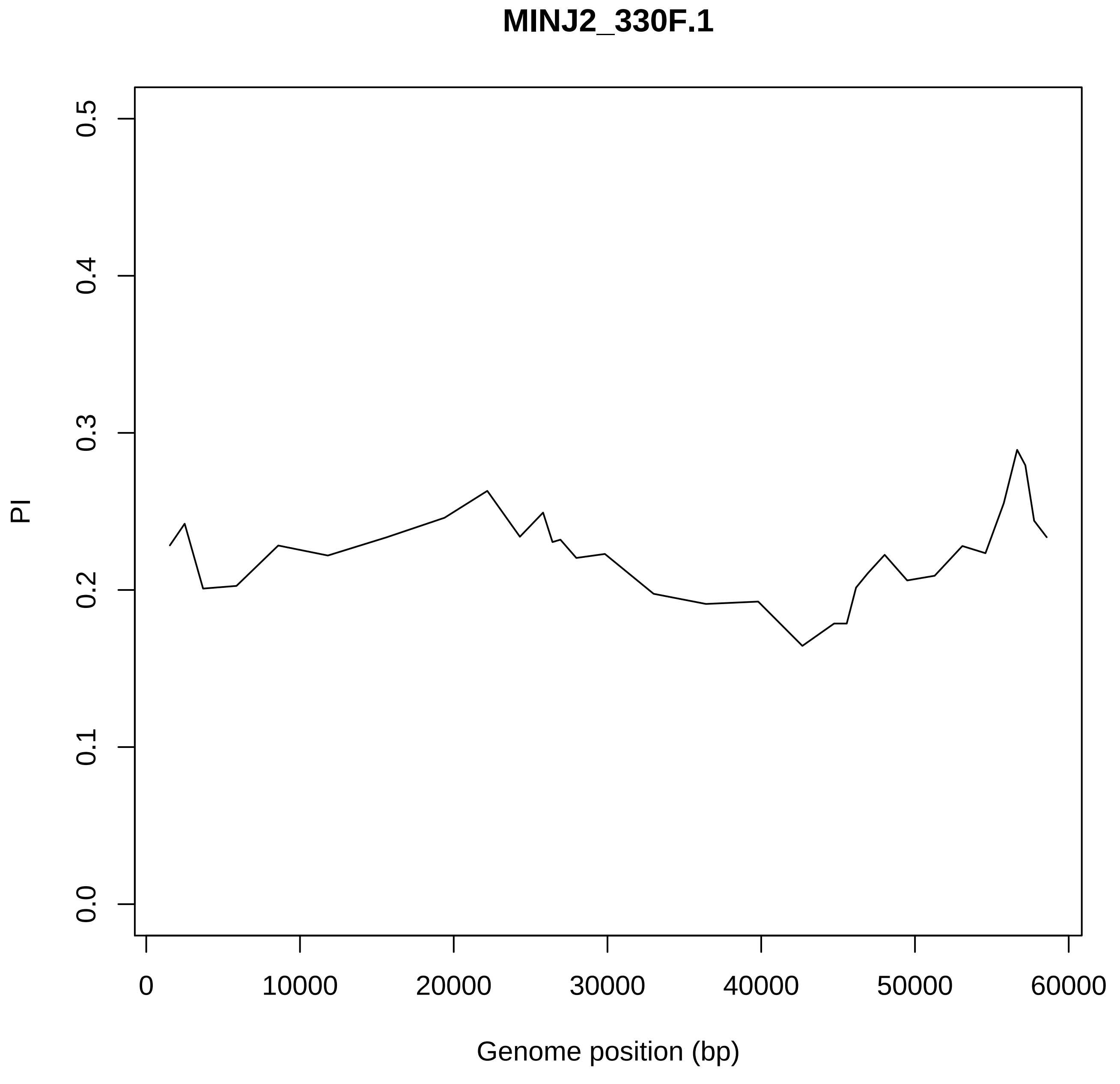

MINJ2_330F.1
PI
0.0
0.1
0.2
0.3
0.4
0.5
0
10000
20000
30000
40000
50000
60000
Genome position (bp)

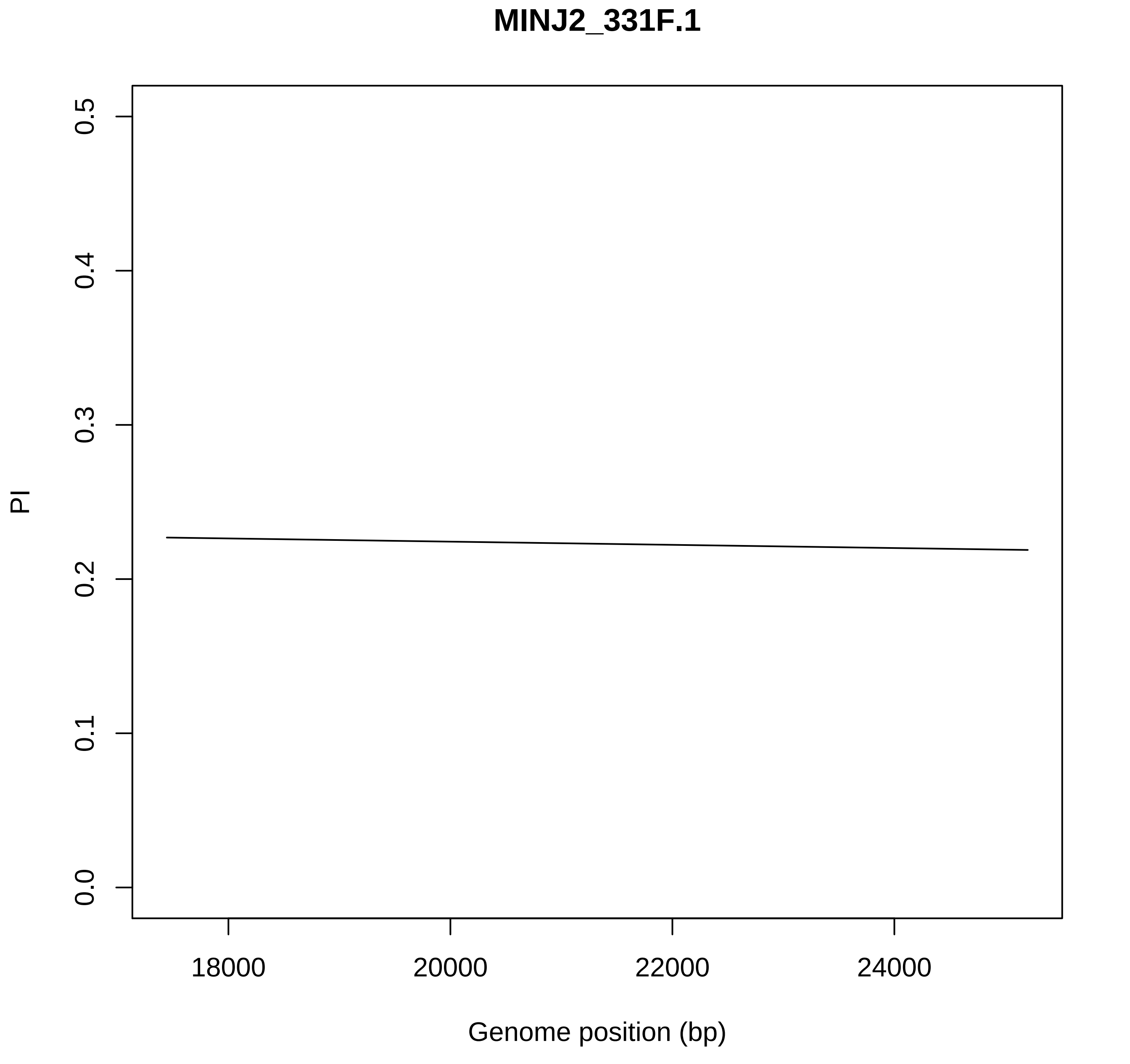
MINJ2_331F.1
PI
0.0
0.1
0.2
0.3
0.4
0.5
18000
20000
22000
24000
Genome position (bp)

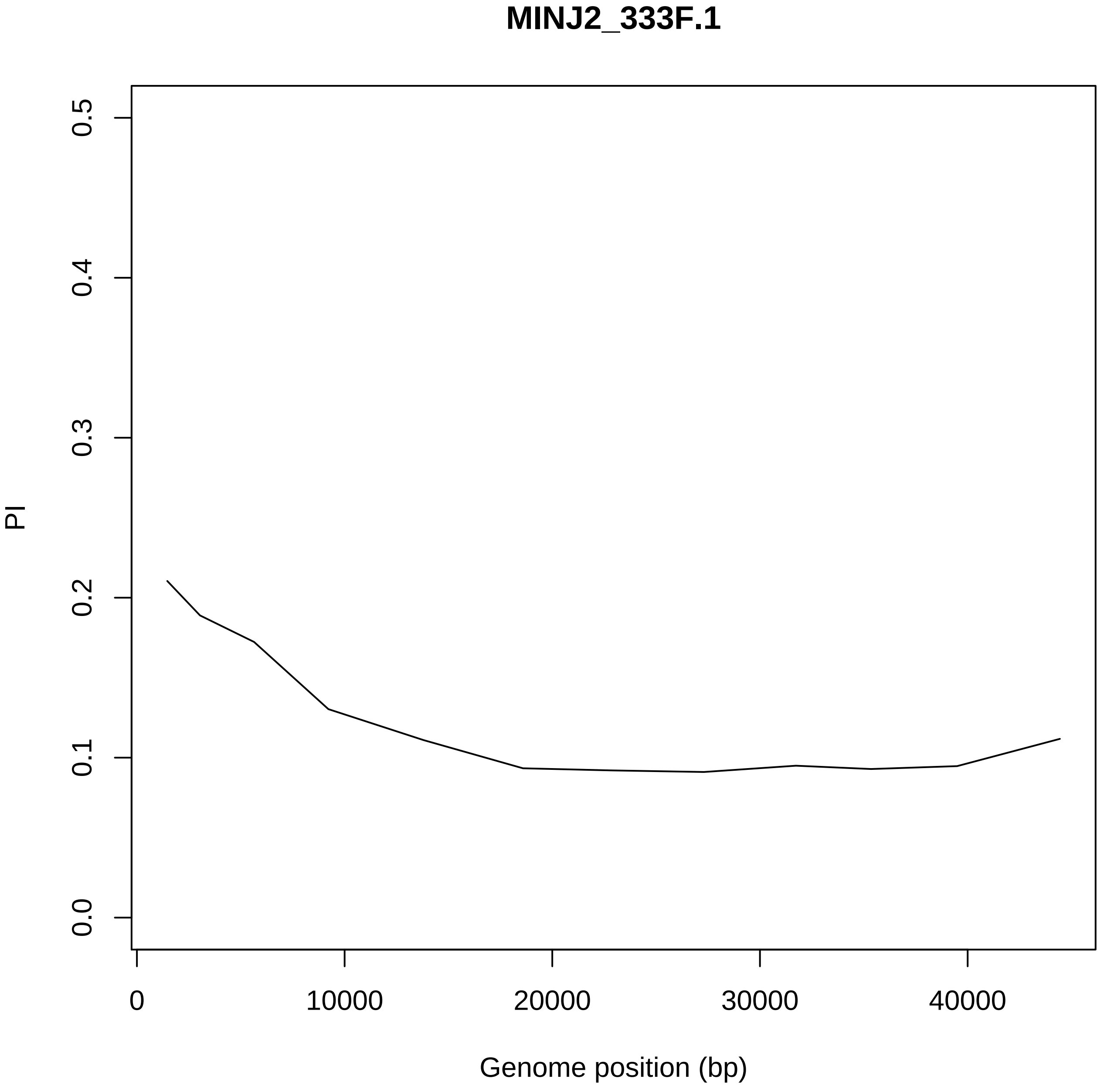

MINJ2_333F.1
PI
0.0
0.1
0.2
0.3
0.4
0.5
0
10000
20000
30000
40000
Genome position (bp)

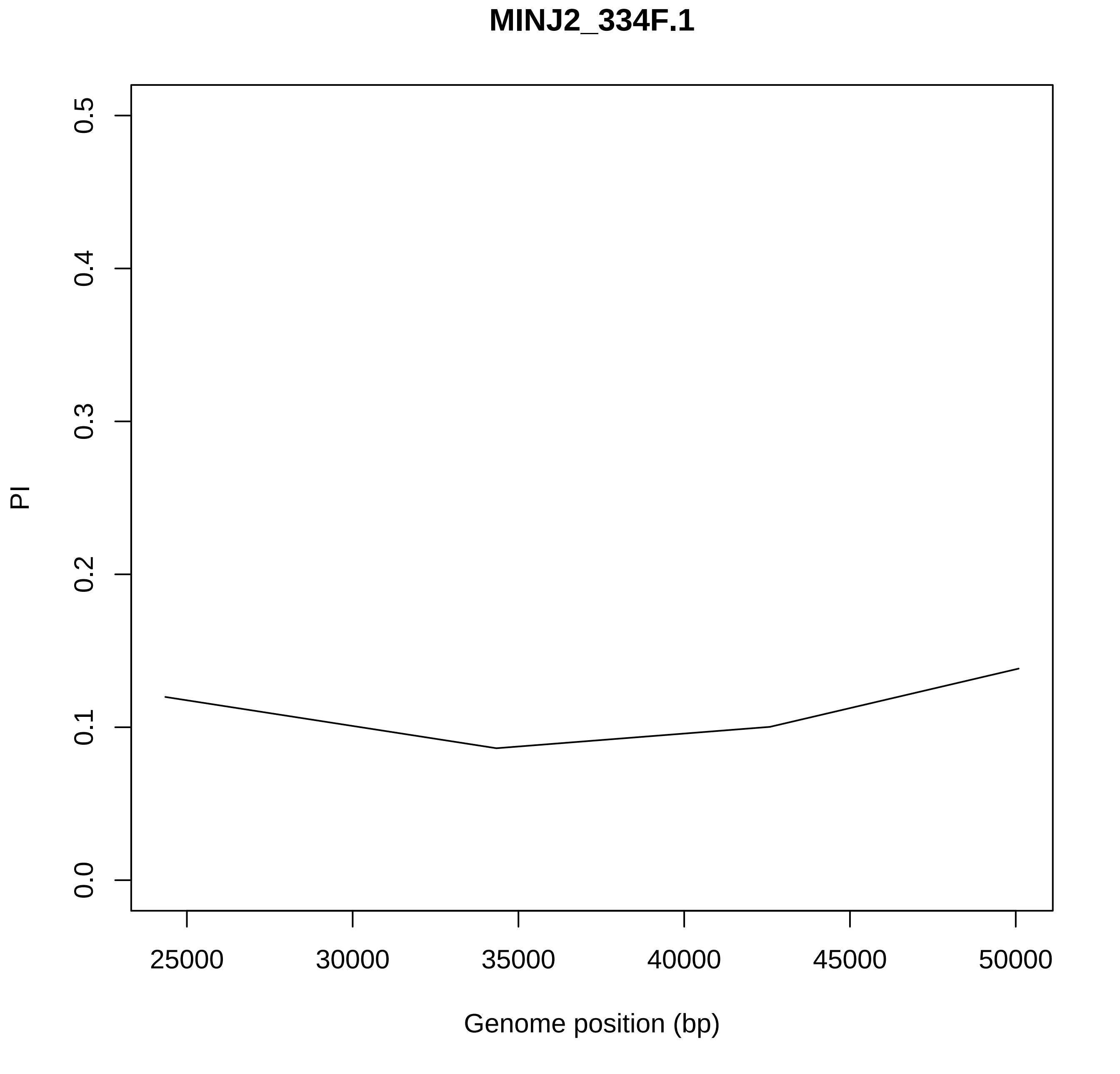
MINJ2_334F.1
PI
0.0
0.1
0.2
0.3
0.4
0.5
25000
30000
35000
40000
45000
50000
Genome position (bp)

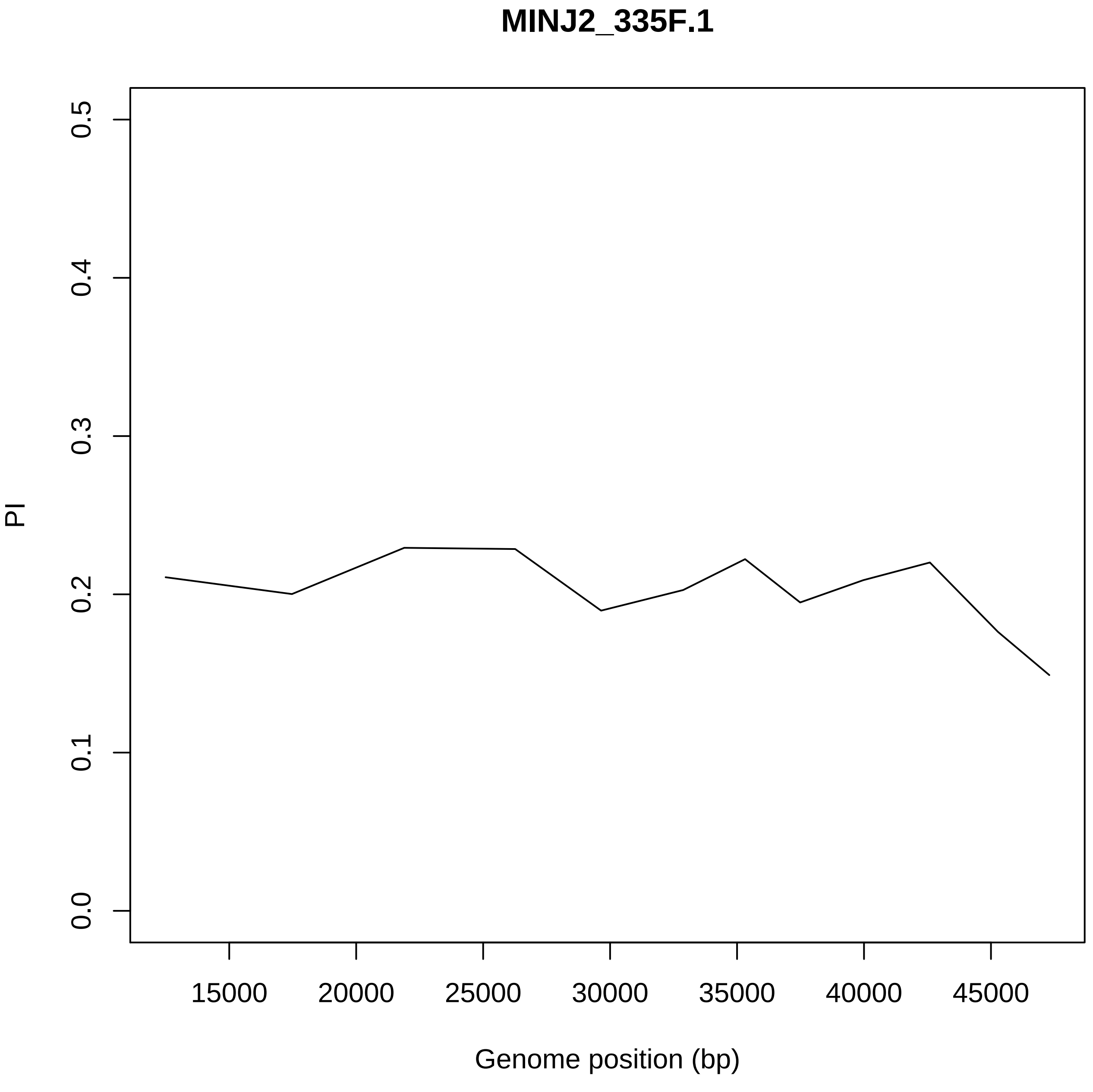

MINJ2_335F.1
PI
0.0
0.1
0.2
0.3
0.4
0.5
15000
20000
25000
30000
35000
40000
45000
Genome position (bp)

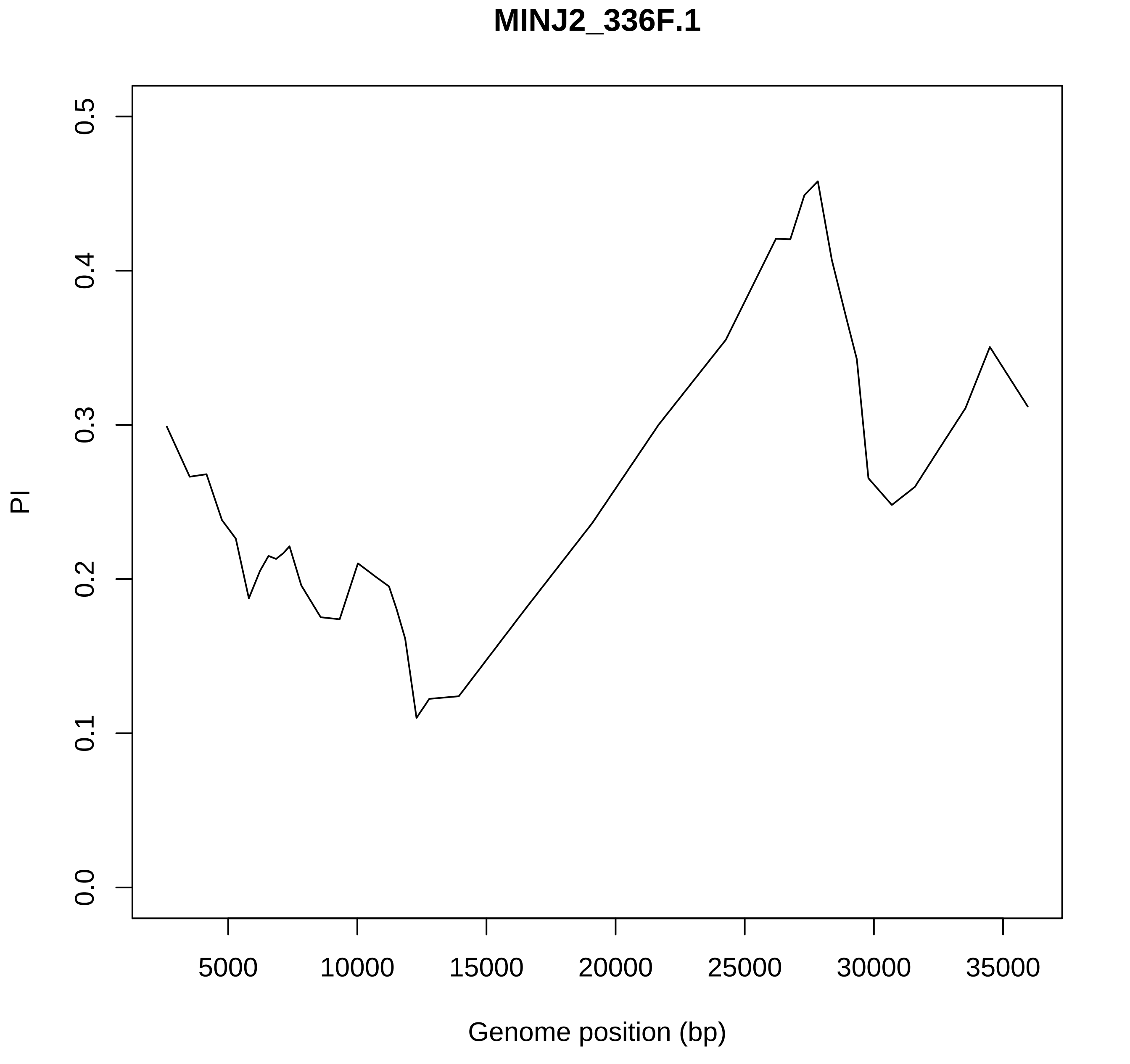
MINJ2_336F.1
PI
0.0
0.1
0.2
0.3
0.4
0.5
5000
10000
15000
20000
25000
30000
35000
Genome position (bp)

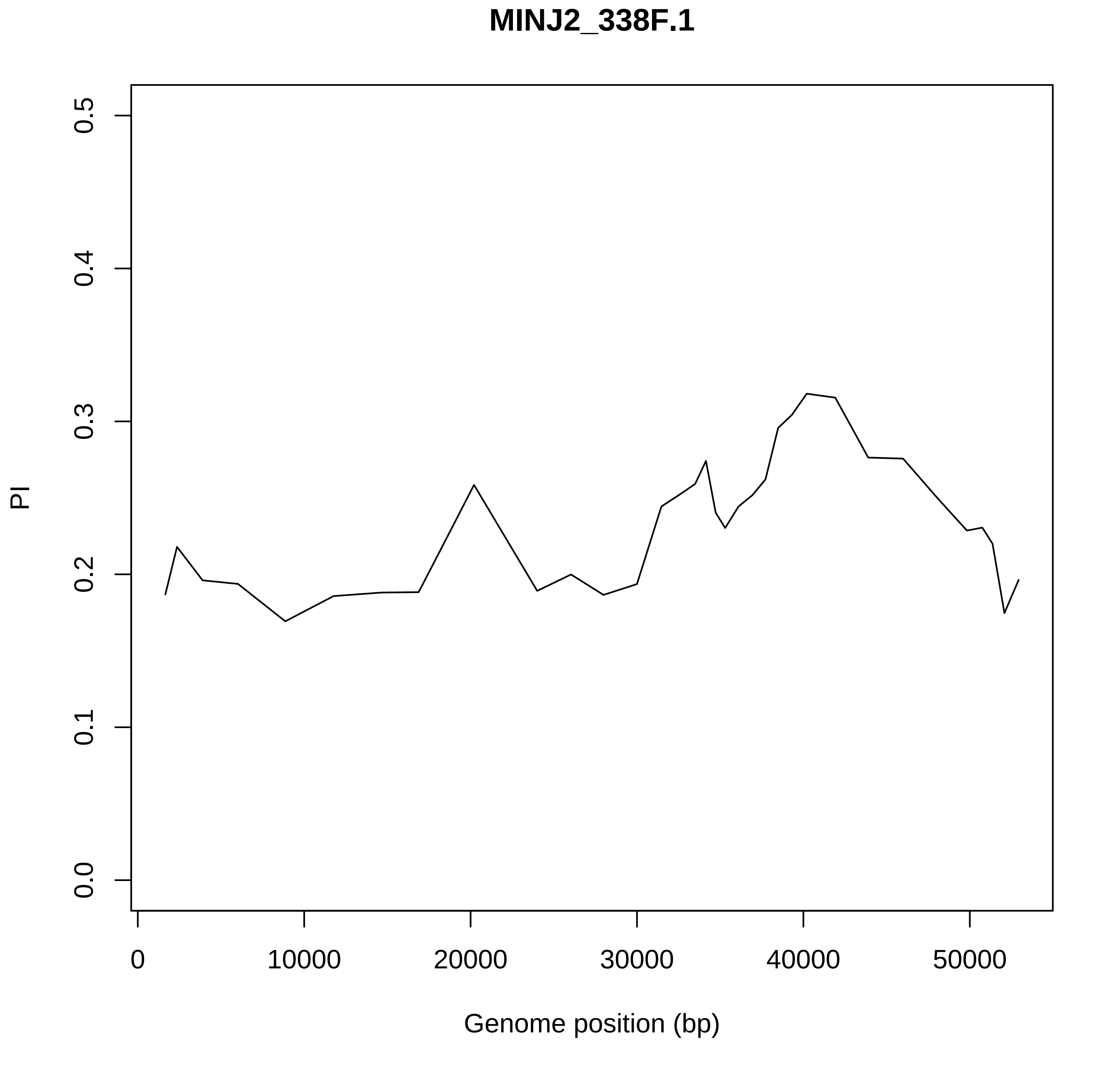

MINJ2_338F.1
PI
0.0
0.1
0.2
0.3
0.4
0.5
0
10000
20000
30000
40000
50000
Genome position (bp)

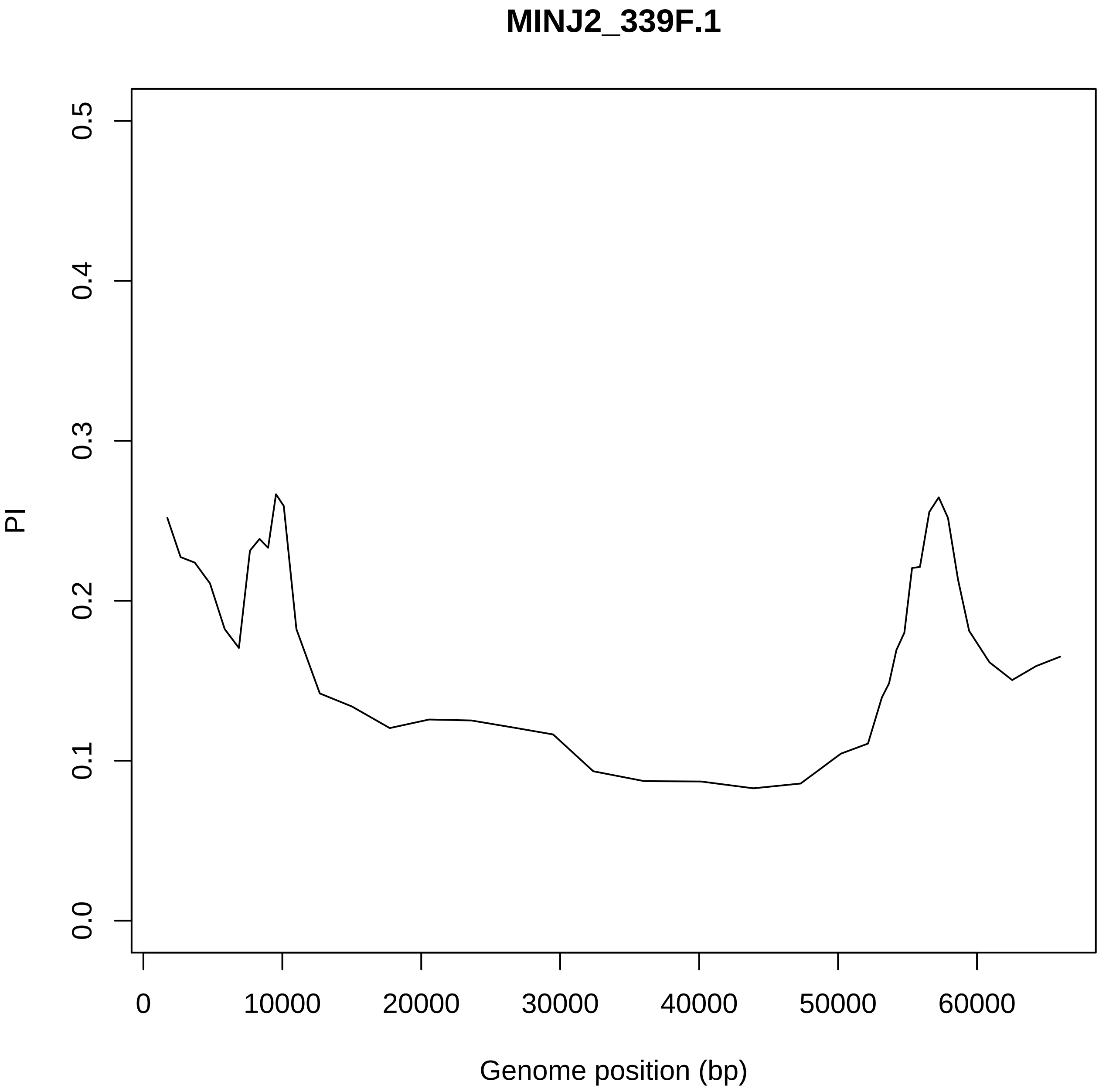
MINJ2_339F.1
PI
0.0
0.1
0.2
0.3
0.4
0.5
0
10000
20000
30000
40000
50000
60000
Genome position (bp)

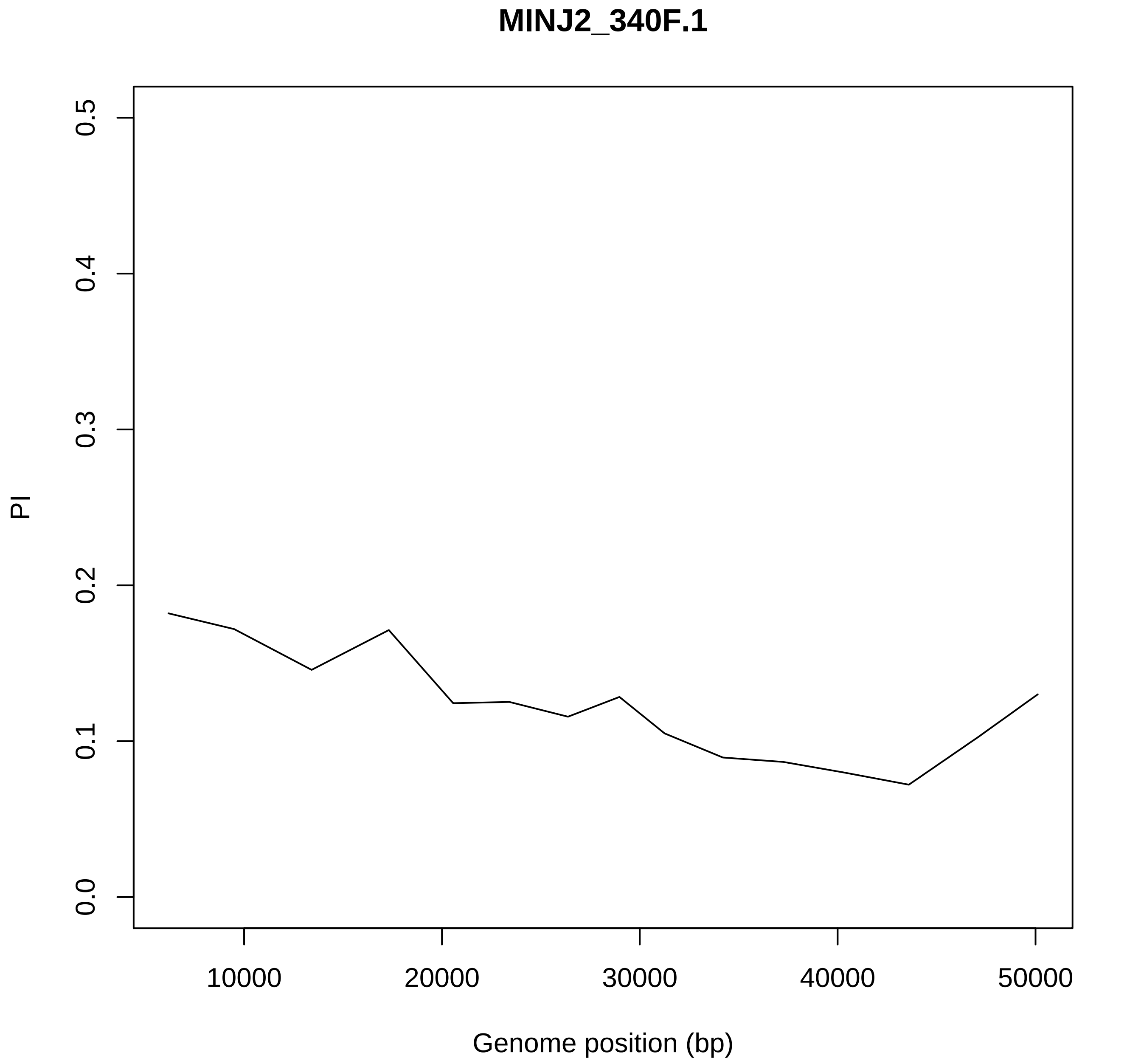
MINJ2_340F.1
PI
0.0
0.1
0.2
0.3
0.4
0.5
10000
20000
30000
40000
50000
Genome position (bp)

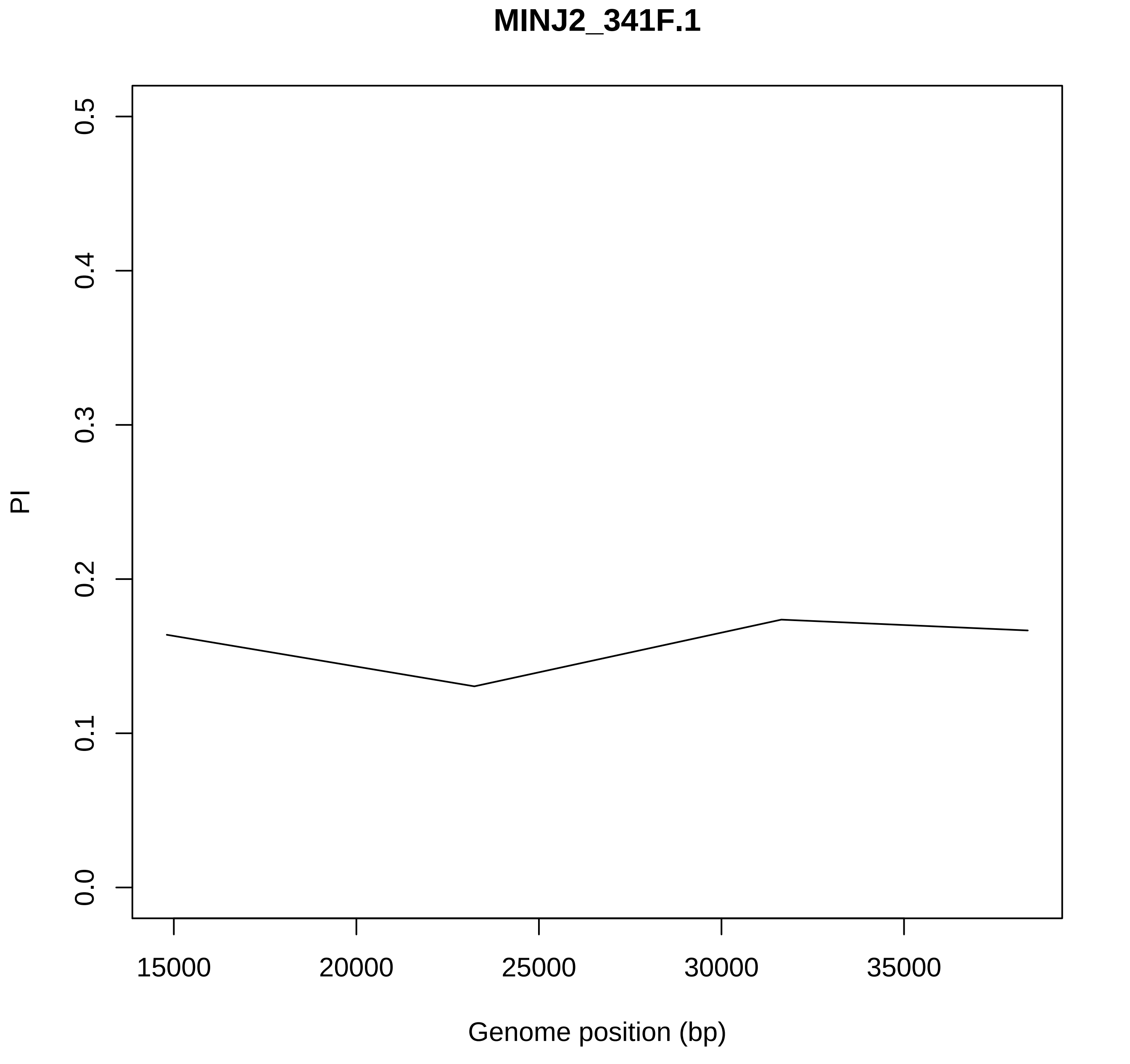
MINJ2_341F.1
PI
0.0
0.1
0.2
0.3
0.4
0.5
15000
20000
25000
30000
35000
Genome position (bp)

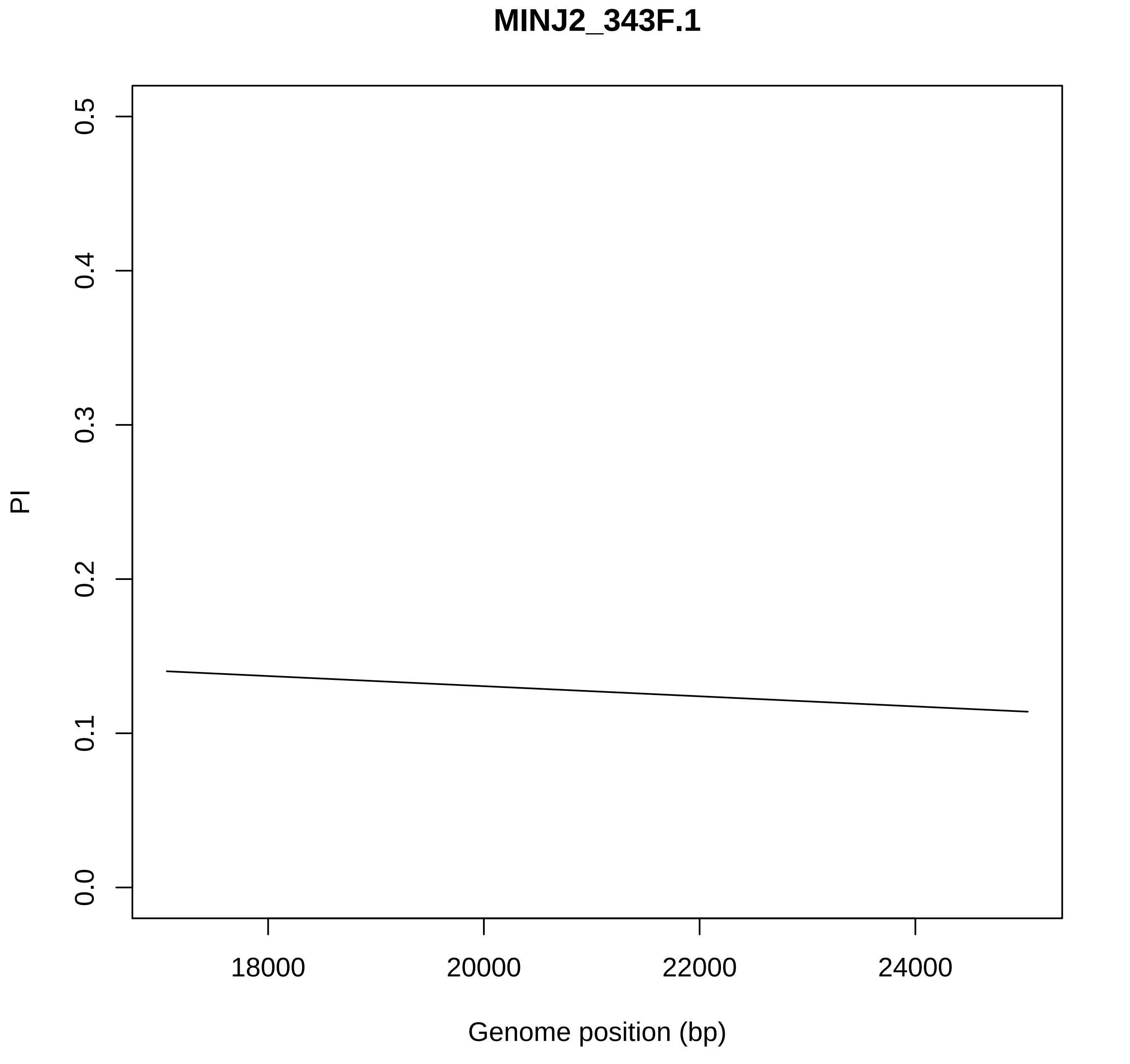
MINJ2_343F.1
PI
0.0
0.1
0.2
0.3
0.4
0.5
18000
20000
22000
24000
Genome position (bp)

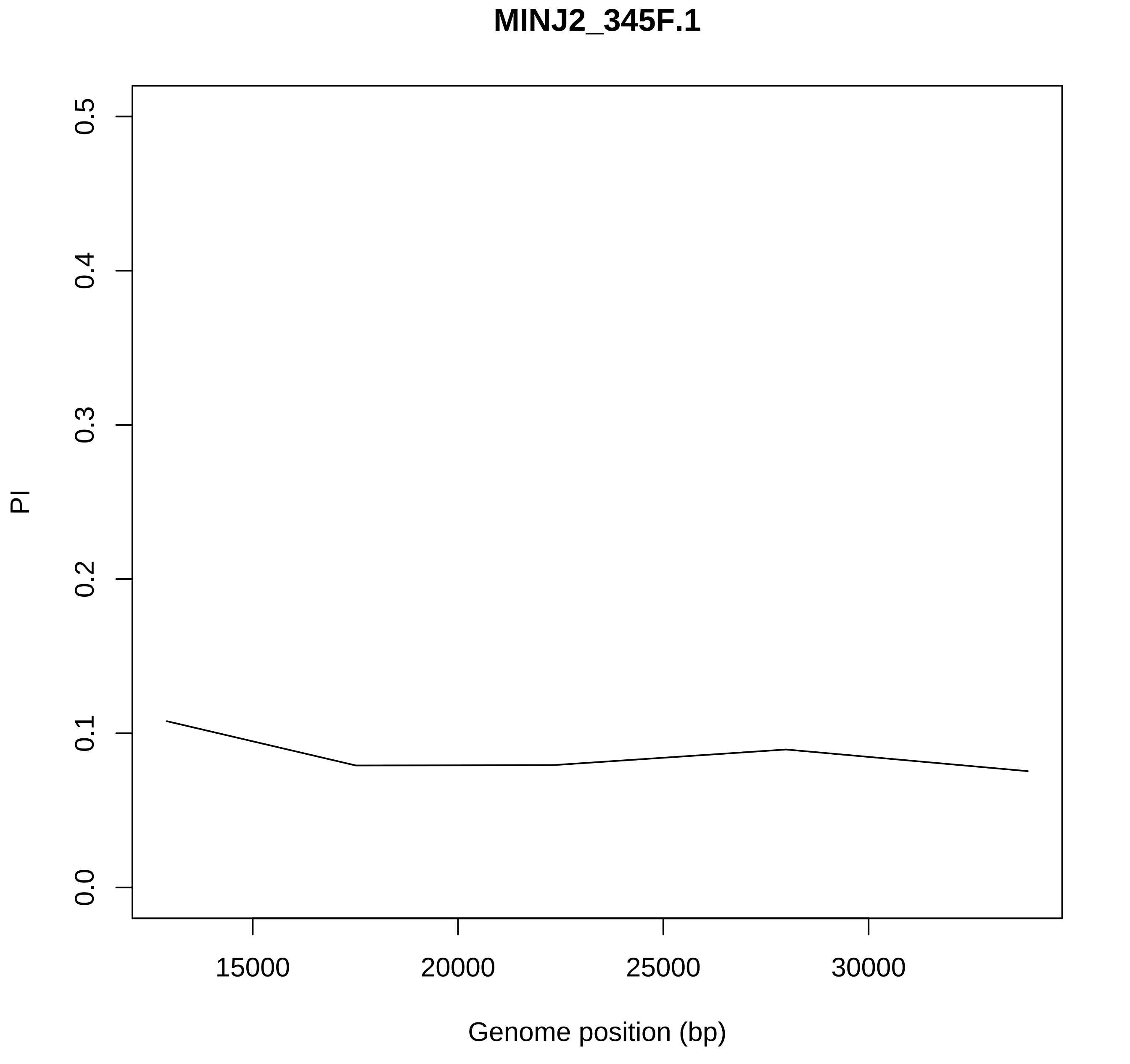
MINJ2_345F.1
PI
0.0
0.1
0.2
0.3
0.4
0.5
15000
20000
25000
30000
Genome position (bp)

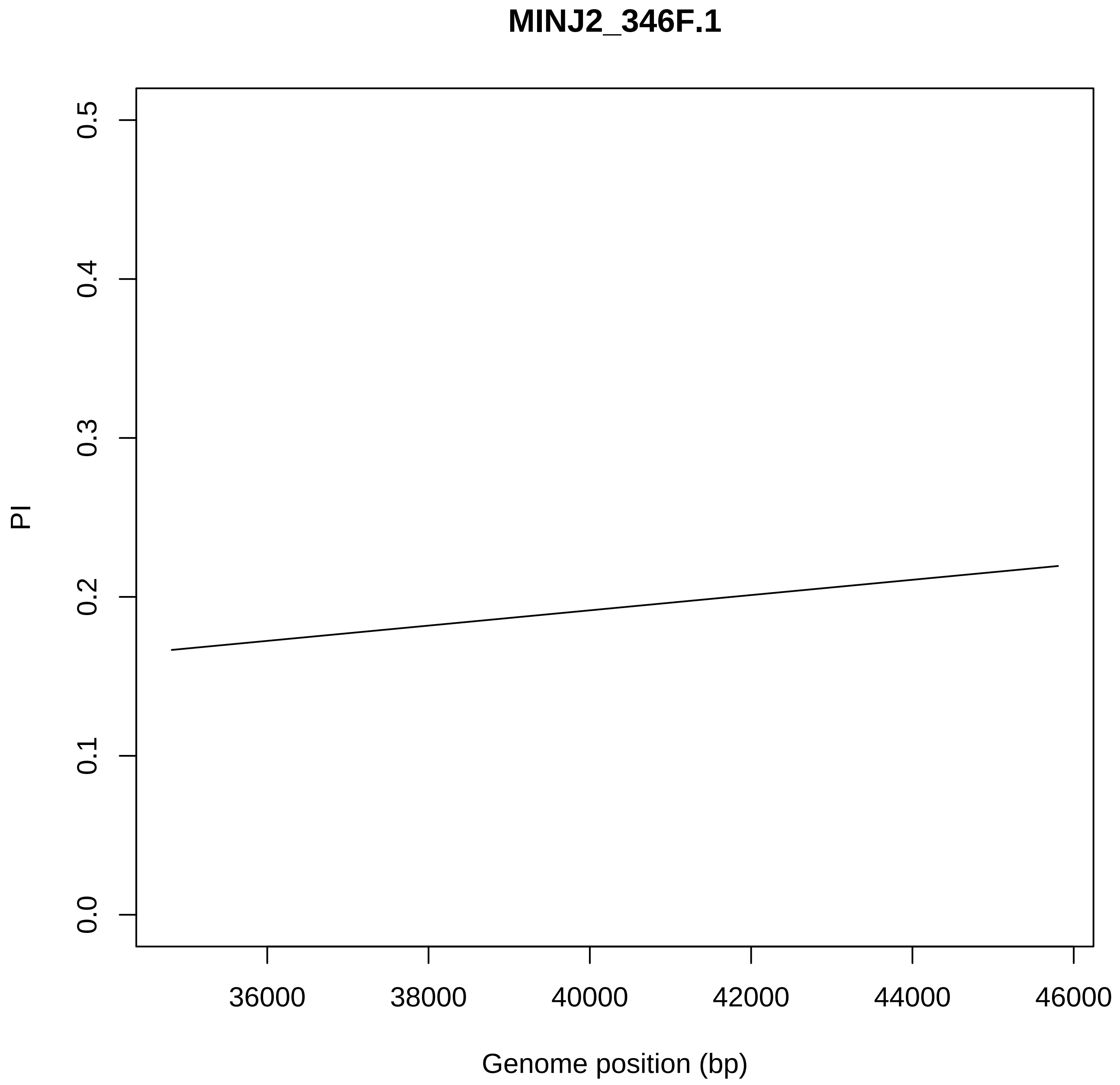
MINJ2_346F.1
PI
0.0
0.1
0.2
0.3
0.4
0.5
36000
38000
40000
42000
44000
46000
Genome position (bp)

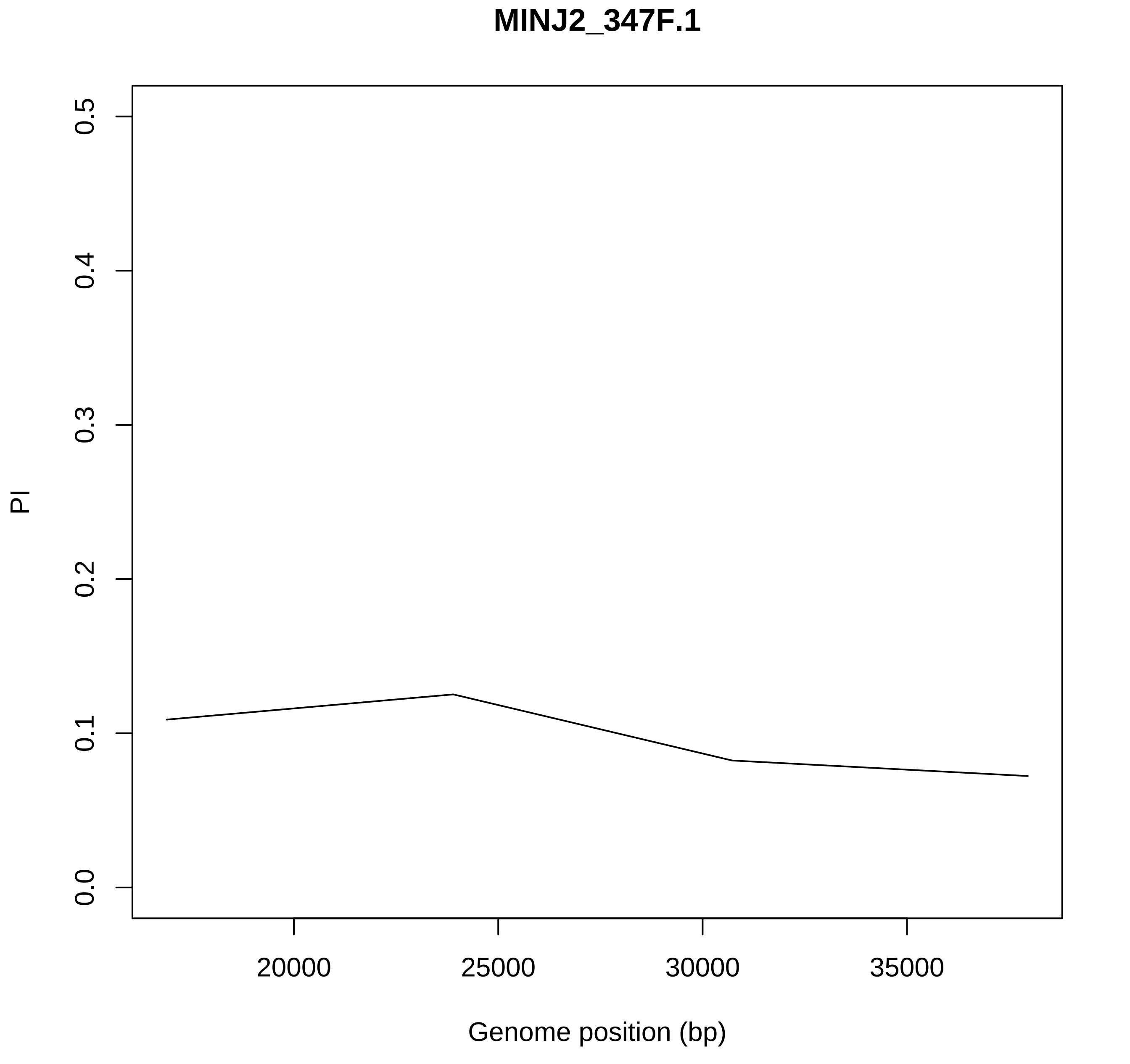
MINJ2_347F.1
PI
0.0
0.1
0.2
0.3
0.4
0.5
20000
25000
30000
35000
Genome position (bp)

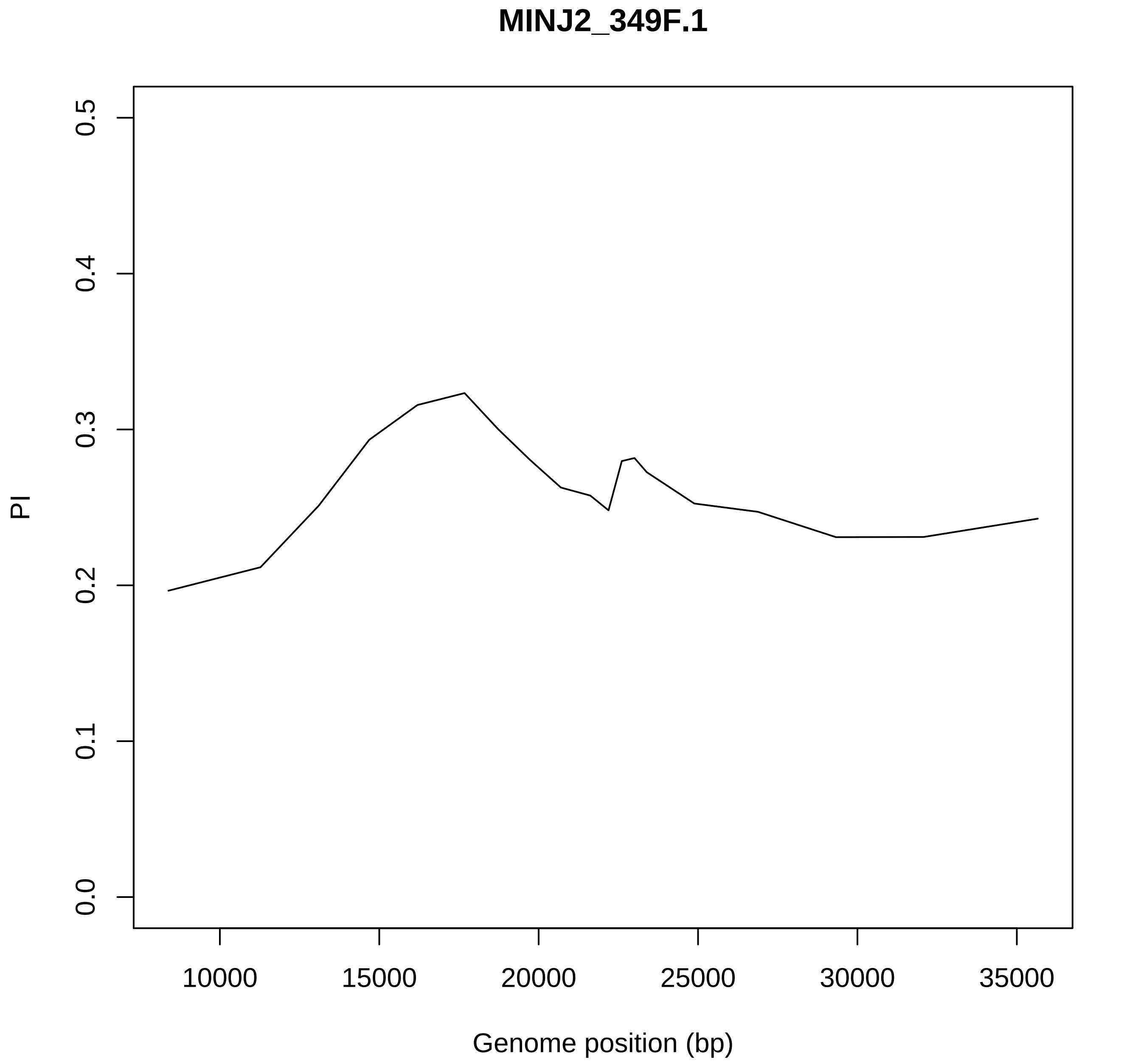

MINJ2_349F.1
PI
0.0
0.1
0.2
0.3
0.4
0.5
10000
15000
20000
25000
30000
35000
Genome position (bp)

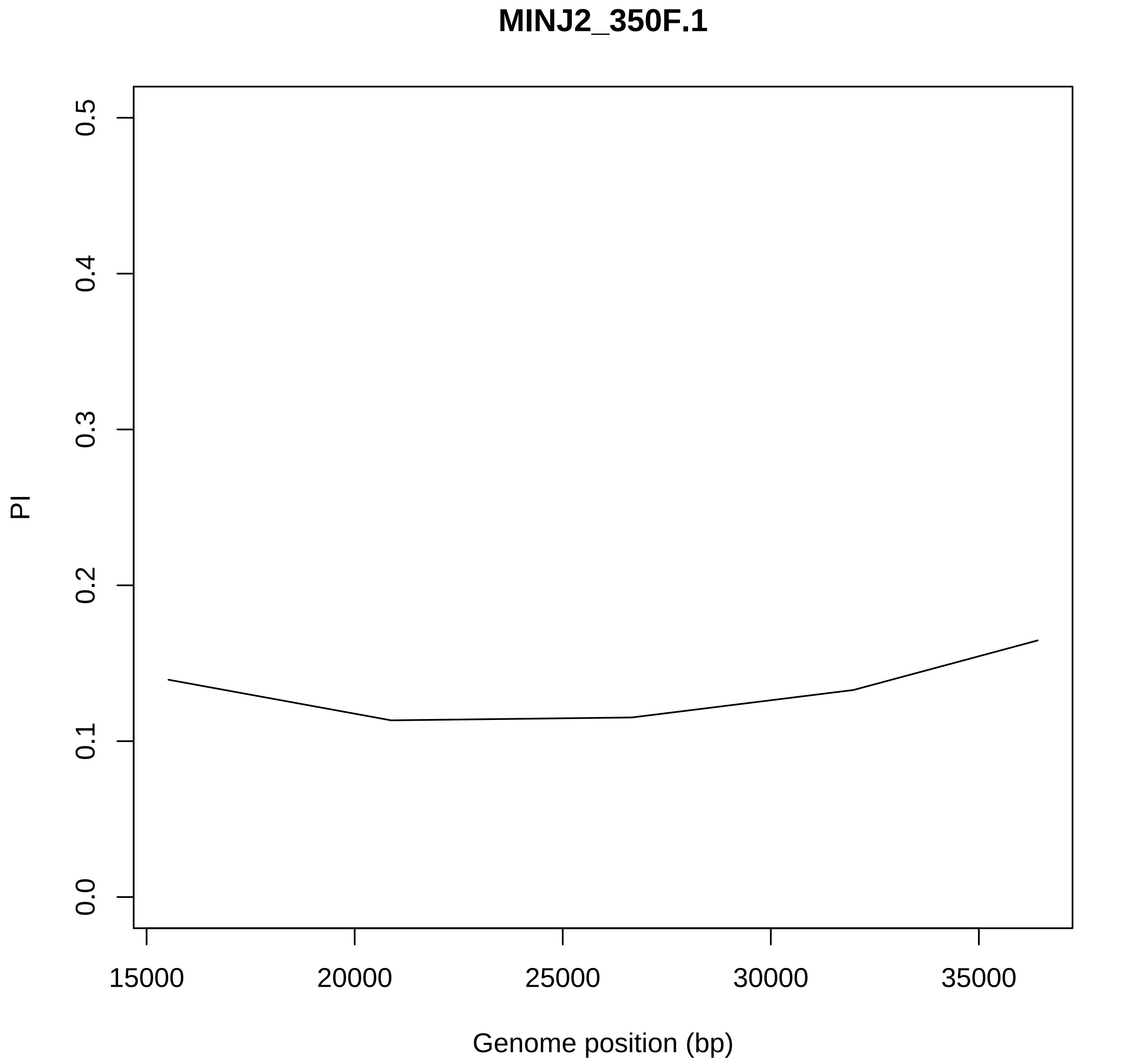

MINJ2_350F.1
PI
0.0
0.1
0.2
0.3
0.4
0.5
15000
20000
25000
30000
35000
Genome position (bp)

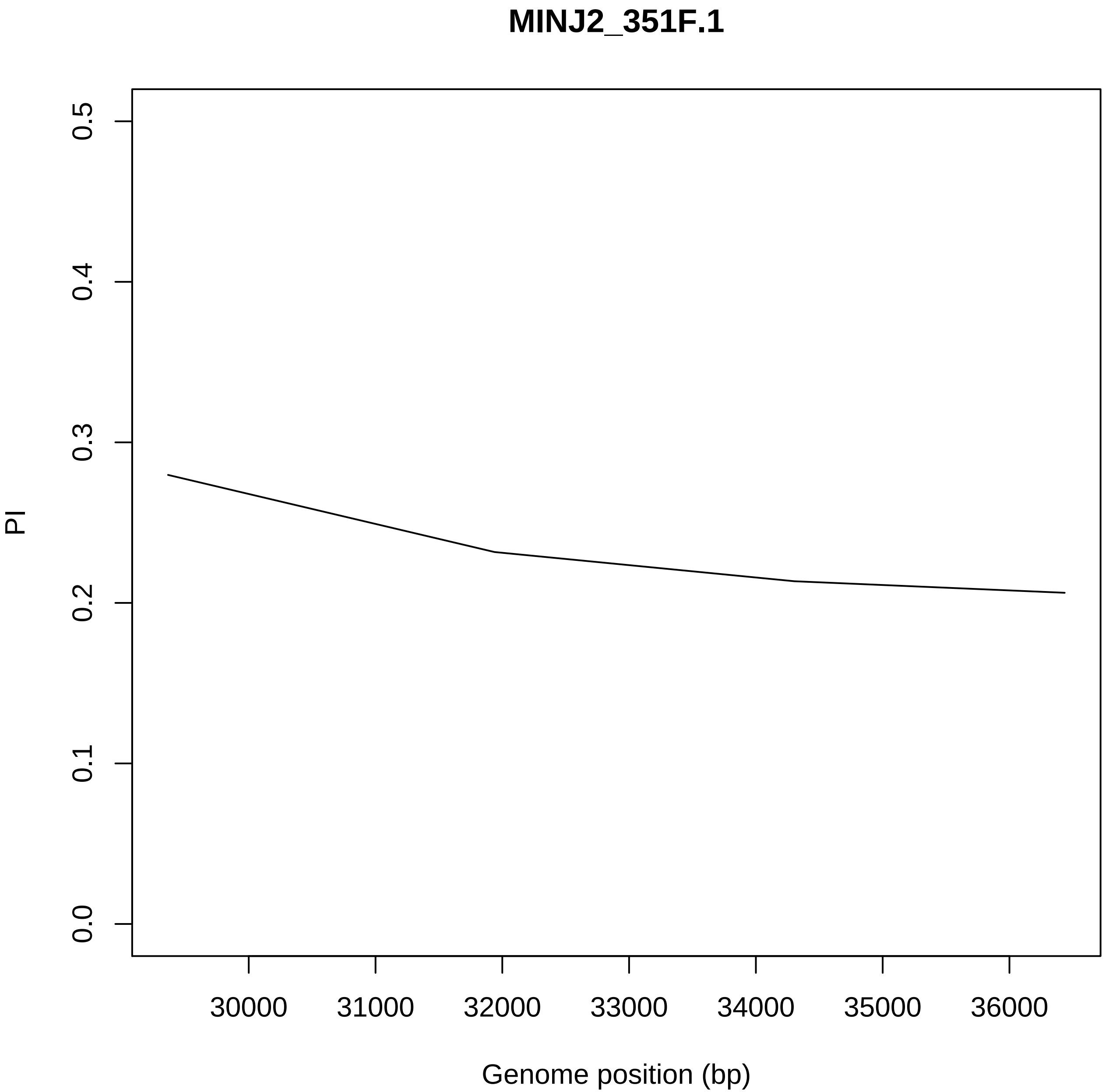

MINJ2_351F.1
PI
0.0
0.1
0.2
0.3
0.4
0.5
30000
31000
32000
33000
34000
35000
36000
Genome position (bp)

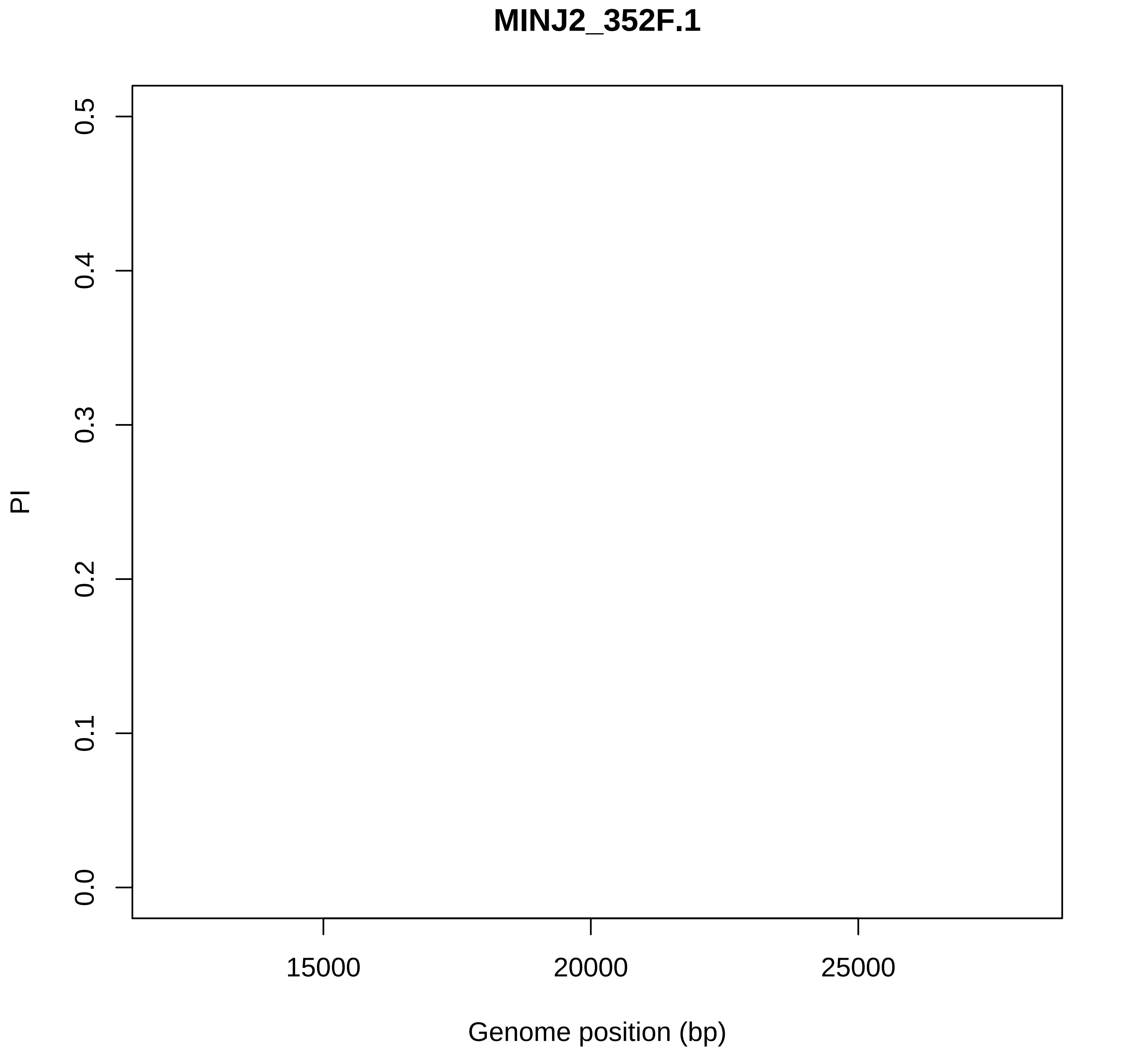
MINJ2_352F.1
PI
0.0
0.1
0.2
0.3
0.4
0.5
15000
20000
25000
Genome position (bp)

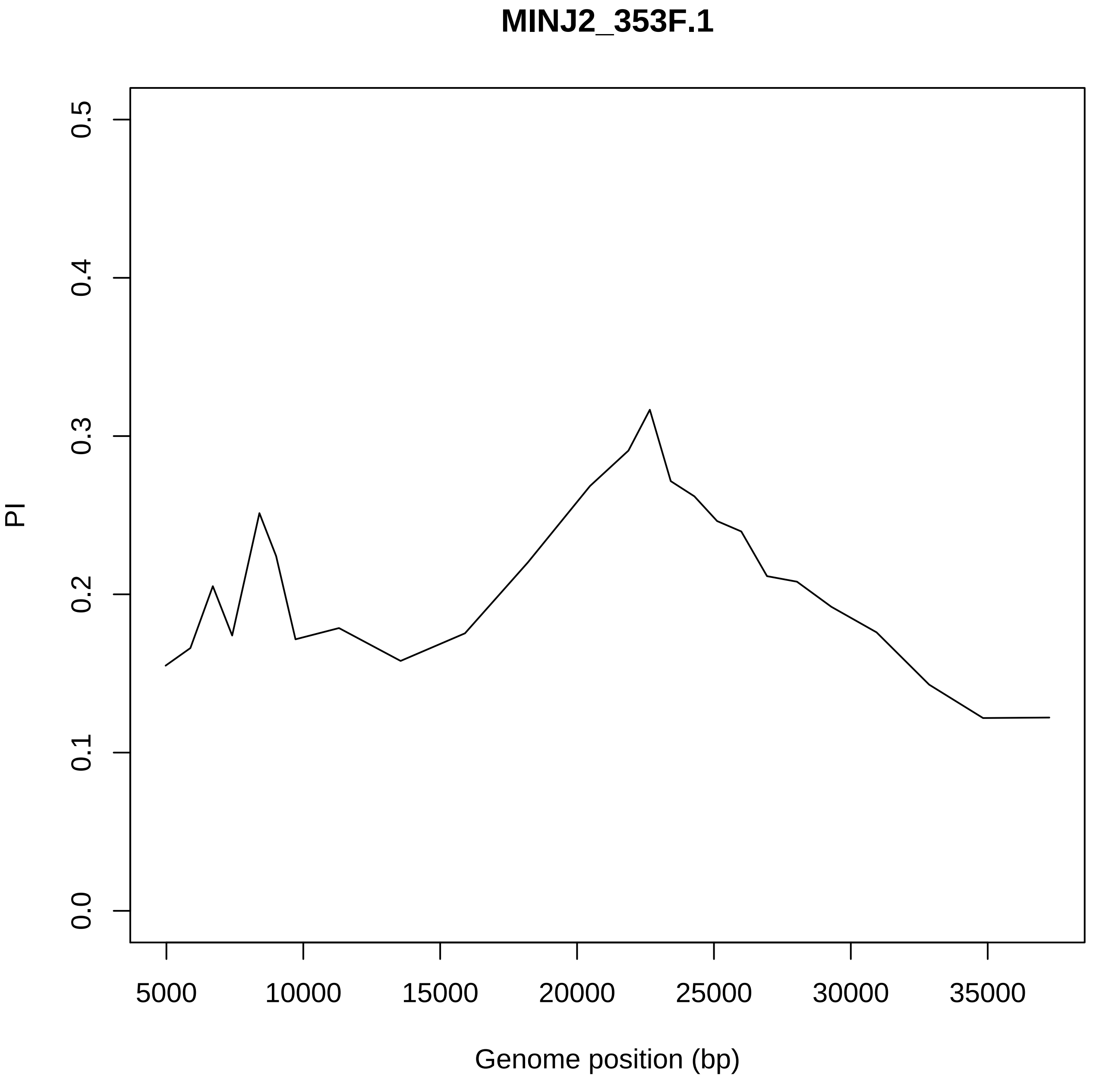
MINJ2_353F.1
PI
0.0
0.1
0.2
0.3
0.4
0.5
5000
10000
15000
20000
25000
30000
35000
Genome position (bp)

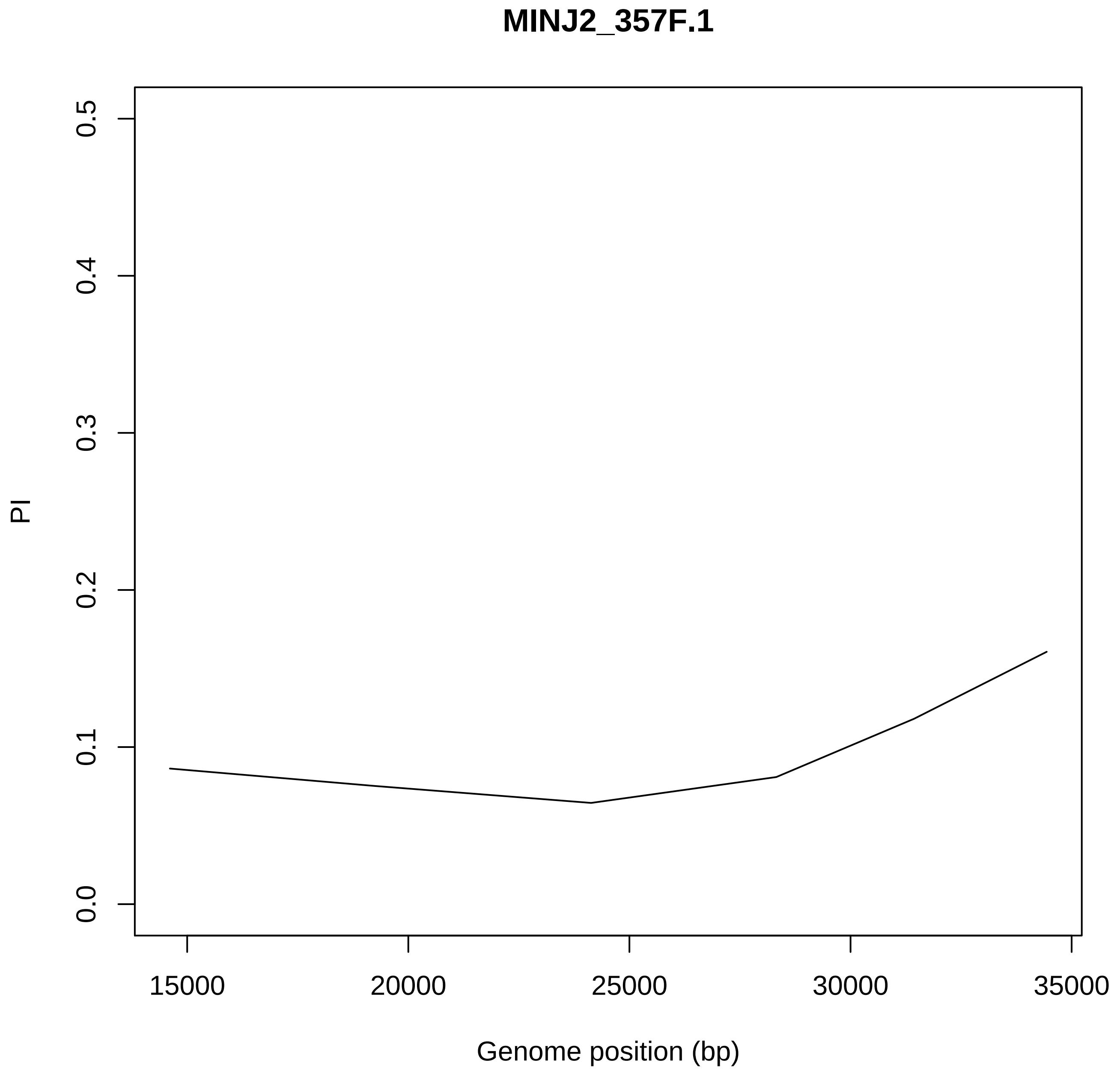

MINJ2_357F.1
PI
0.0
0.1
0.2
0.3
0.4
0.5
15000
20000
25000
30000
35000
Genome position (bp)

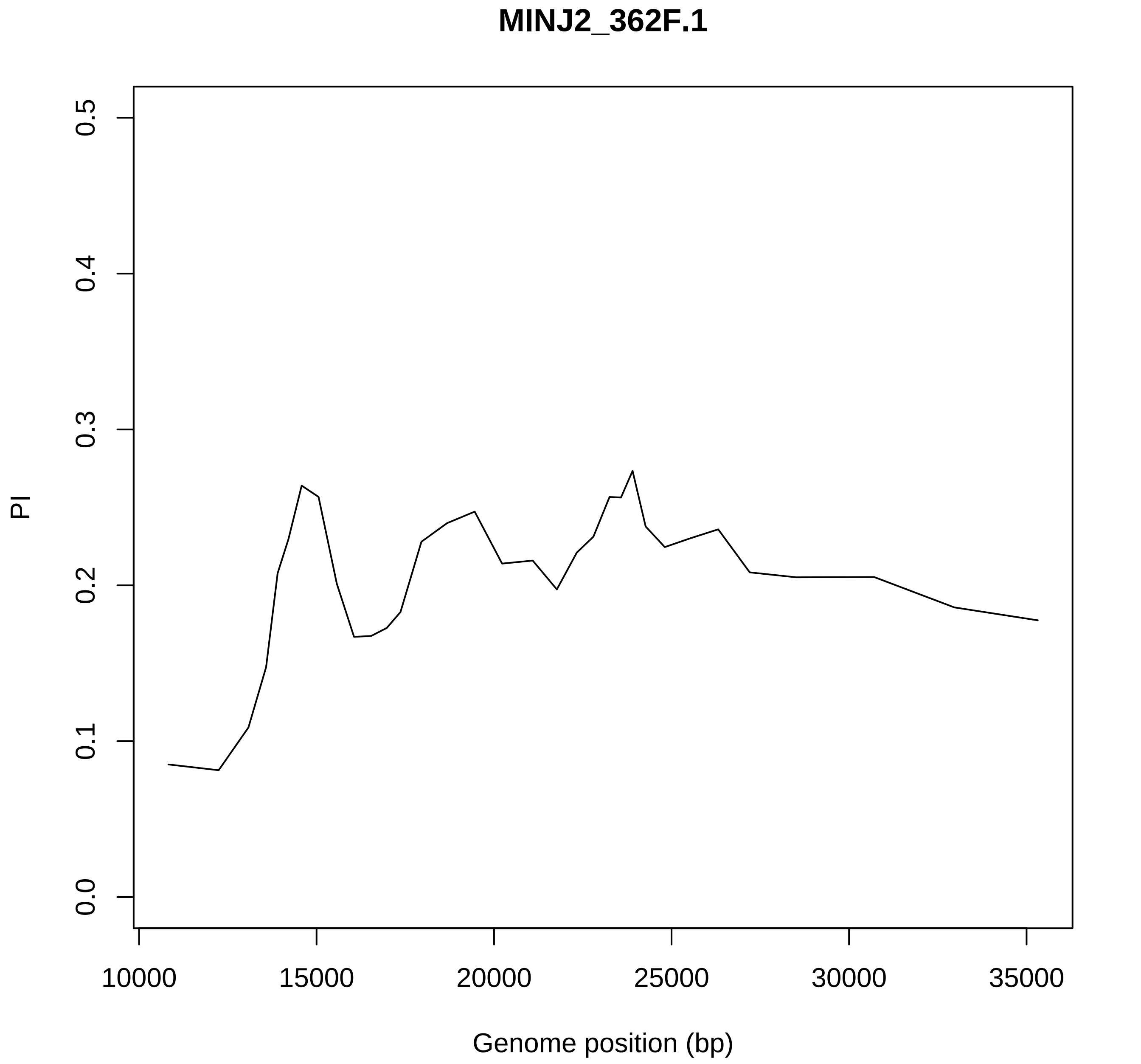

MINJ2_362F.1
PI
0.0
0.1
0.2
0.3
0.4
0.5
10000
15000
20000
25000
30000
35000
Genome position (bp)

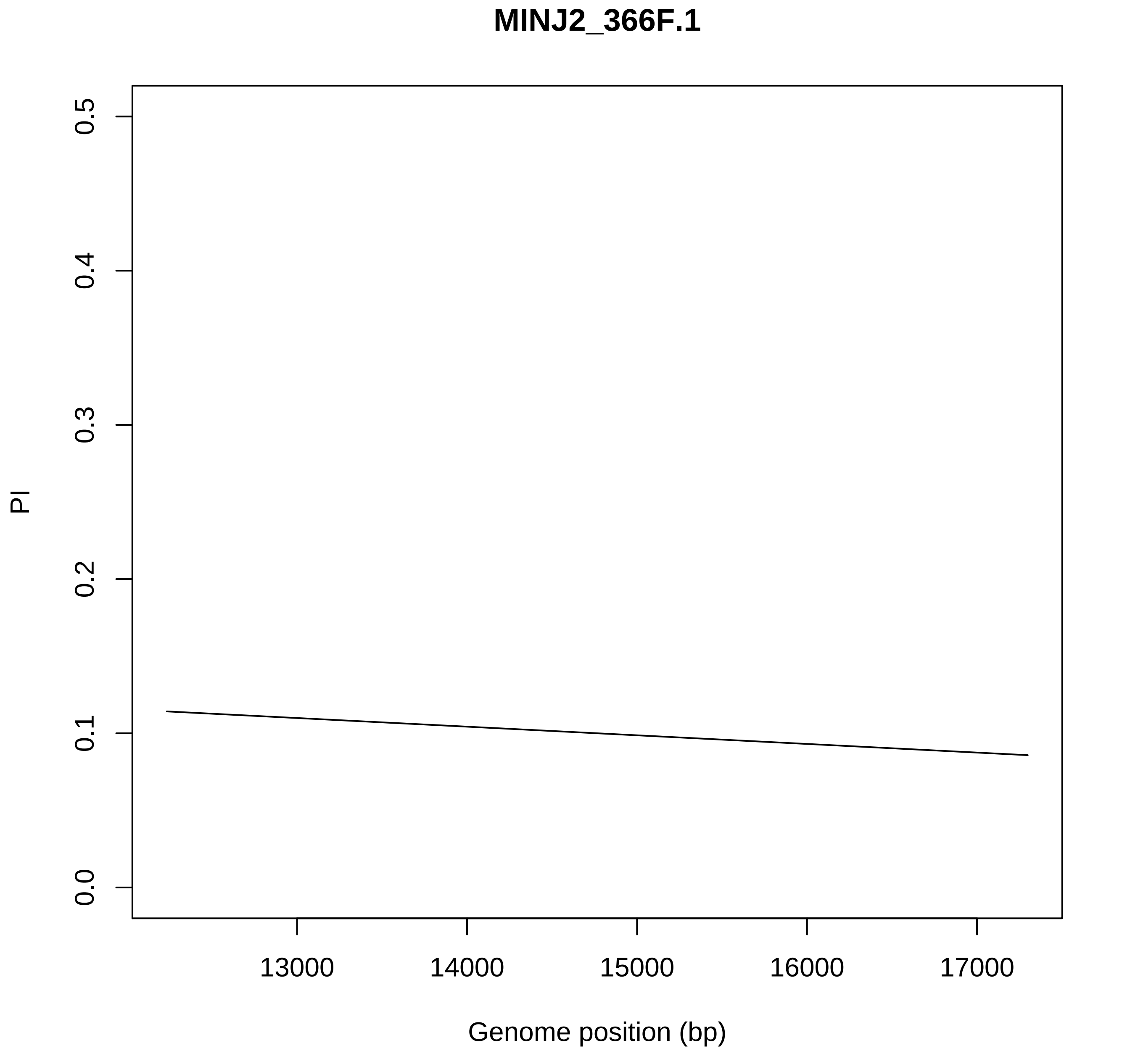
MINJ2_366F.1
PI
0.0
0.1
0.2
0.3
0.4
0.5
13000
14000
15000
16000
17000
Genome position (bp)

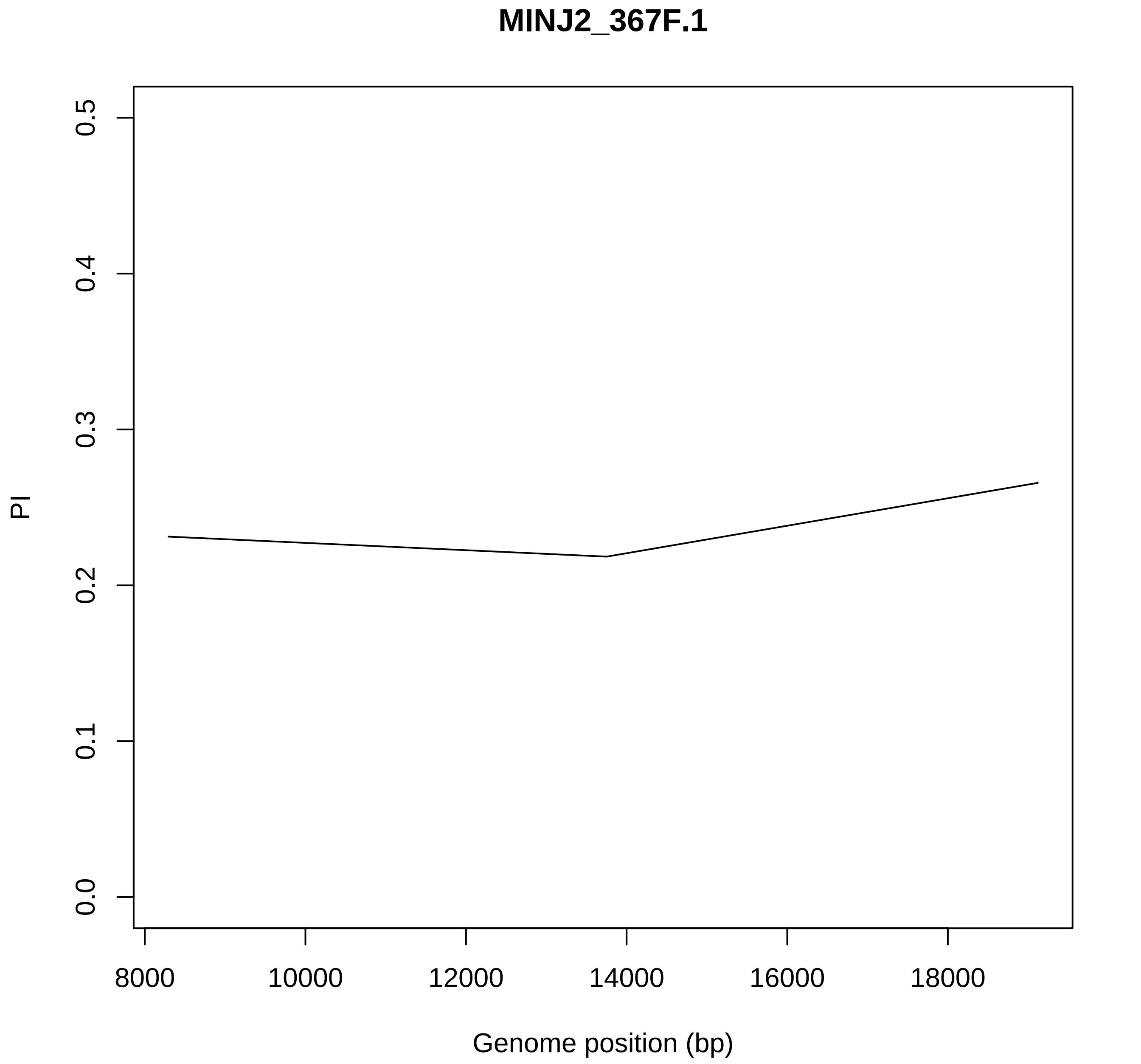
MINJ2_367F.1
PI
0.0
0.1
0.2
0.3
0.4
0.5
8000
10000
12000
14000
16000
18000
Genome position (bp)

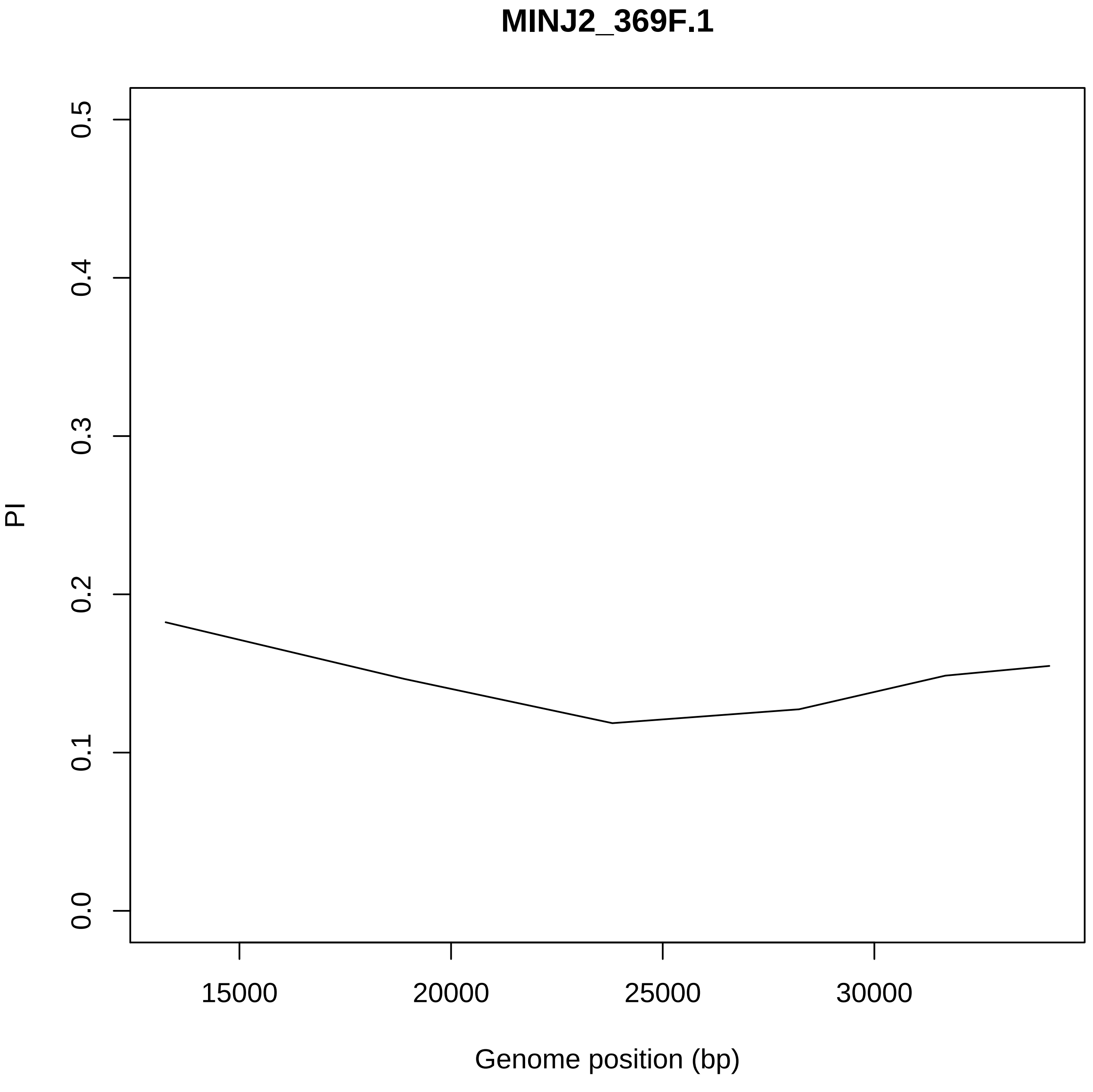

MINJ2_369F.1
PI
0.0
0.1
0.2
0.3
0.4
0.5
15000
20000
25000
30000
Genome position (bp)

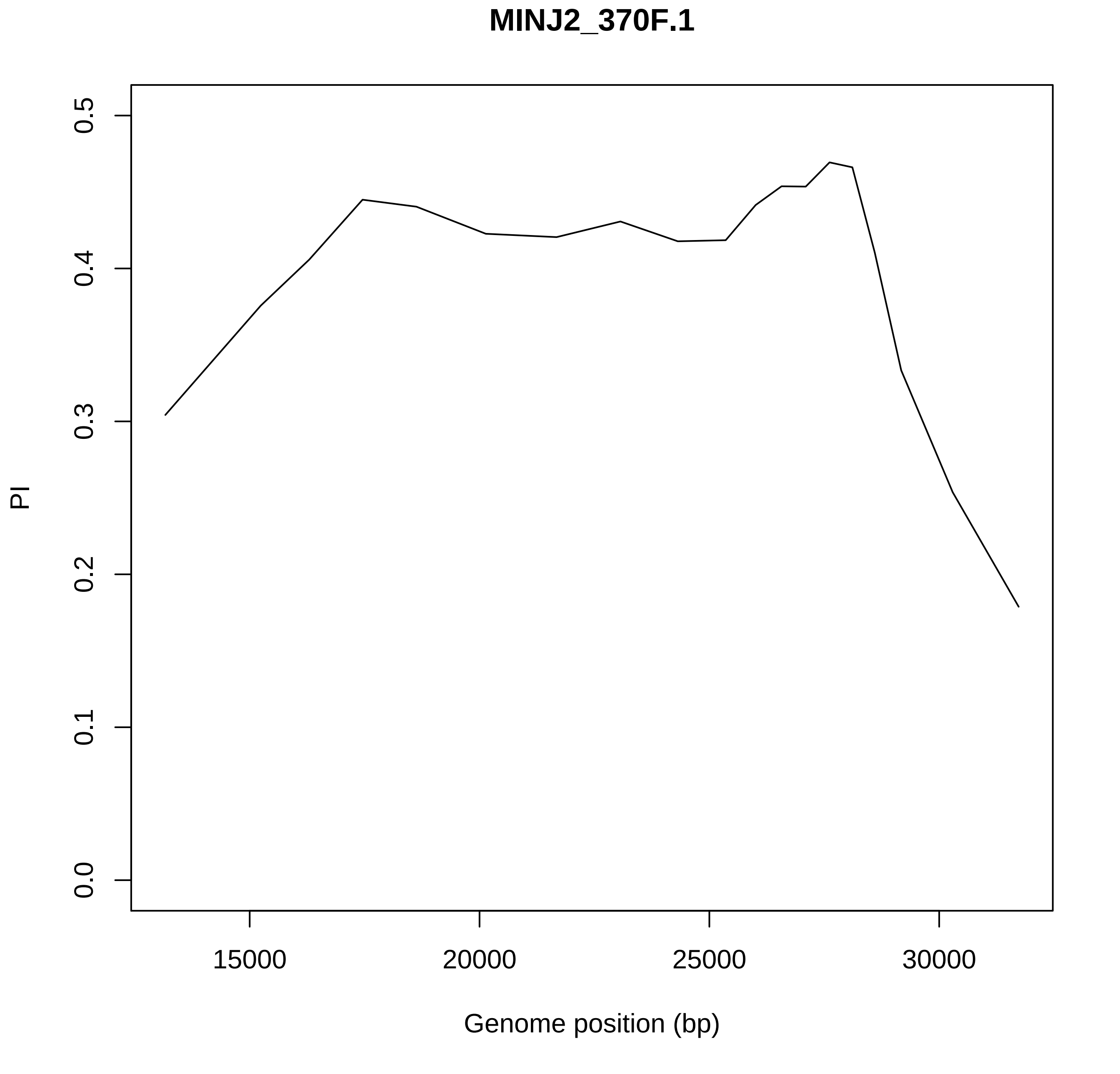

MINJ2_370F.1
PI
0.0
0.1
0.2
0.3
0.4
0.5
15000
20000
25000
30000
Genome position (bp)

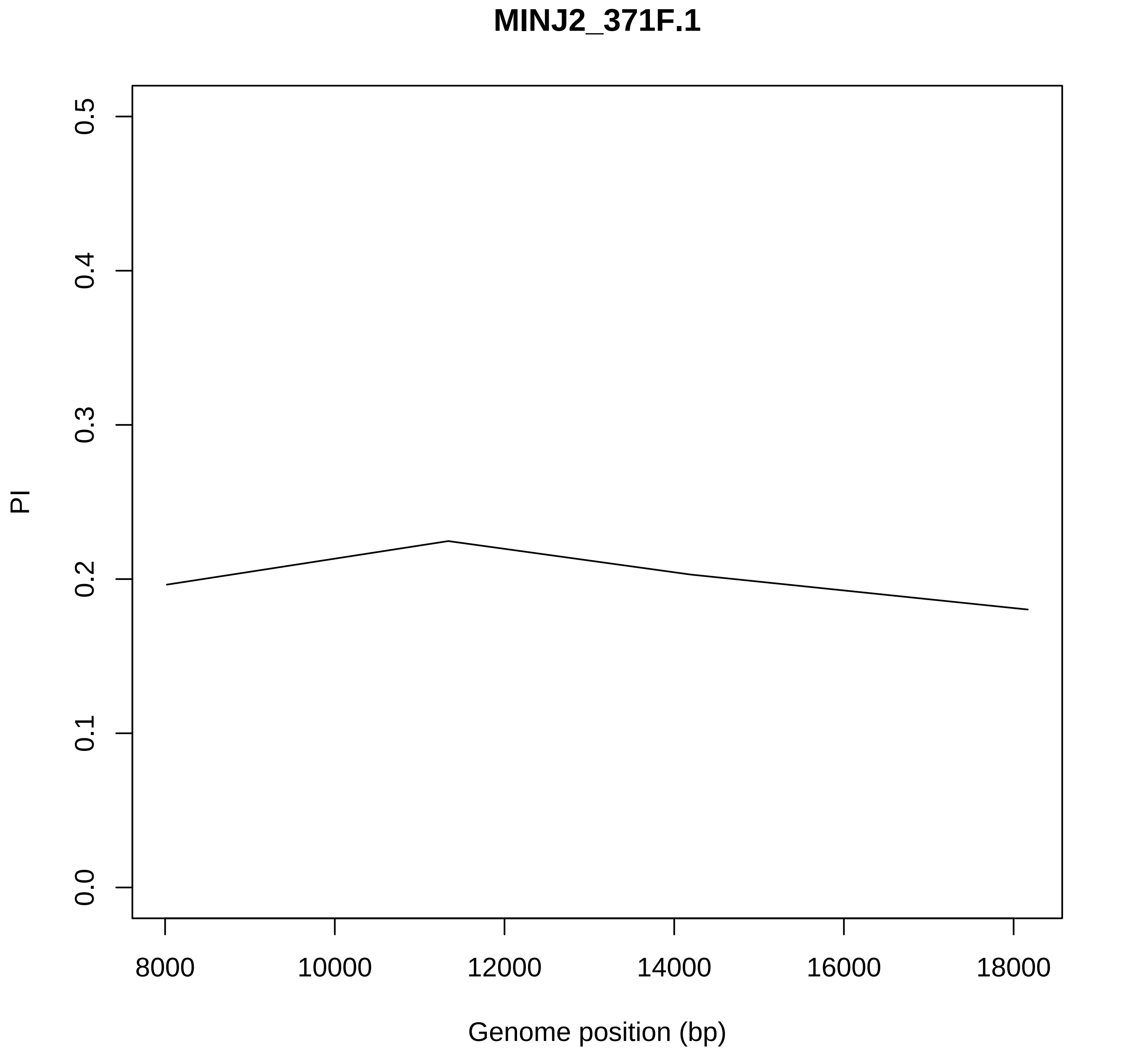
MINJ2_371F.1
PI
0.0
0.1
0.2
0.3
0.4
0.5
8000
10000
12000
14000
16000
18000
Genome position (bp)

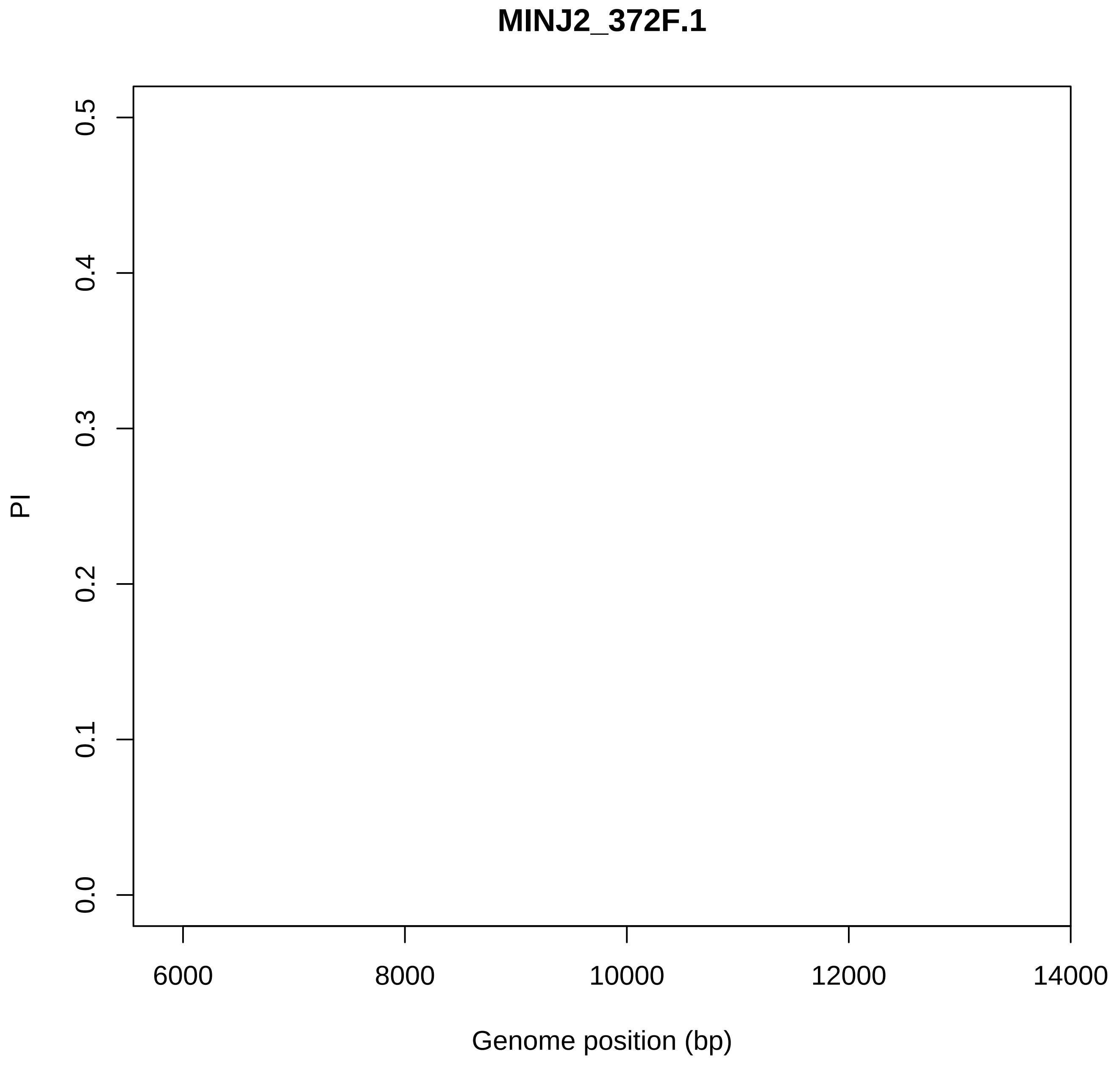
MINJ2_372F.1
PI
0.0
0.1
0.2
0.3
0.4
0.5
6000
8000
10000
12000
14000
Genome position (bp)

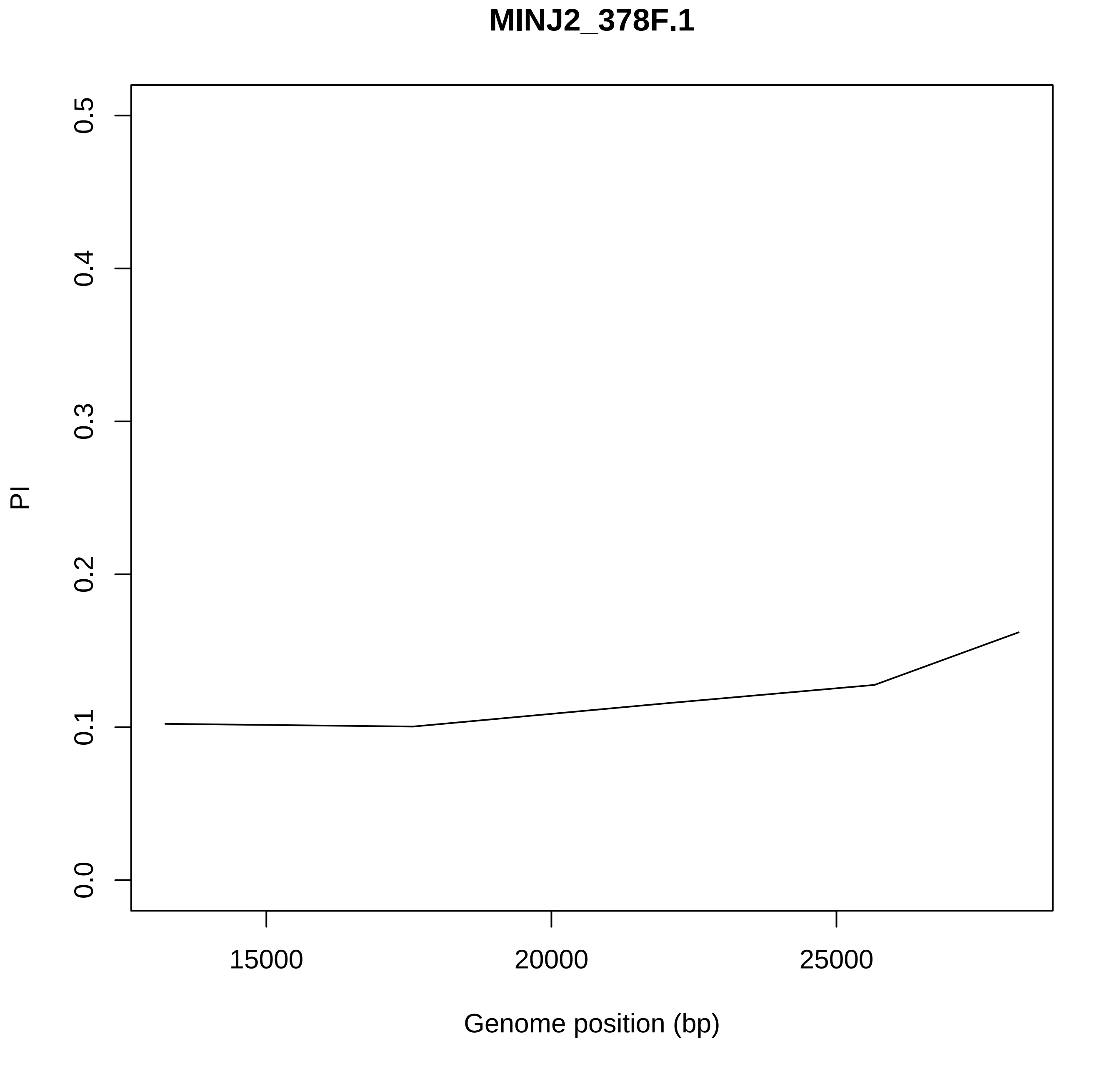
MINJ2_378F.1
0.0
0.1
0.2
0.3
0.4
0.5
PI
15000
20000
25000
Genome position (bp)

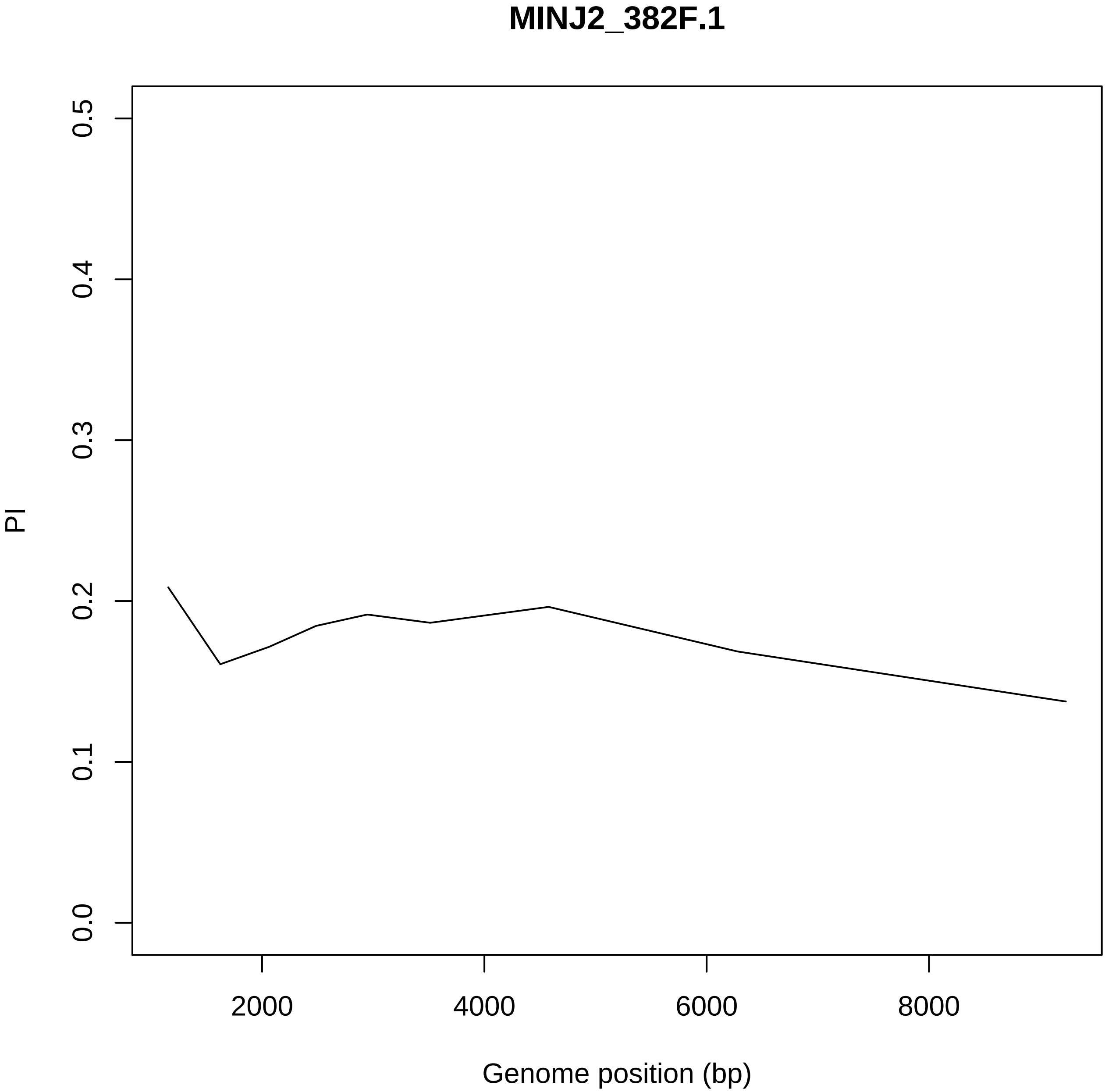
MINJ2_382F.1
PI
0.0
0.1
0.2
0.3
0.4
0.5
2000
4000
6000
8000
Genome position (bp)

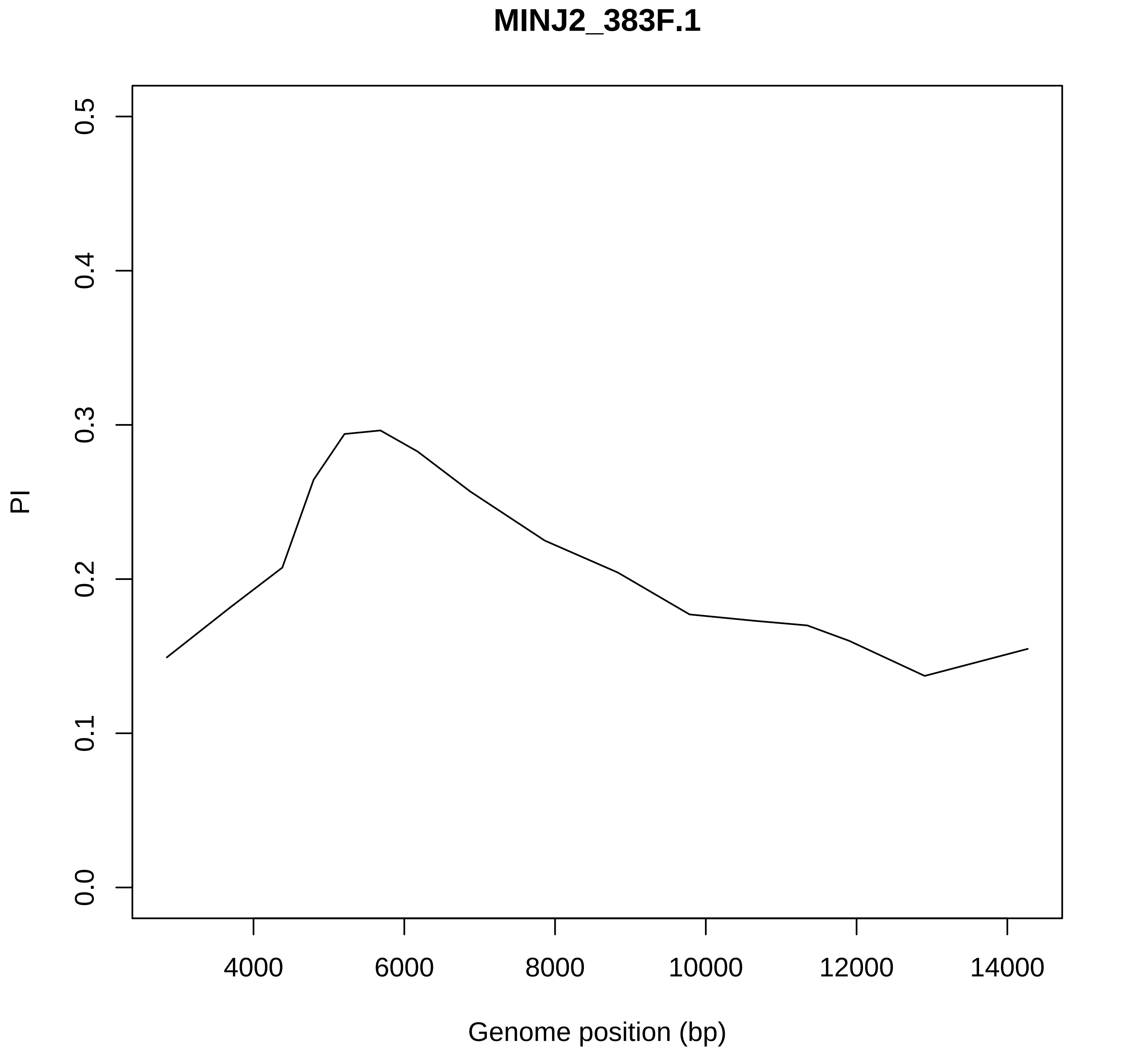

MINJ2_383F.1
PI
0.0
0.1
0.2
0.3
0.4
0.5
4000
6000
8000
10000
12000
14000
Genome position (bp)

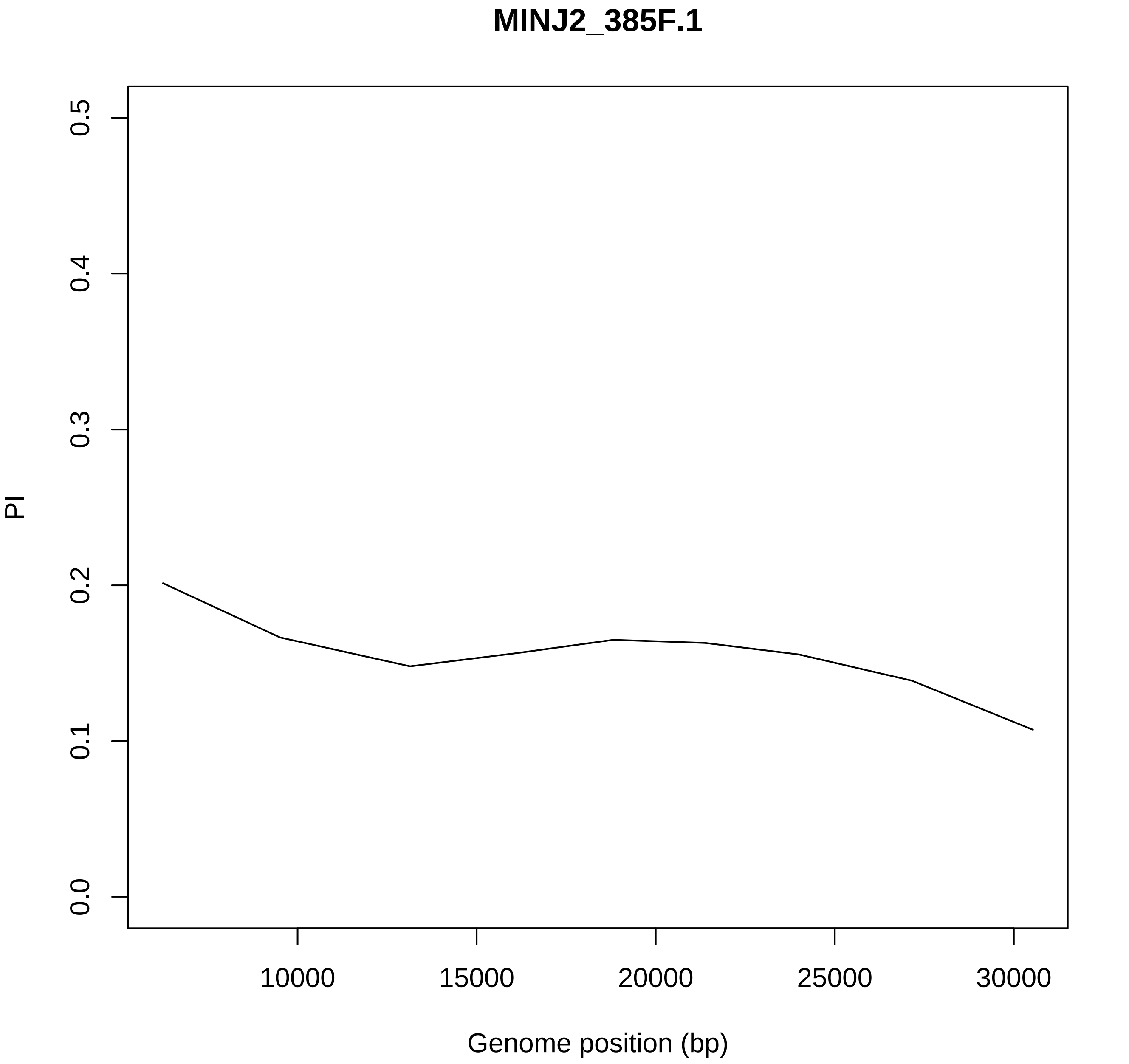

MINJ2_385F.1
PI
0.0
0.1
0.2
0.3
0.4
0.5
10000
15000
20000
25000
30000
Genome position (bp)

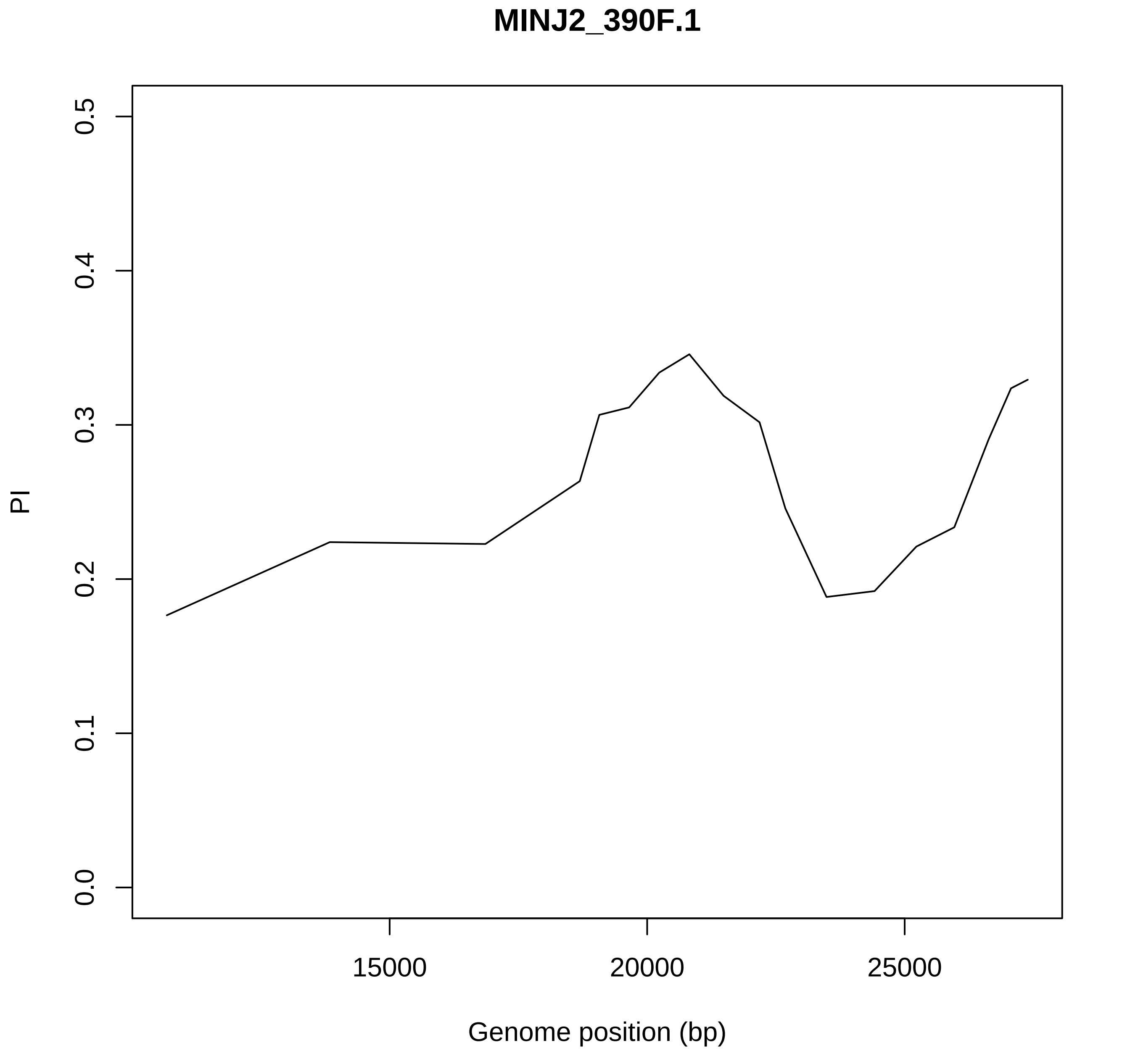
MINJ2_390F.1
PI
0.0
0.1
0.2
0.3
0.4
0.5
15000
20000
25000
Genome position (bp)

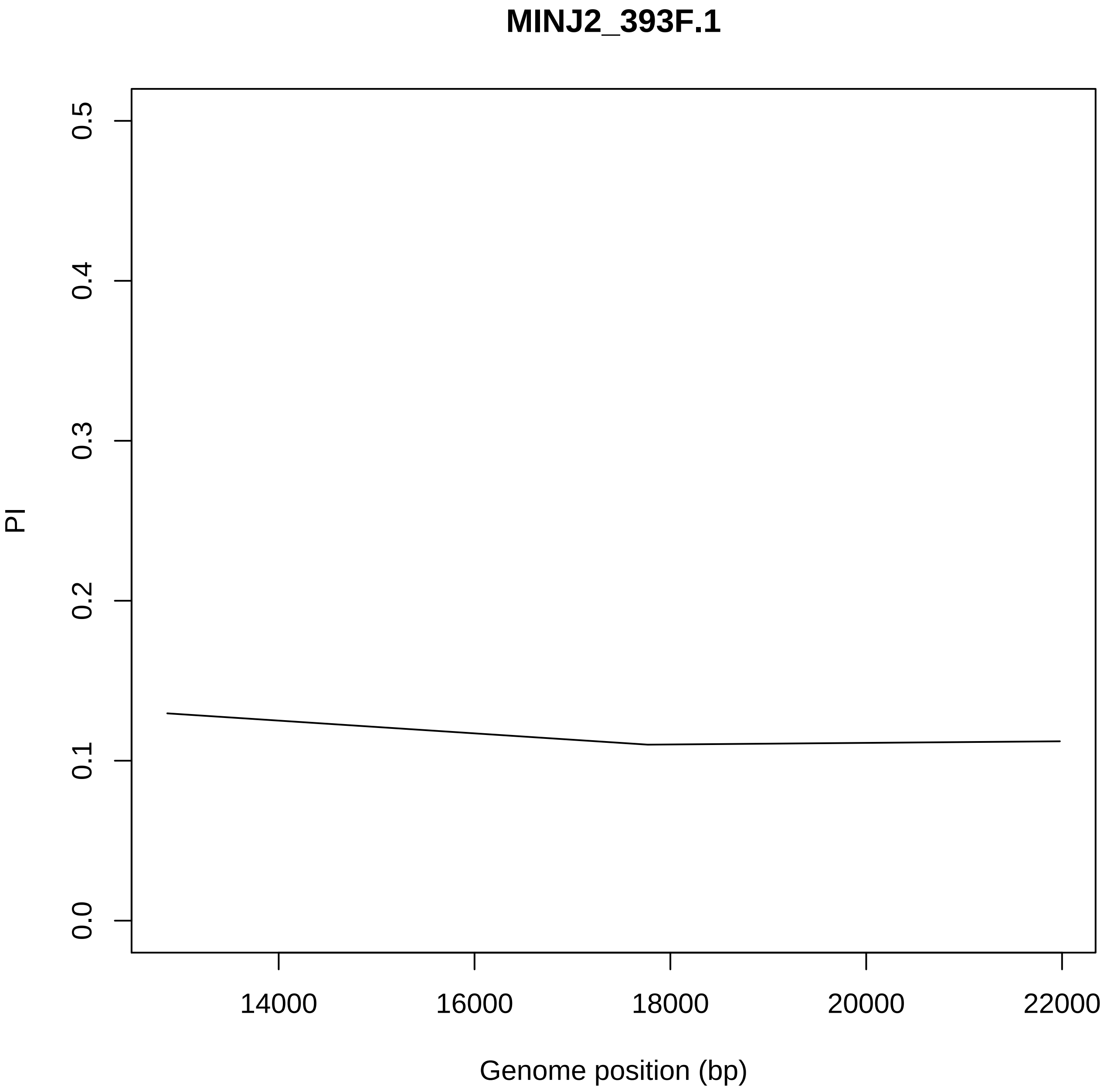

MINJ2_393F.1
PI
0.0
0.1
0.2
0.3
0.4
0.5
14000
16000
18000
20000
22000
Genome position (bp)

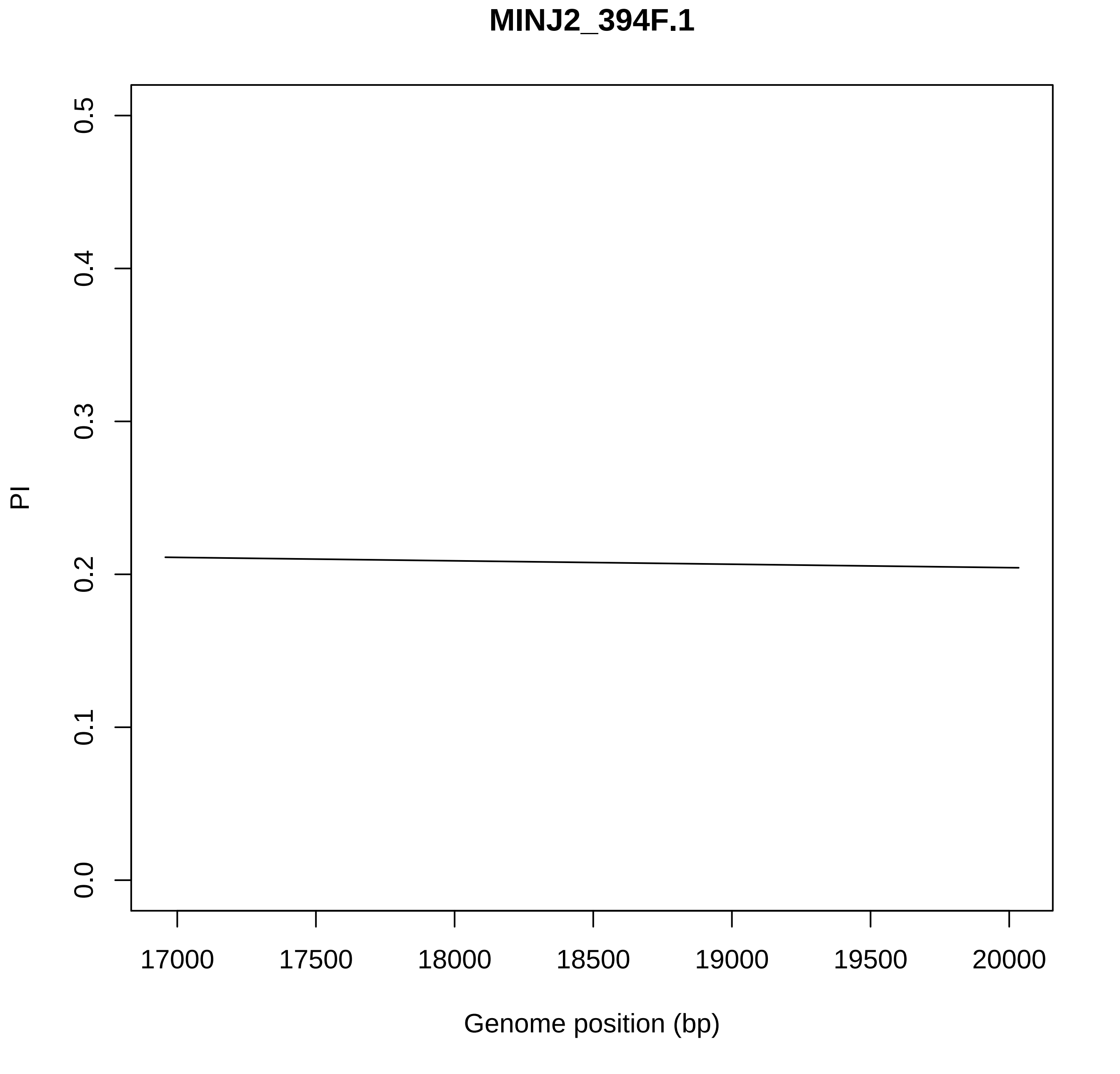
MINJ2_394F.1
PI
0.0
0.1
0.2
0.3
0.4
0.5
17000
17500
18000
18500
19000
19500
20000
Genome position (bp)

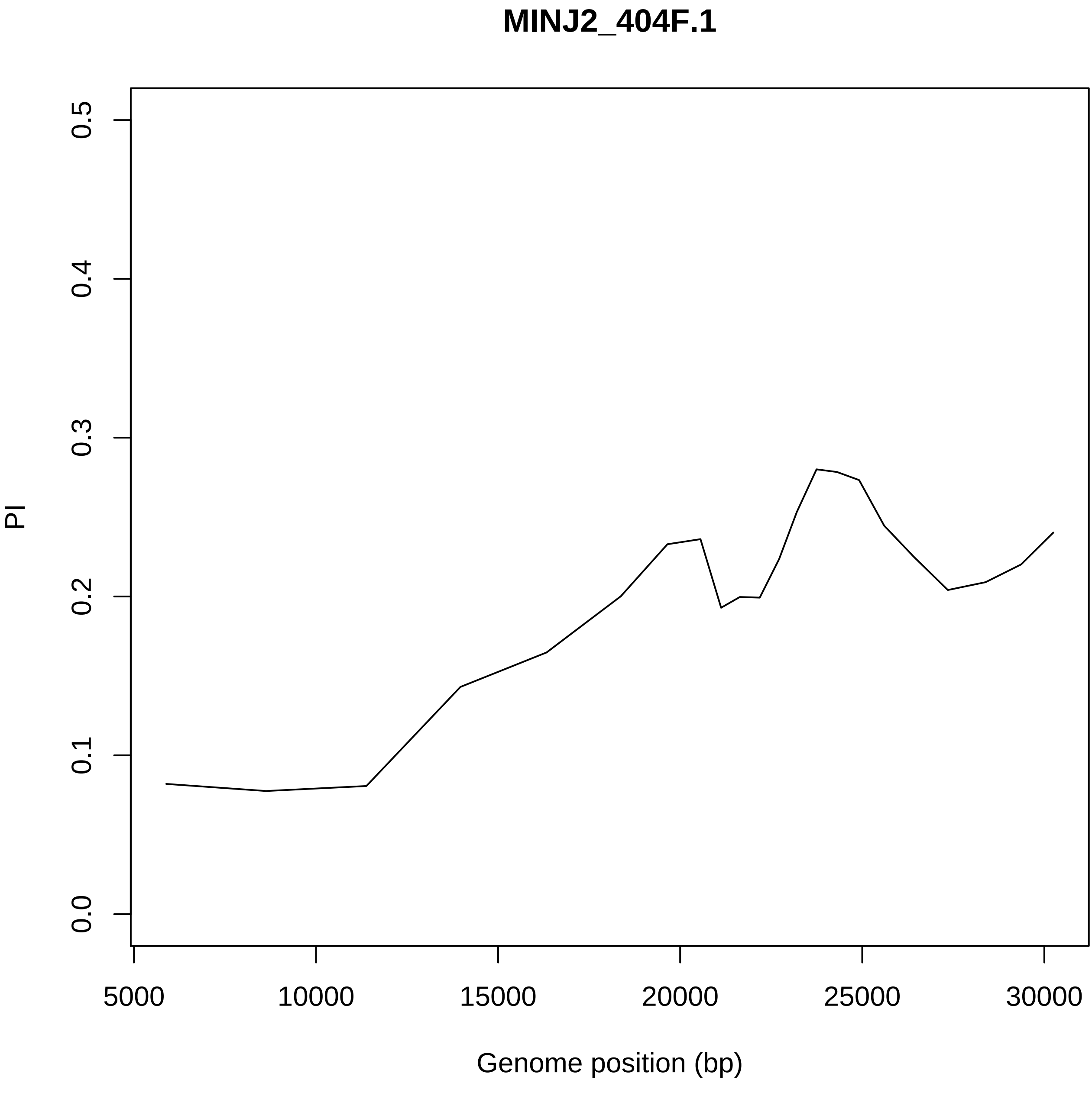

MINJ2_404F.1
PI
0.0
0.1
0.2
0.3
0.4
0.5
5000
10000
15000
20000
25000
30000
Genome position (bp)

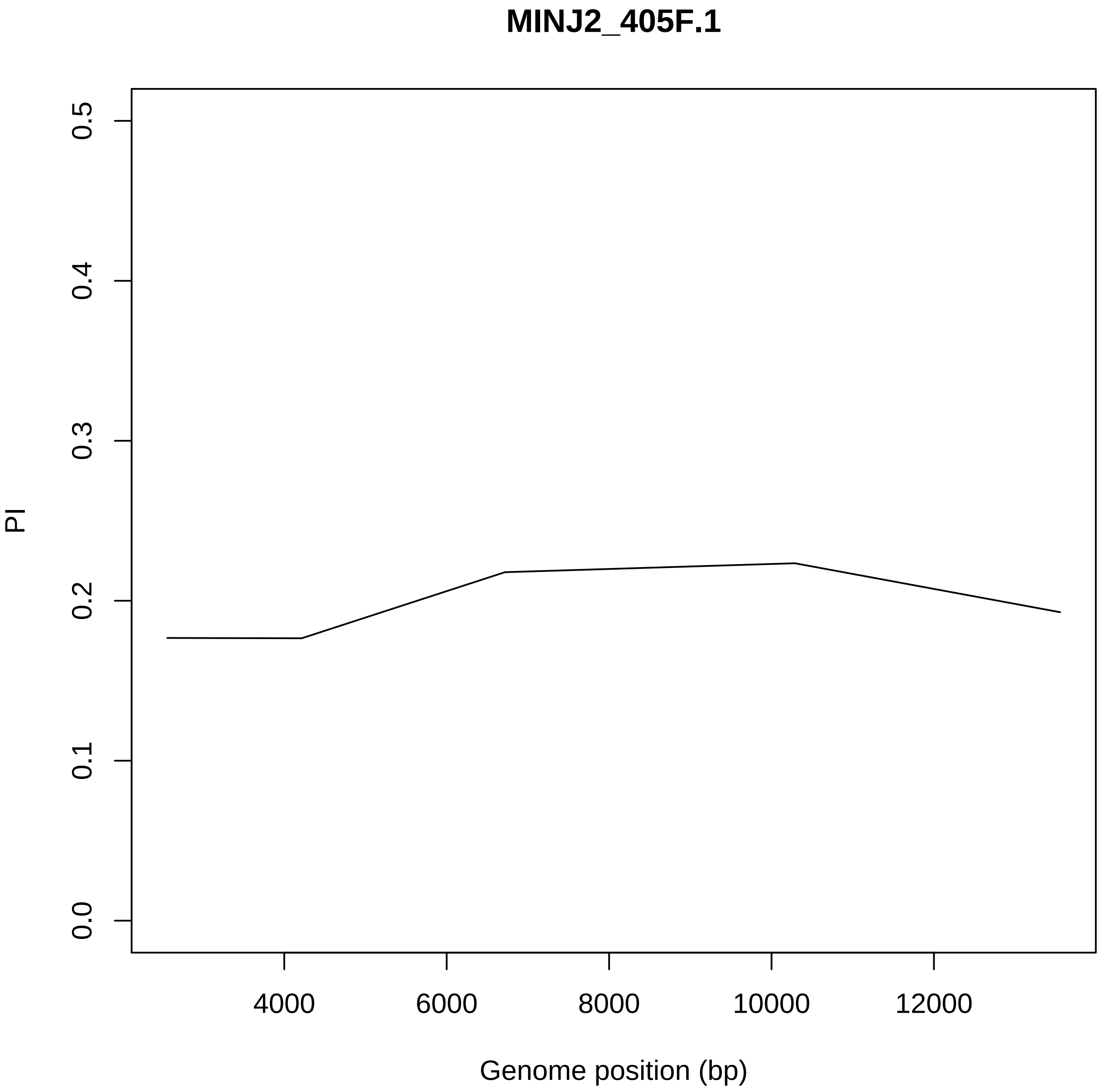
MINJ2_405F.1
PI
0.0
0.1
0.2
0.3
0.4
0.5
4000
6000
8000
10000
12000
Genome position (bp)
