# Supplementary material for: Root‐knot nematode genetic diversity associated with host compatibility to sweetpotato cultivars
Source: Mol Plant Pathol. 2020 Jun 17;21(8):1088–98. doi: 10.1111/mpp.12961 (PMC7368124; doi:10.1111/mpp.12961)
Supplement: Supplementary file 7 — TABLE S1 Summary of Illumina short read sequences accumulated for each isolate [file MPP-21-1088-s007.pdf]

Table S1 Summary of Illumina short read sequences accumulated for each isolate.			
File name	Total Sequences	Sequence length (Mb)	Covrerage (folds)
Chb_Ash001_sorted.bam	12,014,704	1,213	6.742
Chb_Tgn001_sorted.bam	21,406,488	2,162	12.011
Chb_Ycmt001_sorted.bam	7,537,184	761	4.229
Fkok_Hkck001_sorted.bam	12,381,872	1,251	6.948
Hkd_Mr002_sorted.bam	6,175,758	624	3.465
Hkd_Mr003_sorted.bam	10,698,408	1,081	6.003
Ibrk_Tkb002_sorted.bam	16,333,612	1,650	9.165
Kch_Smnt001_sorted.bam	12,616,870	1,274	7.079
Kch_Ts001_sorted.bam	5,600,580	566	3.143
Kmmt_Gs001_sorted.bam	12,690,012	1,282	7.121
Kmmt_Gs003_sorted.bam	6,617,064	668	3.713
Kmmt_Gs004_sorted.bam	13,057,408	1,319	7.327
Kmmt_Gs005_sorted.bam	11,833,550	1,195	6.640
Kmmt_Gs006_sorted.bam	13,350,836	1,348	7.491
Kmmt_Gs008_sorted.bam	13,230,550	1,336	7.424
Kmmt_Gs009_sorted.bam	16,093,708	1,625	9.030
Kmmt_Gs010_sorted.bam	16,461,154	1,663	9.237
Kmmt_Msk001_sorted.bam	8,333,892	842	4.676
Kmmt_Msk002_sorted.bam	7,493,280	757	4.205
Kmmt_Nshr001_sorted.bam	22,172,130	2,239	12.441
Kmmt_Ymg001_sorted.bam	9,474,498	957	5.316
Kmmt_Ymg002_sorted.bam	10,482,164	1,059	5.882
Mie_Ykic001_sorted.bam	13,568,444	1,370	7.613
Myzk_Hyg001_sorted.bam	10,387,508	1,049	5.829
Myzk_Myknj001_sorted.bam	14,066,834	1,421	7.893
Myzk_Tn001_sorted.bam	13,101,480	1,323	7.351
Oit_Tkd001_sorted.bam	15,034,848	1,519	8.436
Oknw_Isgk001_sorted.bam	11,583,748	1,170	6.500
Oknw_Isgk002_sorted.bam	13,068,690	1,320	7.333
Oknw_Ogm001_sorted.bam	17,129,292	1,730	9.611
Oknw_Ynsr001_sorted.bam	15,118,078	1,527	8.483
Unkwn_Unkwn001_sorted.bam	16,642,454	1,681	9.338
Ymgt_Unkwn001_sorted.bam	11,167,234	1,128	6.266
Ymgt_Unkwn002_sorted.bam	13,032,294	1,316	7.313
Hkd_Mr001_2_S1_sorted.bam	20,151,450	1,532	8.508
Ibrk_Chks001_S7_sorted.bam	7,462,080	567	3.151
Kgsm_Ky001_S12_sorted.bam	14,291,998	1,086	6.034
Kgsm_Ngsm001_S13_sorted.bam	20,354,062	1,547	8.594
Kgsm_Nktn001_S14_sorted.bam	13,163,074	1,000	5.558
Kmmt_Gs002_2_S4_sorted.bam	11,040,030	839	4.661
Kmmt_Gs007_2_S5_sorted.bam	13,643,518	1,037	5.761
Kmmt_Ots001_2_S6_sorted.bam	7,240,764	550	3.057
Kmmt_Shr001_S11_sorted.bam	11,098,378	843	4.686
Mie_Mtzk001_2_S3_sorted.bam	8,992,728	683	3.797
Ngsk_Ssb001_S10_sorted.bam	41,380,710	3,145	17.472
Ngt_Kjkw001_2_S2_sorted.bam	30,362,490	2,308	12.820
Shzok_Shmz001_S9_sorted.bam	19,905,550	1,513	8.405
Ymns_Malps001_S8_sorted.bam	5,451,728	414	2.302
Mean	13,635,316	1,260	7.001
